# The Role of Serine/Threonine-Specific Protein Kinases in Cyanobacteria - SpkB Is Involved in Acclimation to Fluctuating Conditions in *Synechocystis* sp. PCC 6803

**DOI:** 10.1016/j.mcpro.2023.100656

**Published:** 2023-10-04

**Authors:** Thomas Barske, Philipp Spät, Hendrik Schubert, Peter Walke, Boris Maček, Martin Hagemann

**Affiliations:** 1 Department of Plant Physiology, Institute of Biosciences, University of Rostock, Rostock, Germany; 2 Department of Organismic Interactions, Interfaculty Institute of Microbiology and Infection Medicine Tübingen, University of Tübingen, Tübingen, Germany; 3 Department of Quantitative Proteomics, Interfaculty Institute for Cell Biology, University of Tübingen, Tübingen, Germany; 4 Department of Aquatic Ecology, Institute of Biosciences, University of Rostock, Rostock, Germany; 5 Interdisciplinary Faculty, Department Life, Light and Matter, University of Rostock, Rostock, Germany

**Keywords:** CO_2_, H_2_O_2_, inorganic carbon, protein phosphorylation, stress, cyanobacteria, protein kinase

## Abstract

Protein phosphorylation *via* serine/threonine protein kinases (Spk) is a widespread mechanism to adjust cellular processes toward changing environmental conditions. To study their role(s) in cyanobacteria, we investigated a collection of 11 completely segregated *spk* mutants among the 12 annotated Spks in the model cyanobacterium *Synechocystis* sp. PCC 6803. Screening of the mutant collection revealed that especially the mutant defective in SpkB encoded by *slr1697* showed clear deviations regarding carbon metabolism, that is, reduced growth rates at low CO_2_ or in the presence of glucose, and different glycogen accumulation patterns compared to WT. Alterations in the proteome of Δ*spkB* indicated changes of the cell surface but also metabolic functions. A phospho-proteome analysis revealed the absence of any phosphorylation in two proteins, while decreased phosphorylation of the carboxysome-associated protein CcmM and increased phosphorylation of the allophycocyanin alpha subunit ApcA was detected in Δ*spkB*. Furthermore, the regulatory P_II_ protein appeared less phosphorylated in the mutant compared to WT, which was verified in Western blot experiments, indicating a clearly delayed P_II_ phosphorylation in cells shifted from nitrate-containing to nitrate-free medium. Our results indicate that SpkB is an important regulator in *Synechocystis* that is involved in phosphorylation of the P_II_ protein and additional proteins.

## Highlights

• Protein phosphorylation *via* serine/threonine protein kinases (Spk) is a widespread mechanism.
• A collection of 11 completely segregated *spk* mutants was investigated in *Synechocystis* PCC 6803.
• Especially the mutant defective in SpkB encoded by *slr1697* showed clear deviations regarding carbon metabolism.
• A phospho-proteome analysis revealed the absence of any phosphorylation in two proteins.
• Furthermore, the regulatory P_II_ protein appeared less phosphorylated in the mutant compared to WT.

## Graphical Abstract

**Figure:**
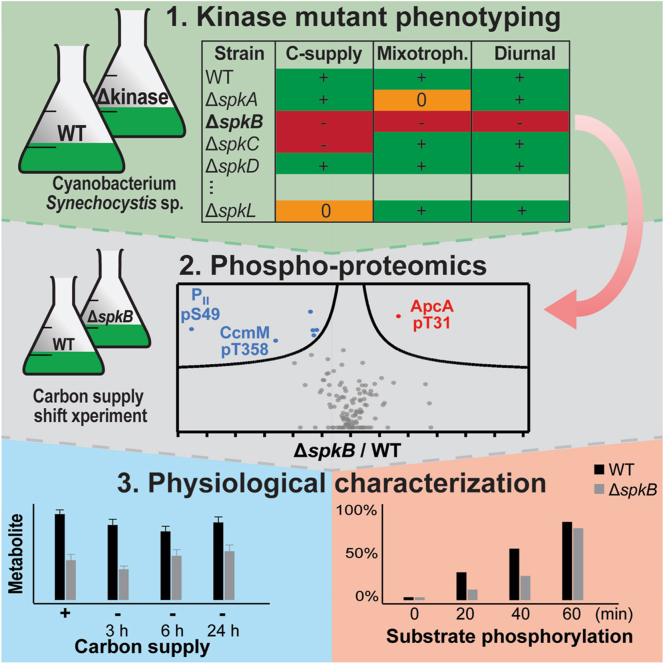

## In Brief

Mutation of the serine/threonine protein kinase SpkB resulted in many phenotypical alterations in the cyanobacterium *Synechocystis* sp. PCC 6803. A subsequent phospho-proteome analysis revealed the absence of any phosphorylation in two proteins, while decreased phosphorylation of the carboxysome-associated protein CcmM and the regulatory P_II_ protein was observed. Our results indicate that SpkB is an important regulator of the carbon/nitrogen metabolism in *Synechocystis*.

Cyanobacteria are the only prokaryotes performing oxygenic photosynthesis. It is well established that ancient cyanobacteria evolved oxygen-evolving photosynthesis approximately 3 billion years ago, which was later conveyed *via* endosymbiosis in a eukaryotic host cell initiating the evolution of phototrophic algae and plants (1). Due to this close evolutionary relationship, cyanobacteria are often used as models to study basic processes of photosynthesis and its regulation. Moreover, cyanobacteria received increasing attention as so-called green cell factories because they can be engineered as producers of bioenergy or feedstock using atmospheric CO_2_ and sun light (2, 3).

Cyanobacteria as all other phototrophic organisms must coordinate light capturing *via* photosynthetic complexes and carbon fixation *via* the Calvin-Benson cycle under fluctuating conditions, mainly the availability of light and CO_2_. Light for photosynthesis changes in diurnal light/dark cycles as well as during the day due to shading by clouds and in aquatic organisms by mixing in the water column. CO_2_ became increasingly limiting during the course of evolution because the activities of photosynthetic organisms decreased its atmospheric contents to present day low concentration of about 0.04% CO_2_ and also initiated the accumulation of high concentration of about 20% O_2_. The present day composition of Earth’s atmosphere severely impacts the activity of ribulose 1,5-phosphate carboxylase/oxygenase (RubisCO) that has a rather low CO_2_ affinity and can also react with O_2_ in the competing oxygenase reaction (4, 5). In aquatic systems, the availability of inorganic carbon (Ci - dissolved CO_2_ and bicarbonate) is fluctuating according to changes in pH and temperature values.

To acclimate to limiting and fluctuating Ci conditions, cyanobacteria and many algae evolved efficient CO_2_-concentrating mechanisms (CCM) (reviewed in (6, 7)). The activity of the cyanobacterial CCM is regulated on different layers, for example, many genes for Ci transporters crucial for the CCM function are upregulated under limiting Ci at transcriptional levels (*e.g.*, (8, 9)). The changes in the CCM activities are accompanied by different metabolic composition in cyanobacteria at fluctuating Ci conditions (10, 11). However, the expression of genes for enzymes involved in primary carbon metabolism is not significantly changed in the cyanobacterial model strain *Synechocystis* sp. PCC 6803 (9, 12), which gave rise to the hypothesis that posttranslational control plays an important role in regulating carbon partitioning according to Ci availability (13).

Posttranslational control of carbon partition can be achieved by direct regulation of key enzymes in carbon metabolism (*e.g.*, (14)), but the most wide-spread mechanism is related to posttranslational modifications of proteins (reviewed in (15)). Among them, reversible phosphorylation especially on serine or threonine residues by serine/threonine-specific protein kinases (Spks) represent a wide-spread measure to modulate activities of enzymes and regulatory proteins. In the cyanobacterial model strain *Synechocystis* sp. PCC 6803 (hereafter *Synechocystis*), 12 different Spks have been annotated in its genome (http://genome.microbedb.jp/cyanobase/GCA_000009725.1) (see supplemental Table S1 for gene IDs and nomenclature), which can be separated by sequence features into two large clusters: eukaryotic-like Pkn-type and ABC1-type Spks (16, 17). In contrast, more than 500 different phosphorylation events on serine/threonine residues on about 250 proteins of *Synechocystis* have been detected in the last years (12, 18, 19, 20), which raises the question about the specificity of Spks in *Synechocystis* or alternative mechanisms for serine/threonine phosphorylation.

A previous attempt to analyze a collection of *spk* mutants in *Synechocystis* revealed that SpkE is associated with the response to cold stress, while the eukaryotic-like kinases SpkC and SpkF are involved in phosphorylation of GroES (21). Furthermore, the *Synechocystis* kinases SpkA (22) and SpkB (23) have been shown to be important for cell motility, while SpkG has been associated to high salt tolerance (24). Studies with recombinant Spk proteins verified that SpkA, SpkB, SpkC, SpkD, and SpkF are biochemically active protein kinases because they were able to phosphorylate themselves and/or artificial substrates such as casein or histones using *in vitro* assays with radiolabeled ATP (22, 23, 25). Only a few relations between Spks and specific protein targets in *Synechocystis* have been solved to date. In addition to the mentioned involvement of SpkC and SpkF in GroES phosphorylation (21), SpkB has been shown to phosphorylate GlyS (glycyl-tRNA synthetase subunit beta) (26), while SpkG is involved in the phosphorylation of the ferredoxin Fd5 (27). Moreover, several potential phosphorylation substrates of SpkC were identified by phospho-proteomics, among them proteins involved in the CCM that were differentially phosphorylated under changing Ci conditions (12).

To study the role(s) of Spks in *Synechocystis*, we initially screened a collection of 11 completely segregated *spk* mutants under different conditions. Particularly, the mutant defective in SpkB, encoded by the gene *slr1697*, showed clear deviations regarding the carbon metabolism compared to WT. The proteome of Δ*spkB* revealed several distinct alterations, which indicate changes of the cell surface but also metabolic functions. Analysis of the phospho-proteome showed the absence of any phosphorylation in two proteins, while decreased phosphorylation of the carboxysome-associated protein CcmM and the regulatory P_II_ protein were observed in the mutant compared to the WT. Collectively, our results indicate that SpkB is an important regulator under different environmental conditions in *Synechocystis* and seems to interact in the P_II_ phosphorylation and probably with further substrates in a kinase network.

## Experimental Procedures

### Strains and Cultivation

All mutant strains analyzed in this study were established either in the *Synechocystis* sp. strain PCC 6803M (WT M) (28) or strain PCC 6803 (WT F) (29). The kinase-deficient mutants were generated by disrupting the reading frames of the kinase-encoding genes by insertion of a gene cartridge encoding for different antibiotic resistance proteins retrieved from the pUC4K (Amersham) and derivatives pUC4S and pUC4G (supplemental Fig. S1). The identity of the mutations was confirmed by genotyping of the mutant strains with mutation-specific primer pairs (supplemental Table S2). All strains and their origin are listed in the supplemental Table S3.

### Photoautotrophic Growth at Different CO_2_ Conditions

WT and mutant cells were precultivated in glass tubes filled with BG11 (TES pH 8.0; ref. (29)) aerated with 5% CO_2_ (high CO_2_, HC) at 30 °C and 120 μmol photons m^−2^ s^−1^ for 3 days. Eventually, *Synechocystis* suspension was adjusted to an optical density at 720 nm (OD720) of 0.2 with BG 11 TES pH 7.0 and grown under ambient air (low CO_2_ of about 0.04%, LC). The growth was monitored with the Multi-Cultivator MC-1000-OD system (Photon Systems Instruments), in which the OD720 increase was continuously recorded at 30 °C and 100 μmol photons m^−2^ s^−1^ for 4 days. Samples from HC- and LC-acclimated cells were used to record absorption spectra from 400 to 750 nm. Spectra data were concomitantly utilized for pigment quantification. Raw growth data were normalized to their start OD720 (OD720_N_ = OD720_n_/OD720_0_) and subsequently normalized to the WT from 0 to 1 (OD720_NMut_ = (OD720_Mut_ − OD720_NWTmin_)/(OD720_NWTmax_ − OD720_NWTmin_)).

### Mixotrophic Growth at Ambient Air

Cells of WT and mutants deficient in Spks were inoculated from material maintained on solid medium into Erlenmeyer flasks filled with 100 ml BG11. The strains were precultivated shaking (140 rpm) at ambient air (LC) with 35 μmol photons m^−2^ s^−1^ and 30 °C for 7 days. Ahead of the growth experiment, cultures were adjusted to OD750 of 0.5 with BG11 (TES pH 8.0) and subsequently split into two separate cultures with one supplemented with 10 mM glucose whereas the remaining served as a control. The strains were grown shaking at ambient air with 35 μmol photons m^−2^ s^−1^ and 30 °C. Growth was monitored by measuring manually the OD750 using a photometer every 24 h until the cultures reached the stationary growth phase. Raw growth data were normalized to their start OD750 and subsequently normalized to the WT from 0 to 1 (OD750_NMut_ = (OD750_Mut_ − OD750_WTmin_)/(OD750_WTmax_ − OD750_WTmin_)).

### Drop Dilution Assay

Kinase-deficient mutants together with their respective WT were precultivated in shaking flask with BG11 (TES pH 8.0) at 30 °C and 100 μmol photons m^−2^ s^−1^ in an LC environment until they reached the desired OD750. Upon drop dilution assay, the suspension was adjusted to the OD750 of 0.2 with BG11 (TES pH 8.0). Then, the suspension was serial diluted to 1:10, 1:100, or 1:1000. Two microliters of the dilution series were spotted on solid BG11 plates (TES pH 8.0; 1.5% bacto agar) containing different supplements. For mixotrophic conditions, 10 mM glucose and for a salinity environment 500 mM NaCl were added to the medium. Plates were either incubated at 30 °C and 100 μmol photons m^−2^ s^−1^ at continuous light (photo mixotrophic, high salinity) or at diurnal light conditions (12 h light/12 h darkness) at 30 °C and 75 μmol photons m^−2^ s^−1^ for 4 days. Pictures were taken together with their respective control plate.

### Tolerance Towards Externally Supplied H_2_O_2_

To test the sensitivity of the mutant strains towards externally supplied reactive oxygen species (ROS), cells were probed with increasing amount of H_2_O_2_. Strains were precultivated in shaking flasks in BG11 (TES pH 8.0) at 30 °C and light of 100 μmol photons m^−2^ s^−1^ in an LC environment until they reached the desired OD750. Cell suspensions were then adjusted to OD750 of 0.4 and probed with either 3 mM or 4 mM of H_2_O_2_ followed by an incubation under grow light conditions for 1 h. After H_2_O_2_ incubation, the suspension was serial diluted to 1:10, 1:100, or 1:1000. Two microliters of the dilution series were spotted on solid BG11 plates (TES pH 8.0; 1.5% bacto agar). Surviving cells were recovered for 4 days at 30 °C and 100 μmol photons m^−2^ s^−1^ at constant light. Pictures were taken together with their respective control plate.

### Glycogen Quantification

For glycogen quantification, *Synechocystis* was precultivated in a batch culture under HC (BG11 TES pH 8.0) conditions at 30 °C and 100 μmol photons m^−2^ s^−1^ continuous light until desired OD750 of 1 was reached. Upon shift to LC, cells were spun down (5 min; 4000 rpm) and resuspended in BG11 TES pH 7.0. *Synechocystis* was further cultivated under LC condition for 24 h. Samples for glycogen quantification were taken at an HC-acclimated state and 3 h and 24 h of LC acclimation. Cellular glycogen content was determined by applying the method described (30). Glycogen was quantified as glucose in the supernatant using the o-toluidine reagent and using a glucose standard curve.

### NO_3_^−^ Deprivation Experiment

Cells of the *Synechocystis* WT together with mutant Δ*spkB* were precultivated at HC (BG11 pH 8.0) until desired OD750 was reached. The night before the initial shift, cells were adjusted to OD750 of 1 in BG11 (TES 7.0) and transiently acclimated to LC conditions. Before the NO_3_ deprivation, cells were harvested (5 min; 5000 rpm; 4 °C) and immediately snap frozen in liquid nitrogen until further processing. Remaining cells were spun down (5 min; 5000 rpm; 4 °C) and resuspended in BG11 TES pH 7.0 without the addition NaNO_3_. Samples were taken 20, 40, 60, or 120 min after the initial nitrogen shift and snap frozen in liquid nitrogen.

### Protein Extraction and Native-PAGE

Harvested cells were resuspended in 300 μl protein extraction buffer (20 mM Tris–HCl pH 7.5, 100 mM NaCl) and subsequently broken by sonicating (six times 15 bursts at a duty cycle of 20 and an output of 75%; Bandelin Sonoplus HD70). The disrupted cell suspension was then spun down for 20 min at 3000 rpm and 4 °C. The crude extract was used for quantifying total protein content (ROTI Nanoquant). Crude extracts were probed with native sample buffer (187.5 mM Tris–HCl pH 6.8, 30% (v/v) glycerol, 0.0015% (v/v) bromophenol blue) in a 3:1 ratio. Ten micrograms of crude protein extract were loaded on a native-PAGE (15% separation gel; Rotiphorese 40) and separated for about 3 h at 170 V. Proteins were transferred to a polyvinylidene difluoride membrane (Thermo Fisher Scientific) using a semidry blot system (Thermo Fisher Scientific) applying a constant current of 6 V for 90 min. The immunoblot was probed with a P_II_-antibody (31) that was diluted 1:1000.

### HCO_3_^−^-Dependent Photosynthetic O_2_-Evolution Rates

HCO_3_^−^-dependent photosynthetic O_2_-evolution rates were quantified using an S1 Oxygraph (Hansatech Instruments). Suspensions of HC- or LC-acclimated cells were adjusted to Chl*a* 10 μg ml^−1^ with CO_2_-free BG11. Three milliliters of the adjusted suspension were used to quantify O_2_ evolution rates at 30 °C and at a saturating light intensity of 300 μmol photons m^−2^ s^−1^ in the presence of increasing HCO_3_^−^ concentrations (0, 33.3, 66.7, 133.3, 266.7, 400, 533.3, 666.7, 1000, 1333.3, 2000, or 2666.7 μM). Photosynthetic rates at each HCO_3_^−^ concentration were recorded at 30 s intervals.

### Metabolite Analysis

For metabolite analysis, cells were precultivated in a batch culture under either LC (BG11 TES pH 7.0) or HC (BG11 TES pH 8.0) conditions at 30 °C and continuous light of 100 μmol photons m^−2^ s^−1^ until desired OD750 of 1 was reached. Upon shift, cells were spun down (5 min, 4000 rpm) and resuspended in either BG11 TES pH 7.0 or BG11 TES 8.0. Cells were further cultivated under either LC or HC condition for 24 h. A 5 ml sample was taken at a long-term HC- and LC-acclimated state and after 1, 3, 6, or 24 h of acclimating to LC and HC environment, respectively. Cells were harvested by quick filtration on nitrocellulose filters (25 mm, Porafil, Macherey-Nagel). Metabolites were quantified as described (30).

### Cell Cultivation for Phospho-Proteome Analyses

Cells of the WT and mutant Δs*pkB* were precultivated in glass tube batch cultures under HC conditions (5% CO_2_) in buffered BG11 medium (TES pH 8.0) at 30 °C and 120 μmol photons m^−2^ s^−1^ until the cell suspension reached OD750 of ∼1. The cultures were transferred daily into fresh BG11 medium to avoid nutrient limitation. Two days before the shift experiment, the precultures were adjusted to OD750 of 1.0 and split into three individual cultures that were kept under HC conditions. After growth for 24 h, the cell suspensions were again readjusted to OD750 of 1.0. Cells were harvested by centrifugation and resuspended in fresh BG11 medium to inoculate six individual cultures of 120 ml with an OD750 of 1.0. To allow recovery and acclimation to the new conditions, cells were aerated with 5% CO_2_ in the light for 2 h before HC samples were taken. For harvesting, 40 ml cell suspension were separated from each individual culture and split into two equal portions and transferred into 50 ml tubes filled with ice for rapid inhibition. The cells were immediately pelleted by centrifugation (6000*g*, 7 min, 4 °C). Cell pellets were washed with 7 ml ice-cold PBS-buffer, snap-frozen in liquid nitrogen, and stored at −80 °C until further processing. For the shift to LC conditions, the medium of the remaining culture was removed by centrifugation and the cells were resuspended in the same volume of BG11 (TES pH 7.0) and further cultivated with ambient air (0.04% CO_2_) aeration. Samples from LC-shifted cultures were harvested after 3 h and 24 h. Three independent cultivations were conducted for replicated phospho-proteome analyses.

### LC-MS/MS-Based Phospho-Proteome Analyses

Proteins were extracted from cell pellets by resuspension and heating in detergent buffer (4% w/v sodium dodecyl sulfate in 100 mM Tris–HCl, pH 8) supplemented with phosphatase inhibitors (glycerol-2-phosphate, sodium fluoride, and sodium orthovanadate, 5 mM each) to 95 °C for 10 min, followed by sonification for 30 s using a micro-tip. Sample extracts were then reduced in presence of 10 mM DTT and alkylated in the presence of 5.5 mM iodoacetamide in the dark, each for 1 h at room temperature. Samples were centrifuged (13,000*g*, 15 min, 20 °C) and the supernatant was subjected to protein precipitation with acetone/methanol (8 + 1 sample volume) at −20 °C overnight. Protein pellets were washed using ice cold 80% v/v acetone until the supernatant was colorless and air dried, followed by resuspension in 2 ml urea buffer (6 M urea, 2 M thiourea in 100 mM Tris–HCl, pH 7.5). Protein concentrations were measured by Bradford assay and 3 mg protein per sample were digested to peptides at room temperature using proteomics grade endoproteinases Lys-C (30 μg; 3 h predigestion) and trypsin (30 μg) diluted in four sample volumes of 1 mM Tris–HCl, pH 8. After overnight incubation, peptide solutions were acidified with TFA to pH 2.5. One third (1 mg) of the material was separated from each sample and added to a standard mixture, containing equal fractions from all conditions and replicates. Subsequently, samples and standard mixture were loaded on C18 columns (Sep-Pak 1 cc, Waters) and dimethyl labeled as described previously (32). Labeled peptides were mixed in a 1:1:1 ratio with 6 mg total yield, always containing WT (light label) and Δ*spkB* (intermediate label) from the same condition as well as the standard mixture (heavy label) for quantitative comparison between different conditions, as outlined in the experimental design (supplemental Fig. S2). For proteome analyses, an aliquot corresponding to 0.1 mg was separated from each triplex mixture. Peptides were subsequently separated using high-pH reversed-phase peptide fractionation (Pierce Product # 84868, Thermo Fisher Scientific), resulting in nine individually measured fractions. The remaining material was subjected to phosphopeptide enrichment by TiO_2_ chromatography using Sachtopore-NP 5 μm titania spheres (ZirChrom Separations) in a 10:1 peptide:TiO_2_ ratio in 6% v/v TFA/80% v/v acetonitrile solution for 10 min. For each mix, eight consecutive enrichment rounds were performed, of which rounds 1 + 2, 3 to 5, and 6 to 8 were pooled, since our previous studies using this method (*e.g.*, (12)) revealed that phosphopeptides are gradually depleted during consecutive enrichment rounds. All samples from proteome fractionation and phosphopeptide enrichment were subjected to StageTip purification before mass spectrometry (MS) analysis (33).

For MS measurements, 250 ng material from proteome fractions and all available material from pooled phosphopeptide enrichment fractions were loaded onto a C18 nanoHPLC column (20 cm, 75 μM ID, NewObjective), in-house packed with ReproSil-Pur 1.9 μm C18 material (Dr Maisch), and separated by RP-chromatography on an EASY-nLC 1200 system using 60 min segmented linear gradients at a constant flow rate of 200 nl min^−1^. Eluting peptides were ionized via an on-line coupled ESI source and analyzed on an Orbitrap Exploris 480 mass spectrometer (Thermo Fisher Scientific) in the positive-ion mode. During data-dependent acquisition, MS spectra were acquired with an m/z range of 300 to 1750 at resolution 60,000. The 20 most intense multiple charged ions were selected for fragmentation by higher-energy collisional dissociation and fragment ions were recorded at MS2 resolution 30,000. Automatic gain control targets and maximum injection times were set for MS and MS2 scans to instrument setting “standard” and “auto”, respectively. Dynamic exclusion of previously sequenced precursor ions was set to 30 s in all measurements.

### MS-Data Processing

Acquired MS raw data was processed with MaxQuant software suite (version 1.6.0.8; https://www.maxquant.org/) using the following search parameters: Trypsin was defined as a cleaving enzyme and up to two missed cleavages were allowed. Dimethylation on peptide N-termini and lysine residues was defined as light (+28.03 Da), intermediate (+32.06 Da), and heavy (+36.08 Da) labels. Carbamidomethylation of cysteines was set as fixed modification, and methionine oxidation, protein N-terminal acetylation and phosphorylation of serine, threonine and tyrosine residues were set as variable modifications. “Requantification,” “match between runs,” and “iBAQ” were enabled. The initial mass tolerance of precursor ions was limited to 20 ppm and 0.5 ppm for fragment ions. False discovery rates of (modified)peptides and proteins were limited to 1% each. Protein groups identified based on single peptides were only accepted with comprehensive fragment ion series (Supplemental Spectra of Single Peptide Identifications). A minimum of two ratio counts was required for quantification and normalized ratios, adjusted for mixing errors, were utilized for statistical analysis. Phosphopeptides surpassing an Andromeda score threshold of 40 were manually inspected with stringent acceptance criteria including Posterior Error Probability, detection in previous studies, and comprehensive fragment ion series (Supplemental Spectra of Phosphopeptides). Biological reliability was verified by intensity-based correlation of quantified proteins and phosphopeptides (see Results).

### Experimental Design and Statistical Rationale

MS-based phospho-proteome analyses were performed for each experimental condition (HC, 3 h and 24 h LC) separately in three independent biological replicates. In each replicate, triplex dimethyl labeled peptides of the WT (light labeled, L), strain Δ*spkB* (intermediate labeled, M), and the standard mixture (heavy labeled, H) were present. Nine proteome fractions and three pooled phosphopeptide fractions per replicate resulted in 36 samples (27 proteome and nine phospho-proteome measurements) per condition. The total of 108 samples from all experimental conditions was processed in MaxQuant with an experimental design defining condition and replicate of each sample (supplemental Fig. S2). Statistical analysis of significantly changing proteins and phosphorylation events was performed with Perseus software suite (version 1.6.5.0; https://maxquant.net/perseus/). For the direct comparison of Δ*spkB* to WT at each condition (including all replicates), normalized M/L label ratios were analyzed. For the strain-specific comparison of the proteome between different conditions, normalized ratios relative to the H-labeled standard mixture were calculated as 1/[(H/X LC condition)/(H/X HC condition)], with X = L for WT and X = M for Δ*spkB*, respectively. Student’s *t* test was used to determine proteins and p-events with significantly changed abundances compared to the overall distribution between independent replicates. A *p*-value ≤0.05 was defined as cutoff in all analyses.

## Results

### Mutant Collection Screening Under Different Growth Condition

The coding sequences of all annotated *spk* genes were interrupted by an antibiotic gene cartridge using appropriate restriction sites as done in a previous study to analyze the role of Spks in *Synechocystis* (21). Subsequent PCR analyses revealed that in 11 cases, the corresponding WT fragment was completely absent in the mutant genome, that is, only the mutation-specific fragment remained visible verifying that the mutant is completely segregated (supplemental Fig. S1). Only one exception was observed. Despite several attempts under different segregation conditions, the gene *sll0005* encoding SpkH could never be completely removed from the mutant genome. These results indicated that SpkH or other protein(s) encoded in the *sll0005*-containing operon is essential for viability of *Synechocystis* under our laboratory conditions, whereas all other Spks are dispensable.

**Fig. 1:**
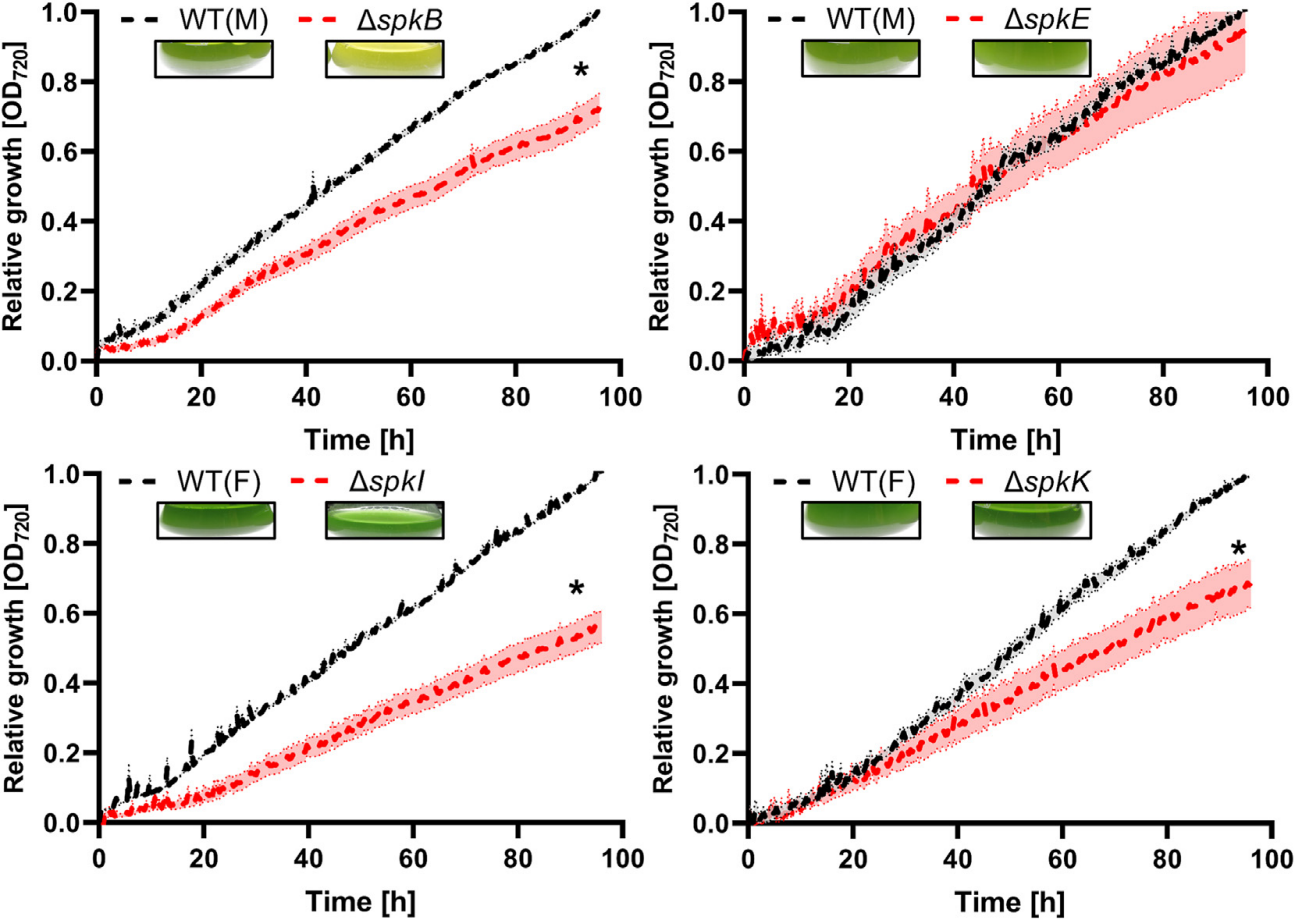
**Growth of selected *spk* mutants including** Δ***spkB* in response to low carbon conditions.** Cells of the *Synechocystis* WT and mutant strains Δ*spkB*, Δ*spkE*, Δ*spkI*, and Δ*spkK* were pre-acclimated to high CO_2_ (5%, HC) and transferred at time point 0 h to ambient air CO_2_ (0.04%, LC) conditions. Strains were grown at continuous light of 100 μmol photons m^−2^ s^−1^ and 30 °C. Growth is shown as relative increase of optical density at 720 nm (∗*p* > 0.05; n = 12). The insets show the optical appearance of cyanobacterial suspensions acclimated to LC.

The collection of 11 completely segregated *spk* mutants was then screened under different growth conditions. Initially, the ability of all mutants to acclimate to LC conditions was analyzed. To this end, mutant strains were precultured under HC conditions and then transferred to LC conditions for 4 days in continuous light. In addition to the previously characterized mutant Δ*spkC* (12), the mutants Δ*spkB*, Δ*spkI*, and Δ*spkK* showed significantly slower growth than the corresponding WT (Fig. 1 and supplemental Fig. S3). Especially, mutant Δ*spkB* is also less pigmented compared to WT under LC conditions. This bleaching phenotype is based on lower amounts of phycobilipigments and chlorophyll *a* leading to the yellowish appearance of this culture (Fig. 1; absorption spectra and pigment contents are shown in supplemental Fig. S4 and supplemental Table S4). The slower growth of strain Δ*spkB* could be complemented by ectopic expression of *spkB* on the plasmid pVZ322 (supplemental Fig. S5). Growth of the mutant Δ*spkB* was also tested under long-term LC conditions, in which the mutant grew again at a lower rate than the WT (data not shown).

Subsequently, the mutant collection was screened for differences in the ability to grow under diurnal conditions (12 h light/12 h dark). Only mutant Δ*spkE* showed slower growth than WT under diurnal conditions, whereas all other strains grew similar as WT (supplemental Fig. S6). Next, the growth of all strains was compared under high salt conditions of 500 mM NaCl. Among the *spk* mutants, a reduced salt tolerance was observed for Δ*spkI* (supplemental Fig. S6). Another important stress for photoautotrophic cyanobacteria represents oxidative stress. To test this condition, cell suspensions of all strains were exposed to externally supplied 3 or 4 mM H_2_O_2_ in the light for 1 h, which was found in preliminary experiments to be critical for the survival of our *Synechocystis* WT cells. Then, cells were harvested by centrifugation, resuspended in fresh BG11 medium, and spotted in dilutions onto plates to screen for surviving cells. The drop dilution assay showed that most strains have WT-like H_2_O_2_ resistance, while the mutants Δ*spkB* and Δ*spkG* seem to be more resistant against exposure to 4 mM H_2_O_2_, indicated by their better survival than WT (supplemental Fig. S7).

**Fig. 2:**
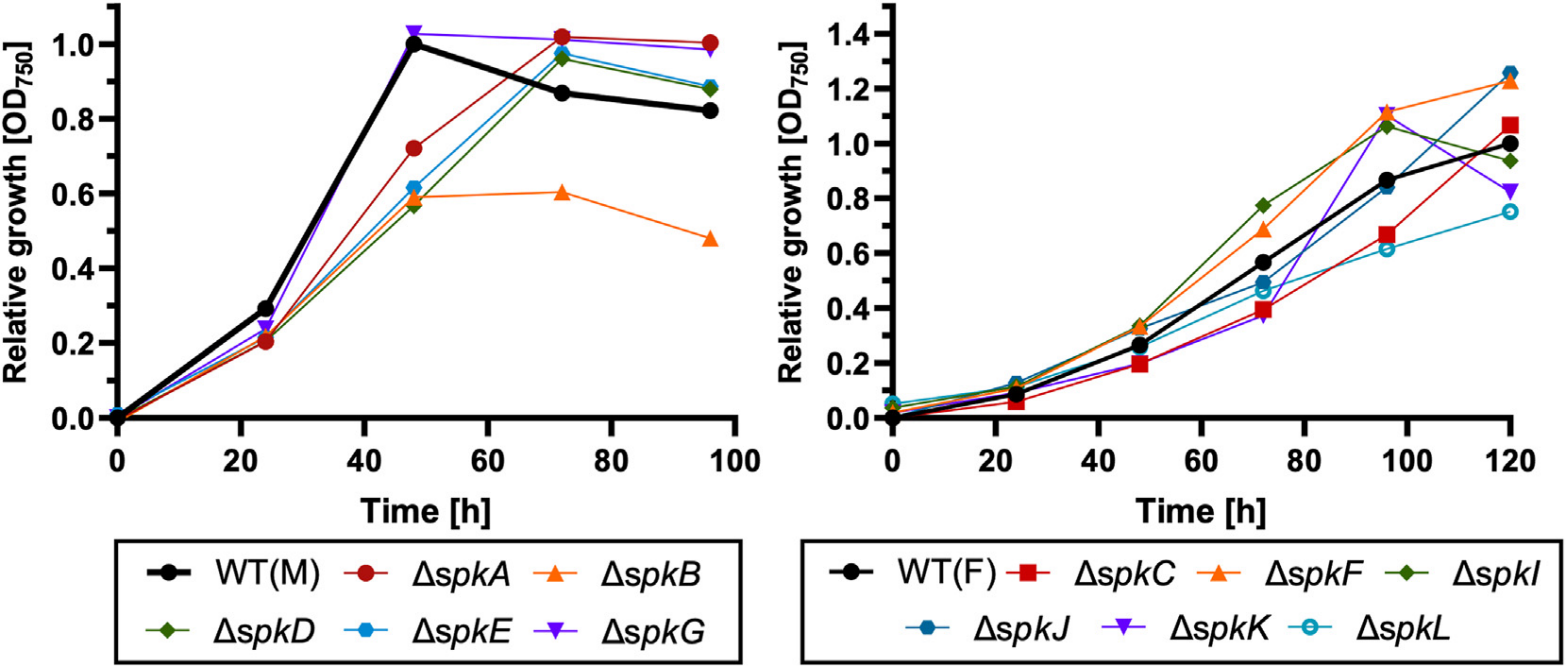
**Growth of kinase-deficient mutants Δ*spkA-L* under mixotrophic conditions.** Kinase-deficient mutants and their respective WT were precultivated in shaking flasks with BG11 (TES pH 8.0) at continuous light of 50 μmol photons m^−2^ s^−1^ and ambient air until the desired OD750 was reached. The cell suspensions were then inoculated in fresh BG11 (TES pH 8.0) supplemented with 10 mM glucose at an OD750 of 0.5. The growth was monitored for up to 4 days by measuring OD750 every 24 h. Retrieved data were normalized to WT levels (n = 3).

Next, we tested mixotrophic growth in flasks under LC conditions of ambient air because *Synechocystis* is not only able to grow photoautotrophic but can also use external glucose (29). Among all strains, only mutant Δ*spkB* showed clearly diminished growth in the presence of glucose (Fig. 2). The glucose-sensitive phenotype of this mutant was not only observed under continuous light but also diurnal conditions (supplemental Fig. S6). Summarizing, among all mutants, most deviations were found for strains defective in SpkB or SpkI (Table 1).

**Table 1:** Comprehensive overview on growth phenotypes of mutants Δ*spkA-L*

ΔKinase	HC-LC	Mixotroph	Diurnal	Diurnal+	Salt	H_2_O_2_/tolerance
Δ*spkA*	+++	++	+++	+++	++	+++
Δ*spkB*	+	+	+++	+	+++	++
Δ*spkC*	+	+++	+++	+++	+++	+++
Δ*spkD*	+++	+++	+++	+++	++	+++
Δ*spkE*	+++	+++	+	+	+++	+++
Δ*spkF*	+++	++	+++	+++	+++	+++
Δ*spkG*	++	++	+++	+++	+++	+
Δ*spkI*	+	++	++	++	+	+++
Δ*spkJ*	++	+++	+++	+++	+++	+++
Δ*spkK*	+	++	+++	+++	+++	++
Δ*spkL*	++	++	+++	+++	+++	++

The response of different mutant strains was compared in liquid or on solid media with the corresponding WT. +++, WT-like growth; ++, slight difference; +, strong difference.

**Fig. 3:**
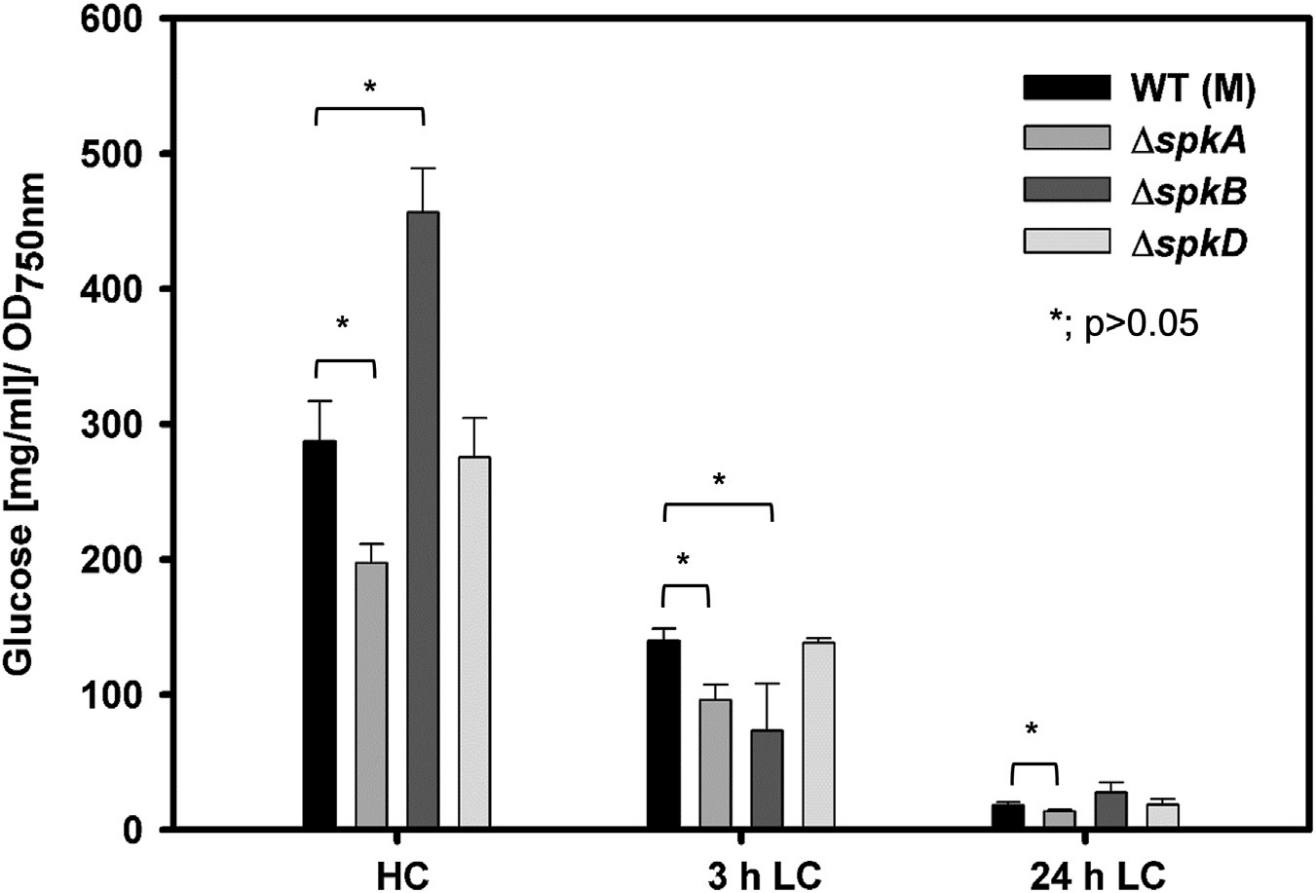
**Glycogen accumulation in selected *spk* mutants including Δ*spkB*.** Glycogen was quantified as glucose liberated by α-glucosidase from extracts of *Synechocystis* WT and mutant cells, which were precultivated under high CO_2_ conditions (HC, 5% CO_2_) and then shifted for 3 or 24 h into low CO_2_ (LC, 0.04% CO_2_) of ambient air.

Among the investigated strains, the mutant Δ*spkB* seems to be especially affected under different carbon conditions, since it grew slower under LC and also under glucose supplementation. Therefore, we analyzed some metabolic features of this strain. It has been shown that excess organic carbon is stored as glycogen in cyanobacterial cells under HC conditions, while glycogen is hardly detectable under LC conditions (34). The glycogen content of mutant Δ*spkB* was higher than WT cells when grown at HC; however, it was faster consumed after LC shift (Fig. 3). Hence, the slower growth of Δ*spkB* under LC conditions is not directly connected with a delayed glycogen consumption.

Targeted metabolome analysis permitted the quantification of important intermediates related to C- and N-metabolism in mutant and WT cells (supplemental Table S5). Regarding the steady state contents of 3-phosphoglycerate and 2-phosphoglycolate, that is, the products of carboxylation or oxygenation reaction of RubisCO, we could not see significant differences between WT and mutant Δ*spkB* when grown at HC and LC and when shifted to LC and HC conditions, respectively (supplemental Fig. S8). However, differences were found in the contents of glutamine and arginine (supplemental Fig. S9). The content of these amino acids is lowered in Δ*spkB* when grown at LC conditions but became similar to WT levels when mutant cells were shifted back into HC conditions. The opposite was observed when cells were pregrown at HC. The glutamine and arginine contents were initially similar to WT but became diminished in Δ*spkB* after LC shift (supplemental Fig. S9). In contrast, the amounts of glutamate, which represents by far the most abundant soluble amino acid, did not show significant differences between WT and mutant Δ*spkB*.

Collectively, the growth and metabolic characterizations of the mutant Δ*spkB* indicated that SpkB seems to be involved in the carbon/nitrogen homeostasis in *Synechocystis*.

### Proteome Analysis of Mutant ΔspkB

**Fig. 4:**
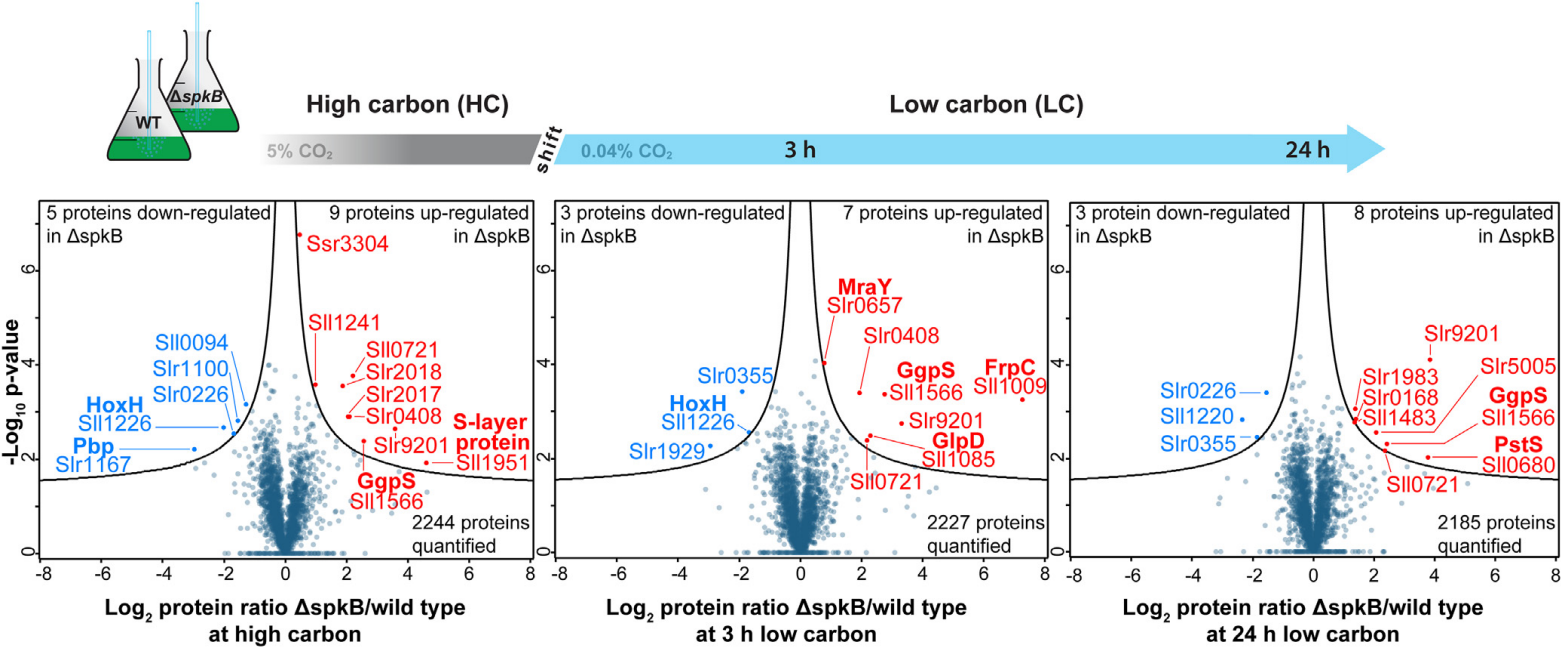
**Quantitative comparison of proteome changes between mutant Δ*spkB* and WT grown under different inorganic carbon conditions.** Cells of the *Synechocystis* WT and mutant Δ*spkB* were acclimated to high carbon conditions (HC, 5% CO_2_) and then shifted to low CO_2_ of ambient air (LC, 0.04% CO_2_) for 3 h or 24 h. Corresponding volcano plots indicate changes in protein abundance between mutant Δ*spkB* and WT at each condition (*left*: HC; *middle*: 3 h LC; *right*: 24 h LC). Proteins with significantly different abundances between both strains were analyzed in a *t* test (*p*-value = 0.05; S0 = 2) and are displayed in color (*blue* = decreased abundance in Δ*spkB*; *red* = increased abundance in Δ*spkB*).

To obtain a direct insight how this protein kinase might impact the carbon/nitrogen homeostasis in *Synechocystis*, we performed a phospho-proteome analysis. To this end, cells of the *Synechocystis* WT and mutant Δ*spkB* were precultivated under HC conditions and then shifted for 3 and 24 h into LC, identical to our previous study (12). The proteome analysis identified 2460 proteins, equivalent to approximately 67% of the annotated *Synechocystis* proteome. Among them, approximately 2200 proteins were quantified in all biological replicates at the three different sampling points (supplemental Fig. S10). Detected peptides of SpkB were either exclusively identified in WT or more abundant compared to Δ*spkB*, confirming the inactivation of this kinase in the mutant strain (supplemental Table S6). The response of the proteome to LC conditions in WT cells corresponds to the results of our previous study (12), indicating a good reproducibility of the experiment (supplemental Fig. S10). Only a relatively low number of proteins showed significant elevated or decreased levels (*p*-value 0.05) in cells of the mutant Δ*spkB* compared to WT (Fig. 4; data available in supplemental Table S7). Most changes between mutant and WT were consistently observed during all sampling points, that is, differences between the two strains are mostly independent from Ci supply. Generally, the Ci-responsive proteins such as subunits of the bicarbonate uptake systems or specific NDH1 complexes were induced to almost the same extent in LC-shifted mutant as in WT cells (supplemental Figs. S11 and S12; data available in supplemental Table S8).

In addition to several hypothetical or unknown proteins, the upregulated proteins belong to four functional groups in mutant Δ*spkB*. First, proteins of the cell envelope such as the surface layer protein Sll1951 and FrpC (Sll1009), the latter also comprises an SLH domain and might be functionally connected to the S-layer (35), showed the highest increases. Second, several type 4 pilin-like proteins (encoded in the operon *slr2015-17*) that are essential for cell motility are found at higher abundances in mutant Δ*spkB*, whereas the amount of the type 4 pilin-like protein Slr1929 is strongly diminished (supplemental Table S7). Reduced protein abundances were likewise observed for the type 4 pilin-like protein Slr1928 and Slr1931. These proteome changes are consistent with the observed nonmotile phenotype of the mutant Δ*spkB* (23). Third, two proteins involved in the synthesis of the compatible solute glucosylglycerol (36), that is, GgpS (Sll1566) and GlpD (Sll1085) are also present at elevated levels in mutant extracts, which might indicate that the changed cell surface strength influences the cellular turgor pressure and thereby mimicking a salt or osmotic stress situation. Interestingly, an elevated level of GgpS was recently reported as common feature in four mutants with deleted regulatory proteins of the carbon metabolism in *Synechocystis* (37), which might indicate that GgpS accumulation due to mutation of *spkB* could be also associated with a dysregulated carbon metabolism. Fourth, the two PstS proteins of *Synechocystis* (Sll0680 and Slr1247) and the alkaline phosphatase Sll0654 accumulated in Δ*spkB*, which point at a phosphate limitation in the mutant because these three proteins belong to the phosphate regulon in *Synechocystis* (38). In addition to the type 4 pilin-like protein Slr1929, the histidine kinase 37 (Hik37, Sll0094) and subunits of hydrogenase (*e.g.*, HoxH, Sll1226) were found among the proteins with lowered abundance in mutant Δ*spkB* (supplemental Table S7). Collectively, the proteome data show that the absence of the SpkB has rather low impact on the cellular protein composition and does not provide clear hints regarding the observed changes in its growth at different carbon conditions. Since SpkB is a serine/threonine-specific protein kinase, but its specific target proteins have not been identified so far, we further analyzed the phospho-proteome of WT and Δ*spkB*.

### Phospho-Proteome Analysis of Mutant Δ*spkB*

To analyze the SpkB-specific changes in protein phosphorylation, we enriched phosphorylated peptides from the total protein extracts of the WT and Δ*spkB* by TiO_2_-affinity chromatography and subjected enriched fractions to LC-MS/MS–based identification. In total, 227 phosphorylated serine, threonine, and tyrosine residues (p-events) were reliably identified on 115 different proteins (supplemental Table S9). About 70% of these p-events have been identified before including many proteins bearing multiple p-events, particularly subunits of the phycobilisome or the protein CcmM. Interestingly, we observed that some phosphopeptides can be exclusively detected in a multiple phosphorylated state (*e.g.*, phosphorylation of RpoD at T148 and S155), whereas other multiply phosphorylated peptides reveal one constant and several occasional p-events (*e.g.*, phosphorylation of Sll0103 at T380 and either T384 or T387) (supplemental Table S9). Due to the identification of many low abundant p-events present in single replicates, the quantification rate of p-events resides between 40 and 50% at different Ci conditions. Overall, more p-events were detected after the shift towards LC conditions than in nonstressed HC conditions, indicating a potential role of protein phosphorylation during stress acclimation (supplemental Fig. S13).

The data set was first screened for p-events that were exclusively identified in WT cells and absent in Δ*spkB* (supplemental Table S9). To this end, phosphorylation of the proteins Sll1545 (T266) and Slr0483 (T34 or T35) was only detected during several time points in at least on replicate in WT samples. Slr1545 is the glutathione S-transferase, Gst1, which plays an important role in the redox regulation of proteins among cyanobacteria (39). The Slr0483 protein is a membrane protein of unknown function that bears a CAAD domain (cyanobacterial aminoacyl-tRNA synthetase appended domain, PMID: 18775859). Interestingly, SpkB has been shown to phosphorylate GlyS (glycyl-tRNA synthetase subunit beta) that also bears a CAAD domain (26).

**Fig. 5:**
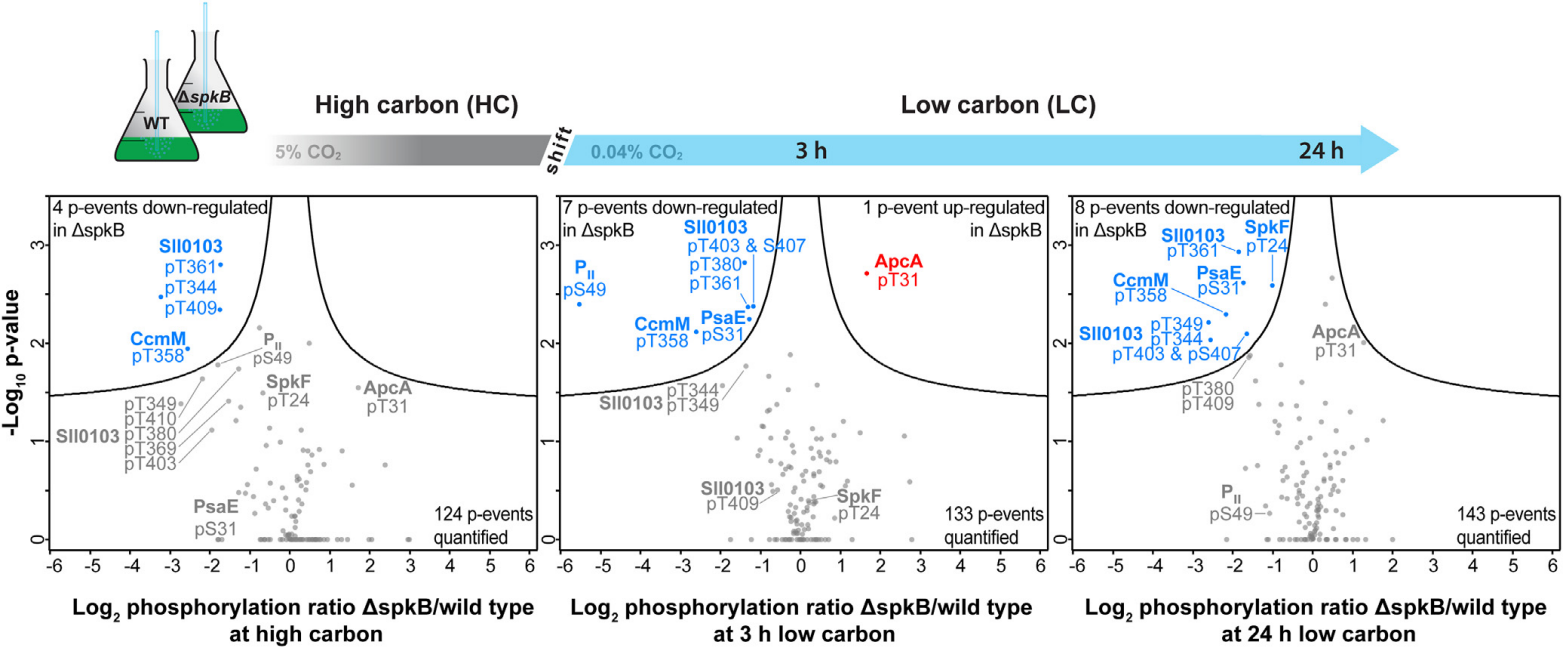
**Quantitative comparison of phospho-proteome changes between mutant Δ*spkB* and WT grown under different inorganic carbon conditions.** Cells of the *Synechocystis* WT and mutant Δ*spkB* were acclimated to high carbon conditions (HC, 5% CO_2_) and then shifted to low CO_2_ of ambient air (LC, 0.04% CO_2_) for 3 h or 24 h. Corresponding volcano plots indicate changes in phosphorylation abundance between mutant Δ*spkB* and WT at each condition (*left*: HC; *middle*: 3 h LC; *right*: 24 h LC). P-events with significantly different abundances between both strains were analyzed in a *t* test (*p*-value = 0.05; S0 = 2) and are displayed in color (*blue* = decreased abundance in Δ*spkB*; *red* = increased abundance in Δ*spkB*).

In addition to these proteins without any detected phosphorylation in Δ*spkB*, a few p-events were identified with significantly diminished phosphorylation in this mutant. Since the proteomes of WT and Δ*spkB* revealed overall similar protein abundances at all Ci conditions and none of the significantly regulated proteins (Fig. 4) are phosphorylated, we directly compared phosphopeptide ratios between both strains without prior normalization to protein levels. Overall 11 p-events were detected on the Sll0103 protein, which is a predicted membrane–embedded Ca^2+^-regulated chloride channel (40). In addition to its approx. 50% reduced protein abundance in Δ*spkB*, six p-events were disproportionately less phosphorylated at all conditions in the mutant (Fig. 5). Multiple p-sites were also detected for the carboxysomal CcmM protein, which was equally abundant in WT and Δ*spkB*. Four of its five detected p-events revealed uniformly reduced phosphorylation regardless of the Ci regime, of which T358 was classified to be significant. In addition, phosphorylation of S31 on the photosystem I (PSI) subunit PsaE was significantly lower at LC conditions in Δ*spkB*, while the corresponding protein levels were only slightly reduced. PsaE is situated at the donor site of PSI and plays an important role in the ferredoxin docking and cyclic electron transport (*e.g.*, (41)). Interestingly, the (auto)phosphorylation of SpkF at T24 was significantly diminished at 24 h LC in the mutant Δ*spkB* accompanied with generally reduced protein levels in this strain. Furthermore, the phosphorylation of the P_II_ (GlnB) protein at S49 was strongly reduced in Δ*spkB* compared to the WT when grown at HC and especially when shifted for 3 h to LC (Fig. 5). The P_II_ protein is the master regulator of many aspects in the carbon/nitrogen homeostasis in cyanobacteria and other organisms as well (reviewed in (42)). Contrary to this, one phosphoprotein revealed also significantly enhanced phosphorylation in Δ*spkB*, which is the allophycocyanin A (ApcA, Slr2067) at T31. Two further p-sites showed similar trends at HC and partially at LC (Fig. 5 and supplemental Table S9). ApcA is one of the terminal emitters of light energy from the phycobilisome towards especially the photosystem II (PSII) in cyanobacteria (43).

### Impact of Changes in the Phospho-Proteome on the Physiology of Mutant ΔspkB

The phospho-proteome indicated specific changes in the phosphorylation of different proteins in the mutant missing the serine/threonine kinase SpkB. The CcmM protein is involved in carboxysome biogenesis and its structural organization thereby playing an important role in the cyanobacterial CCM (*e.g.*, (44)). Subsequently, we compared the Ci-dependent photosynthesis in cells of the mutant Δ*spkB* and the WT after acclimation to HC and LC conditions, respectively. These experiments did not reveal any significant differences between the two strains regarding V_max_ and K_m_ values of photosynthesis (supplemental Fig. S14).

**Fig. 6:**
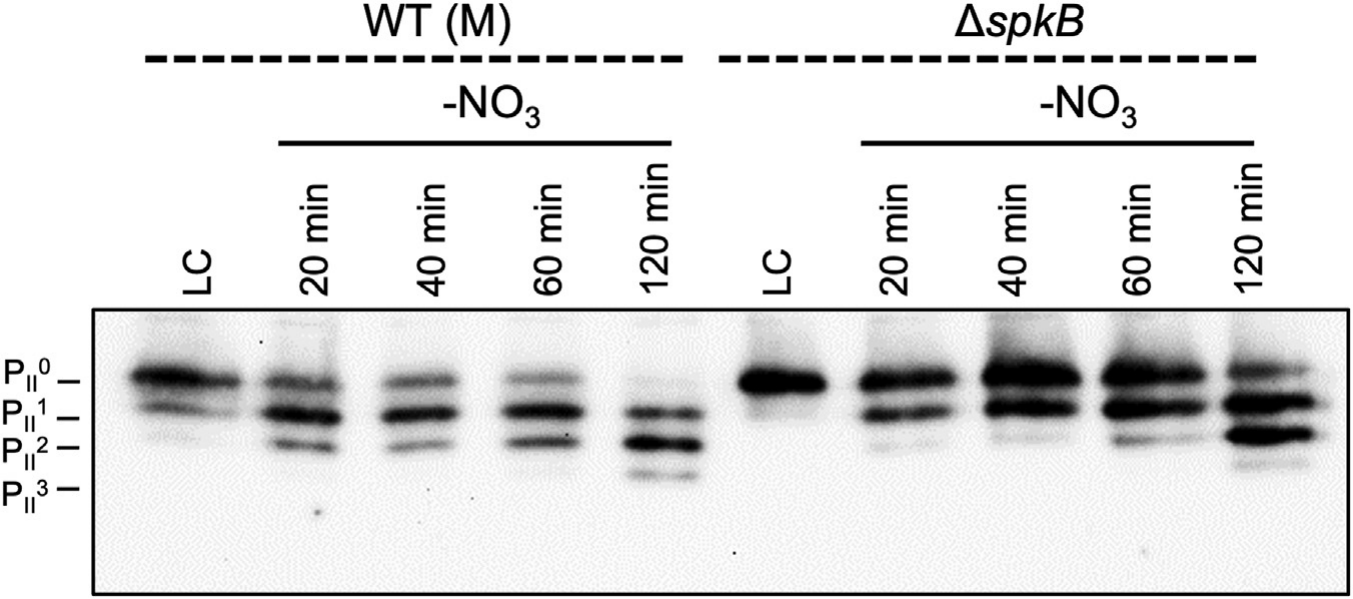
**Visualization of the P**_**II**_**phosphorylation state in *Synechocystis* WT(M) and mutant Δ*spkB*.** Strains were cultivated under LC (ambient air; BG11 pH 7.0) in nitrate (NO_3_) containing medium. Prior to the shift, LC-acclimated cells were harvested (5 min; 5000 rpm; 4 °C) from WT and kinase-deficient mutant and snap frozen in liquid nitrogen until further processing. Upon shift, *Synechocystis* was spun down (5 min; 5000 rpm; RT) and washed in BG11 without the addition of NO_3_. Cells were resuspended in BG11 without NO_3_. Samples were taken 20, 40, 60, and 120 min after the initial nitrogen shift (5 min; 5000 rpm; 4 °C, snap frozen in liquid nitrogen). *Synechocystis* cells were lysed by sonication. Ten micrograms of crude cell extract were loaded on a native-PAGE (15% separation gel) and after immunoblotting probed with an P_II_-antibody (1:1000). Indicated are the phosphorylation states of the subunits of the P_II_-trimer.

Since we detected significantly different P_II_ phosphorylation states between WT and Δ*spkB* by LC-MS/MS, we aimed to verify this observation by Western-blotting after protein separation under native conditions. This technique permitted the distinct visualization of the differentially phosphorylated P_II_ trimers, which can be separated in four bands (31). The upper band represents the nonphosphorylated form and the lower running bands correspond to trimeric P_II_ protein with 1, 2, or 3 phosphorylated subunits (Fig. 6). It has been shown that cultivation at LC in nitrate-containing BG11 medium results in least phosphorylated P_II_, whereas the transfer into N-free BG11 medium induces rapid P_II_ phosphorylation in *Synechocystis* cells (*e.g.*, (45)). Thus, LC-grown cells were shifted into nitrate-free BG11 medium to verify differences in the P_II_ phosphorylation state in Δ*spkB*. At all time points, the nonphosphorylated upper P_II_ band revealed higher intensities than bands of phosphorylated P_II_ trimers in the blot with protein extracts from Δ*spkB*. It is also obvious that the phosphorylation of P_II_ was slower and less complete after shift into N-free BG11 medium in Δ*spkB* compared to WT (Fig. 6). These results verify that the absence of SpkB results in less intensive P_II_ phosphorylation as indicated in the phospho-proteome analysis; however, the P_II_ phosphorylation is not completely abolished pointing at the action of alternative or substituting Spks on P_II_ in *Synechocystis*.

## Discussion

Bacteria as other organisms need to acclimate to changing conditions in their natural environments, which often involves transcriptional control using a network of many transcription factors and multiple sigma factors. Such acclimation strategies are also well documented in model cyanobacteria. For example, genome-wide expression changes under diverse environmental conditions have been analyzed in *Synechocystis* and are summarized in the database CyanoEXpress (http://cyanoexpress.sysbiolab.eu/). In several cases, a good correlation between the transcriptome and proteome has been reported for *Synechocystis*, for example, after high salt or low CO_2_ stress (12, 36). However, despite many changes in the LC-induced proteome, most enzymes involved in the central carbon and nitrogen metabolism remained at unchanged abundances, whereas the metabolome was clearly different between HC and LC conditions (*e.g.*, (10, 34)). These findings made it likely that posttranscriptional control of enzyme activities is an important mechanism for the optimization of carbon partitioning under different CO_2_ supply (13). One often discussed possibility to fine tune metabolic processes is differential protein phosphorylation *via* protein kinases in bacteria (15). Therefore, a detailed analysis of serine/protein kinases in *Synechocystis* could shed light on the question to which extend this regulatory layer is important for environmental stress acclimation in cyanobacteria.

Our results revealed that the absence of specific protein kinases is often correlated with significant alterations in their capability to grow at different environmental conditions. However, the absence of some Spks, for example SpkA, D, F, J, and L, have had only low or no impact on the performance of *Synechocystis* under our test conditions, whereas other *spk* mutations resulted in clear phenotypic alterations (see Table 1). Only one protein kinase, SpkH encoded by *sll0005*, was found to be essential for viability. Recently, it has been shown that recombinant SpkH is an active protein kinase of the ABC1 class that could phosphorylate casein *in vitro* (46).

Our and previous results indicate that protein phosphorylation *via* serine/threonine kinases is indeed important for the stress acclimation; however, it is not possible to decide whether these defects are indirect or which specific protein target might be responsible for the changed phenotype. Among the 11 investigated *spk* mutants, the strain missing SpkB showed the highest number of growth changes under the different stresses. Moreover, it was particularly affected under conditions with changed carbon availability, which is related with our attempts to understand changed carbon partitioning under different Ci conditions. Therefore, we decided to investigate the mutant Δ*spkB* in more detail.

Previous *in silico* investigation of cyanobacterial Spks showed that they bear considerable sequence similarity to eukaryotic Spk families (16), thereby, SpkB belongs to the large group of PKN-type kinases and is grouped in the CAMK subfamily (17). The recombinant SpkB protein was investigated and proven to be an active Spk because it showed ATP-dependent autophosphorylation and phosphorylation of typical artificial kinase substrates such as casein (23). A few studies already analyzed phenotypical alterations of the mutant Δ*spkB*. Initially, the loss of motility was reported for mutant Δ*spkB* (23). This phenotype is well supported by our proteome study, which showed significant alterations in the abundance of cell surface and pilin proteins (see Fig. 4), which are certainly involved in the motility of *Synechocystis*. A later study revealed that the SpkB kinase activity can be modulated by redox changes, where the Cys-rich N-terminal extension was shown to be the target of thioredoxin-mediated redox changes (26). Correspondingly, we found that the glutathione S-transferase, Gst1, which plays an important role in the redox regulation of proteins among cyanobacteria (39), is not phosphorylated in the mutant Δ*spkB* anymore. These findings implied that SpkB might be important for the acclimation towards different redox conditions. Accordingly, growth of mutant Δ*spkB* was affected under conditions promoting redox imbalance in *Synechocystis* such as high light or iron starvation (26). Changes of Ci availability, which were analyzed here, certainly also impact the cellular redox homeostasis. It can be assumed that cells grown under HC conditions are less reduced than cells shifted into LC. Limited Ci supply increases the acceptor limitation of the photosystems because less reducing equivalents and ATP are needed in the Calvin-Benson cycle (see discussion in ref. (47)). Such changes in the redox state of the cells are also obvious in the transcriptome and proteome of *Synechocystis* cells shifted from HC into LC because stress proteins associated with high light or redox stress (*e.g.*, *hliP*’s) are more abundant in cells with limited Ci availability (*e.g.*, (48)). However, in contrast to the study of Mata-Cabana *et al*. (26), who reported an increased sensitivity of Δ*spkB* towards the addition of methyl-viologen and thus a decreased ROS resistance, we found an improved ROS resistance after supplementation of the medium with H_2_O_2_ (supplemental Fig. S7). This difference could be explained by the different ROS stress scenarios. Addition of methyl-viologen impacts the activity of PSI, which then generates ROS inside the cell. Externally applied H_2_O_2_ needs to diffuse inside the cell, before it could harm cellular processes. It is possible that the changed cell wall properties of mutant Δ*spkB* found in our proteome analysis somehow decrease the H_2_O_2_ inward diffusion making the cell more resistant against this externally applied ROS.

To obtain a more direct hint about the specific role of SpkB, the phospho-proteome of mutant Δ*spkB* and WT was compared under different Ci conditions. Among the many detected p-events, the phosphorylation of the proteins Sll1545 (T266) and Slr0483 (T34 or T35) were exclusively found at different Ci conditions in WT samples but never in Δ*spkB*, which highlights them as potential substrates of SpkB. Slr1545 is the glutathione S-transferase, Gst1, which plays important role in the redox regulation of proteins among cyanobacteria (39); hence its change in phosphorylation is consistent with the observed changes in ROS tolerance of the *Synechocystis* Δ*spkB* mutant. The Slr0483 protein is a membrane protein of unknown function that bears a CAAD domain (cyanobacterial aminoacyl-tRNA synthetase appended domain, PMID: 18775859). Previously, the GlyS (glycyl-tRNA synthetase subunit beta), a protein from the same functional group as Slr0483 that, however, was found to be nonphosphorylated in the present study, has been reported as SpkB substrate (26). There, GlyS was less phosphorylated during *in vivo* [γ-^32^P] ATP-labeling experiments under different redox conditions in mutant Δ*spkB* compared to WT. The ability of SpkB to use GlyS as substrate was verified by *in vitro* kinase assays with recombinant SpkB protein (26). In our present and previous *in vivo* studies (12, 19), GlyS was only detected on proteome levels but never in a phosphorylated state (see supplemental Tables S7 and S9). Since the localization of GlyS p-sites could previously not be identified (26), it remains unclear whether GlyS phosphorylation potentially evades identification by common LC-MS/MS–based phospho-proteome analysis, for example, due to ineligible peptide properties, or whether it is *in vivo* essential in *Synechocystis*.

In addition to these proteins without any detected phosphorylation in Δ*spkB*, other p-events were identified with significantly diminished phosphorylation in Δ*spkB*. The changed phosphorylation of CcmM came into our focus because this protein is important for the CCM in cyanobacteria (reviewed in (7)) and could thus be related to the reduced growth after shifts into LC conditions. However, we did not find a changed Ci affinity of whole cell photosynthesis between Δ*spkB* and WT. A closer inspection of the p-events in the *Synechocystis* CcmM revealed that the four phosphorylation sites are not at all conserved in the CcmM proteins of *Synechococcus elongatus* PCC 7942 or of *Anabaena* sp. PCC 7120. This notion makes it unlikely that differential CcmM phosphorylation might be a key factor in the condensate formation with RubisCO during carboxysome genesis in cyanobacteria (44). Phycobiliproteins are other examples bearing multiple p-events. Among them, phosphorylation of allophycocyanin A (ApcA) at T31 was significantly increased while the other identified seven p-events on this protein and those in other phycobilisome subunits were less strongly increased or remained unchanged (Fig. 5 and supplemental Table S9). ApcA is one of the terminal emitters of light energy from the phycobilisome especially on the PSII in cyanobacteria (43). Changed phycobilisome phosphorylation has been previously implicated in the possible regulation of photosynthetic light absorption among cyanobacteria (49). However, since ApcA phosphorylation at T31 became increased in a mutant with missing protein kinase SpkB, this alteration is most likely an indirect effect.

Finally, significant less phosphorylation at S49 of the P_II_ protein was detected in the phospho-proteome analysis and supported by Western blots detecting the different P_II_ phosphorylated trimers (Figs. 5 and 6). Despite the long-known phosphorylation of the P_II_ protein in cyanobacteria (31), the involved protein kinase remained elusive, while the phosphatase specific for dephosphorylation of S49 in P_II_ has been identified as PphA (50). Hence, SpkB appears to be a promising candidate for a P_II_-specific Spk. It has been shown that cells grown under LC conditions in N-rich BG11 medium show the least P_II_ phosphorylation, which is then triggered in response to N-limitation (31, 45). These conditions were selected to compare the time course of P_II_ phosphorylation in the mutant Δ*spkB* and WT. Corresponding to the putative role of SpkB as P_II_-specific kinase, the P_II_ phosphorylation level was always lower than in WT. Especially after shift into N-free BG11 medium, a substantial amount of P_II_ remained nonphosphorylated (see Fig. 6). Nevertheless, a fraction of P_II_ became still phosphorylated, which points to the presence of a substituting kinase that can phosphorylate P_II_ with lower efficiency. It should be noted that similar observations were made in a mutant of *S. elongatus* PCC 7942, in which the *spkB* ortholog *synpcc7942_1294* was inactivated (51). However, we cannot rule out that SpkB is involved in the inactivation of PphA because an elevated dephosphorylation of P_II_ in the mutant Δ*spkB* could also explain the observed changes in the P_II_ phosphorylation level. Clearly, more experiments are needed in the future, especially *in vitro* phosphorylation assays of pure SpkB and P_II_ protein with and without PphA, to reveal whether or not SpkB is indeed a specific P_II_ kinase or if the observed phosphorylation changes are indirectly related to the mutation of *spkB*.

A strongly increased P_II_ phosphorylation was recently observed in a mutant defective in the histidine kinase Hik8 (37), which has been shown to play an important regulatory role for the carbon metabolism in *Synechocystis* (52, 53). Interestingly, hyper-phosphorylation of P_II_ in the *hik8* mutant was accompanied by a significantly reduced glycogen accumulation, which was reversed to WT levels by expression a P_II_ variant with mutated S49 phosphorylation site (37). These findings are consistent with our observation that the mutant Δ*spkB* with decreased P_II_ phosphorylation at S49 contains more glycogen under HC conditions. However, P_II_ is not directly acting on glycogen synthesis or carbon metabolism. Recently, the P_II_-interacting protein PirC has been identified as key regulator to switch carbon flow between lower glycolysis and glycogen accumulation because PirC can inhibit phosphoglycerate mutase under N-limiting conditions when the accumulation of the metabolic regulator 2-oxoglutarate releases it from its P_II_-bound state (14). Our proteome data revealed that the amount of PirC is lower (log_2_ FC −0.6, below our significance threshold, supplemental Table S7) in mutant Δ*spkB* than WT under HC conditions, while the PirC and the glycogen amounts are similar 24 h after LC shift. Hence, in addition to the changed P_II_ phosphorylation, the changes of PirC amounts in mutant Δ*spkB* could also contribute to the observed alterations in glycogen contents.

Collectively, our results strongly support the notion that protein phosphorylation *via* Spks play an important role in stress acclimation of cyanobacteria. Among the annotated Spks, SpkB seems to be of particular importance because its absence directly or indirectly results in many phenotypic alterations and changes in the phospho-proteome. However, it seems to act in a kinase network as it is exemplified in the P_II_ phosphorylation. The action of multiple kinases on specific target proteins in *Synechocystis* has also been shown in the phosphorylation of GroES (21). This corresponds to the general observation that bacterial Spks have a rather relaxed substrate specificity (54). Furthermore, yet not-identified kinase types might exist among bacteria or nonenzymatic phosphorylation, for example, *via* acetyl phosphate (55), is much more widespread than presently assumed, which could close the gap between the relatively low number of annotated Spks and the much higher number of p-events detected in *Synechocystis* proteins (20). Finally, the spectrum of posttranslational modifications regulating metabolic enzymes involved in carbon metabolism goes beyond protein phosphorylation. For example, carbamylation of lysines has recently been identified as a wide-spread modification in *Synechocystis*, which can regulate protein activities such as RubisCO but also the P_II_ protein in a Ci-dependent manner (56). Summarizing, to fully understand metabolic regulations such as carbon partitioning in cyanobacteria, the complete spectrum of protein modifications and their impacts on enzyme activities needs to be more considered in the future.

## Data Availability

The original contributions presented in the study are included in the article and the associated Supplementary Material; further inquiries can be directed to the corresponding author. The mass spectrometry proteomics data have been deposited at the ProteomeXchange Consortium *via* the PRIDE (57) partner repository with the dataset identifier PXD040383 (Access to the protected data for reviewers: Username: reviewer_pxd040383@ebi.ac.uk; Password: afmsTbpk).

## Supplemental data

This article contains supplemental data.

## Conflict of interest

The authors declare no competing interests.

## Supplemental Data

Supplemental Tables S1–S9

Supplemental spectra of phosphopeptides

Supplemental spectra of single peptide identifications
