# Supplementary material for: The Role of Serine/Threonine-Specific Protein Kinases in Cyanobacteria - SpkB Is Involved in Acclimation to Fluctuating Conditions in Synechocystis sp. PCC 6803
Source: Mol Cell Proteomics. 2023 Oct 4;22(11):100656. doi: 10.1016/j.mcpro.2023.100656 (PMC10651672; doi:10.1016/j.mcpro.2023.100656)
Supplement: Supplemental Figures S1–S14 [file mmc1.pdf]

Supplemental data for the manuscript:

The role of serine/threonine-specific protein kinases in cyanobacteria - SpkB is involved in acclimation to fluctuating conditions in *Synechocystis* sp. PCC 6803

Thomas Barske[1]*, Philipp Spät[2,3]*, Hendrik Schubert[4], Peter Walke[1], Boris Maček[3], Martin Hagemann[1,5]**

* - the first two authors contributed equally to the study

1 – Institute of Biosciences, Department of Plant Physiology, University of Rostock, Rostock, Germany

2 - Interfaculty Institute of Microbiology and Infection Medicine Tübingen, Department of Organismic Interactions, University of Tübingen, Tübingen, Germany

3 - Interfaculty Institute for Cell Biology, Department of Quantitative Proteomics, University of Tübingen, Tübingen, Germany

4 - Institute of Biosciences, Department of Aquatic Ecology, University of Rostock, Rostock, Germany

5 – Interdisciplinary Faculty, Department Life, Light and Matter, University of Rostock, Rostock, Germany

Corresponding author: Martin Hagemann, Institut für Biowissenschaften, Abteilung Pflanzenphysiologie, Universität Rostock, A.-Einstein-Str. 3, Rostock D-18059, Germany; Tel: +49(0)3814986110; Fax: +49(0)3814986112; Email: martin.hagemann@uni-rostock.de

Supplemental Figure S1: Cloning strategies and verification of Δ*spkA-L.*

Supplemental Figure S2: Experimental design for triplex dimethylation labeling.

Supplemental Figure S3: Growth of Δ*spk* mutants in response to low Ci conditions.

Supplemental Figure S4: Pigmentation of Δ*spk* mutants grown at different Ci conditions.

Supplemental Figure S5: Growth of a strain ectopically expressing *spkB* at low Ci conditions.

Supplemental Figure S6: Sensitivity of kinase-deficient mutants towards different conditions.

Supplemental Figure S7: Sensitivity of kinase-deficient mutants towards external H_2O_2

Supplemental Figure S8: Values of 3PGA and 2PG in Δ*spkB* under different Ci conditions.

Supplemental Figure S9: Values of Gln, Glu, and Arg in Δ*spkB* under different Ci conditions.

Supplemental Figure S10: Venn diagrams and reproducibility of the proteome analysis.

Supplemental Figure S11: Proteome changes under low Ci conditions in the WT.

Supplemental Figure S12: Proteome changes under low Ci conditions in the Δ*spkB*.

Supplemental Figure S13: Venn diagrams and reproducibility of phospho-proteome analyses.

Supplemental Figure S14: Inorganic-carbon-dependent photosynthetic activity.

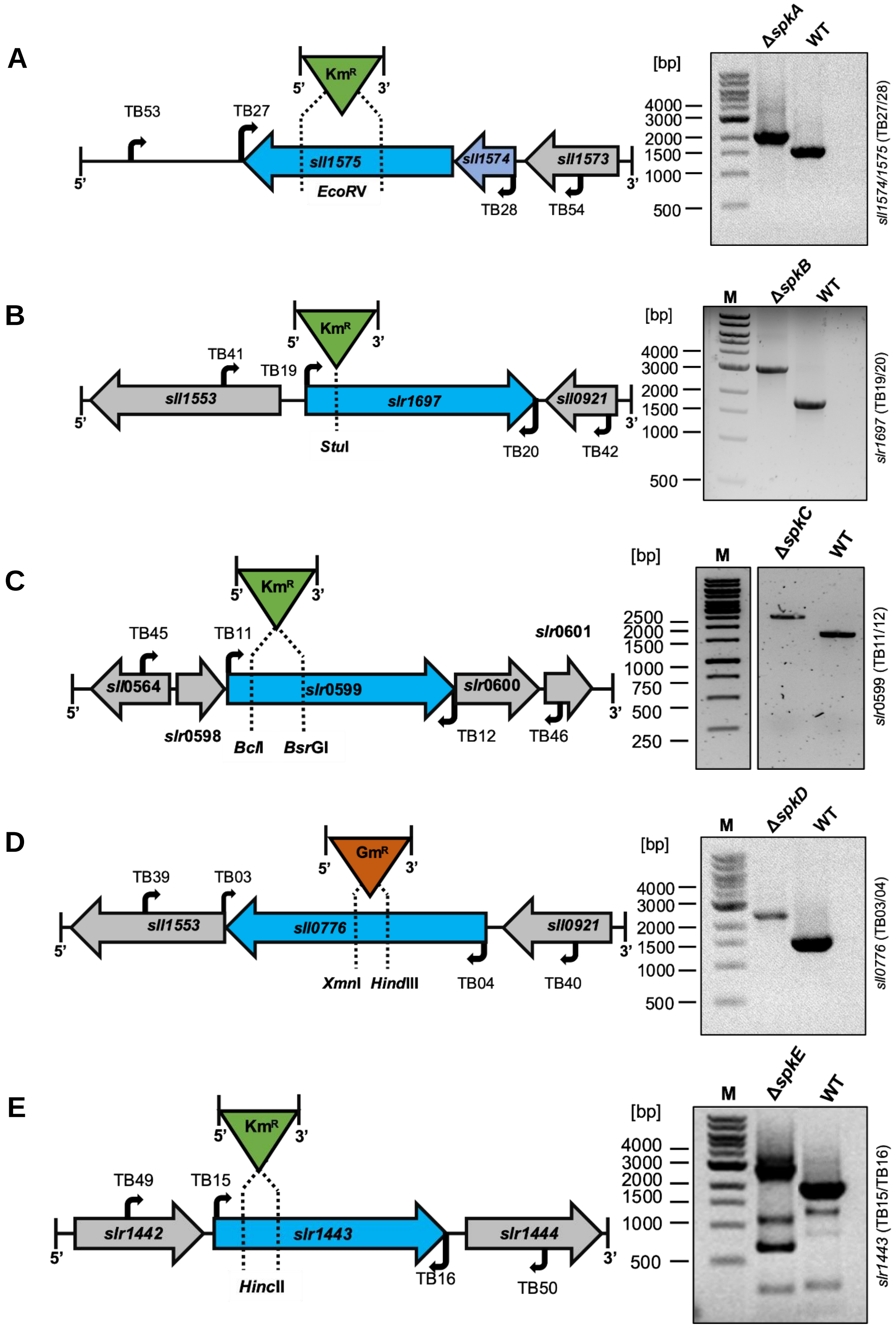
A
KmR
5'
3'
TB53
TB27
sll1575
sll1574
sll1573
EcoRV
TB28
TB54
ΔspkA
WT
[bp]
4000
3000
2000
1500
1000
500
sll1574/1575 (TB27/28)
B
KmR
TB41
TB19
sll1553
slr1697
sll0921
StuI
TB20
TB42
M
ΔspkB
WT
[bp]
4000
3000
2000
1500
1000
500
slr1697 (TB19/20)
C
KmR
TB45
TB11
slr0601
sll0564
slr0599
slr0600
slr0598
BclI
BsrGI
TB12
TB46
M
ΔspkC
WT
[bp]
2500
2000
1500
1000
750
500
250
slr0599 (TB11/12)
D
GmR
TB39
TB03
sll1553
sll0776
sll0921
XmnI
HindIII
TB04
TB40
M
ΔspkD
WT
[bp]
4000
3000
2000
1500
1000
500
sll0776 (TB03/04)
E
KmR
TB49
TB15
slr1442
slr1443
slr1444
HincII
TB16
TB50
M
ΔspkE
WT
[bp]
4000
3000
2000
1500
1000
500
slr1443 (TB15/TB16)

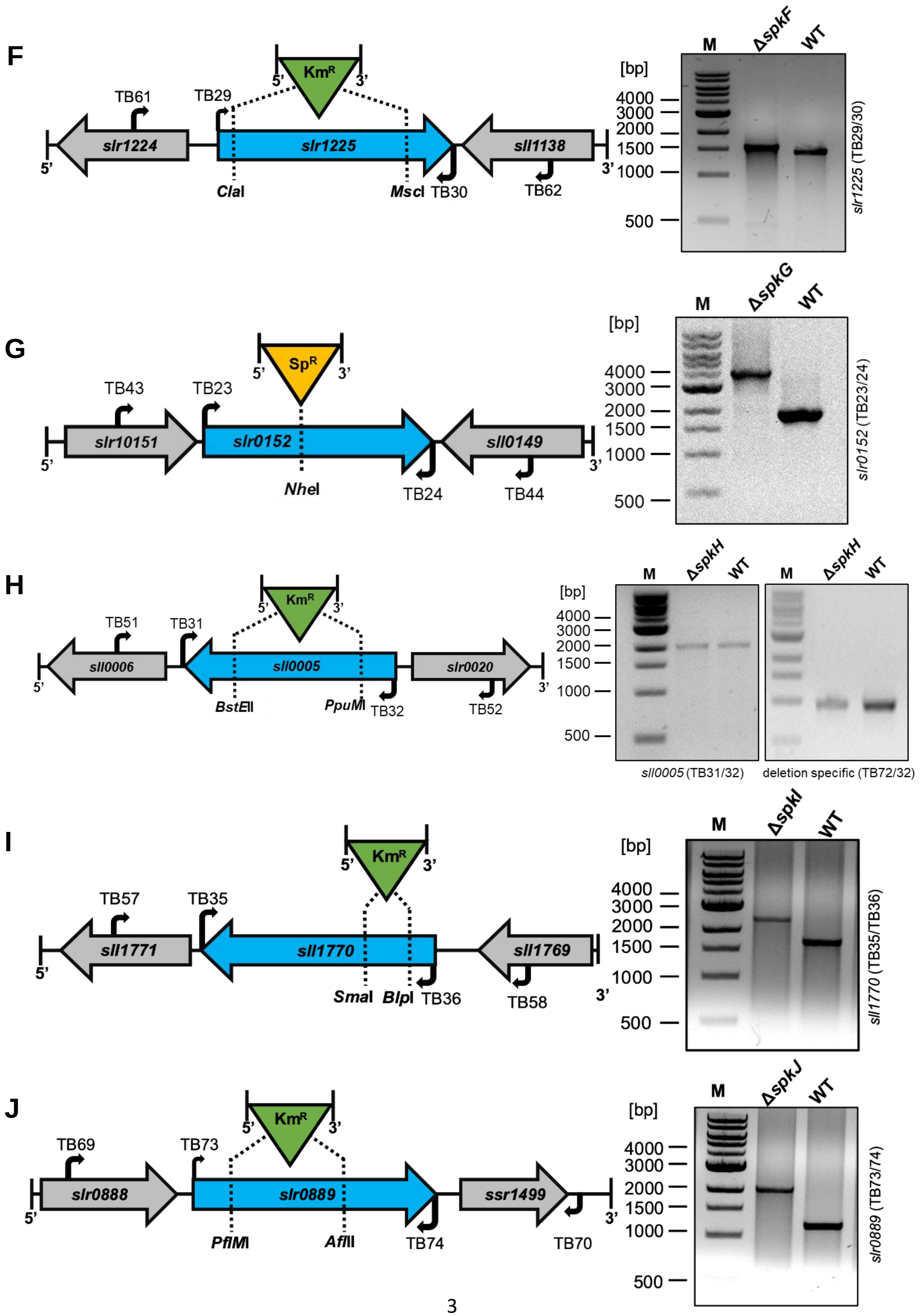
F
KmR
5’
3’
TB61
TB29
slr1224
slr1225
sll1138
5’
3’
ClaI
MscI
TB30
TB62
M
ΔspkF
WT
[bp]
4000
3000
2000
1500
1000
500
slr1225 (TB29/30)
G
SpR
5’
3’
TB43
TB23
slr10151
slr0152
sll0149
5’
3’
NheI
TB24
TB44
M
ΔspkG
WT
[bp]
4000
3000
2000
1500
1000
500
slr0152 (TB23/24)
H
KmR
5’
3’
TB51
TB31
sll0006
sll0005
slr0020
5’
3’
BstEII
PpuMI
TB32
TB52
M
ΔspkH
WT
M
ΔspkH
WT
[bp]
4000
3000
2000
1500
1000
500
sll0005 (TB31/32)
deletion specific (TB72/32)
I
KmR
5’
3’
TB57
TB35
sll1771
sll1770
sll1769
5’
3’
SmaI
BlpI
TB36
TB58
M
ΔspkI
WT
[bp]
4000
3000
2000
1500
1000
500
sll1770 (TB35/TB36)
J
KmR
5’
3’
TB69
TB73
slr0888
slr0889
ssr1499
5’
3’
PflMI
AflII
TB74
TB70
M
ΔspkJ
WT
[bp]
4000
3000
2000
1500
1000
500
slr0889 (TB73/74)

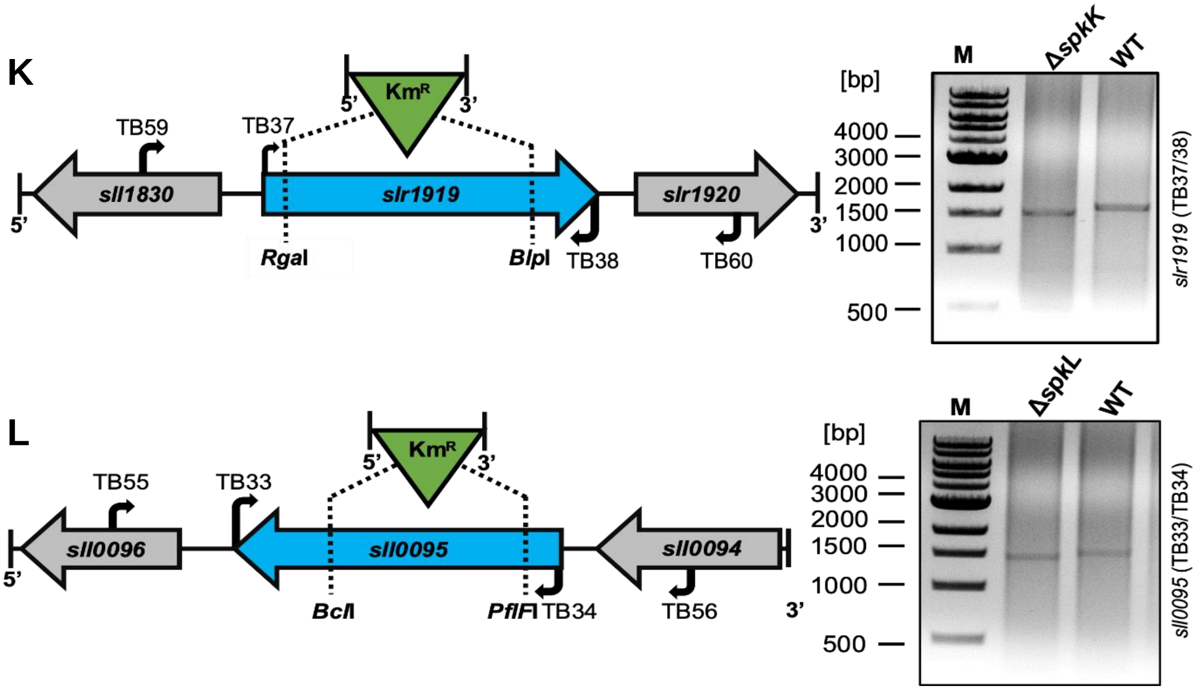

Suppl. Figure S1: Cloning strategies and verification of Δ*spkA-L*. The kinase encoding genes were amplified together with 500 bp up- and downstream sequences from chromosomal DNA of *Synechocystis* were subcloned in either pJet1.2/blunt (CloneJet, Thermofisher Scientific, USA) or pGEMT (Promega, USA) for sequencing. The reading frames were disrupted by the insertion of an antibiotic resistance cartridge. The constructs were used to transform *Synechocystis* (**A-L**).

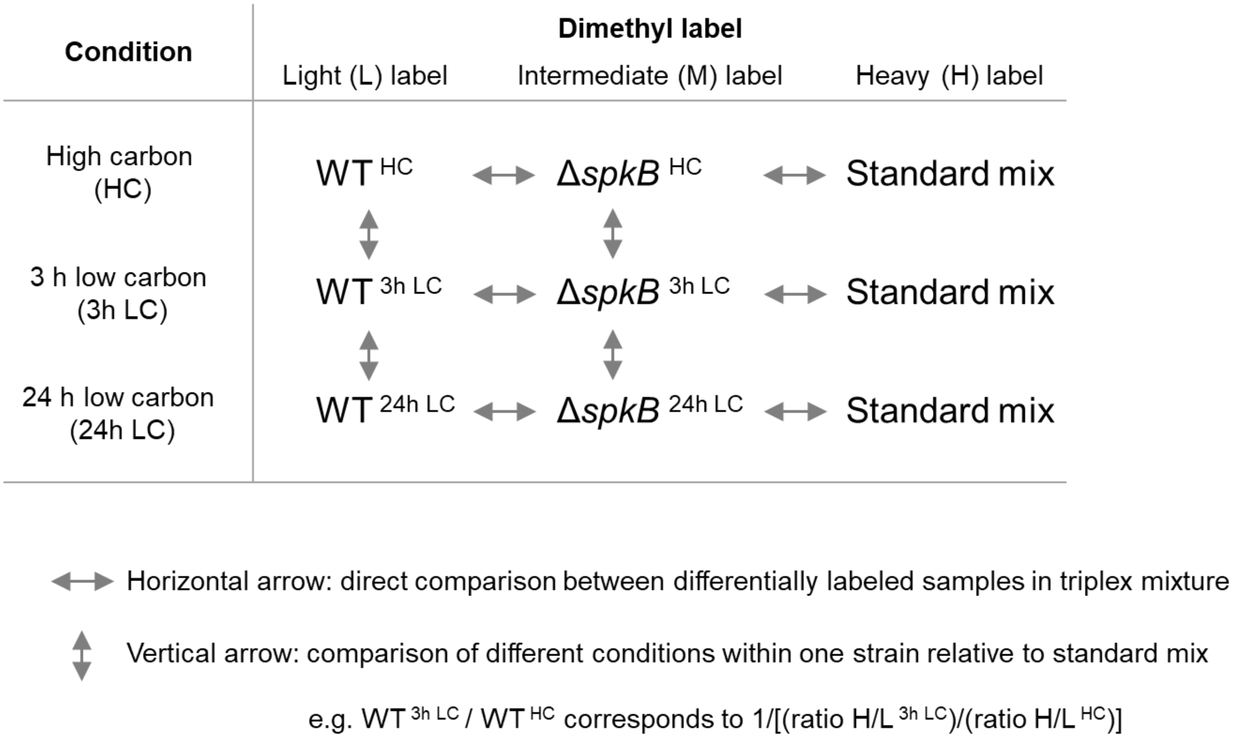

Suppl. Figure S2: Experimental design for triplex dimethylation labeling. Indicated is the chemical peptide dimethyl labeling strategy, which was applied for the direct comparison between the phospho-proteomes of the wild type (WT) and mutant Δ*spkB* within each condition. For each triplex labeling mix, Lys-C/trypsin digested protein extracts from the WT and Δ*spkB* cultured at identical conditions were light and intermediate heavy label labeled, respectively. In addition, a heavy labeled standard mix, consisting of all samples, was added for the comparison between different conditions for the WT and Δ*spkB*, respectively.

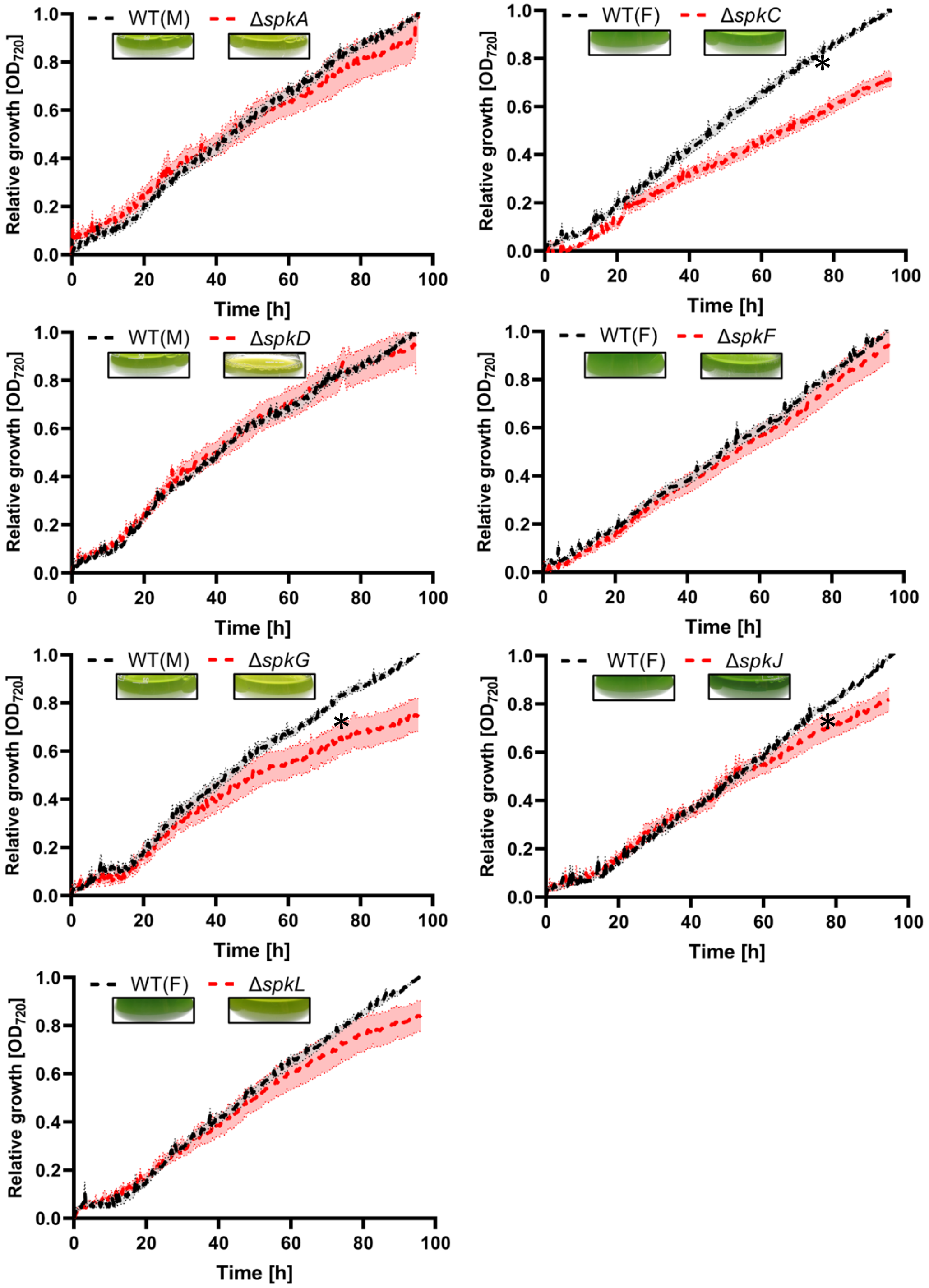

Suppl. Figure S3: Growth of different *spk* mutants in response to low inorganic carbon conditions. Cells of the *Synechocystis* wild type (WT) and mutant strains were long-term acclimated to high CO_2 (5%, HC) and were transferred at time point 0 h to ambient air CO_2 (0.04%, LC) conditions. All strains were grown at continuous light of 100 µmol photons m^{-2} s^{-1} and 30°C. Growth is shown as relative increase of optical density at 720 nm (*, p> 0.05; n =12). The insets show the optical appearance of cyanobacterial suspensions acclimated to LC for 4 days.

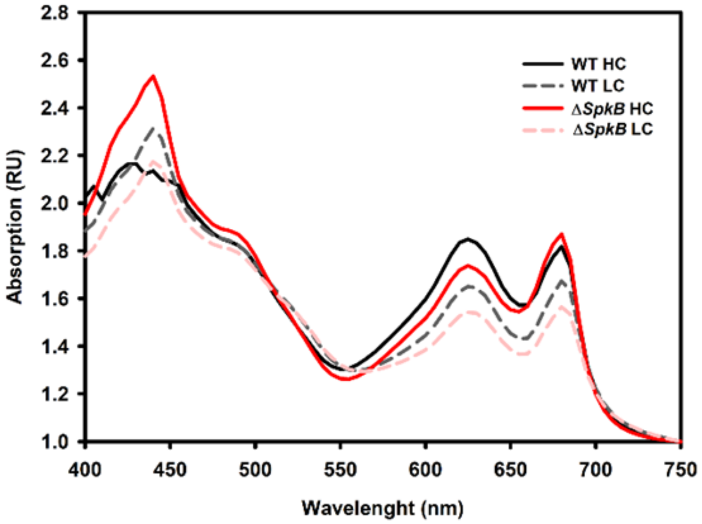

Suppl. Figure S4: Pigmentation of the *Synechocystis* wild type (WT) and Δ*spkB* grown either under HC or LC conditions. Combined spectra (400-750nm) of cell suspensions grown under different Ci conditions (HC n=3; LC n=4). Data are normalized to OD_{750}.

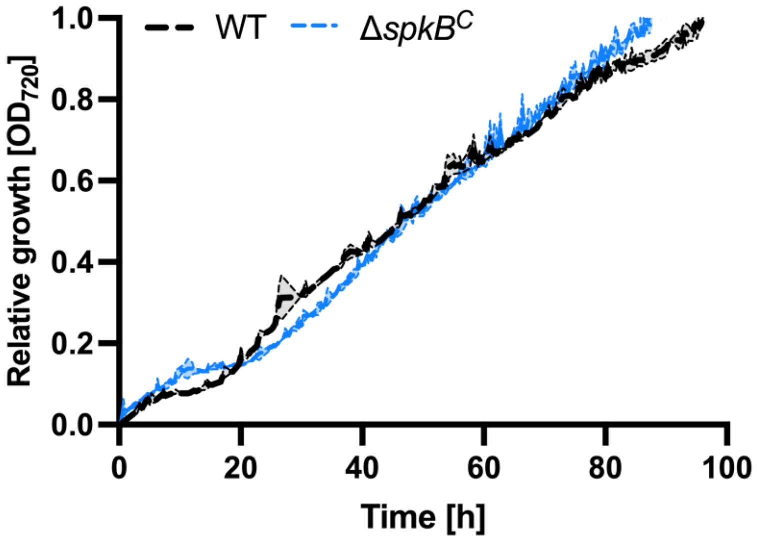

Suppl. Figure S5: Growth of a strain ectopically expressing *spkB* in response to low inorganic carbon conditions. Cells of the wild type (WT) and a clone expressing *spkB* ectopically on the plasmid pVZ322, Δ*spkB*^{c} were pre-cultivated under HC conditions with full antibiotic concentration (50 µg/ml kanamycin, 20 µg/ml spectinomycin) for 3 days. Cell suspensions were then adjusted to OD_{750} 0.2 with BG11 pH 7.0 and grown for 4 days in the Multicultivator at ambient air (LC conditions) and a light quantity of 100 µmol photons m^{-2} s^{-1}. (n=3).

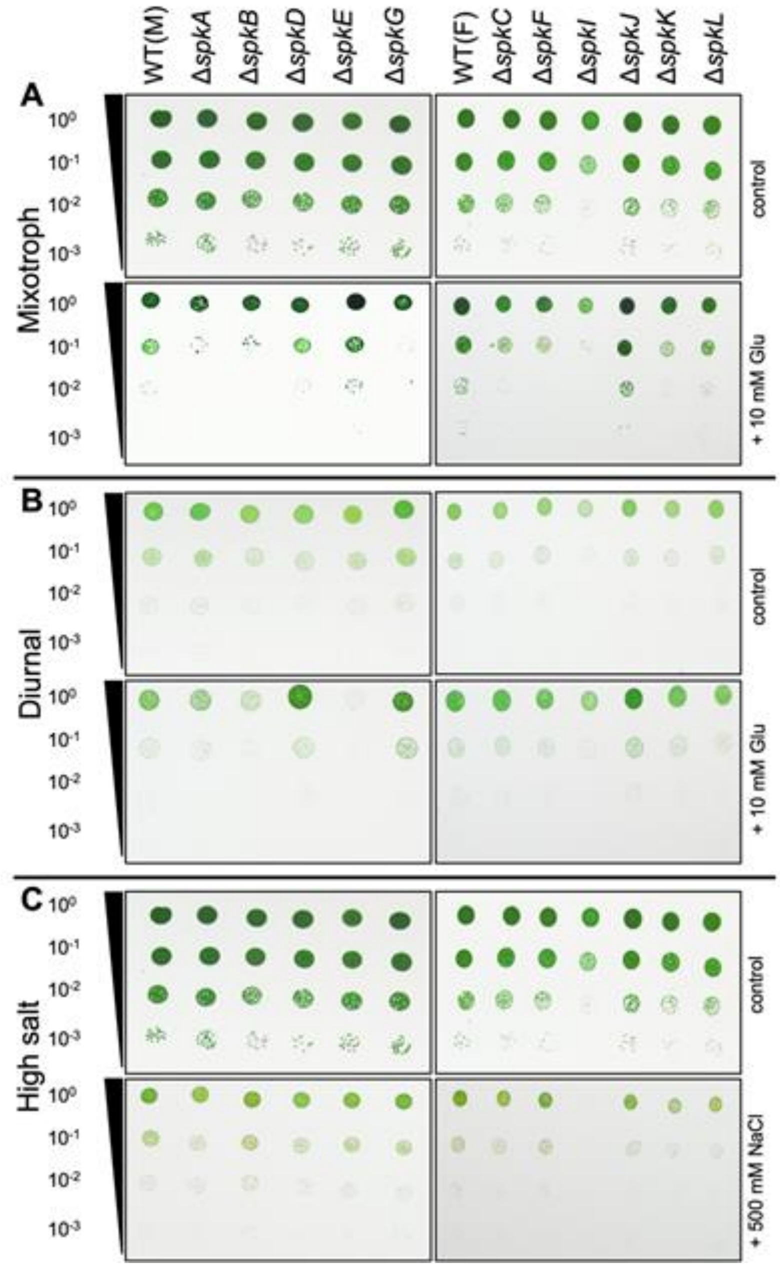

Suppl. Figure S6: Sensitivity of kinase-deficient mutants Δ*spkA-L* towards different environmental conditions (Drop dilution assay). Kinase-deficient mutants and their respective WT were pre-cultivated in shaking flasks with BG11 (TES pH 8.0) at continuous light of 100 µmol photons m^{-2} s^{-1} and ambient air (LC, 0.04% CO) until they reached the desired OD_{750}. Upon drop dilution assay, cell suspensions were adjusted to the OD_{750} of 0.2 with BG11 (TES pH 8.0). Subsequently, the cell suspension was serial diluted to 1:10, 1:100 and 1:1000. 2 µl of the diluted suspensions were spotted on solid BG11 plates (TES pH 8.0, 1.5% bacto agar) containing different supplements. **A:** Mixotrophic conditions in continuous light without (control) or with 10 mM glucose. **B:** Diurnal growth conditions (12 h light of 75 µmol photons m^{-2} s^{-1}/ 12 h darkness) without (control) or with 10 mM glucose. **C:** High salt conditions in continuous light without (control) or with 500 mM NaCl. Plates were incubated at 30°C for 4 days. Pictures were taken together with their respective control plate.

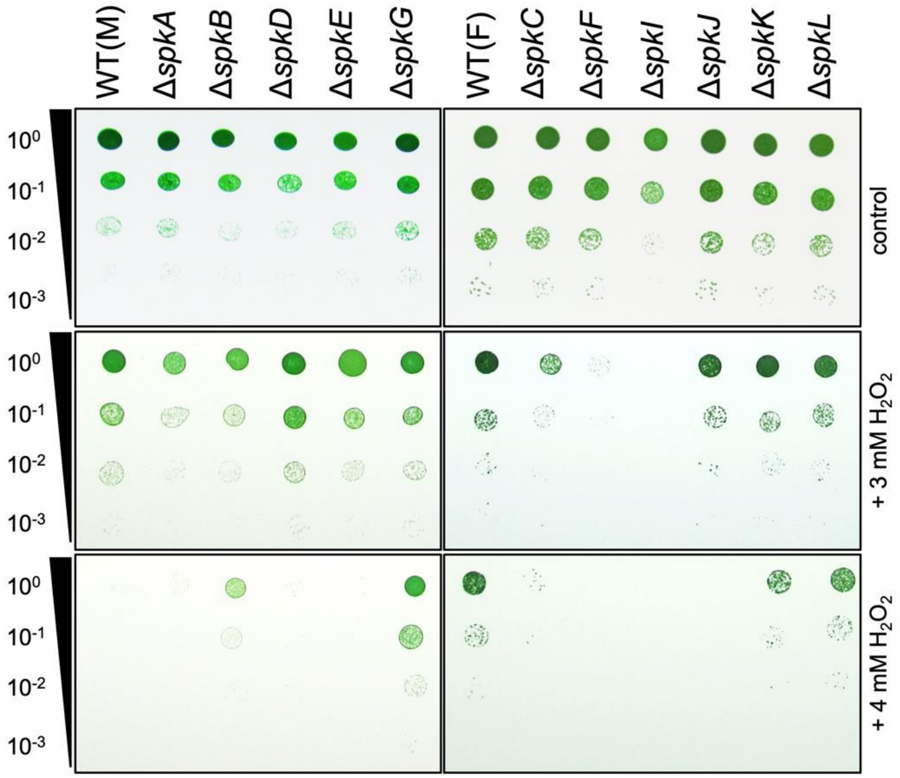

Suppl. Figure S7: Sensitivity of kinase-deficient mutants towards external H_2O_2. *Synechocystis* was pre-cultivated in shaking flasks in BG11 (TES pH 8.0) at 30°C and a light quantity of 100 µmol photons m^{-2} s^{-1} in an LC environment until they reached the desired OD750. Synechocystis was then adjusted to OD750 of 0.4 and probed with either 3 mM or 4 mM of H_2O_2 followed by an incubation under grow light conditions for 1h. After incubation, the suspension was diluted in serial dilution to 1:10, 1:100 and 1:1000. 2 µl of the dilution series were spotted on solid BG11 plates (TES pH 8.0; 1.5 % bacto agar). *Synechocystis* strains recovered for 4 d at 30°C and 100 µmol photons m^{-2} s^{-1} at constant light. Photos were taken together with their respective control plate.

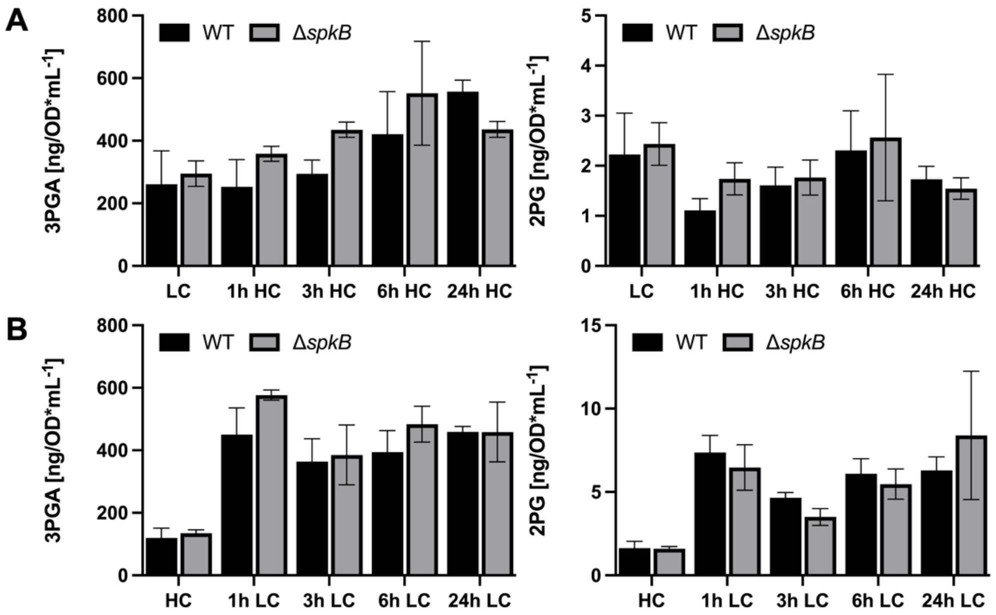

Suppl. Figure S8: Steady state values of 3PGA and 2PG in cells of the wild type (WT) and Δ*spkB* shifted from LC to HC (A) and HC to LC (B) conditions. *Synechocystis* was pre-acclimated to either HC (BG11 pH 8.0) and LC (BG11 pH 7.0) conditions and subsequently adjusted to the OD_{750} of 1. Upon shift the medium was removed by centrifugation and the pellets were re-suspended in either BG11 pH 8.0 (A) or BG11 pH 7.0 (B) and bubbled with either HC (A) or ambient air (LC; B). 5 ml culture were taken after 1; 3; 6 and 24 h after the initial shift and used for LC-MS analysis.

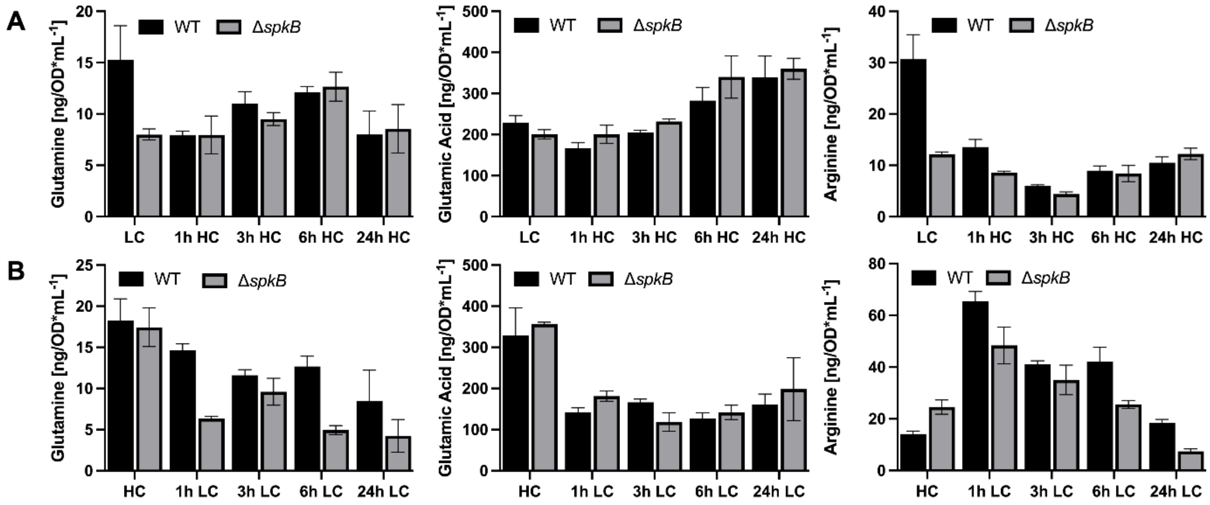

Suppl. Figure S9: Amino acid contents in the *Synechocystis* wild type (WT) and Δ*spkB* grown under different inorganic carbon conditions. *Synechocystis* strains were pre-acclimated to either LC (**A**, 0.04% CO_2, BG11 pH 7.0) or HC (**B**, 5% CO_2, BG11 pH 8.0) conditions. Upon shift, the medium was removed by centrifugation and the pellets were re-suspended in either BG11 pH 8.0 (**A**) or BG11 pH 7.0 (**B**) and then aerated with either CO_2-enriched (HC, **A**) or ambient air (LC, **B**). Cells from 5 ml culture were harvested 1, 3, 6, and 24 h after the initial shift and used for LC-MS analysis.

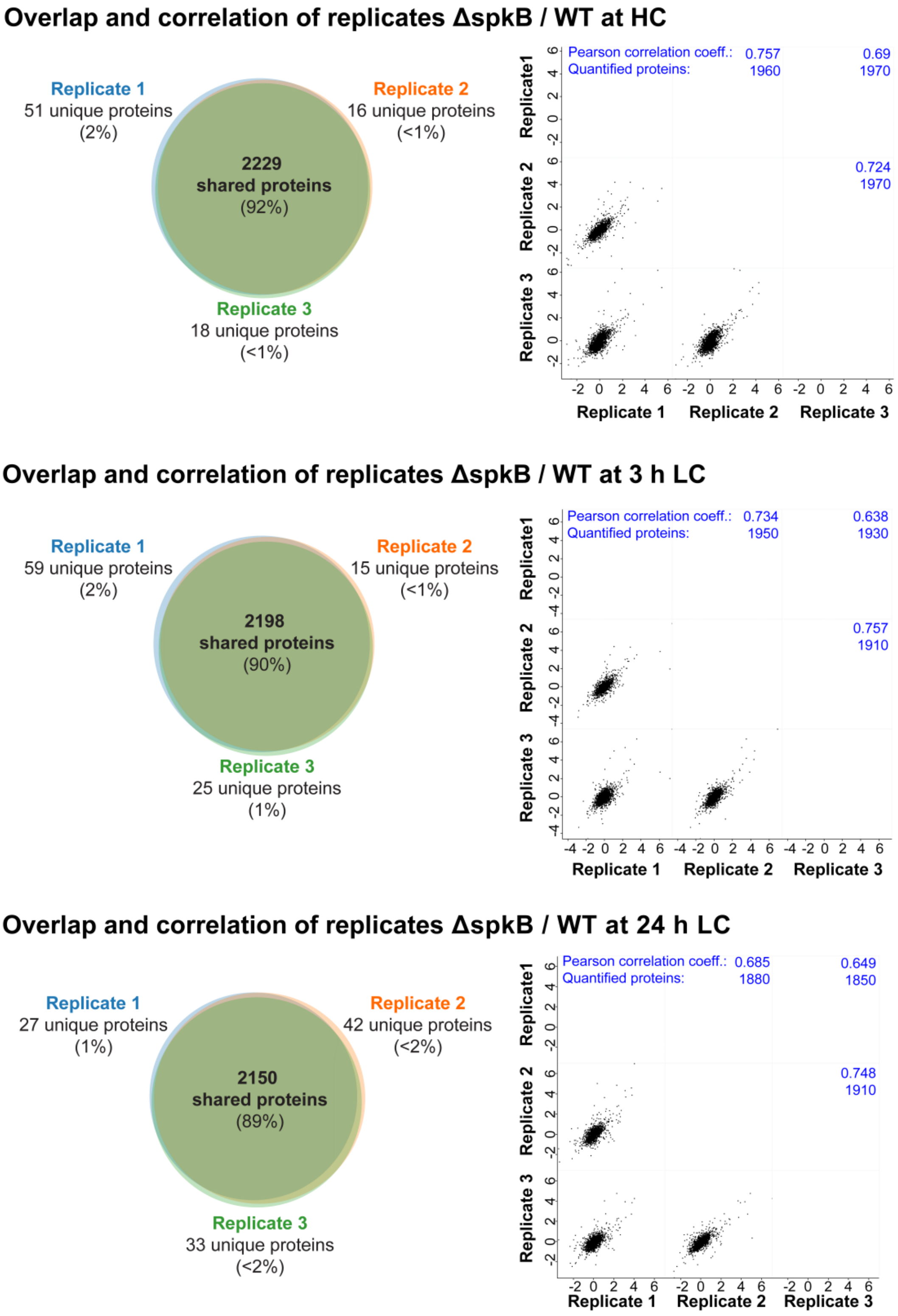

Suppl. Figure S10: Overlap and intensity-based correlation of quantified proteins between wild type (WT) and Δ*spkB* among replicates in the proteome analysis. Left: Venn diagrams indicate the numbers of detected and shared proteins of three independent replicates. **Right**: Shown is the correlation of protein intensities (in $\log_{10}$ scale) between independent replicates for each sampling point.

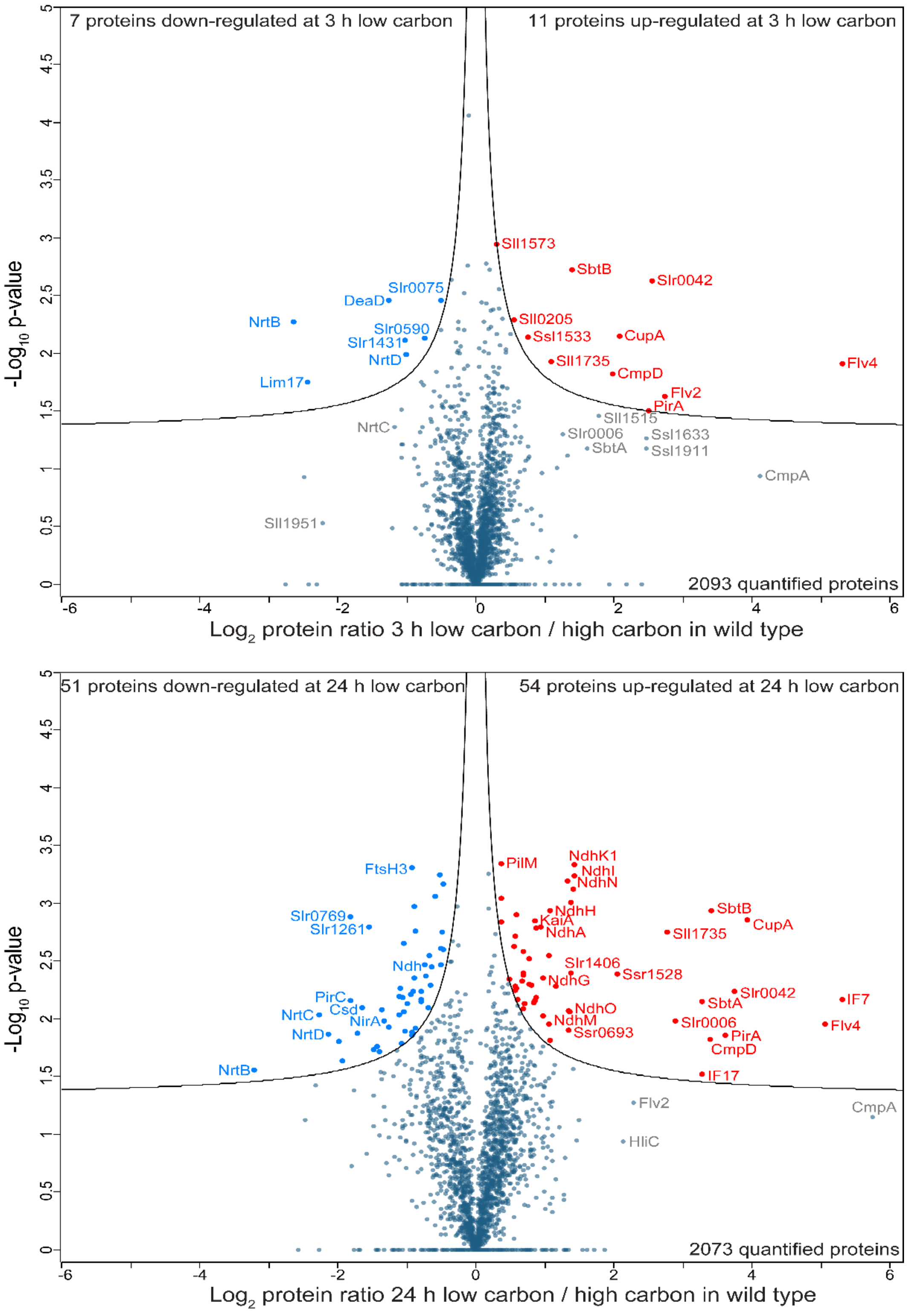

Suppl. Figure S11: Change of the proteome in response to low carbon conditions in the wild type (WT). Displayed are abundance ratios of quantified proteins between 3 h LC (top) and 24 h LC (bottom) relative to HC conditions. Proteins with significant changes (indicated in blue and red) were analyzed in a one-sample *t*-test (p=0.05; S0=0.5)

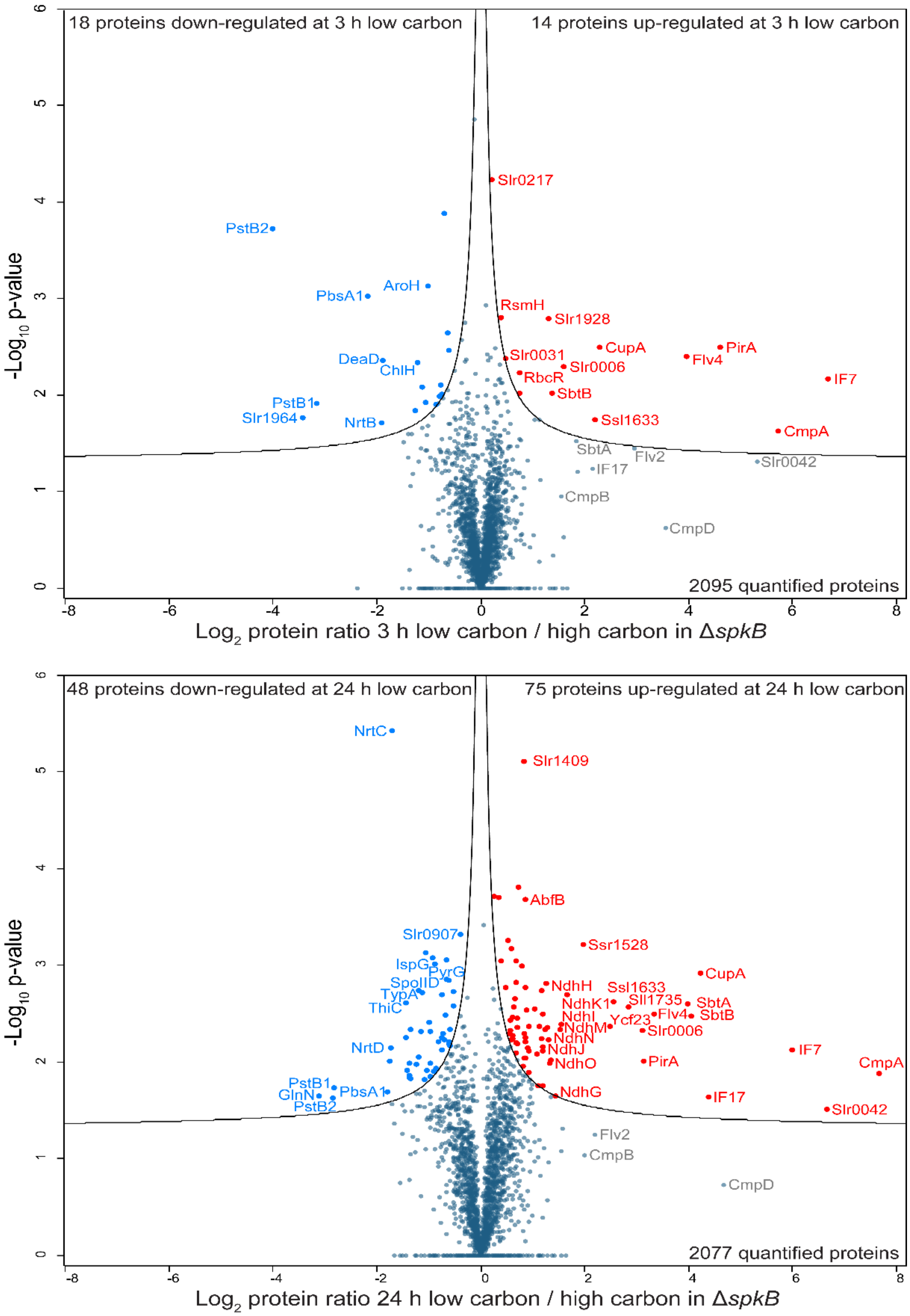

Suppl. Figure S12: Change of the proteome in response to low carbon conditions in the mutant Δ*spkB*. Displayed are abundance ratios of quantified proteins between 3 h LC (top) and 24 h LC (bottom) relative to HC conditions. Proteins with significant changes (indicated in blue and red) were analyzed in a one-sample *t*-test (p=0.05; S0=0.5)

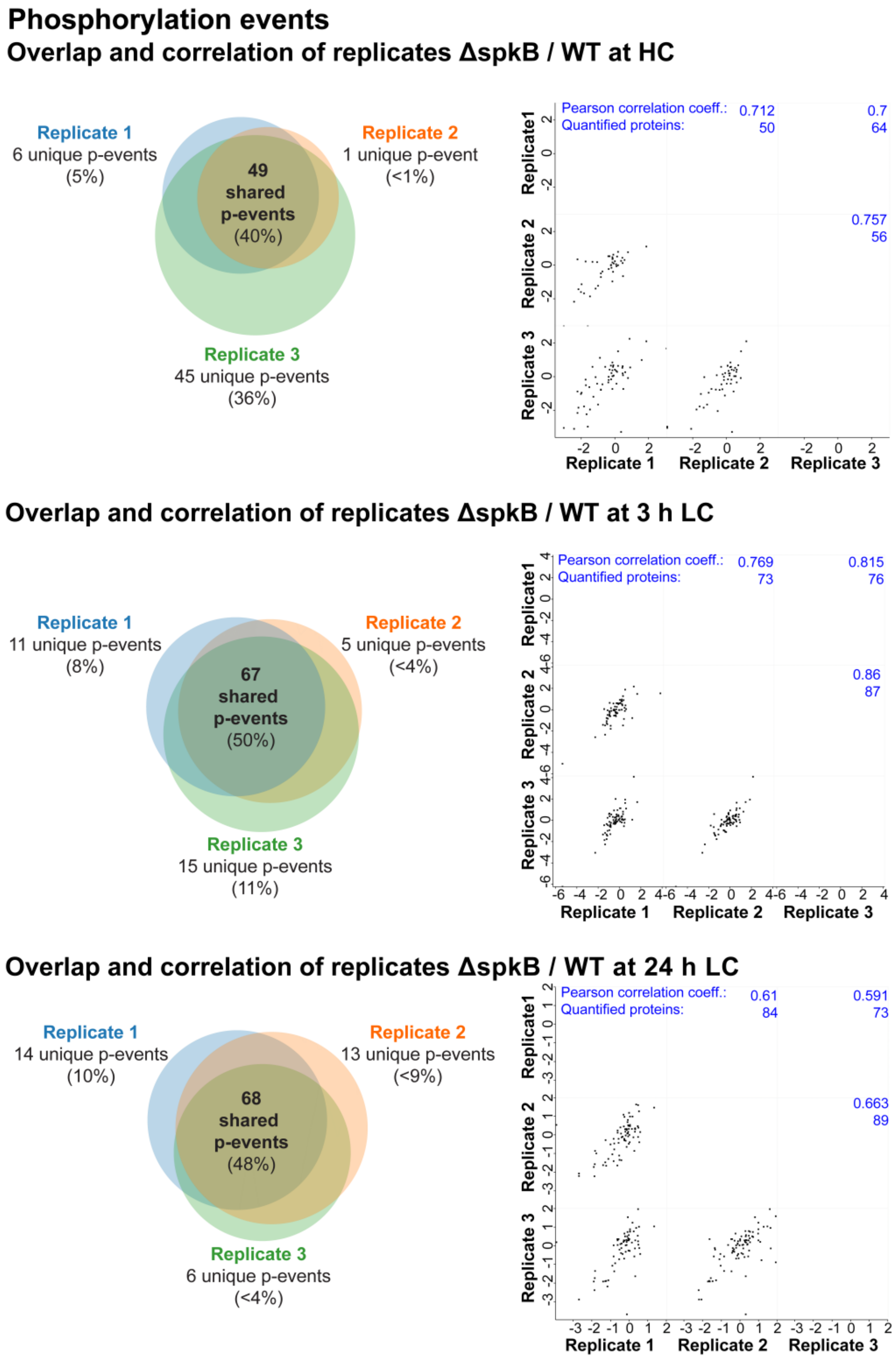

Suppl. Figure S13: Overlap and intensity-based correlation of quantified phosphorylation events between WT and Δ*spkB* among replicates in the phospho-proteome analysis. Left: Venn diagrams indicate the numbers of detected and shared p-events of three independent replicates. **Right**: Shown is the correlation of phospho-peptide intensities (in log_2 scale) between independent replicates for each sampling point.

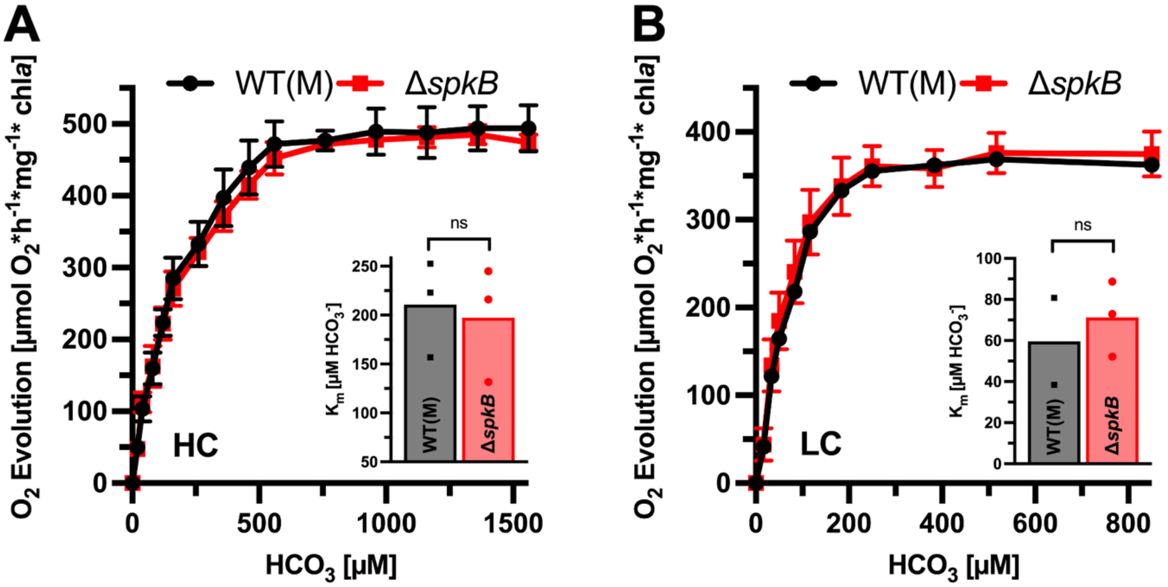

Suppl. Figure S14: Inorganic carbon-Ci-dependent O_2 evolution rates. Photosynthetic activity of wild-type (WT (M)) or Δ*spkB* mutant cells grown at HC (**A**) or LC (**B**) conditions was measured with increasing bicarbonate concentrations using the oxygen electrode under saturating light conditions. Inlets represent the calculated K_m values. (n = 3, student's *t*-test, two-tailed, * p<0.05)
