# Supplementary material for: The Role of Serine/Threonine-Specific Protein Kinases in Cyanobacteria - SpkB Is Involved in Acclimation to Fluctuating Conditions in Synechocystis sp. PCC 6803
Source: Mol Cell Proteomics. 2023 Oct 4;22(11):100656. doi: 10.1016/j.mcpro.2023.100656 (PMC10651672; doi:10.1016/j.mcpro.2023.100656)

Supplemental data for the manuscript:

The role of serine/threonine-specific protein kinases in cyanobacteria - SpkB is involved in acclimation to fluctuating conditions in *Synechocystis* sp. PCC 6803

Thomas Barske[1]*, Philipp Spät[2,3]*, Hendrik Schubert[4], Peter Walke[1], Boris Maček[3], Martin Hagemann[1,5]**

* - the first two authors contributed equally to the study

1 – Institute of Biosciences, Department of Plant Physiology, University of Rostock, Rostock, Germany

2 - Interfaculty Institute of Microbiology and Infection Medicine Tübingen, Department of Organismic Interactions, University of Tübingen, Tübingen, Germany

3 - Interfaculty Institute for Cell Biology, Department of Quantitative Proteomics, University of Tübingen, Tübingen, Germany

4 - Institute of Biosciences, Department of Aquatic Ecology, University of Rostock, Rostock, Germany

5 – Interdisciplinary Faculty, Department Life, Light and Matter, University of Rostock, Rostock, Germany

Corresponding author: Martin Hagemann, Institut für Biowissenschaften, Abteilung Pflanzenphysiologie, Universität Rostock, A.-Einstein-Str. 3, Rostock D-18059, Germany; Tel: +49(0)3814986110; Fax: +49(0)3814986112; Email: martin.hagemann@uni-rostock.de

Supplemental spectra of phosphopeptides

20210828_PS_SCy031_phospho_R3_TiO2_LC3h_F3 − Scan number: 7004

Gene Names	Charge	m/z	Mass	Mass error [Da]	Mass error [ppm]	Retention time	PEP	Score	Precursor Intensity
sll0018	2	527.7319	1053.449	−0.00011725	−0.22823	11.893	2.0968e−05	137.89	20781520

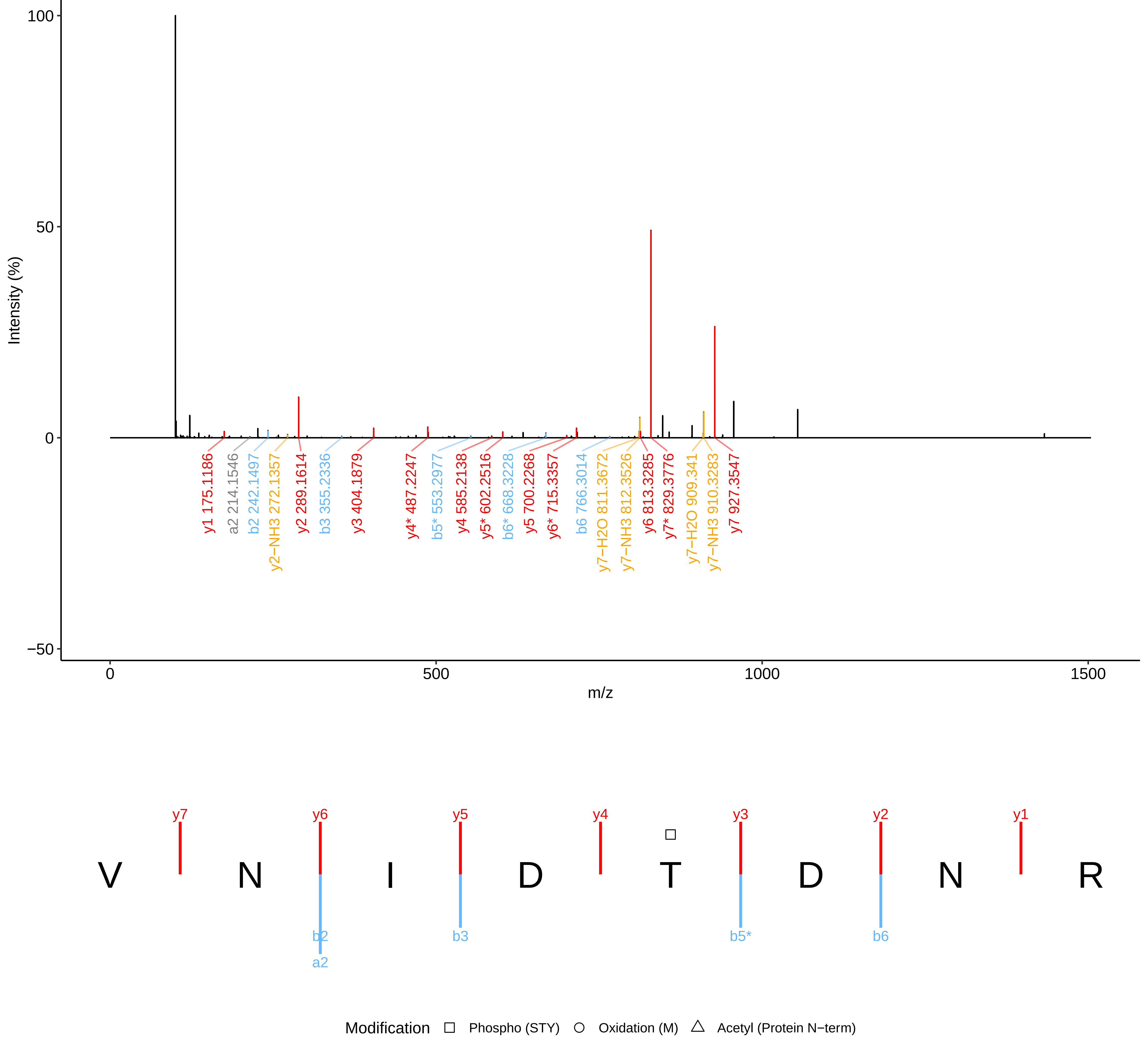

20211212_PS_SCy031_proteome_R1_24hLC_F1 − Scan number: 10035

Gene Names	Charge	m/z	Mass	Mass error [Da]	Mass error [ppm]	Retention time	PEP	Score	Precursor Intensity
sll0019	2	683.8606	1365.707	−0.0014713	−2.1514	16.219	0.015306	62.107	24251556

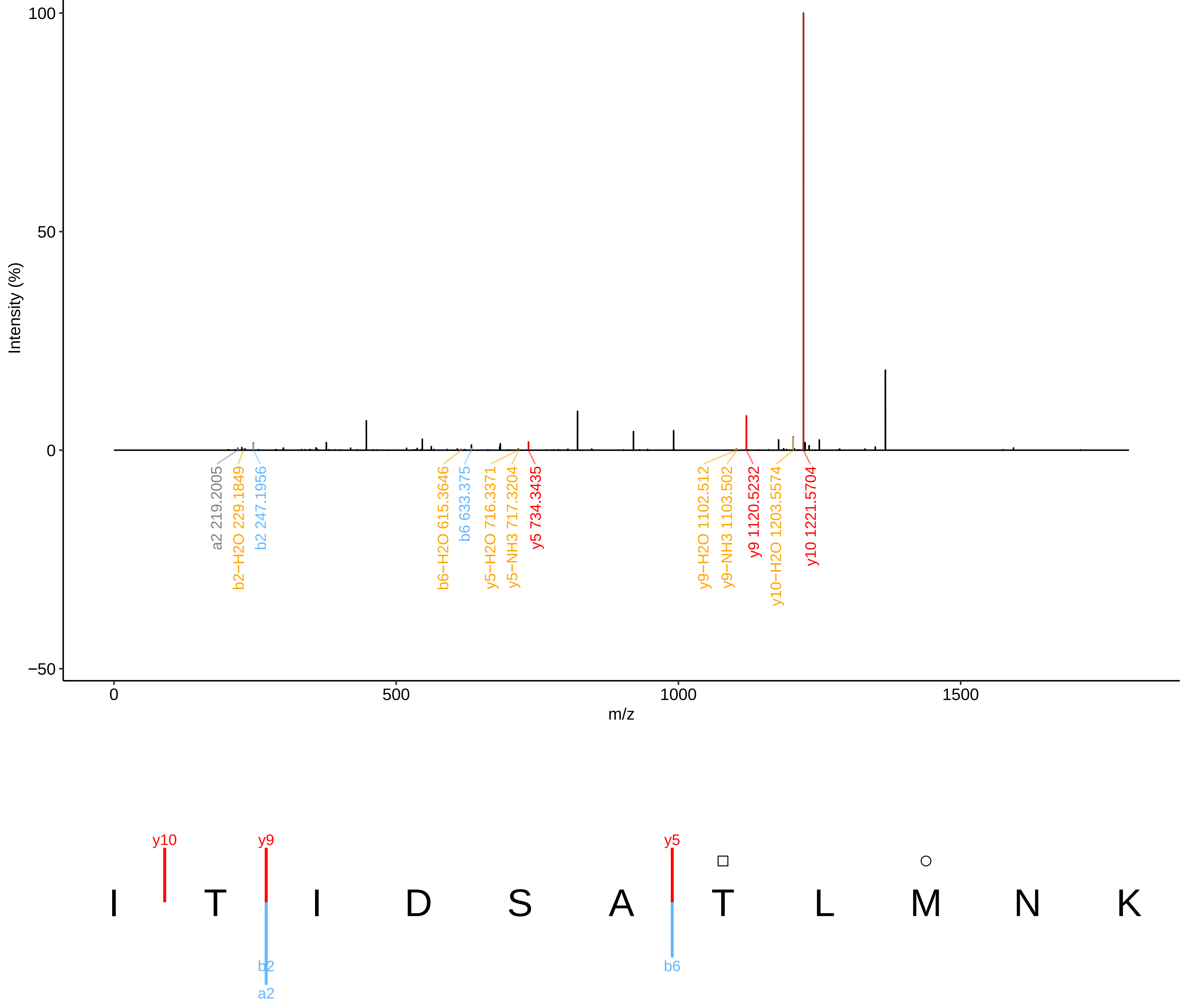

Modification □ Phospho (STY) ○ Oxidation (M) △ Acetyl (Protein N−term)

20210828_PS_SCy031_phospho_R2_TiO2_LC3h_F2 − Scan number: 26315

Gene Names	Charge	m/z	Mass	Mass error [Da]	Mass error [ppm]	Retention time	PEP	Score	Precursor Intensity
sll0068	2	994.0141	1986.014	0.0016488	1.6579	36.04	6.2163e−100	264.52	11869583

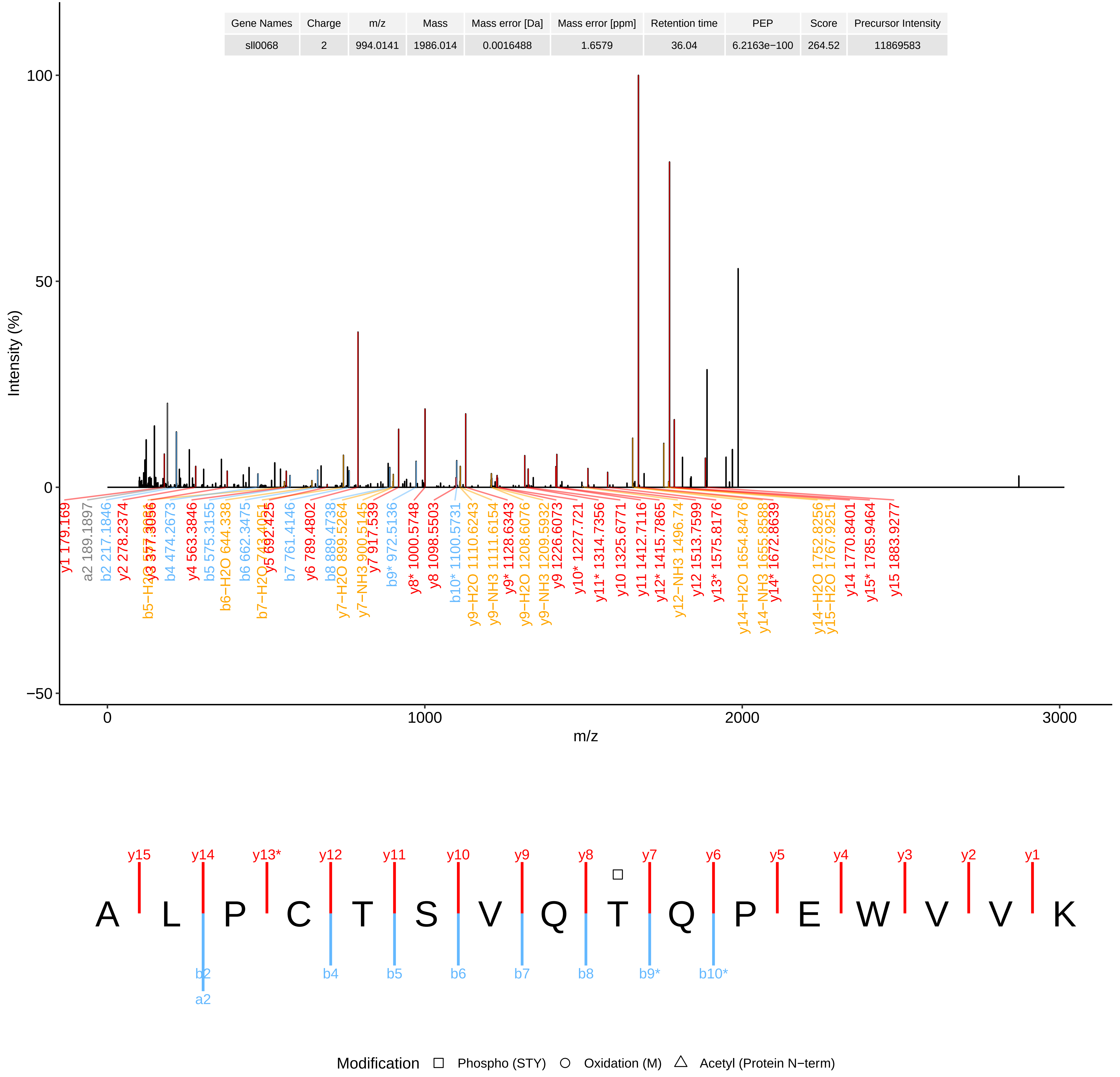

20210828_PS_SCy031_phospho_R3_TiO2_HC_F1 − Scan number: 5838

Gene Names	Charge	m/z	Mass	Mass error [Da]	Mass error [ppm]	Retention time	PEP	Score	Precursor Intensity
sll0103	2	735.3724	1468.73	0.00036697	0.52477	10.219	1.4618e−08	152.34	16847516

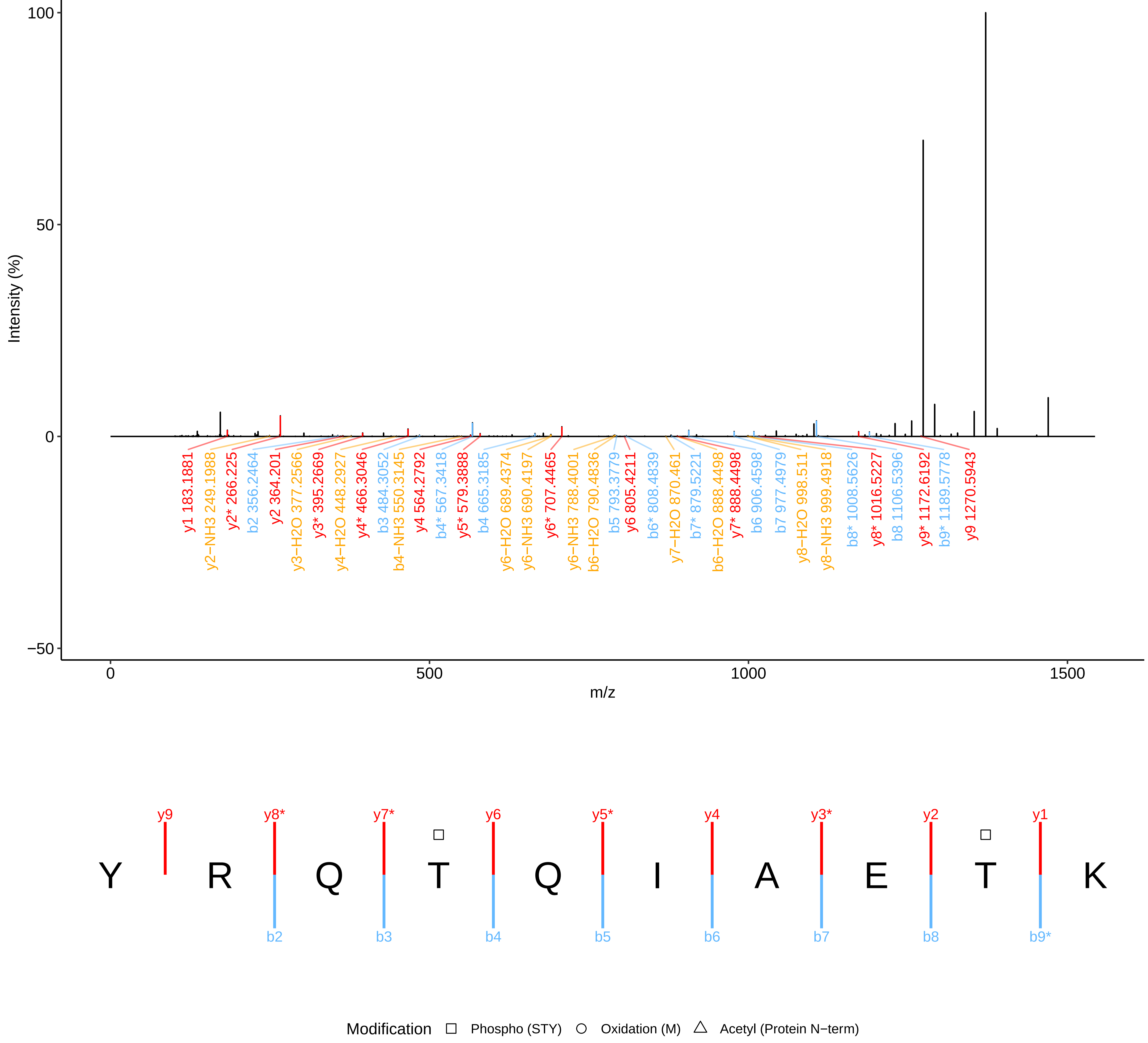

20210828_PS_SCy031_phospho_R3_TiO2_HC_F1 − Scan number: 5838

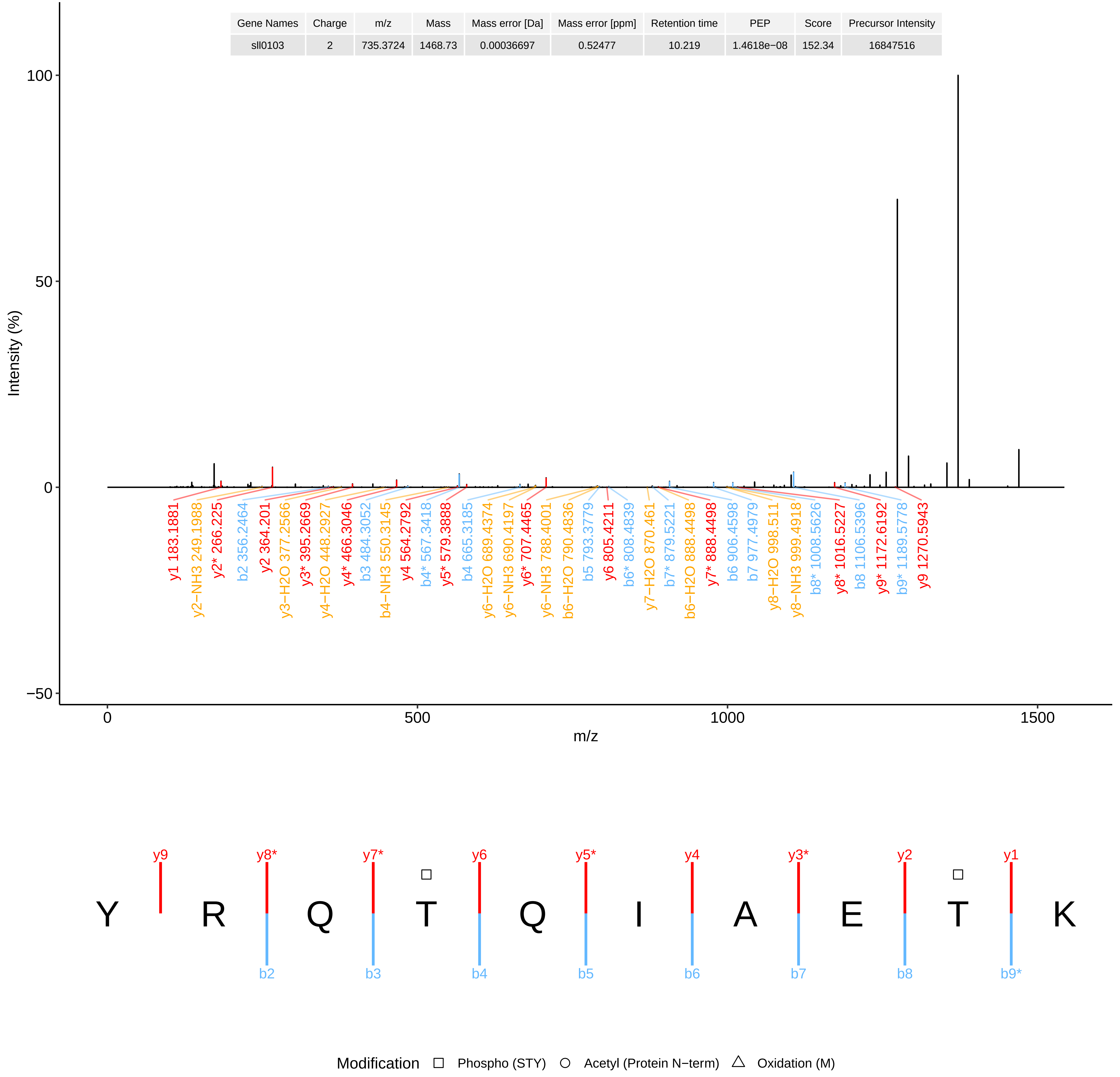

20210828_PS_SCy031_phospho_R3_TiO2_HC_F2 − Scan number: 20674

Gene Names	Charge	m/z	Mass	Mass error [Da]	Mass error [ppm]	Retention time	PEP	Score	Precursor Intensity
sll0103	2	663.828	1325.641	−0.00046911	−0.73783	28.958	6.601e−49	220.06	39796504

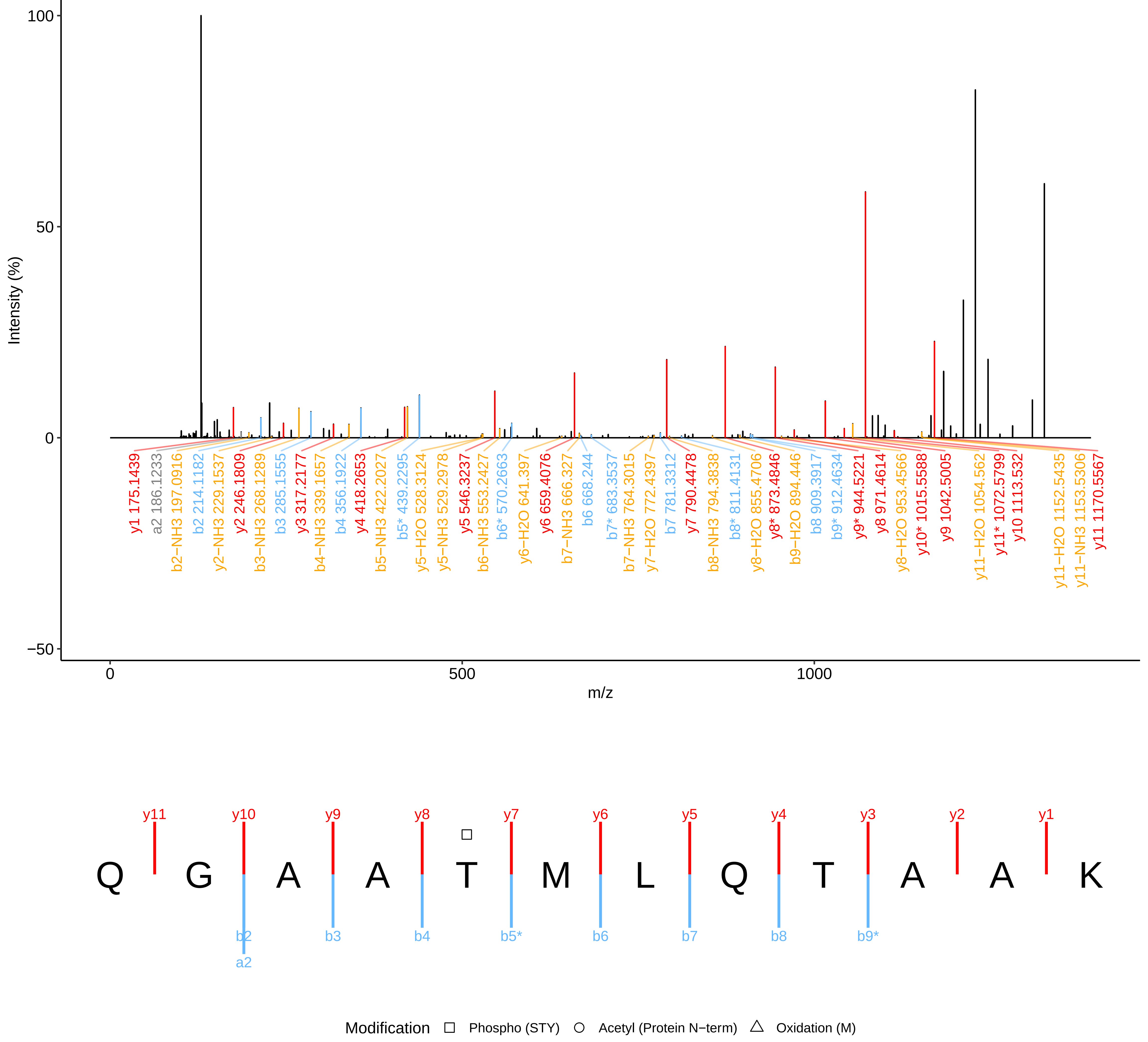

20210828_PS_SCy031_phospho_R3_TiO2_LC3h_F3 − Scan number: 9296

Gene Names	Charge	m/z	Mass	Mass error [Da]	Mass error [ppm]	Retention time	PEP	Score	Precursor Intensity
sll0103	2	500.2327	998.4508	−0.00019796	−0.41922	14.766	0.009808	83.862	13647808

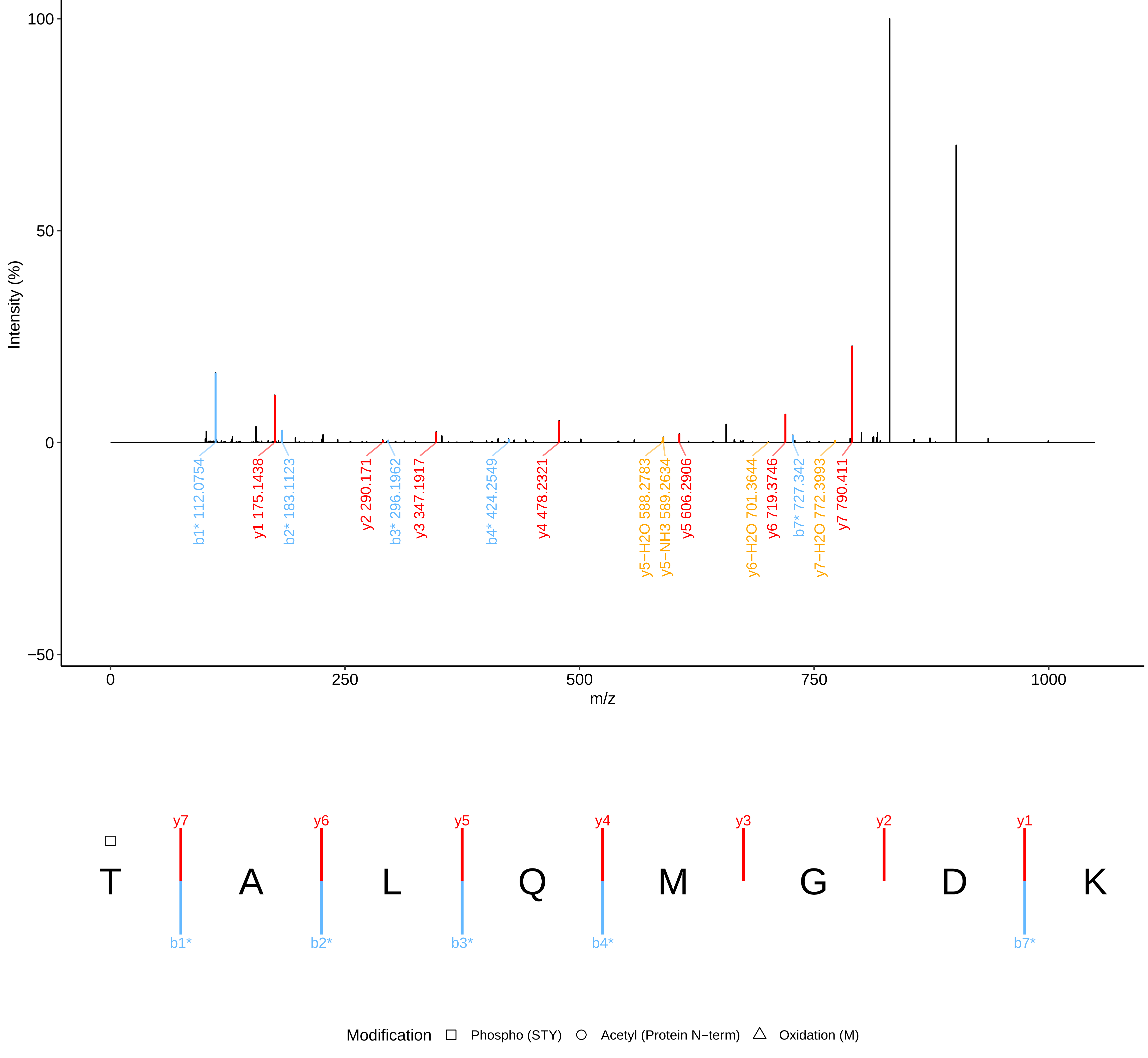

20210828_PS_SCy031_phospho_R3_TiO2_LC3h_F1 − Scan number: 22431

Gene Names	Charge	m/z	Mass	Mass error [Da]	Mass error [ppm]	Retention time	PEP	Score	Precursor Intensity
sll0103	3	914.7557	2741.245	−0.00018246	−0.20212	31.214	1.4322e−104	224.58	10666208

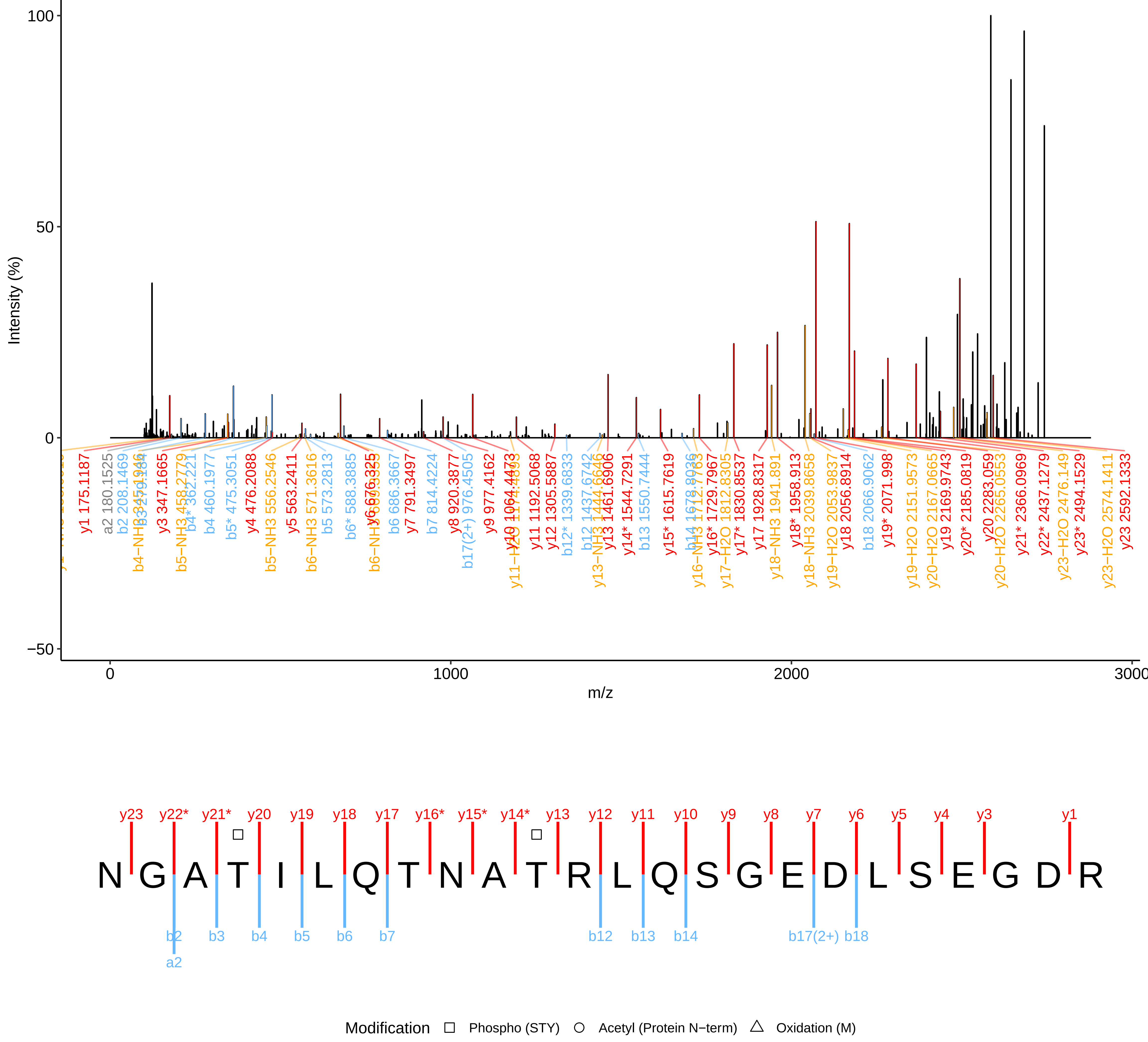

20210828_PS_SCy031_phospho_R3_TiO2_LC24h_F1 − Scan number: 14954

Gene Names	Charge	m/z	Mass	Mass error [Da]	Mass error [ppm]	Retention time	PEP	Score	Precursor Intensity
sll0103	2	724.3207	1446.627	NA	NA	21.572	0.016335	73.593	NA

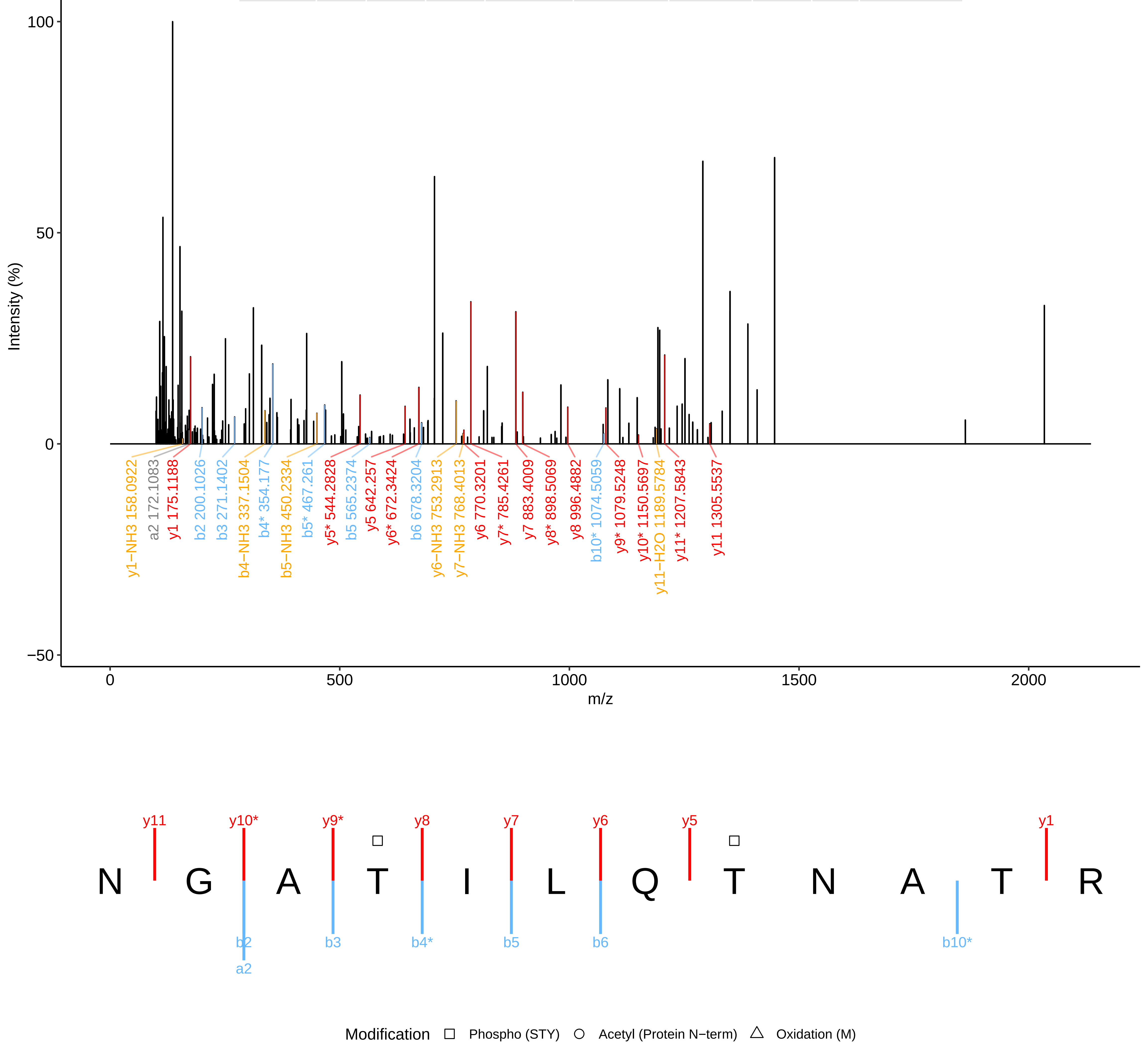

20210823_PS_SCy031_R1_phospho_LC3h_TiO2_1 − Scan number: 22788

Gene Names	Charge	m/z	Mass	Mass error [Da]	Mass error [ppm]	Retention time	PEP	Score	Precursor Intensity
sll0103	3	913.4159	2737.226	NA	NA	31.438	5.6054e−51	190.15	5744815

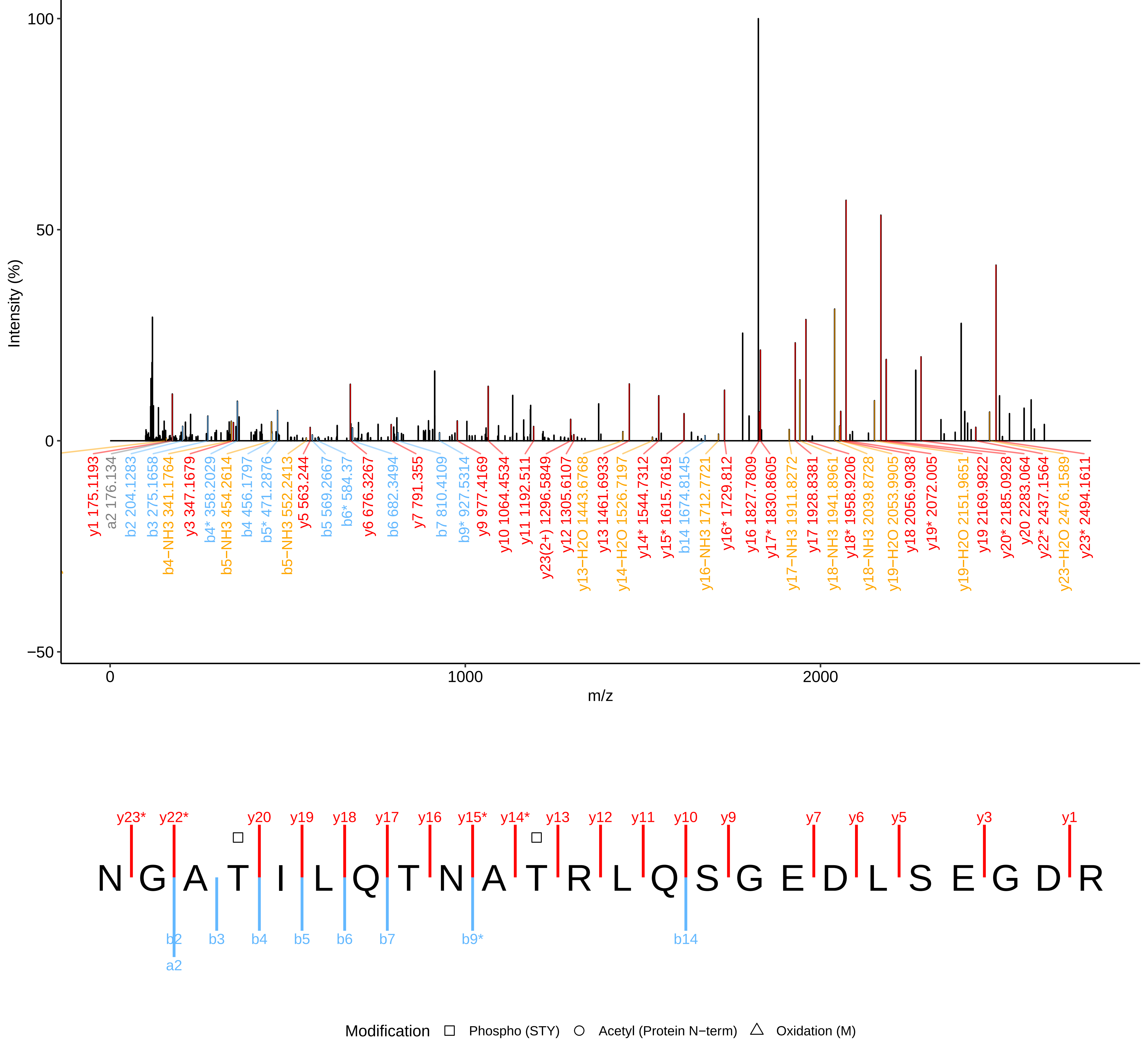

20210828_PS_SCy031_phospho_R2_TiO2_LC3h_F1 − Scan number: 10882

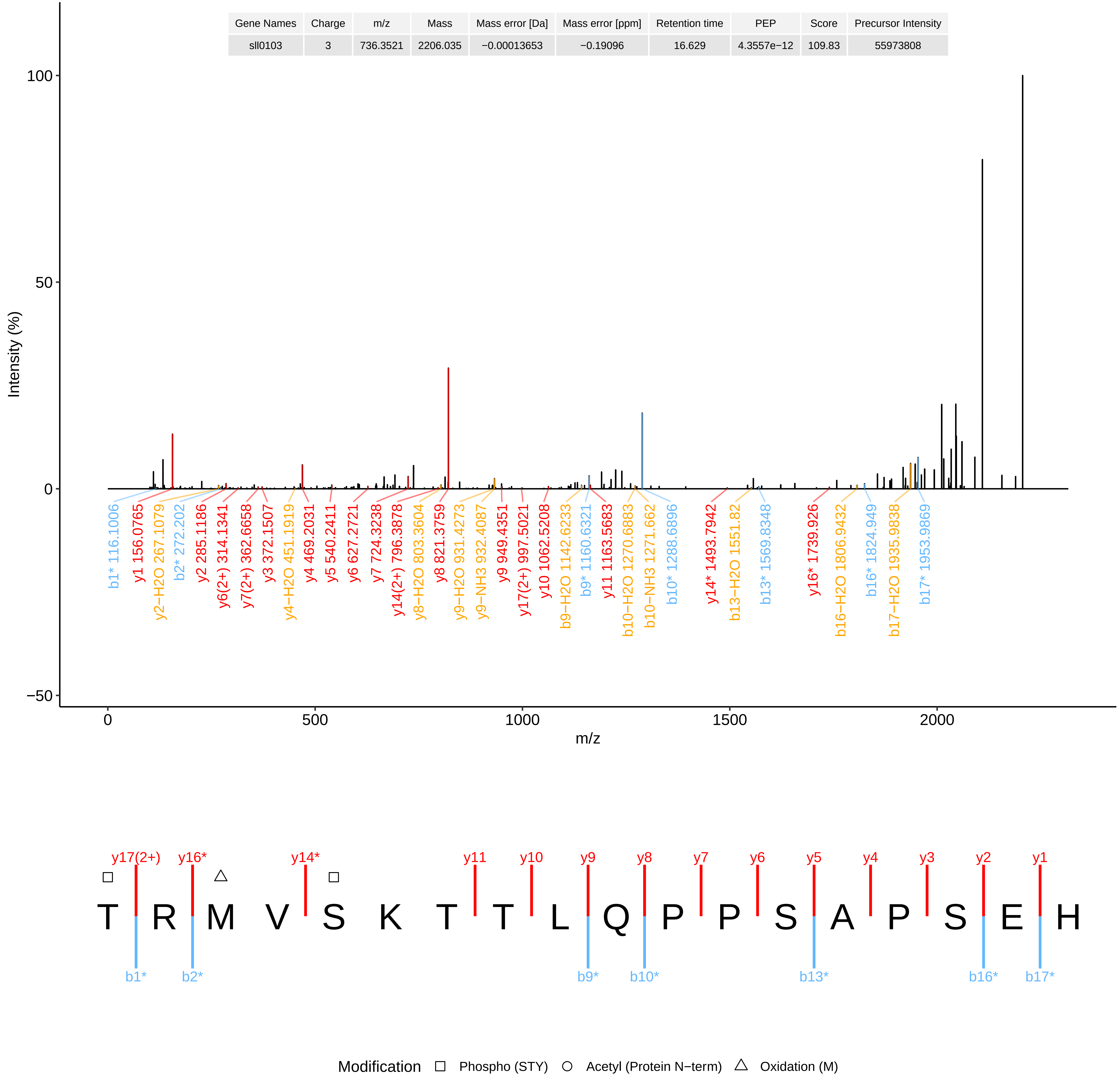

20210828_PS_SCy031_phospho_R2_TiO2_LC24h_F1 − Scan number: 13323

Gene Names	Charge	m/z	Mass	Mass error [Da]	Mass error [ppm]	Retention time	PEP	Score	Precursor Intensity
sll0103	3	733.7	2198.078	−0.00021908	−0.30872	19.727	3.6077e−07	84.156	17139028

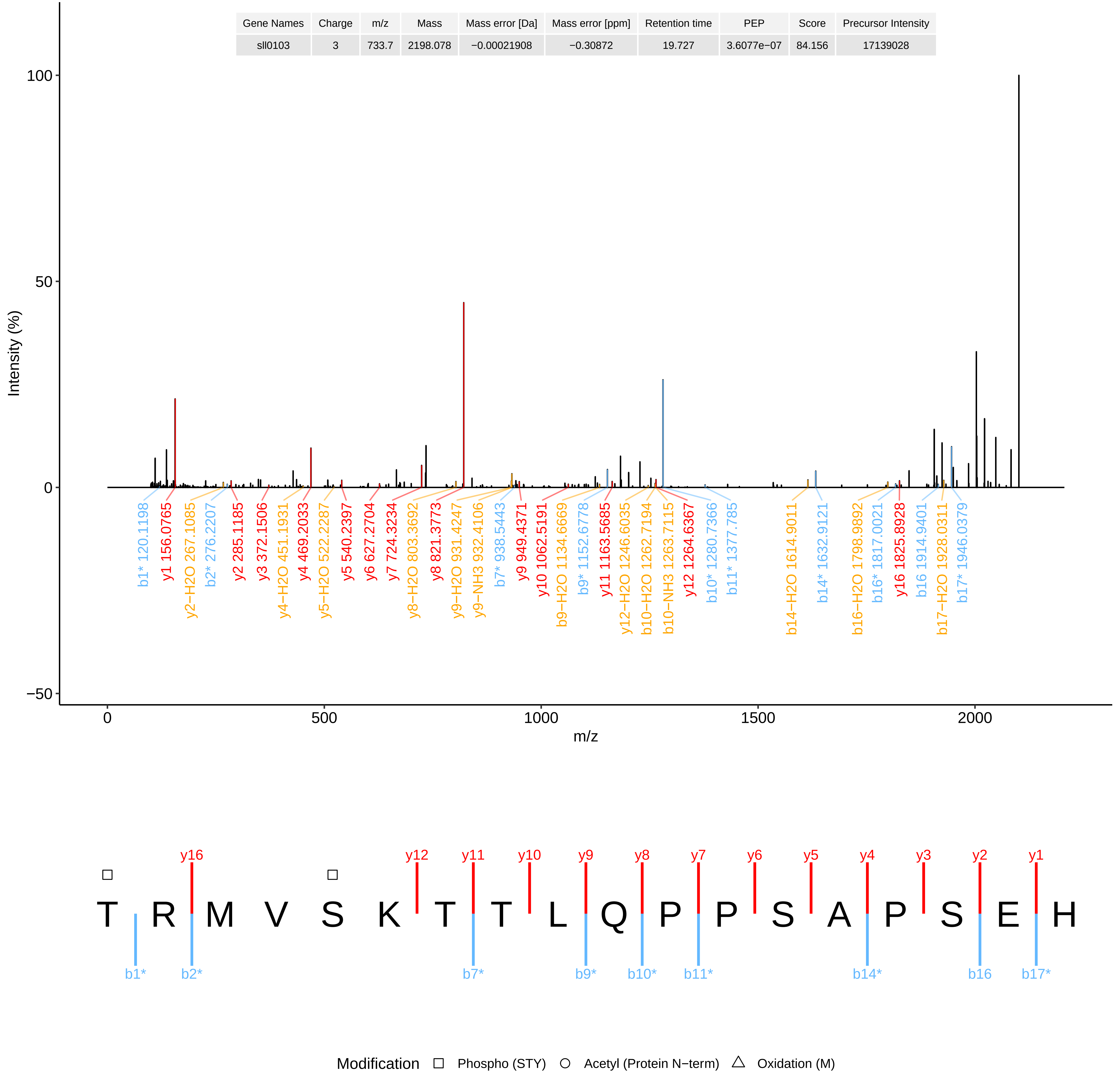

20210823_PS_SCy031_R1_phospho_LC24h_TiO2_3 − Scan number: 11976

Gene Names	Charge	m/z	Mass	Mass error [Da]	Mass error [ppm]	Retention time	PEP	Score	Precursor Intensity
sll0103	2	690.833	1379.651	−8.9685e−06	−0.01333	18.046	8.5745e−37	205.12	70906856

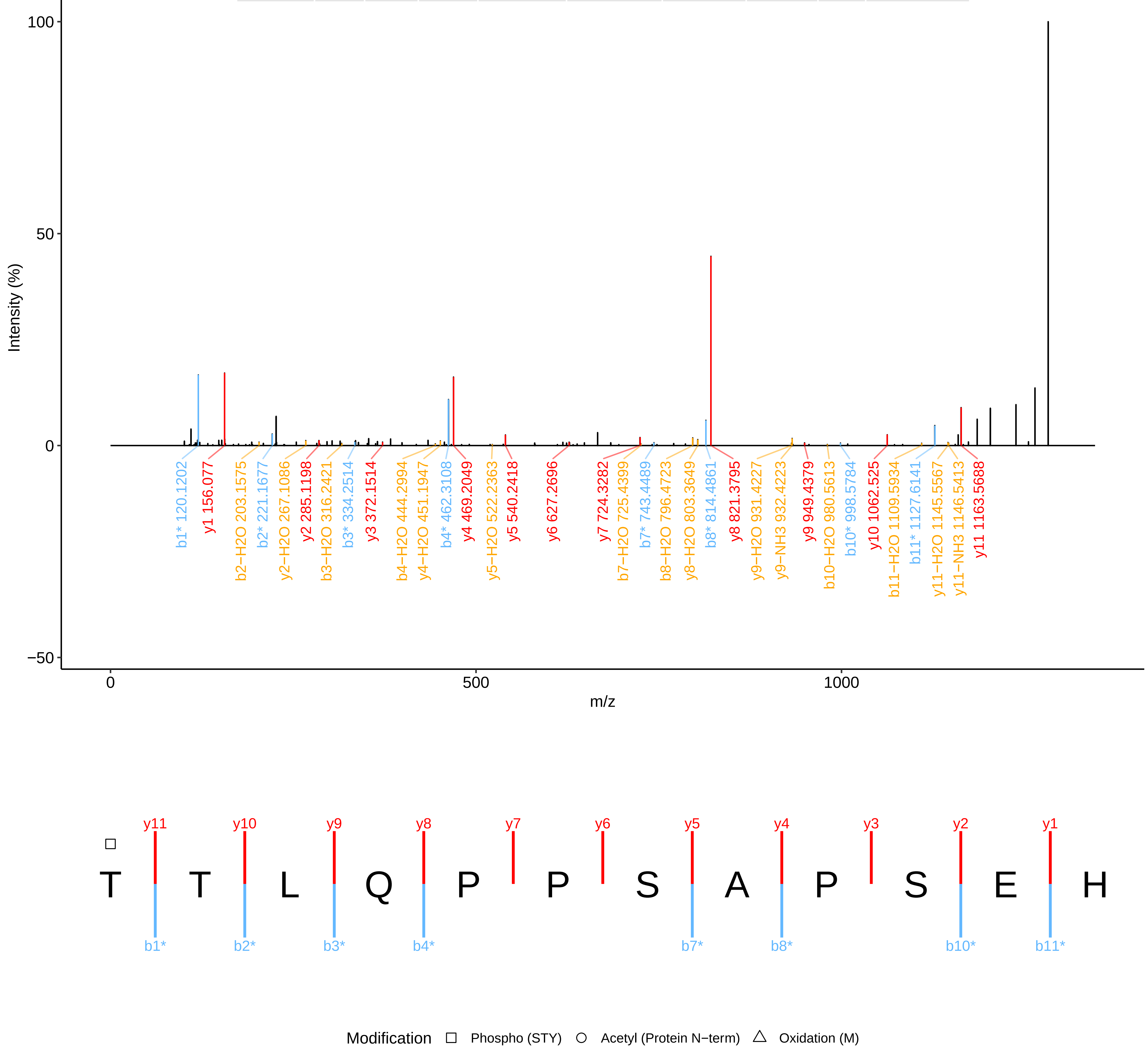

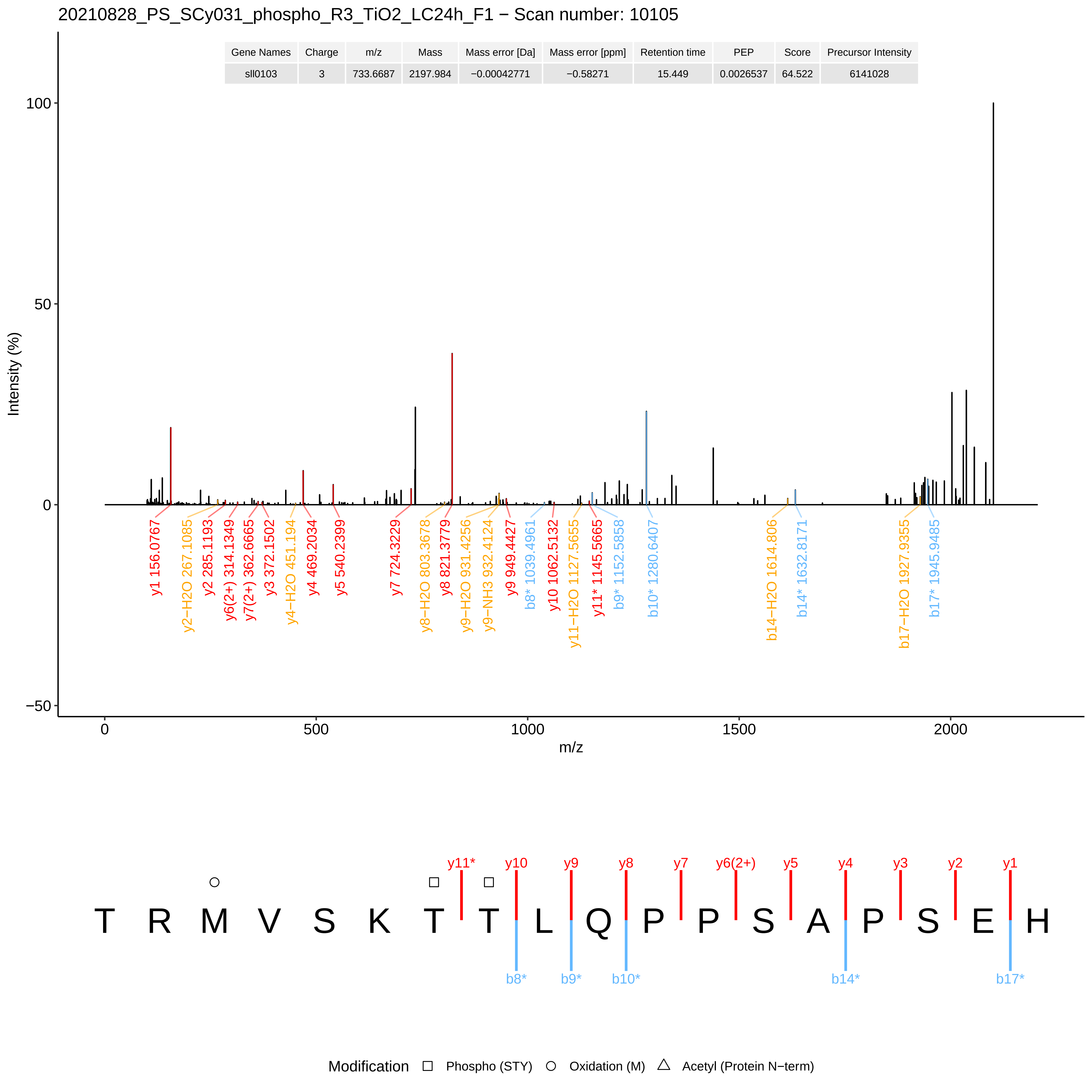

20210828_PS_SCy031_phospho_R3_TiO2_LC24h_F1 − Scan number: 10105
Gene Names | Charge | m/z | Mass | Mass error [Da] | Mass error [ppm] | Retention time | PEP | Score | Precursor Intensity
sll0103 | 3 | 733.6687 | 2197.984 | −0.00042771 | −0.58271 | 15.449 | 0.0026537 | 64.522 | 6141028
Intensity (%)
100
50
0
−50
m/z
0
500
1000
1500
2000
y1 156.0767
y2−H2O 267.1085
y2 285.1193
y6(2+) 314.1349
y7(2+) 362.6665
y3 372.1502
y4−H2O 451.194
y4 469.2034
y5 540.2399
y7 724.3229
y8−H2O 803.3678
y8 821.3779
y9−H2O 931.4256
y9−NH3 932.4124
y9 949.4427
b8* 1039.4961
y10 1062.5132
y11−H2O 1127.5655
y11* 1145.5665
b9* 1152.5858
b10* 1280.6407
b14−H2O 1614.806
b14* 1632.8171
b17−H2O 1927.9355
b17* 1945.9485
y11* y10 y9 y8 y7 y6(2+) y5 y4 y3 y2 y1
T R M V S K T T L Q P P S A P S E H
b8* b9* b10* b14* b17*
Modification
Phospho (STY)
Oxidation (M)
Acetyl (Protein N−term)

20210828_PS_SCy031_phospho_R3_TiO2_HC_F3 − Scan number: 22801

Gene Names	Charge	m/z	Mass	Mass error [Da]	Mass error [ppm]	Retention time	PEP	Score	Precursor Intensity
sll0108	2	831.3507	1660.687	0.0001592	0.19477	31.797	4.5112e−17	133.71	4792871

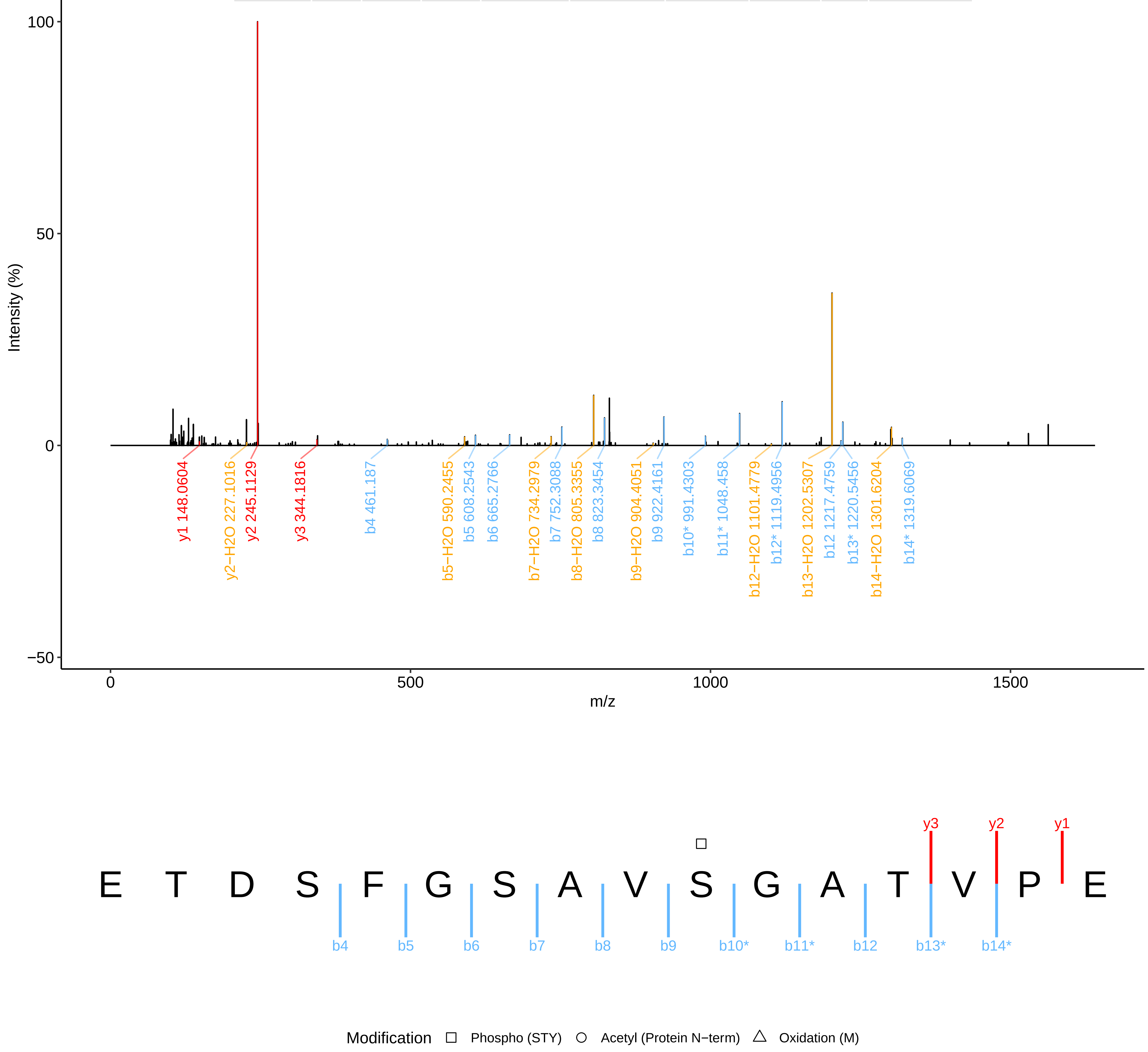

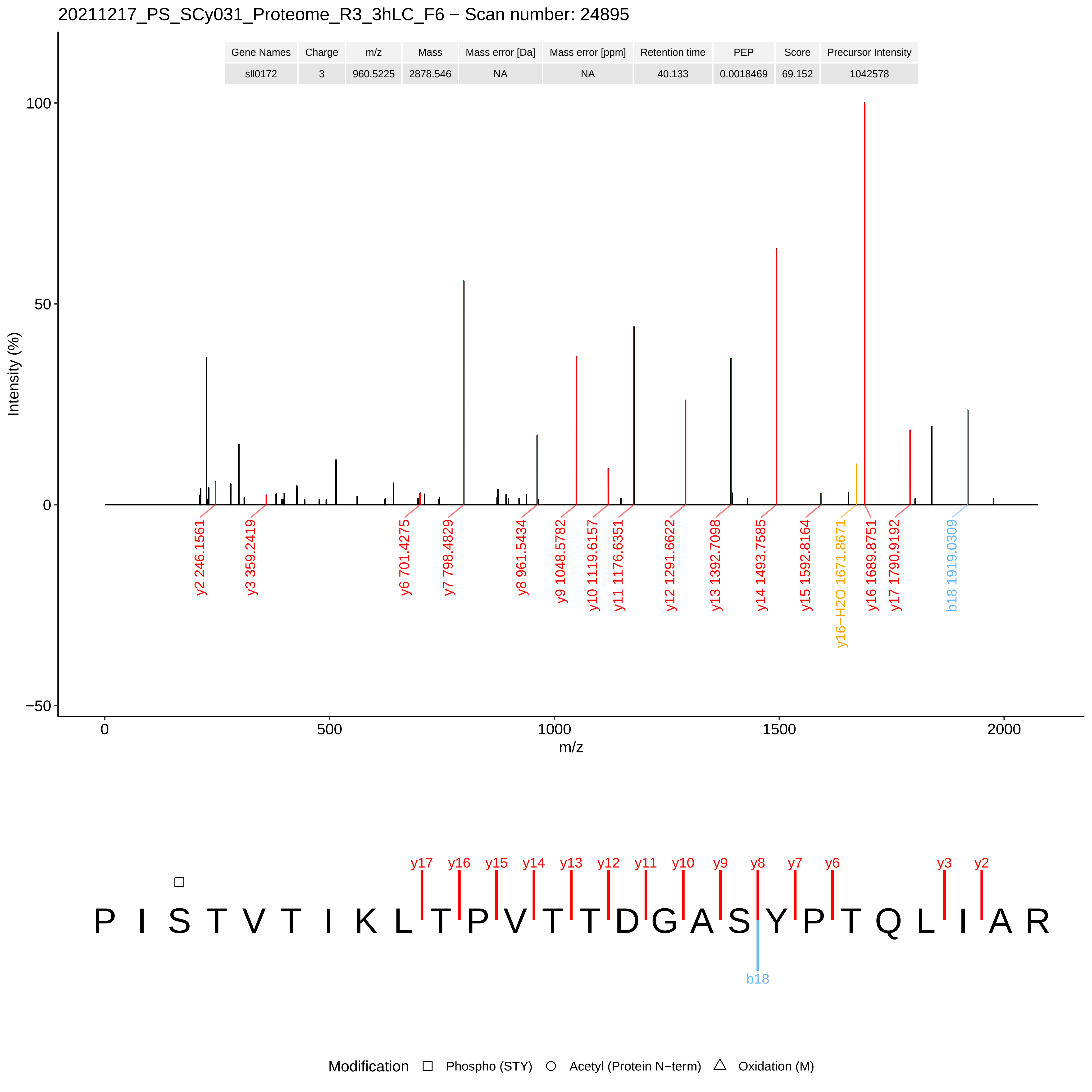
20211217_PS_SCy031_Proteome_R3_3hLC_F6 − Scan number: 24895
Gene Names | Charge | m/z | Mass | Mass error [Da] | Mass error [ppm] | Retention time | PEP | Score | Precursor Intensity
sll0172 | 3 | 960.5225 | 2878.546 | NA | NA | 40.133 | 0.0018469 | 69.152 | 1042578
Intensity (%)
m/z
y2 246.1561
y3 359.2419
y6 701.4275
y7 798.4829
y8 961.5434
y9 1048.5782
y10 1119.6157
y11 1176.6351
y12 1291.6622
y13 1392.7098
y14 1493.7585
y15 1592.8164
y16−H2O 1671.8671
y16 1689.8751
y17 1790.9192
b18 1919.0309
y17 y16 y15 y14 y13 y12 y11 y10 y9 y8 y7 y6 y3 y2
P I S T V T I K L T P V T T D G A S Y P T Q L I A R
b18
Modification □ Phospho (STY) ○ Acetyl (Protein N−term) △ Oxidation (M)

20210828_PS_SCy031_phospho_R3_TiO2_LC3h_F2 − Scan number: 15289

Gene Names	Charge	m/z	Mass	Mass error [Da]	Mass error [ppm]	Retention time	PEP	Score	Precursor Intensity
sll0271	3	594.6747	1781.002	NA	NA	22.183	0.00020239	100.97	3908663

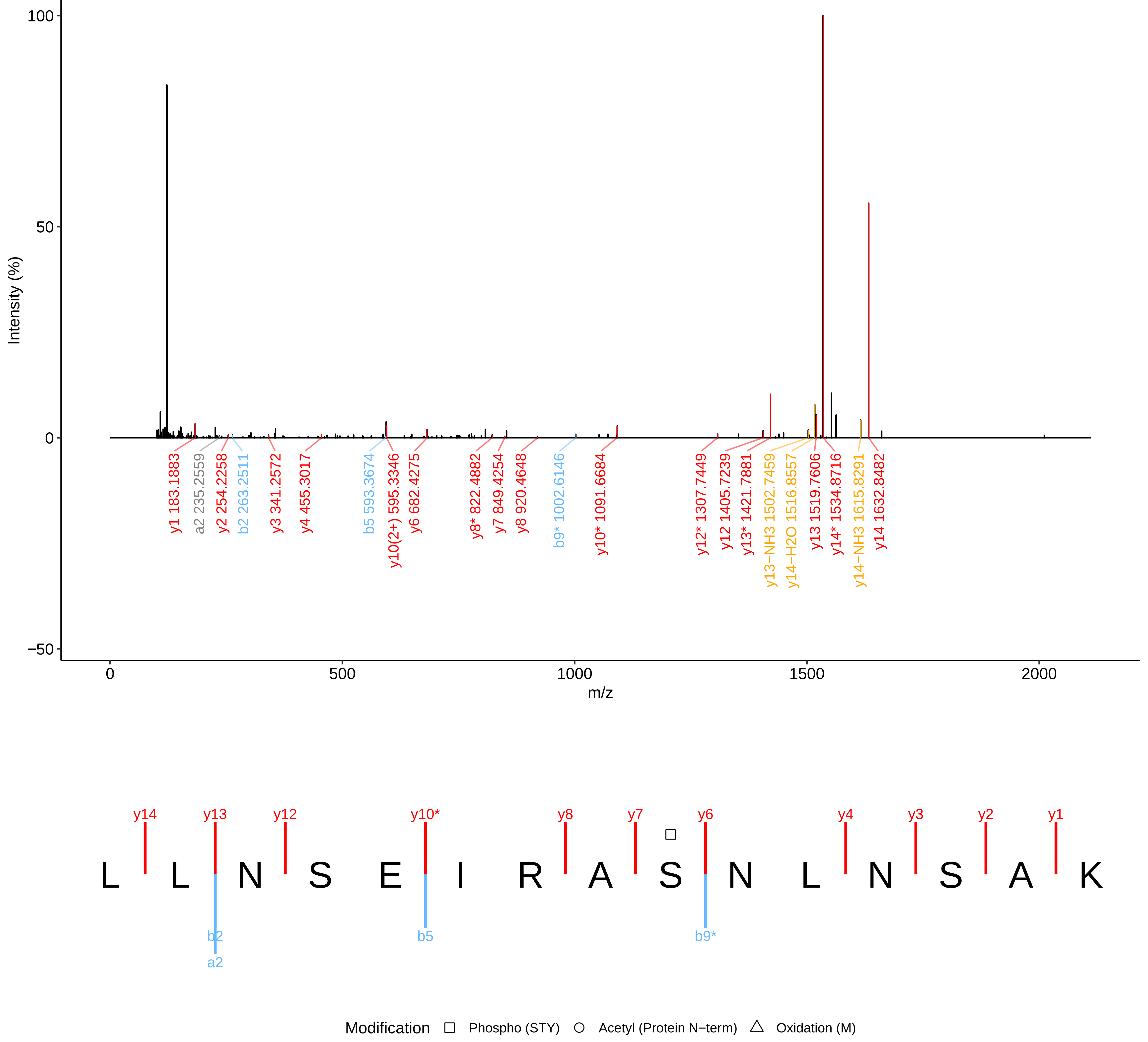

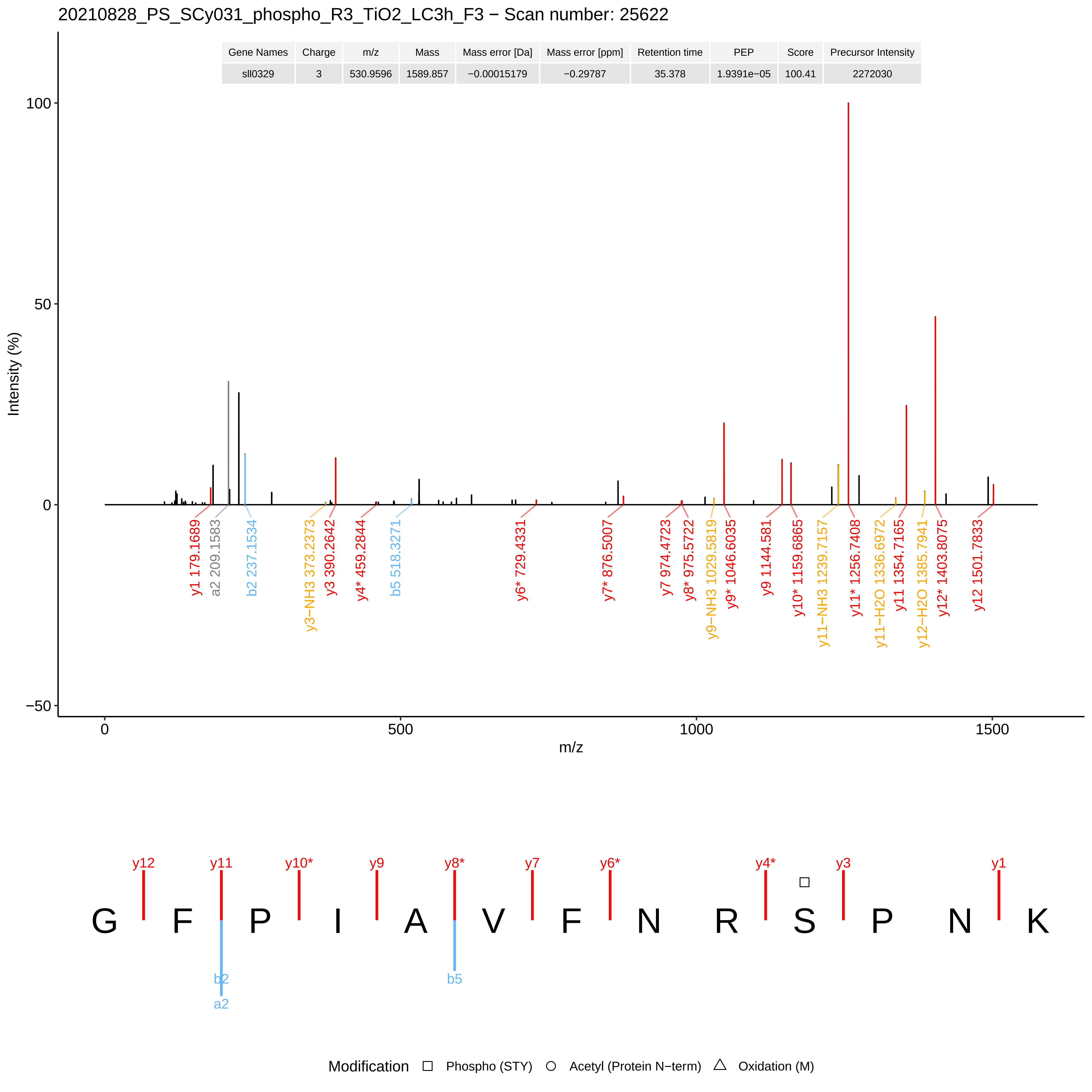

20210828_PS_SCy031_phospho_R3_TiO2_LC3h_F3 − Scan number: 25622
Gene Names | Charge | m/z | Mass | Mass error [Da] | Mass error [ppm] | Retention time | PEP | Score | Precursor Intensity
sll0329 | 3 | 530.9596 | 1589.857 | −0.00015179 | −0.29787 | 35.378 | 1.9391e−05 | 100.41 | 2272030
Intensity (%)
m/z
y1 179.1689
a2 209.1583
b2 237.1534
y3−NH3 373.2373
y3 390.2642
y4* 459.2844
b5 518.3271
y6* 729.4331
y7* 876.5007
y7 974.4723
y8* 975.5722
y9−NH3 1029.5819
y9* 1046.6035
y9 1144.581
y10* 1159.6865
y11−NH3 1239.7157
y11* 1256.7408
y11−H2O 1336.6972
y11 1354.7165
y12−H2O 1385.7941
y12* 1403.8075
y12 1501.7833
G F P I A V F N R S P N K
y12 y11 y10* y9 y8* y7 y6* y4* y3 y1
b2 a2 b5
Modification
Phospho (STY)
Acetyl (Protein N−term)
Oxidation (M)

20210828_PS_SCy031_phospho_R3_TiO2_LC3h_F1 − Scan number: 8270

Gene Names	Charge	m/z	Mass	Mass error [Da]	Mass error [ppm]	Retention time	PEP	Score	Precursor Intensity
sll0359	2	577.3256	1152.637	NA	NA	13.315	0.00062604	67.214	25628628

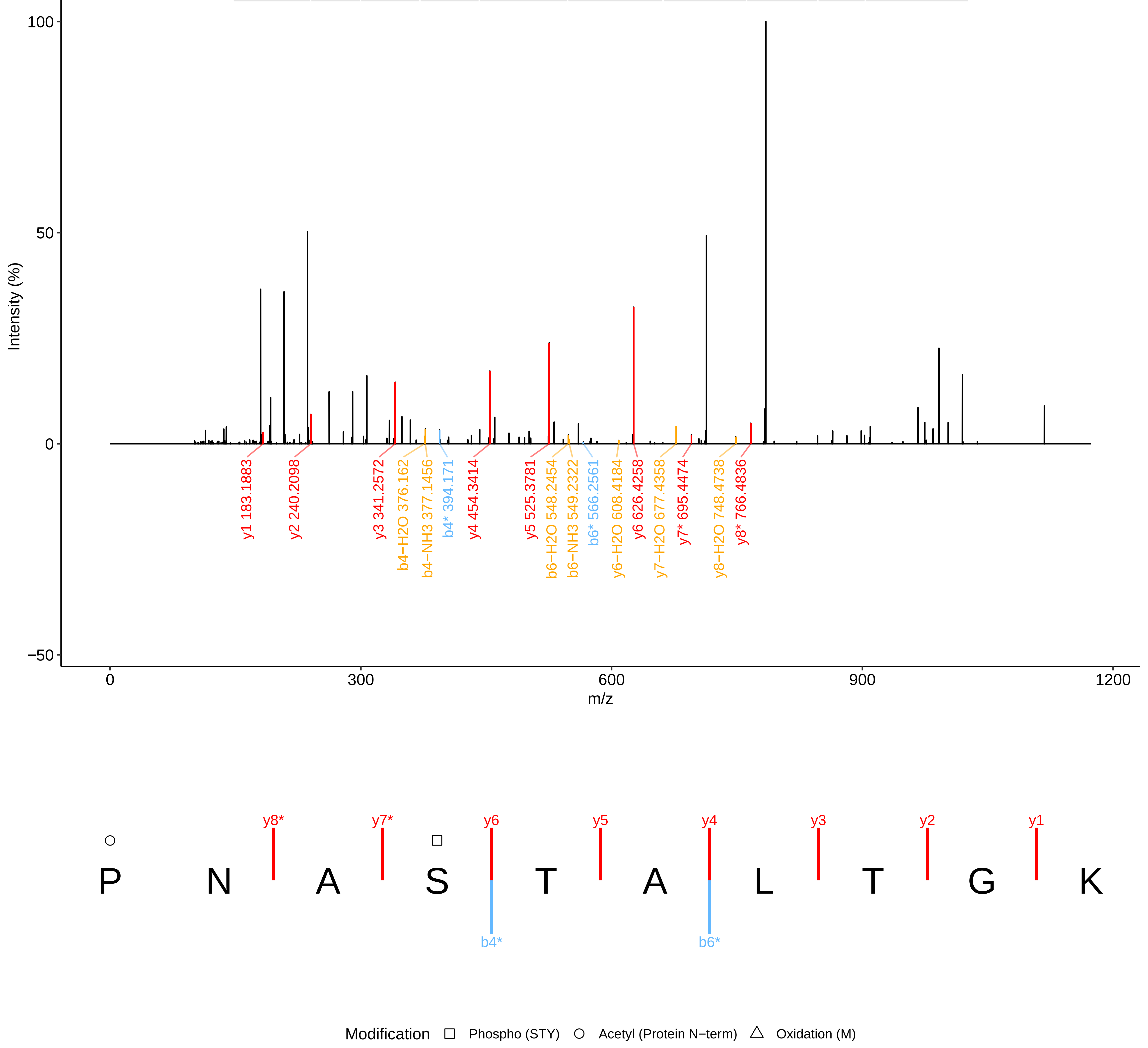

20211217_PS_SCy031_Proteome_R3_HC_F4 − Scan number: 21925

Gene Names	Charge	m/z	Mass	Mass error [Da]	Mass error [ppm]	Retention time	PEP	Score	Precursor Intensity
sll0497	2	528.7845	1055.554	0.00084779	1.6033	33.762	0.032161	75.311	9899942

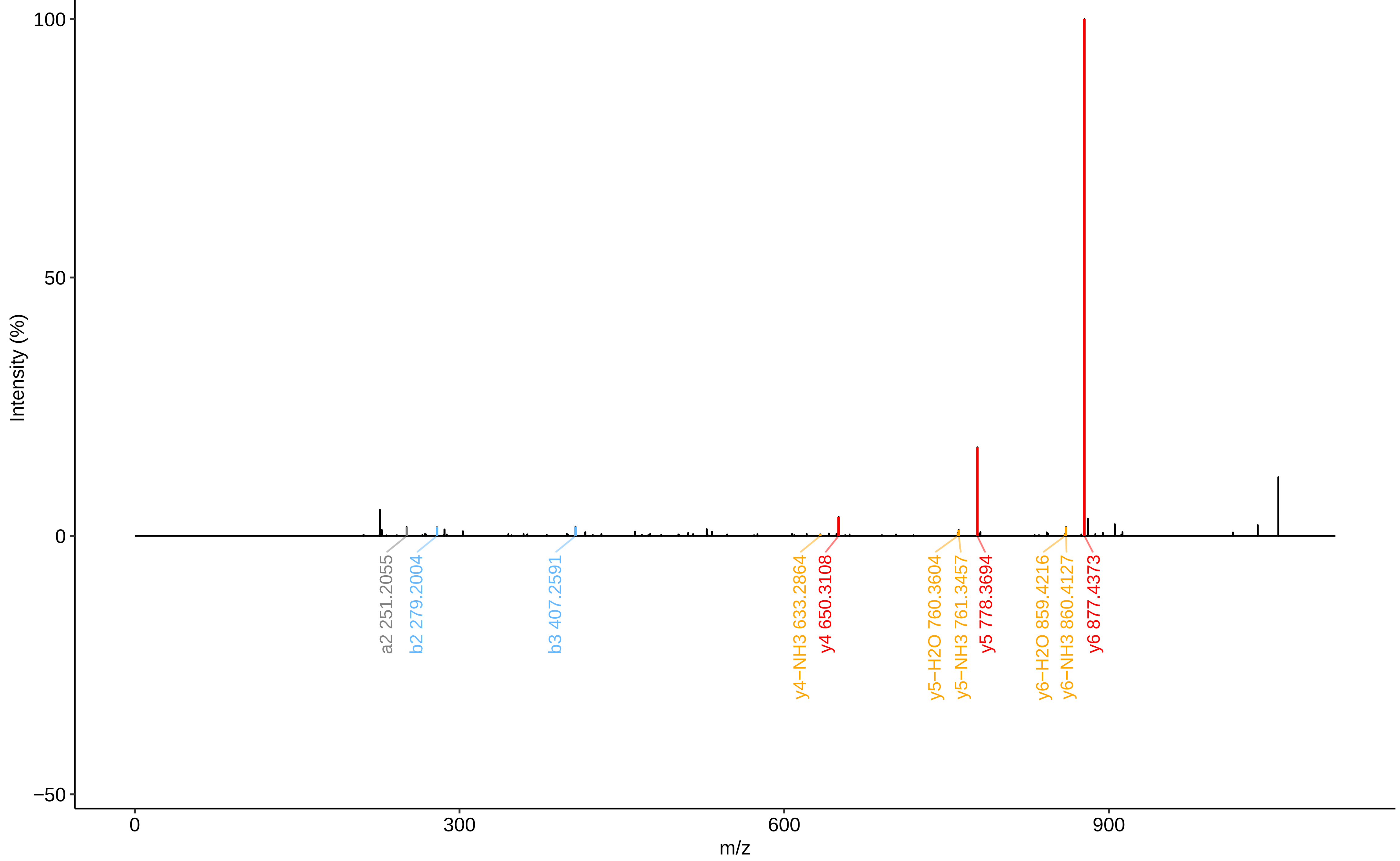

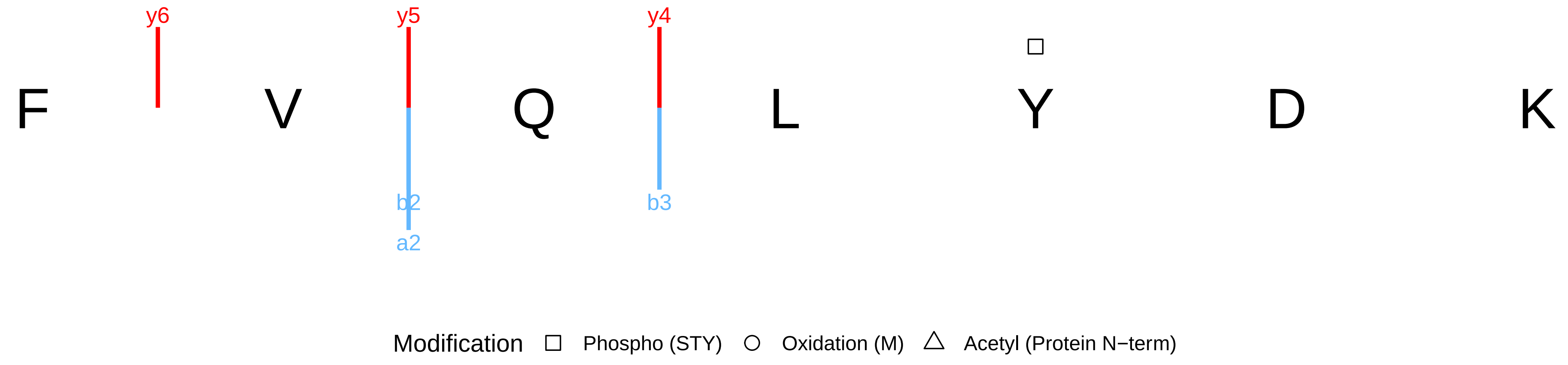

20210823_PS_SCy031_R1_phospho_LC24h_TiO2_3 − Scan number: 18234

Gene Names	Charge	m/z	Mass	Mass error [Da]	Mass error [ppm]	Retention time	PEP	Score	Precursor Intensity
sll0617	3	658.6491	1972.926	−0.00031554	−0.47906	25.906	1.0226e−38	204.94	6586889

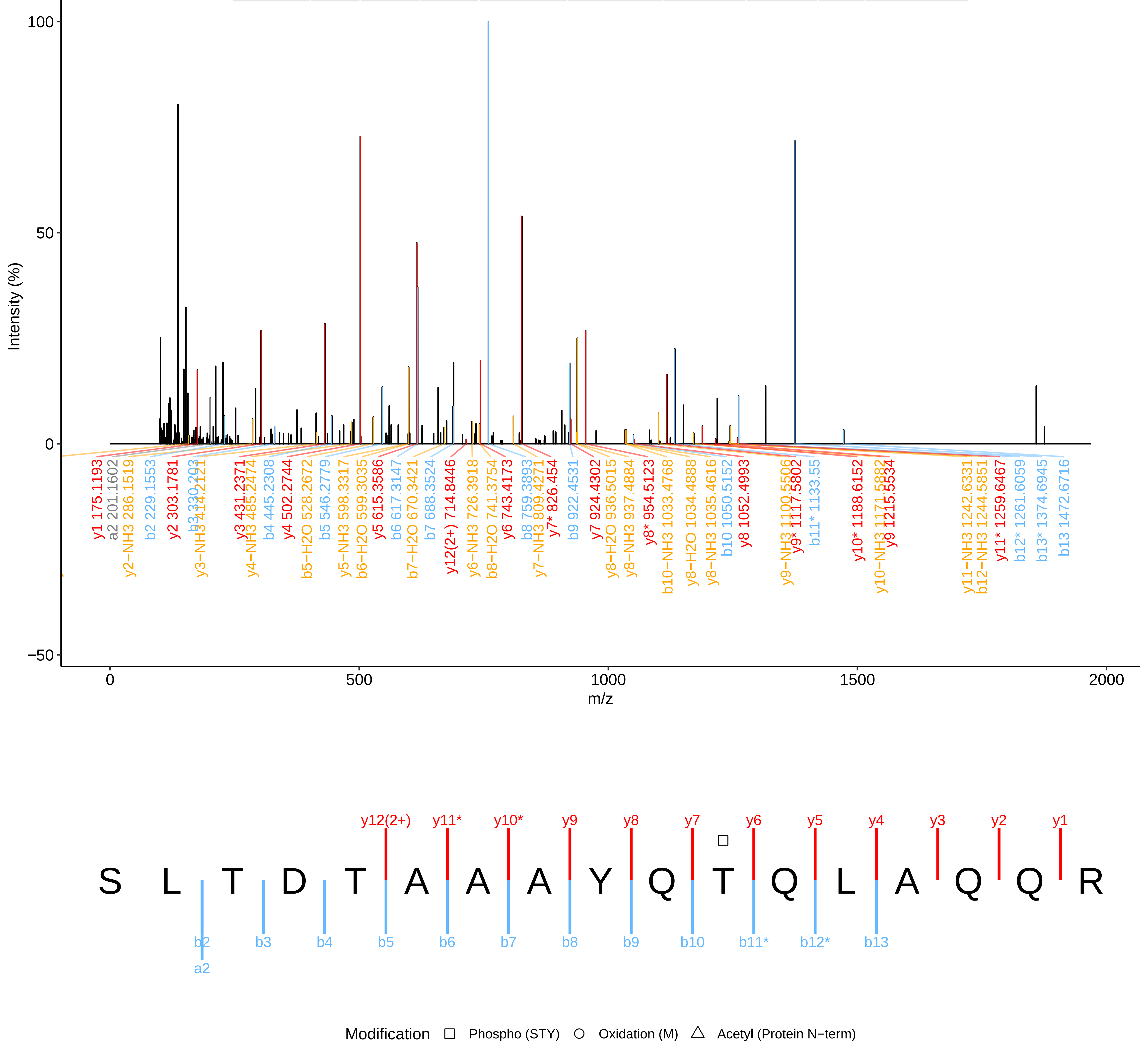

20210828_PS_SCy031_phospho_R3_TiO2_HC_F3 − Scan number: 29015

Gene Names	Charge	m/z	Mass	Mass error [Da]	Mass error [ppm]	Retention time	PEP	Score	Precursor Intensity
sll0617	2	1211.083	2420.152	NA	NA	39.656	4.9091e−15	132.36	NA

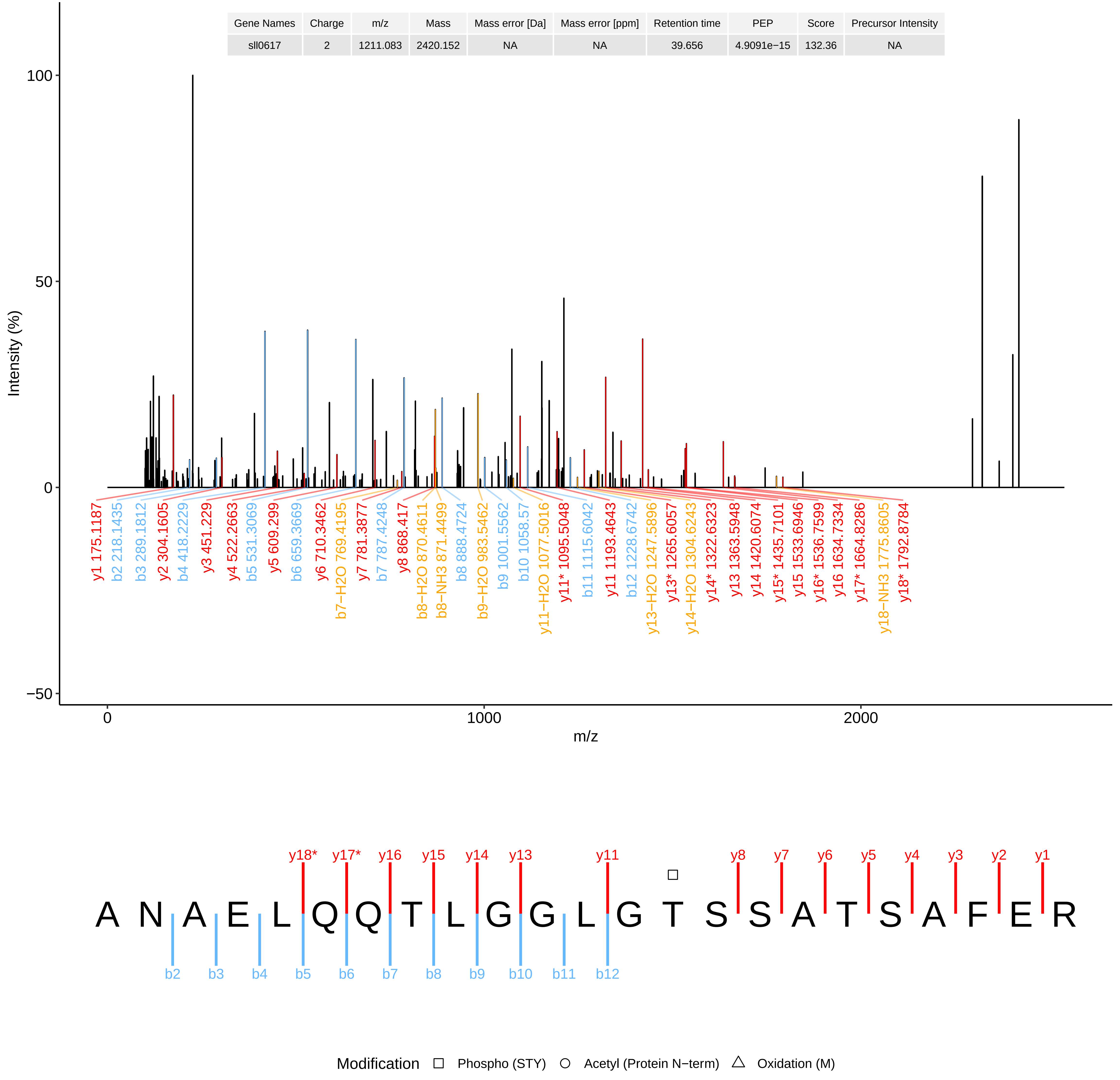

20210828_PS_SCy031_phospho_R3_TiO2_LC3h_F1 − Scan number: 28484

Gene Names	Charge	m/z	Mass	Mass error [Da]	Mass error [ppm]	Retention time	PEP	Score	Precursor Intensity
sll0617	2	1211.083	2420.152	0.00081223	0.67038	38.935	4.1805e−59	218.25	6977024

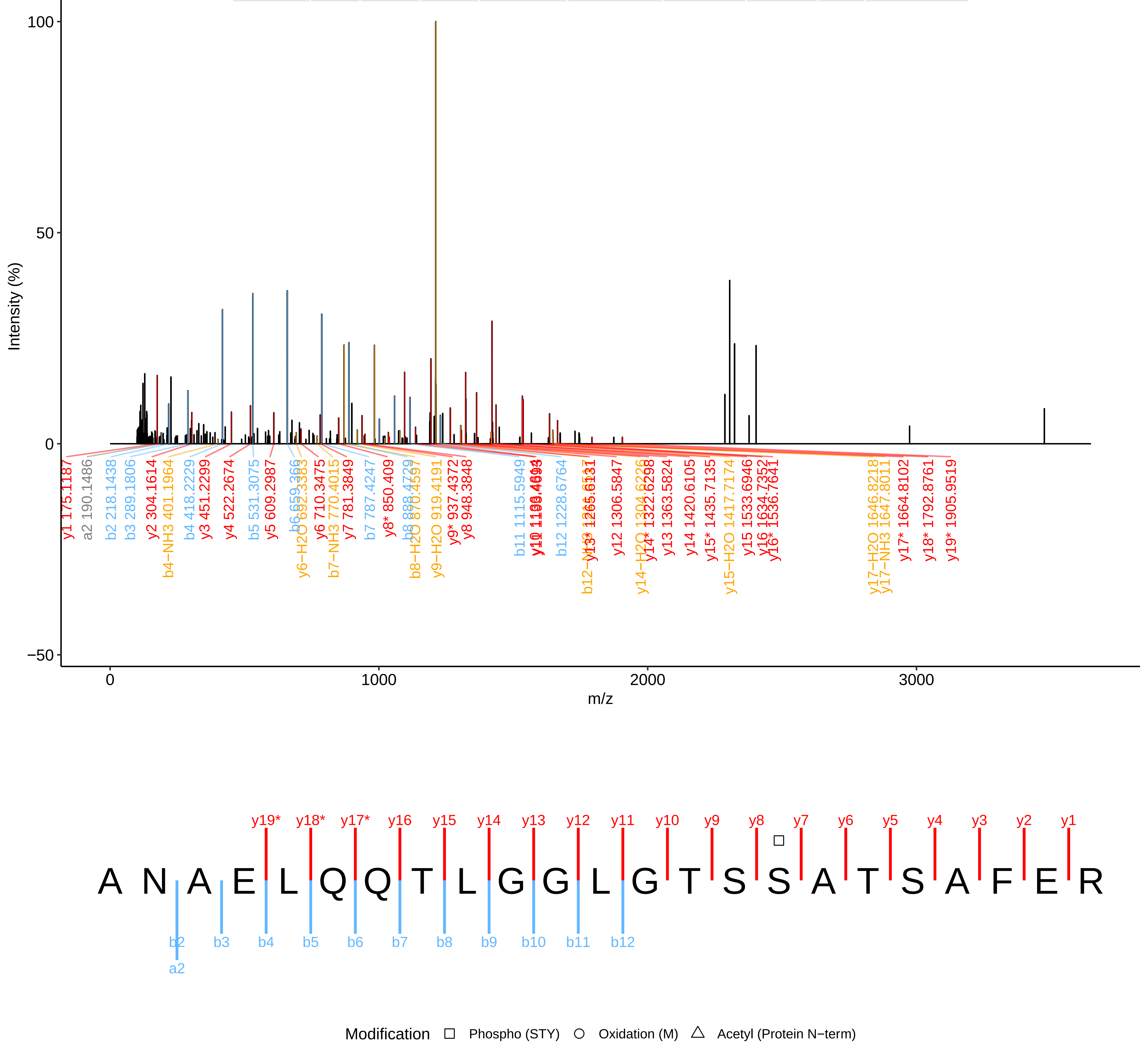

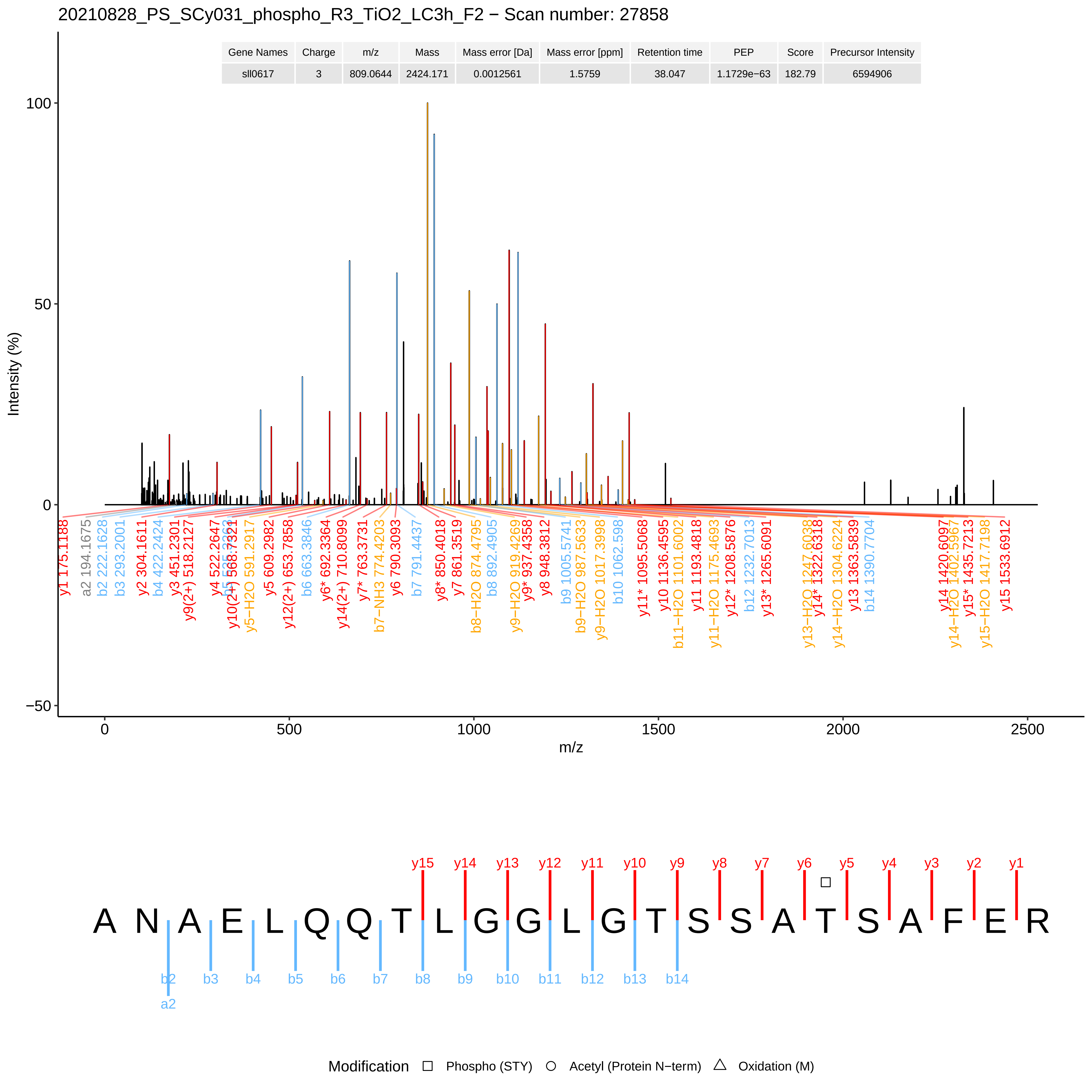
20210828_PS_SCy031_phospho_R3_TiO2_LC3h_F2 − Scan number: 27858
Gene Names | Charge | m/z | Mass | Mass error [Da] | Mass error [ppm] | Retention time | PEP | Score | Precursor Intensity
sll0617 | 3 | 809.0644 | 2424.171 | 0.0012561 | 1.5759 | 38.047 | 1.1729e−63 | 182.79 | 6594906
Intensity (%)
m/z
y1 175.1188
a2 194.1675
b2 222.1628
b3 293.2001
y2 304.1611
b4 422.2424
y3 451.2301
y9(2+) 518.2127
y4 522.2647
b5 535.3263
y10(2+) 568.7321
y5−H2O 591.2917
y5 609.2982
y12(2+) 653.7858
b6 663.3846
y6* 692.3364
y14(2+) 710.8099
y7* 763.3731
b7−NH3 774.4203
y6 790.3093
b7 791.4437
y8* 850.4018
y7 861.3519
b8−H2O 874.4795
b8 892.4905
y9−H2O 919.4269
y9* 937.4358
y8 948.3812
b9 1005.5741
b9−H2O 987.5633
y9−H2O 1017.3998
b10 1062.598
y11* 1095.5068
y10 1136.4595
b11−H2O 1101.6002
y11 1193.4818
y11−H2O 1175.4693
y12* 1208.5876
b12 1232.7013
y13* 1265.6091
y13−H2O 1247.6038
y14* 1322.6318
y14−H2O 1304.6224
y13 1363.5839
b14 1390.7704
y14 1420.6097
y14−H2O 1402.5967
y15* 1435.7213
y15−H2O 1417.7198
y15 1533.6912
A N A E L Q Q T L G G L G T S S A T S A F E R
y15 y14 y13 y12 y11 y10 y9 y8 y7 y6 y5 y4 y3 y2 y1
b2 b3 b4 b5 b6 b7 b8 b9 b10 b11 b12 b13 b14
a2
Modification □ Phospho (STY) ○ Acetyl (Protein N−term) △ Oxidation (M)

20210828_PS_SCy031_phospho_R2_TiO2_LC24h_F2 − Scan number: 28455

Gene Names	Charge	m/z	Mass	Mass error [Da]	Mass error [ppm]	Retention time	PEP	Score	Precursor Intensity
sll0617	2	1209.071	2416.127	NA	NA	38.788	1.7838e−36	187.09	NA

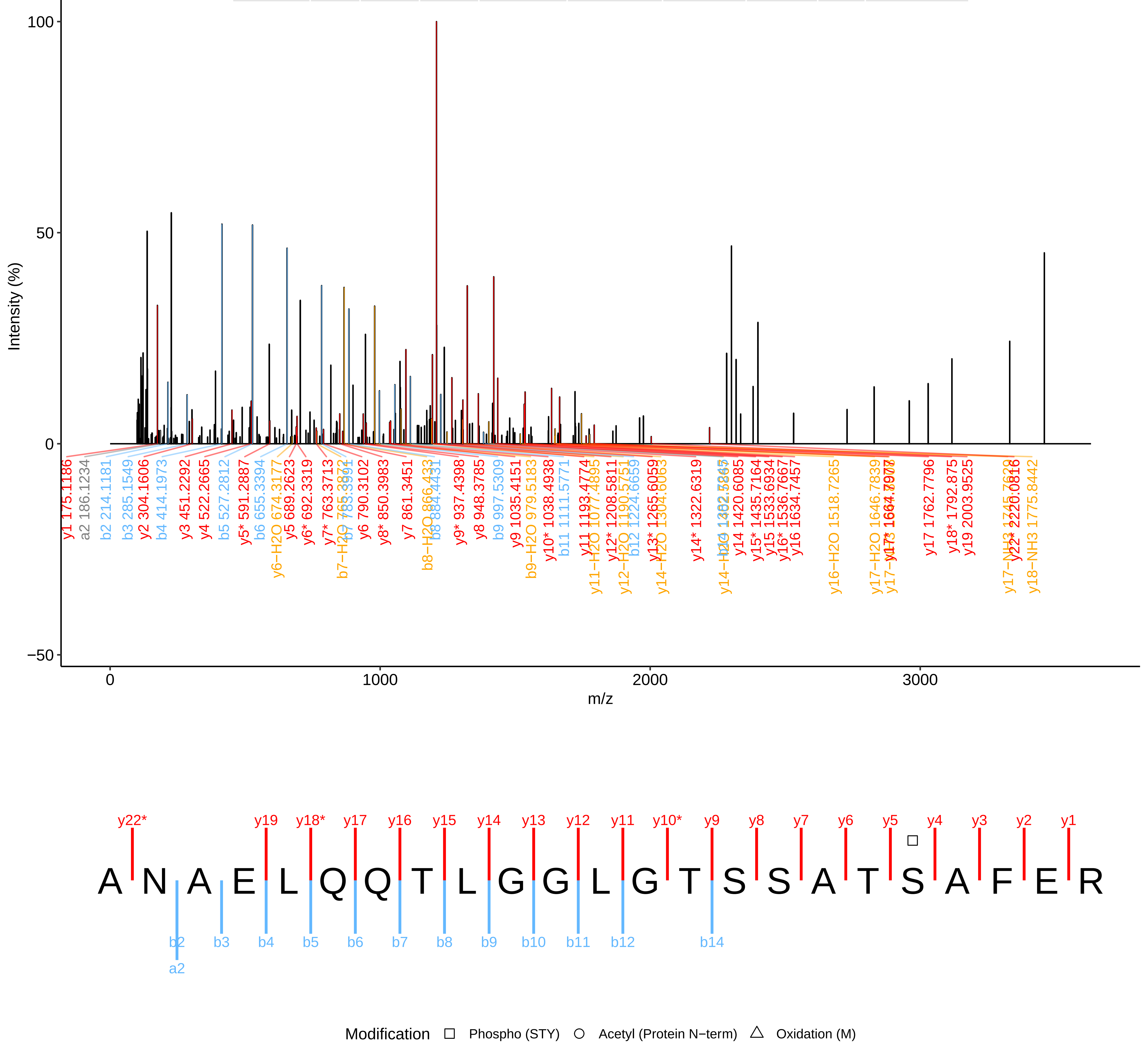

20210828_PS_SCy031_phospho_R3_TiO2_LC3h_F3 − Scan number: 23084

Gene Names	Charge	m/z	Mass	Mass error [Da]	Mass error [ppm]	Retention time	PEP	Score	Precursor Intensity
sll0726	3	761.0312	2280.072	NA	NA	32.152	6.1804e−05	79.36	NA

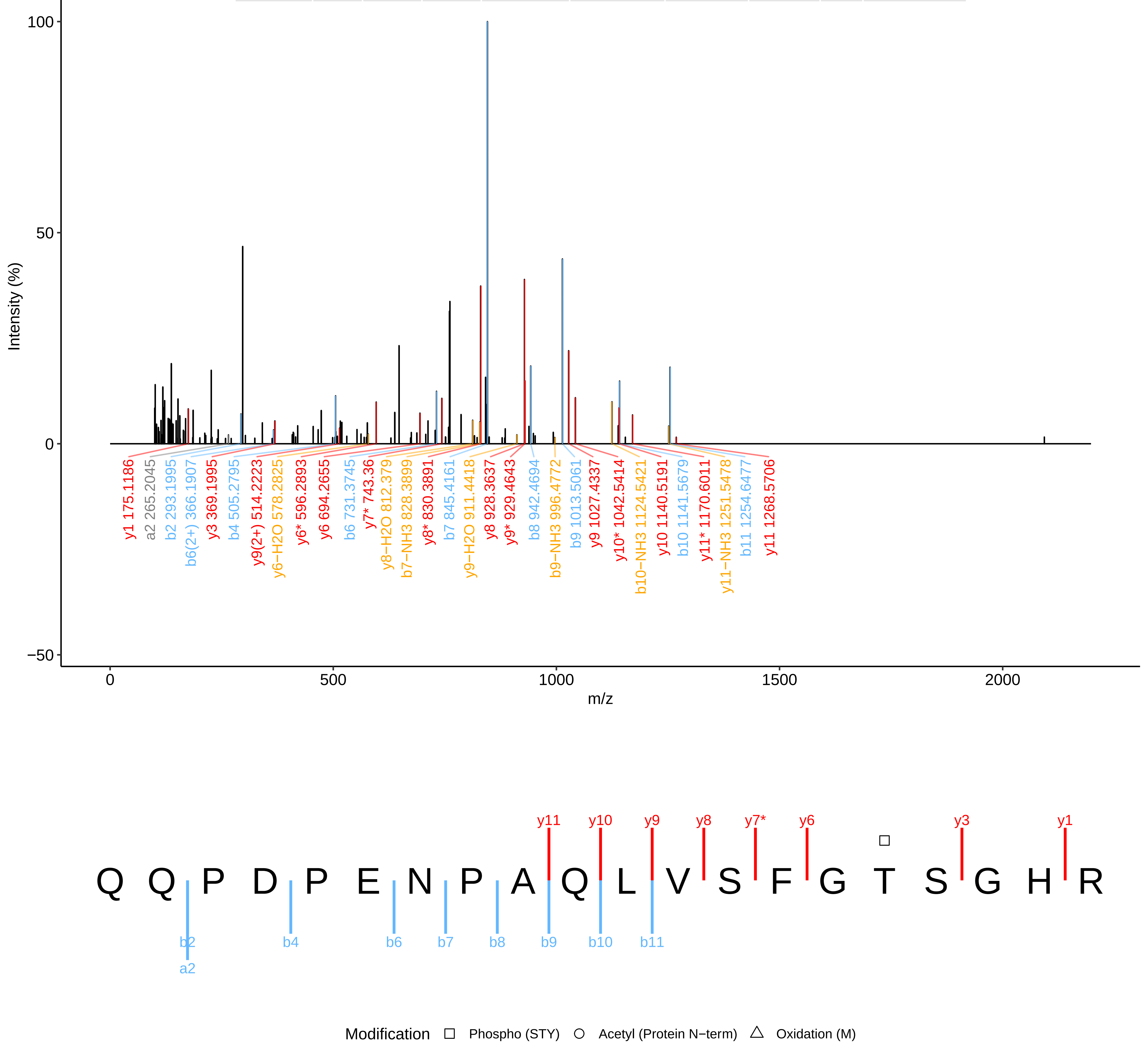

20210823_PS_SCy031_R1_phospho_LC3h_TiO2_2 − Scan number: 26194

Gene Names	Charge	m/z	Mass	Mass error [Da]	Mass error [ppm]	Retention time	PEP	Score	Precursor Intensity
sll0726	2	1194.625	2387.235	0.00032326	0.27902	35.919	0	440.76	24747018

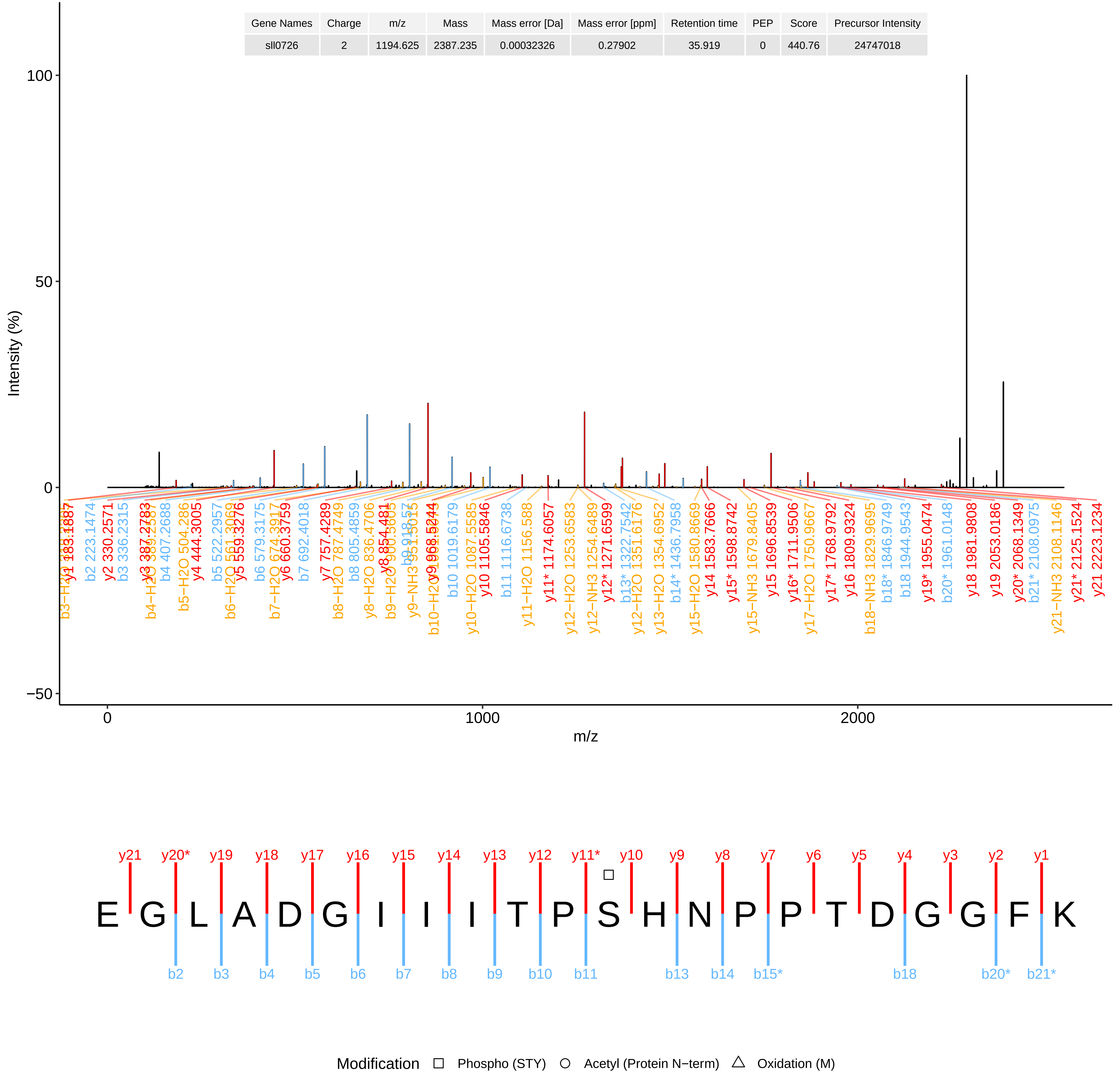

20210828_PS_SCy031_phospho_R3_TiO2_LC3h_F3 − Scan number: 19490

Gene Names	Charge	m/z	Mass	Mass error [Da]	Mass error [ppm]	Retention time	PEP	Score	Precursor Intensity
sll0851	2	680.3023	1358.59	0.00045536	0.66935	27.608	0.001738	62.732	2295399

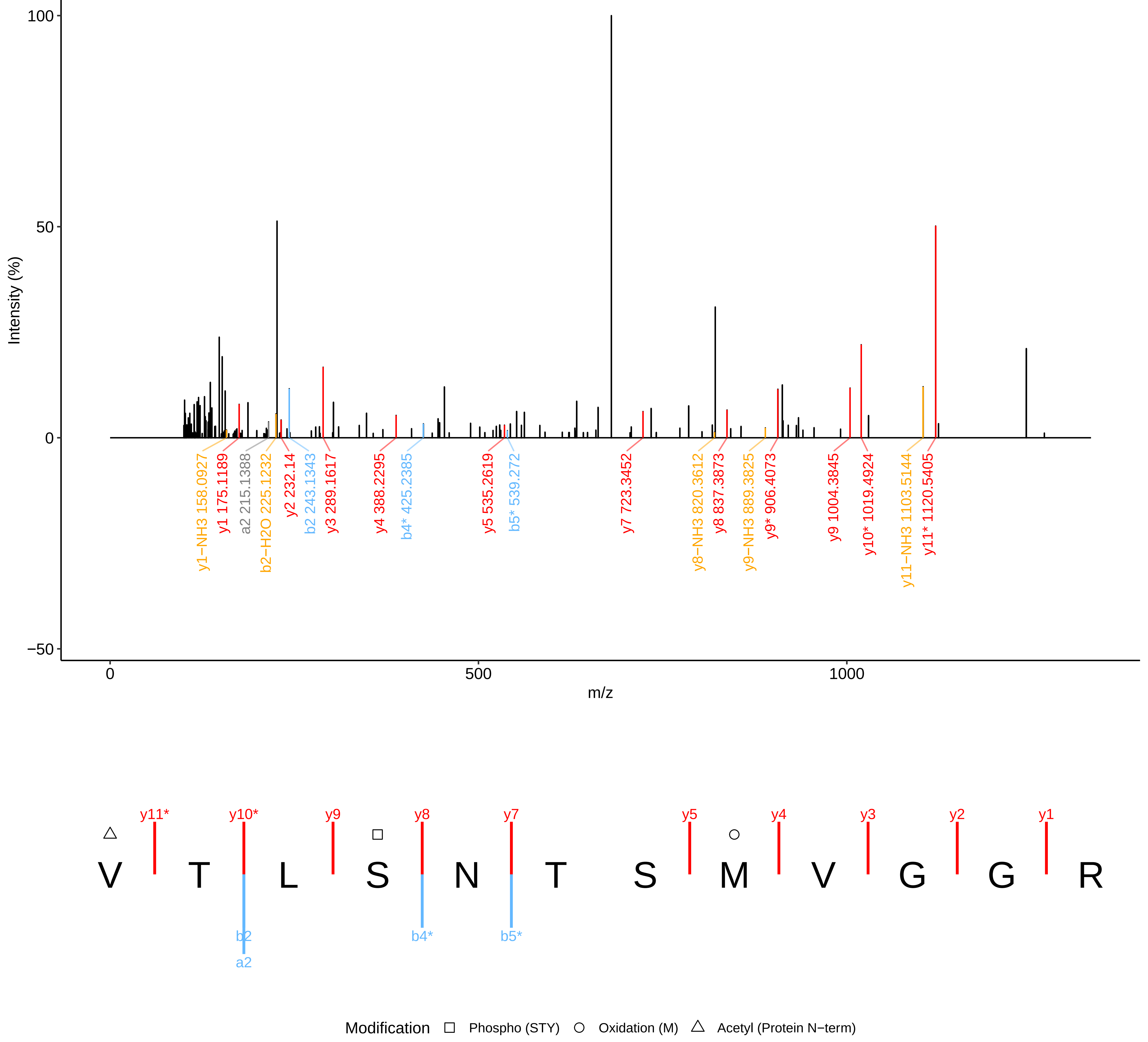

20210828_PS_SCy031_phospho_R3_TiO2_LC3h_F3 − Scan number: 17816

Gene Names	Charge	m/z	Mass	Mass error [Da]	Mass error [ppm]	Retention time	PEP	Score	Precursor Intensity
sll0877	2	713.3297	1424.645	0.001098	1.5701	25.497	0.00025736	121.24	5063640

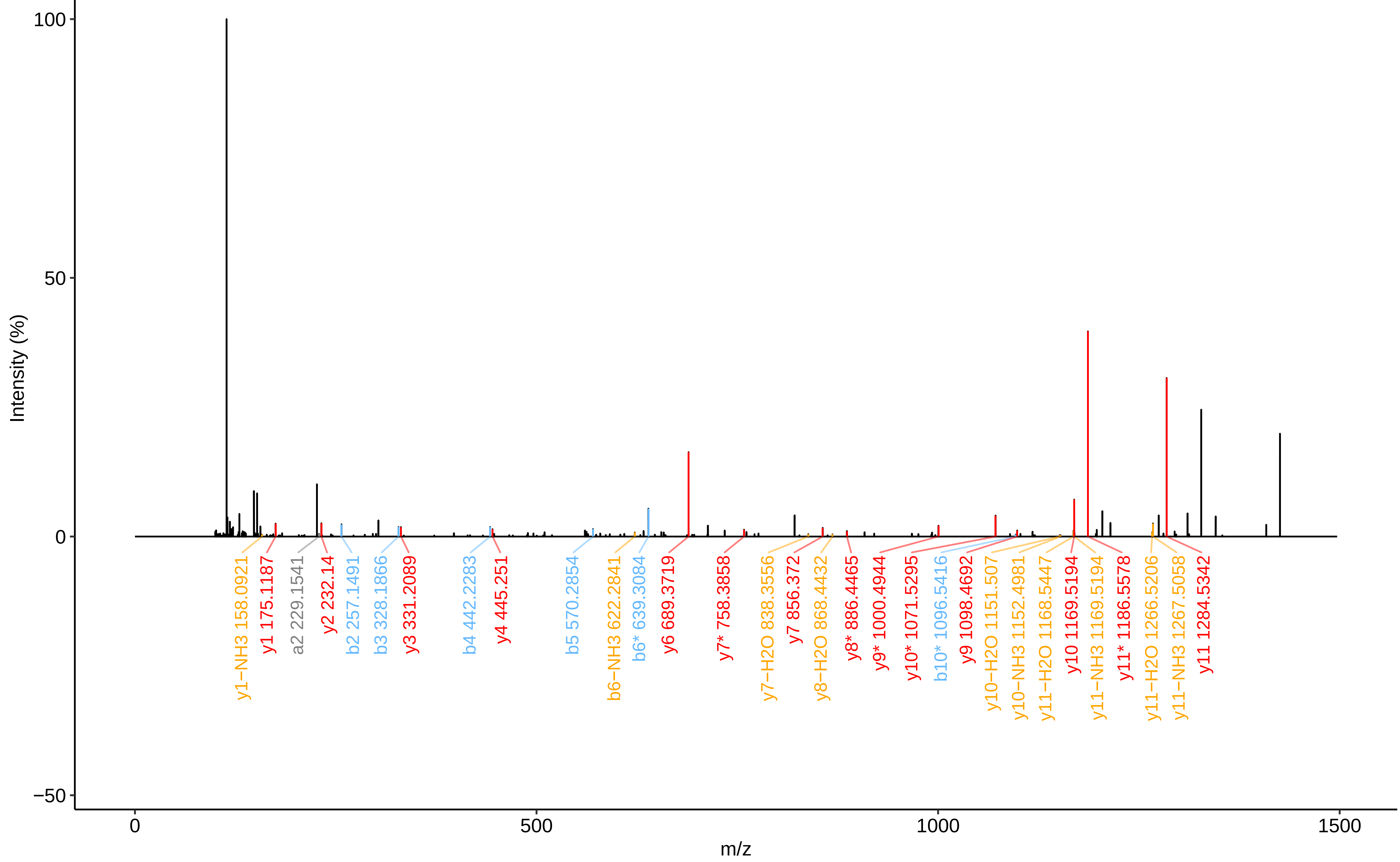

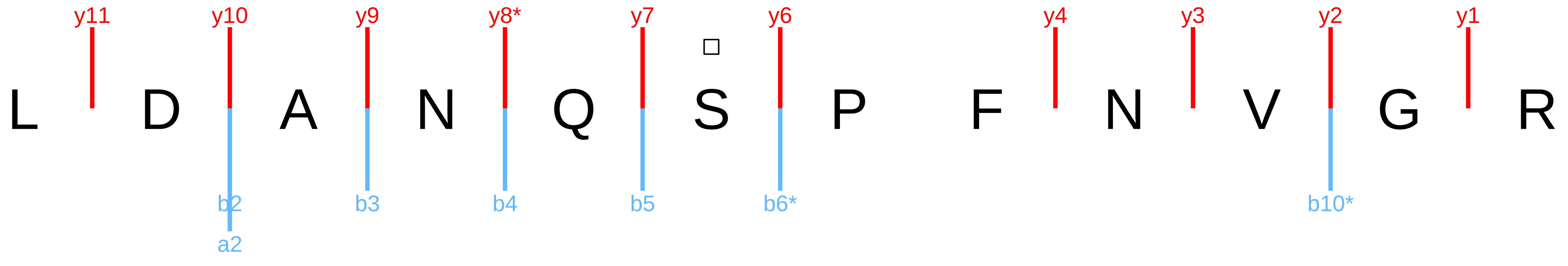

Modification □ Phospho (STY) ○ Acetyl (Protein N−term) △ Oxidation (M)

20210823_PS_SCy031_R1_phospho_LC24h_TiO2_3 − Scan number: 14734

Gene Names	Charge	m/z	Mass	Mass error [Da]	Mass error [ppm]	Retention time	PEP	Score	Precursor Intensity
sll0923	3	647.6181	1939.832	−0.00050079	−0.78625	21.491	5.0422e−19	138.68	5975779

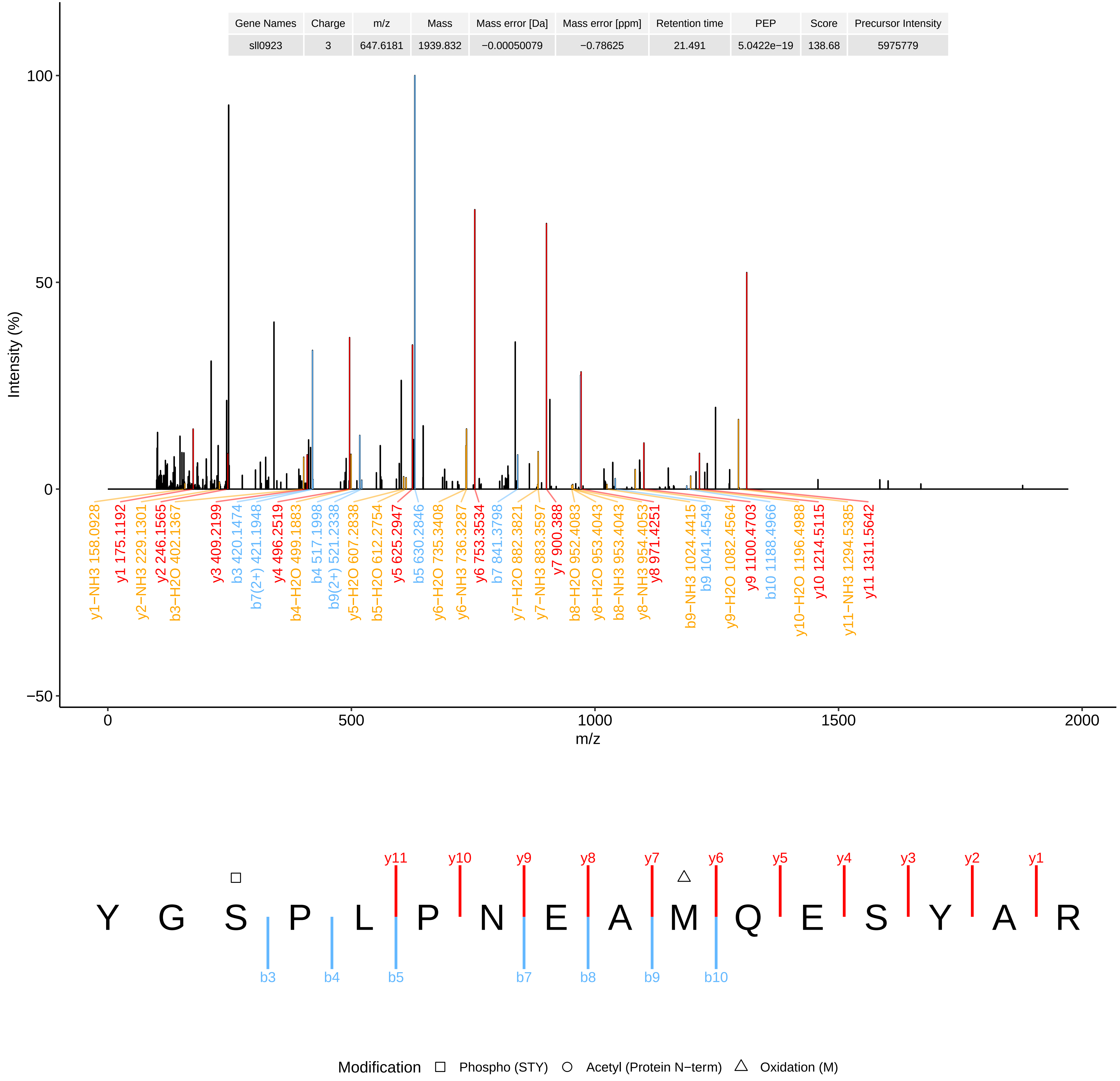

20210828_PS_SCy031_phospho_R3_TiO2_HC_F1 − Scan number: 6304

Gene Names	Charge	m/z	Mass	Mass error [Da]	Mass error [ppm]	Retention time	PEP	Score	Precursor Intensity
sll0923	2	761.8009	1521.587	1.8949e−05	0.024874	10.803	0.00028932	101.46	9157042

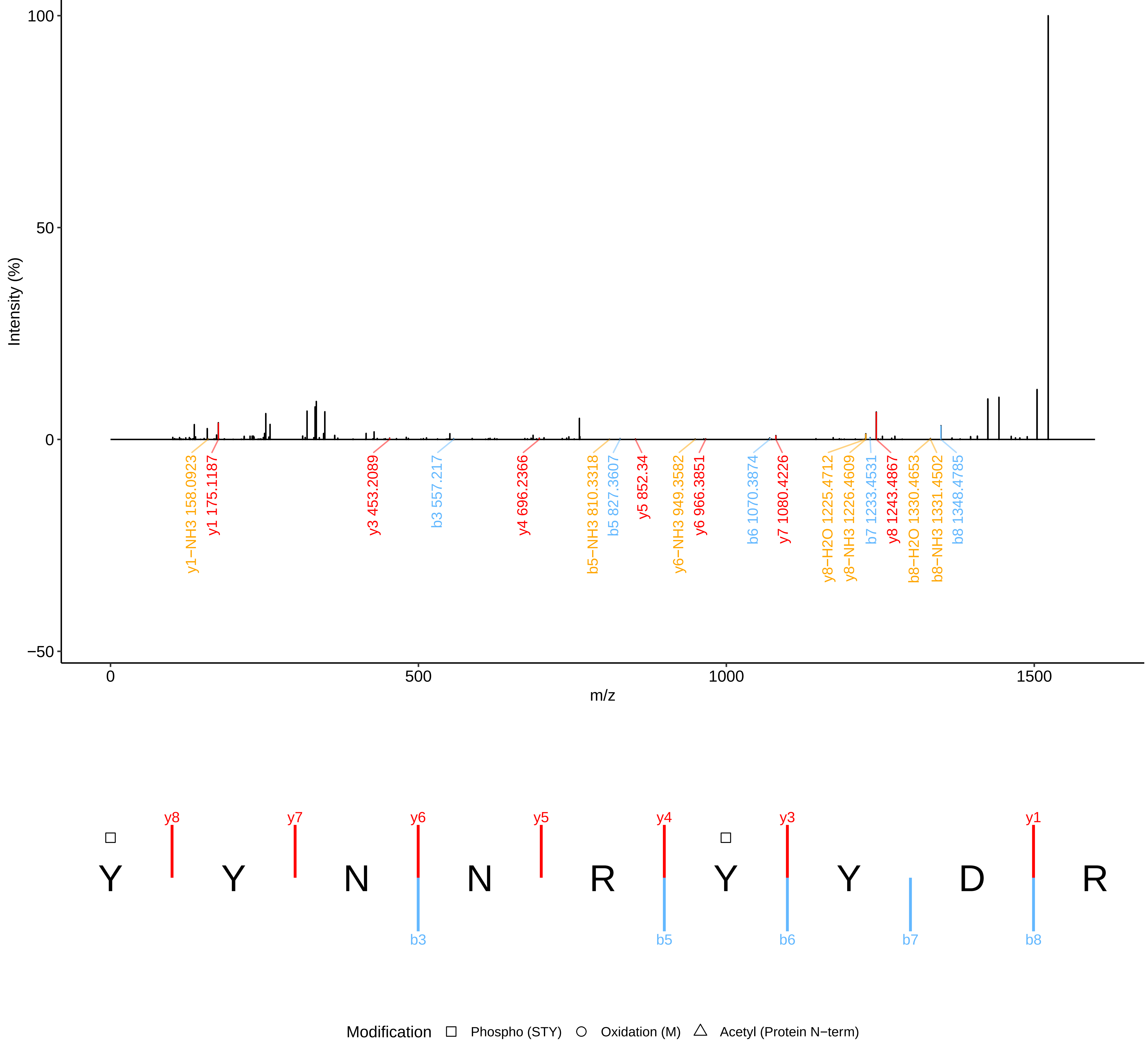

20210828_PS_SCy031_phospho_R2_TiO2_HC_F1 − Scan number: 3961

Gene Names	Charge	m/z	Mass	Mass error [Da]	Mass error [ppm]	Retention time	PEP	Score	Precursor Intensity
sll0923	3	605.6128	1813.816	4.3255e−05	0.074378	7.8007	0.00011238	71.153	16790214

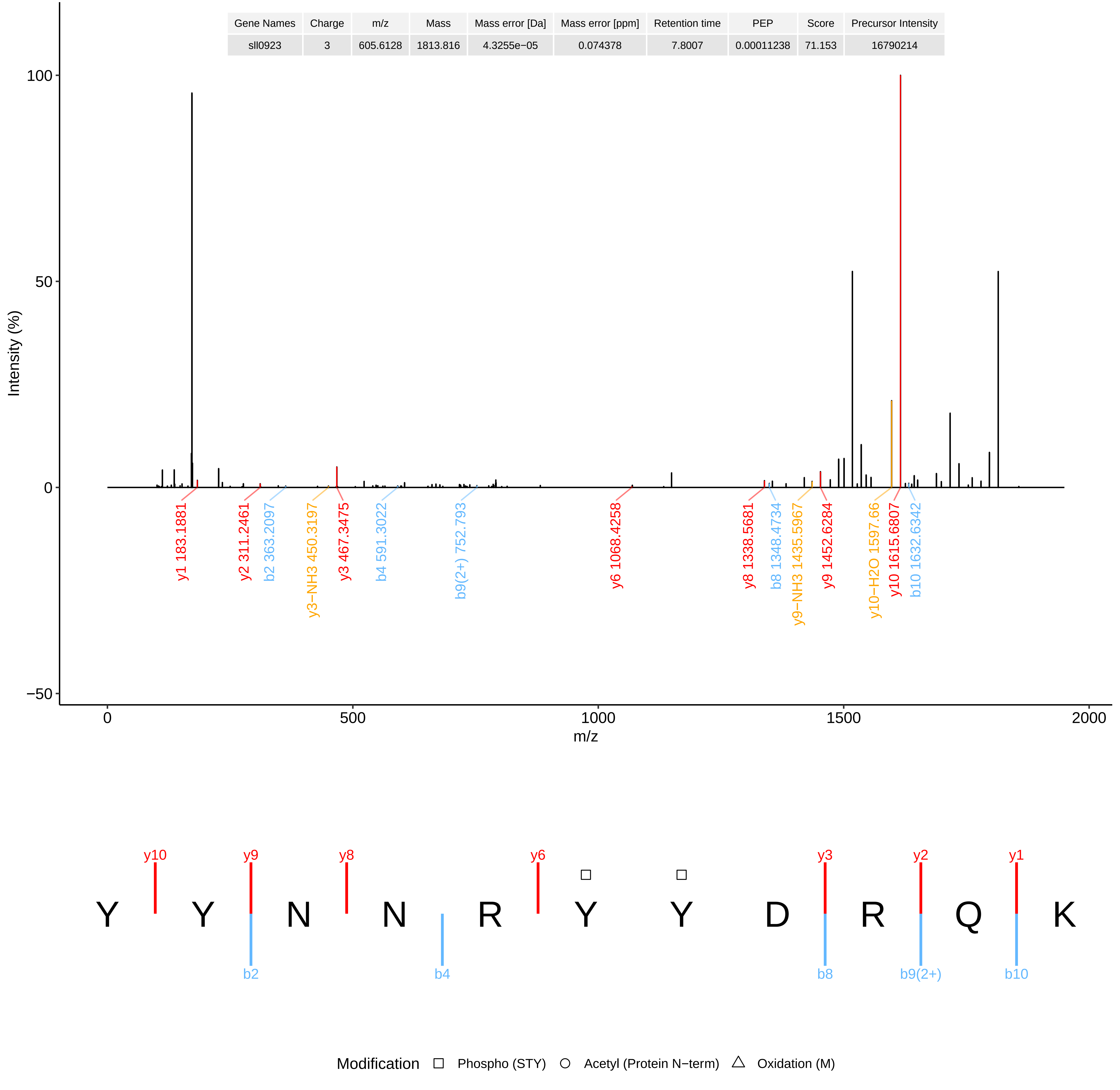

20210823_PS_SCy031_R1_phospho_LC3h_TiO2_1 − Scan number: 23443

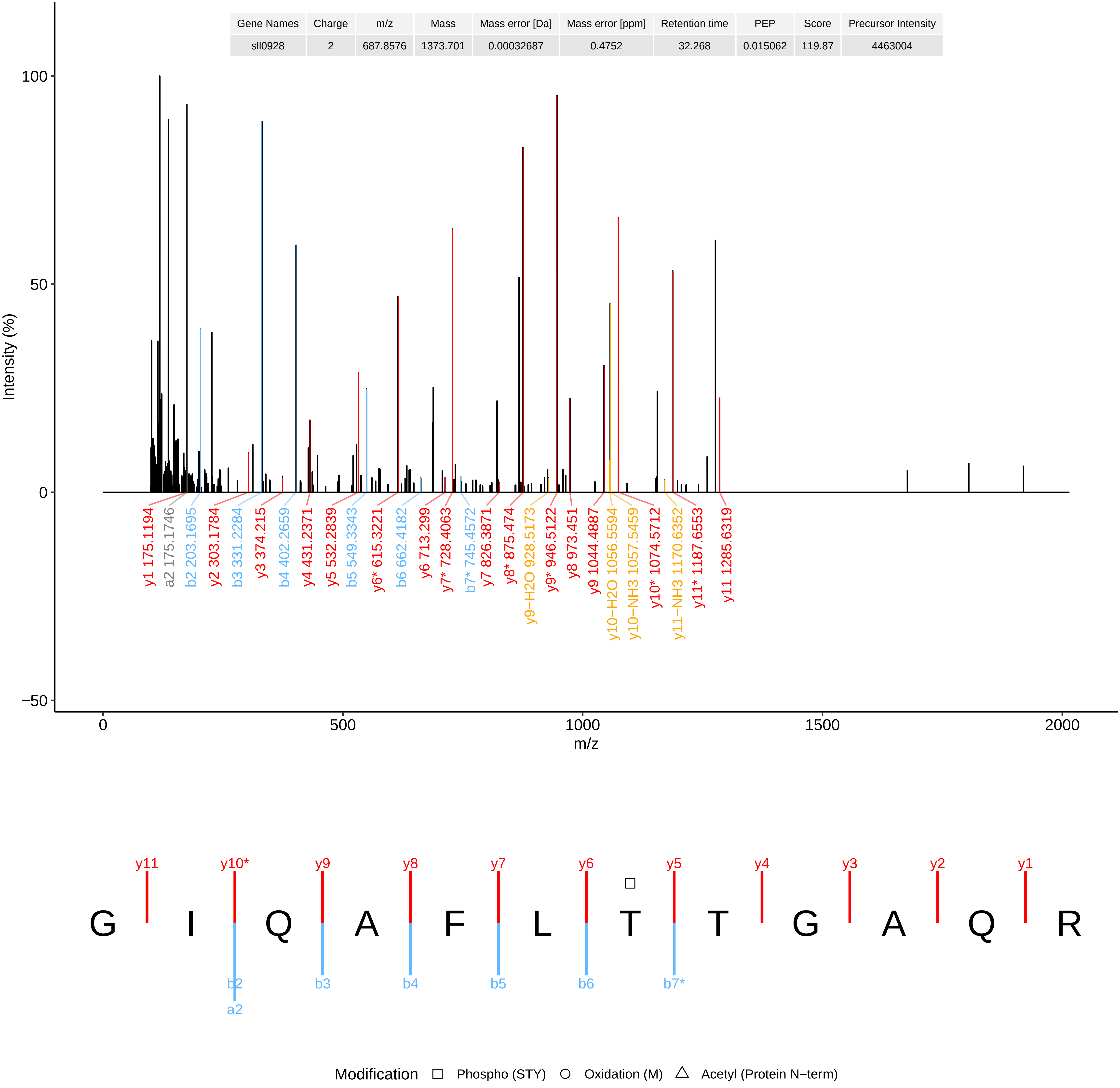

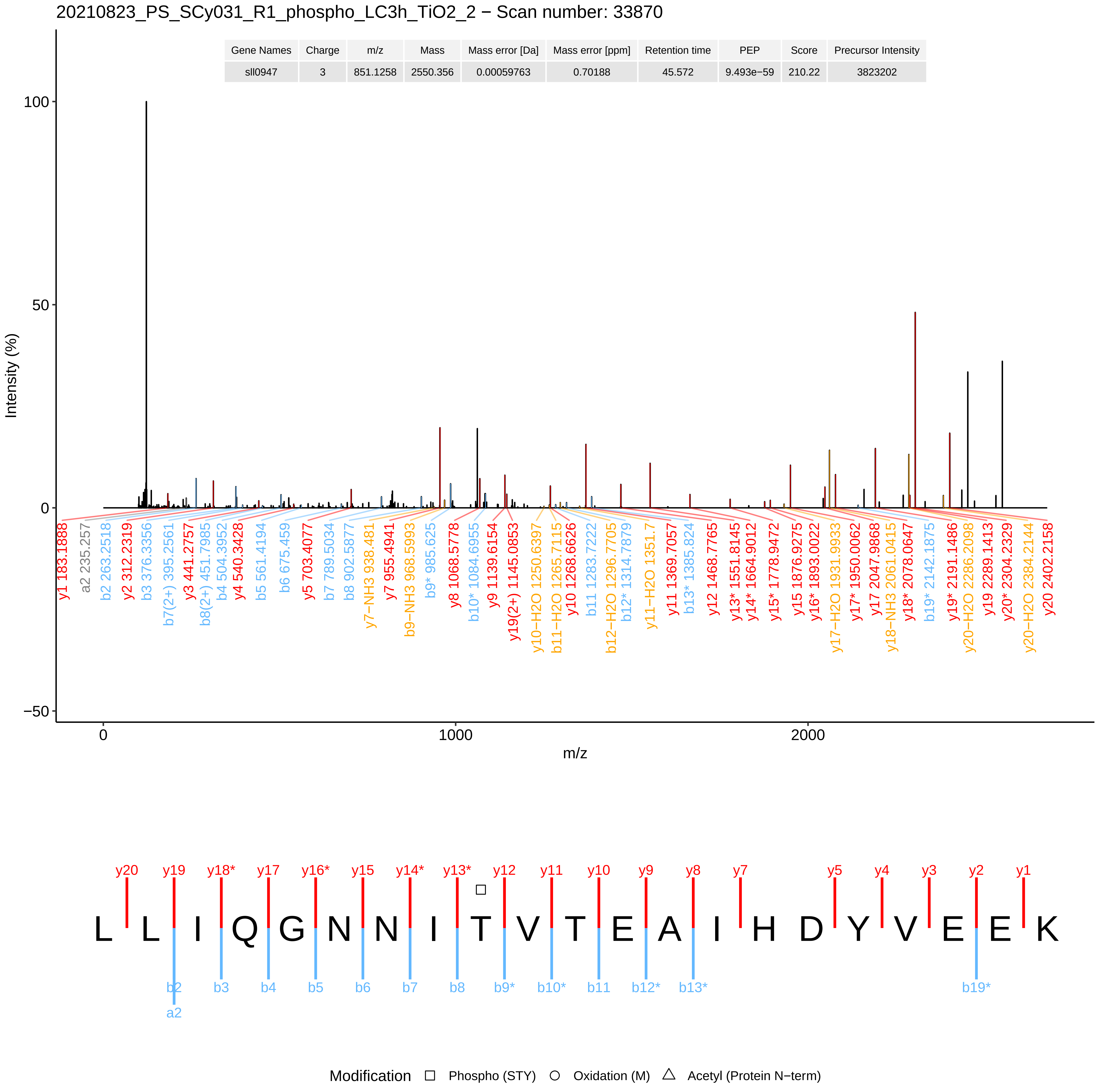
20210823_PS_SCy031_R1_phospho_LC3h_TiO2_2 − Scan number: 33870
Gene Names | Charge | m/z | Mass | Mass error [Da] | Mass error [ppm] | Retention time | PEP | Score | Precursor Intensity
sll0947 | 3 | 851.1258 | 2550.356 | 0.00059763 | 0.70188 | 45.572 | 9.493e−59 | 210.22 | 3823202
Intensity (%)
m/z
y1 183.1888
a2 235.257
b2 263.2518
y2 312.2319
b3 376.3356
b7(2+) 395.2561
y3 441.2757
b8(2+) 451.7985
b4 504.3952
y4 540.3428
b5 561.4194
b6 675.459
y5 703.4077
b7 789.5034
b8 902.5877
y7−NH3 938.481
y7 955.4941
b9−NH3 968.5993
b9* 985.625
y8 1068.5778
b10* 1084.6955
y9 1139.6154
y19(2+) 1145.0853
y10−H2O 1250.6397
b11−H2O 1265.7115
y10 1268.6626
b11 1283.7222
b12−H2O 1296.7705
b12* 1314.7879
y11−H2O 1351.7
y11 1369.7057
b13* 1385.824
y12 1468.7765
y13* 1551.8145
y14* 1664.9012
y15* 1778.9472
y15 1876.9275
y16* 1893.0022
y17−H2O 1931.9933
y17* 1950.0062
y17 2047.9868
y18−NH3 2061.0415
y18* 2078.0647
b19* 2142.1875
y19* 2191.1486
y20−H2O 2286.2098
y19 2289.1413
y20* 2304.2329
y20−H2O 2384.2144
y20 2402.2158
L L I Q G N N I T V T E A I H D Y V E E K
Modification □ Phospho (STY) ○ Oxidation (M) △ Acetyl (Protein N−term)

20210828_PS_SCy031_phospho_R3_TiO2_LC3h_F2 − Scan number: 8721

Gene Names	Charge	m/z	Mass	Mass error [Da]	Mass error [ppm]	Retention time	PEP	Score	Precursor Intensity
sll0947	2	788.8639	1575.713	3.4572e−06	0.0044734	13.947	2.8966e−94	254.63	3590592

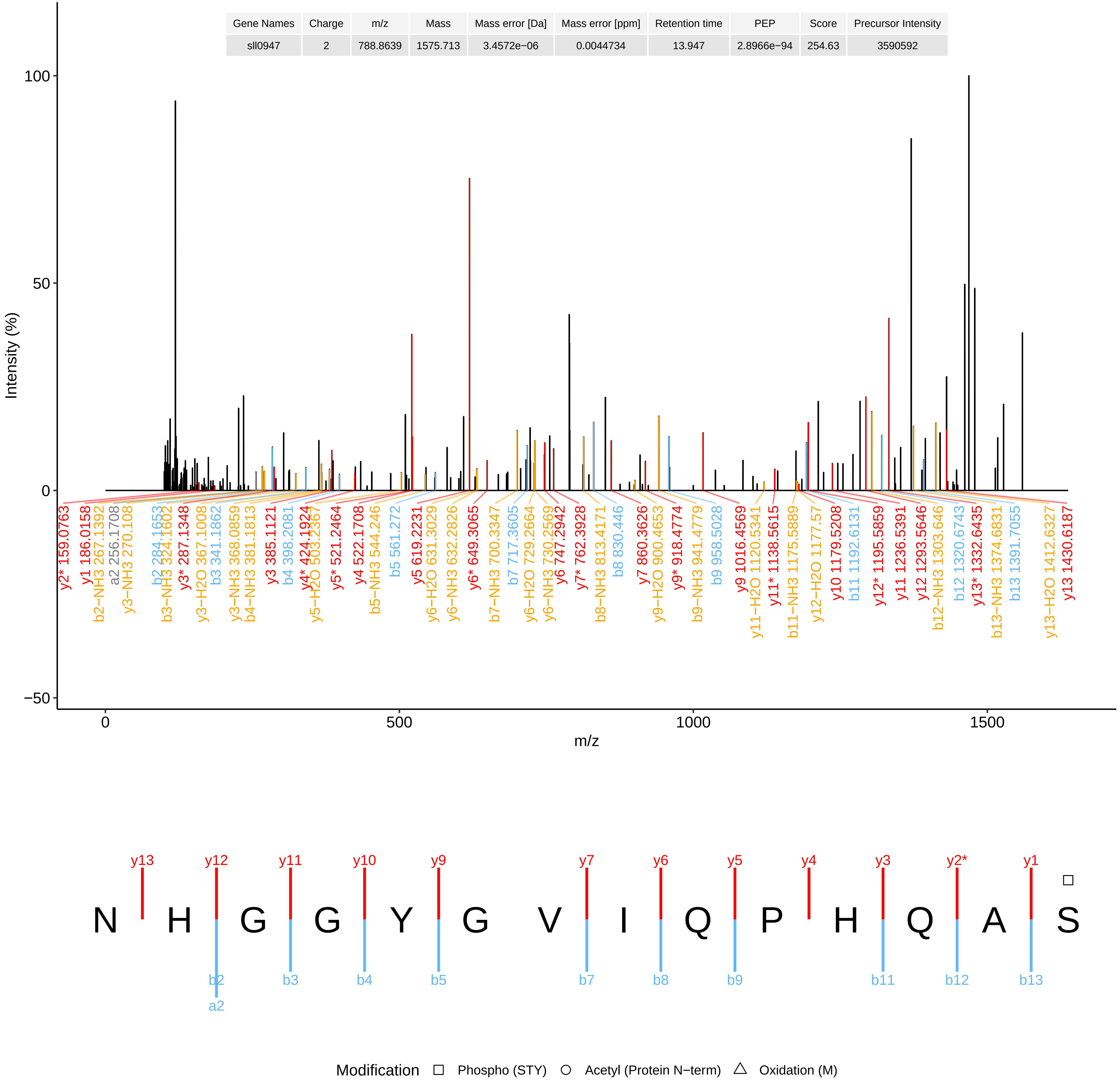

20210828_PS_SCy031_phospho_R2_TiO2_LC24h_F3 − Scan number: 27122

Gene Names	Charge	m/z	Mass	Mass error [Da]	Mass error [ppm]	Retention time	PEP	Score	Precursor Intensity
sll0982	2	880.927	1759.839	3.5292e−05	0.040063	37.2	9.9437e−09	147.17	3056700

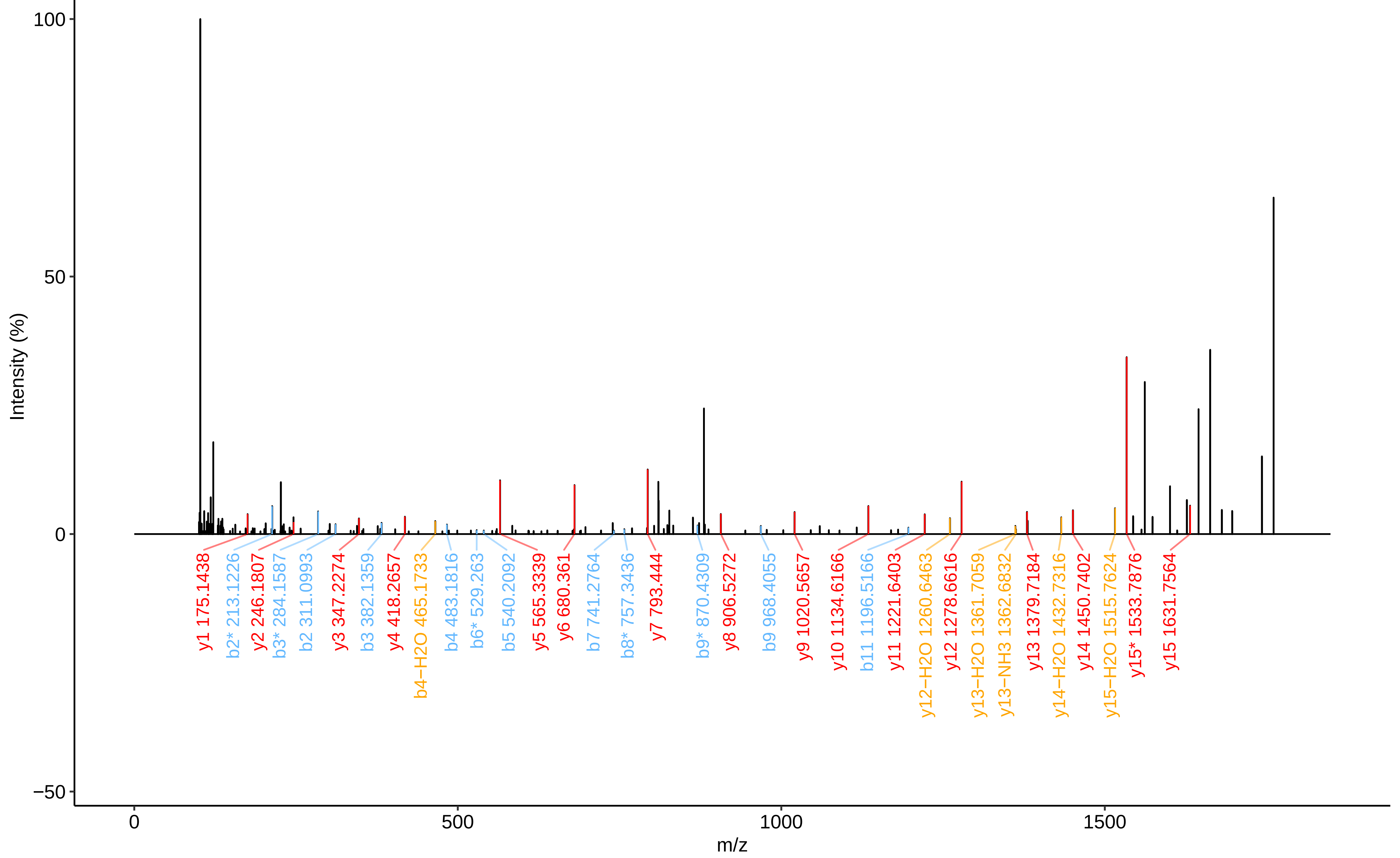

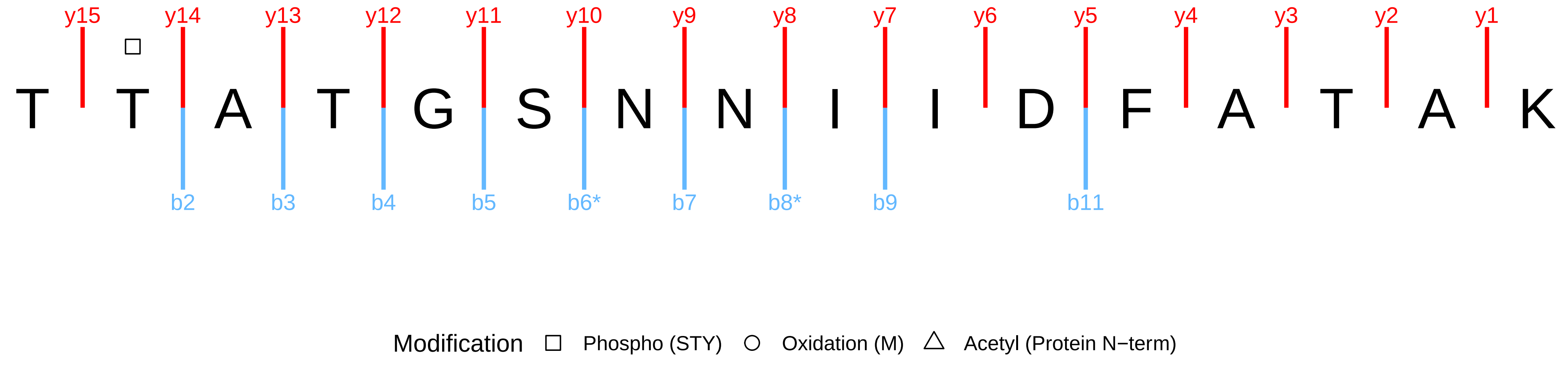

20211217_PS_SCy031_Proteome_R2_24hLC_F6 − Scan number: 20203

Gene Names	Charge	m/z	Mass	Mass error [Da]	Mass error [ppm]	Retention time	PEP	Score	Precursor Intensity
sll1020	2	1049.06	2096.106	−0.0014359	−1.3681	33.966	0.0084395	67.334	4061884

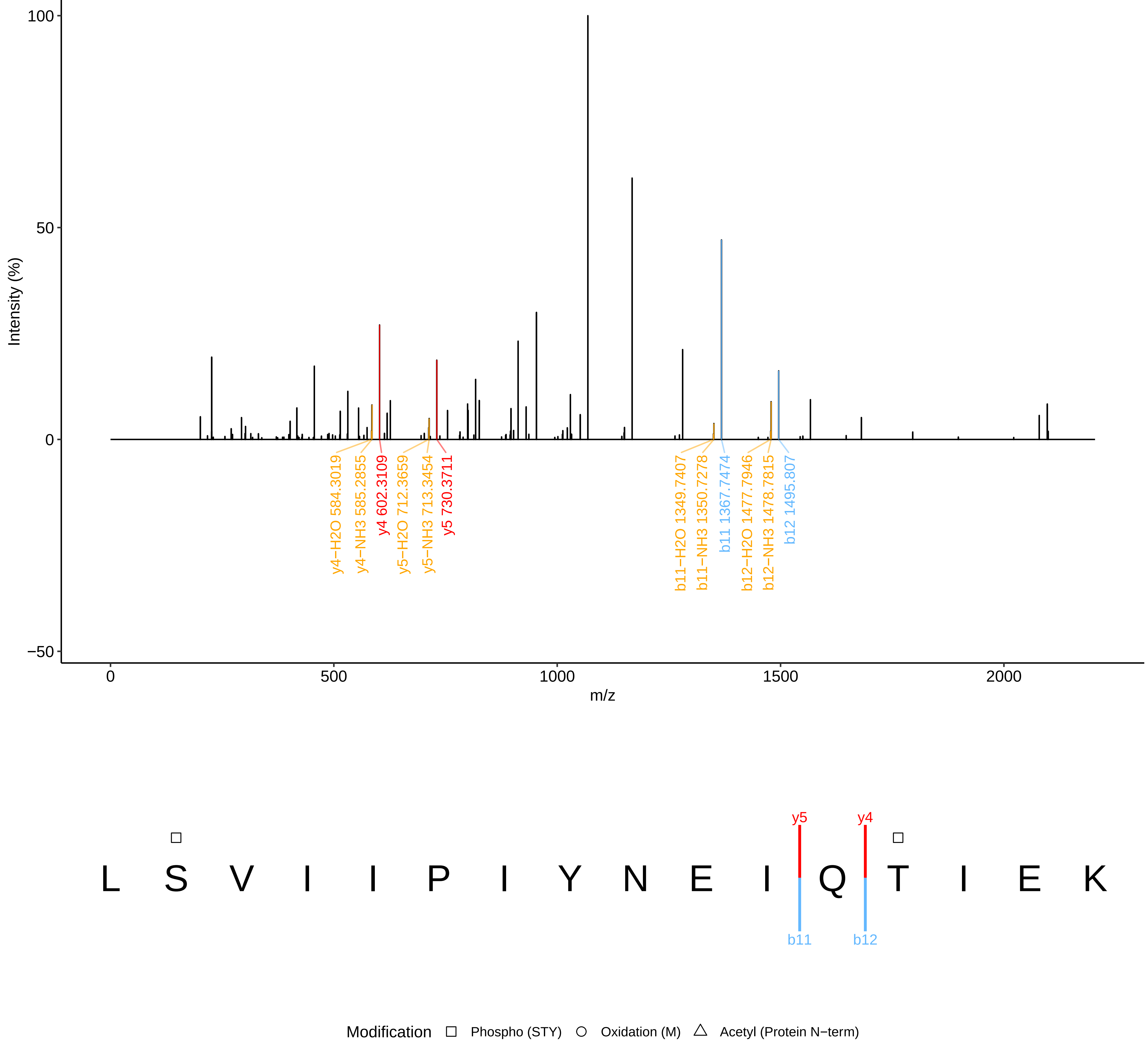

20210828_PS_SCy031_phospho_R2_TiO2_LC3h_F2 − Scan number: 10183

Gene Names	Charge	m/z	Mass	Mass error [Da]	Mass error [ppm]	Retention time	PEP	Score	Precursor Intensity
sll1031	3	673.3199	2016.938	0.00049636	0.73718	15.733	4.0403e−66	231.44	34233284

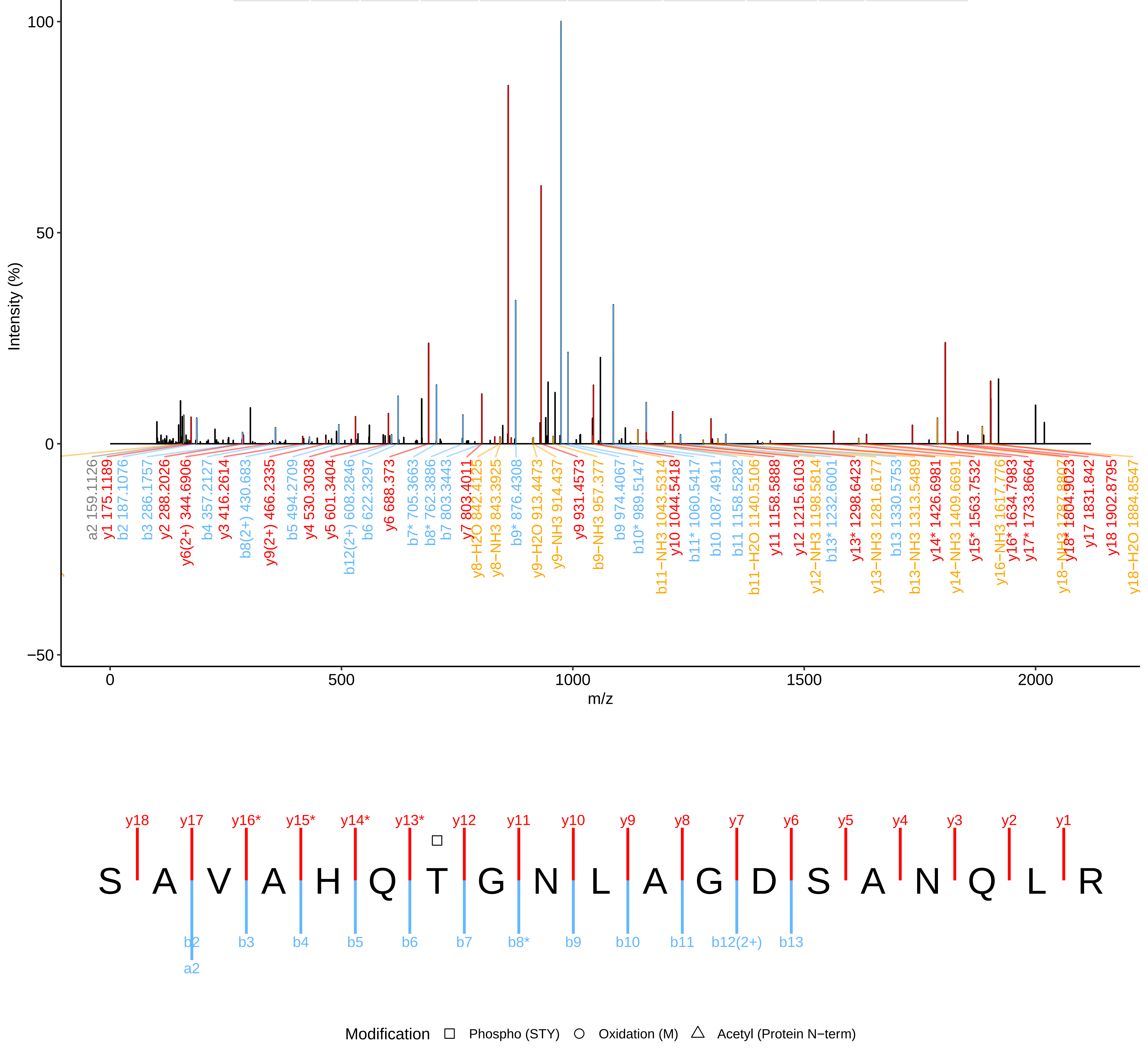

20210828_PS_SCy031_phospho_R3_TiO2_LC24h_F1 − Scan number: 10955

Gene Names	Charge	m/z	Mass	Mass error [Da]	Mass error [ppm]	Retention time	PEP	Score	Precursor Intensity
sll1031	3	1002.75	3005.227	0.0017448	1.7394	16.531	3.3603e−41	155.61	6594457

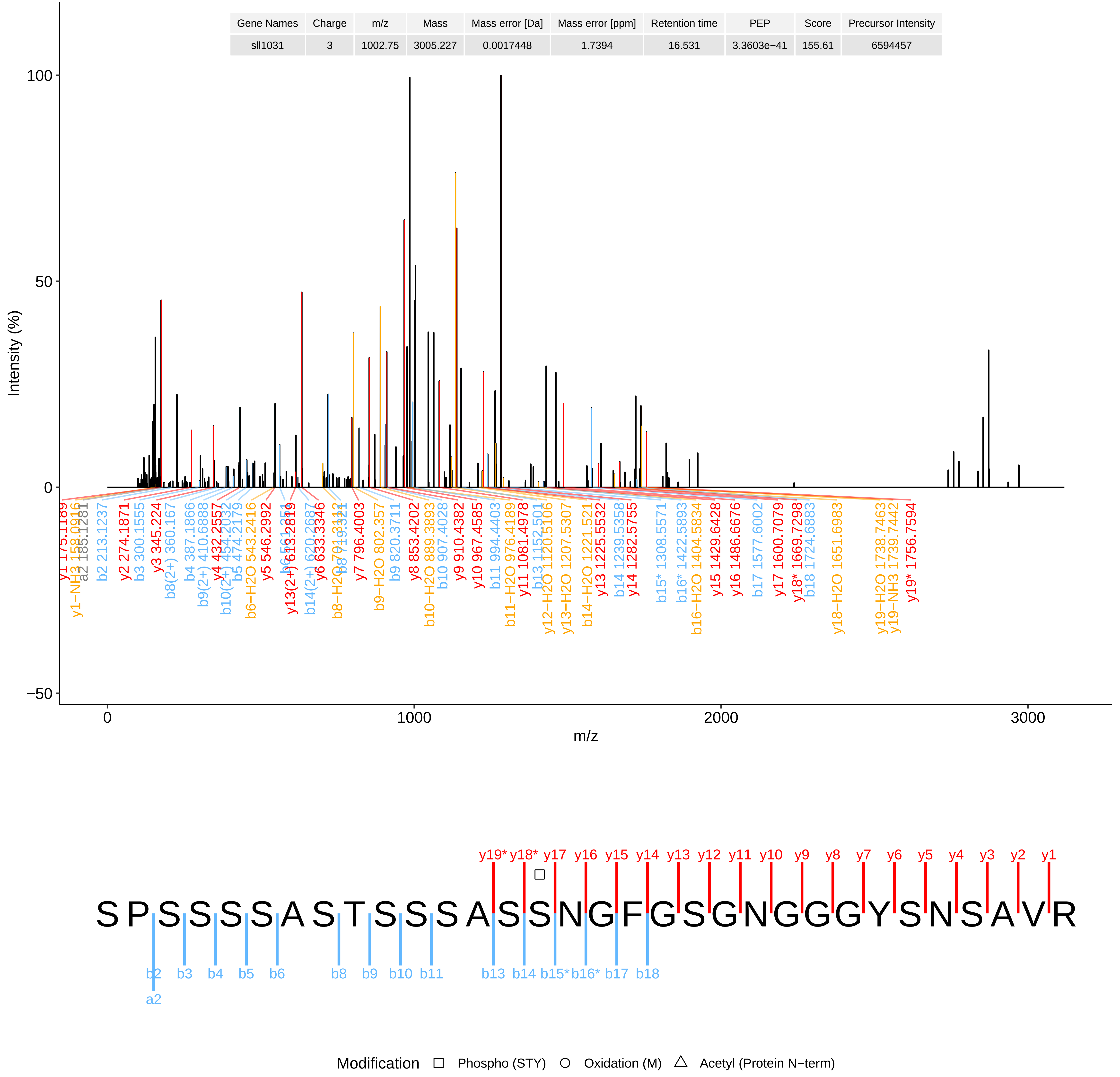

20210828_PS_SCy031_phospho_R3_TiO2_LC24h_F2 − Scan number: 13649

Gene Names	Charge	m/z	Mass	Mass error [Da]	Mass error [ppm]	Retention time	PEP	Score	Precursor Intensity
sll1031	2	619.8107	1237.607	NA	NA	20.168	0.00094721	128.35	3558445

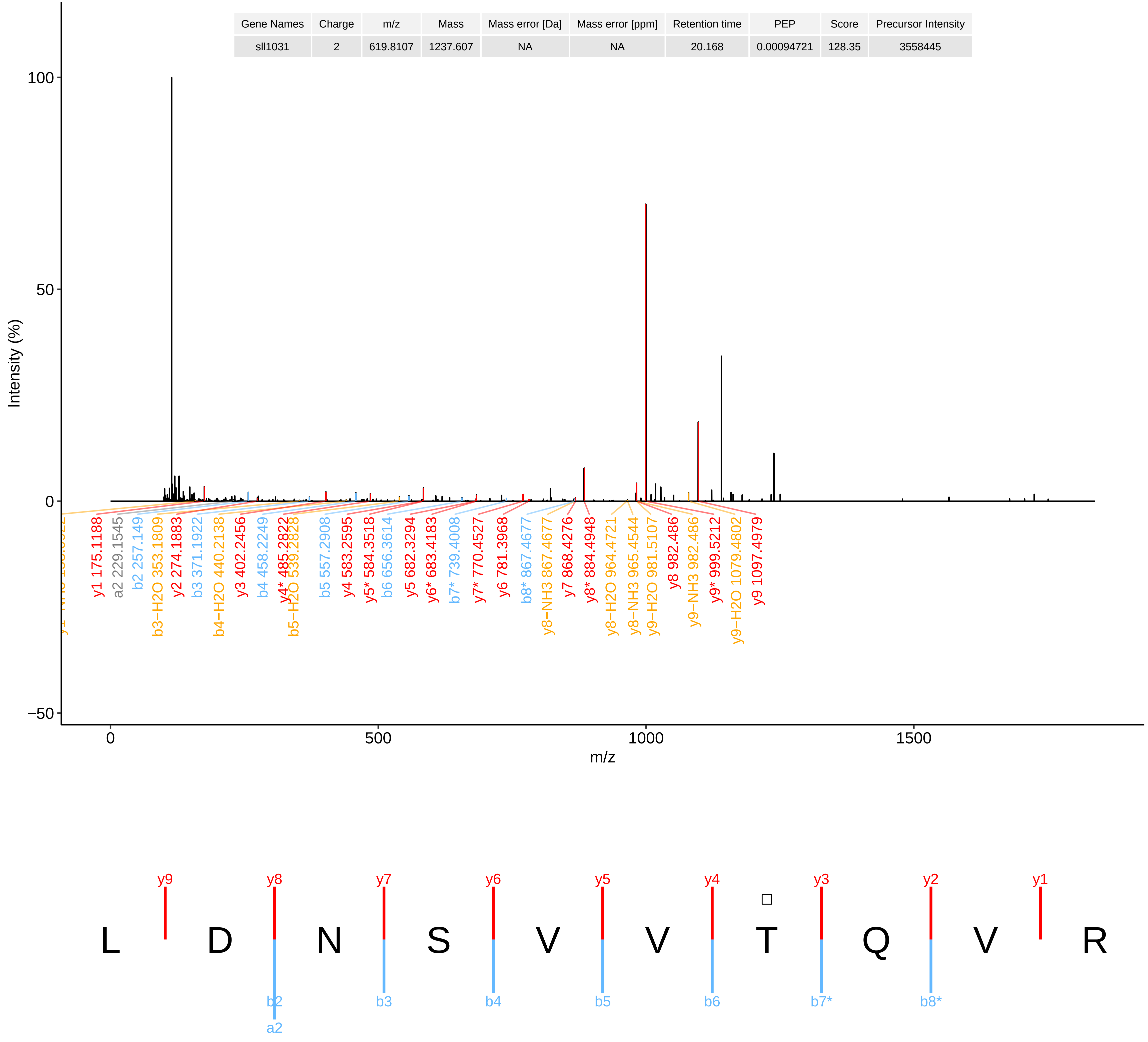

Modification □ Phospho (STY) ○ Acetyl (Protein N−term) △ Oxidation (M)

20210828_PS_SCy031_phospho_R3_TiO2_HC_F2 − Scan number: 9374

Gene Names	Charge	m/z	Mass	Mass error [Da]	Mass error [ppm]	Retention time	PEP	Score	Precursor Intensity
sll1031	4	723.6157	2890.434	0.00052786	0.72897	14.768	0.00023179	68.269	2817494

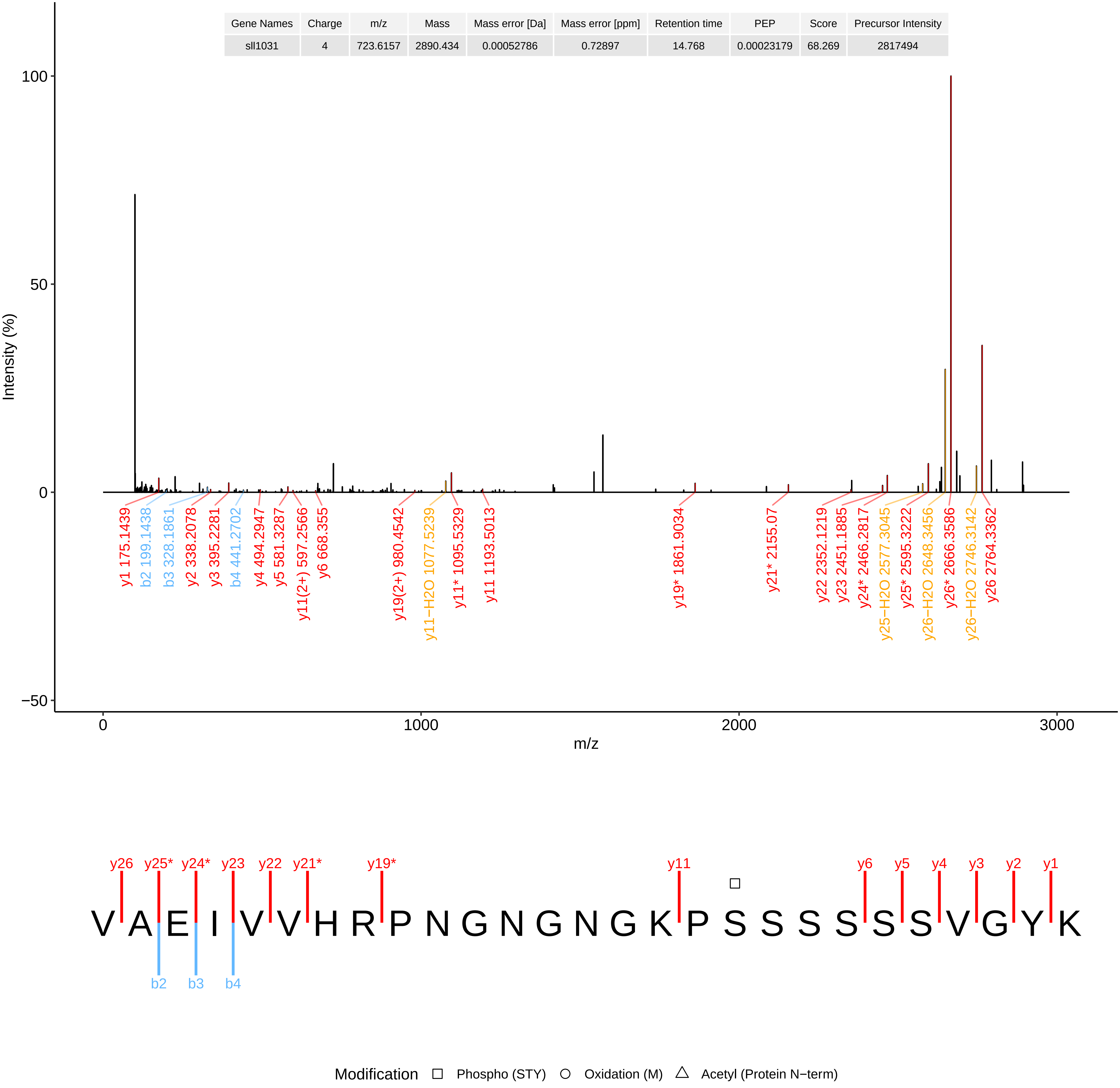

20210828_PS_SCy031_phospho_R3_TiO2_LC3h_F3 − Scan number: 7433

Gene Names	Charge	m/z	Mass	Mass error [Da]	Mass error [ppm]	Retention time	PEP	Score	Precursor Intensity
sll1101	2	497.7521	993.4896	0.0014359	2.9684	12.428	0.019939	43.979	2649579

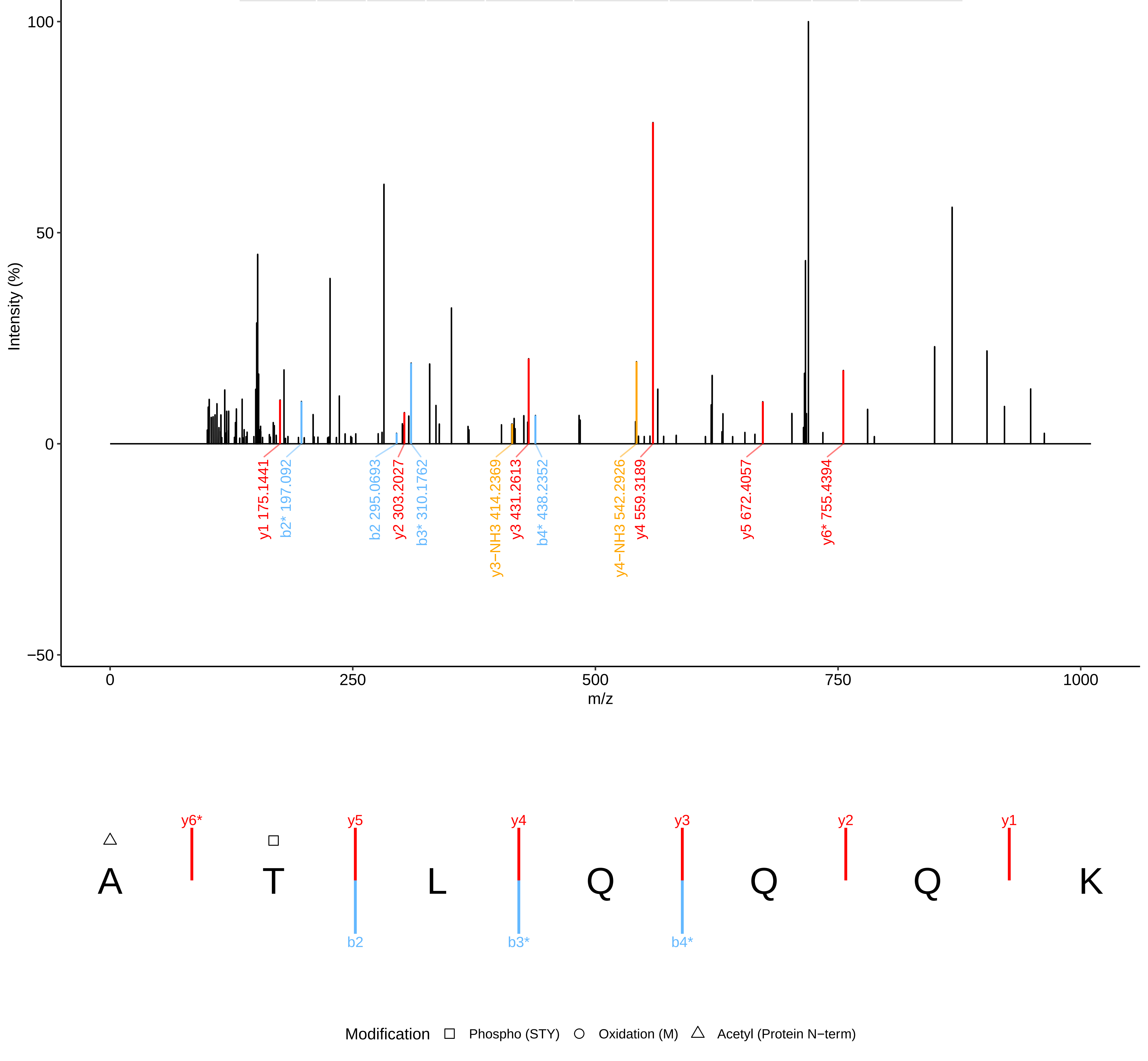

20210828_PS_SCy031_phospho_R3_TiO2_LC3h_F3 − Scan number: 13527

Gene Names	Charge	m/z	Mass	Mass error [Da]	Mass error [ppm]	Retention time	PEP	Score	Precursor Intensity
sll1244	2	587.3401	1172.666	9.4763e−05	0.16134	20.076	0.0010014	149.23	5412064

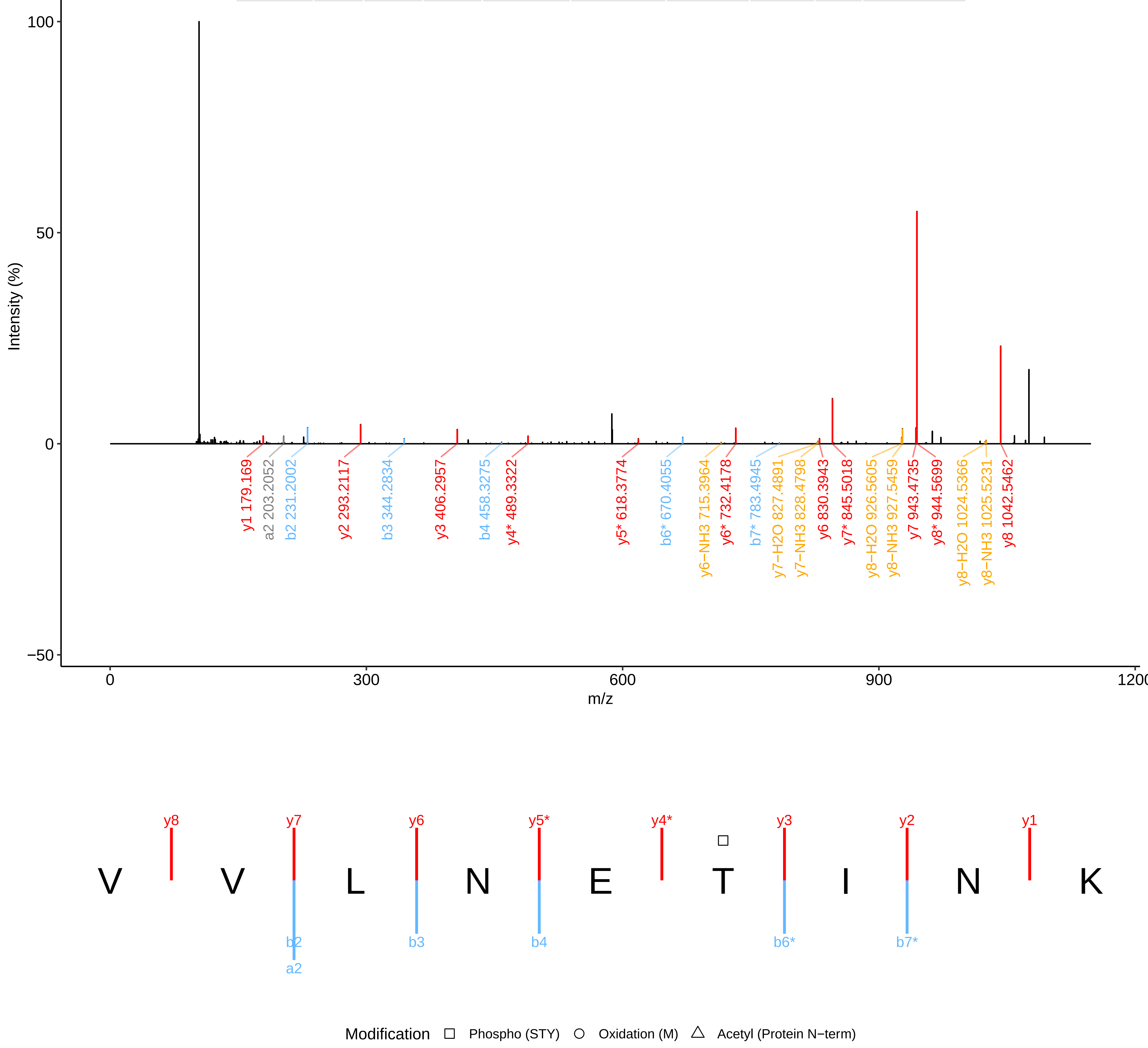

20210823_PS_SCy031_R1_phospho_LC3h_TiO2_1 − Scan number: 15122

Gene Names	Charge	m/z	Mass	Mass error [Da]	Mass error [ppm]	Retention time	PEP	Score	Precursor Intensity
sll1292	2	890.9911	1779.968	−0.0029911	−3.3571	21.773	0.0080266	78.934	15522496

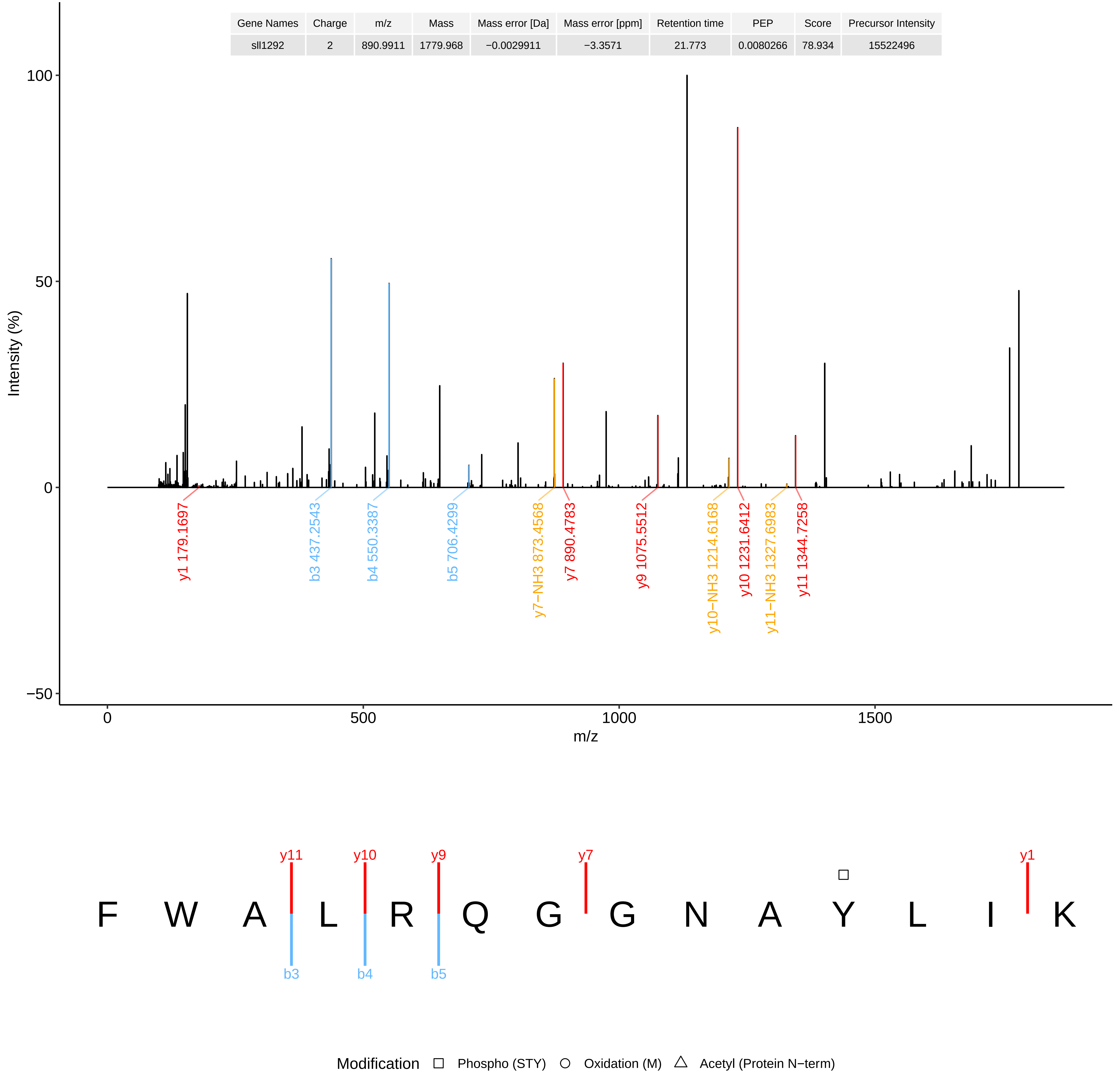

20210828_PS_SCy031_phospho_R3_TiO2_LC24h_F2 − Scan number: 20002

Gene Names	Charge	m/z	Mass	Mass error [Da]	Mass error [ppm]	Retention time	PEP	Score	Precursor Intensity
sll1316	2	860.4067	1718.799	0.00052772	0.61334	28.165	8.7375e−08	137.19	7871910

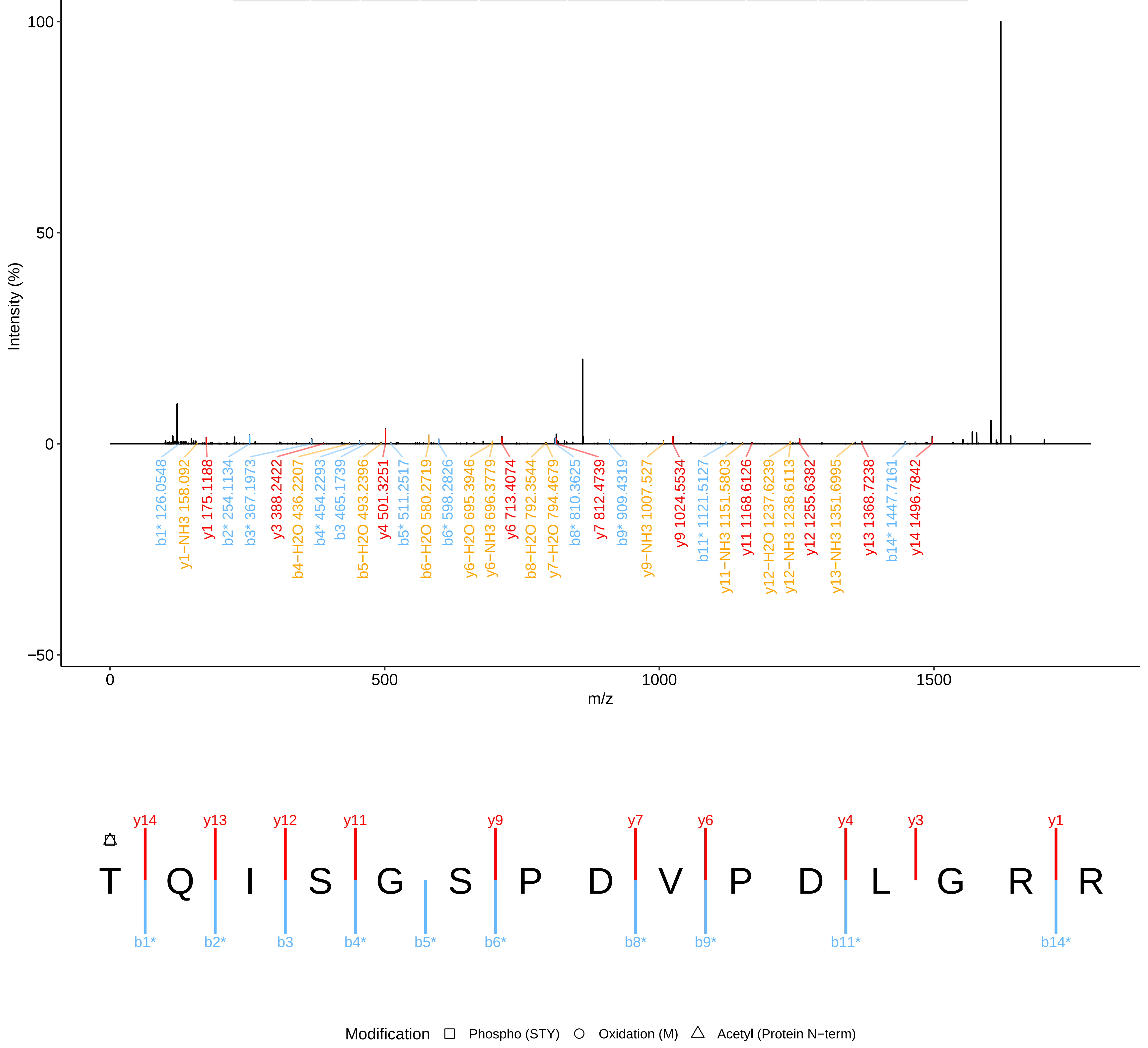

20210823_PS_SCy031_R1_phospho_LC24h_TiO2_1 − Scan number: 28520

Gene Names	Charge	m/z	Mass	Mass error [Da]	Mass error [ppm]	Retention time	PEP	Score	Precursor Intensity
sll1341	2	755.3515	1508.688	−0.00015724	−0.20816	38.611	2.0279e−05	145.31	4198293

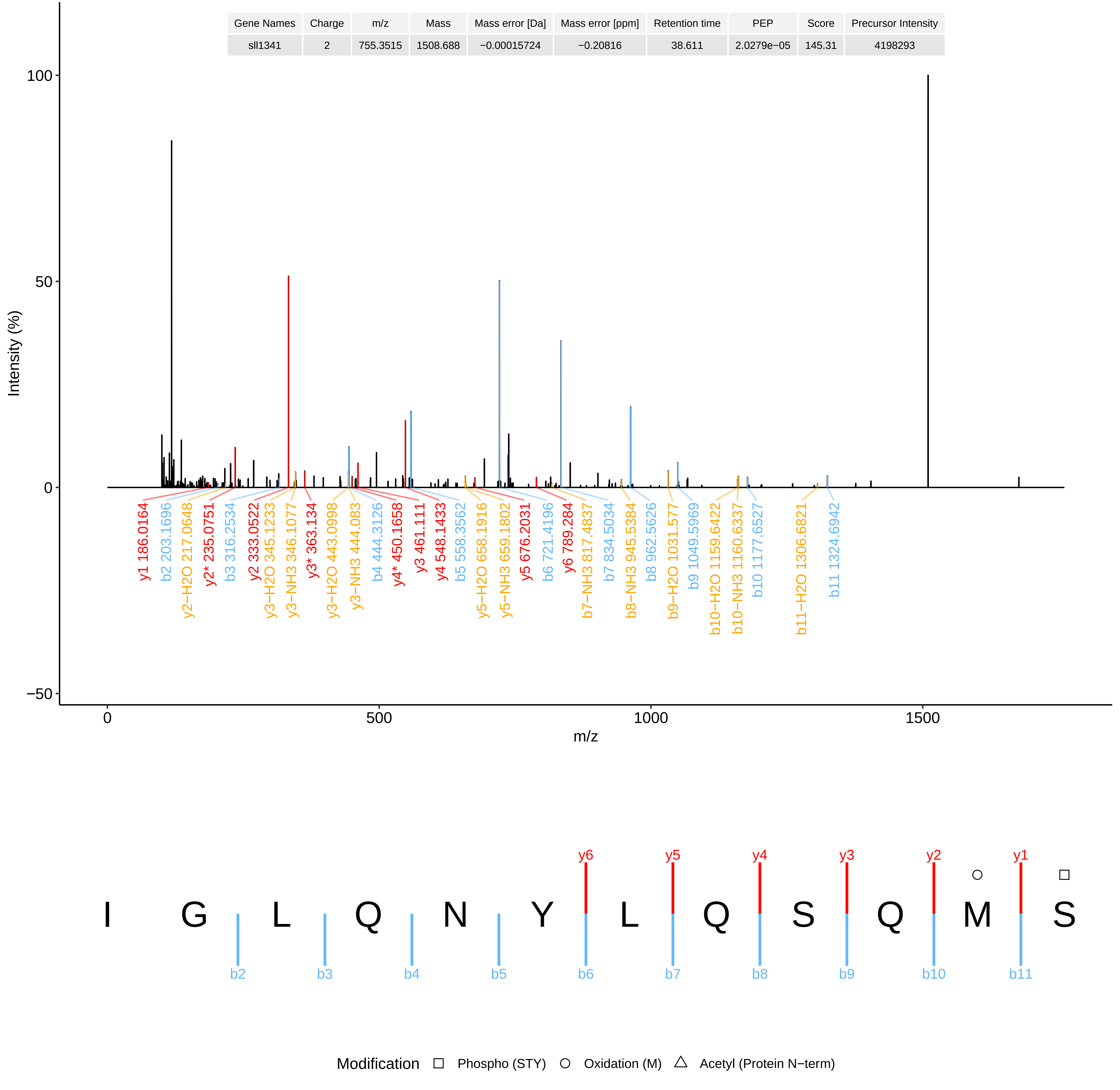

20210823_PS_SCy031_R1_phospho_LC24h_TiO2_3 − Scan number: 30815

Gene Names	Charge	m/z	Mass	Mass error [Da]	Mass error [ppm]	Retention time	PEP	Score	Precursor Intensity
sll1350	2	960.4922	1918.97	18.037	19512	41.746	0.0029153	45.115	3915722

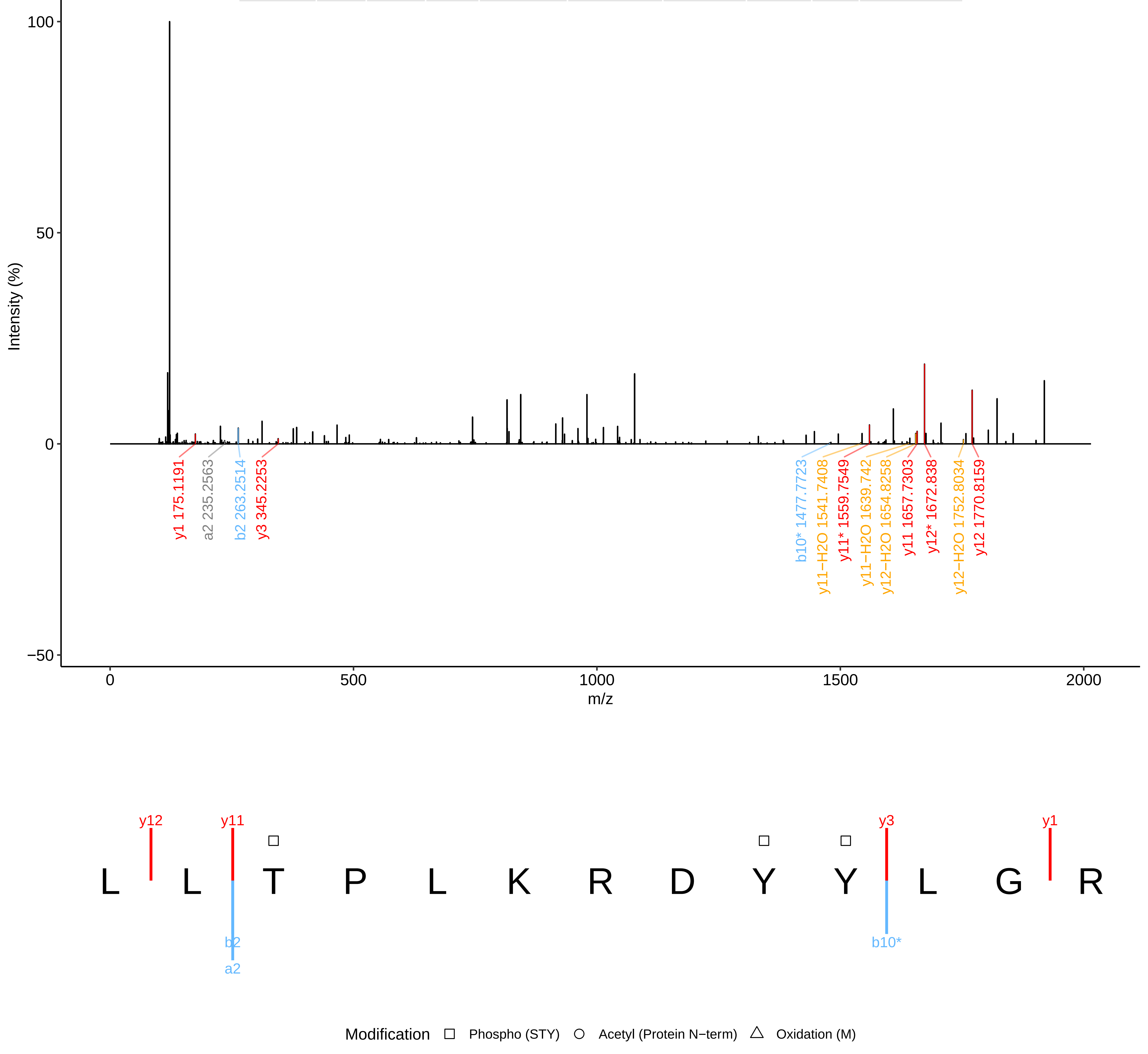

20210823_PS_SCy031_R1_phospho_LC24h_TiO2_2 − Scan number: 26845

Gene Names	Charge	m/z	Mass	Mass error [Da]	Mass error [ppm]	Retention time	PEP	Score	Precursor Intensity
sll1367	3	1004.169	3009.484	0.0012993	1.3257	36.744	0.0022574	45.011	22548562

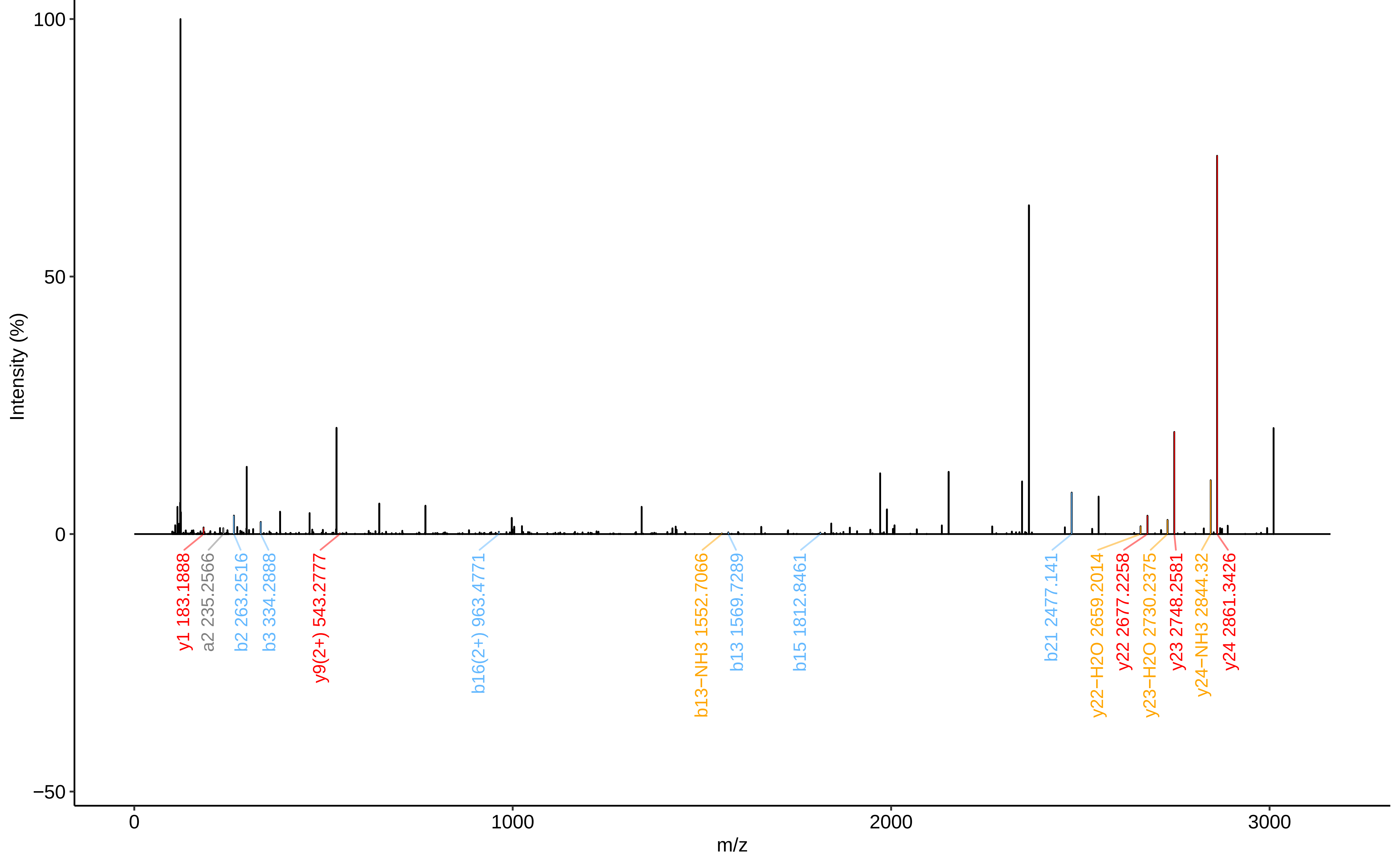

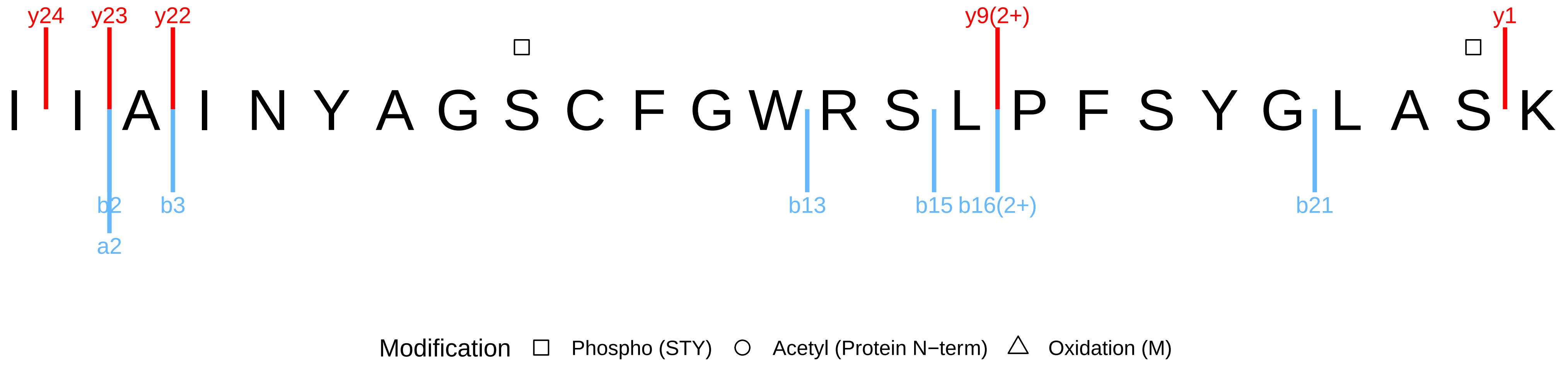

20210828_PS_SCy031_phospho_R2_TiO2_LC3h_F3 − Scan number: 24422

Gene Names	Charge	m/z	Mass	Mass error [Da]	Mass error [ppm]	Retention time	PEP	Score	Precursor Intensity
sll1426	3	1143.144	3426.411	0.0010769	0.95208	33.91	0.00032944	55.662	14254178

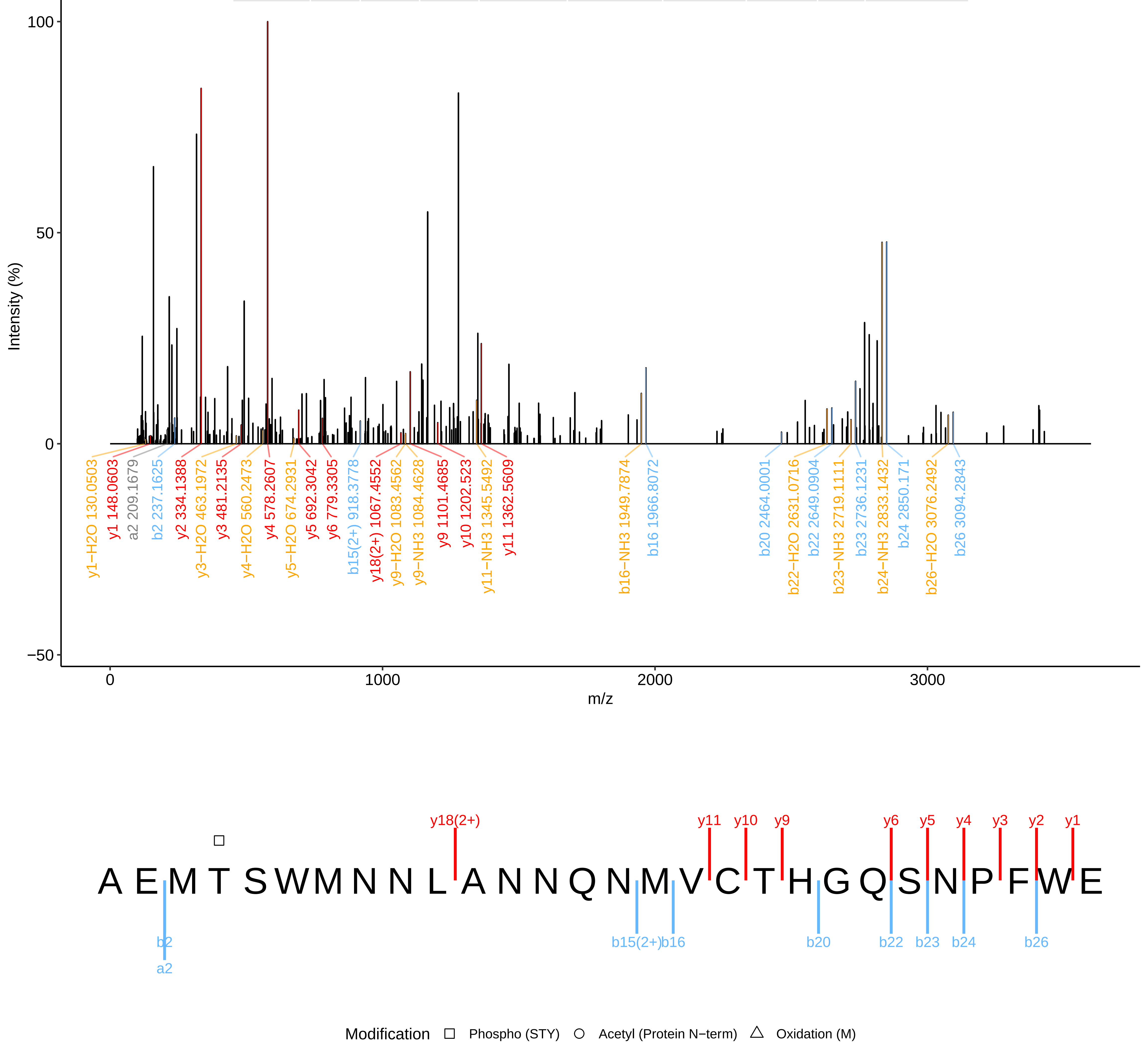

20210828_PS_SCy031_phospho_R2_TiO2_LC3h_F2 − Scan number: 7780

Gene Names	Charge	m/z	Mass	Mass error [Da]	Mass error [ppm]	Retention time	PEP	Score	Precursor Intensity
sll1434	2	721.4006	1440.787	0.00076428	1.0835	12.729	0.0014093	77.894	9406395

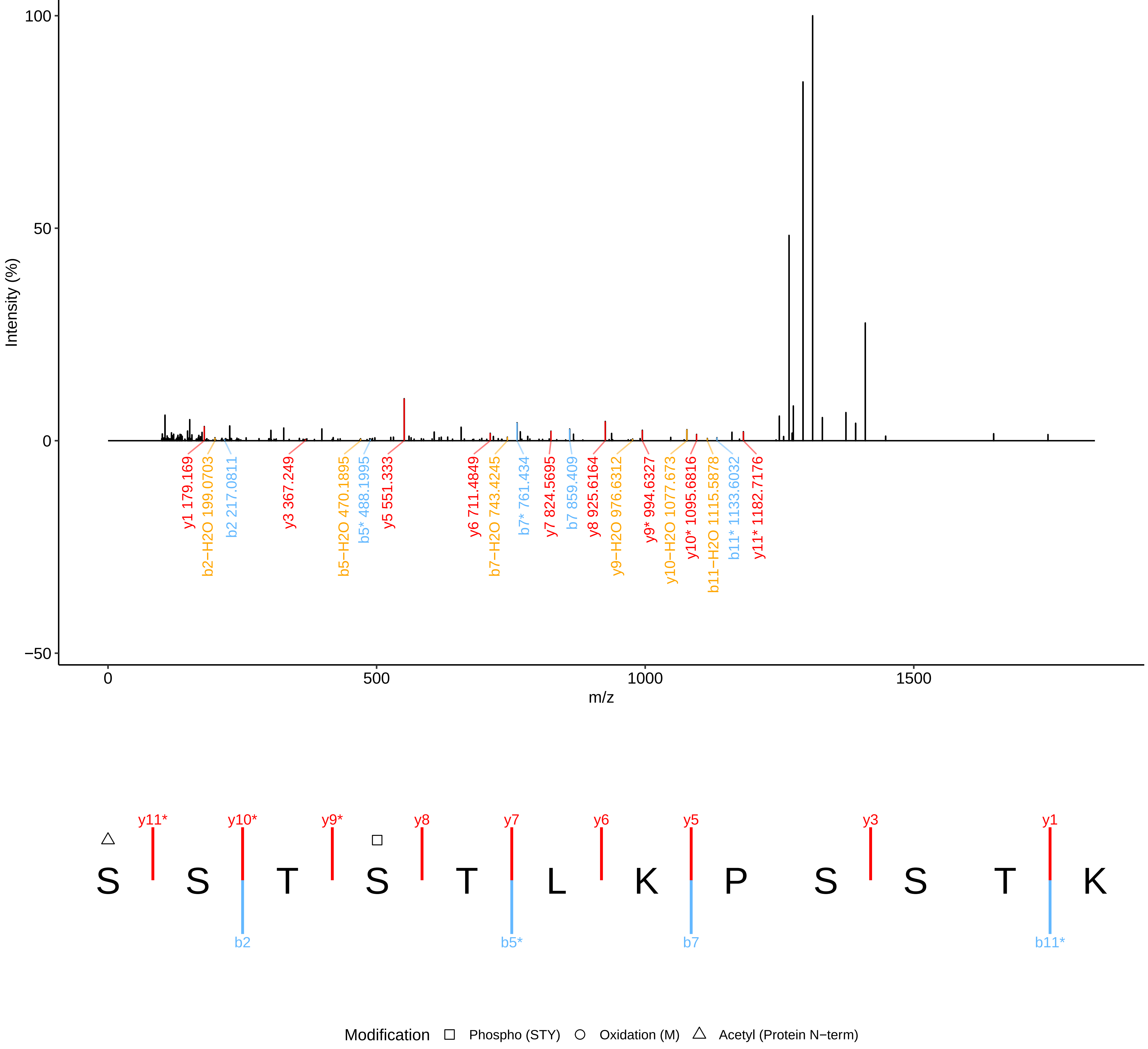

20211212_PS_SCy031_proteome_R1_24hLC_F6 − Scan number: 13949

Gene Names	Charge	m/z	Mass	Mass error [Da]	Mass error [ppm]	Retention time	PEP	Score	Precursor Intensity
sll1464	2	903.9665	1805.918	NA	NA	23.39	0.0095736	47.198	1558253

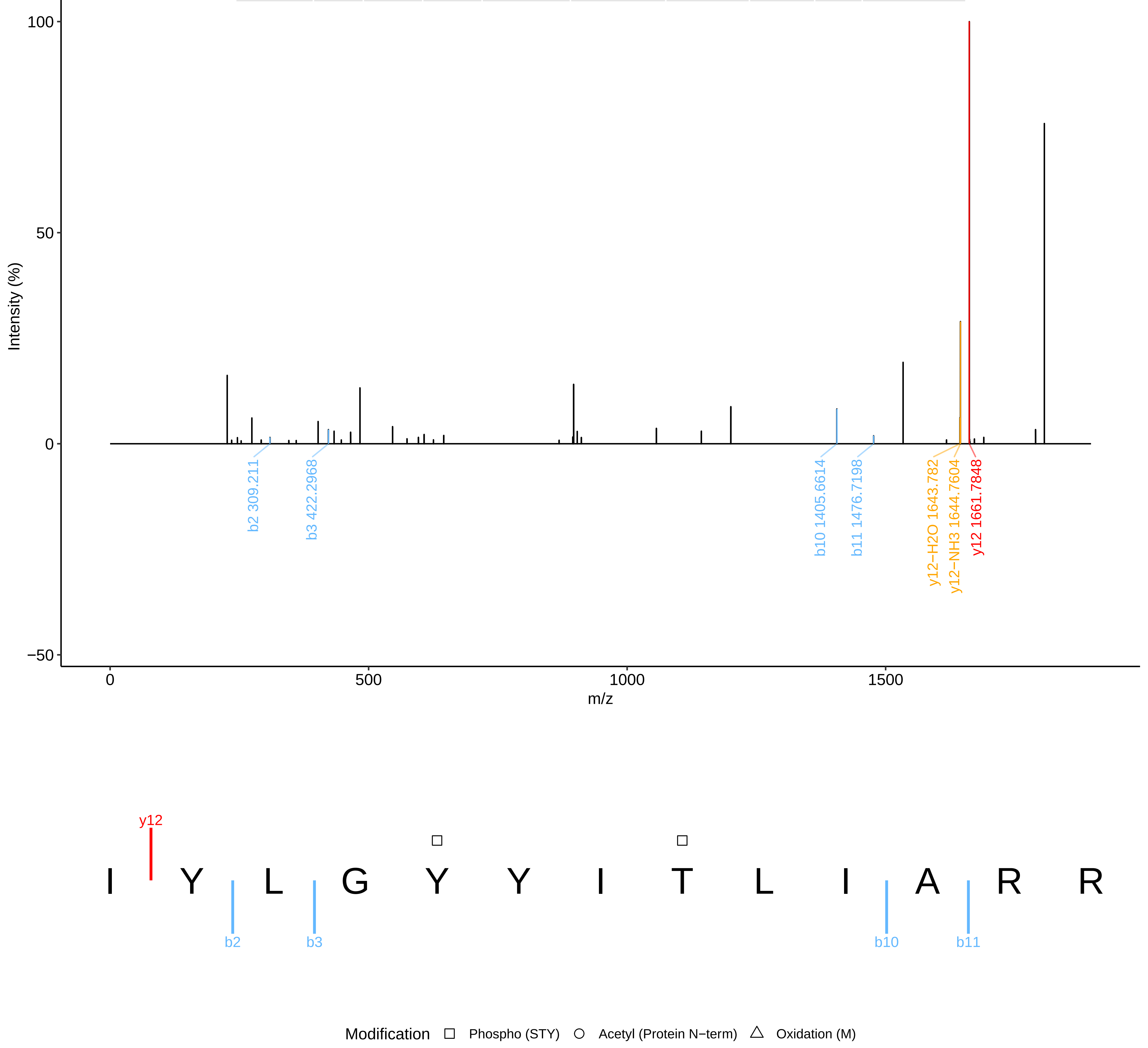

20211212_PS_SCy031_proteome_R1_24hLC_F6 − Scan number: 30518

Gene Names	Charge	m/z	Mass	Mass error [Da]	Mass error [ppm]	Retention time	PEP	Score	Precursor Intensity
sll1536	3	777.7153	2330.124	18.686	25239	45.575	0.0057675	42.813	4345740

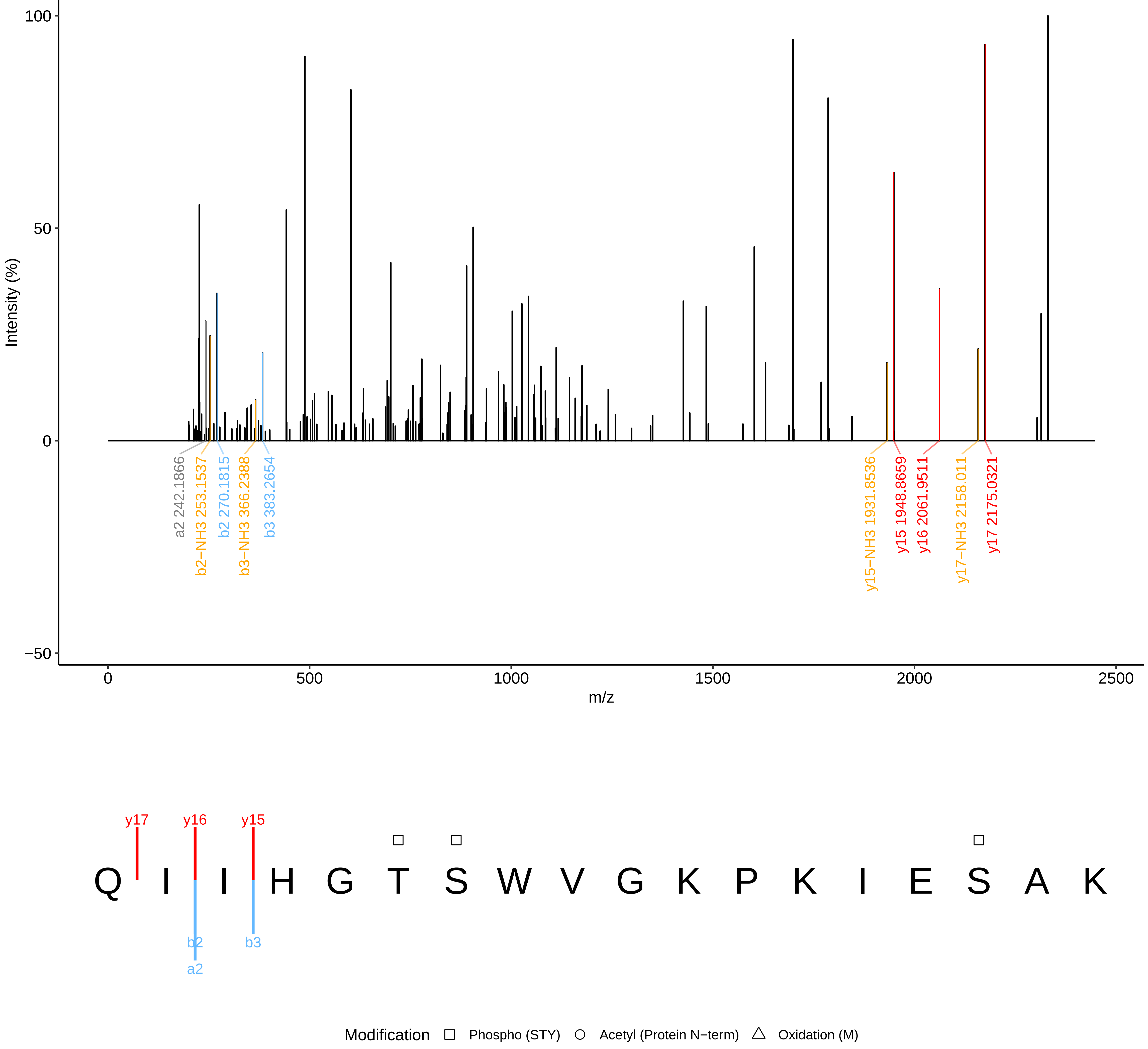

20211217_PS_SCy031_Proteome_R3_HC_F3 − Scan number: 20103

Gene Names	Charge	m/z	Mass	Mass error [Da]	Mass error [ppm]	Retention time	PEP	Score	Precursor Intensity
sll1542	2	734.8509	1467.687	0.000274	0.38012	30.004	0.016654	44.753	16250623

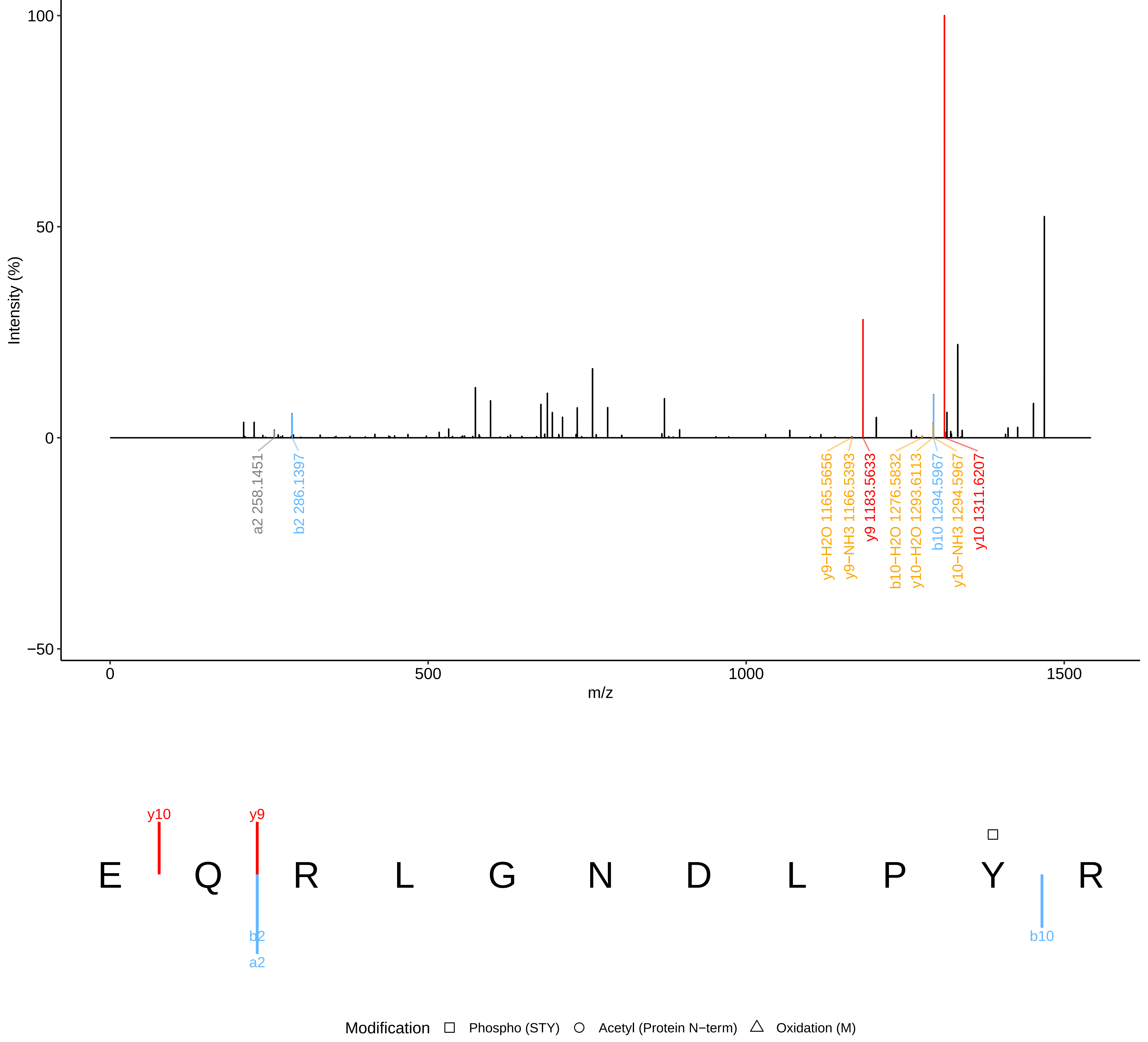

20210828_PS_SCy031_phospho_R2_TiO2_LC3h_F1 − Scan number: 13012

Gene Names	Charge	m/z	Mass	Mass error [Da]	Mass error [ppm]	Retention time	PEP	Score	Precursor Intensity
sll1545	3	947.0636	2838.169	−0.00021055	−0.22224	19.313	1.0754e−18	124.82	4571725

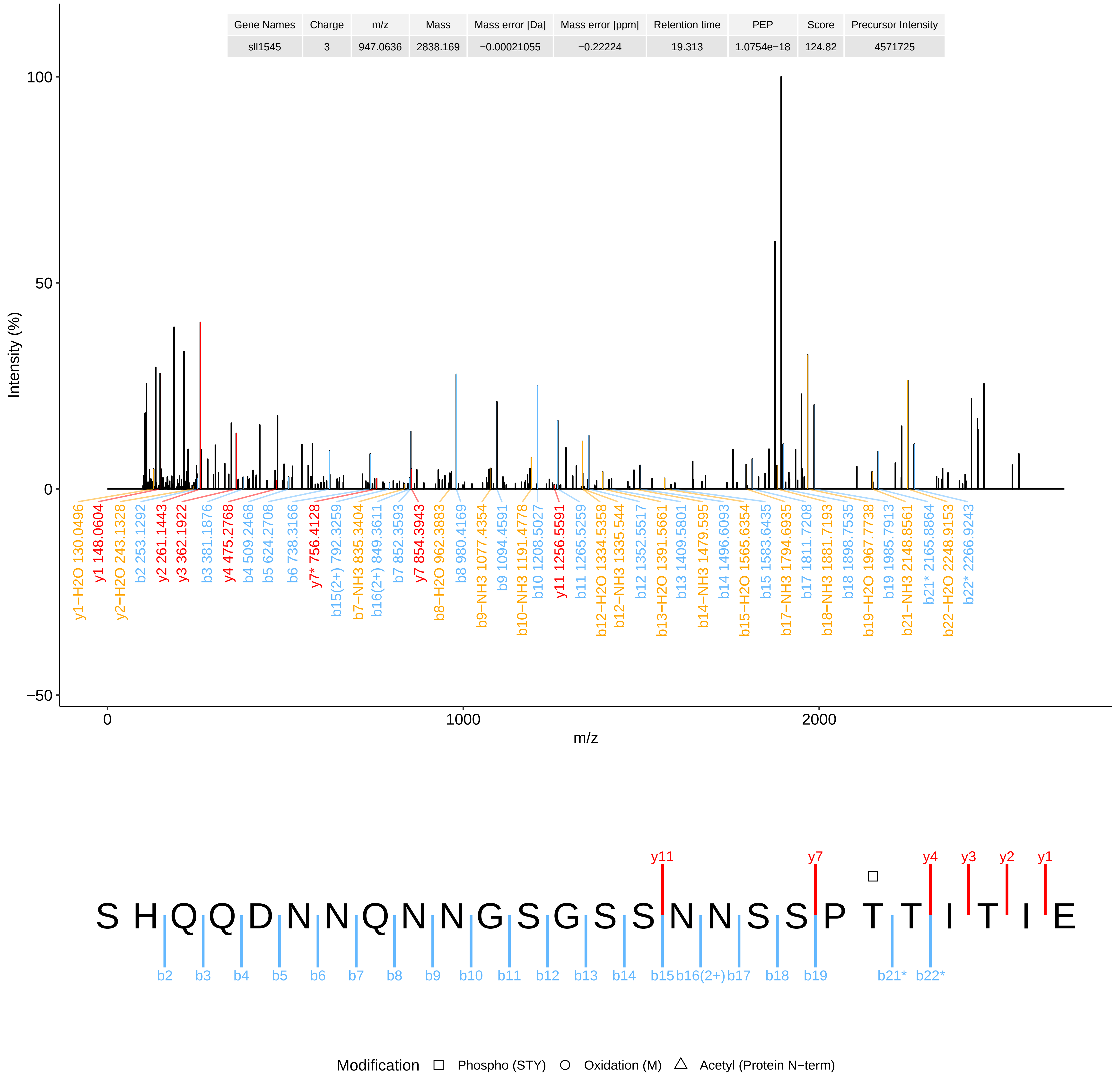

20210828_PS_SCy031_phospho_R3_TiO2_LC3h_F3 − Scan number: 3708

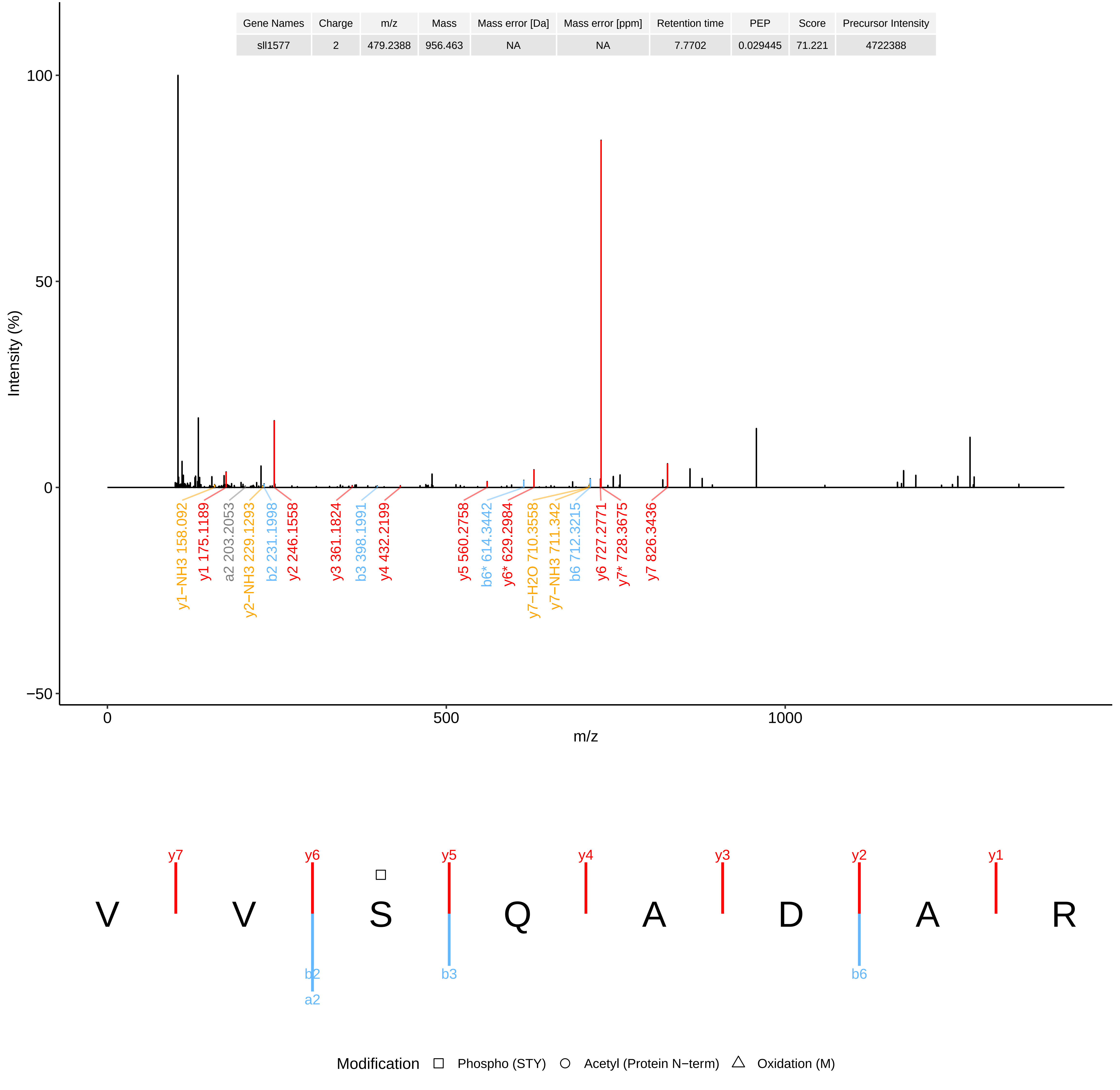

Gene Names	Charge	m/z	Mass	Mass error [Da]	Mass error [ppm]	Retention time	PEP	Score	Precursor Intensity
sll1577	2	479.2388	956.463	NA	NA	7.7702	0.029445	71.221	4722388

20210828_PS_SCy031_phospho_R3_TiO2_LC24h_F2 − Scan number: 31448

Gene Names	Charge	m/z	Mass	Mass error [Da]	Mass error [ppm]	Retention time	PEP	Score	Precursor Intensity
sll1577	2	1131.577	2261.14	0.00048989	0.44719	42.601	0	431.74	17746192

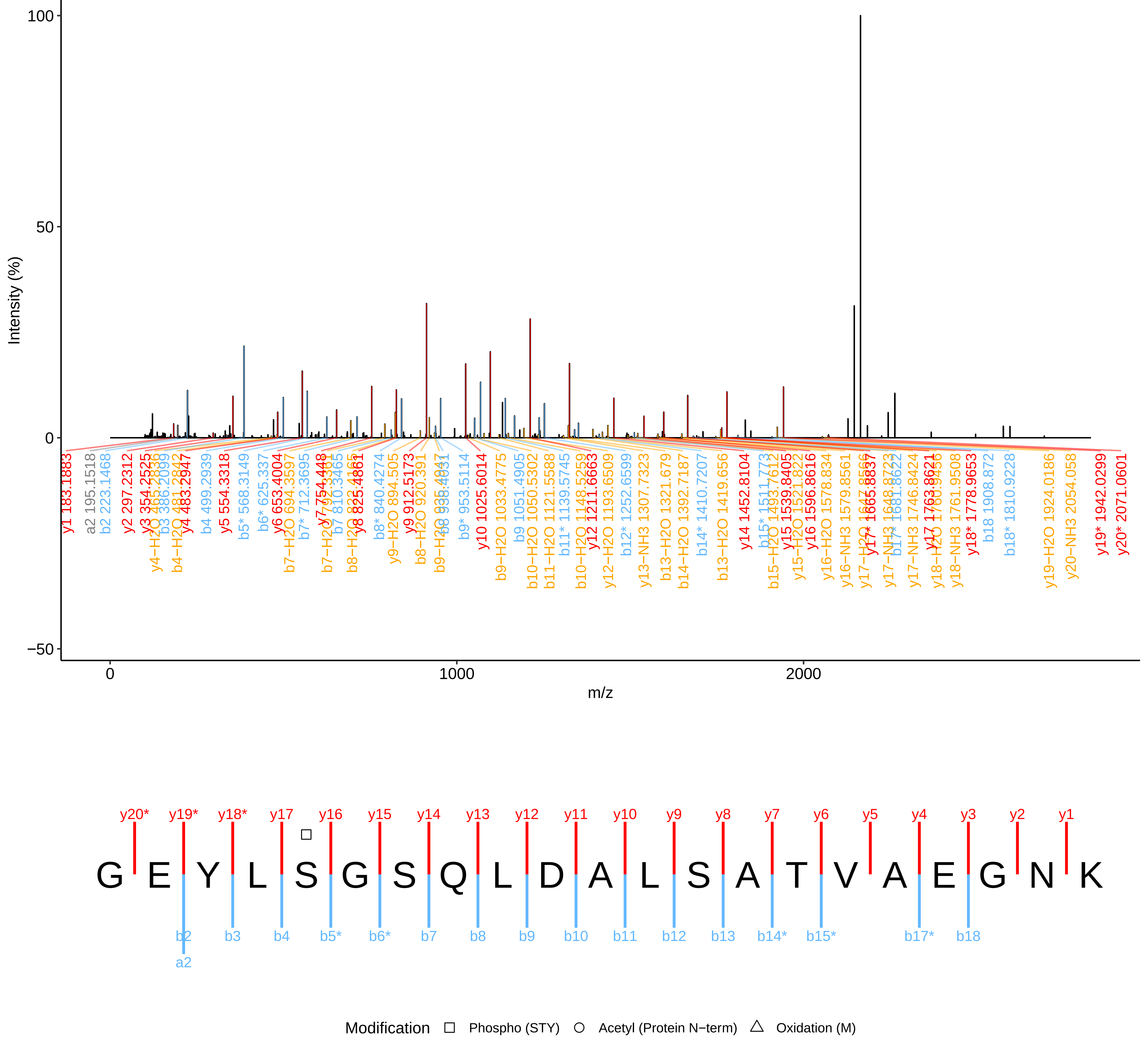

20210828_PS_SCy031_phospho_R3_TiO2_LC3h_F2 − Scan number: 32243

Gene Names	Charge	m/z	Mass	Mass error [Da]	Mass error [ppm]	Retention time	PEP	Score	Precursor Intensity
sll1577	2	1123.533	2245.052	0.00044245	0.40388	43.578	0	446	99541168

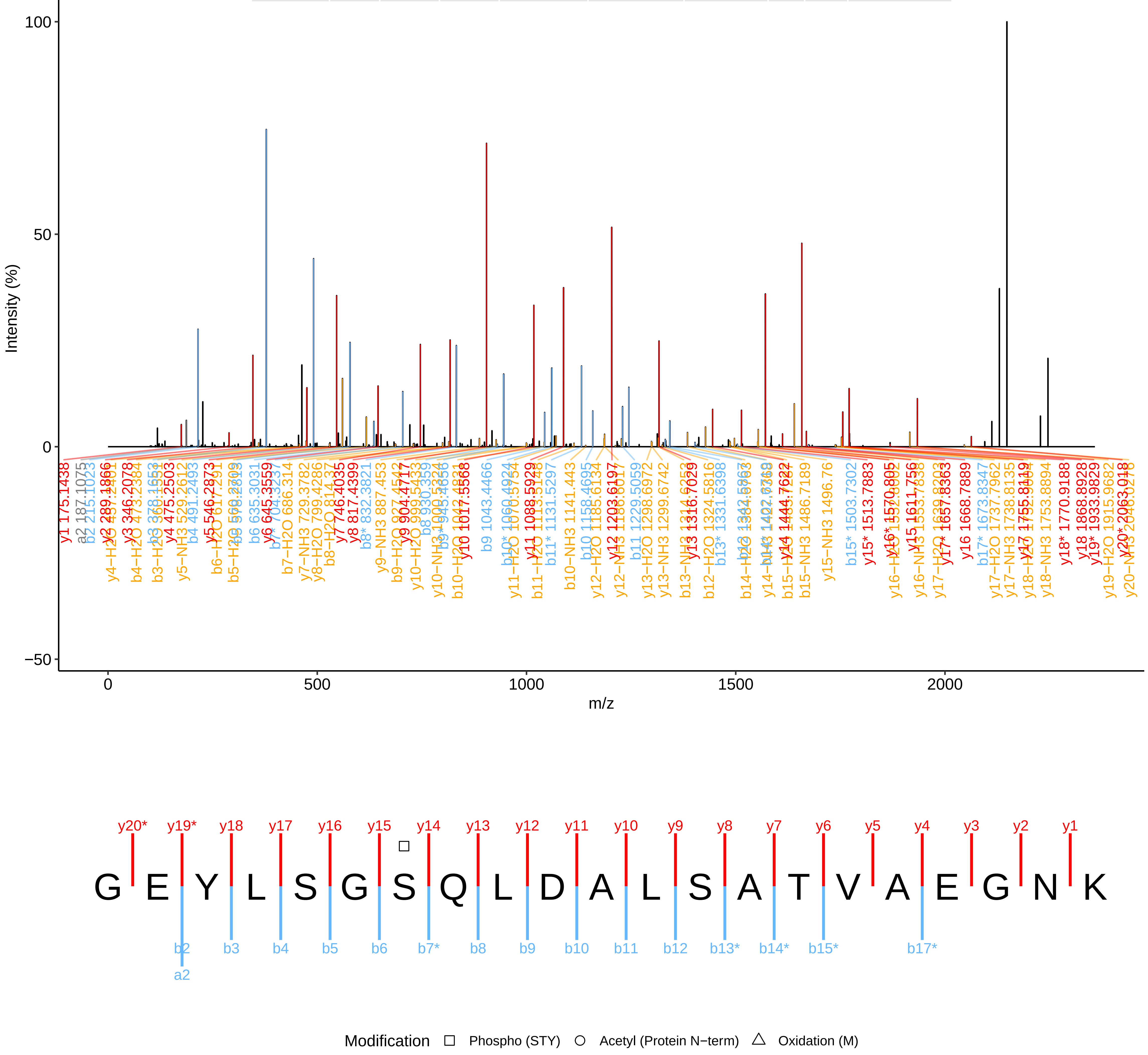

20210828_PS_SCy031_phospho_R3_TiO2_HC_F3 − Scan number: 26095

Gene Names	Charge	m/z	Mass	Mass error [Da]	Mass error [ppm]	Retention time	PEP	Score	Precursor Intensity
sll1577	3	754.7207	2261.14	−0.0026894	−3.5634	35.956	3.9063e−12	136.4	NA

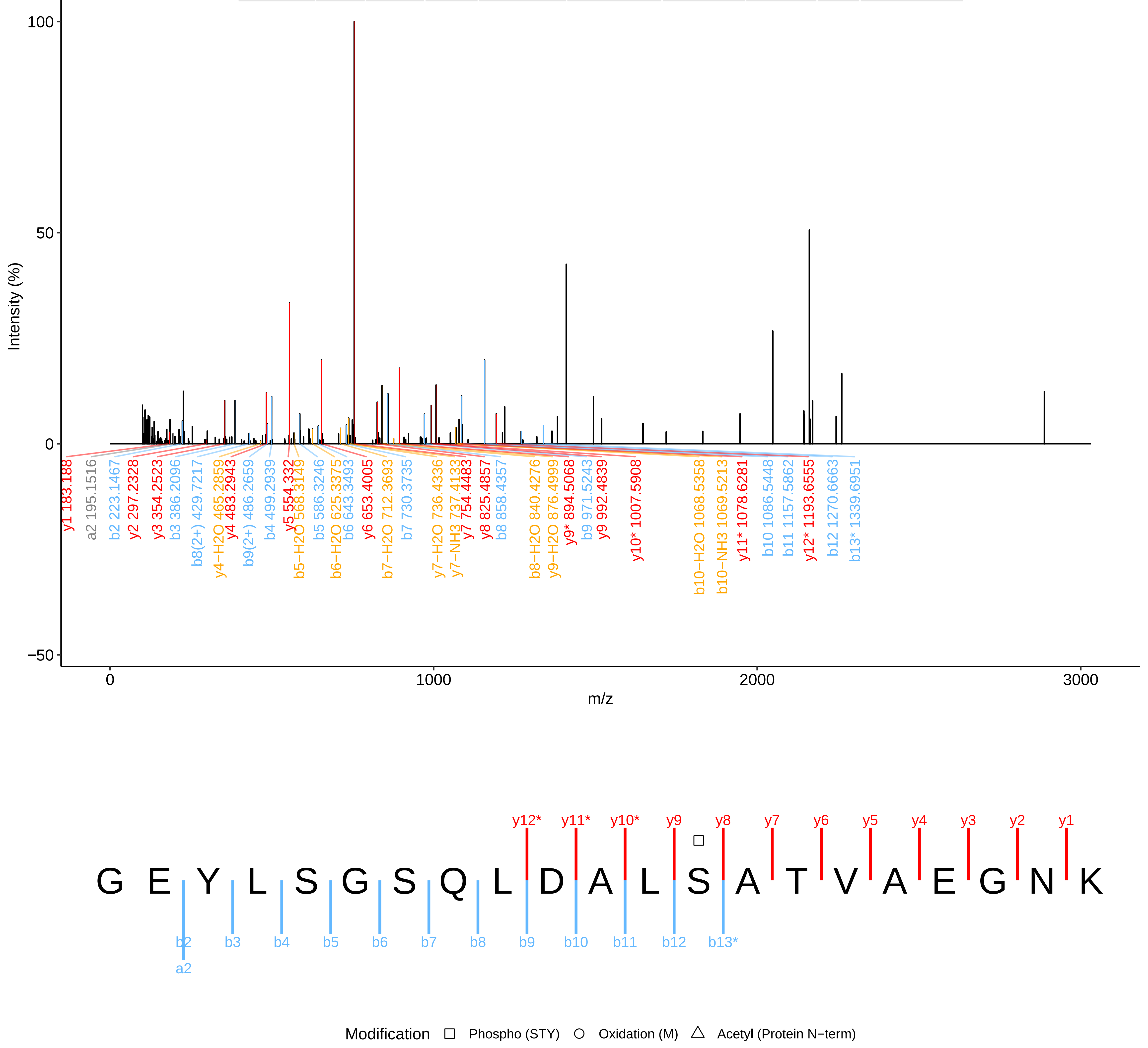

20210828_PS_SCy031_phospho_R3_TiO2_HC_F1 − Scan number: 3730

Gene Names	Charge	m/z	Mass	Mass error [Da]	Mass error [ppm]	Retention time	PEP	Score	Precursor Intensity
sll1577	2	488.2619	974.5092	NA	NA	7.5802	0.034585	72.321	16473532

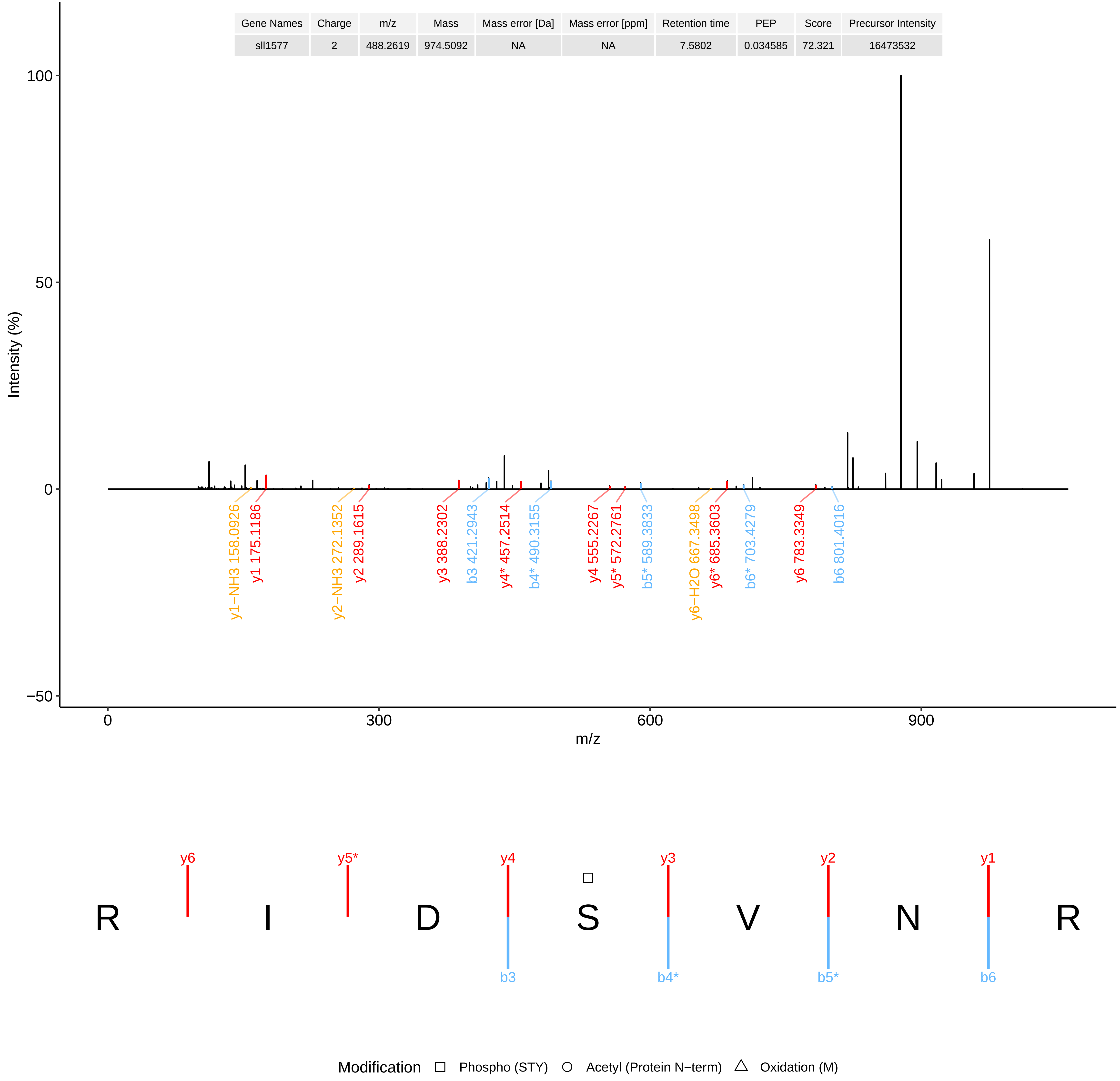

20210828_PS_SCy031_phospho_R2_TiO2_LC3h_F1 − Scan number: 16359

Gene Names	Charge	m/z	Mass	Mass error [Da]	Mass error [ppm]	Retention time	PEP	Score	Precursor Intensity
sll1577	2	730.8861	1459.758	−0.00045064	−0.63217	23.515	3.8093e−29	191.83	25879790

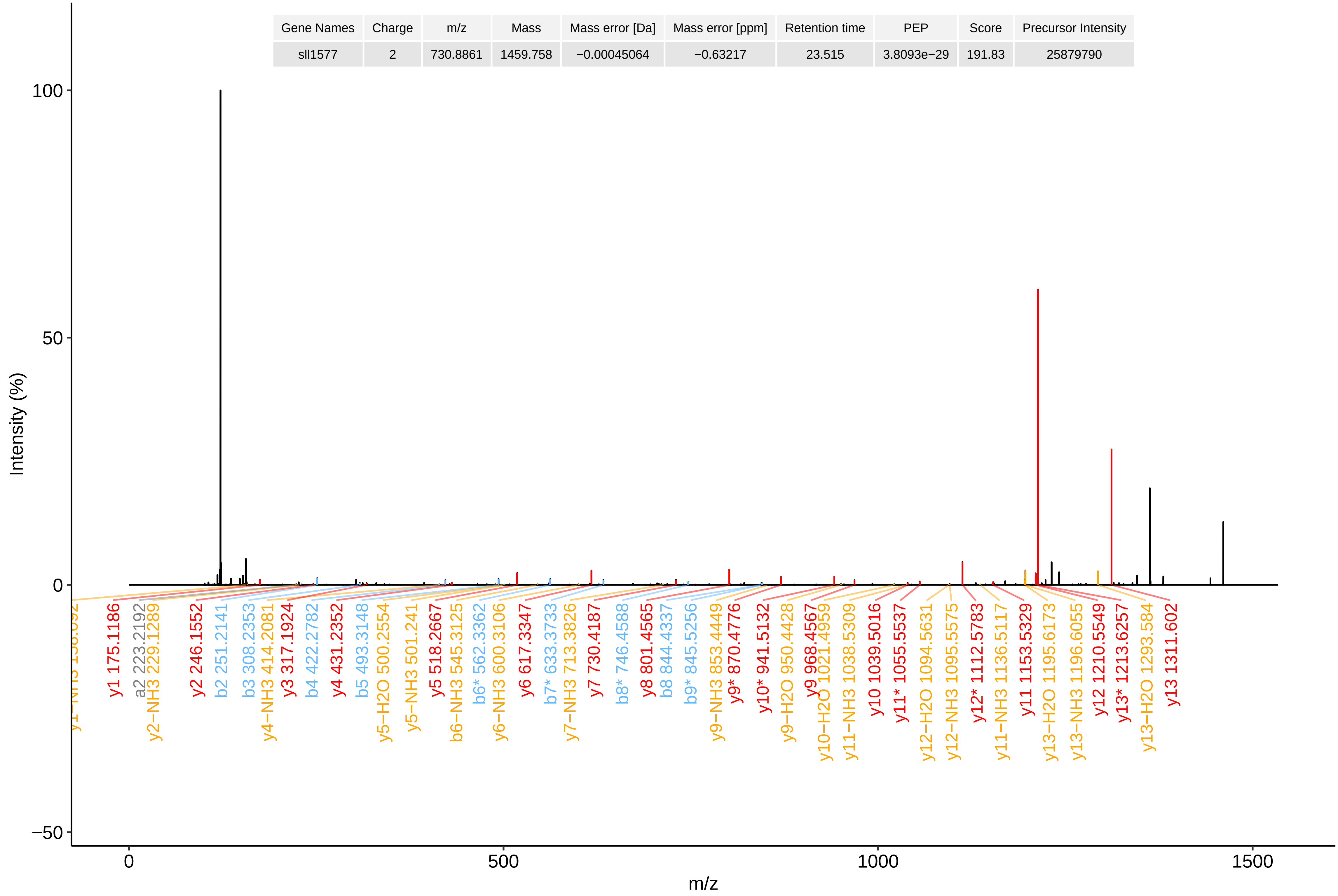

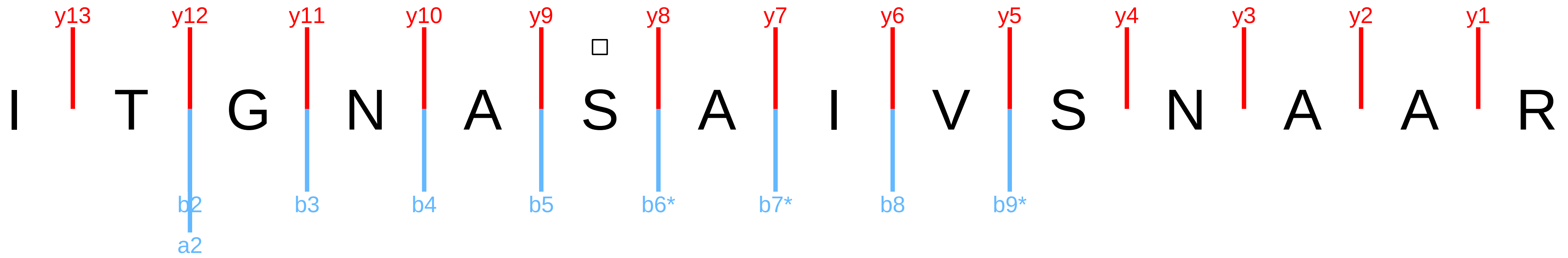

Modification □ Phospho (STY) ○ Acetyl (Protein N−term) △ Oxidation (M)

20210823_PS_SCy031_R1_phospho_HC_TiO2_2 − Scan number: 14245

Gene Names	Charge	m/z	Mass	Mass error [Da]	Mass error [ppm]	Retention time	PEP	Score	Precursor Intensity
sll1577	2	728.8765	1455.738	−0.00049113	−0.68897	20.754	6.3544e−94	249.86	34302640

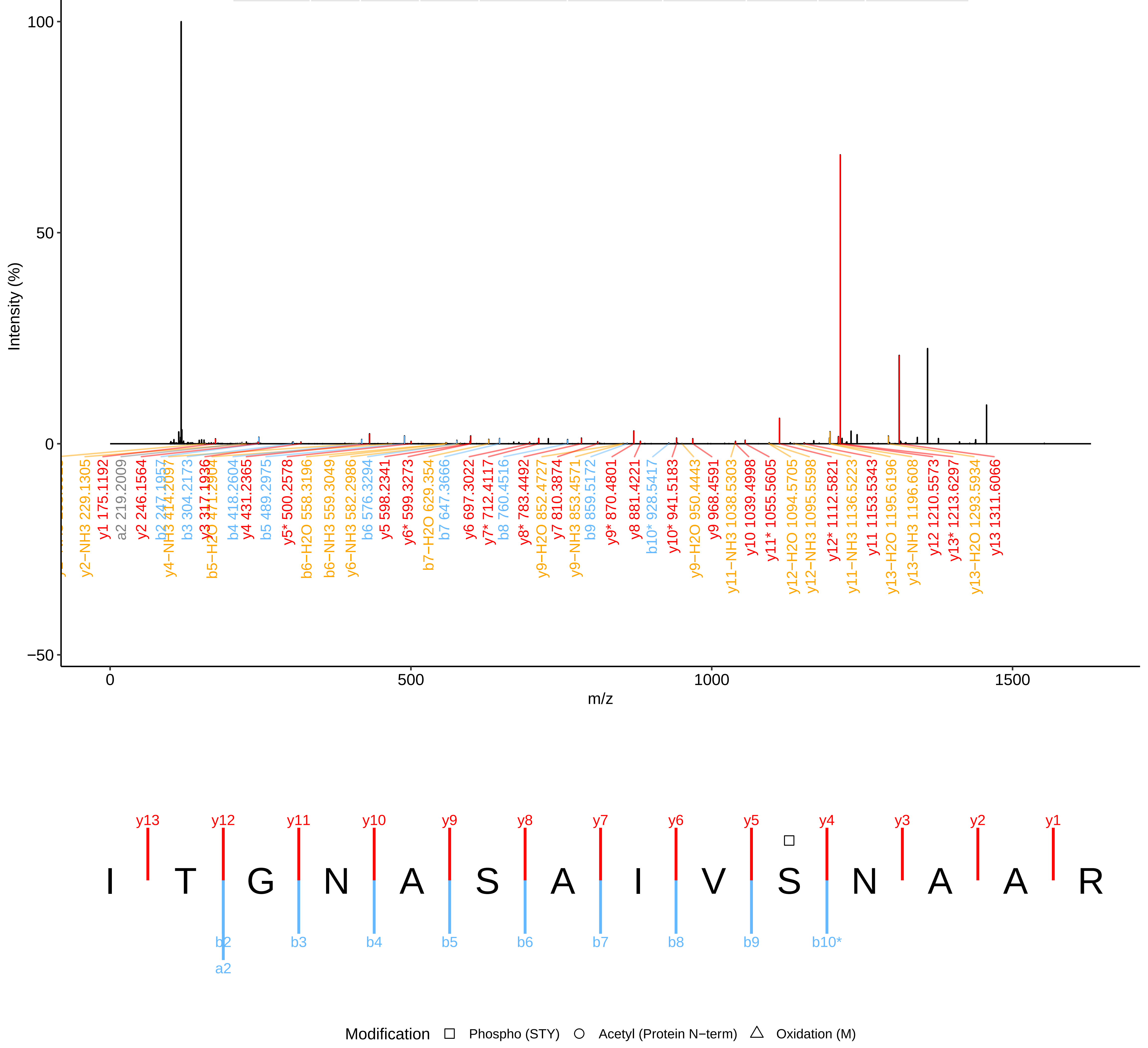

20211217_PS_SCy031_Proteome_R3_3hLC_F7 − Scan number: 31068

Gene Names	Charge	m/z	Mass	Mass error [Da]	Mass error [ppm]	Retention time	PEP	Score	Precursor Intensity
sll1577	3	1101.825	3302.453	NA	NA	47.675	0.002066	57.945	5795104

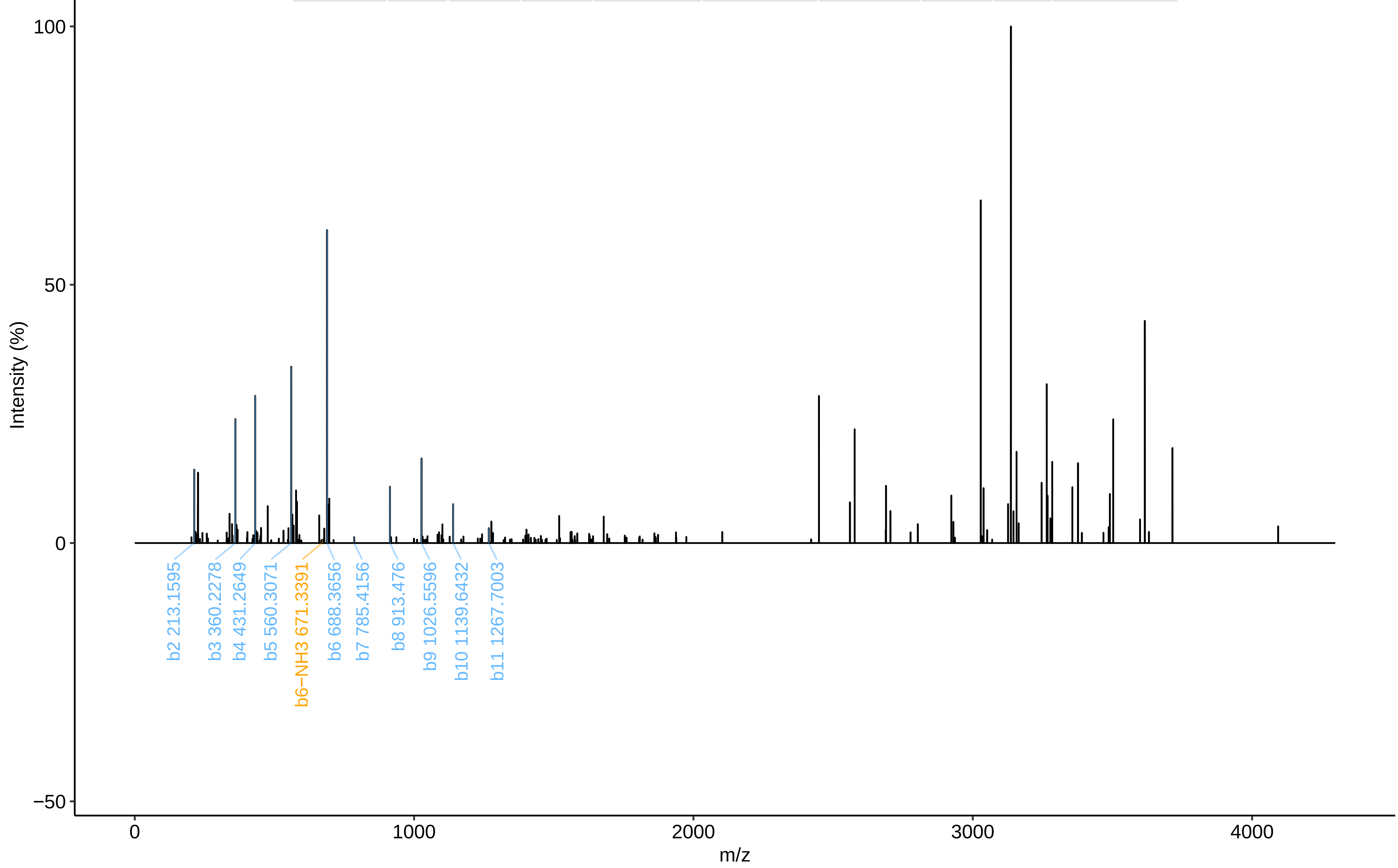

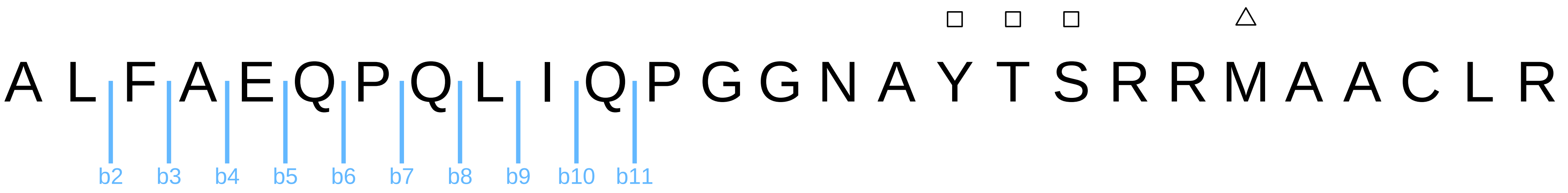

Modification □ Phospho (STY) ○ Acetyl (Protein N−term) △ Oxidation (M)

20211212_PS_SCy031_proteome_R1_HC_F9 − Scan number: 25354

Gene Names	Charge	m/z	Mass	Mass error [Da]	Mass error [ppm]	Retention time	PEP	Score	Precursor Intensity
sll1577	3	894.8256	2681.455	0.0037108	4.1454	40.638	0.00026019	78.236	1065477

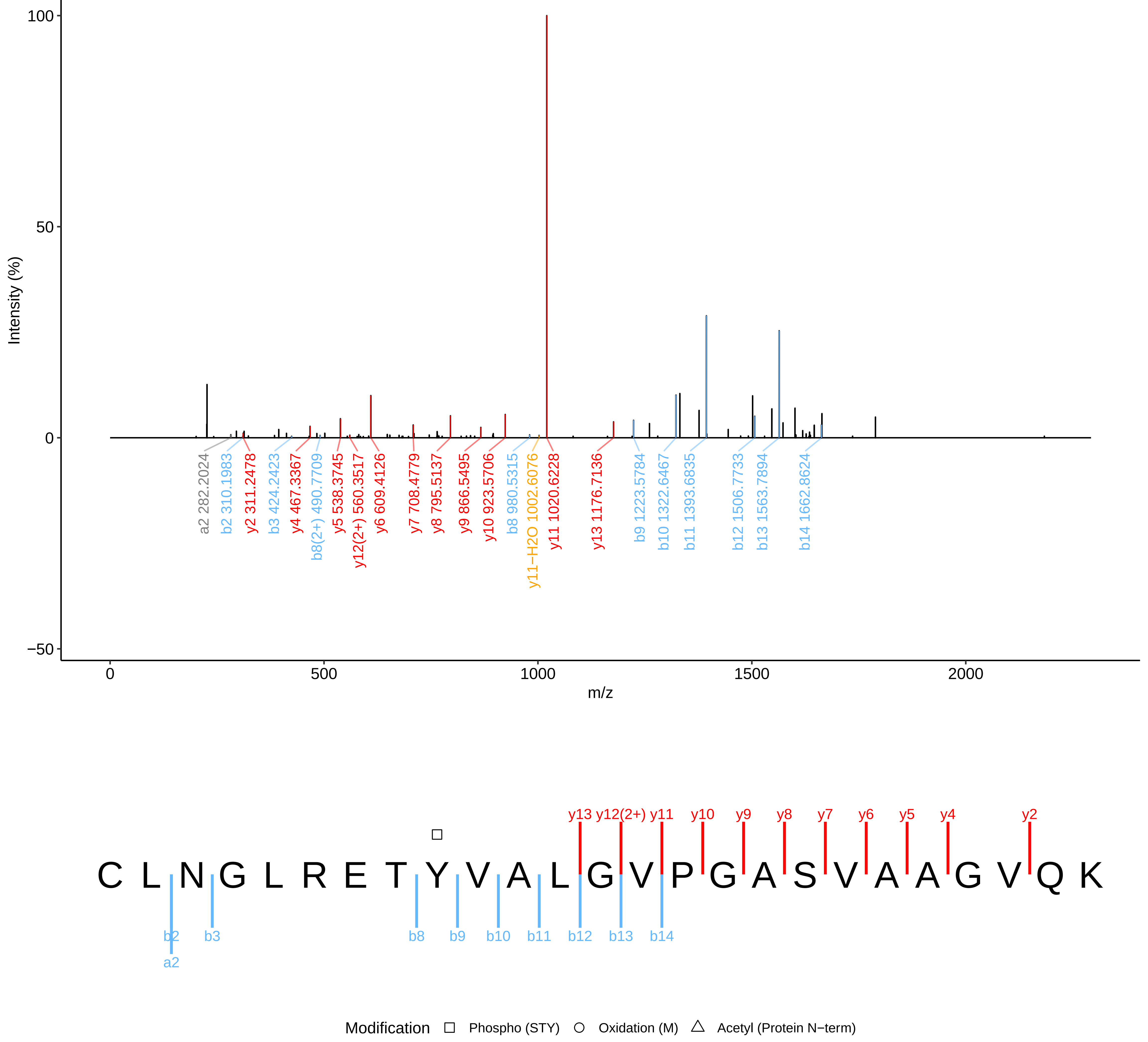

20210828_PS_SCy031_phospho_R2_TiO2_LC24h_F2 − Scan number: 14834

Gene Names	Charge	m/z	Mass	Mass error [Da]	Mass error [ppm]	Retention time	PEP	Score	Precursor Intensity
sll1578	2	864.8854	1727.756	NA	NA	21.63	0.0029391	84.508	23802538

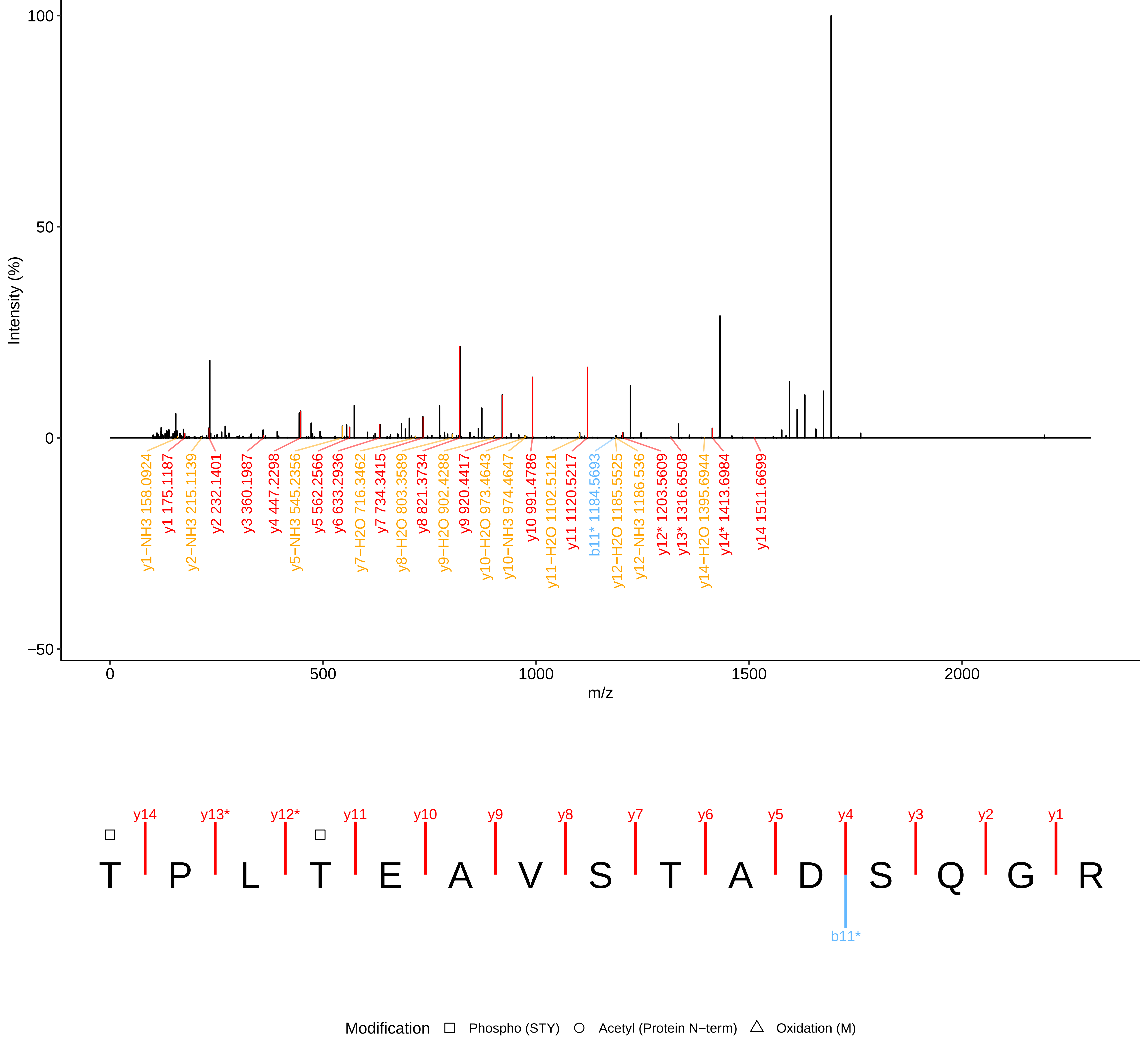

20210828_PS_SCy031_phospho_R3_TiO2_LC3h_F3 − Scan number: 17257

Gene Names	Charge	m/z	Mass	Mass error [Da]	Mass error [ppm]	Retention time	PEP	Score	Precursor Intensity
sll1578	3	680.6613	2038.962	0.0025038	3.6785	24.79	0.0031767	62.765	873733.5

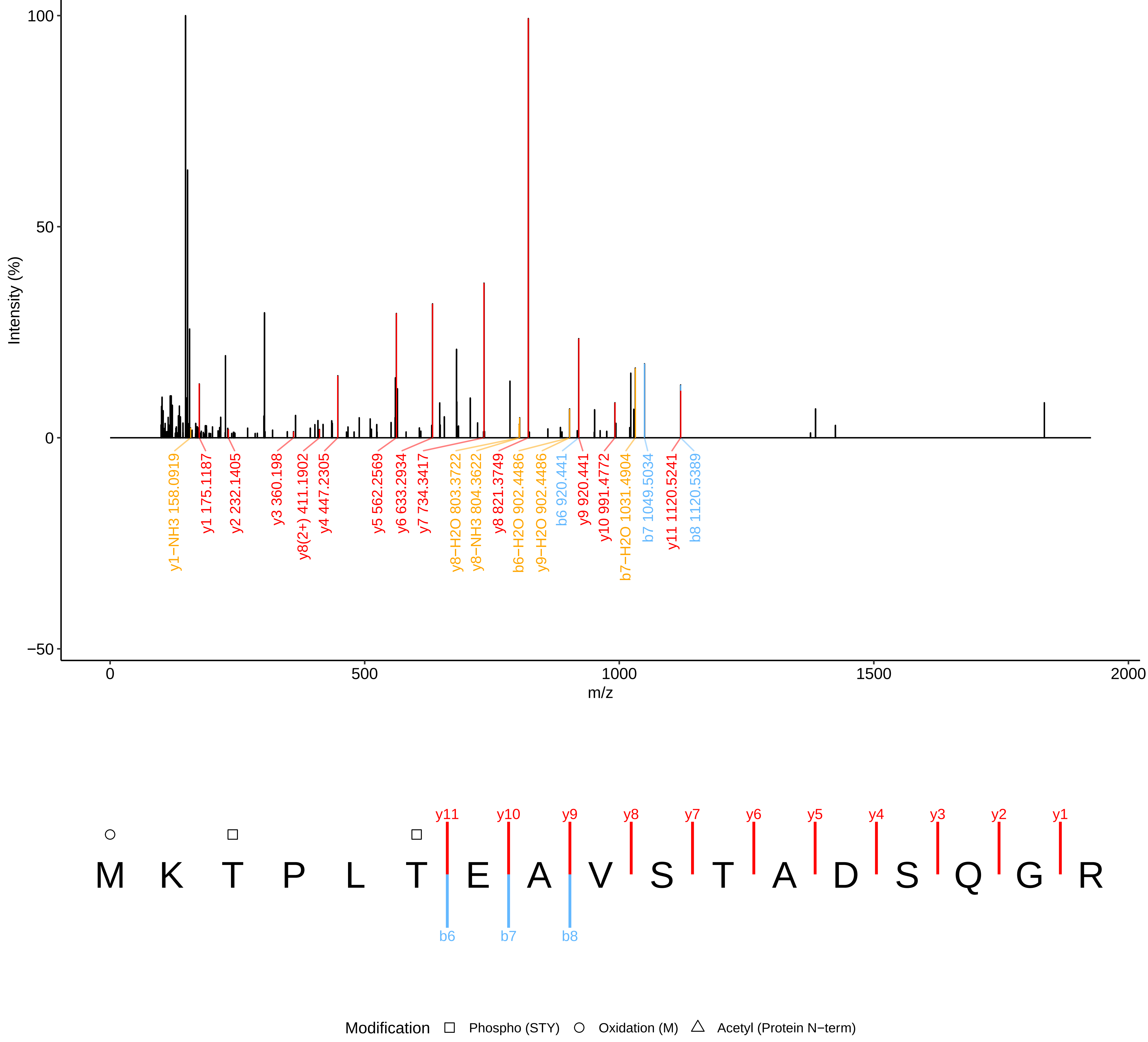

20210828_PS_SCy031_phospho_R3_TiO2_LC24h_F2 − Scan number: 14706

Gene Names	Charge	m/z	Mass	Mass error [Da]	Mass error [ppm]	Retention time	PEP	Score	Precursor Intensity
sll1578	3	550.2705	1647.79	−0.00014083	−0.26165	21.488	1.2958e−34	195.17	8032746

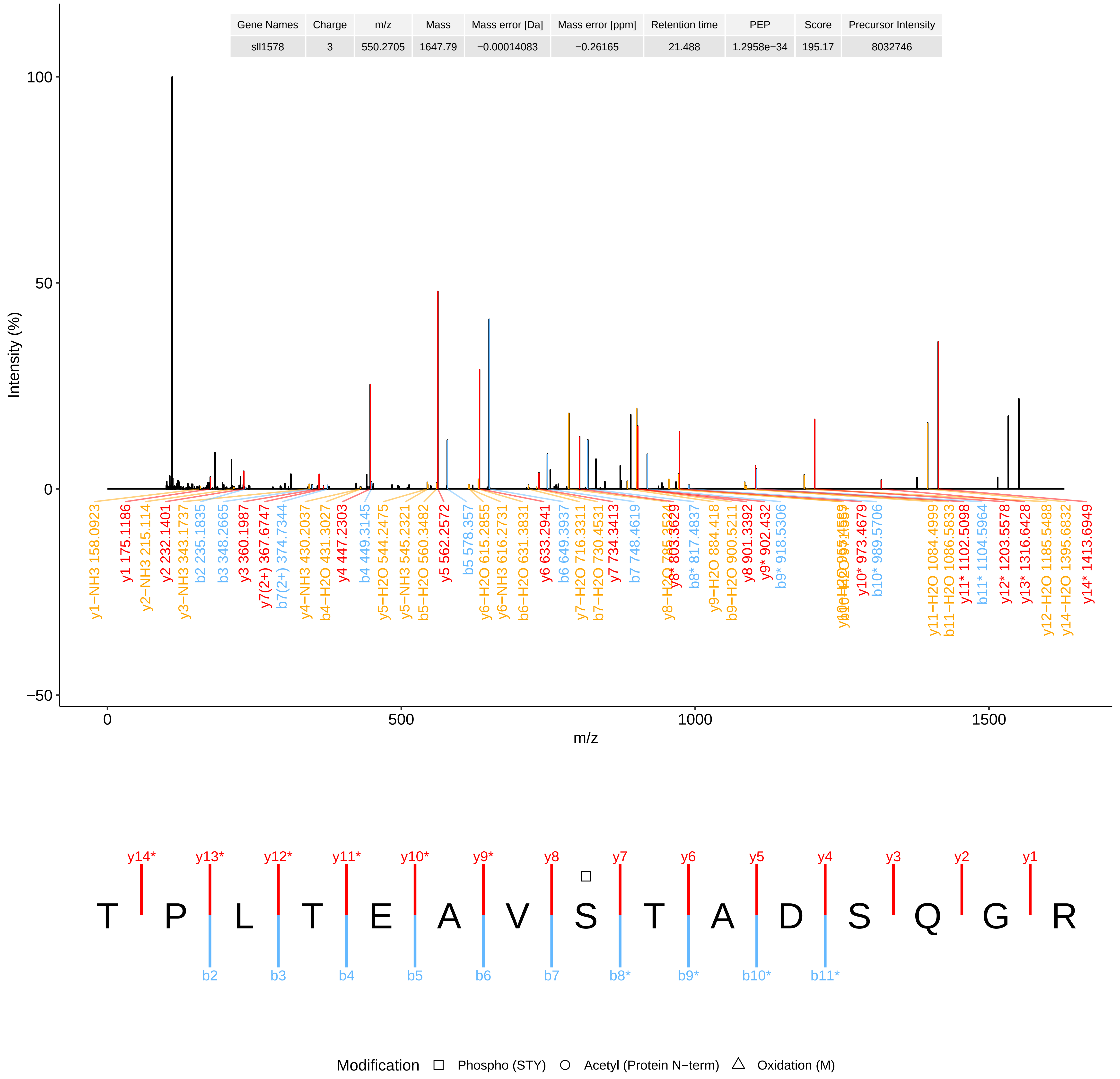

20210828_PS_SCy031_phospho_R3_TiO2_LC3h_F2 − Scan number: 14418

Gene Names	Charge	m/z	Mass	Mass error [Da]	Mass error [ppm]	Retention time	PEP	Score	Precursor Intensity
sll1578	3	548.9308	1643.771	−3.2403e−06	−0.0059029	21.09	3.2519e−11	149.74	6876707

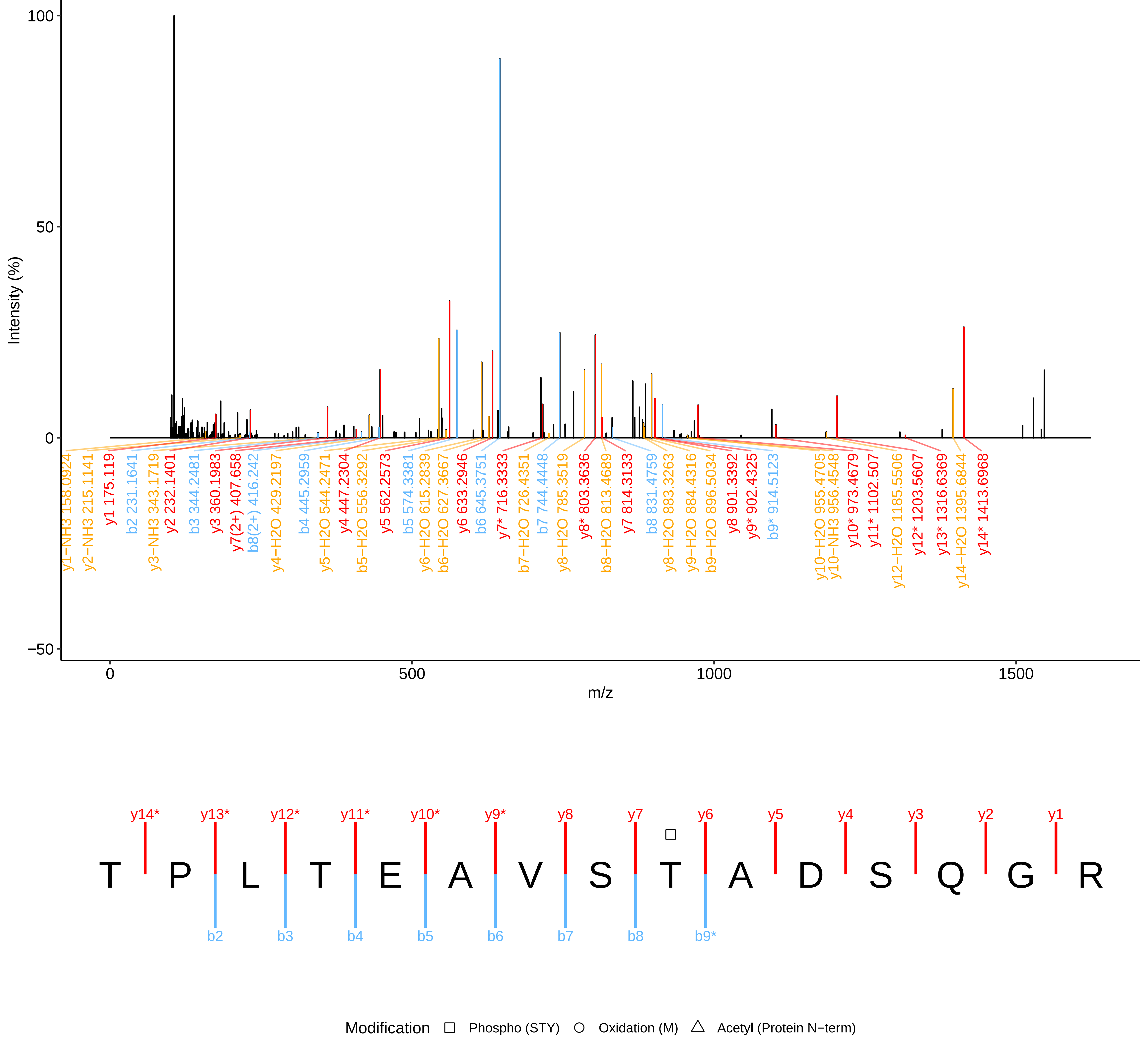

20210828_PS_SCy031_phospho_R2_TiO2_LC24h_F1 − Scan number: 14975

Gene Names	Charge	m/z	Mass	Mass error [Da]	Mass error [ppm]	Retention time	PEP	Score	Precursor Intensity
sll1578	3	547.5891	1639.745	−0.00017476	−0.32468	21.803	0.003909	52.614	NA

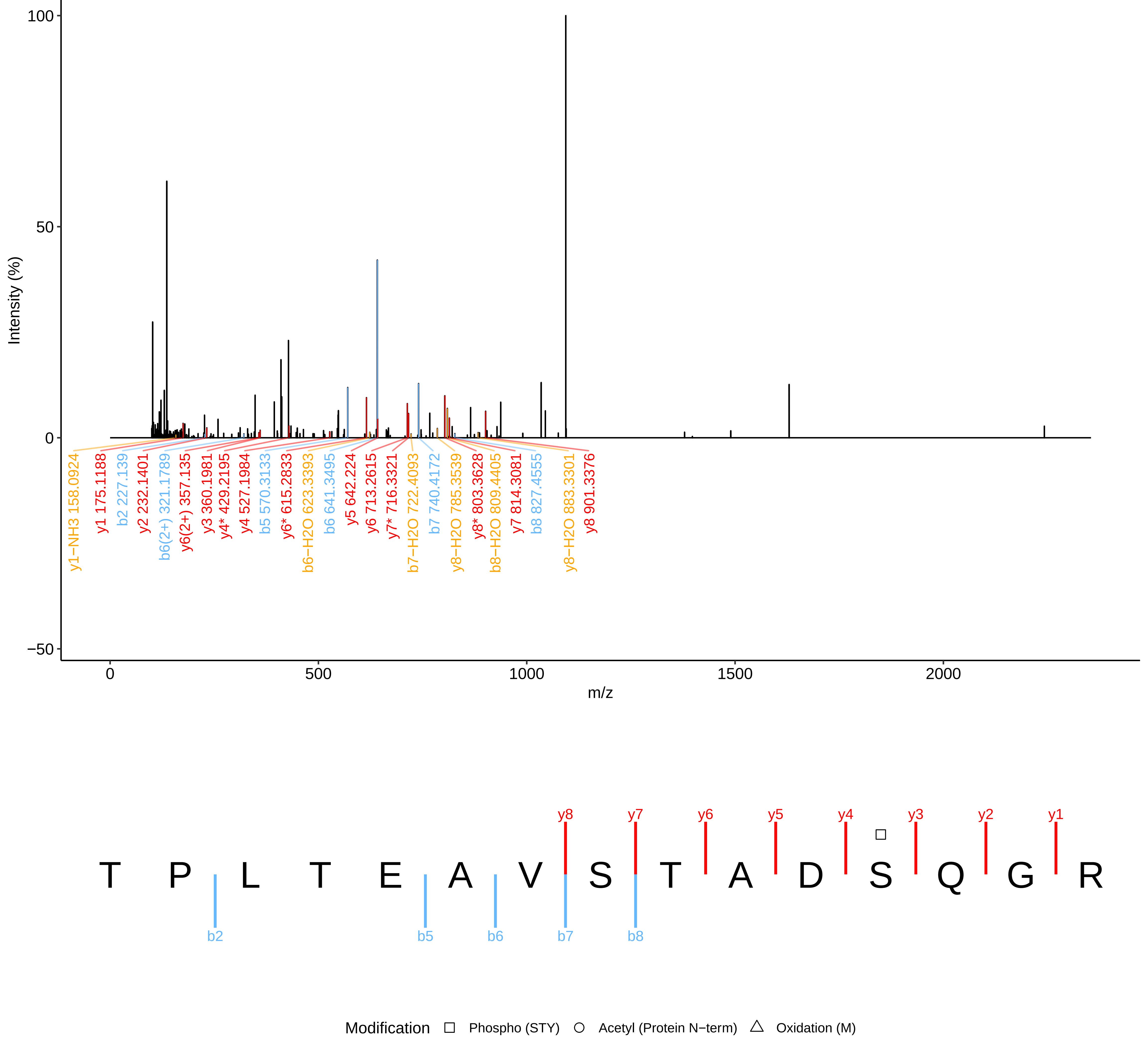

20210828_PS_SCy031_phospho_R3_TiO2_HC_F2 − Scan number: 35324

Gene Names	Charge	m/z	Mass	Mass error [Da]	Mass error [ppm]	Retention time	PEP	Score	Precursor Intensity
sll1578	2	790.9047	1579.795	0.0011473	1.4506	47.418	1.1248e−13	182.33	2411581

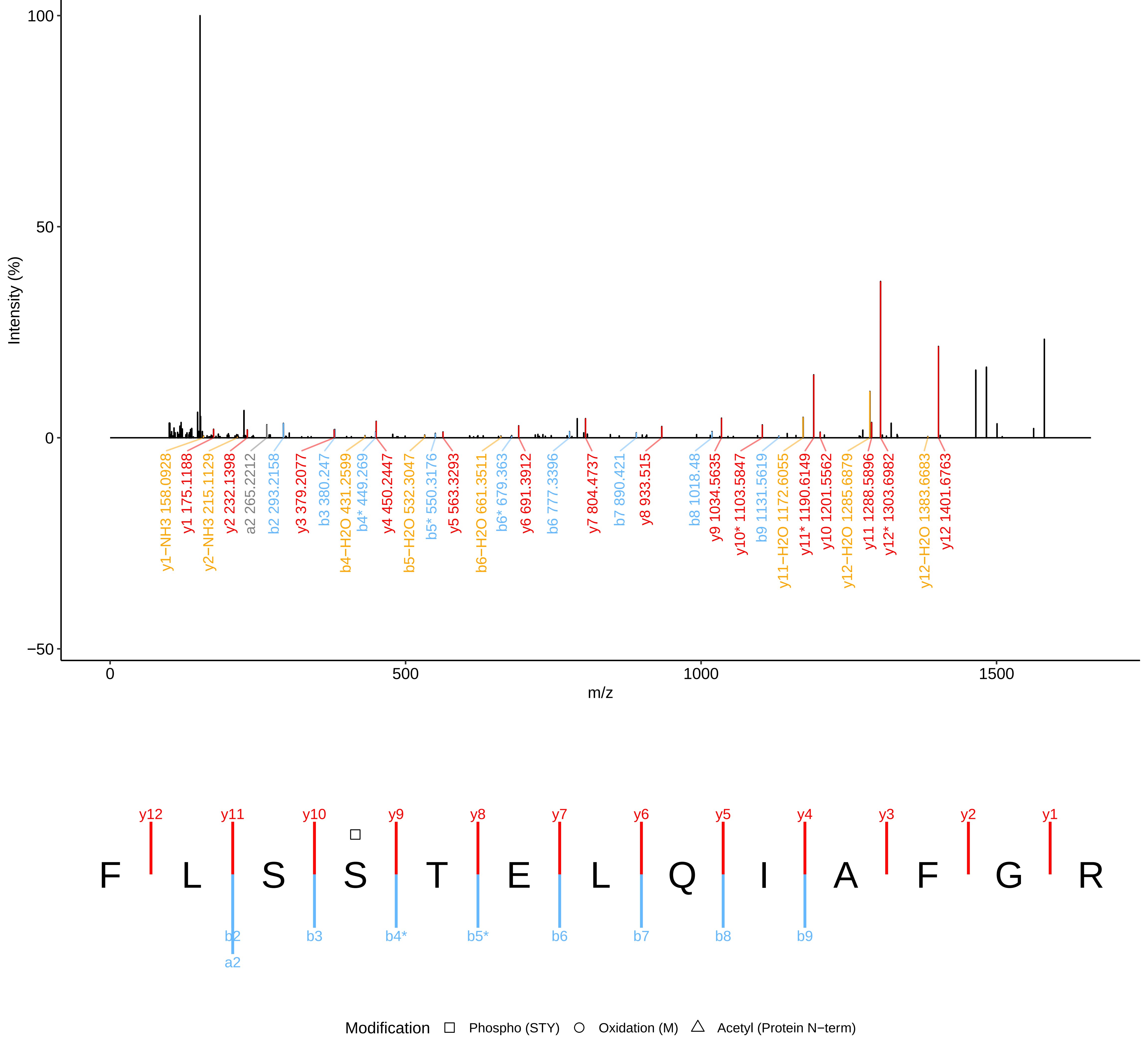

20210828_PS_SCy031_phospho_R3_TiO2_LC3h_F3 − Scan number: 30375

Gene Names	Charge	m/z	Mass	Mass error [Da]	Mass error [ppm]	Retention time	PEP	Score	Precursor Intensity
sll1578	2	1092.539	2183.062	−0.00084292	−0.79184	41.396	0	419.43	5293132

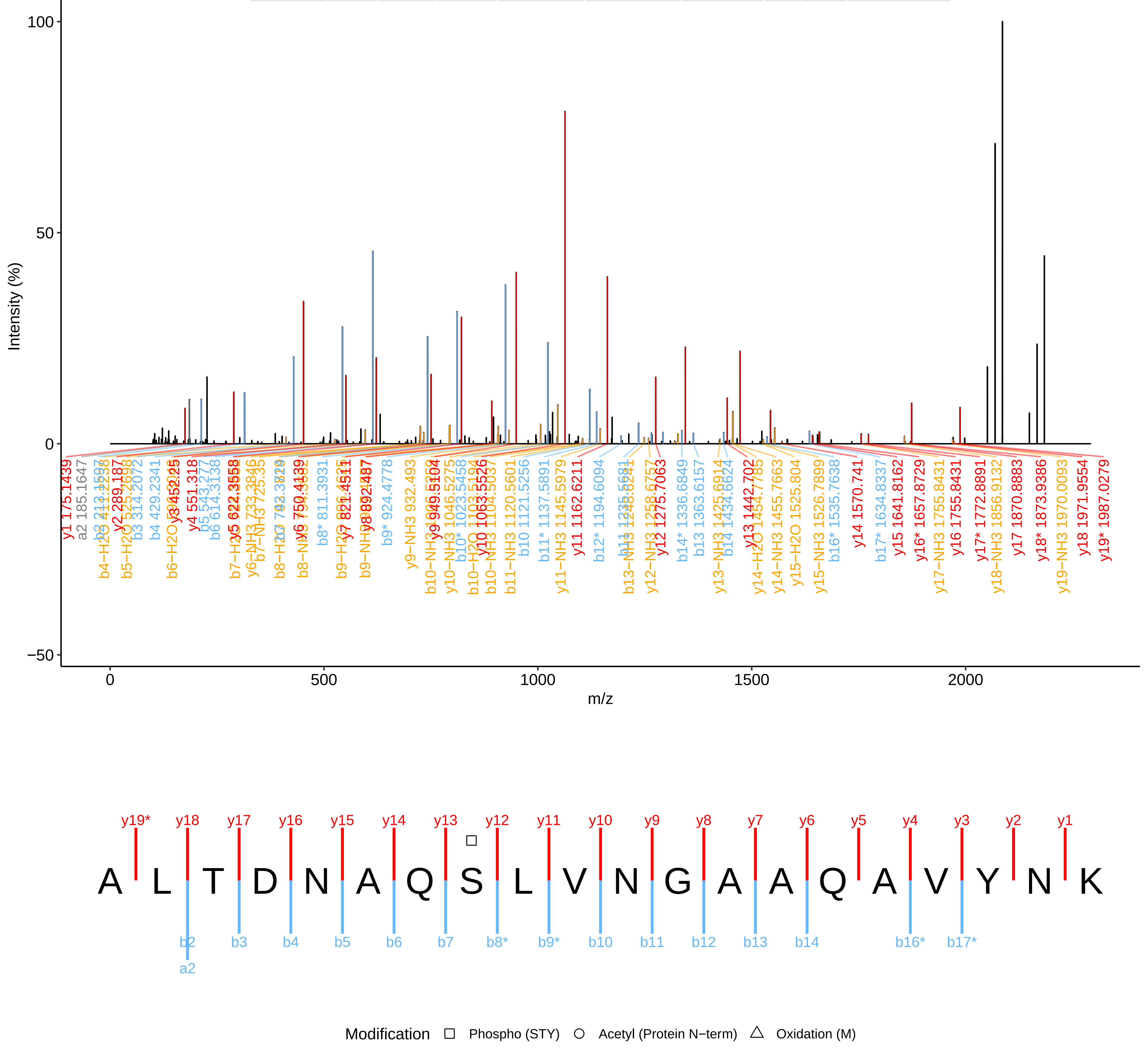

20210828_PS_SCy031_phospho_R3_TiO2_LC24h_F2 − Scan number: 36010

Gene Names	Charge	m/z	Mass	Mass error [Da]	Mass error [ppm]	Retention time	PEP	Score	Precursor Intensity
sll1578	4	1033.756	4130.994	−5.8154e−05	−0.056229	48.362	9.9247e−20	104.57	18443402

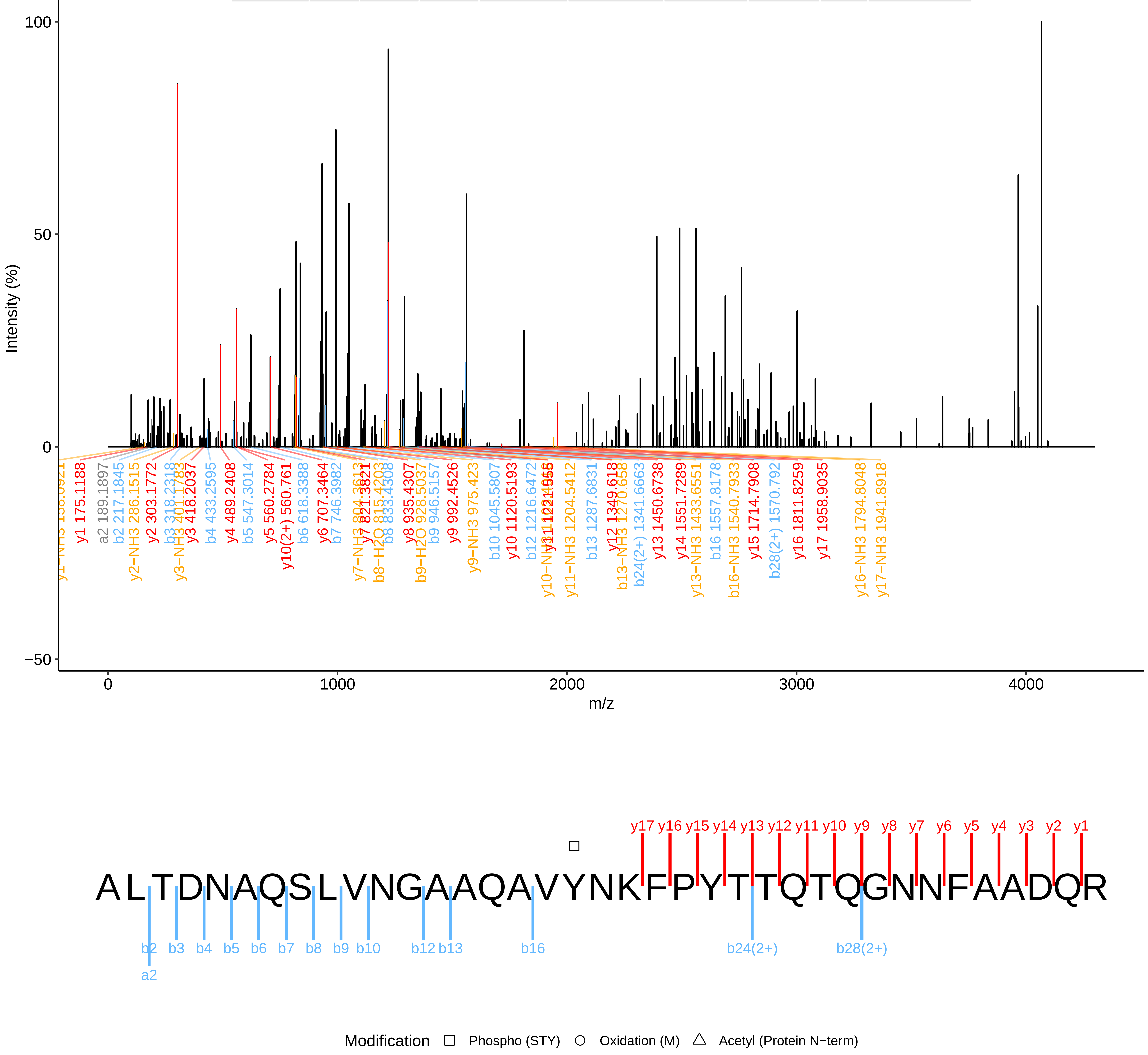

20210823_PS_SCy031_R1_phospho_LC3h_TiO2_2 − Scan number: 15009

Gene Names	Charge	m/z	Mass	Mass error [Da]	Mass error [ppm]	Retention time	PEP	Score	Precursor Intensity
sll1578	3	763.3697	2287.087	0.0006799	0.89027	21.861	5.1611e−24	175.42	29390754

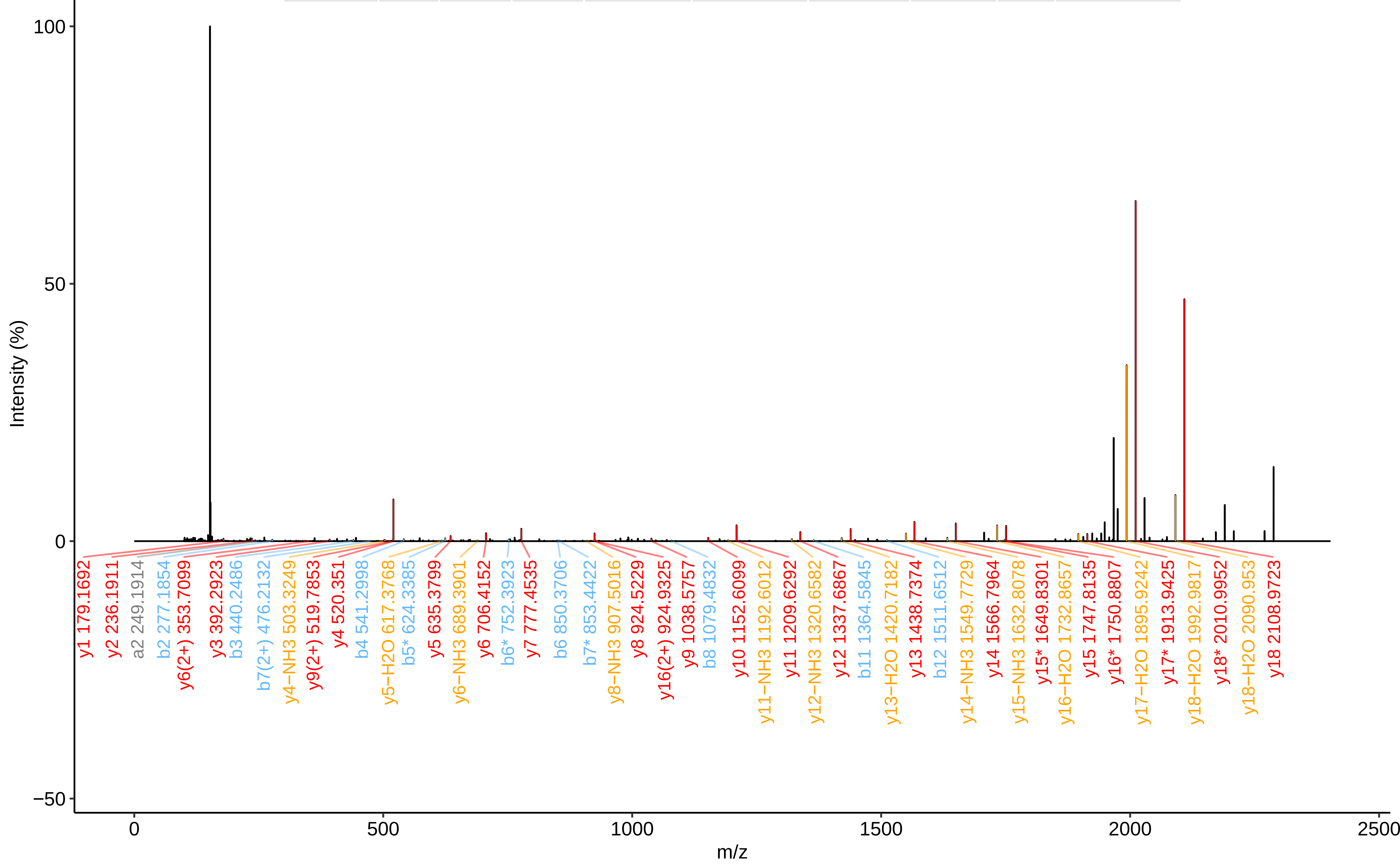

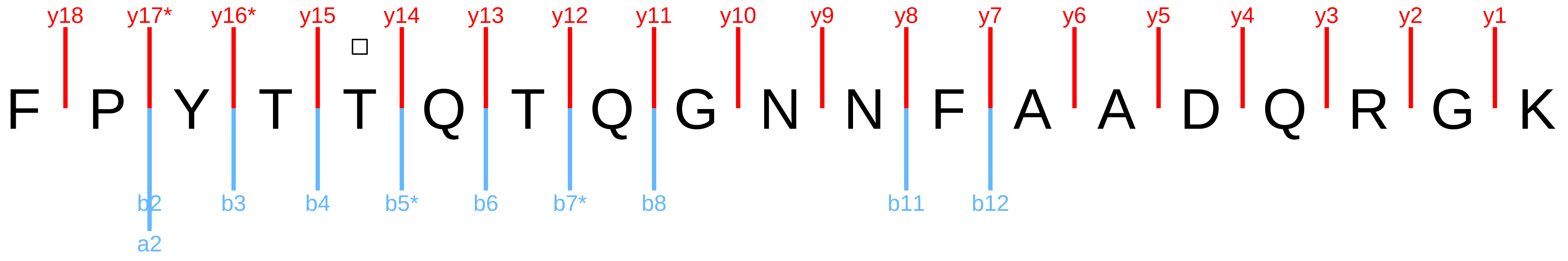

Modification □ Phospho (STY) ○ Oxidation (M) △ Acetyl (Protein N−term)

20210828_PS_SCy031_phospho_R2_TiO2_LC24h_F2 − Scan number: 19063

Gene Names	Charge	m/z	Mass	Mass error [Da]	Mass error [ppm]	Retention time	PEP	Score	Precursor Intensity
sll1578	3	689.6371	2065.89	−0.00020154	−0.29626	26.951	1.5252e−71	232.49	56032664

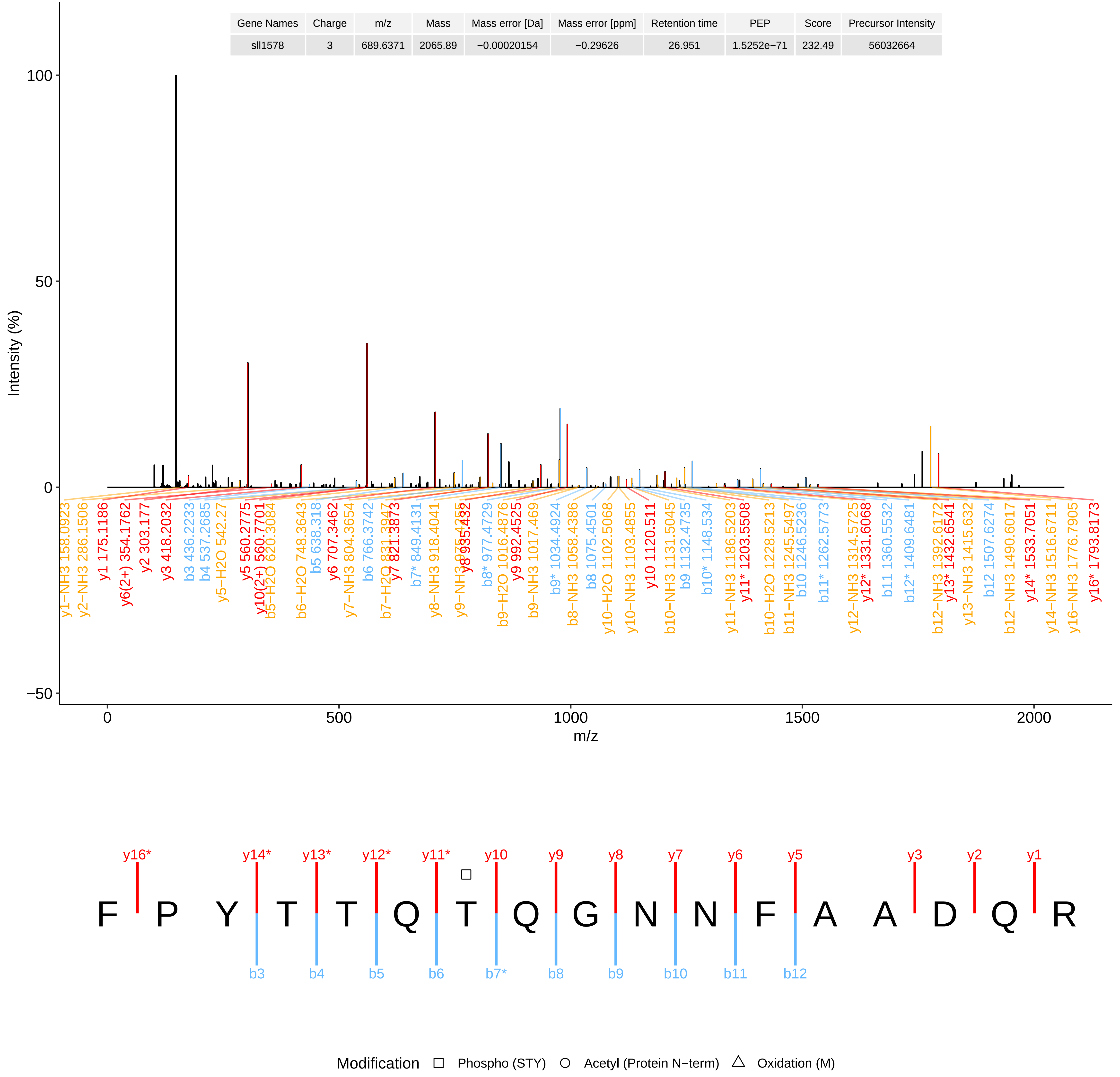

20211217_PS_SCy031_Proteome_R2_HC_F6 − Scan number: 30395

Gene Names	Charge	m/z	Mass	Mass error [Da]	Mass error [ppm]	Retention time	PEP	Score	Precursor Intensity
sll1578	2	900.459	1798.903	NA	NA	47.179	3.1162e−08	148.32	668870.5

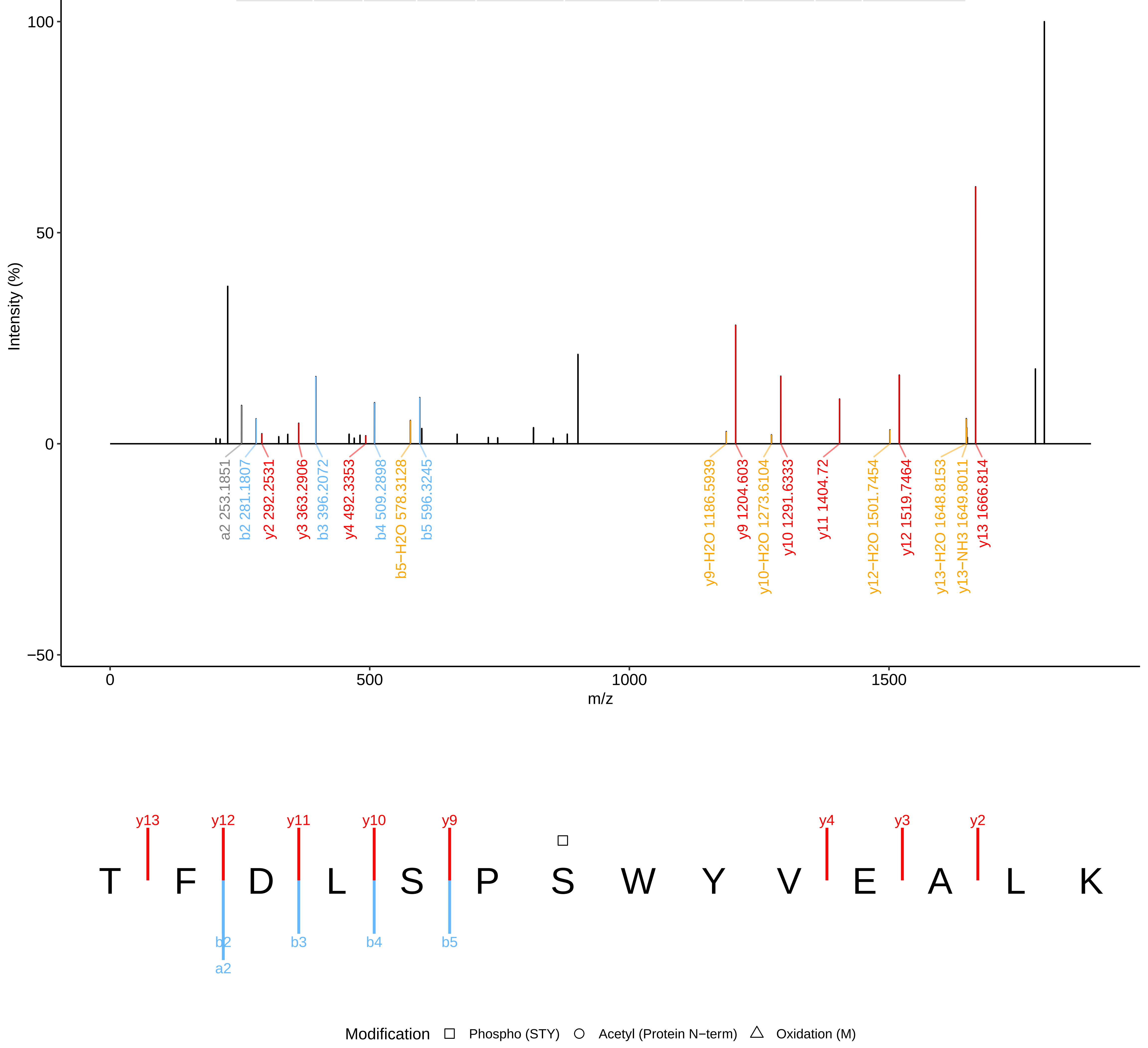

20210828_PS_SCy031_phospho_R2_TiO2_LC24h_F1 − Scan number: 3826

Gene Names	Charge	m/z	Mass	Mass error [Da]	Mass error [ppm]	Retention time	PEP	Score	Precursor Intensity
sll1578	2	555.2555	1108.496	0.00012614	0.22718	7.7908	0.11073	78.287	7987310

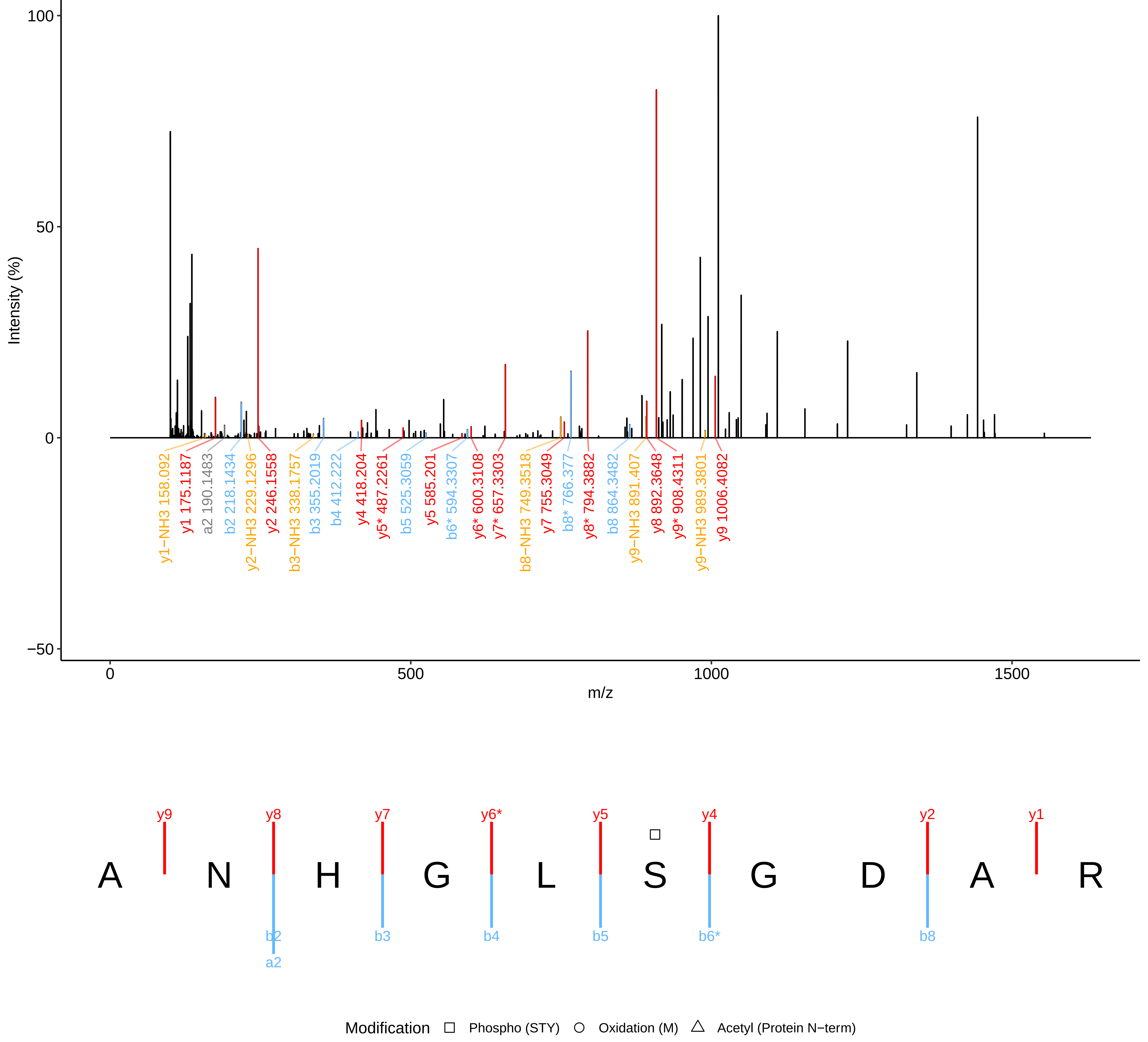

20210828_PS_SCy031_phospho_R3_TiO2_LC3h_F1 − Scan number: 32088

Gene Names	Charge	m/z	Mass	Mass error [Da]	Mass error [ppm]	Retention time	PEP	Score	Precursor Intensity
sll1672	3	652.0288	1953.065	−0.00031046	−0.47591	43.534	3.5813e−05	96.708	941216.7

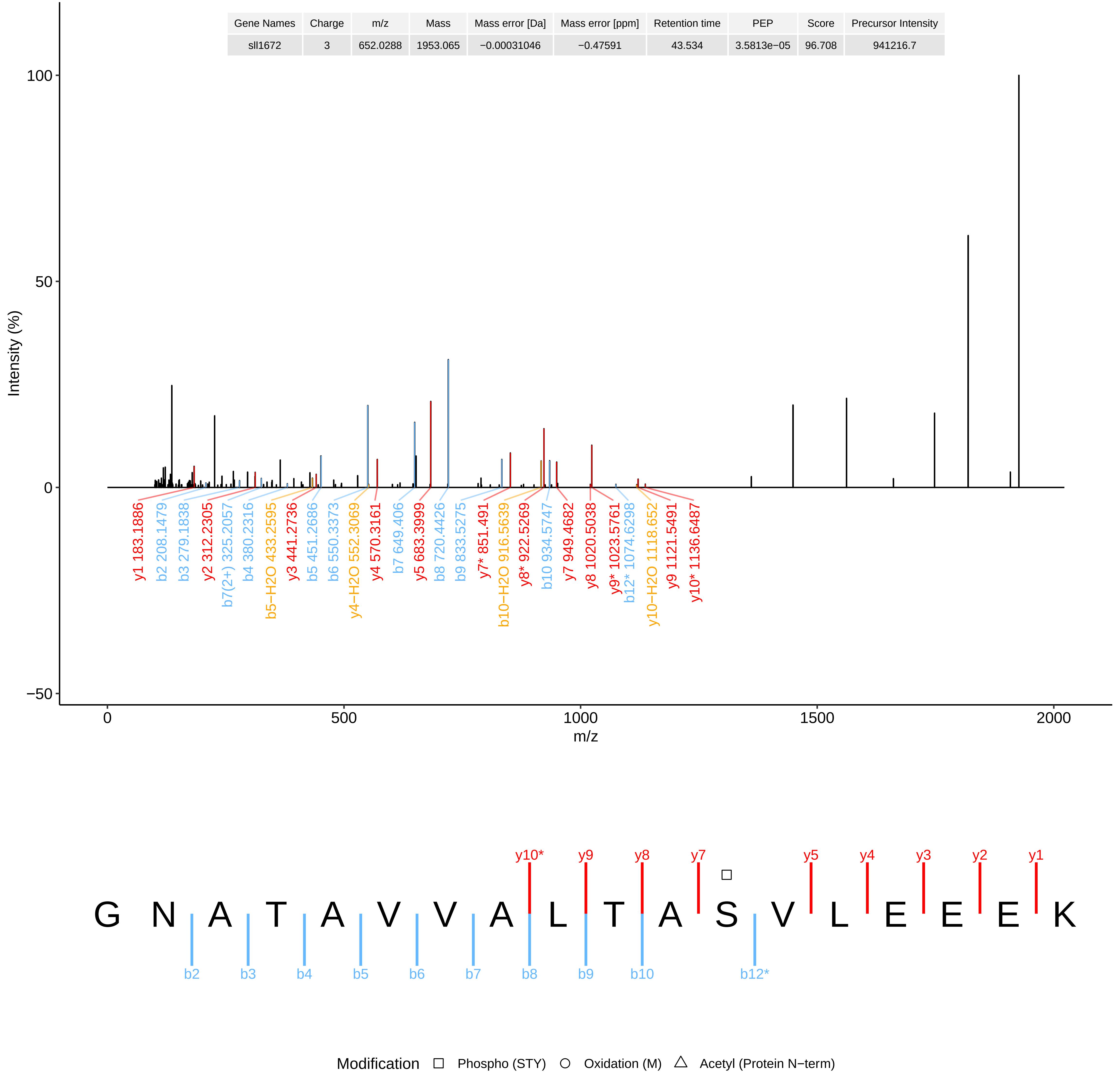

20210828_PS_SCy031_phospho_R2_TiO2_LC3h_F1 − Scan number: 12502

Gene Names	Charge	m/z	Mass	Mass error [Da]	Mass error [ppm]	Retention time	PEP	Score	Precursor Intensity
sll1742	2	855.3971	1708.78	NA	NA	18.669	0.0029309	103.39	NA

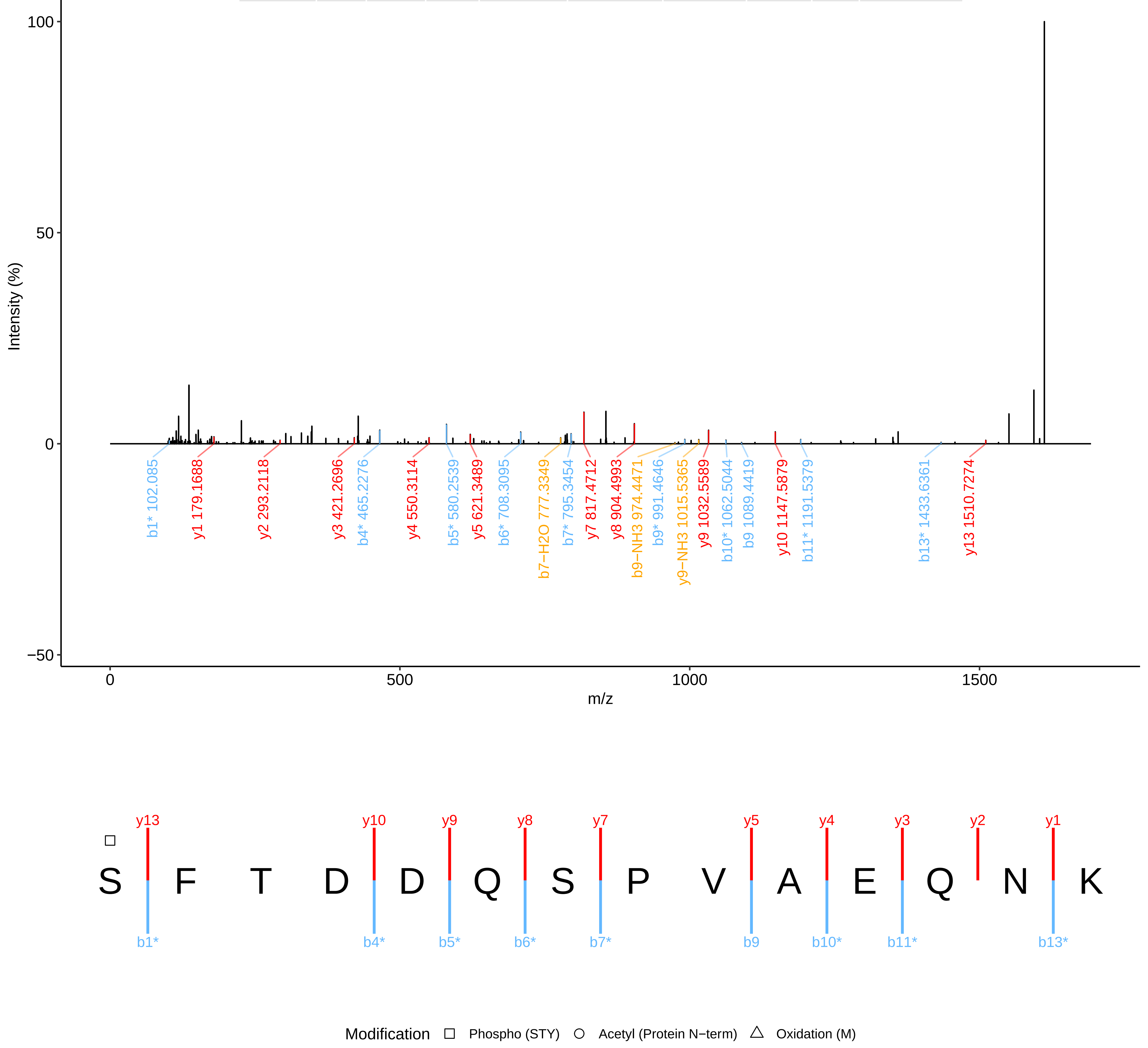

20210823_PS_SCy031_R1_phospho_LC3h_TiO2_2 − Scan number: 27841

Gene Names	Charge	m/z	Mass	Mass error [Da]	Mass error [ppm]	Retention time	PEP	Score	Precursor Intensity
sll1746	2	730.896	1459.777	−0.0015806	−2.1626	37.988	0.010588	136.83	9299892

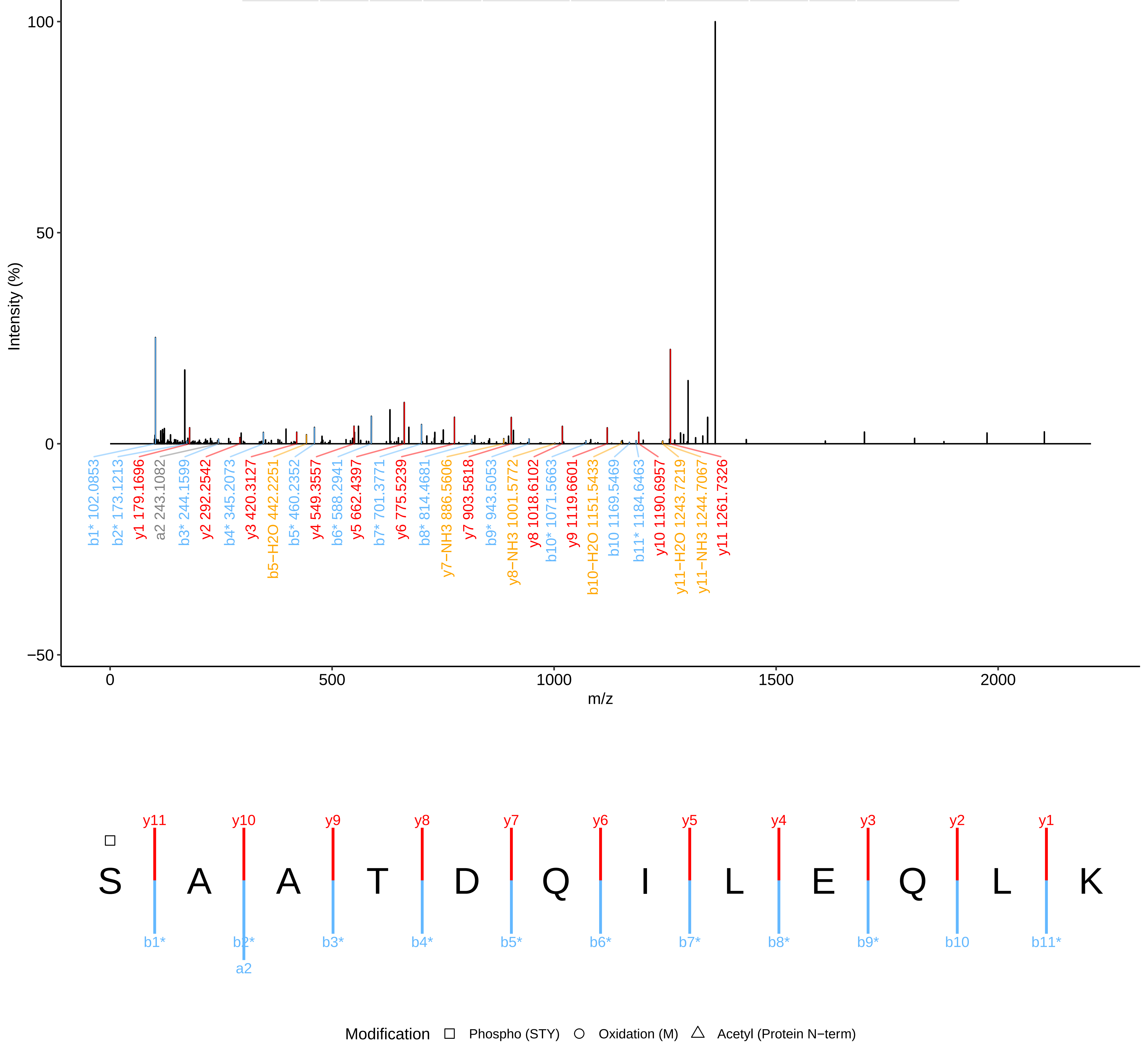

20210828_PS_SCy031_phospho_R3_TiO2_LC3h_F2 − Scan number: 20399

Gene Names	Charge	m/z	Mass	Mass error [Da]	Mass error [ppm]	Retention time	PEP	Score	Precursor Intensity
sll1758	3	782.3784	2344.113	NA	NA	28.645	1.1016e−22	158.1	4476141

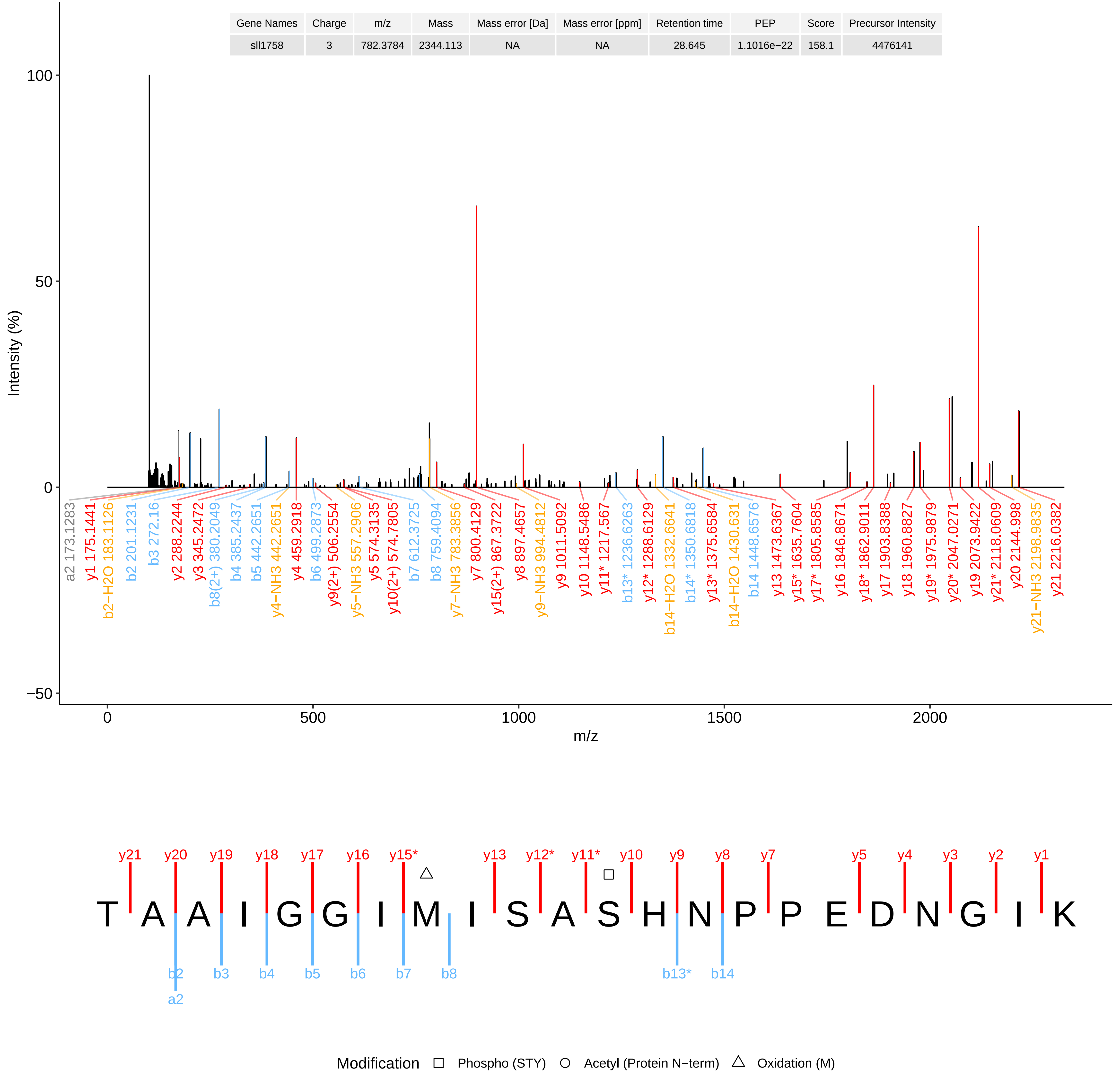

20210828_PS_SCy031_phospho_R2_TiO2_LC24h_F3 − Scan number: 13629

Gene Names	Charge	m/z	Mass	Mass error [Da]	Mass error [ppm]	Retention time	PEP	Score	Precursor Intensity
sll1812	2	843.9367	1685.859	−0.00066222	−0.78468	20.171	0.010455	120.31	8166826

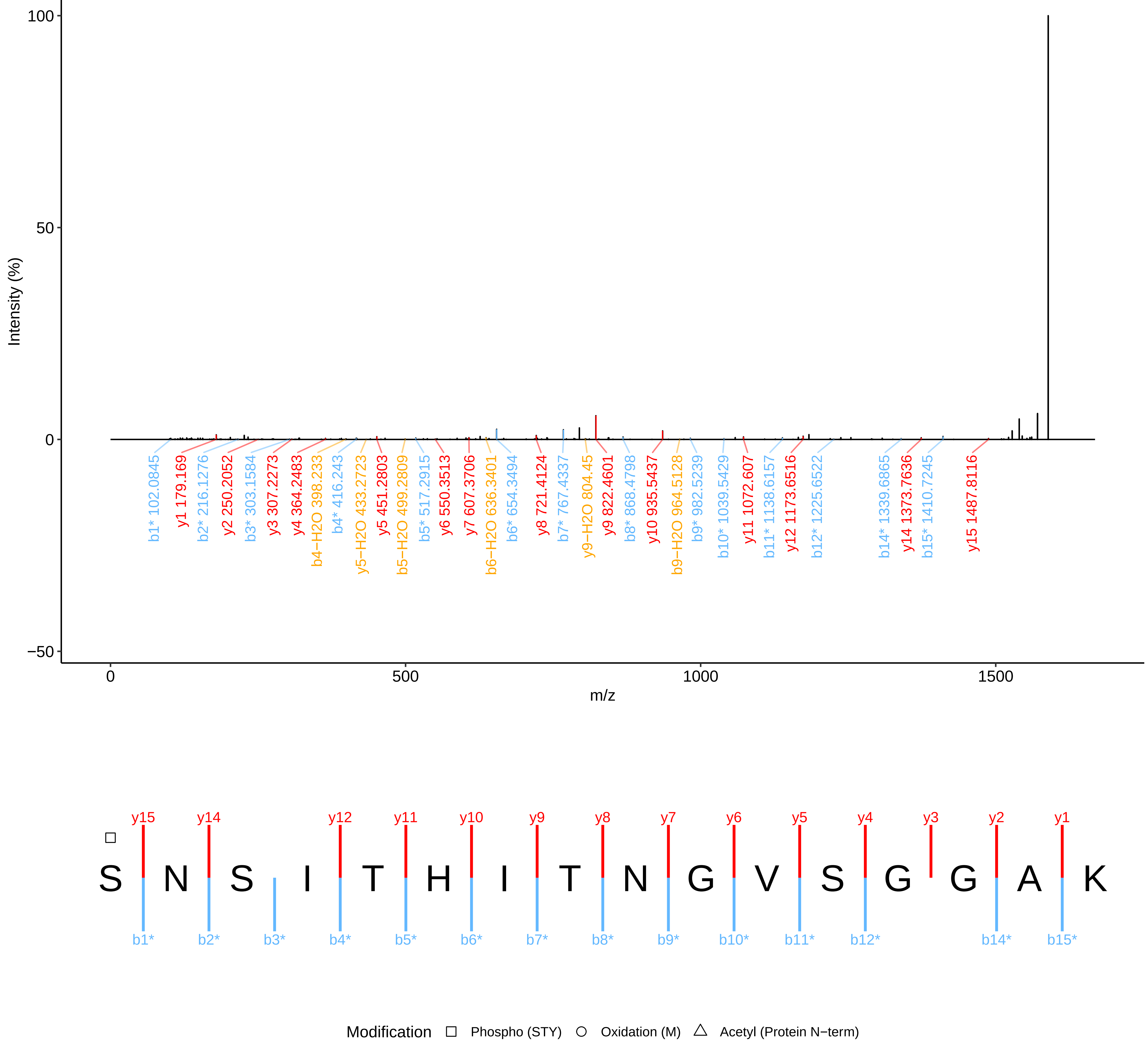

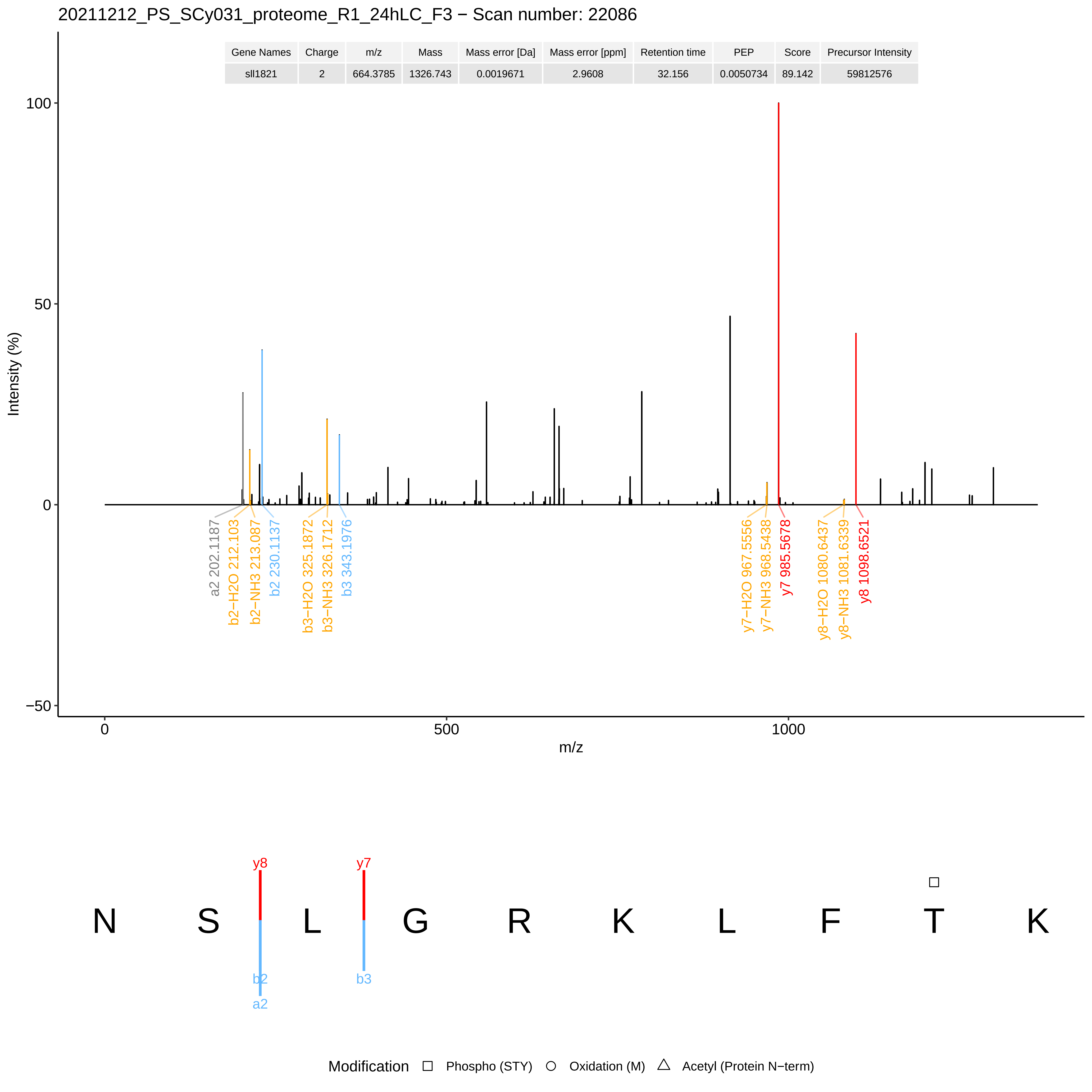
20211212_PS_SCy031_proteome_R1_24hLC_F3 − Scan number: 22086
Gene Names | Charge | m/z | Mass | Mass error [Da] | Mass error [ppm] | Retention time | PEP | Score | Precursor Intensity
sll1821 | 2 | 664.3785 | 1326.743 | 0.0019671 | 2.9608 | 32.156 | 0.0050734 | 89.142 | 59812576
Intensity (%)
100
50
0
−50
0
500
1000
m/z
a2 202.1187
b2−H2O 212.103
b2−NH3 213.087
b2 230.1137
b3−H2O 325.1872
b3−NH3 326.1712
b3 343.1976
y7−H2O 967.5556
y7−NH3 968.5438
y7 985.5678
y8−H2O 1080.6437
y8−NH3 1081.6339
y8 1098.6521
y8
y7
N S L G R K L F T K
b2
a2
b3
Modification
Phospho (STY)
Oxidation (M)
Acetyl (Protein N−term)

20211217_PS_SCy031_Proteome_R2_HC_F2 − Scan number: 37940

Gene Names	Charge	m/z	Mass	Mass error [Da]	Mass error [ppm]	Retention time	PEP	Score	Precursor Intensity
sll1865	2	659.8532	1317.692	NA	NA	53.669	0.012899	68.915	7247495

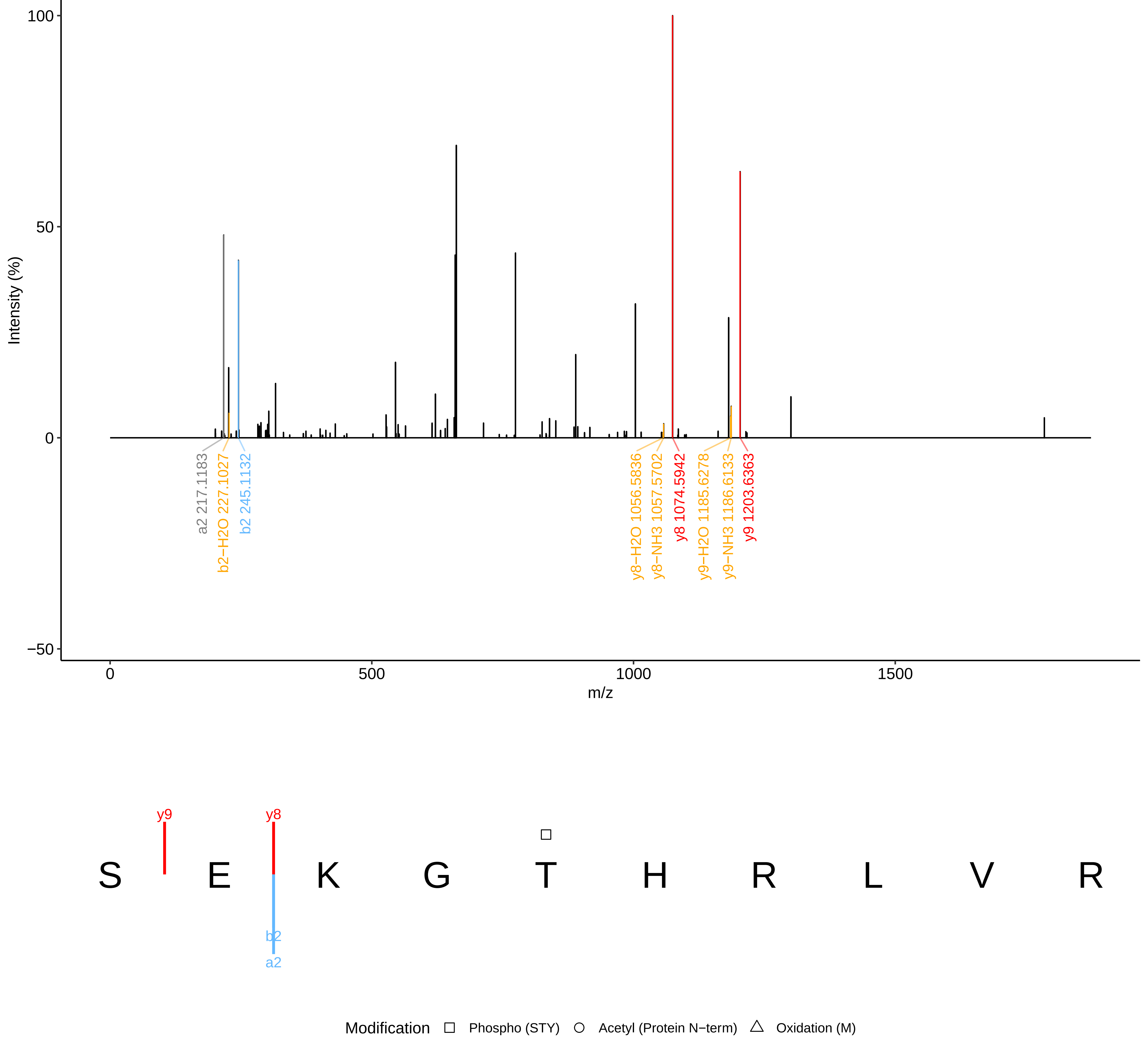

20210828_PS_SCy031_phospho_R3_TiO2_LC24h_F2 − Scan number: 7803

Gene Names	Charge	m/z	Mass	Mass error [Da]	Mass error [ppm]	Retention time	PEP	Score	Precursor Intensity
sll1921	2	661.3467	1320.679	0.0014338	2.168	12.827	0.018633	106.17	7959618

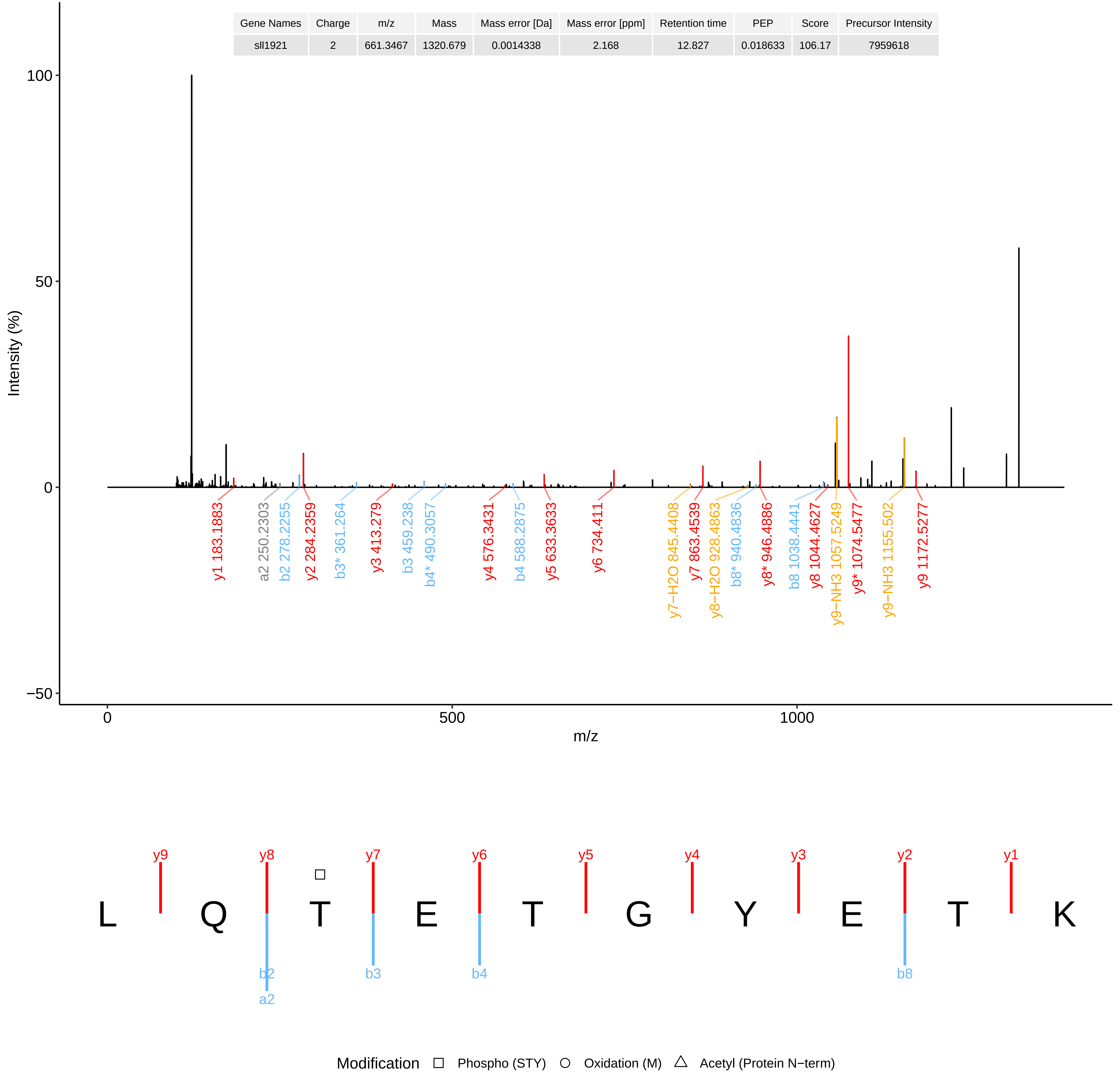

20210823_PS_SCy031_R1_phospho_LC3h_TiO2_2 − Scan number: 28886

Gene Names	Charge	m/z	Mass	Mass error [Da]	Mass error [ppm]	Retention time	PEP	Score	Precursor Intensity
sll1921	2	788.4227	1574.831	0.0004084	0.53995	39.299	1.5925e−112	266.75	23097612

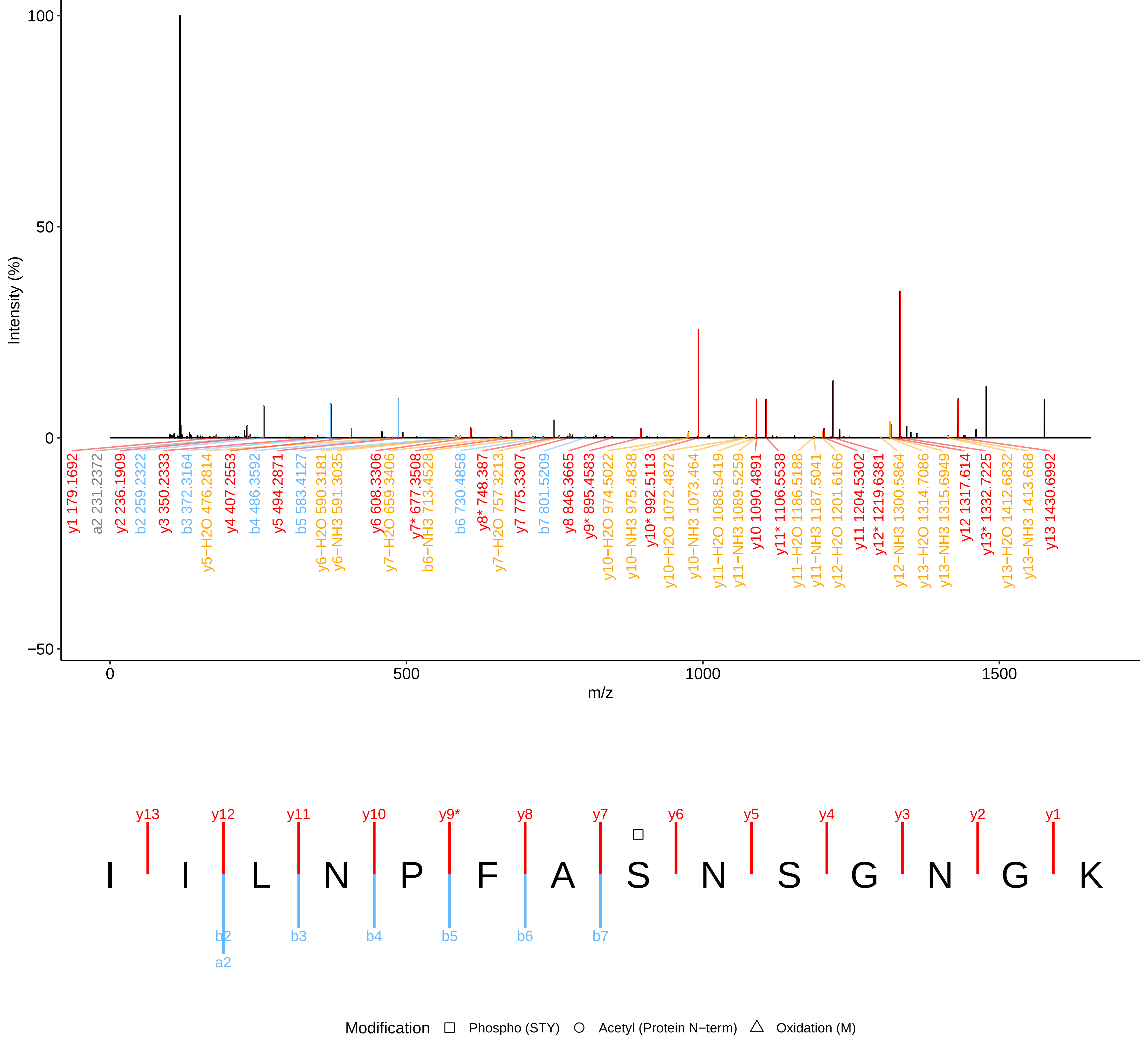

20210828_PS_SCy031_phospho_R3_TiO2_LC24h_F1 − Scan number: 13175

Gene Names	Charge	m/z	Mass	Mass error [Da]	Mass error [ppm]	Retention time	PEP	Score	Precursor Intensity
sll2012	2	780.389	1558.763	0.0012179	1.5596	19.323	0.033495	63.816	5813111

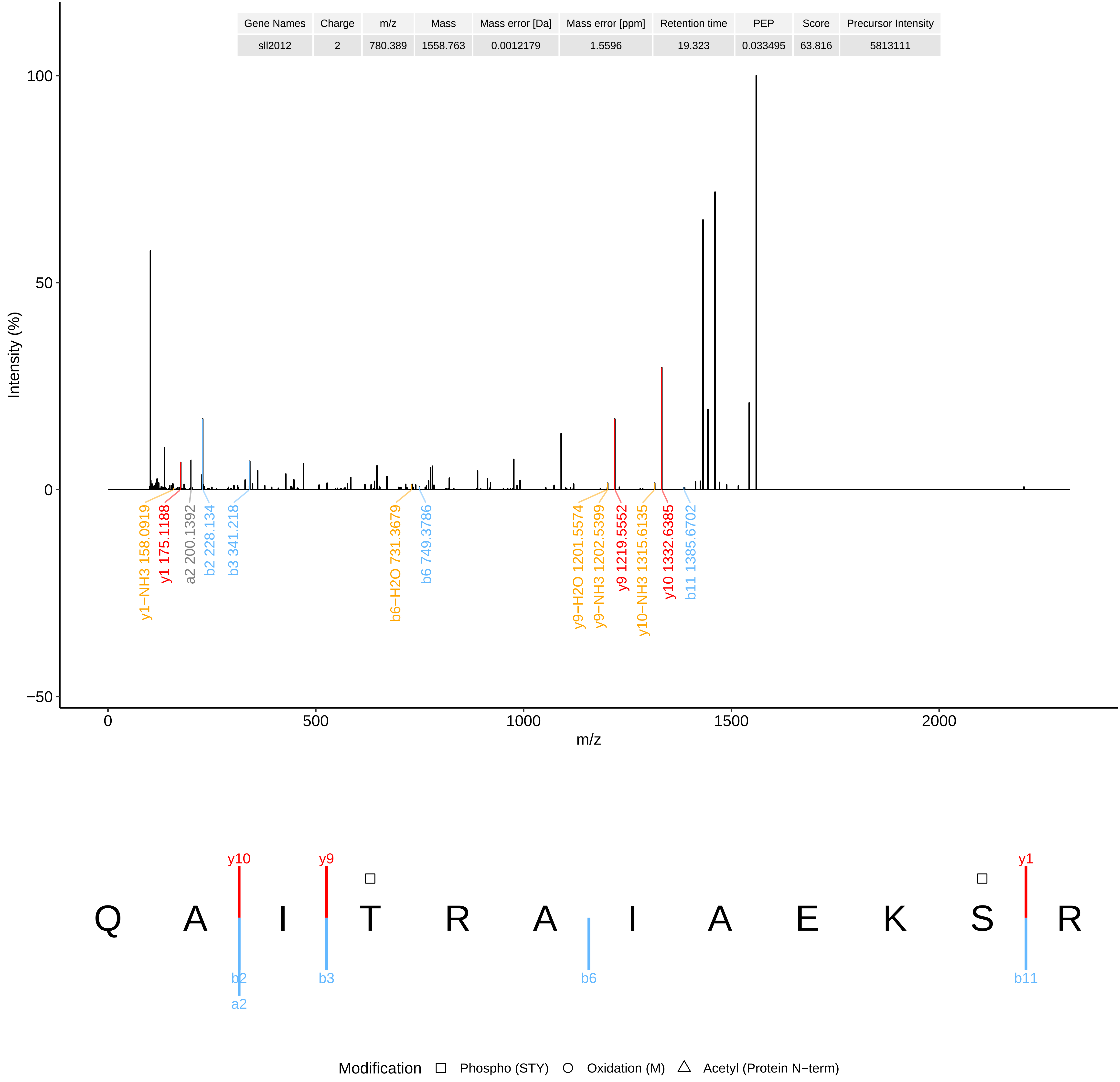

20210828_PS_SCy031_phospho_R2_TiO2_HC_F3 − Scan number: 14100

Gene Names	Charge	m/z	Mass	Mass error [Da]	Mass error [ppm]	Retention time	PEP	Score	Precursor Intensity
sll5066	2	957.4793	1912.944	NA	NA	18.156	0.018428	45.368	3726940

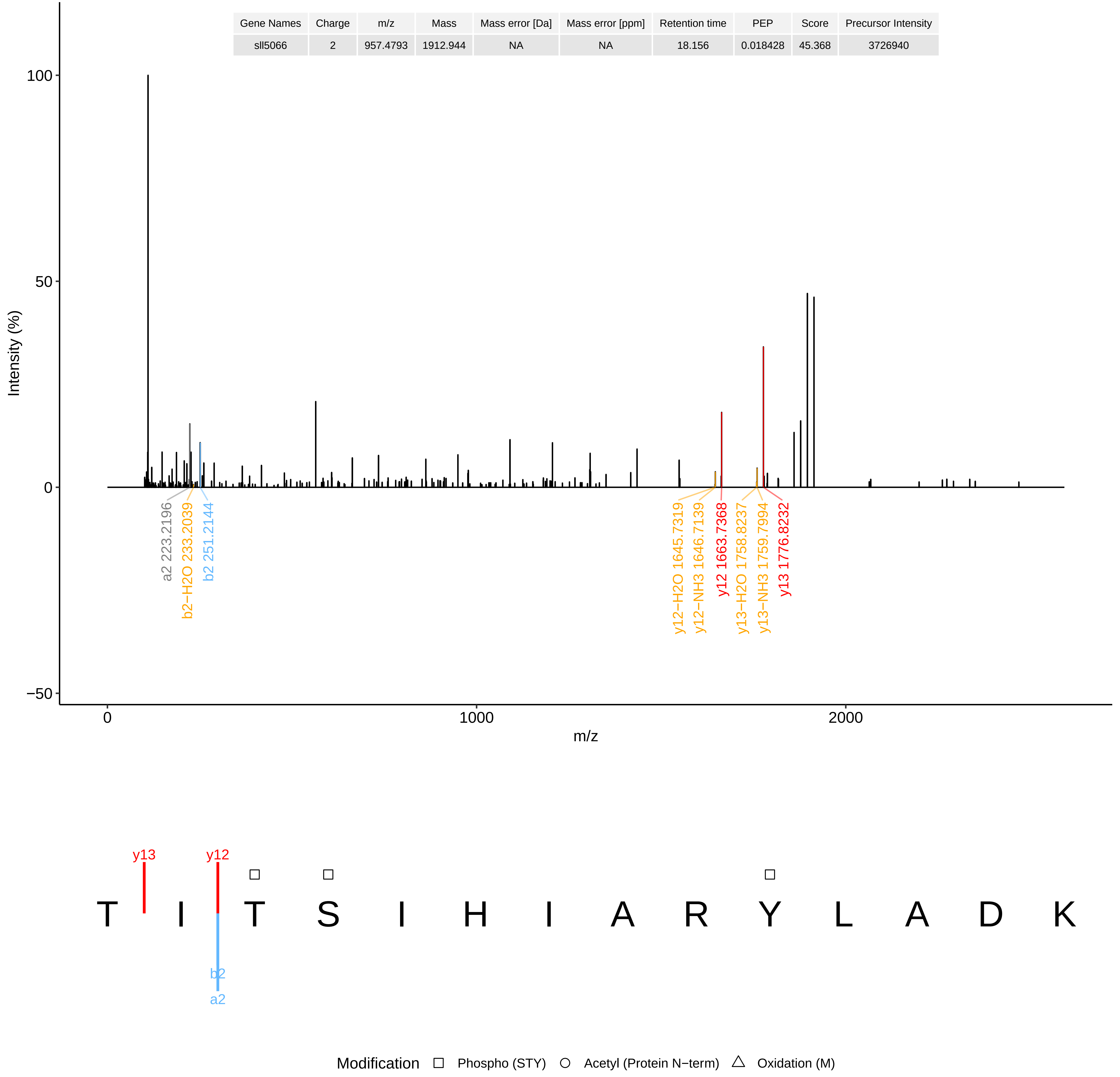

20210828_PS_SCy031_phospho_R2_TiO2_LC24h_F3 − Scan number: 9642

Gene Names	Charge	m/z	Mass	Mass error [Da]	Mass error [ppm]	Retention time	PEP	Score	Precursor Intensity
slr0012	3	569.2323	1704.675	−0.0004377	−0.7689	15.15	0.00024062	125.98	7325214

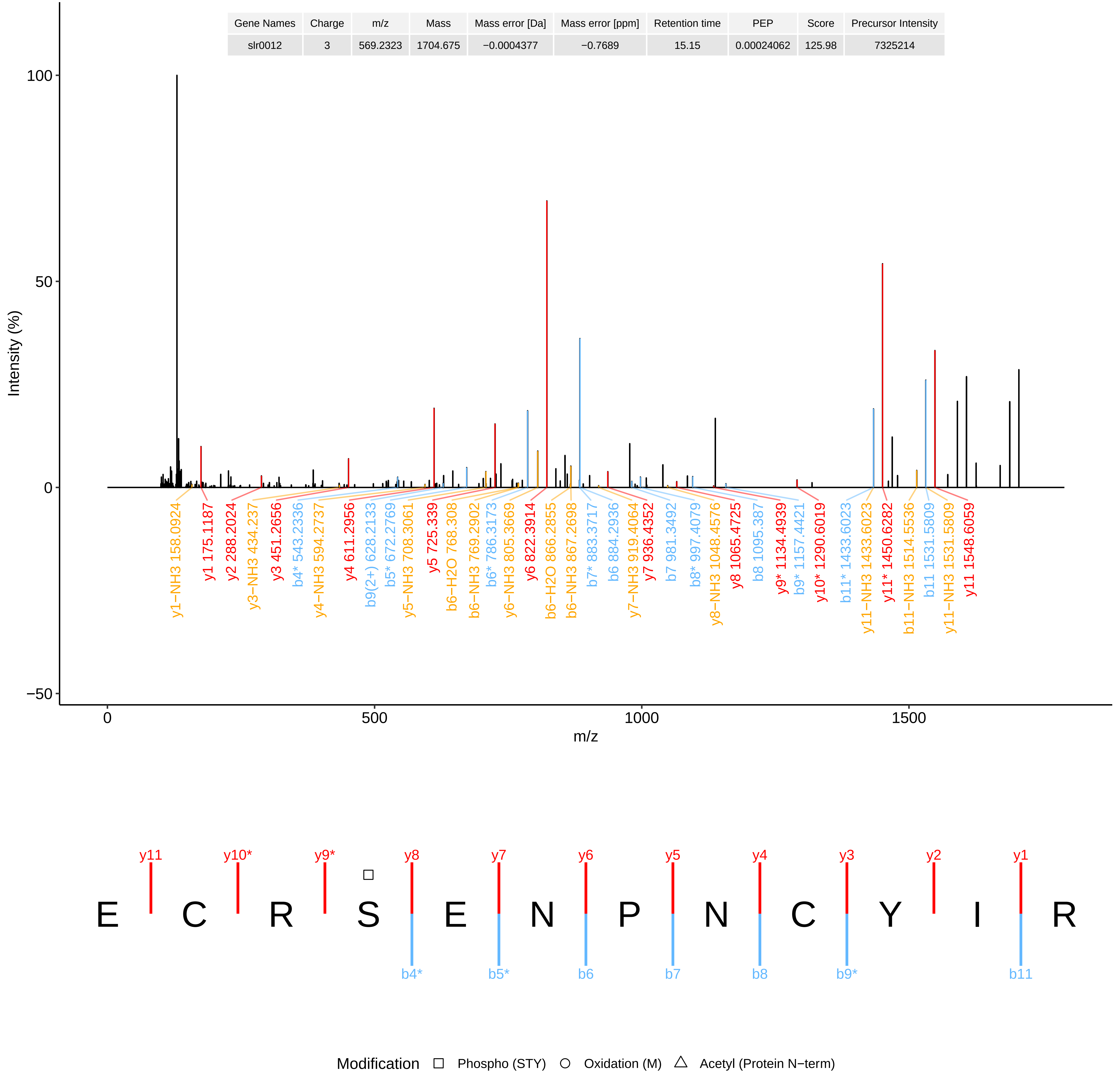

20210828_PS_SCy031_phospho_R2_TiO2_LC24h_F3 − Scan number: 15323

Gene Names	Charge	m/z	Mass	Mass error [Da]	Mass error [ppm]	Retention time	PEP	Score	Precursor Intensity
slr0038	2	745.3911	1488.768	0.0014626	1.9622	22.302	3.3187e−19	192.78	8056805

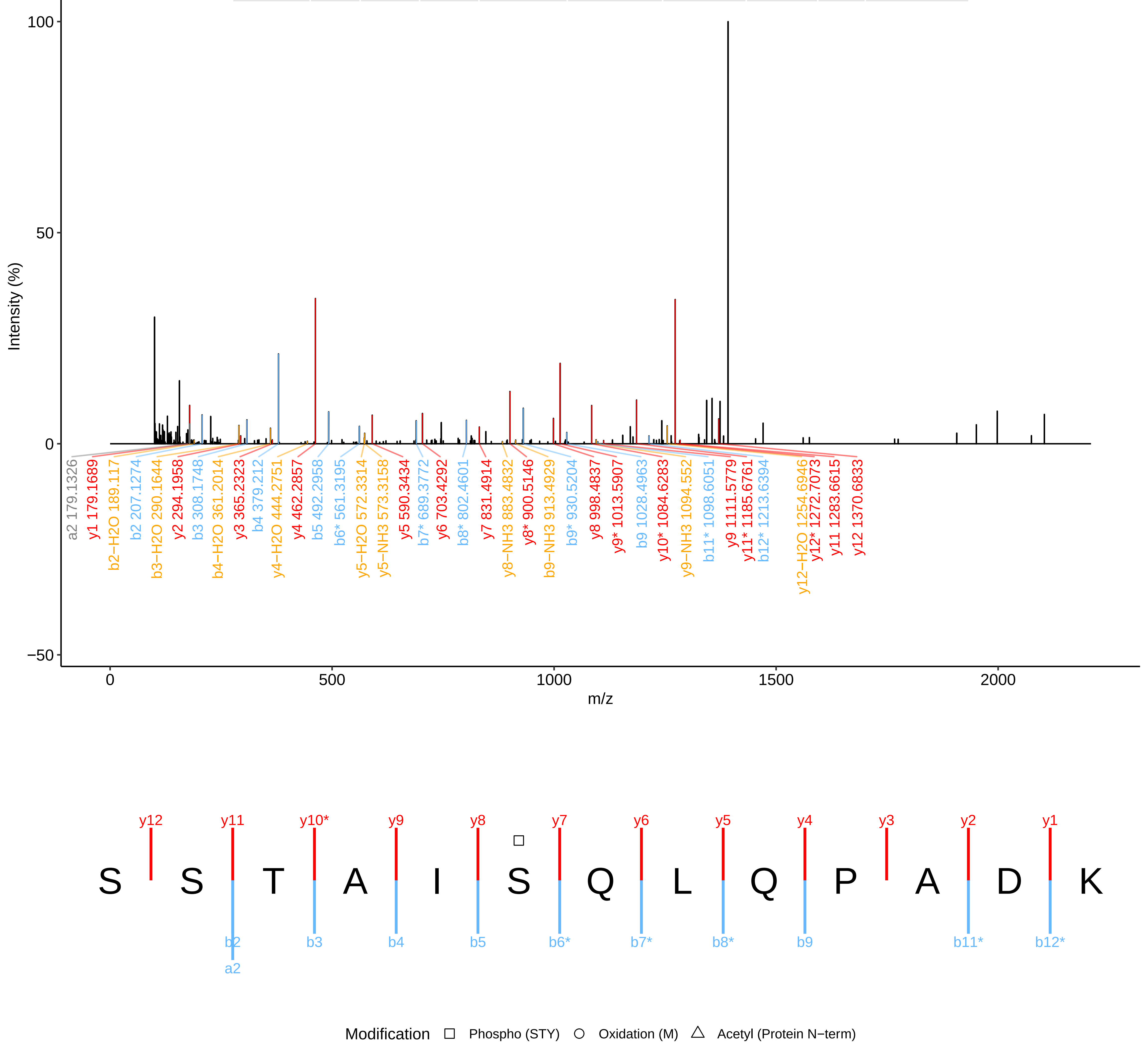

20210828_PS_SCy031_phospho_R2_TiO2_LC24h_F2 − Scan number: 24328

Gene Names	Charge	m/z	Mass	Mass error [Da]	Mass error [ppm]	Retention time	PEP	Score	Precursor Intensity
slr0040	3	1260.617	3778.83	0.0044128	3.4987	33.581	6.9594e−87	196.75	6086583

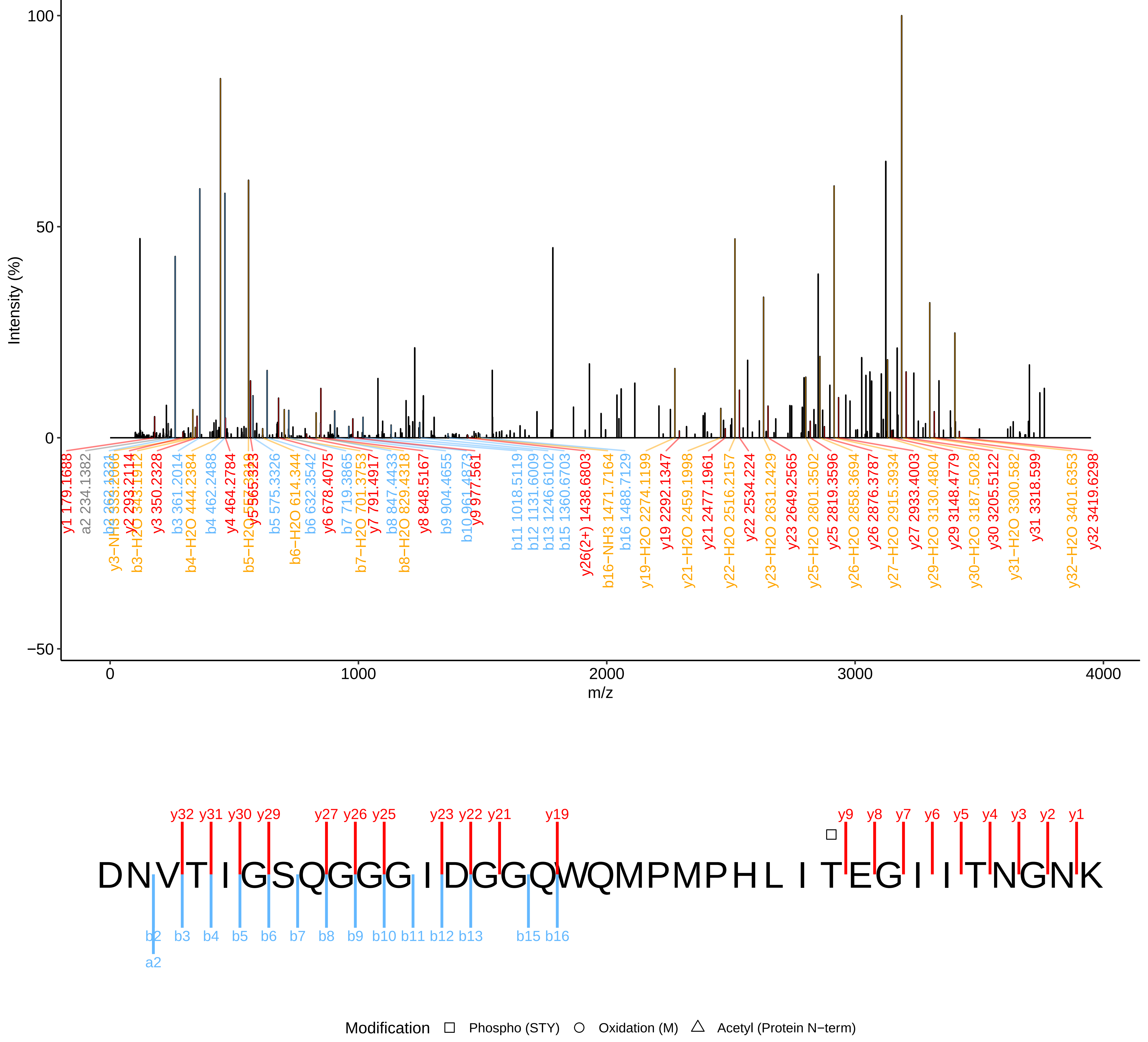

20210828_PS_SCy031_phospho_R2_TiO2_LC24h_F3 − Scan number: 9048

Gene Names	Charge	m/z	Mass	Mass error [Da]	Mass error [ppm]	Retention time	PEP	Score	Precursor Intensity
slr0041	2	464.7104	927.4063	NA	NA	14.399	0.0021077	80.438	2585935

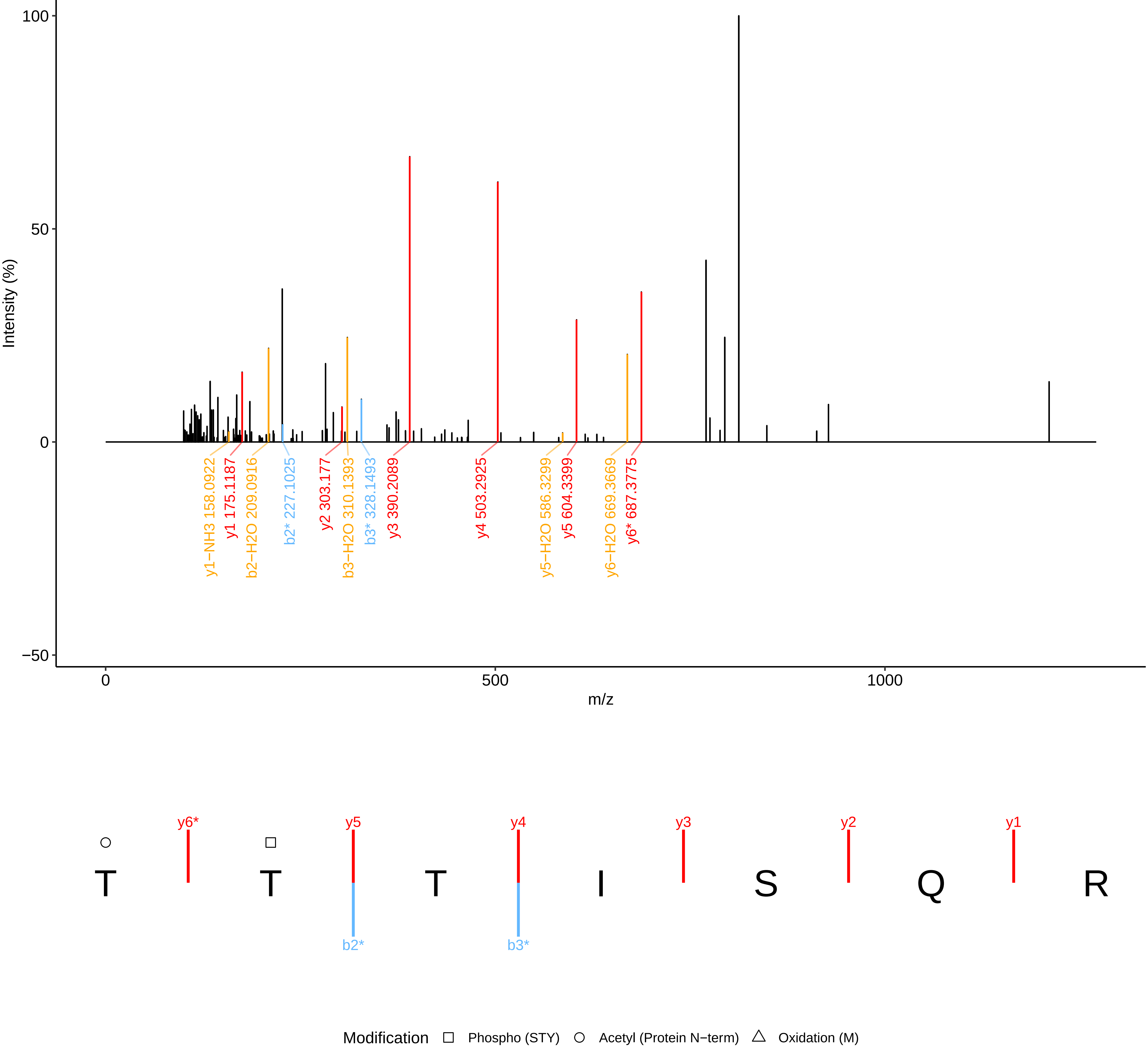

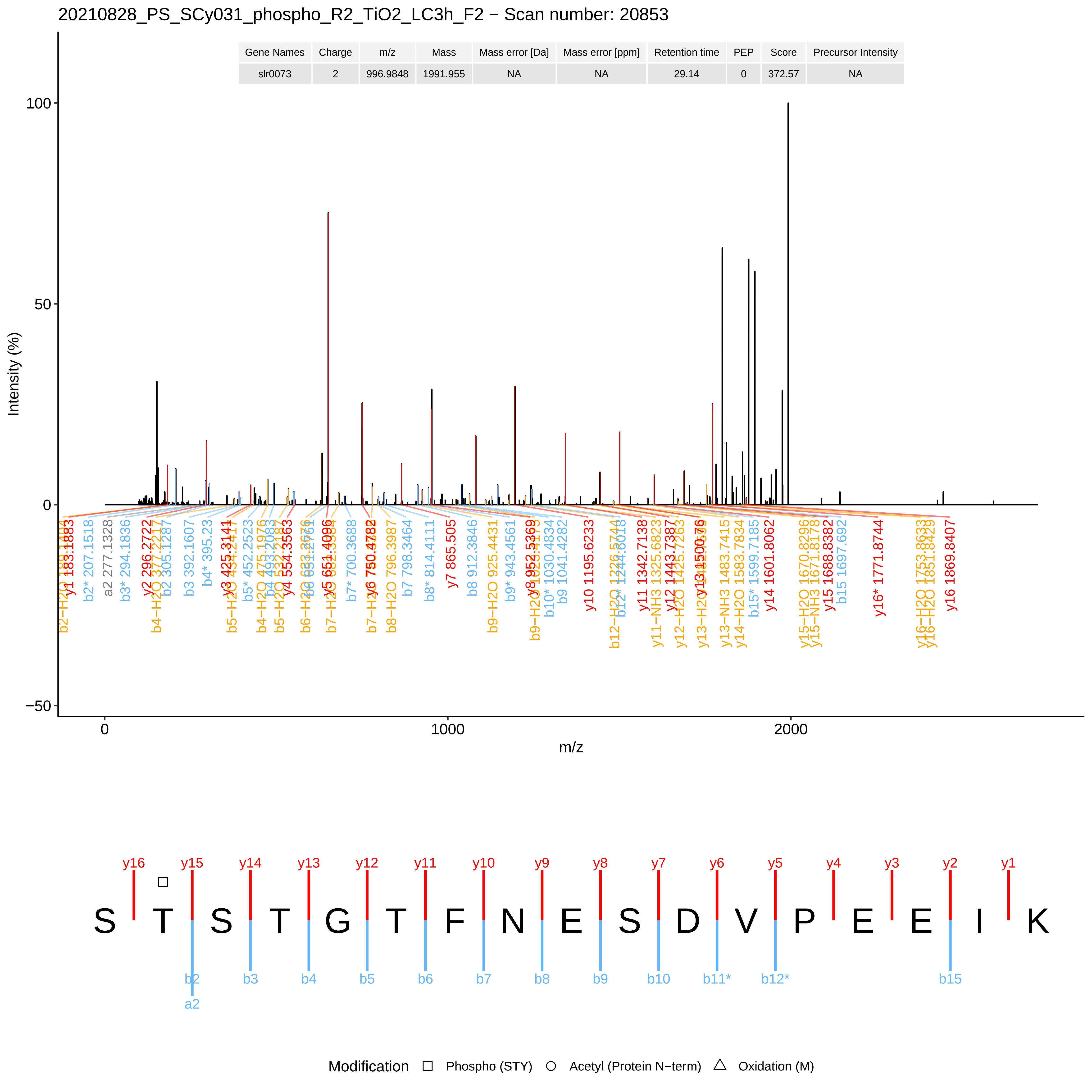

20210828_PS_SCy031_phospho_R2_TiO2_LC3h_F2 − Scan number: 20853
Gene Names | Charge | m/z | Mass | Mass error [Da] | Mass error [ppm] | Retention time | PEP | Score | Precursor Intensity
slr0073 | 2 | 996.9848 | 1991.955 | NA | NA | 29.14 | 0 | 372.57 | NA
Intensity (%)
m/z
S T S T G T F N E S D V P E E I K
y16 y15 y14 y13 y12 y11 y10 y9 y8 y7 y6 y5 y4 y3 y2 y1
b2 b3 b4 b5 b6 b7 b8 b9 b10 b11* b12* b15
a2
Modification
Phospho (STY)
Acetyl (Protein N−term)
Oxidation (M)

20210828_PS_SCy031_phospho_R3_TiO2_HC_F3 − Scan number: 20448

Gene Names	Charge	m/z	Mass	Mass error [Da]	Mass error [ppm]	Retention time	PEP	Score	Precursor Intensity
slr0073	2	996.9848	1991.955	NA	NA	28.833	0.00014721	136.36	3557238

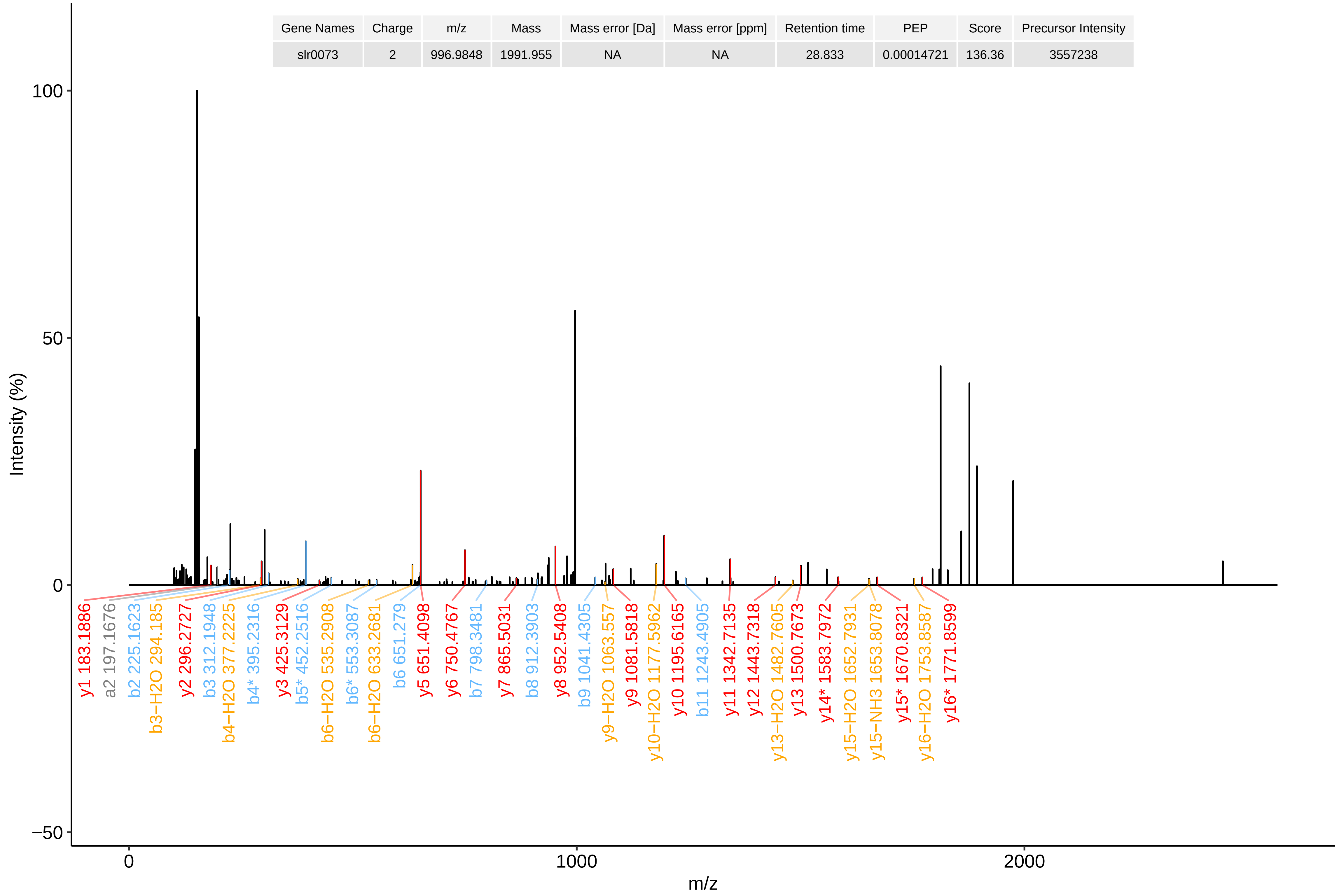

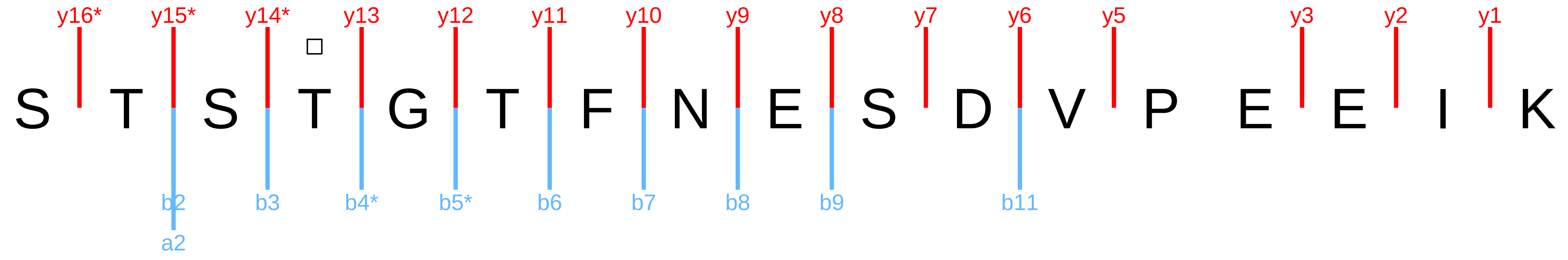

Modification □ Phospho (STY) ○ Acetyl (Protein N−term) △ Oxidation (M)

20210823_PS_SCy031_R1_phospho_LC24h_TiO2_1 − Scan number: 20265

Gene Names	Charge	m/z	Mass	Mass error [Da]	Mass error [ppm]	Retention time	PEP	Score	Precursor Intensity
slr0073	2	992.9656	1983.917	−0.0014185	−1.4278	28.173	1.4709e−258	353.05	7863231

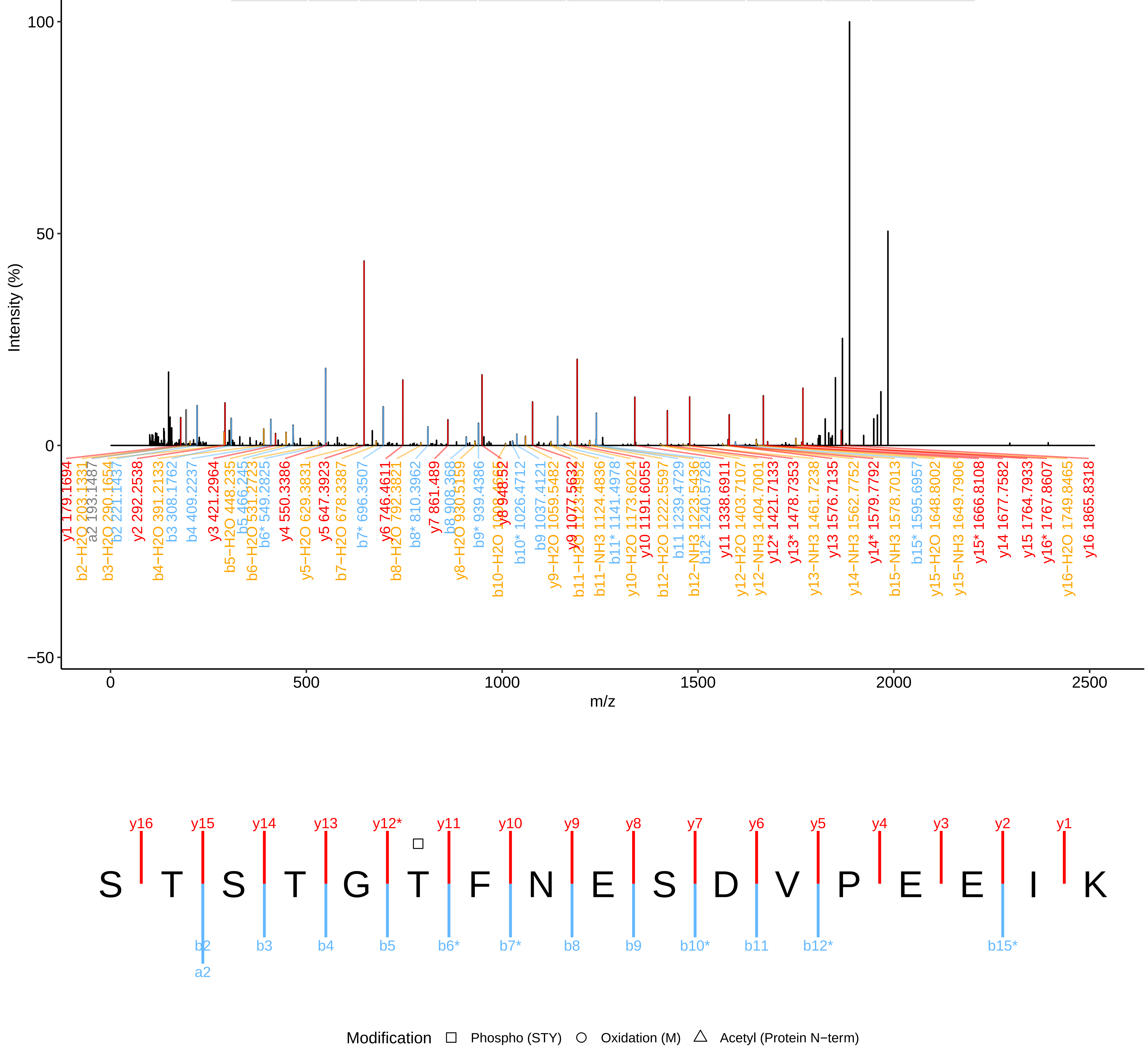

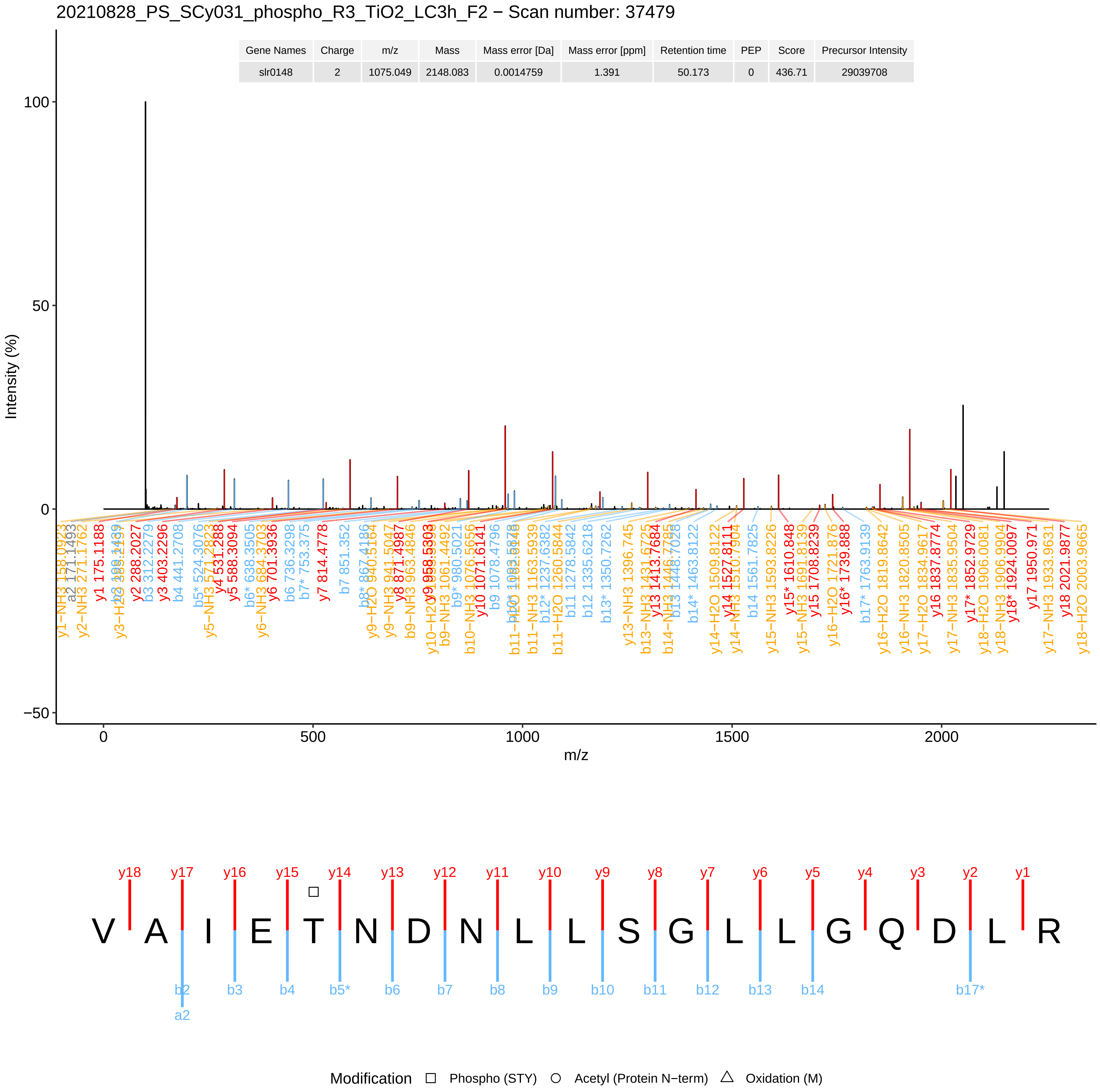

20210828_PS_SCy031_phospho_R3_TiO2_LC3h_F2 − Scan number: 37479
Gene Names | Charge | m/z | Mass | Mass error [Da] | Mass error [ppm] | Retention time | PEP | Score | Precursor Intensity
slr0148 | 2 | 1075.049 | 2148.083 | 0.0014759 | 1.391 | 50.173 | 0 | 436.71 | 29039708
Intensity (%)
m/z
V A I E T N D N L L S G L L G Q D L R
Modification
Phospho (STY)
Acetyl (Protein N−term)
Oxidation (M)

20211217_PS_SCy031_Proteome_R2_24hLC_F1 − Scan number: 18742

Gene Names	Charge	m/z	Mass	Mass error [Da]	Mass error [ppm]	Retention time	PEP	Score	Precursor Intensity
slr0148	3	433.8998	1298.678	0.00027683	0.65618	27.415	0.0002836	72.958	7148821

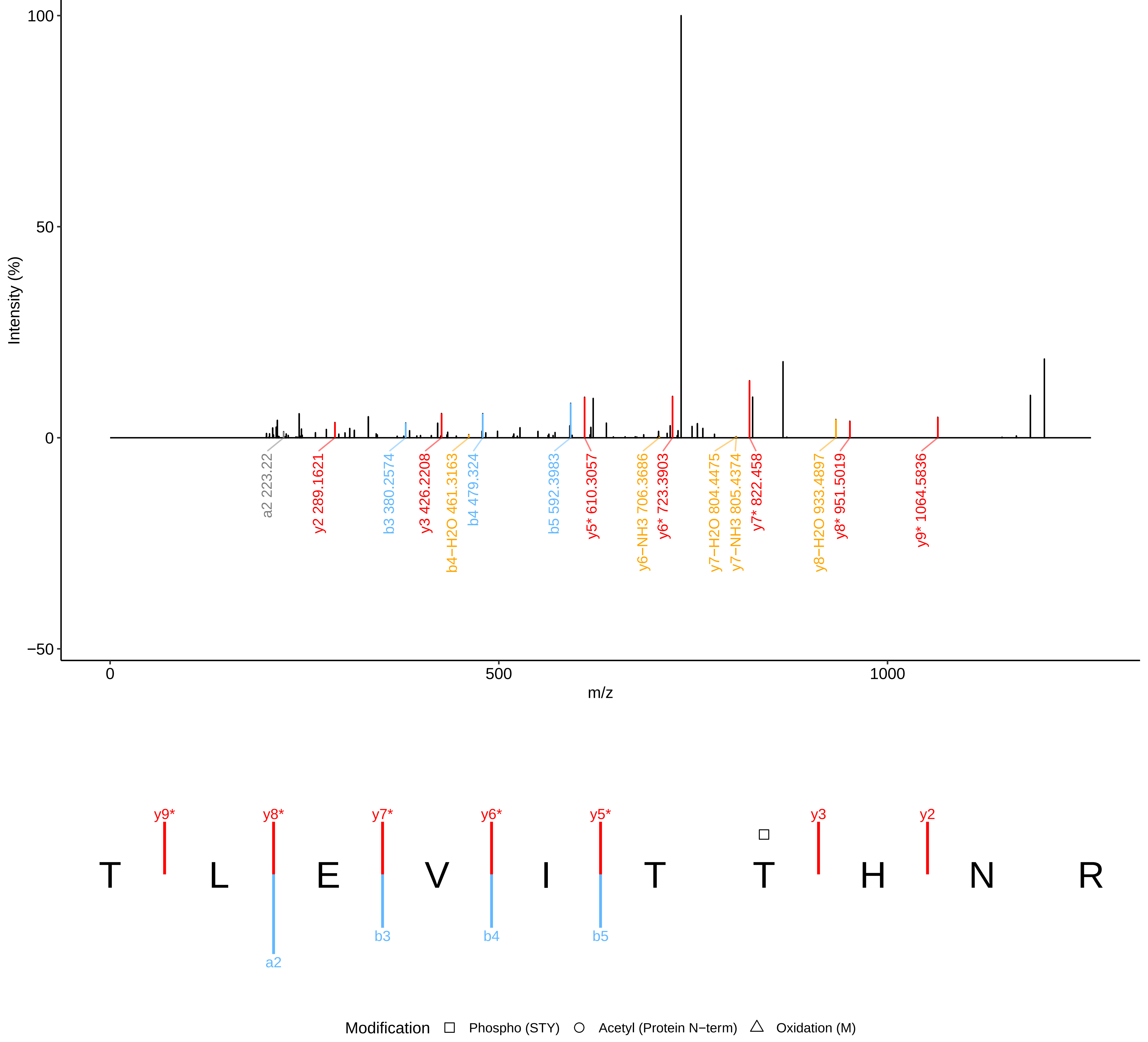

20210828_PS_SCy031_phospho_R2_TiO2_LC24h_F1 − Scan number: 24644

Gene Names	Charge	m/z	Mass	Mass error [Da]	Mass error [ppm]	Retention time	PEP	Score	Precursor Intensity
slr0151	3	956.4693	2866.386	−0.00047893	−0.50055	34.027	7.9665e−16	125.82	2885698

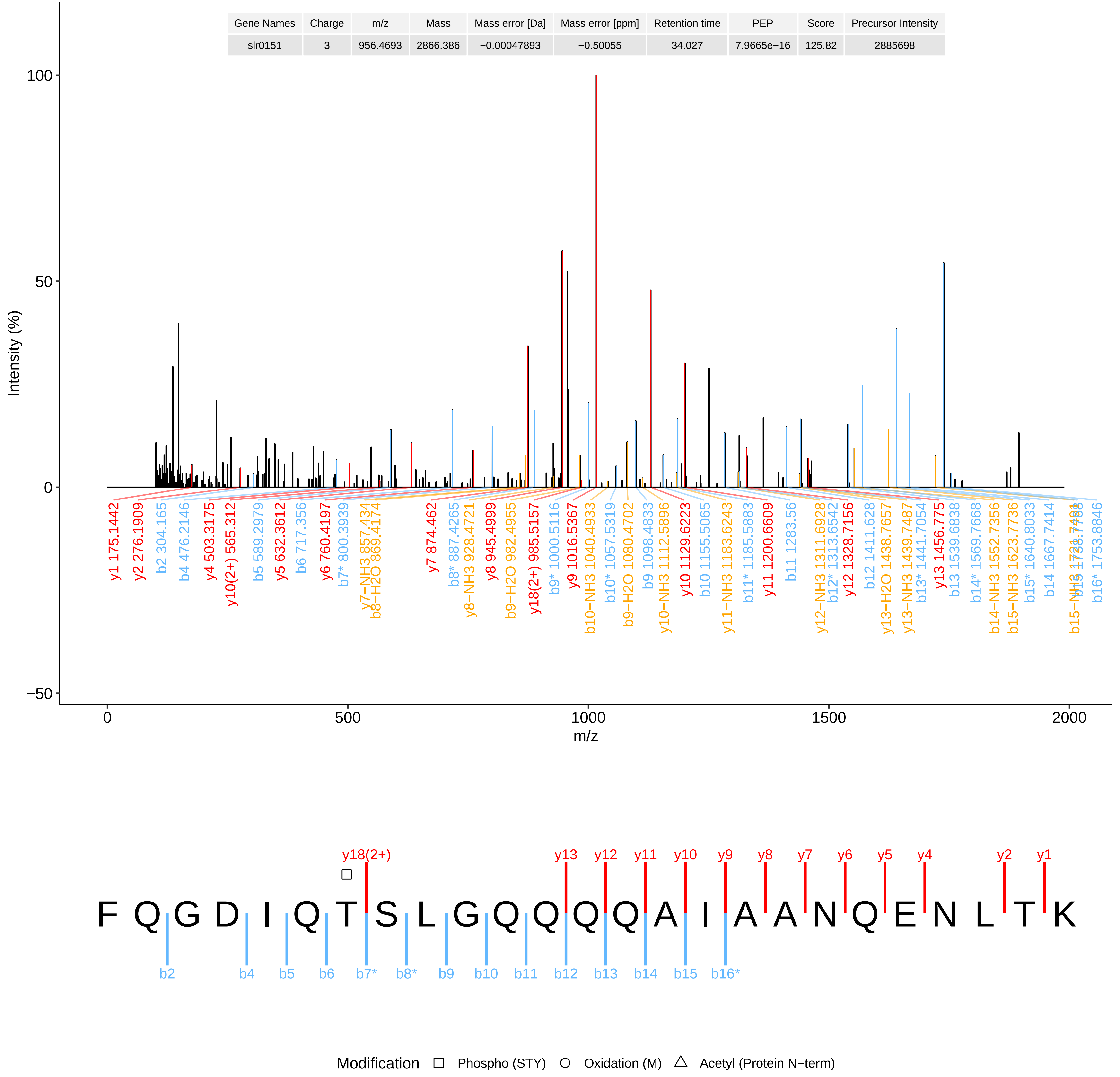

20210828_PS_SCy031_phospho_R3_TiO2_LC24h_F1 − Scan number: 10066

Gene Names	Charge	m/z	Mass	Mass error [Da]	Mass error [ppm]	Retention time	PEP	Score	Precursor Intensity
slr0152	2	806.3208	1610.627	0.00023887	0.30302	15.399	1.6137e−155	290.26	9061053

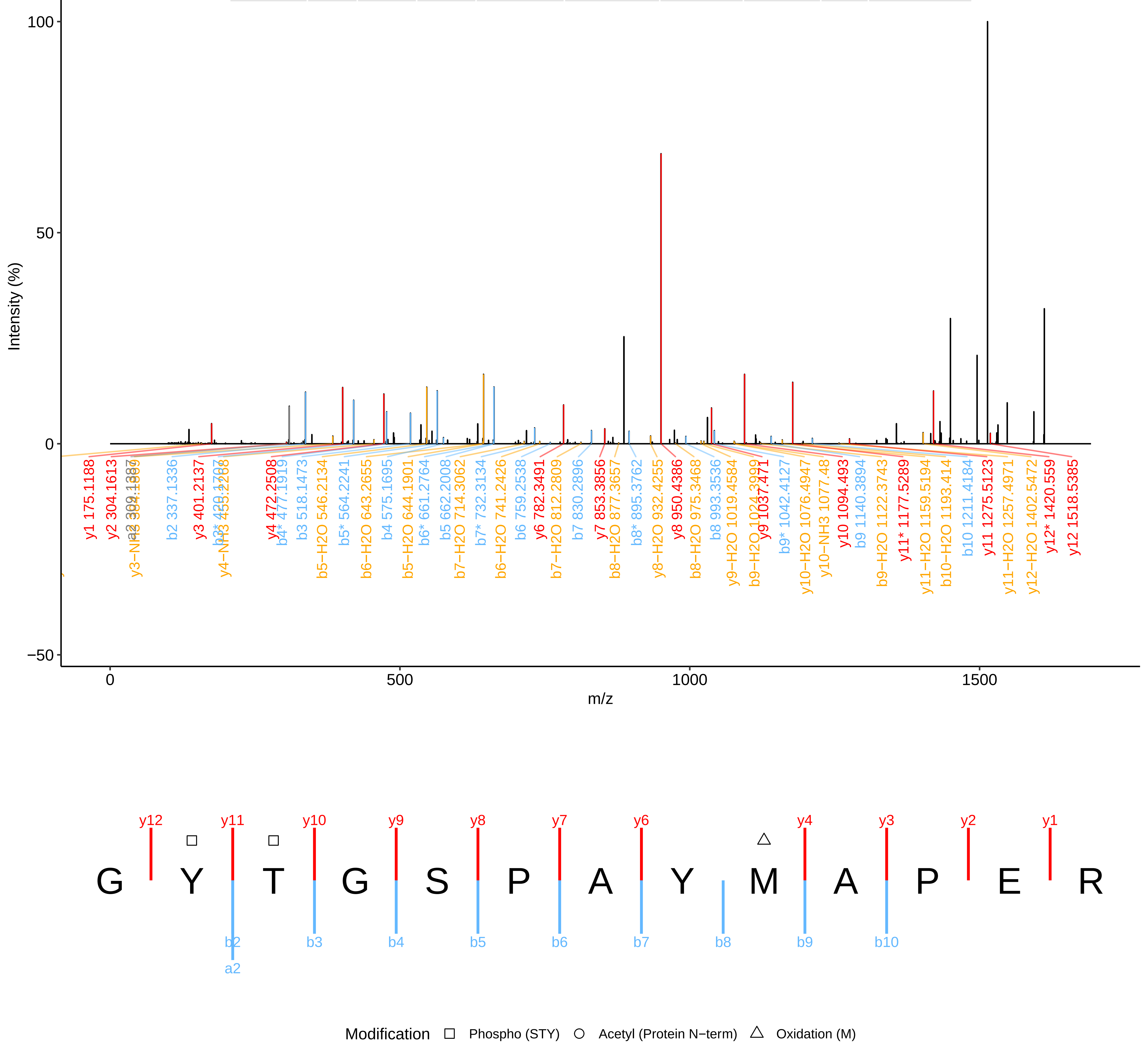

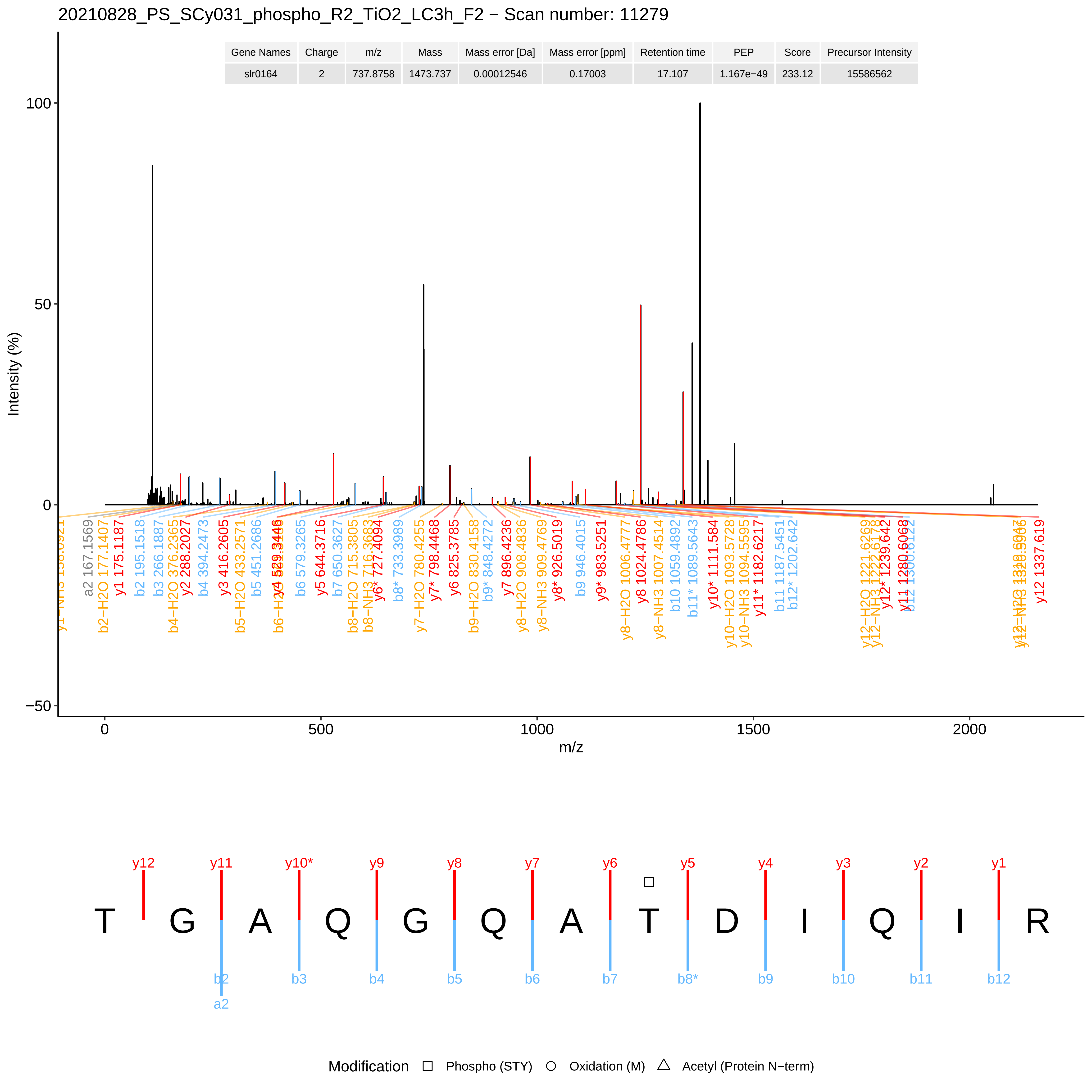

20210828_PS_SCy031_phospho_R2_TiO2_LC3h_F2 − Scan number: 11279
Gene Names | Charge | m/z | Mass | Mass error [Da] | Mass error [ppm] | Retention time | PEP | Score | Precursor Intensity
slr0164 | 2 | 737.8758 | 1473.737 | 0.00012546 | 0.17003 | 17.107 | 1.167e−49 | 233.12 | 15586562
Intensity (%)
m/z
a2 167.1569
b2−H2O 177.1407
y1 175.1187
b2 195.1518
b3 266.1887
b4−H2O 376.2365
y2 288.2027
b4 394.2473
y3 416.2605
b5−H2O 433.2571
b5 451.2686
b6 579.3265
y5 644.3716
b7 650.3627
b8−H2O 715.3805
y6* 727.4094
b8* 733.3989
b7−H2O 780.4255
y7* 798.4468
y6 825.3785
b9−H2O 830.4158
b9* 848.4272
y7 896.4236
y8−H2O 908.4836
y8−NH3 909.4769
y8* 926.5019
b9 946.4015
y9* 983.5251
y8−H2O 1006.4777
y8 1024.4786
y8−NH3 1007.4514
b10 1059.4892
b11* 1089.5643
y10* 1111.584
y10−H2O 1093.5728
y10−NH3 1094.5595
y11* 1182.6217
b11 1187.5451
b12* 1202.642
y12* 1239.642
y11 1280.6068
b12 1300.6122
y12 1337.619
T G A Q G Q A T D I Q I R
y12 y11 y10* y9 y8 y7 y6 y5 y4 y3 y2 y1
b2 b3 b4 b5 b6 b7 b8* b9 b10 b11 b12
a2
Modification
Phospho (STY)
Oxidation (M)
Acetyl (Protein N−term)

20210828_PS_SCy031_phospho_R3_TiO2_HC_F2 − Scan number: 12476

Gene Names	Charge	m/z	Mass	Mass error [Da]	Mass error [ppm]	Retention time	PEP	Score	Precursor Intensity
slr0193	2	1062.985	2123.955	NA	NA	18.659	3.6204e−07	94.958	NA

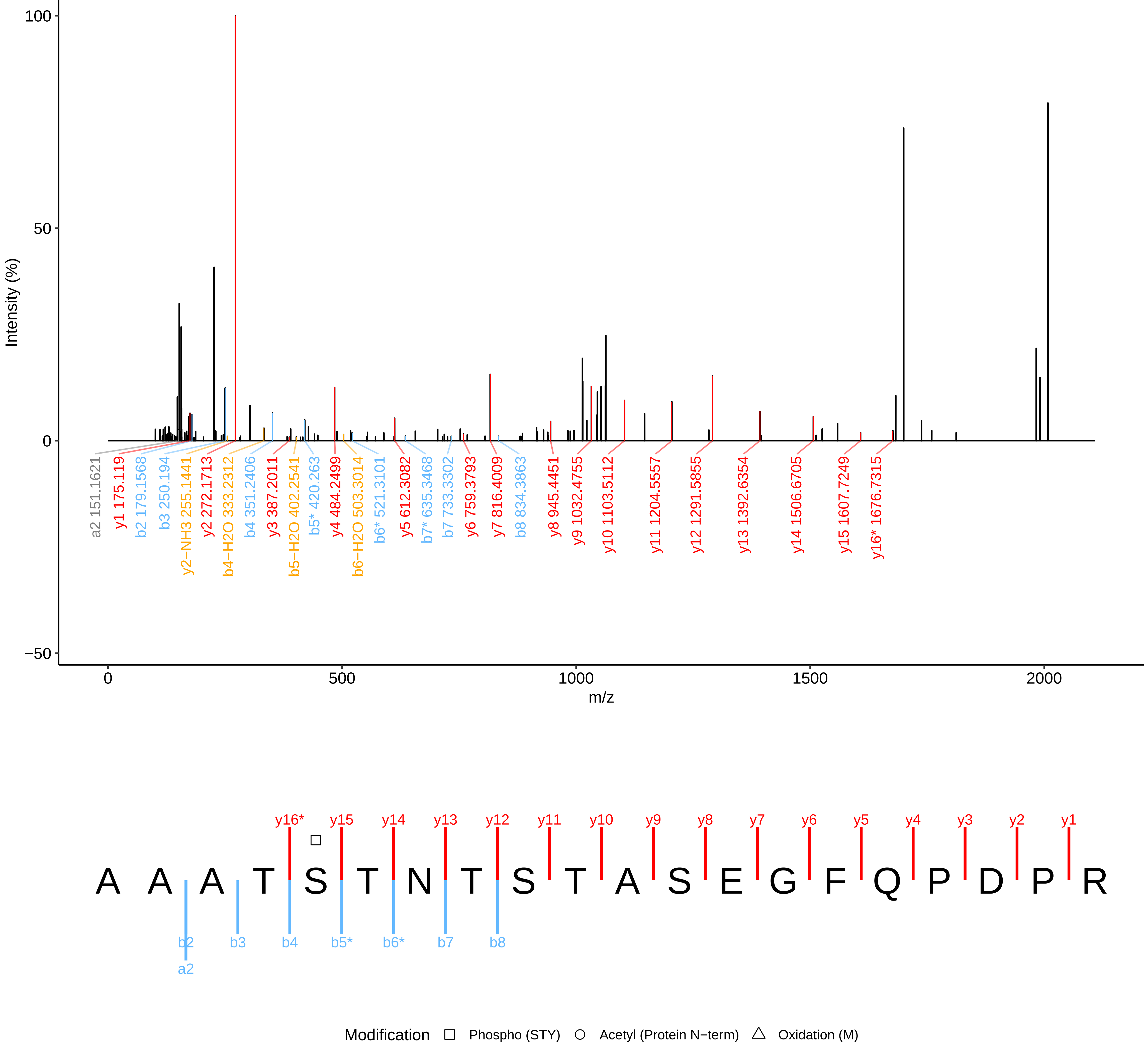

20210823_PS_SCy031_R1_phospho_HC_TiO2_2 − Scan number: 5893

Gene Names	Charge	m/z	Mass	Mass error [Da]	Mass error [ppm]	Retention time	PEP	Score	Precursor Intensity
slr0322	2	468.2122	934.4099	9.7769e−05	0.21622	10.316	0.012355	105.1	17902506

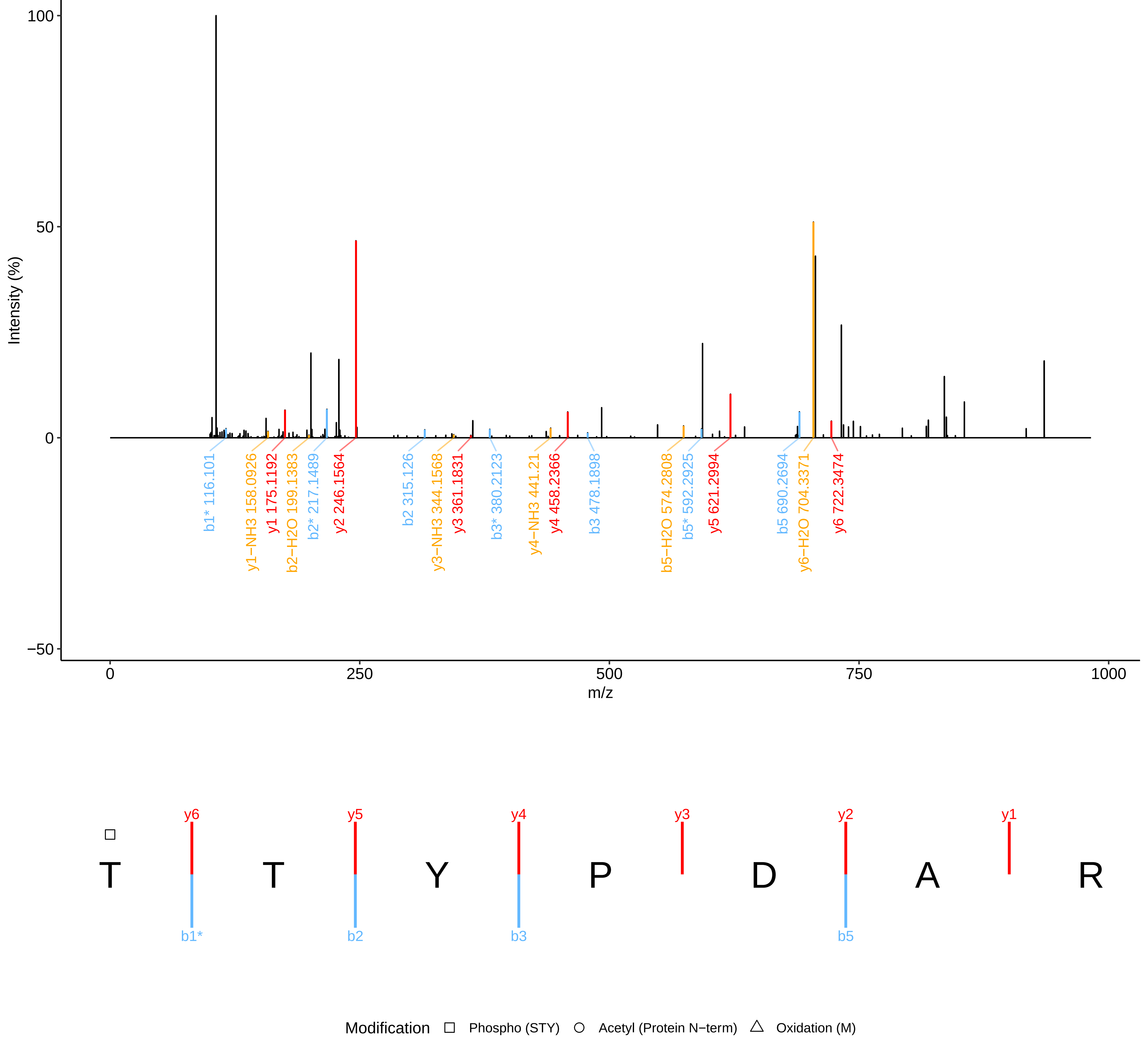

20210823_PS_SCy031_R1_phospho_LC3h_TiO2_2 − Scan number: 6143

Gene Names	Charge	m/z	Mass	Mass error [Da]	Mass error [ppm]	Retention time	PEP	Score	Precursor Intensity
slr0322	2	470.2219	938.4292	−0.00014959	−0.33081	10.762	0.009351	88.177	11726340

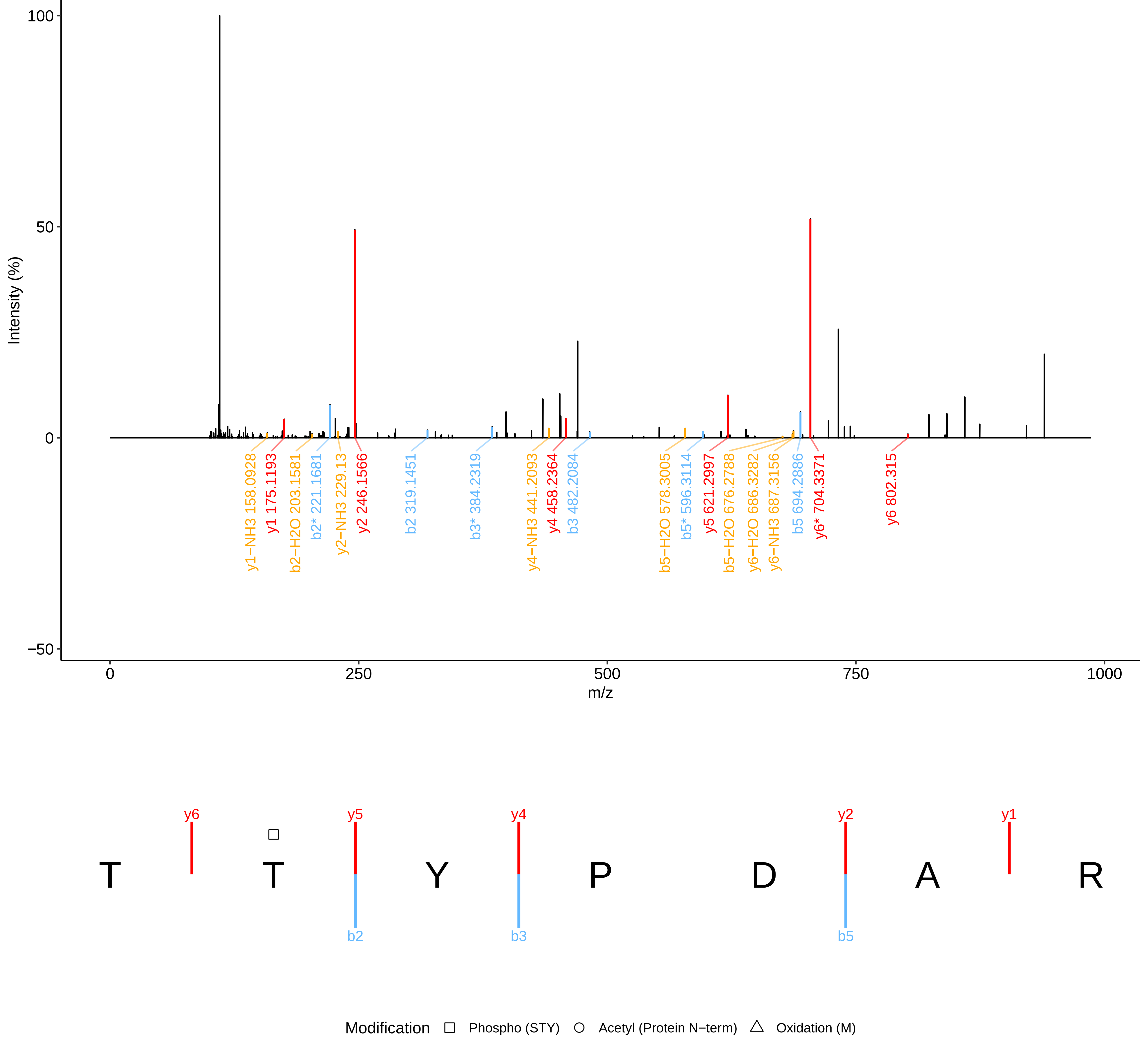

20210828_PS_SCy031_phospho_R3_TiO2_LC24h_F2 − Scan number: 5031

Gene Names	Charge	m/z	Mass	Mass error [Da]	Mass error [ppm]	Retention time	PEP	Score	Precursor Intensity
slr0322	2	430.7246	859.4346	5.7836e−05	0.14014	9.3493	0.0089329	115.06	13802348

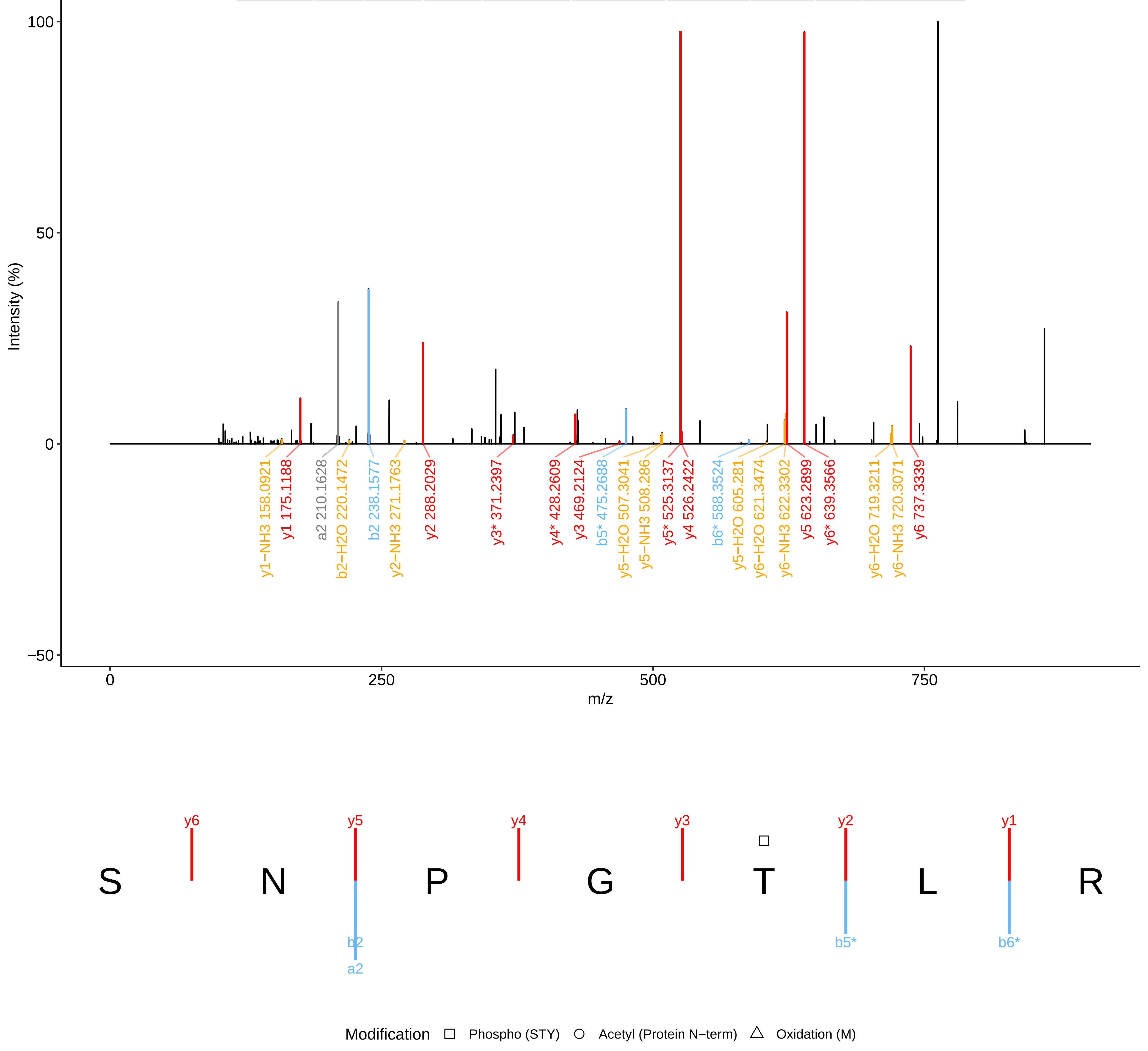

20211217_PS_SCy031_Proteome_R3_HC_F8 − Scan number: 30069

Gene Names	Charge	m/z	Mass	Mass error [Da]	Mass error [ppm]	Retention time	PEP	Score	Precursor Intensity
slr0374	3	741.0485	2220.124	9.3435	12935	51.811	0.0013754	53.995	7088356

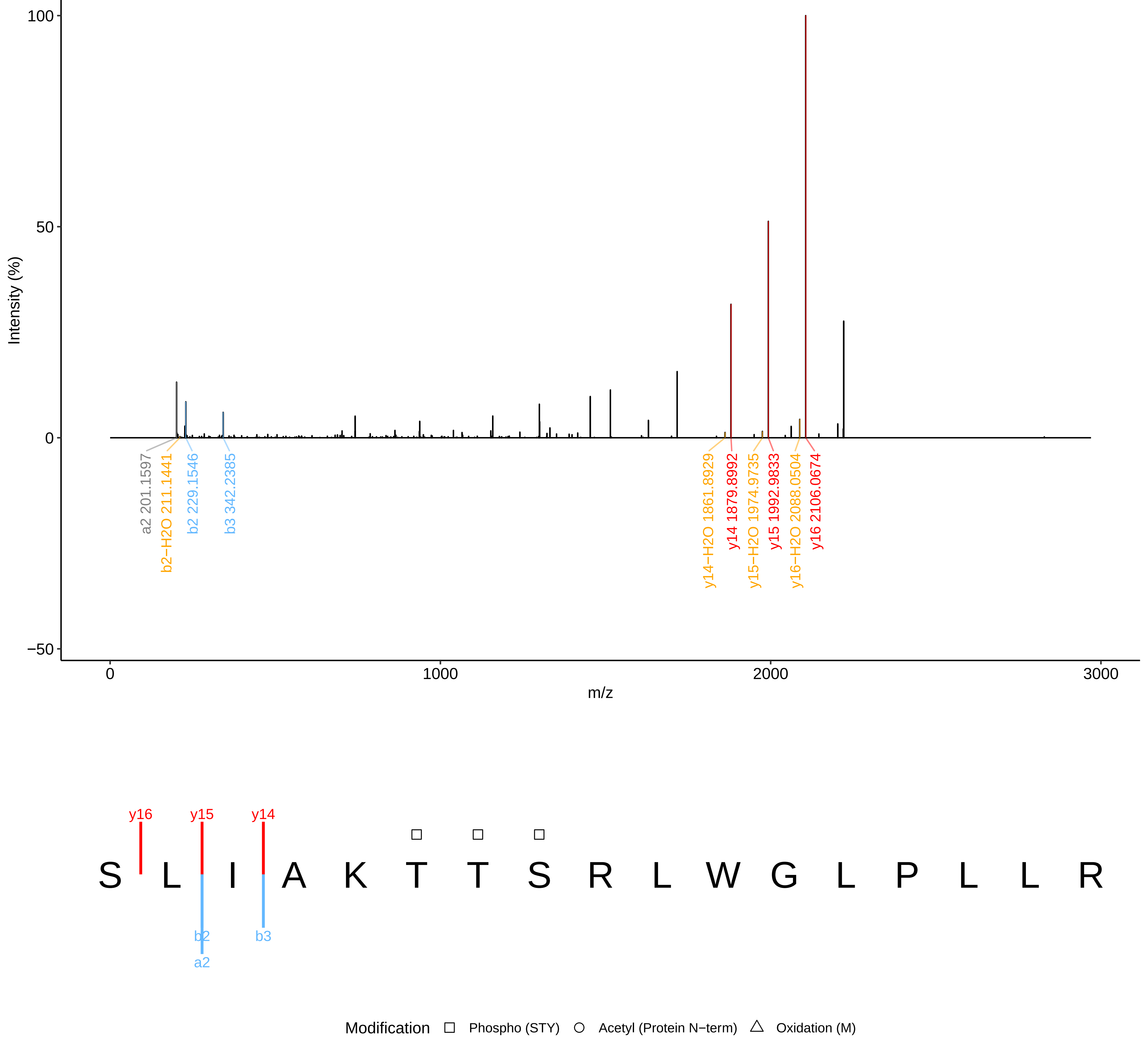

20210828_PS_SCy031_phospho_R3_TiO2_HC_F1 − Scan number: 9852

Gene Names	Charge	m/z	Mass	Mass error [Da]	Mass error [ppm]	Retention time	PEP	Score	Precursor Intensity
slr0377	2	942.9903	1883.966	−0.0032983	−3.4978	15.248	0.0046927	68.893	11503340

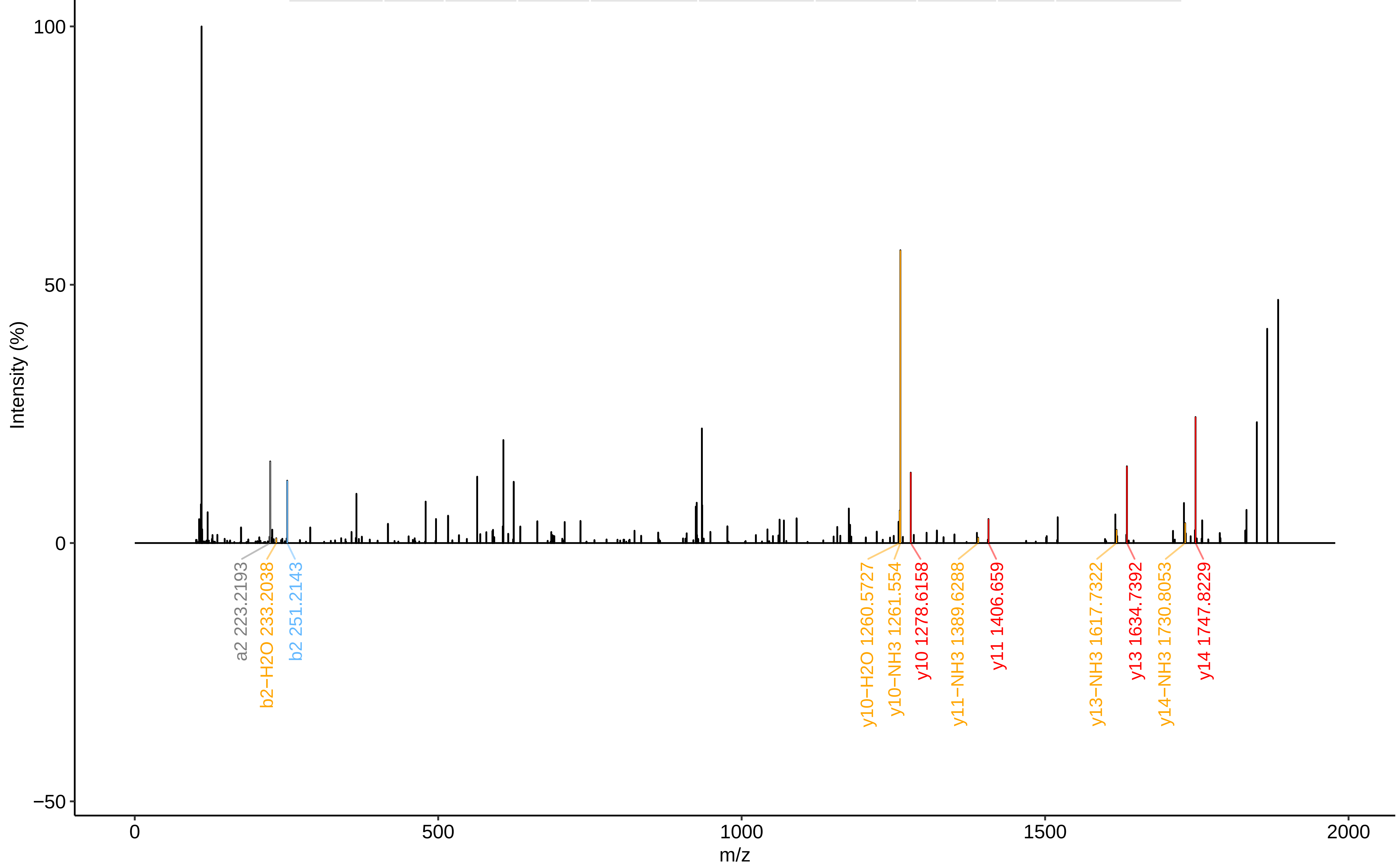

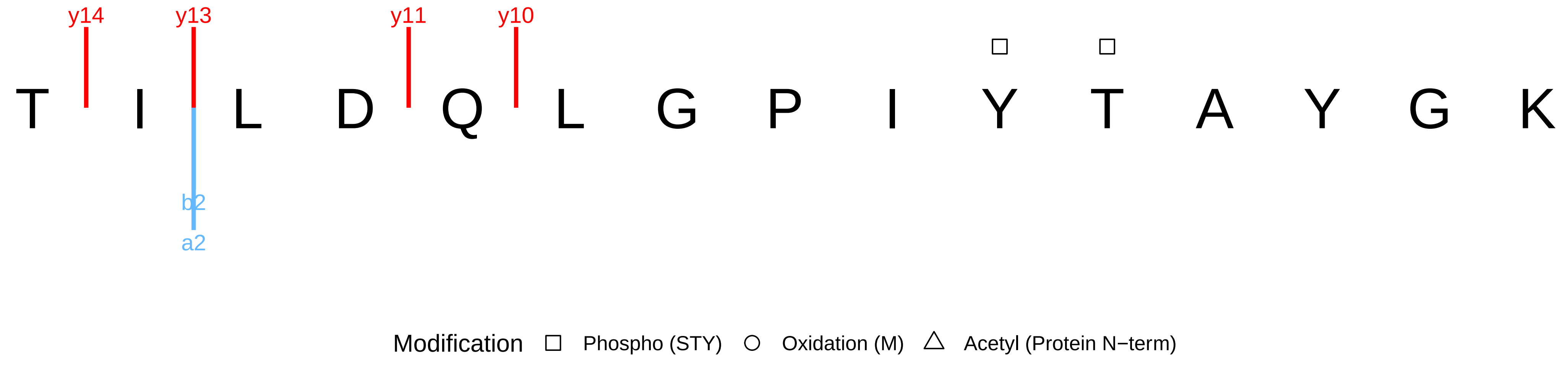

20211212_PS_SCy031_proteome_R1_24hLC_F1 − Scan number: 21577

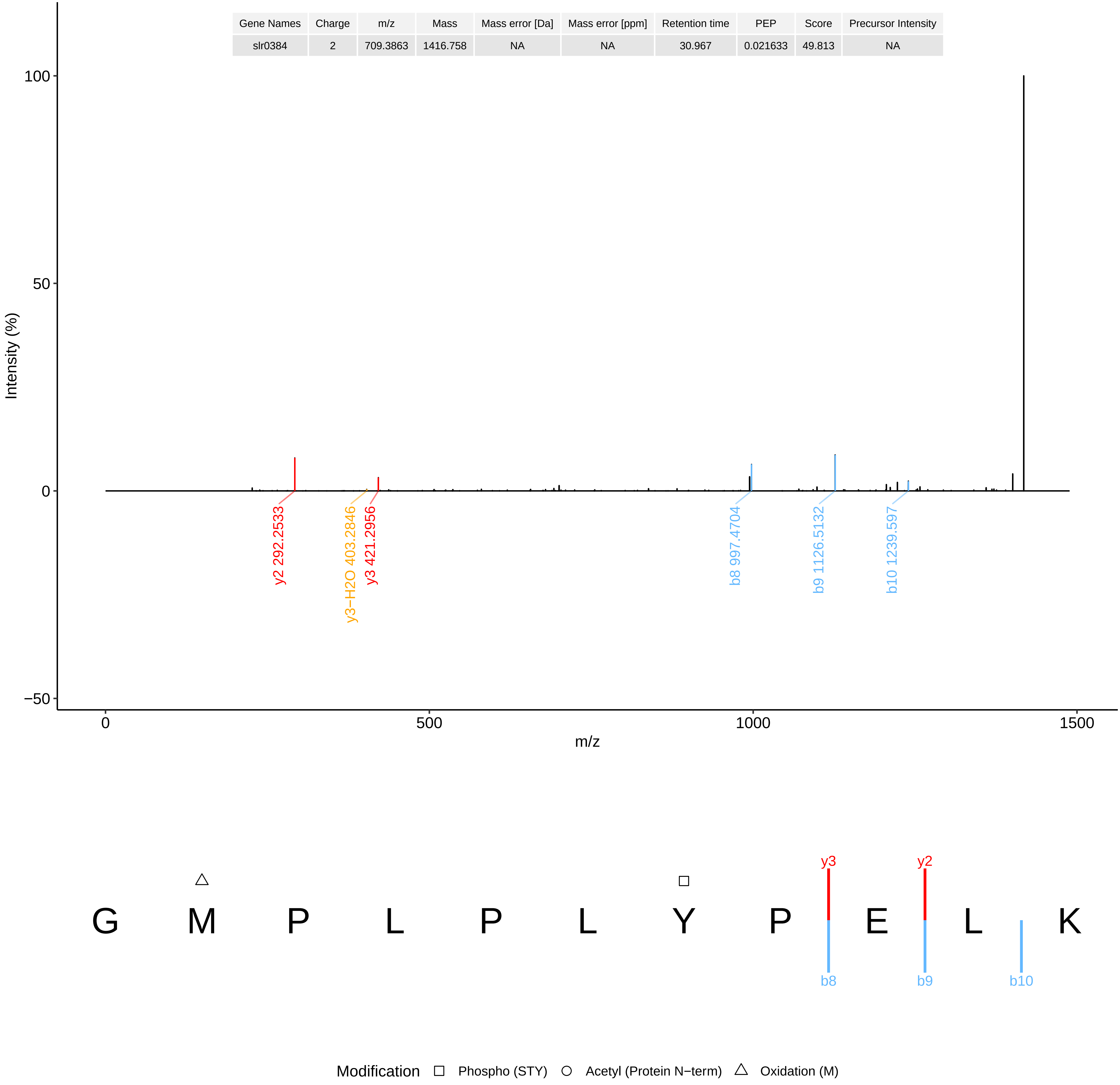

Gene Names	Charge	m/z	Mass	Mass error [Da]	Mass error [ppm]	Retention time	PEP	Score	Precursor Intensity
slr0384	2	709.3863	1416.758	NA	NA	30.967	0.021633	49.813	NA

20210828_PS_SCy031_phospho_R2_TiO2_LC3h_F3 − Scan number: 26589

Gene Names	Charge	m/z	Mass	Mass error [Da]	Mass error [ppm]	Retention time	PEP	Score	Precursor Intensity
slr0397	3	765.0507	2292.13	NA	NA	36.646	1.2472e−05	75.868	NA

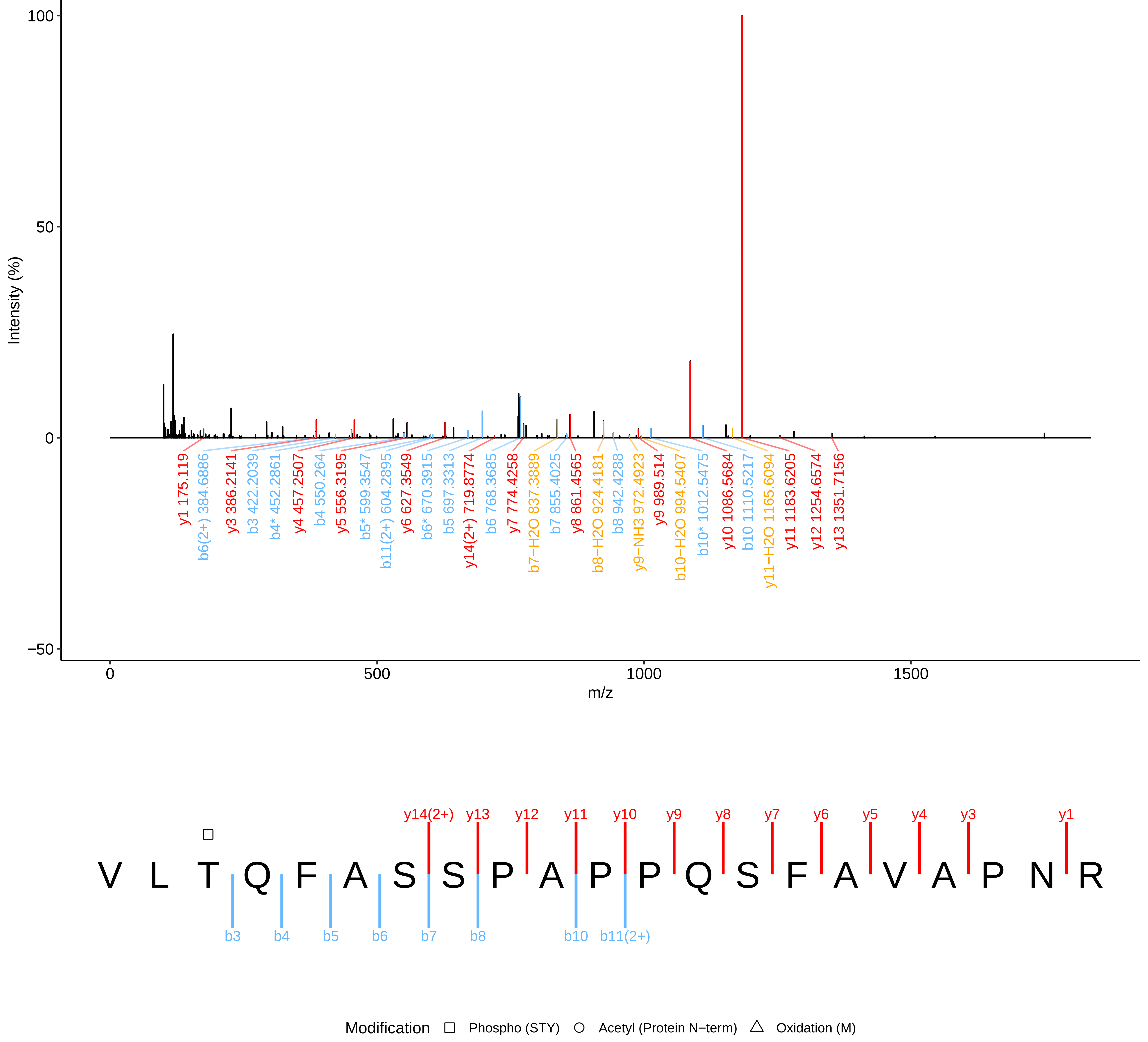

20210823_PS_SCy031_R1_phospho_HC_TiO2_2 − Scan number: 11024

Gene Names	Charge	m/z	Mass	Mass error [Da]	Mass error [ppm]	Retention time	PEP	Score	Precursor Intensity
slr0447	3	762.0296	2283.067	0.0030887	4.0514	16.731	0.015067	55.437	4925306

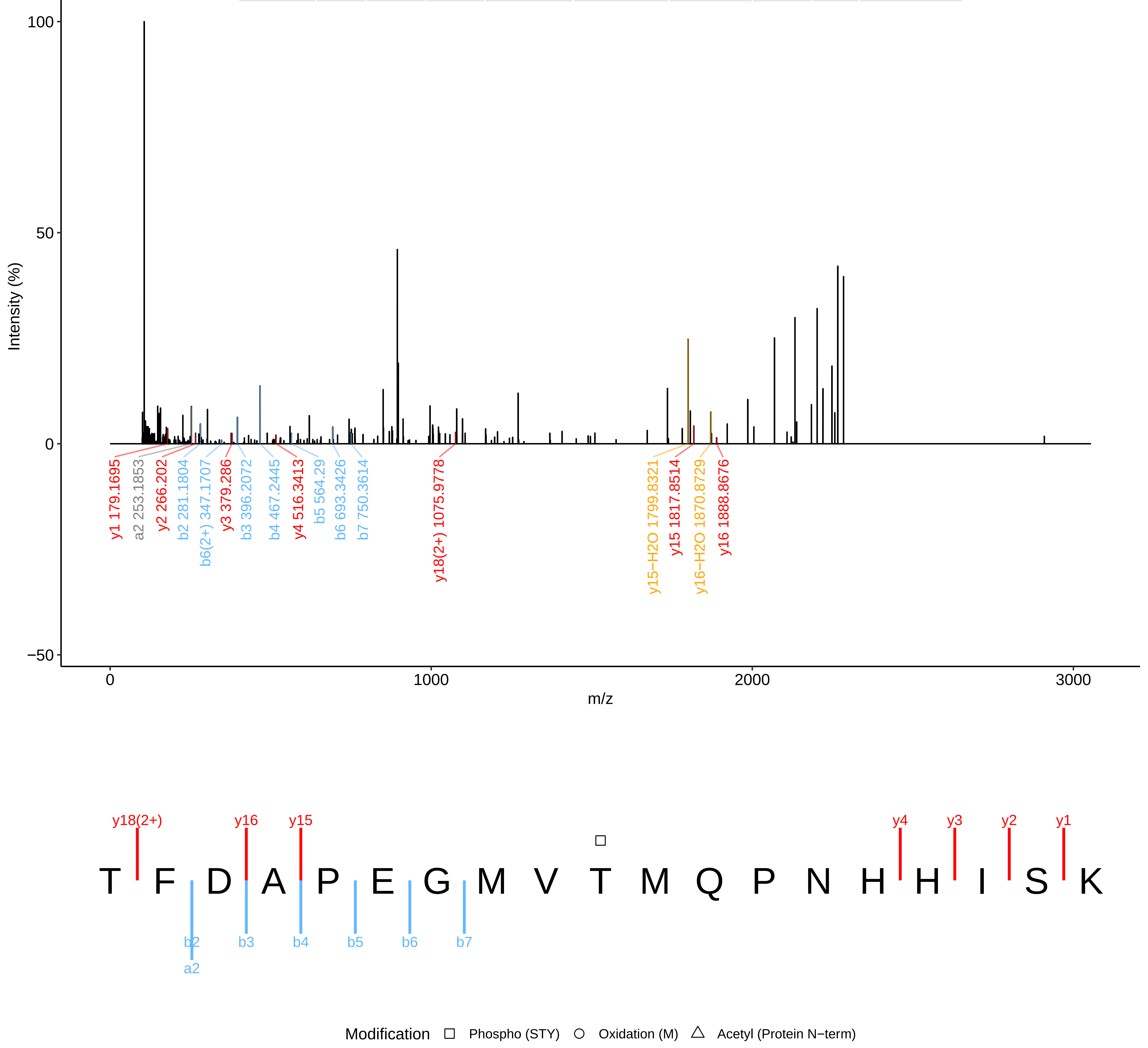

20210828_PS_SCy031_phospho_R3_TiO2_LC24h_F2 − Scan number: 5714

Gene Names	Charge	m/z	Mass	Mass error [Da]	Mass error [ppm]	Retention time	PEP	Score	Precursor Intensity
slr0476	2	1056.999	2111.983	0.0016756	1.5852	10.208	1.3528e−14	162.15	3177458

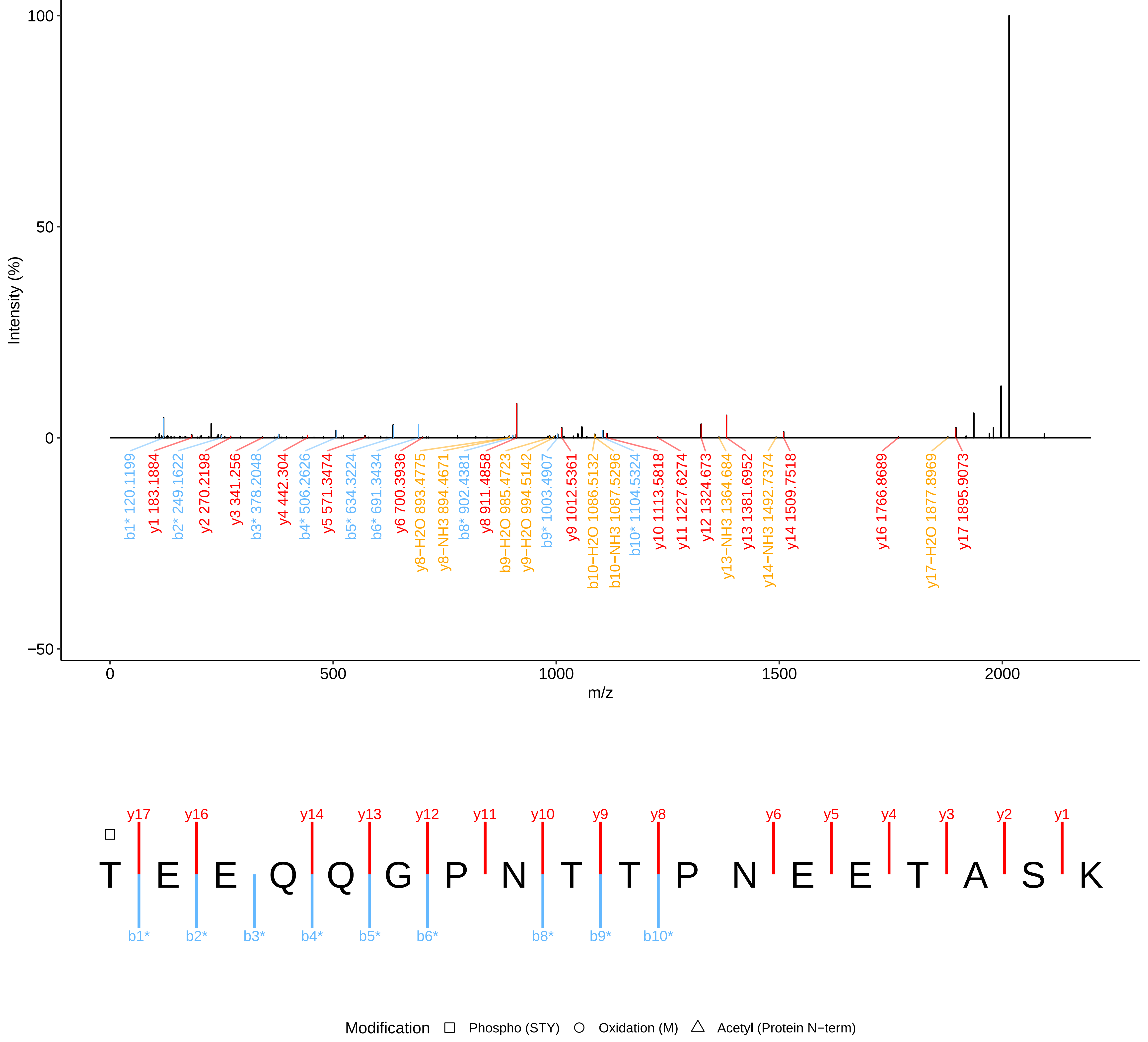

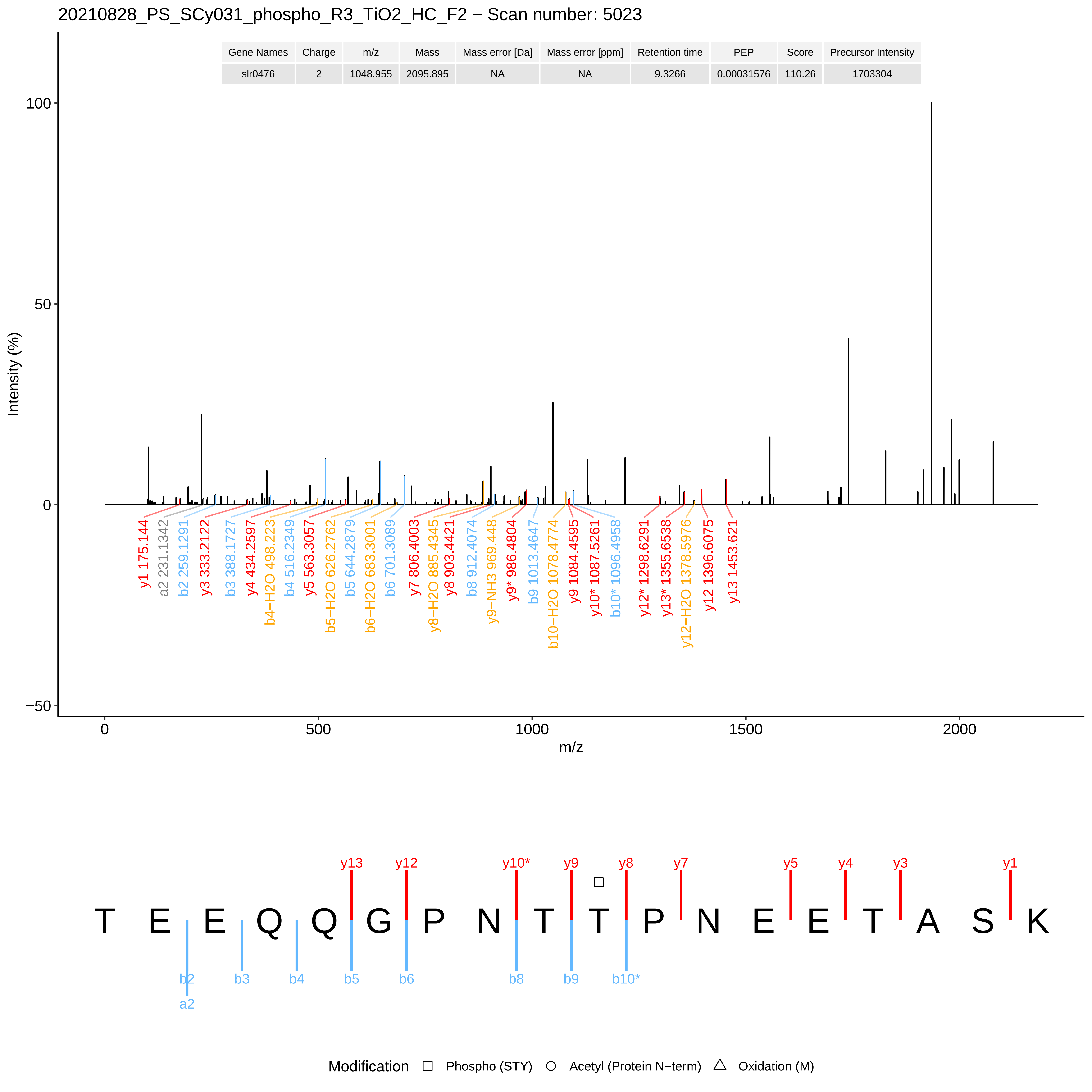

20210828_PS_SCy031_phospho_R3_TiO2_HC_F2 − Scan number: 5023

Gene Names	Charge	m/z	Mass	Mass error [Da]	Mass error [ppm]	Retention time	PEP	Score	Precursor Intensity
slr0476	2	1048.955	2095.895	NA	NA	9.3266	0.00031576	110.26	1703304

Intensity (%)
m/z
y1 175.144
a2 231.1342
b2 259.1291
y3 333.2122
b3 388.1727
y4 434.2597
b4−H2O 498.223
b4 516.2349
y5 563.3057
b5−H2O 626.2762
b5 644.2879
b6−H2O 683.3001
b6 701.3089
y7 806.4003
y8−H2O 885.4345
y8 903.4421
b8 912.4074
y9−NH3 969.448
y9* 986.4804
b9 1013.4647
b10−H2O 1078.4774
y9 1084.4595
y10* 1087.5261
b10* 1096.4958
y12* 1298.6291
y13* 1355.6538
y12−H2O 1378.5976
y12 1396.6075
y13 1453.621
T E E Q Q G P N T T P N E E T A S K
y13 y12 y10* y9 y8 y7 y5 y4 y3 y1
b2 b3 b4 b5 b6 b8 b9 b10*
a2
Modification
Phospho (STY)
Acetyl (Protein N−term)
Oxidation (M)

20210828_PS_SCy031_phospho_R3_TiO2_LC3h_F1 − Scan number: 5119

Gene Names	Charge	m/z	Mass	Mass error [Da]	Mass error [ppm]	Retention time	PEP	Score	Precursor Intensity
slr0476	2	1052.98	2103.945	0.00036402	0.35656	9.3515	0	418.61	11141058

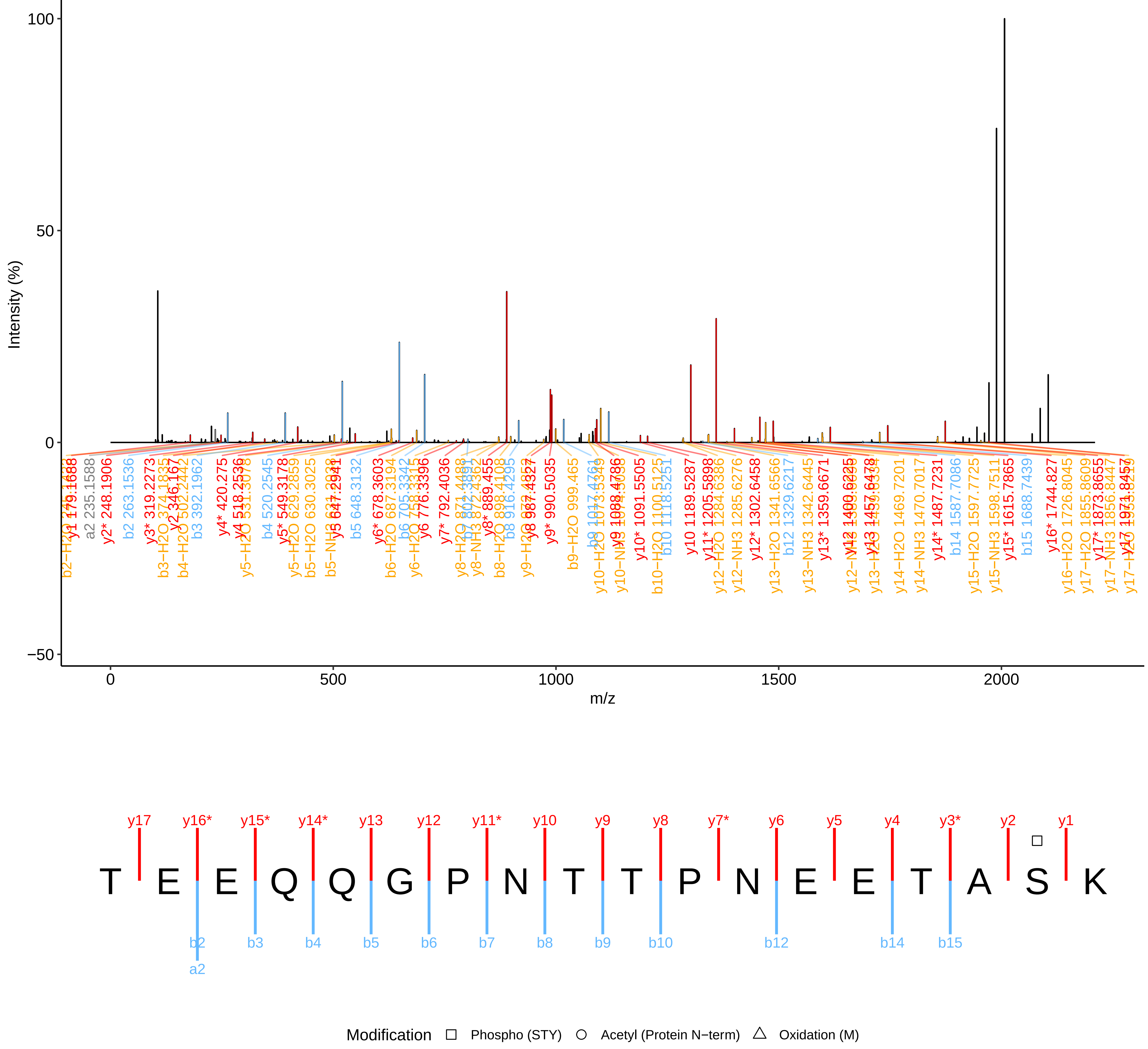

20210828_PS_SCy031_phospho_R2_TiO2_LC24h_F3 − Scan number: 15443

Gene Names	Charge	m/z	Mass	Mass error [Da]	Mass error [ppm]	Retention time	PEP	Score	Precursor Intensity
slr0483	2	752.3764	1502.738	−0.001442	−1.9166	22.451	0.013502	99.927	3133046

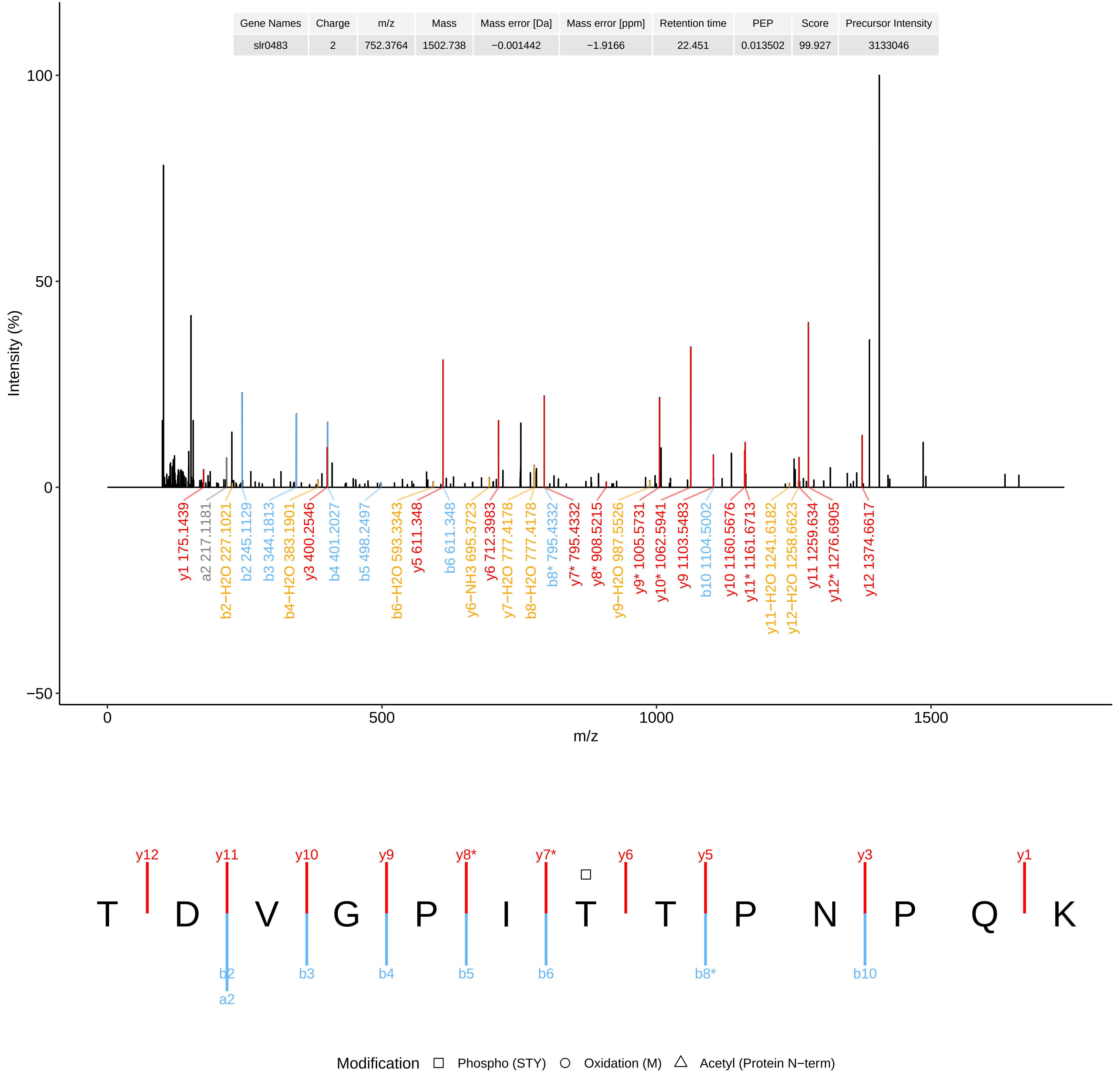

20210823_PS_SCy031_R1_phospho_LC24h_TiO2_2 − Scan number: 14182

Gene Names	Charge	m/z	Mass	Mass error [Da]	Mass error [ppm]	Retention time	PEP	Score	Precursor Intensity
slr0549	2	724.8156	1447.617	NA	NA	20.784	0.0095521	59.997	3045180

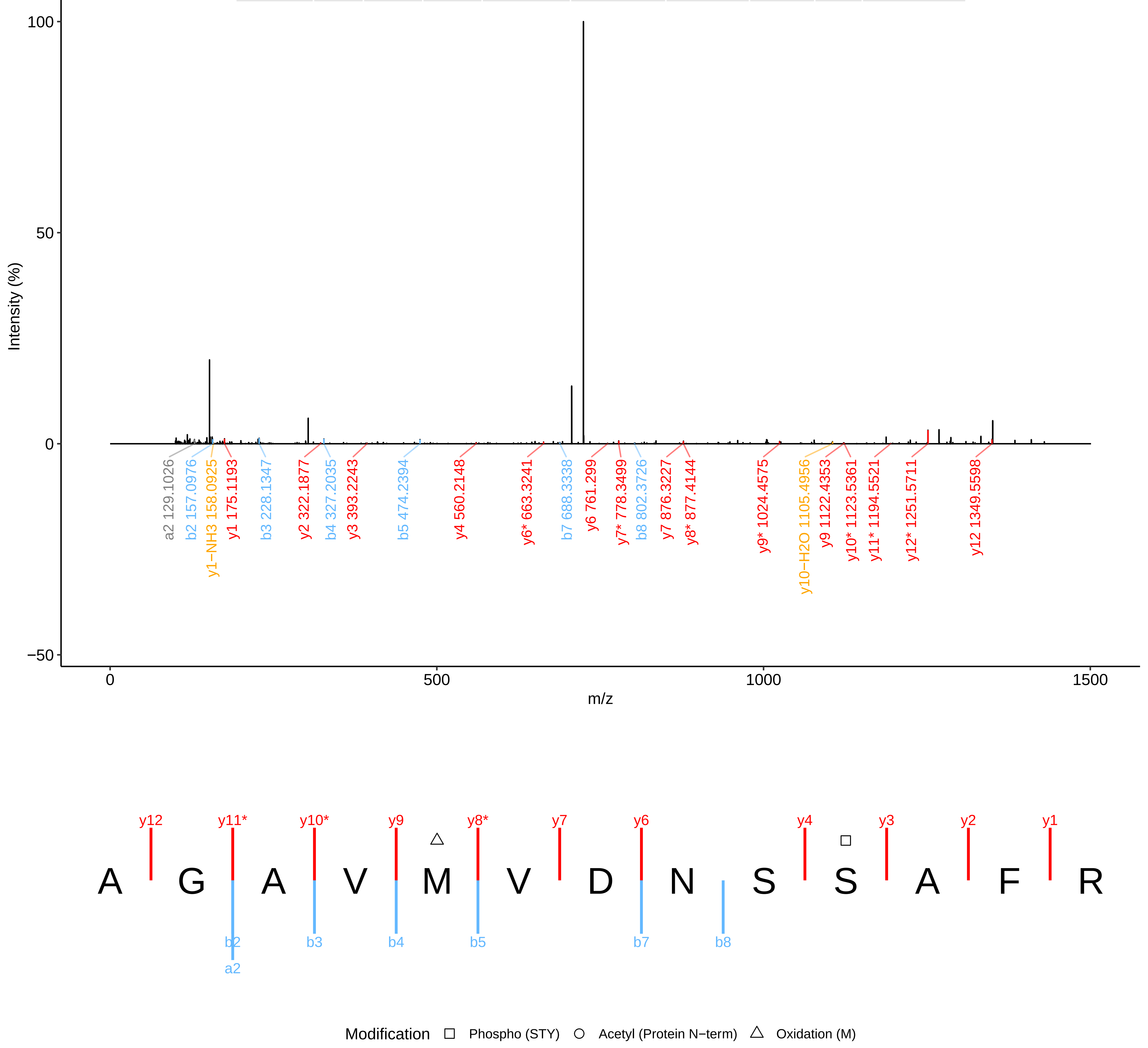

20210828_PS_SCy031_phospho_R3_TiO2_LC3h_F3 − Scan number: 27537

Gene Names	Charge	m/z	Mass	Mass error [Da]	Mass error [ppm]	Retention time	PEP	Score	Precursor Intensity
slr0552	2	740.8669	1479.719	0.00087722	1.2136	37.809	0.012617	77.597	4927213

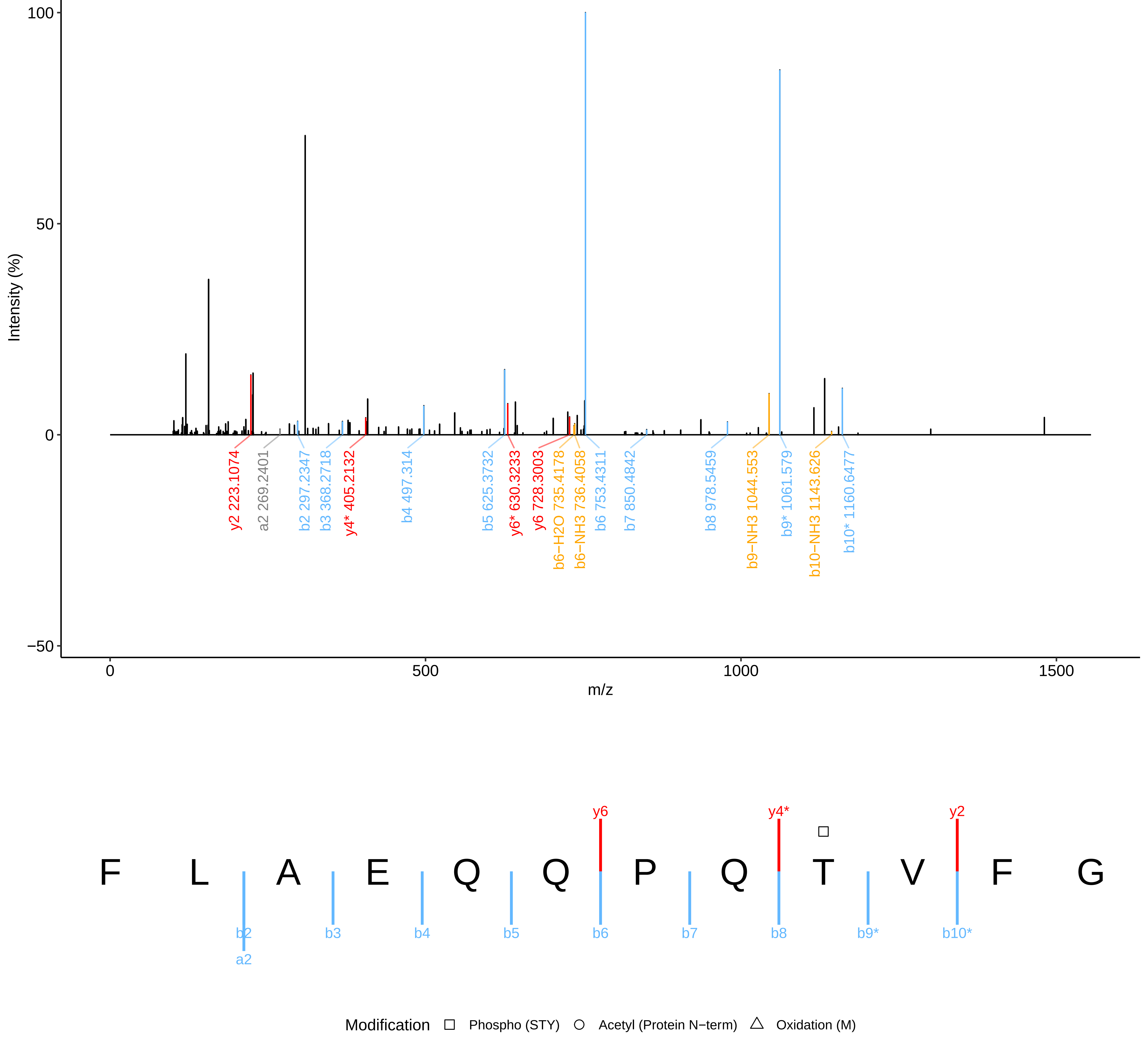

20210828_PS_SCy031_phospho_R3_TiO2_LC3h_F2 − Scan number: 10321

Gene Names	Charge	m/z	Mass	Mass error [Da]	Mass error [ppm]	Retention time	PEP	Score	Precursor Intensity
slr0585	2	518.7361	1035.458	0.00092873	1.8475	15.958	0.011873	77.192	6623852

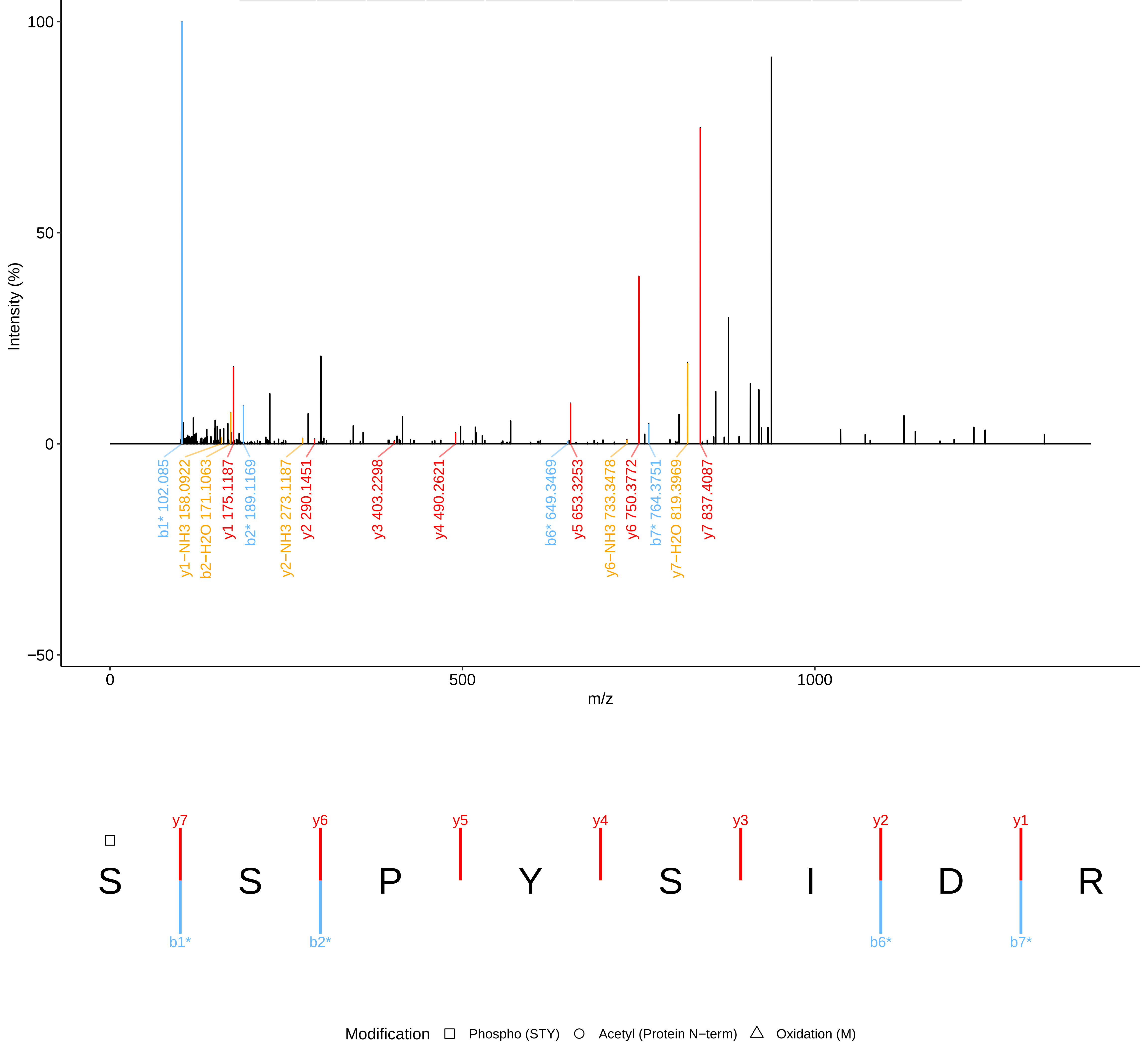

20210828_PS_SCy031_phospho_R3_TiO2_LC3h_F3 − Scan number: 18470

Gene Names	Charge	m/z	Mass	Mass error [Da]	Mass error [ppm]	Retention time	PEP	Score	Precursor Intensity
slr0599	3	926.4742	2776.401	NA	NA	26.317	4.094e−08	94.646	NA

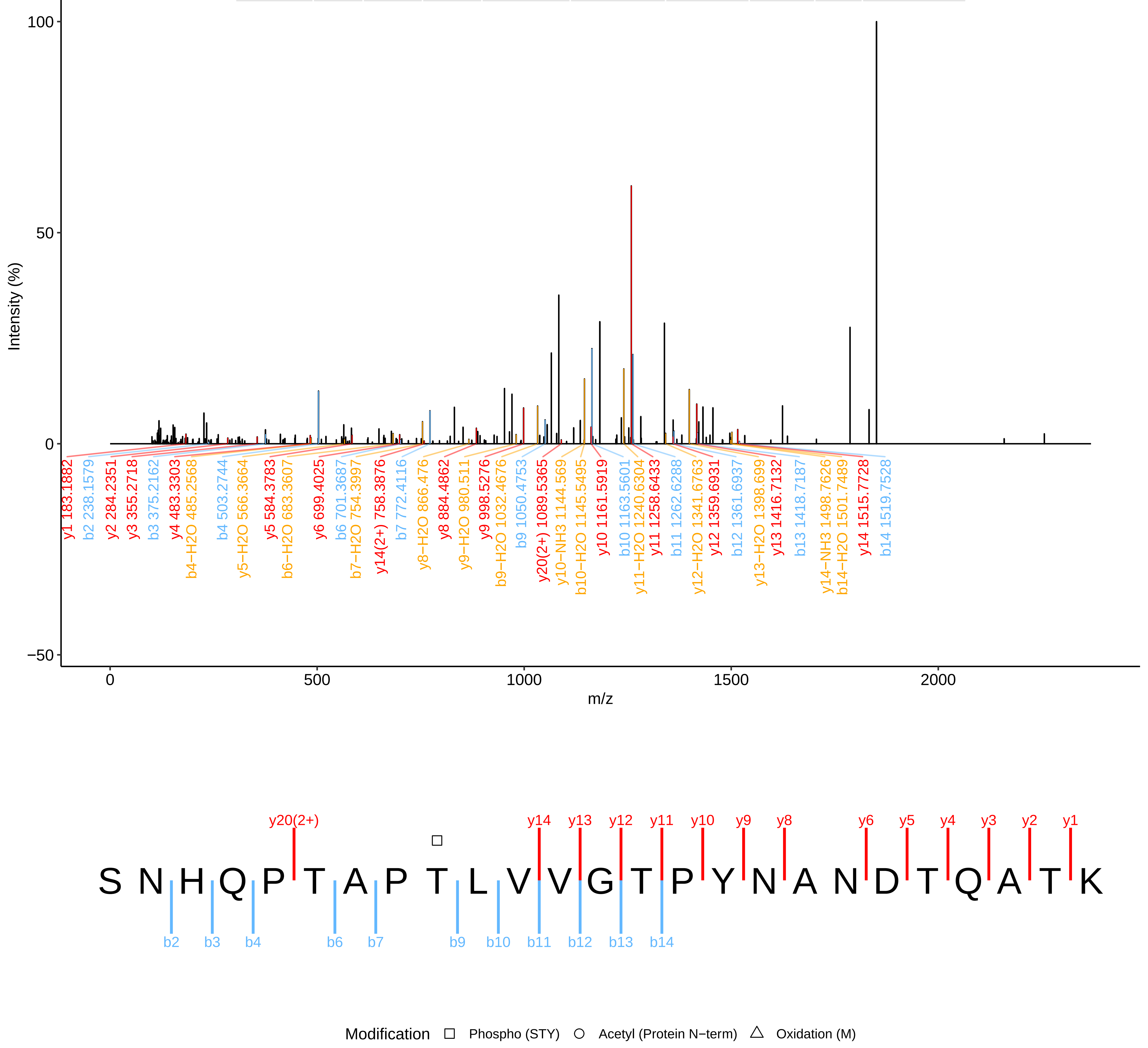

20210823_PS_SCy031_R1_phospho_LC3h_TiO2_2 − Scan number: 18545

Gene Names	Charge	m/z	Mass	Mass error [Da]	Mass error [ppm]	Retention time	PEP	Score	Precursor Intensity
slr0599	3	923.7947	2768.362	NA	NA	26.325	5.5498e−13	101.86	NA

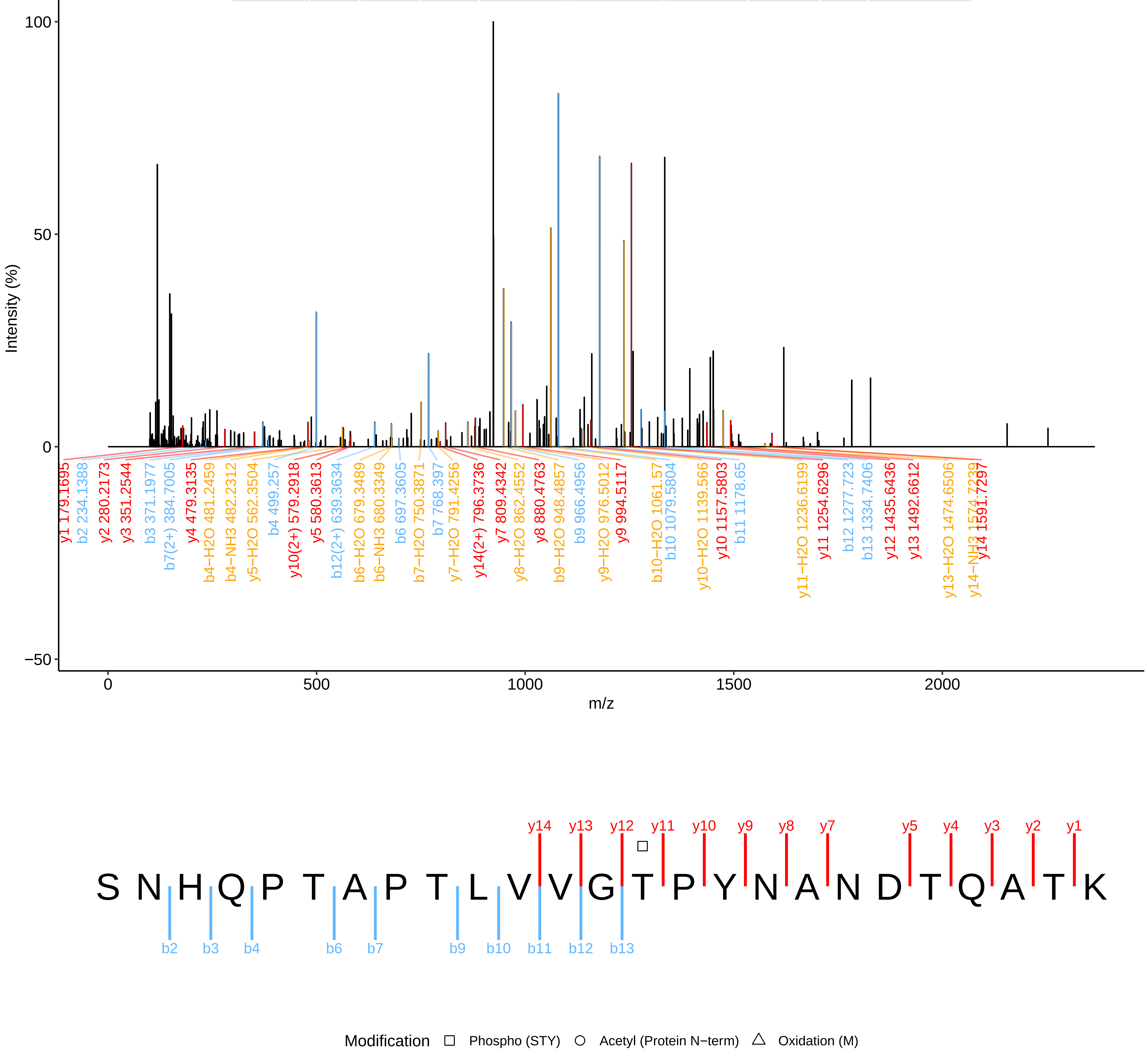

20210828_PS_SCy031_phospho_R3_TiO2_HC_F2 − Scan number: 18501

Gene Names	Charge	m/z	Mass	Mass error [Da]	Mass error [ppm]	Retention time	PEP	Score	Precursor Intensity
slr0599	3	923.7947	2768.362	0.000461	0.51085	26.223	6.1571e−66	179.85	10962599

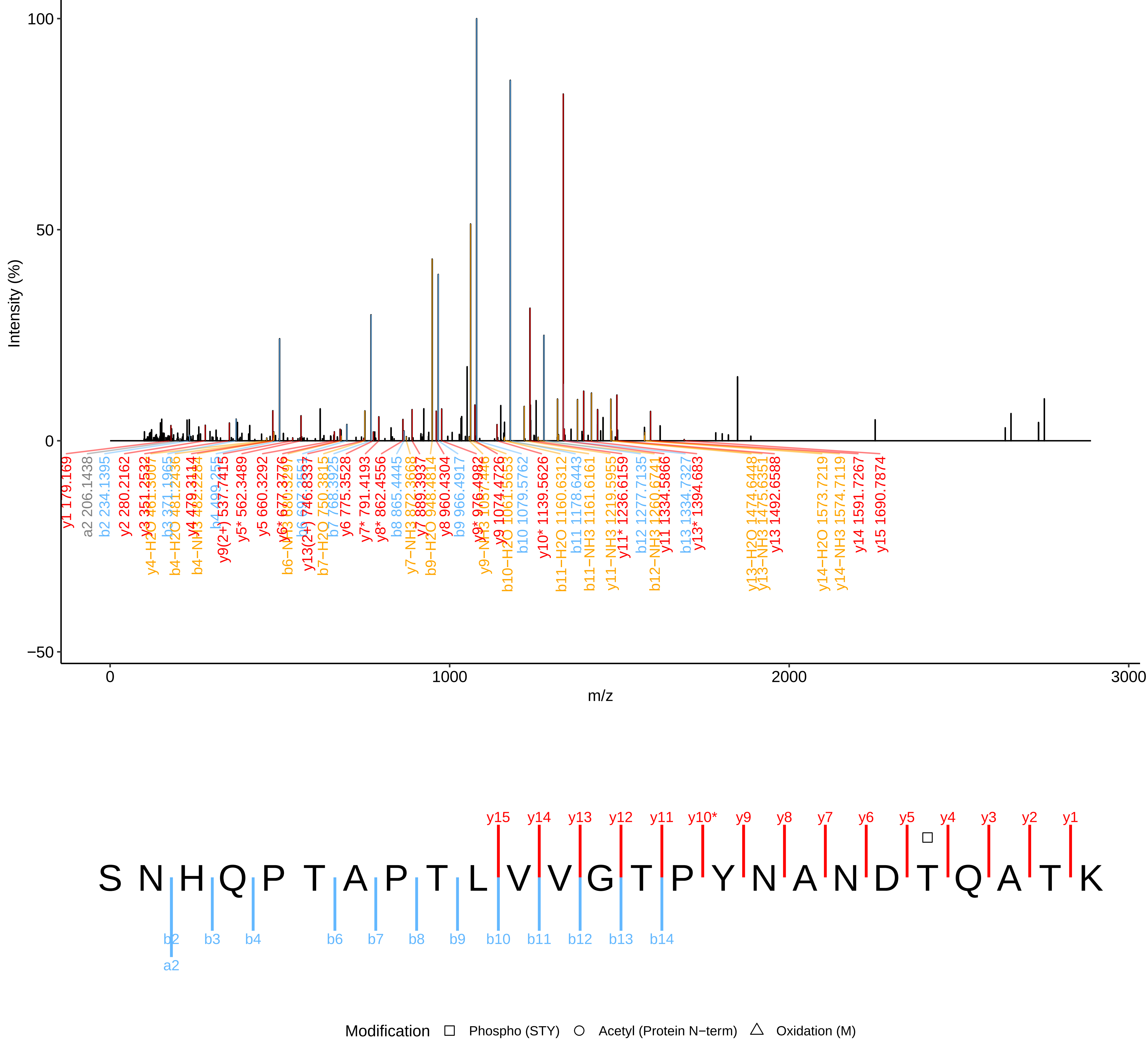

20210823_PS_SCy031_R1_phospho_LC24h_TiO2_1 − Scan number: 16183

Gene Names	Charge	m/z	Mass	Mass error [Da]	Mass error [ppm]	Retention time	PEP	Score	Precursor Intensity
slr0623	2	952.938	1903.861	NA	NA	23.014	0.0035382	90.385	NA

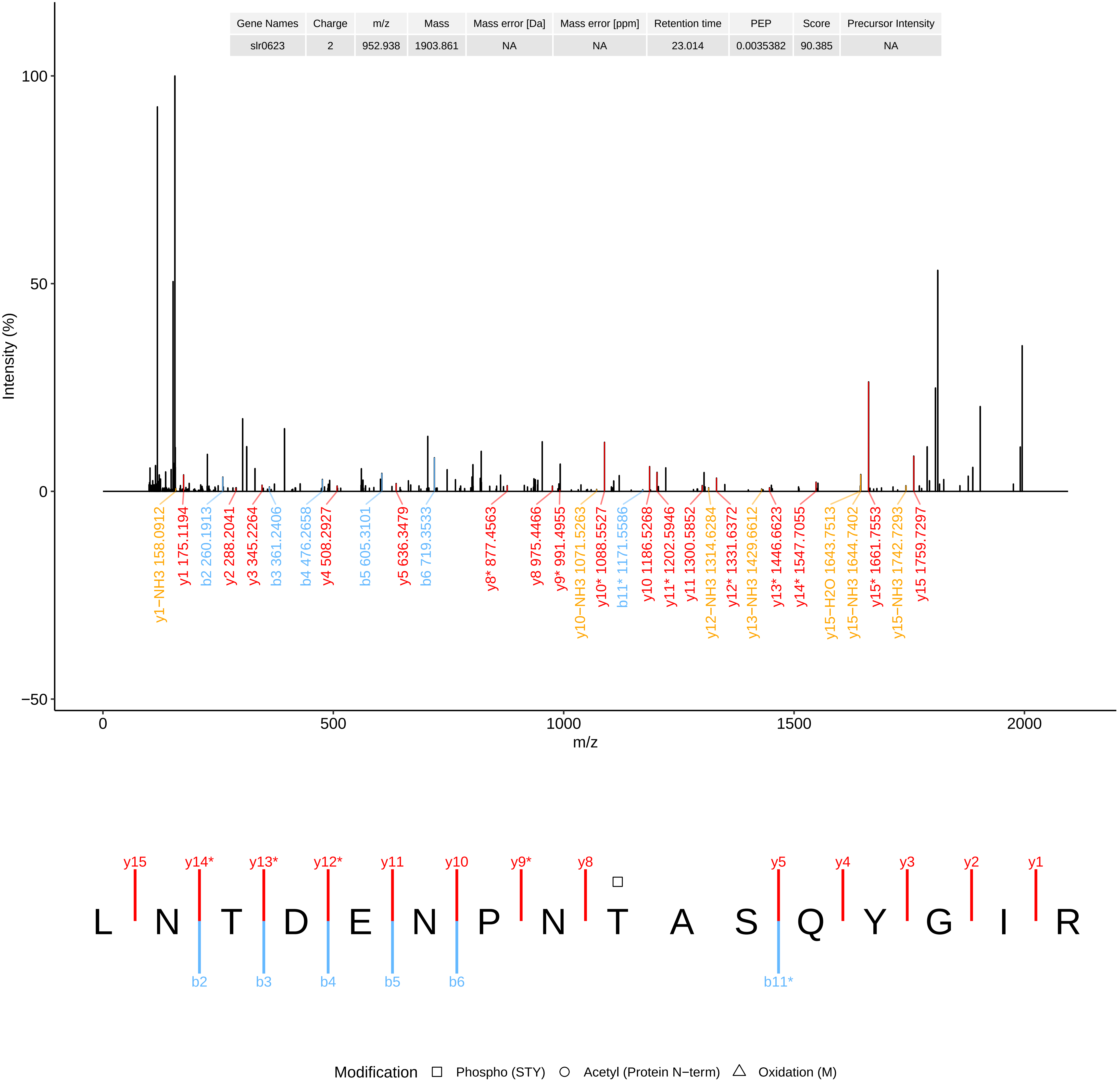

20210828_PS_SCy031_phospho_R3_TiO2_HC_F2 − Scan number: 25239

Gene Names	Charge	m/z	Mass	Mass error [Da]	Mass error [ppm]	Retention time	PEP	Score	Precursor Intensity
slr0700	2	819.3984	1636.782	NA	NA	34.712	0.00023913	115.09	NA

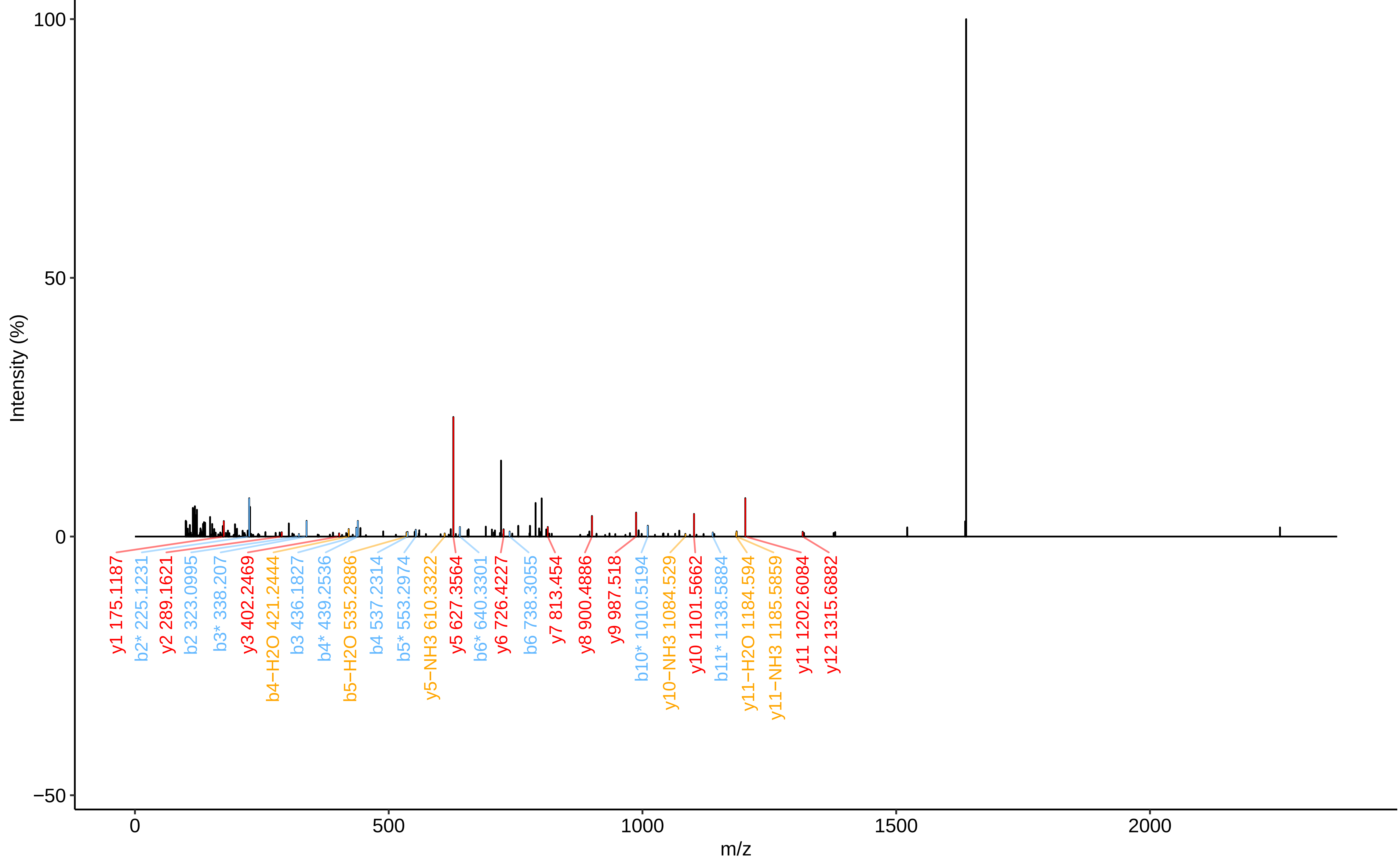

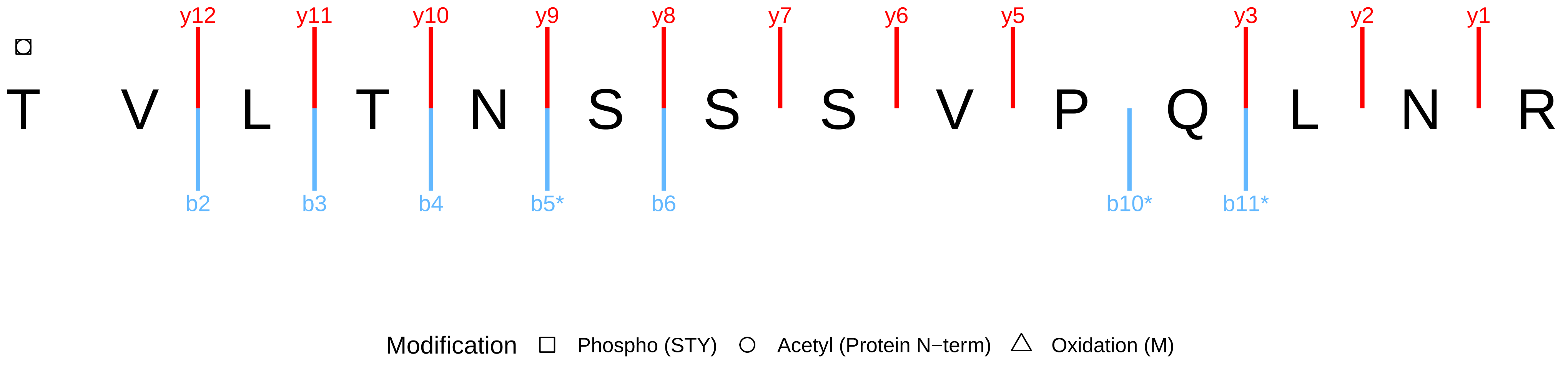

20210828_PS_SCy031_phospho_R2_TiO2_LC24h_F3 − Scan number: 7358

Gene Names	Charge	m/z	Mass	Mass error [Da]	Mass error [ppm]	Retention time	PEP	Score	Precursor Intensity
slr0737	2	628.3325	1254.65	−4.6353e−05	−0.073771	12.278	0.0002039	137.05	11007560

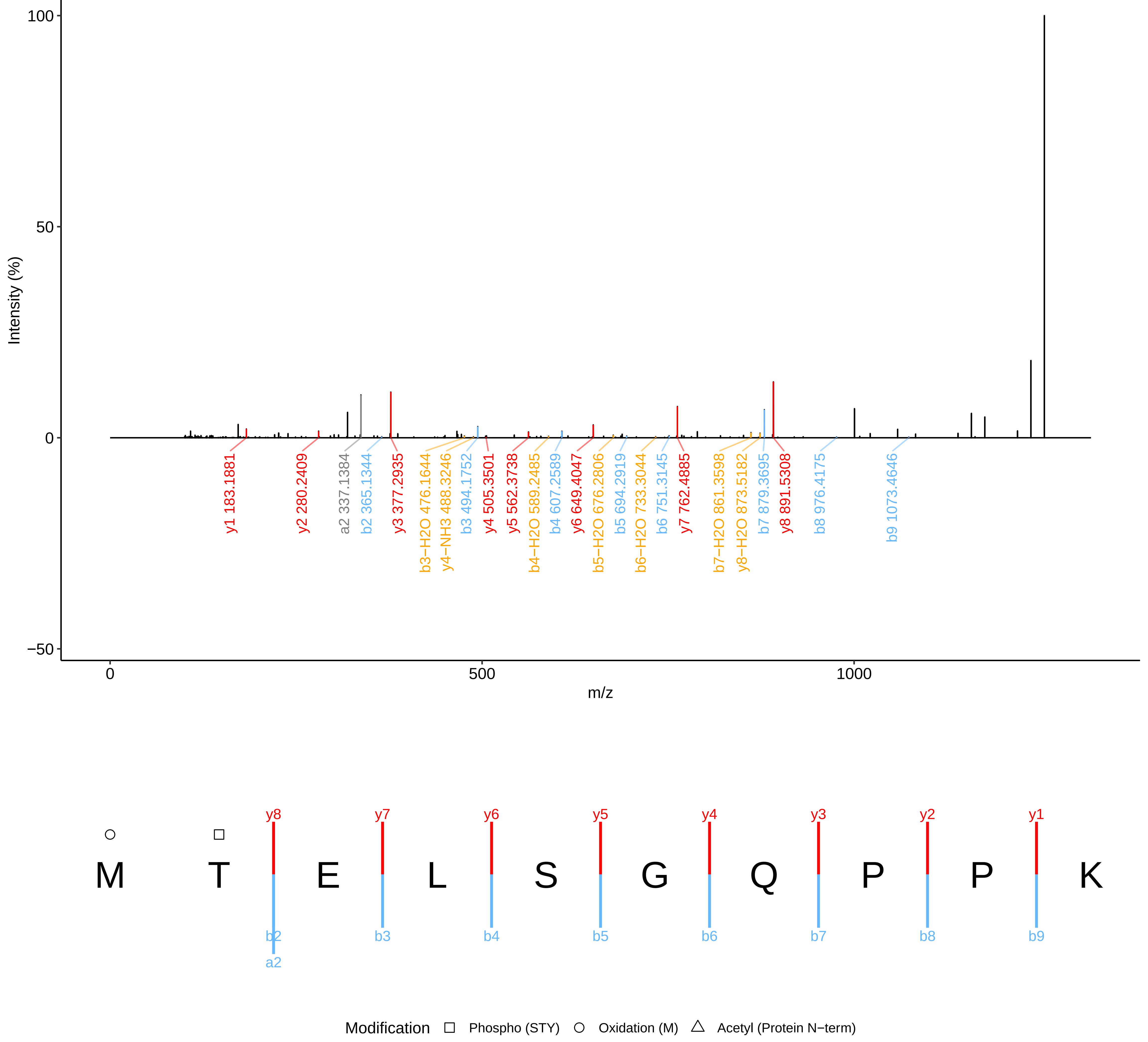

20210823_PS_SCy031_R1_phospho_LC24h_TiO2_3 − Scan number: 7579

Gene Names	Charge	m/z	Mass	Mass error [Da]	Mass error [ppm]	Retention time	PEP	Score	Precursor Intensity
slr0737	2	546.7705	1091.526	−0.00016577	−0.31957	12.543	2.7358e−06	111.86	3263178

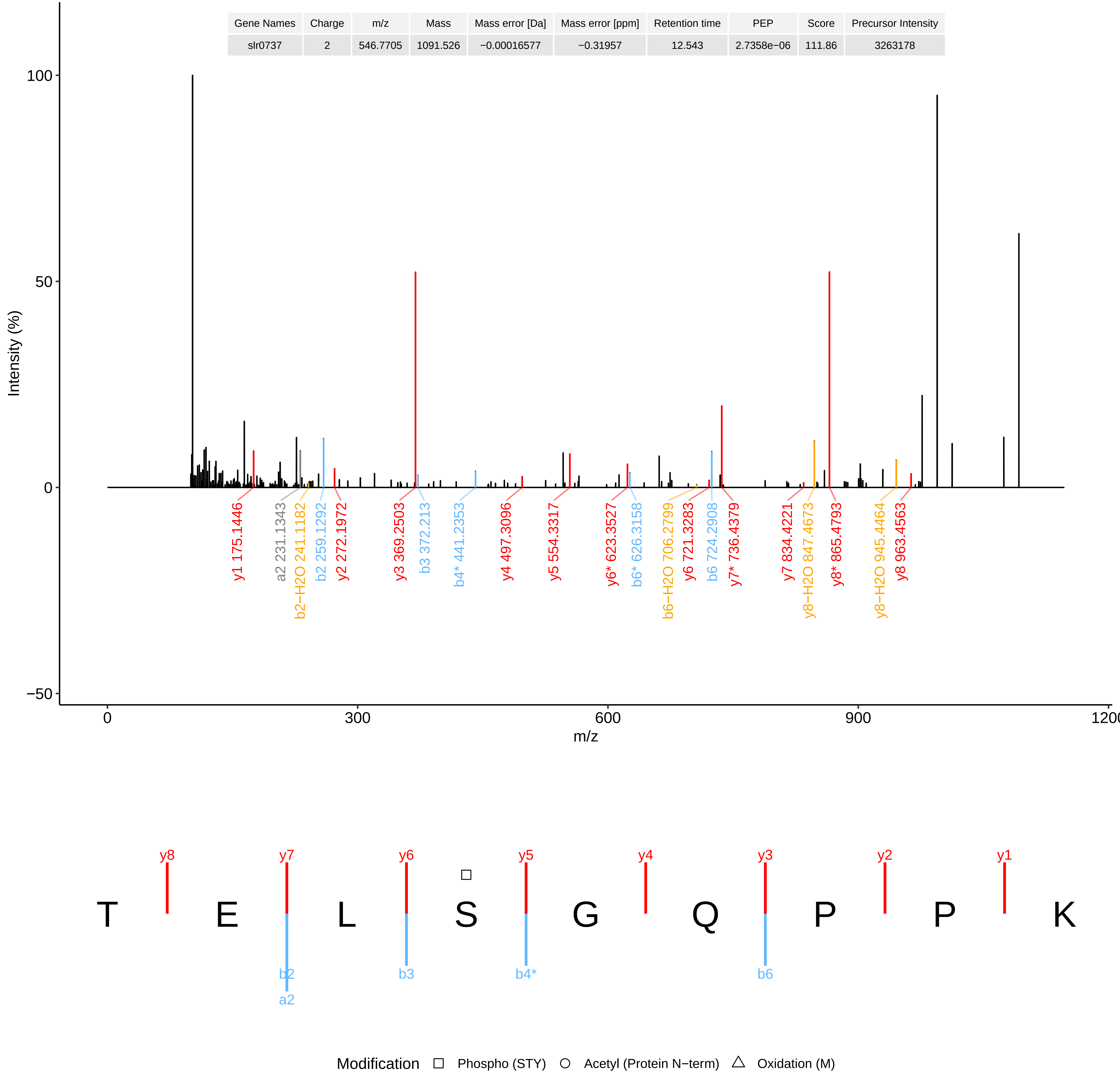

20210828_PS_SCy031_phospho_R2_TiO2_LC24h_F3 − Scan number: 20204

Gene Names	Charge	m/z	Mass	Mass error [Da]	Mass error [ppm]	Retention time	PEP	Score	Precursor Intensity
slr0737	2	588.3359	1174.657	−0.0001722	−0.31181	28.462	0.0016437	87.498	2619715

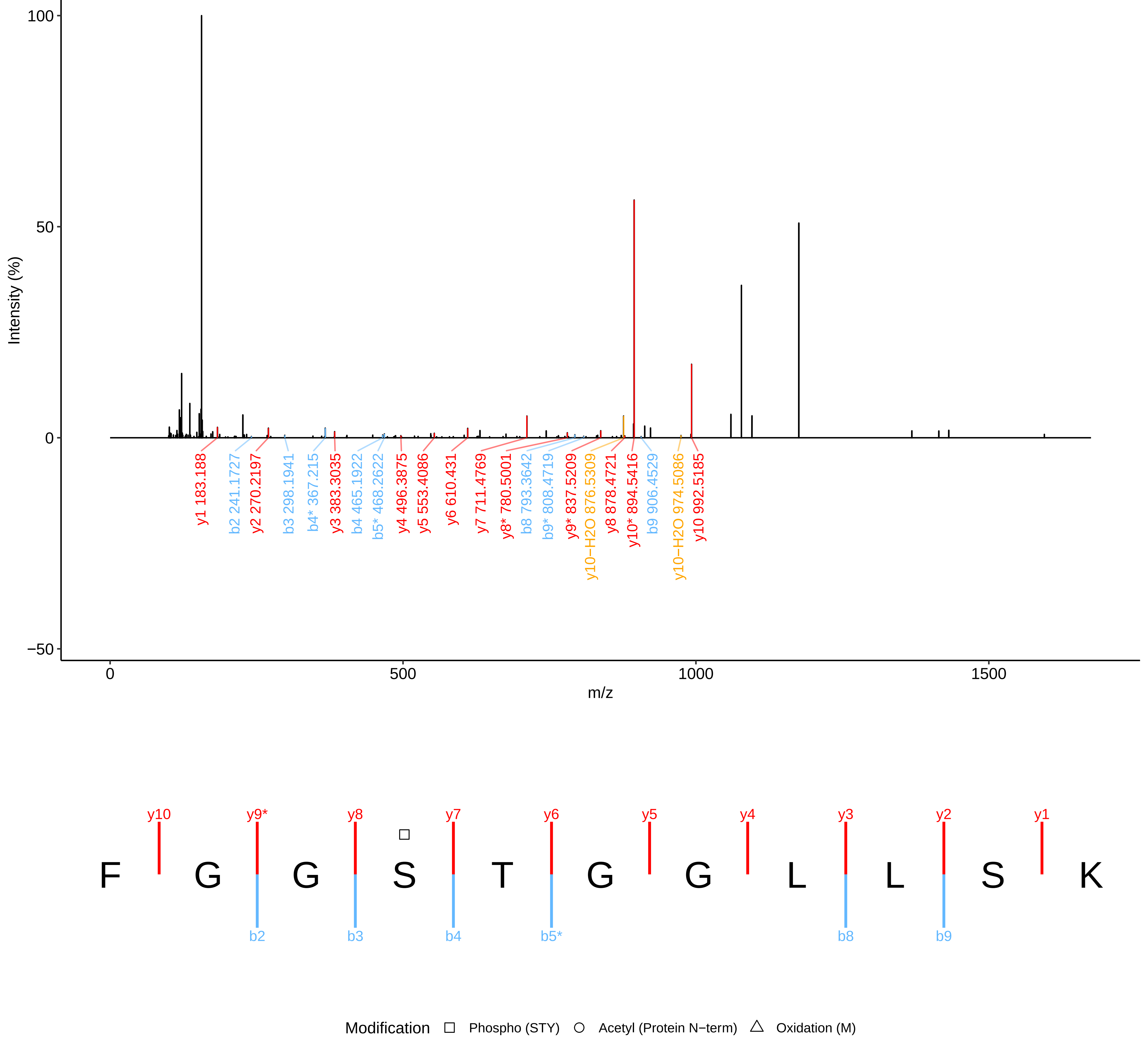

20210828_PS_SCy031_phospho_R3_TiO2_HC_F3 − Scan number: 19479

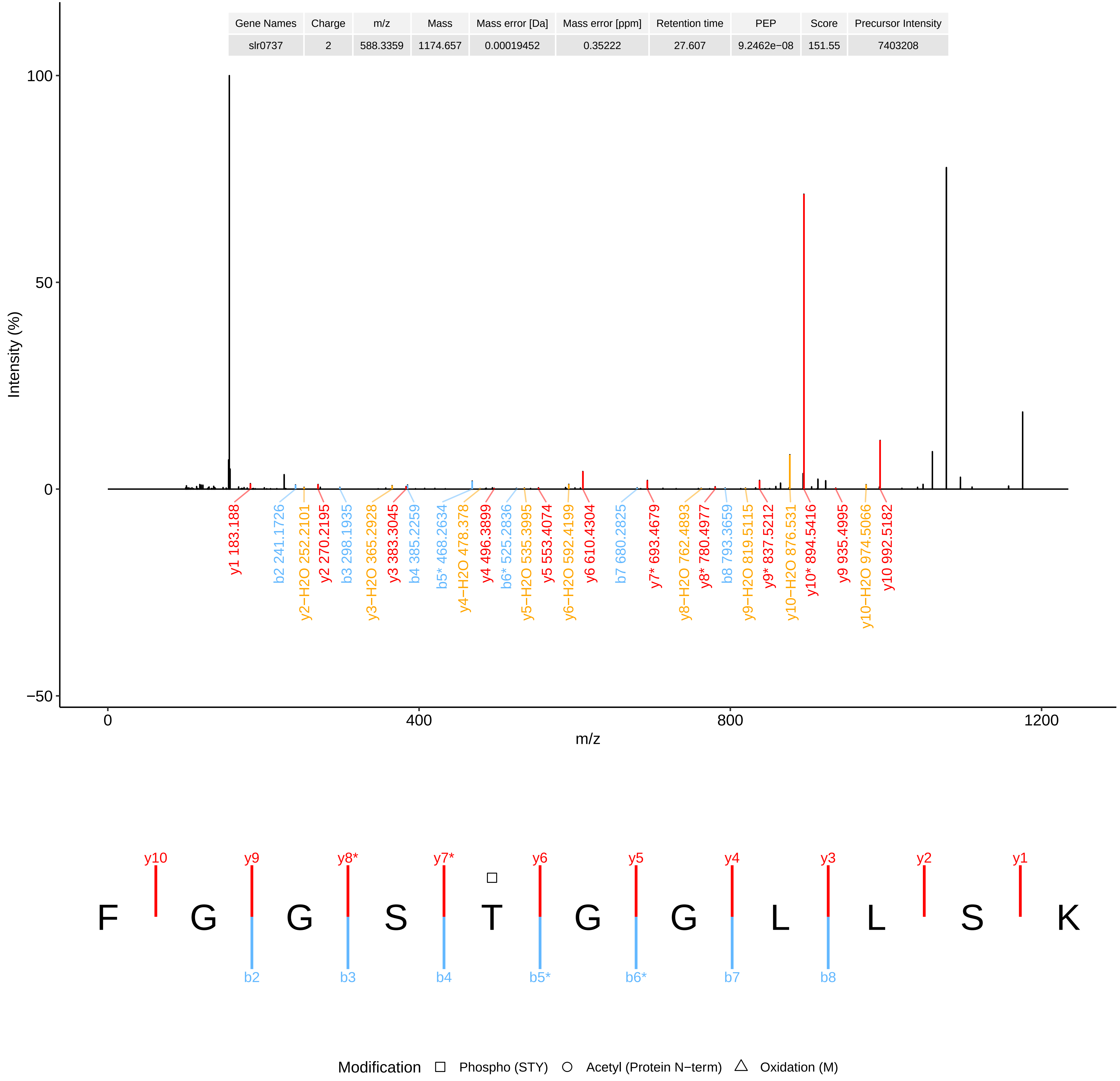

Gene Names	Charge	m/z	Mass	Mass error [Da]	Mass error [ppm]	Retention time	PEP	Score	Precursor Intensity
slr0737	2	588.3359	1174.657	0.00019452	0.35222	27.607	9.2462e−08	151.55	7403208

20210828_PS_SCy031_phospho_R2_TiO2_LC3h_F2 − Scan number: 19560

Gene Names	Charge	m/z	Mass	Mass error [Da]	Mass error [ppm]	Retention time	PEP	Score	Precursor Intensity
slr0737	2	588.3359	1174.657	−0.00036098	−0.65365	27.512	0.00033903	95.417	6691716

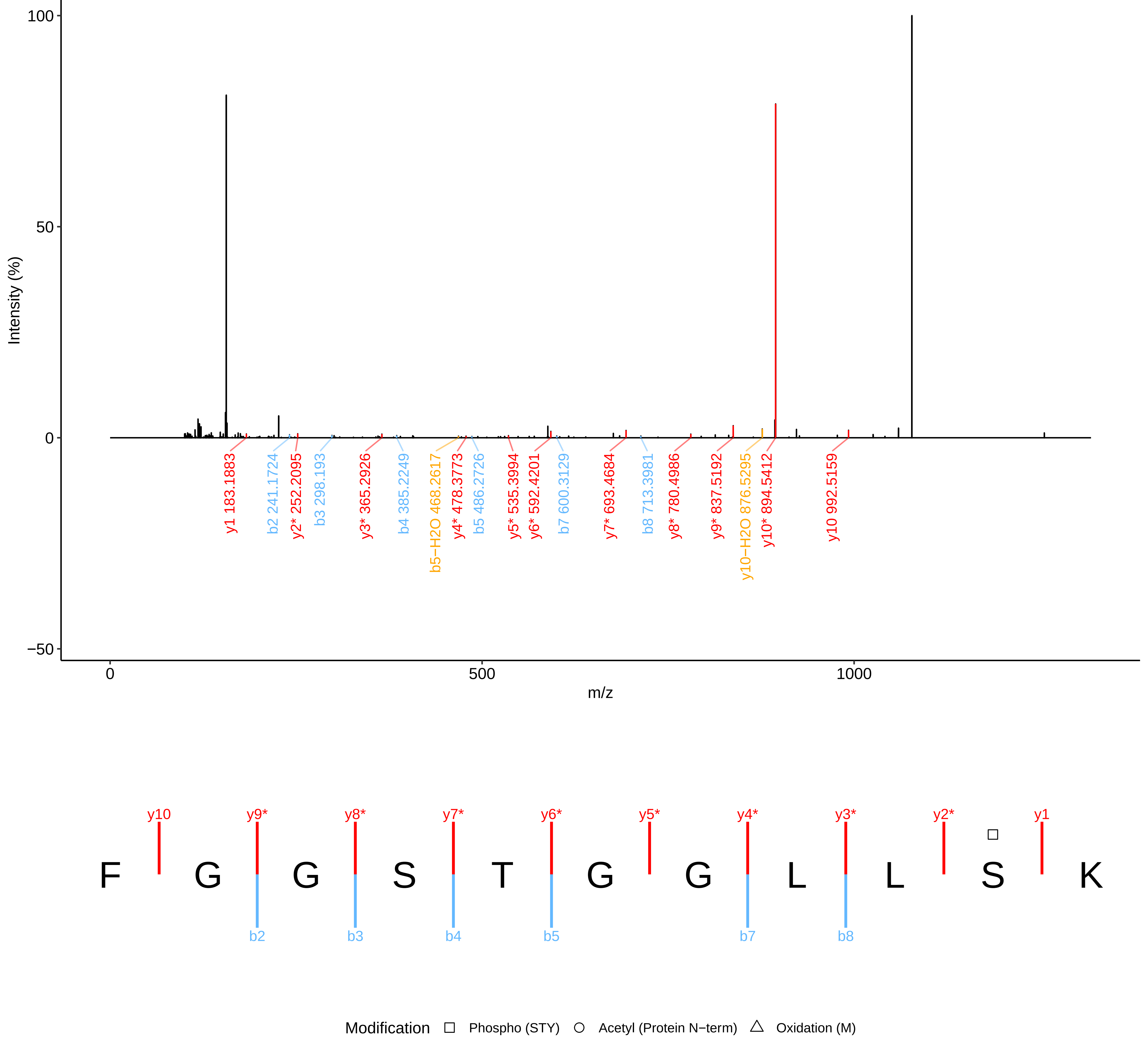

20210828_PS_SCy031_phospho_R3_TiO2_HC_F2 − Scan number: 19416

Gene Names	Charge	m/z	Mass	Mass error [Da]	Mass error [ppm]	Retention time	PEP	Score	Precursor Intensity
slr0737	3	597.6784	1790.013	0.0003081	0.54471	27.374	1.7928e−06	96.848	5549098

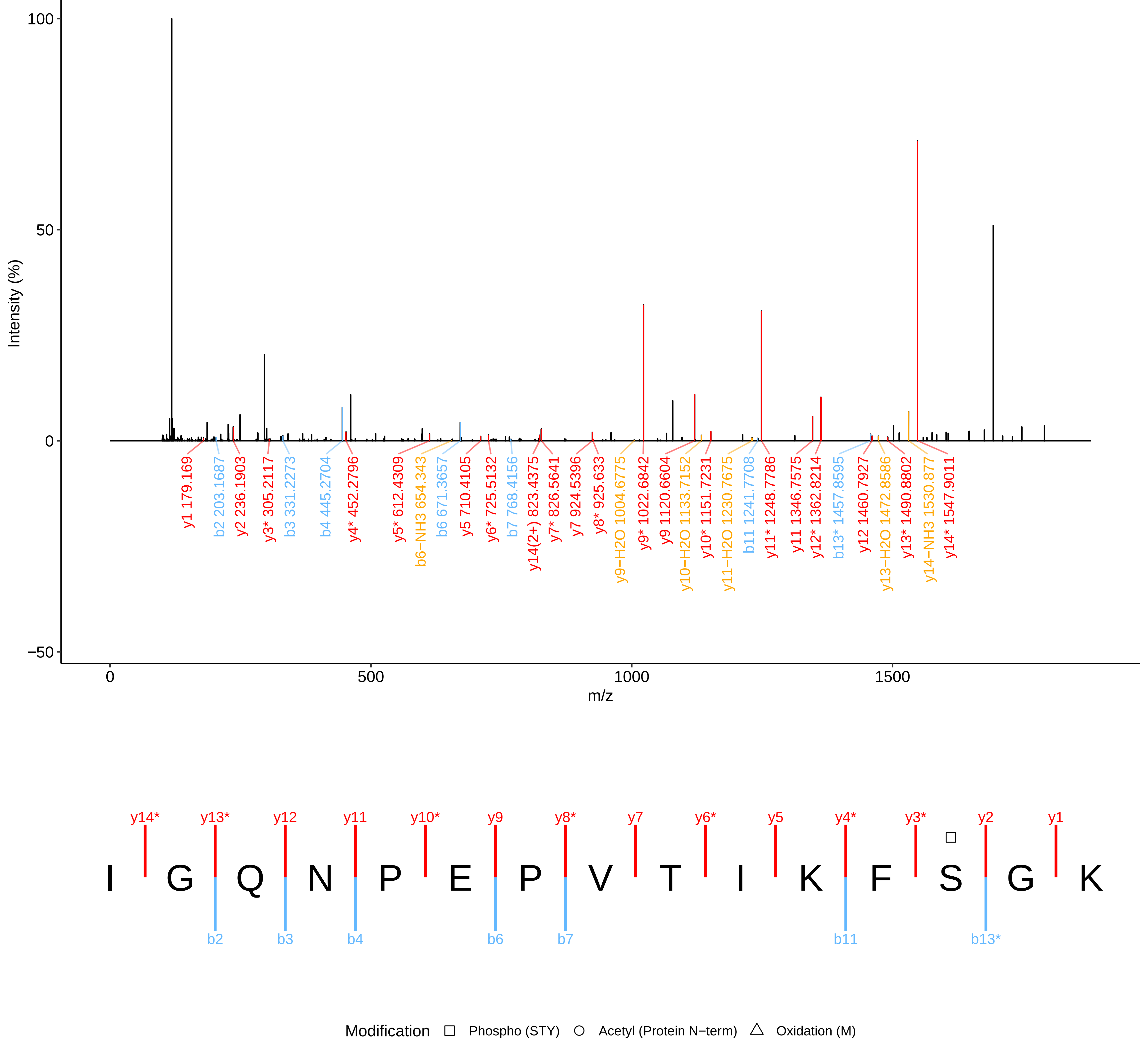

20210828_PS_SCy031_phospho_R3_TiO2_LC3h_F1 − Scan number: 17410

Gene Names	Charge	m/z	Mass	Mass error [Da]	Mass error [ppm]	Retention time	PEP	Score	Precursor Intensity
slr0875	2	780.8764	1559.738	4.8313e−05	0.061871	24.849	0.042299	88.42	3867736

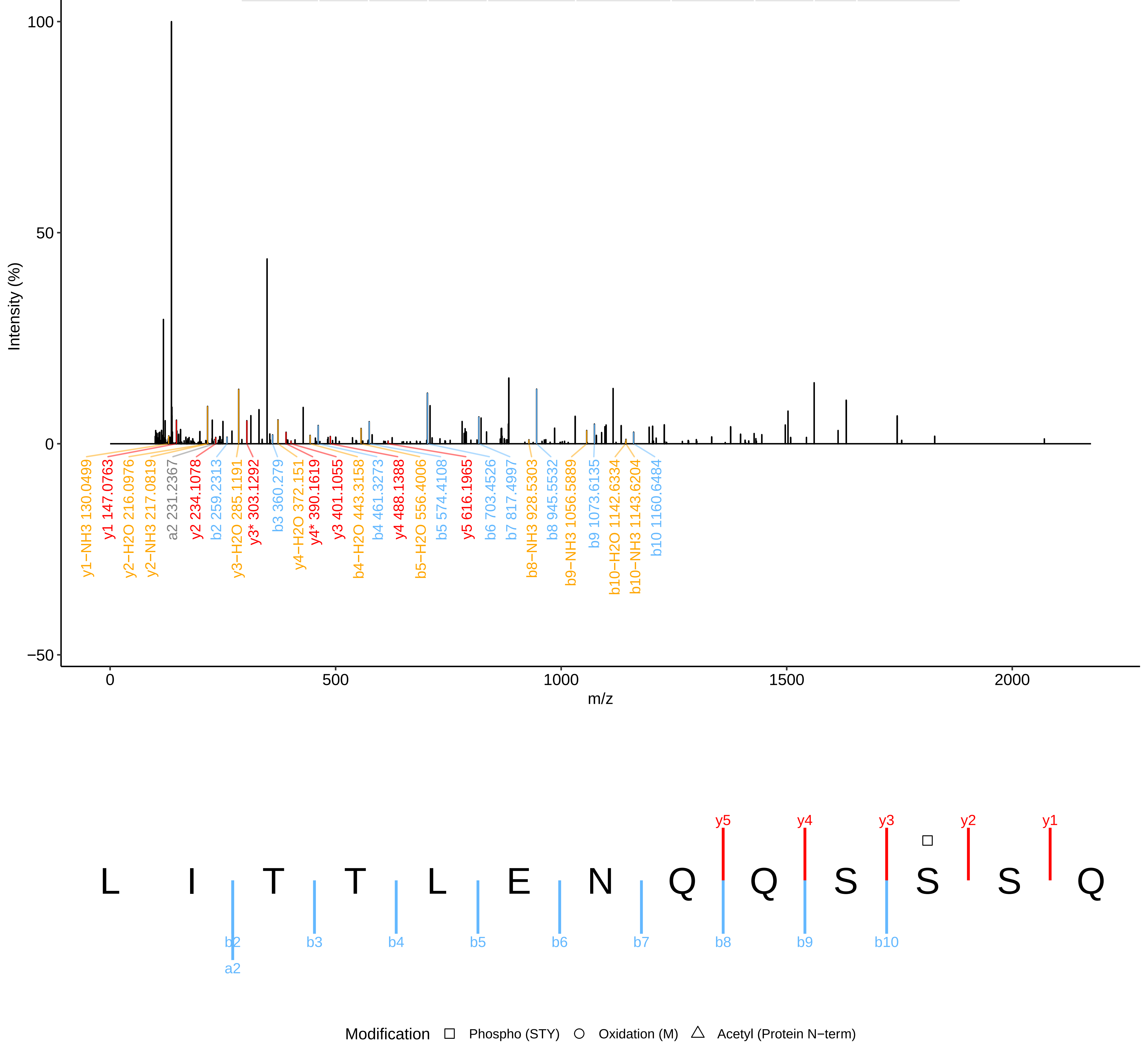

20210828_PS_SCy031_phospho_R3_TiO2_LC24h_F1 − Scan number: 17747

Gene Names	Charge	m/z	Mass	Mass error [Da]	Mass error [ppm]	Retention time	PEP	Score	Precursor Intensity
slr0875	2	782.886	1563.757	0.00095266	1.2169	25.085	0.0014997	137.09	2289801

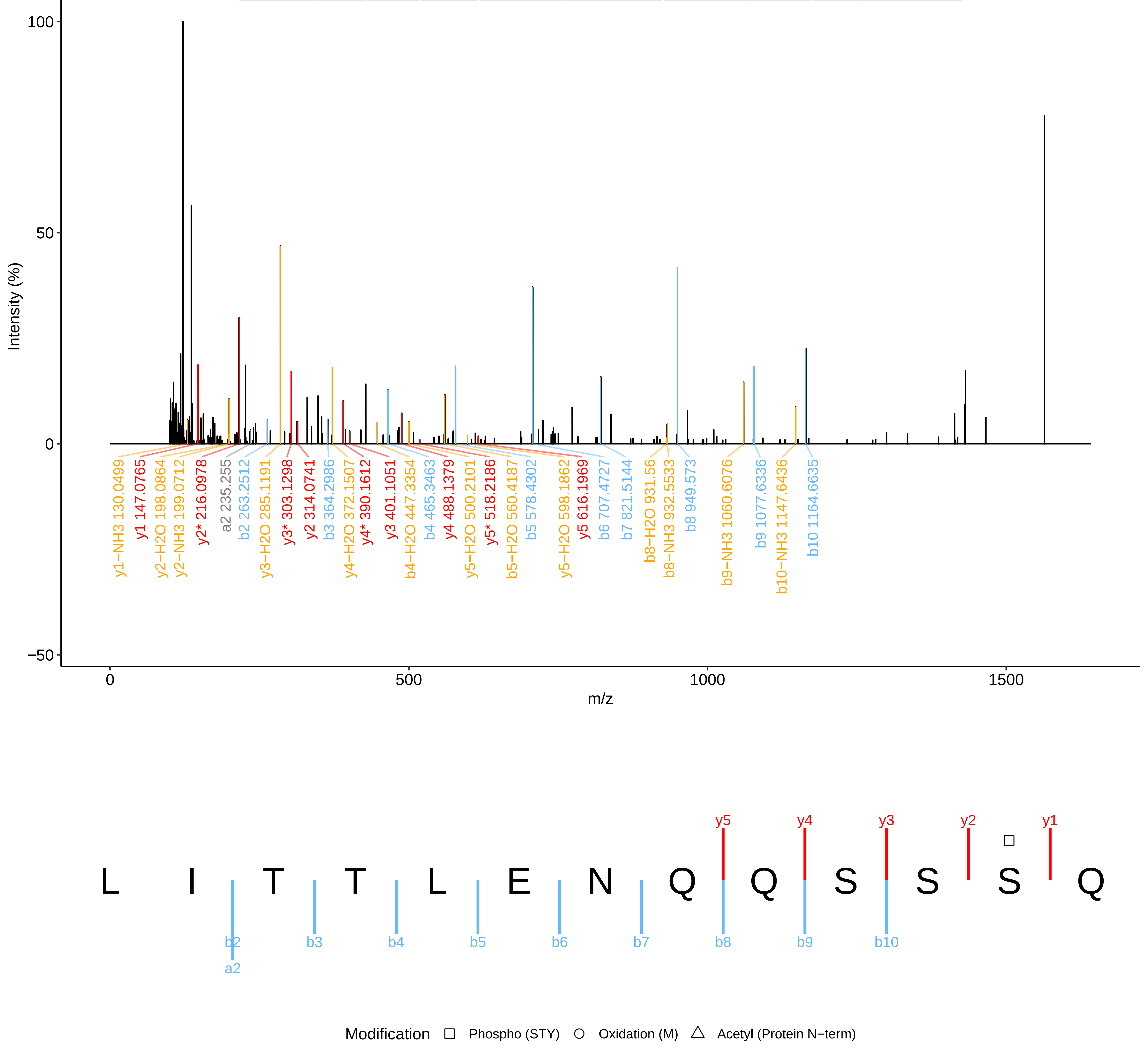

20210823_PS_SCy031_R1_phospho_LC3h_TiO2_1 − Scan number: 27079

Gene Names	Charge	m/z	Mass	Mass error [Da]	Mass error [ppm]	Retention time	PEP	Score	Precursor Intensity
slr0882	2	760.4001	1518.786	16.027	22005	36.871	0.00028209	78.334	12815478

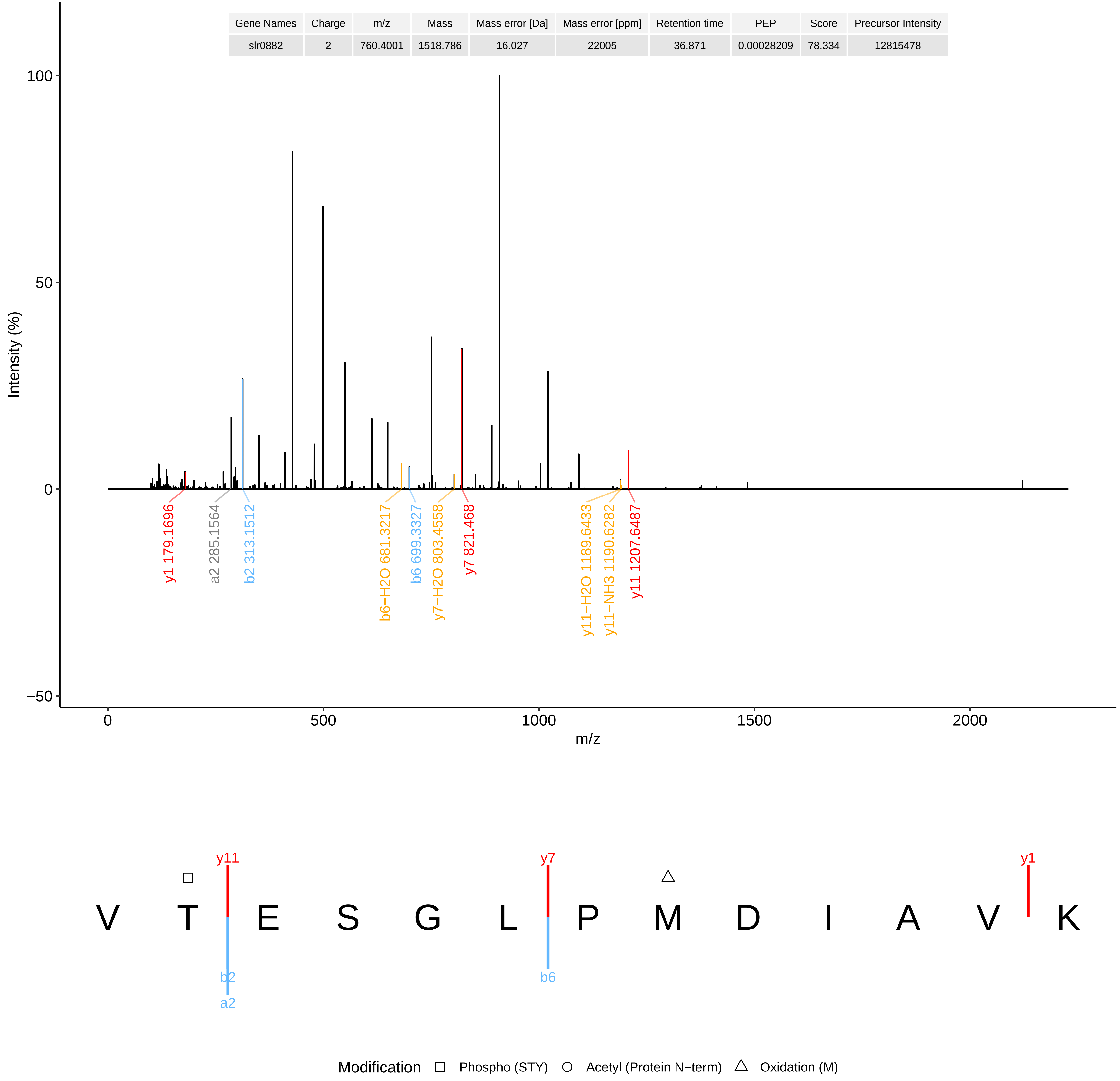

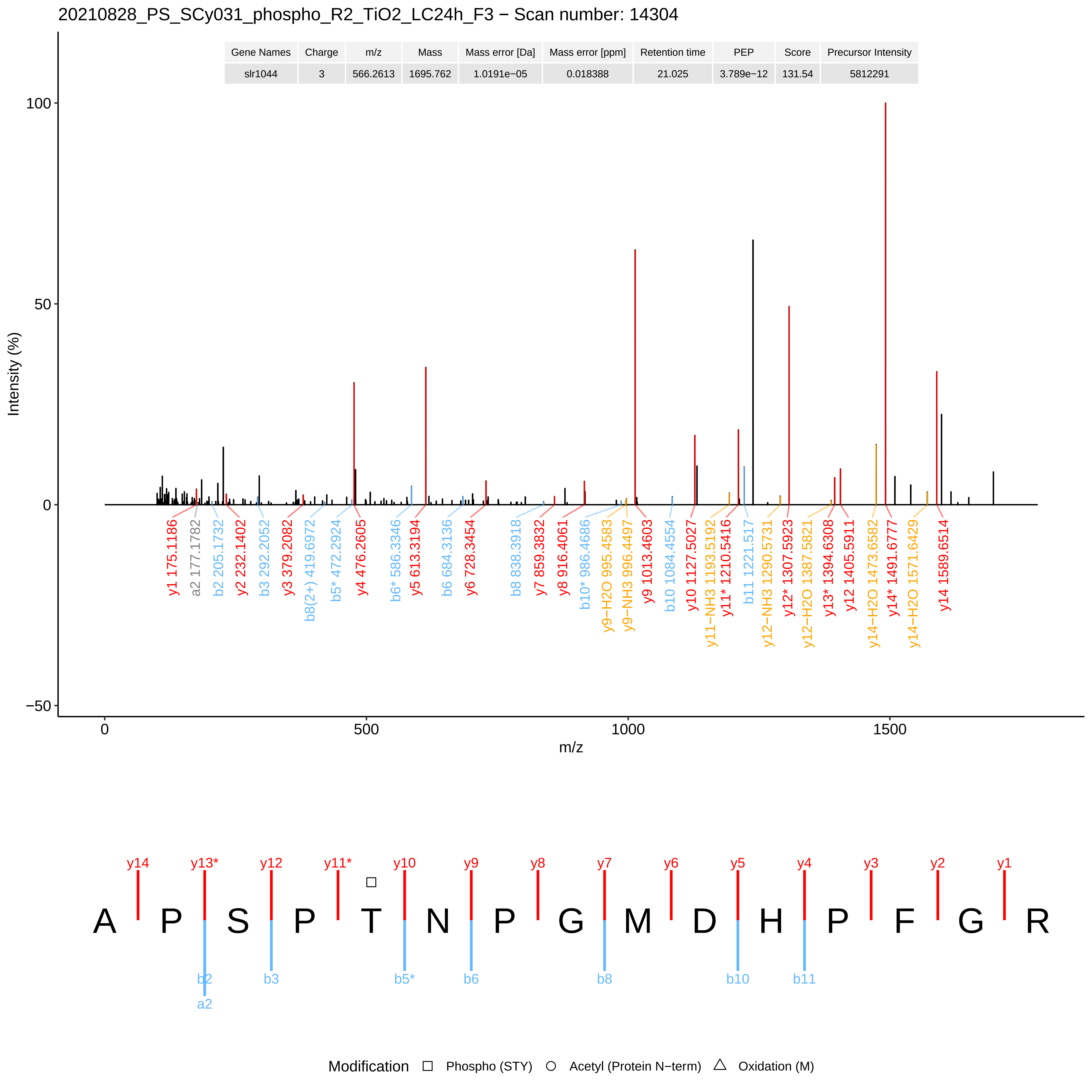
20210828_PS_SCy031_phospho_R2_TiO2_LC24h_F3 − Scan number: 14304
Gene Names | Charge | m/z | Mass | Mass error [Da] | Mass error [ppm] | Retention time | PEP | Score | Precursor Intensity
slr1044 | 3 | 566.2613 | 1695.762 | 1.0191e−05 | 0.018388 | 21.025 | 3.789e−12 | 131.54 | 5812291
Intensity (%)
m/z
y1 175.1186
a2 177.1782
b2 205.1732
y2 232.1402
b3 292.2052
y3 379.2082
b8(2+) 419.6972
b5* 472.2924
y4 476.2605
b6* 586.3346
y5 613.3194
b6 684.3136
y6 728.3454
b8 838.3918
y7 859.3832
y8 916.4061
b10* 986.4686
y9−H2O 995.4583
y9−NH3 996.4497
y9 1013.4603
b10 1084.4554
y10 1127.5027
y11−NH3 1193.5192
y11* 1210.5416
b11 1221.517
y12−NH3 1290.5731
y12* 1307.5923
y12−H2O 1387.5821
y13* 1394.6308
y12 1405.5911
y14−H2O 1473.6582
y14* 1491.6777
y14−H2O 1571.6429
y14 1589.6514
A P S P T N P G M D H P F G R
y14 y13* y12 y11* y10 y9 y8 y7 y6 y5 y4 y3 y2 y1
b2 a2 b3 b5* b6 b8 b10 b11
Modification □ Phospho (STY) ○ Acetyl (Protein N−term) △ Oxidation (M)

20210823_PS_SCy031_R1_phospho_LC24h_TiO2_3 − Scan number: 6220

Gene Names	Charge	m/z	Mass	Mass error [Da]	Mass error [ppm]	Retention time	PEP	Score	Precursor Intensity
slr1044	2	568.2426	1134.471	−0.00013184	−0.23788	10.847	4.3349e−48	210.61	9888116

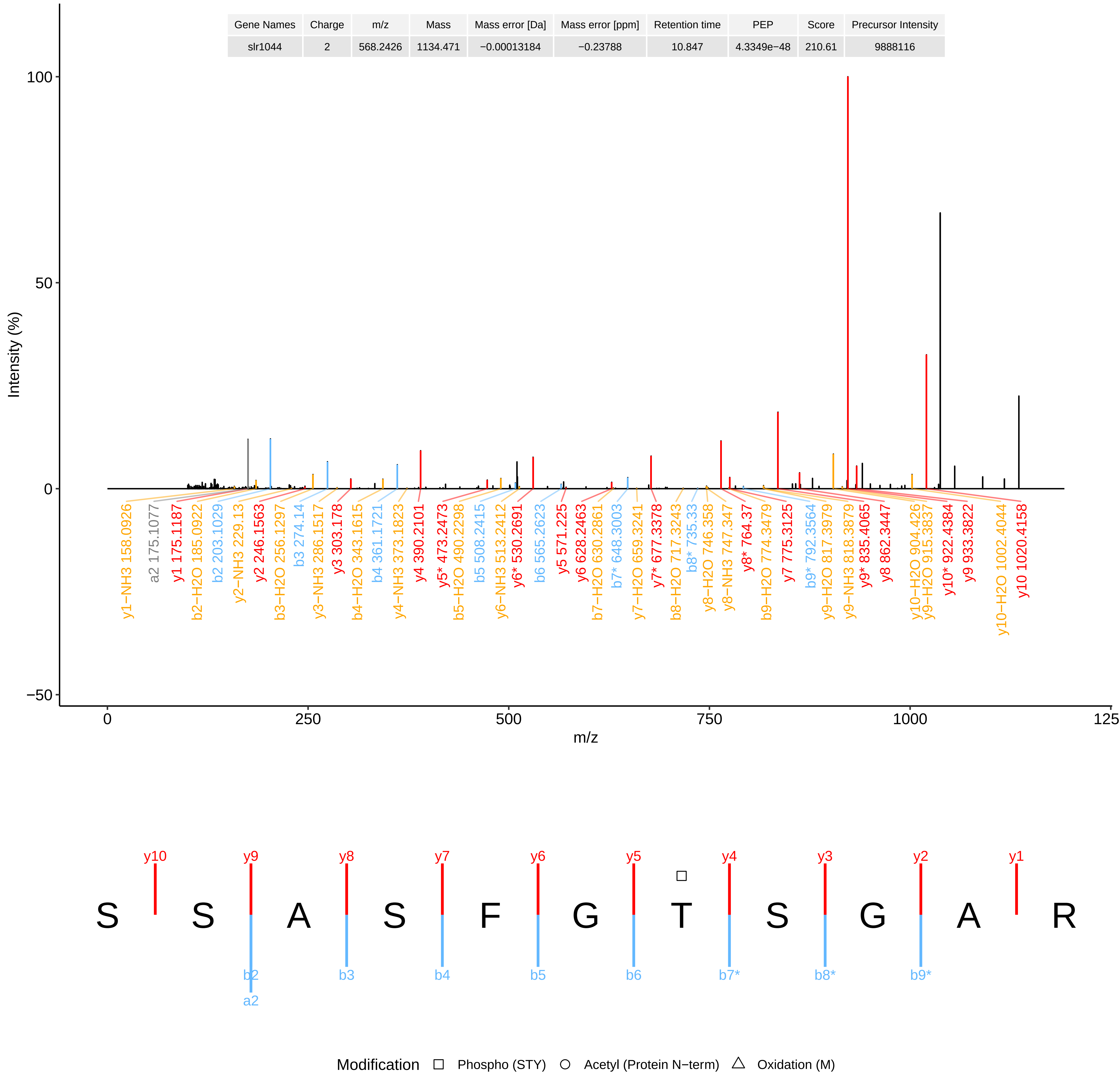

20210828_PS_SCy031_phospho_R3_TiO2_LC24h_F2 − Scan number: 27047

Gene Names	Charge	m/z	Mass	Mass error [Da]	Mass error [ppm]	Retention time	PEP	Score	Precursor Intensity
slr1102	2	1075.553	2149.092	−0.0005204	−0.48362	37.038	1.369e−165	308.1	4577405

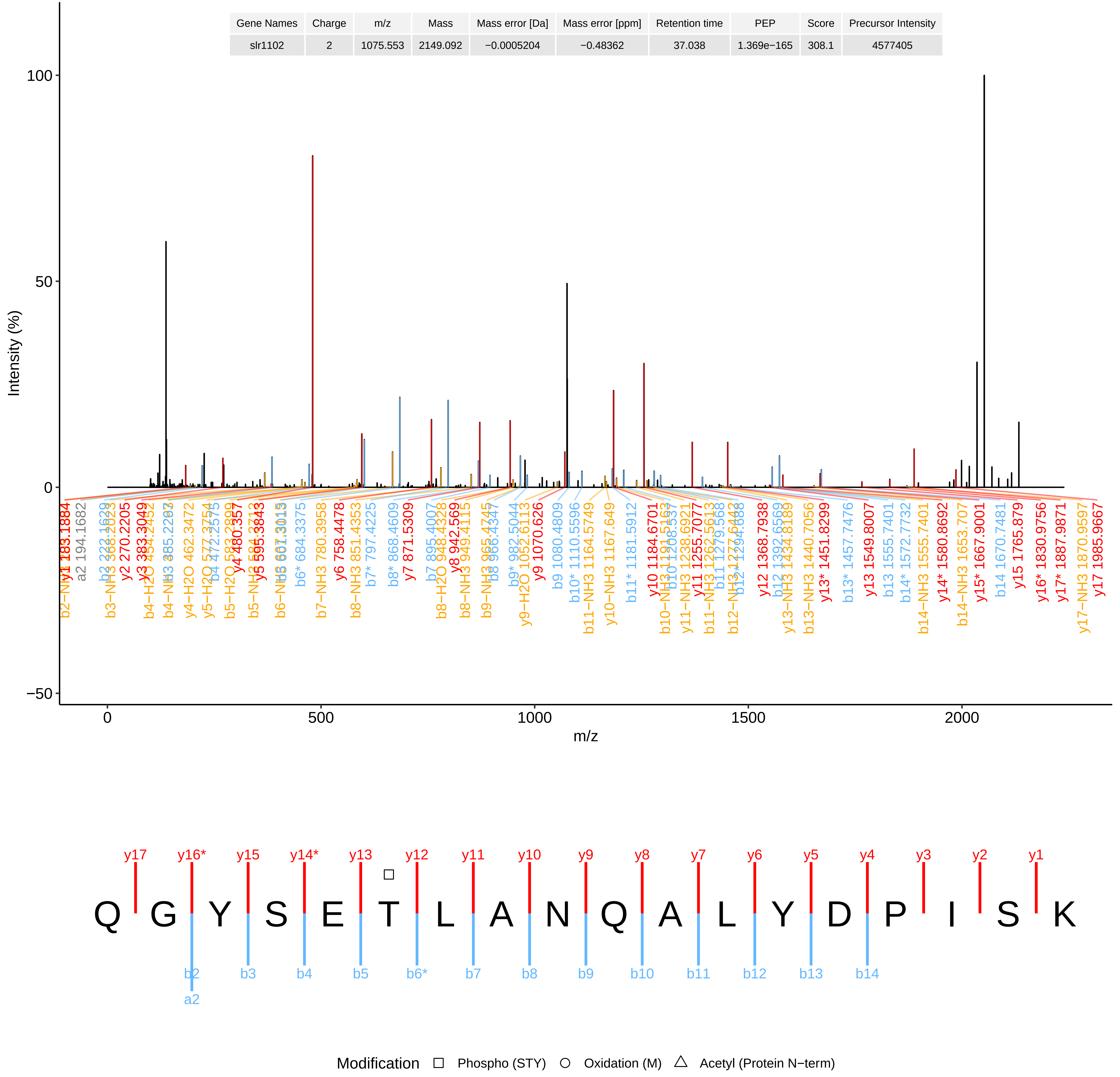

20211212_PS_SCy031_proteome_R1_HC_F9 − Scan number: 17190

Gene Names	Charge	m/z	Mass	Mass error [Da]	Mass error [ppm]	Retention time	PEP	Score	Precursor Intensity
slr1130	2	701.8715	1401.728	14.016	20800	29.362	0.0023279	45.997	3509333

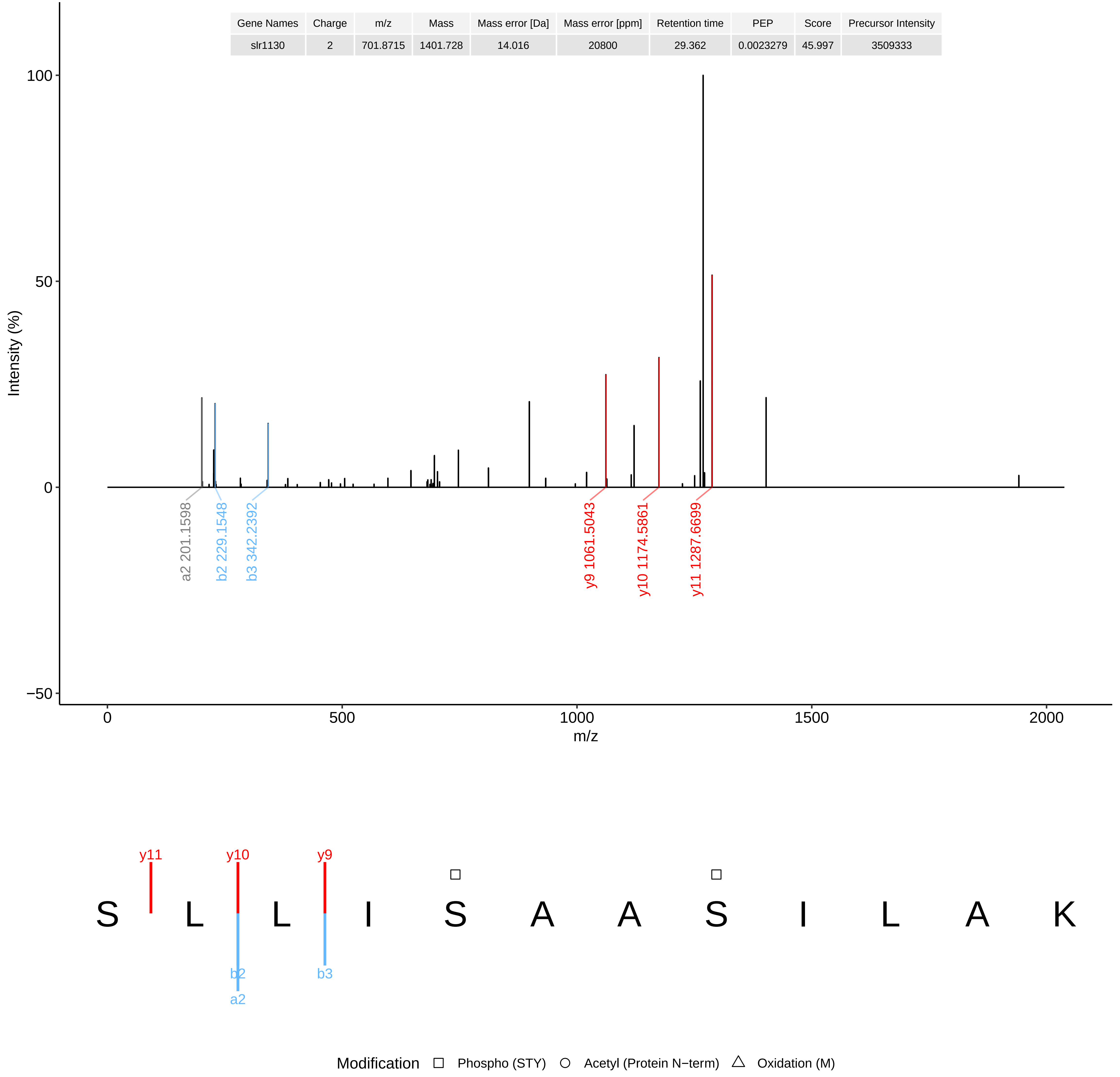

20210828_PS_SCy031_phospho_R3_TiO2_LC3h_F3 − Scan number: 15166

Gene Names	Charge	m/z	Mass	Mass error [Da]	Mass error [ppm]	Retention time	PEP	Score	Precursor Intensity
slr1137	2	765.359	1528.704	0.00099233	1.2966	22.14	1.1205e−63	238.17	5490594

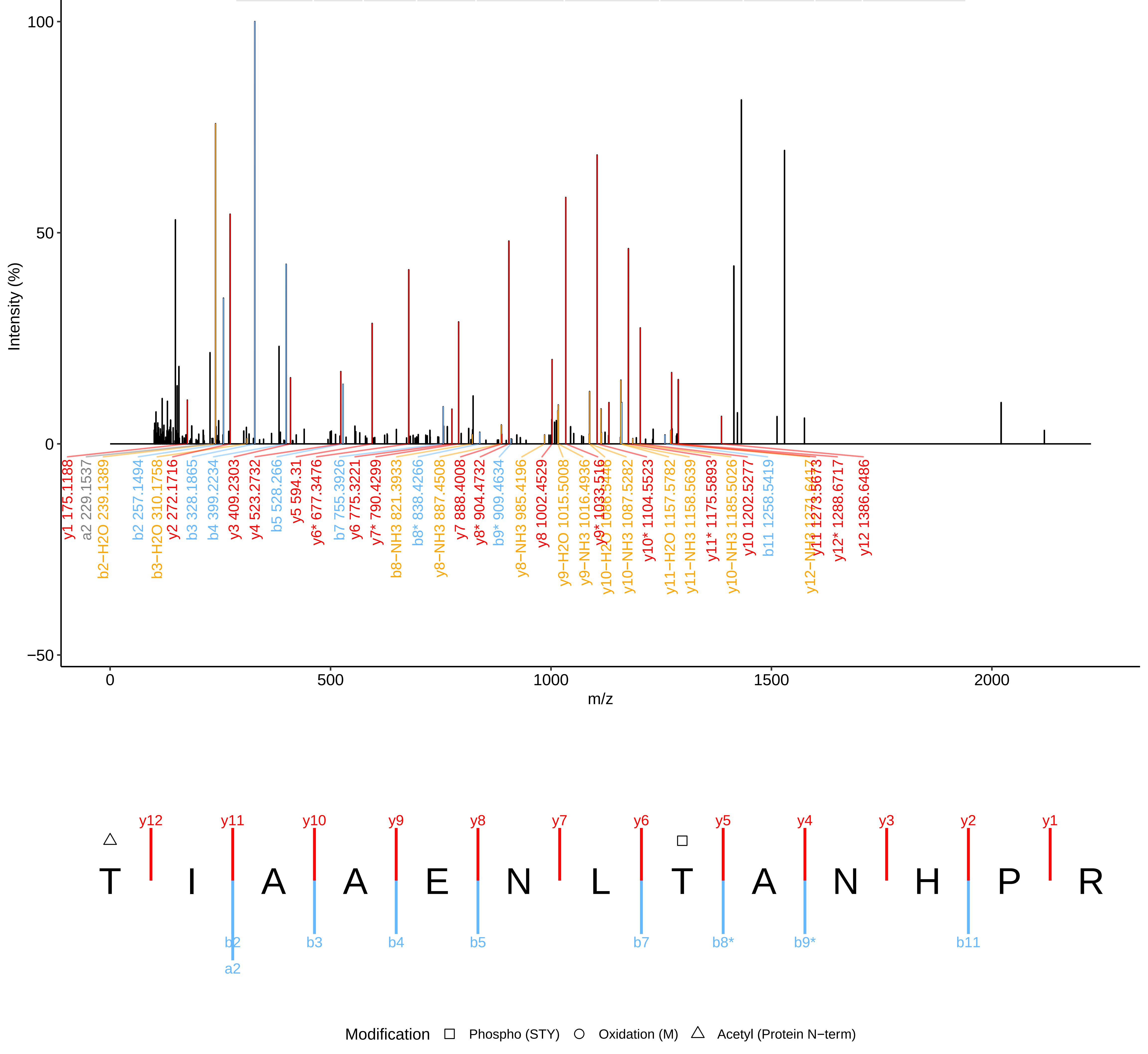

20210823_PS_SCy031_R1_phospho_HC_TiO2_1 − Scan number: 3904

Gene Names	Charge	m/z	Mass	Mass error [Da]	Mass error [ppm]	Retention time	PEP	Score	Precursor Intensity
slr1163	2	871.3843	1740.754	NA	NA	7.745	0.00063436	67.563	5204974

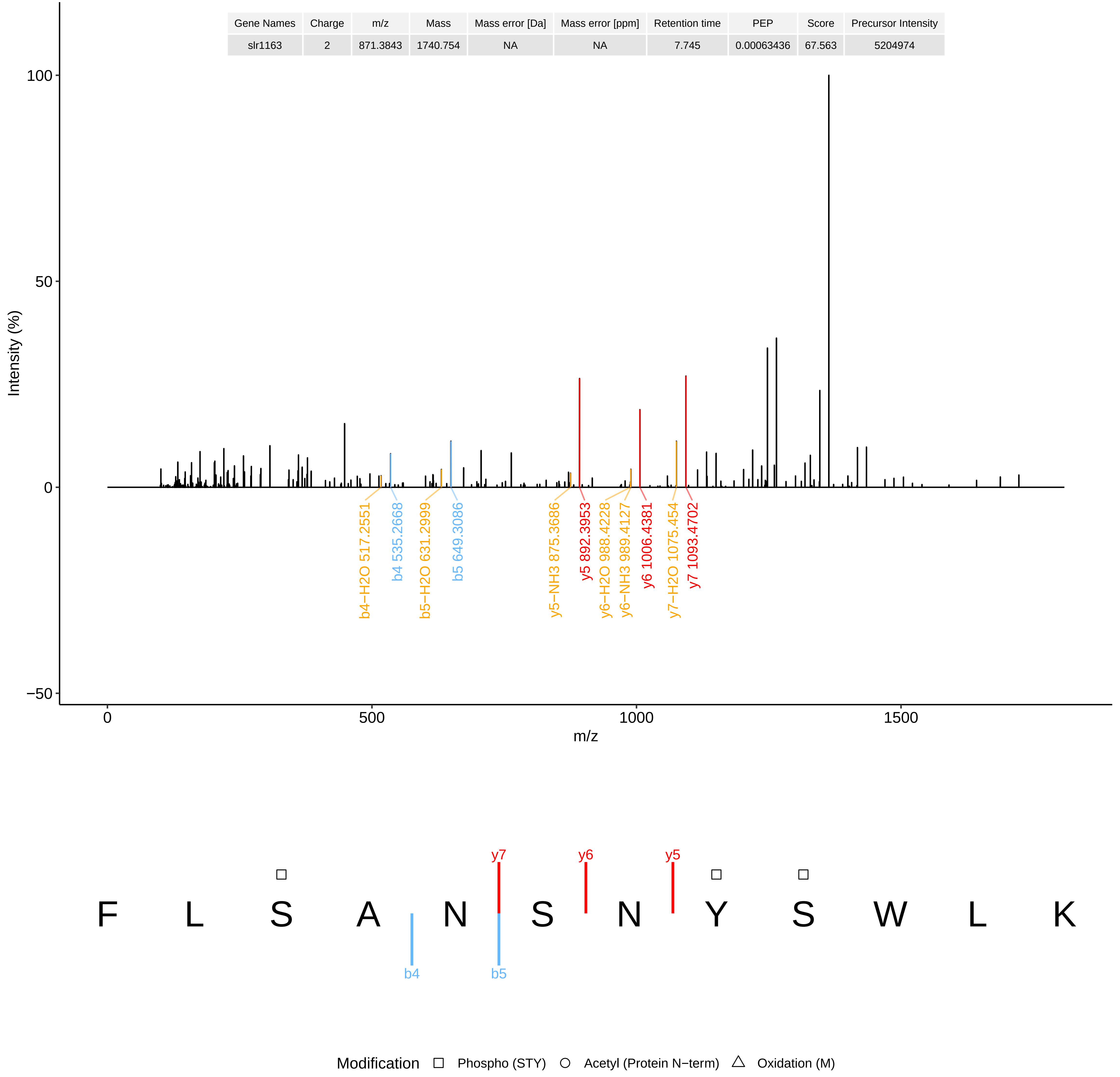

20210828_PS_SCy031_phospho_R3_TiO2_HC_F3 − Scan number: 21609

Gene Names	Charge	m/z	Mass	Mass error [Da]	Mass error [ppm]	Retention time	PEP	Score	Precursor Intensity
slr1225	3	789.7199	2366.138	−0.0010614	−1.3434	30.296	6.5743e−17	162.77	NA

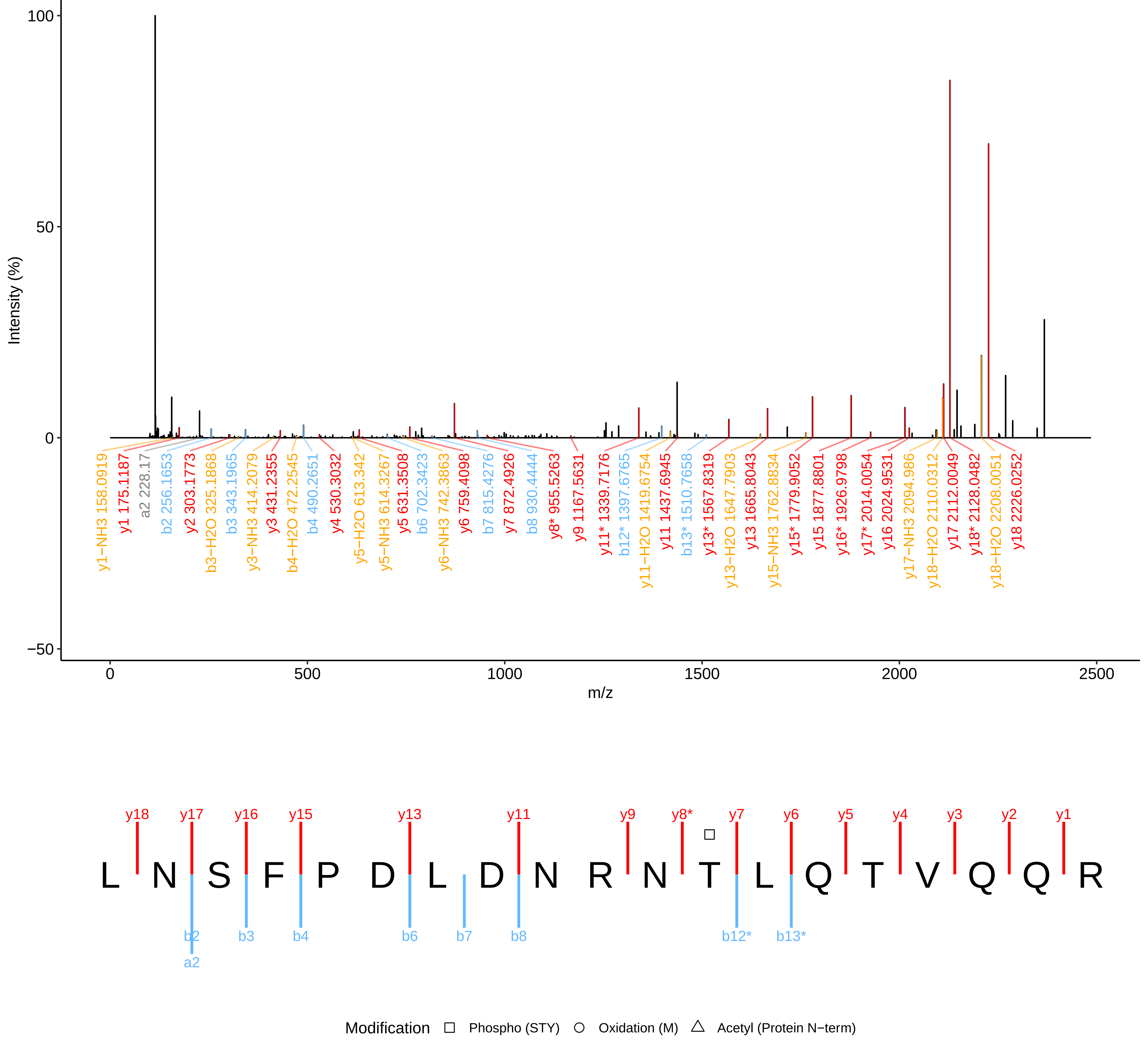

20210828_PS_SCy031_phospho_R3_TiO2_LC24h_F2 − Scan number: 6594

Gene Names	Charge	m/z	Mass	Mass error [Da]	Mass error [ppm]	Retention time	PEP	Score	Precursor Intensity
slr1225	2	598.2952	1194.576	−0.00020101	−0.34403	11.311	5.9976e−08	134.75	2261611

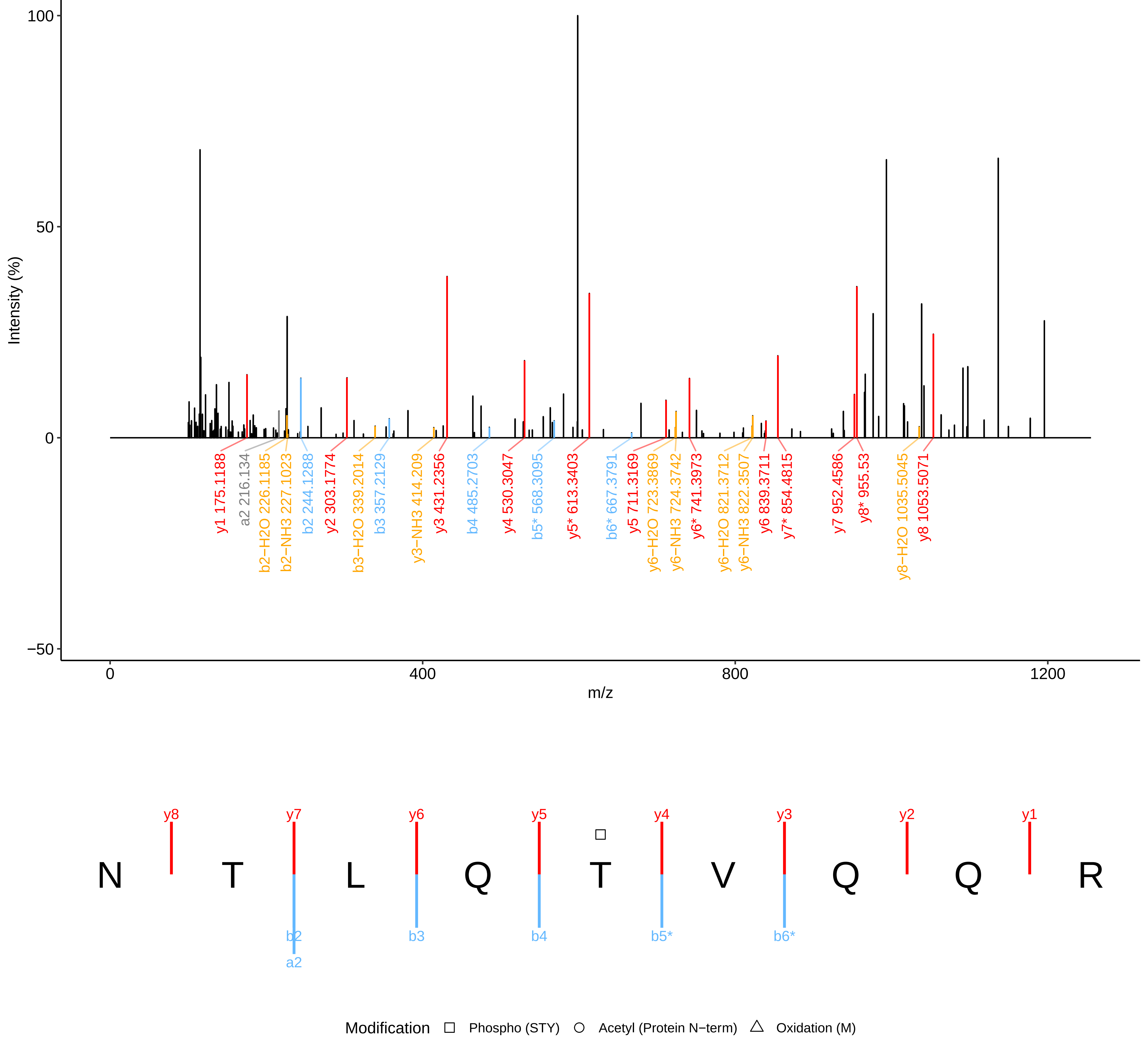

20210828_PS_SCy031_phospho_R3_TiO2_LC24h_F1 − Scan number: 20463

Gene Names	Charge	m/z	Mass	Mass error [Da]	Mass error [ppm]	Retention time	PEP	Score	Precursor Intensity
slr1225	3	1070.541	3208.602	0.0020562	1.9548	28.519	2.3475e−118	197.26	5027588

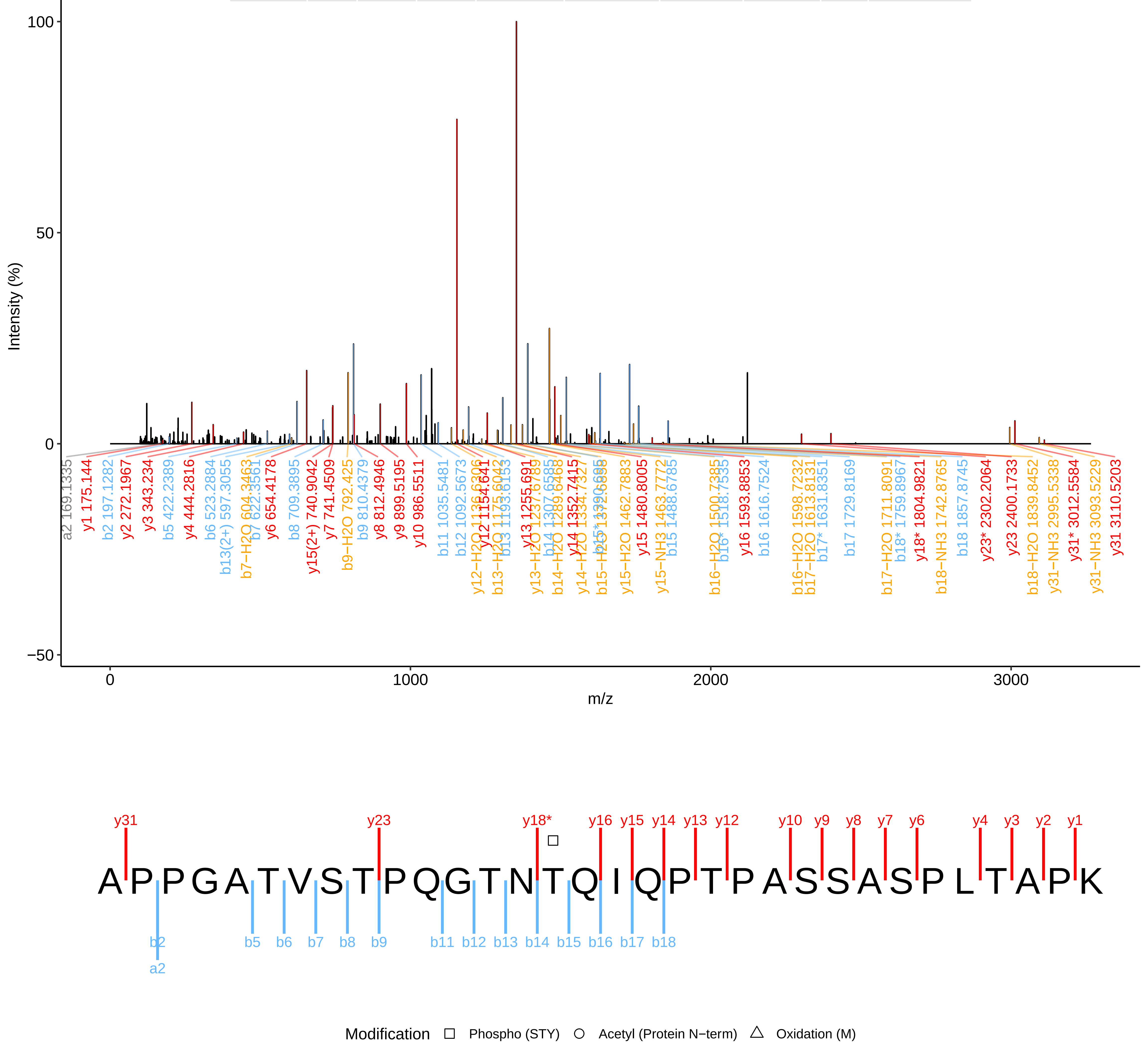

20210828_PS_SCy031_phospho_R3_TiO2_LC3h_F2 − Scan number: 9608

Gene Names	Charge	m/z	Mass	Mass error [Da]	Mass error [ppm]	Retention time	PEP	Score	Precursor Intensity
slr1311;sll1867	2	485.2237	968.4328	NA	NA	15.052	0.0037835	116.9	NA

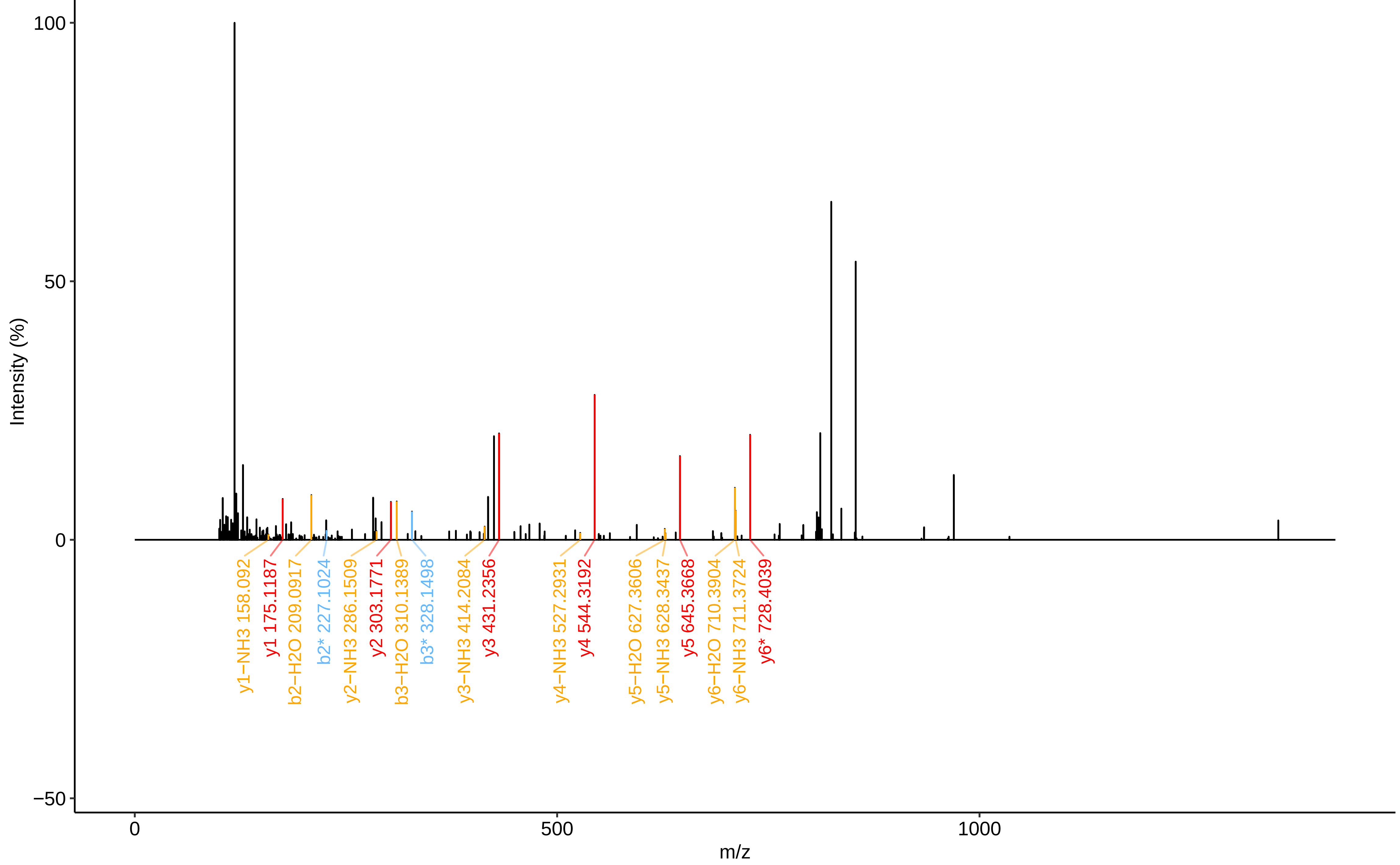

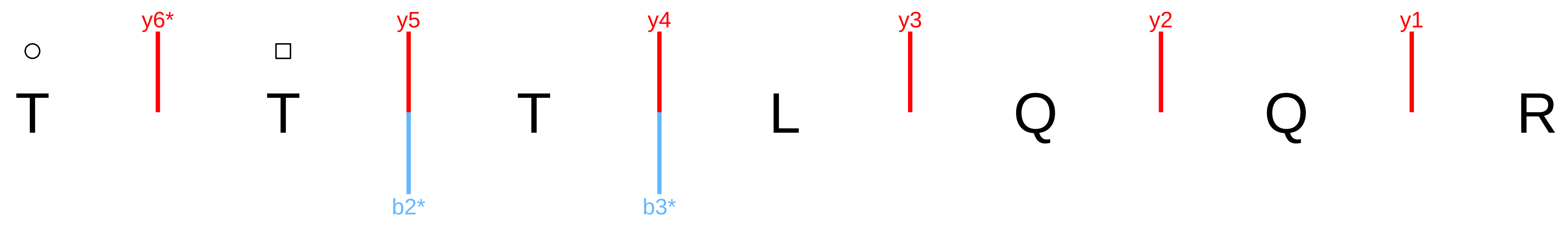

Modification □ Phospho (STY) ○ Acetyl (Protein N−term) △ Oxidation (M)

20210828_PS_SCy031_phospho_R2_TiO2_HC_F3 − Scan number: 18231

Gene Names	Charge	m/z	Mass	Mass error [Da]	Mass error [ppm]	Retention time	PEP	Score	Precursor Intensity
slr1390	2	1613.756	3225.497	NA	NA	23.431	0.0014843	66.436	NA

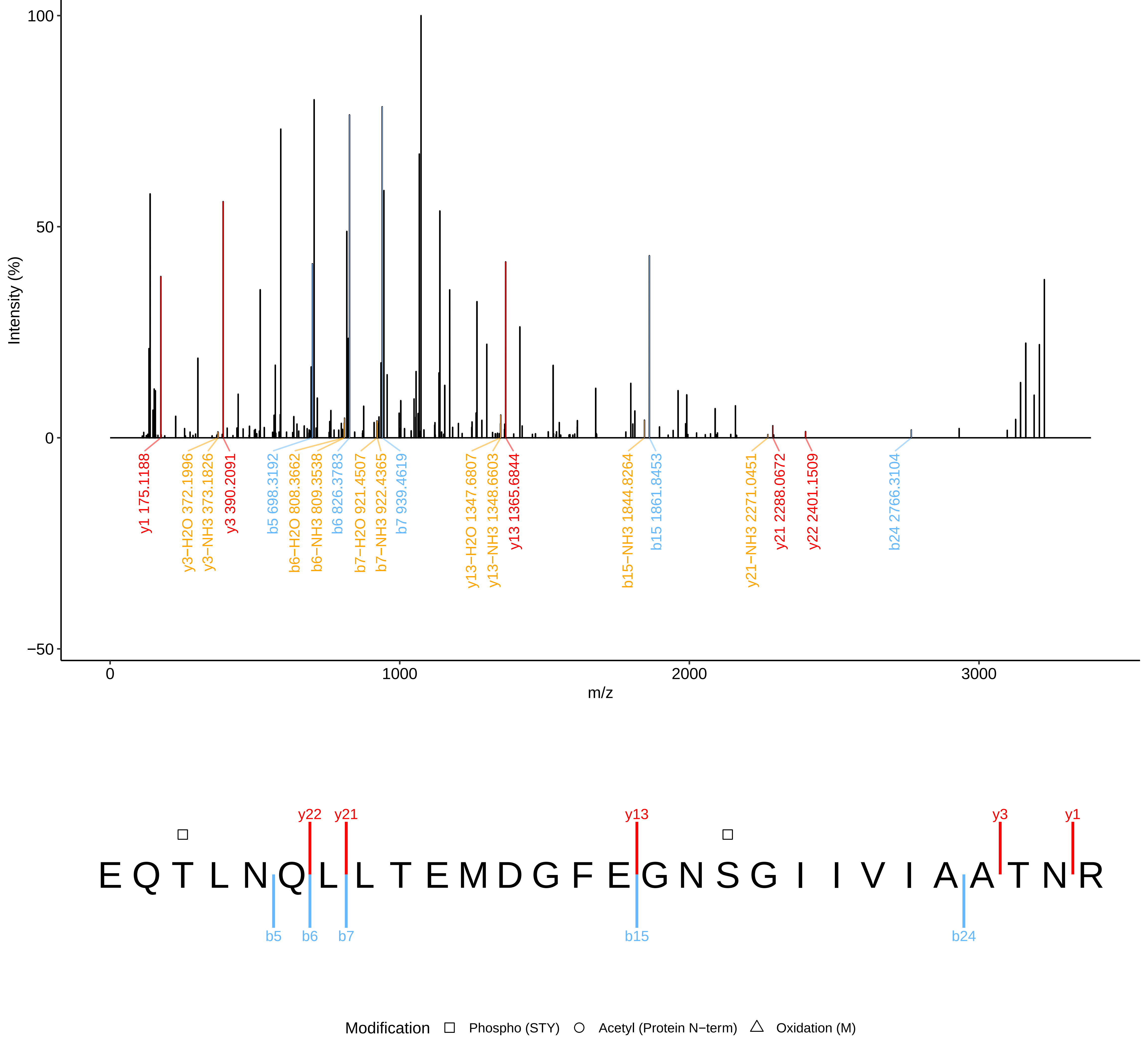

20211212_PS_SCy031_proteome_R1_3hLC_F9 − Scan number: 9256

Gene Names	Charge	m/z	Mass	Mass error [Da]	Mass error [ppm]	Retention time	PEP	Score	Precursor Intensity
slr1476	4	554.542	2214.139	−0.00041541	−0.76009	16.822	0.00022406	56.851	1264415

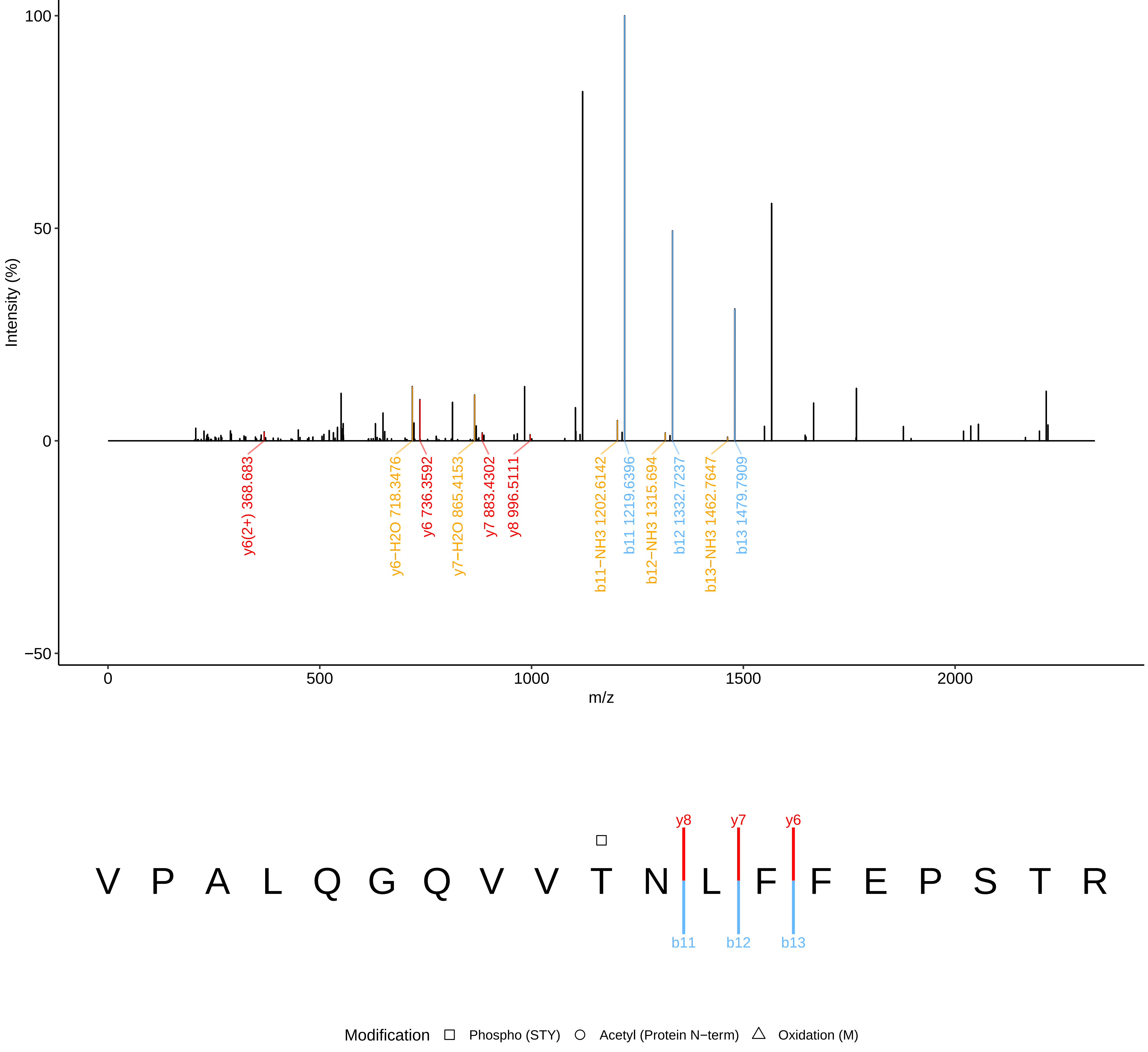

20210828_PS_SCy031_phospho_R3_TiO2_HC_F3 − Scan number: 23233

Gene Names	Charge	m/z	Mass	Mass error [Da]	Mass error [ppm]	Retention time	PEP	Score	Precursor Intensity
slr1505	2	629.8378	1257.661	−0.00074326	−1.1801	32.347	0.036555	88.056	2687040

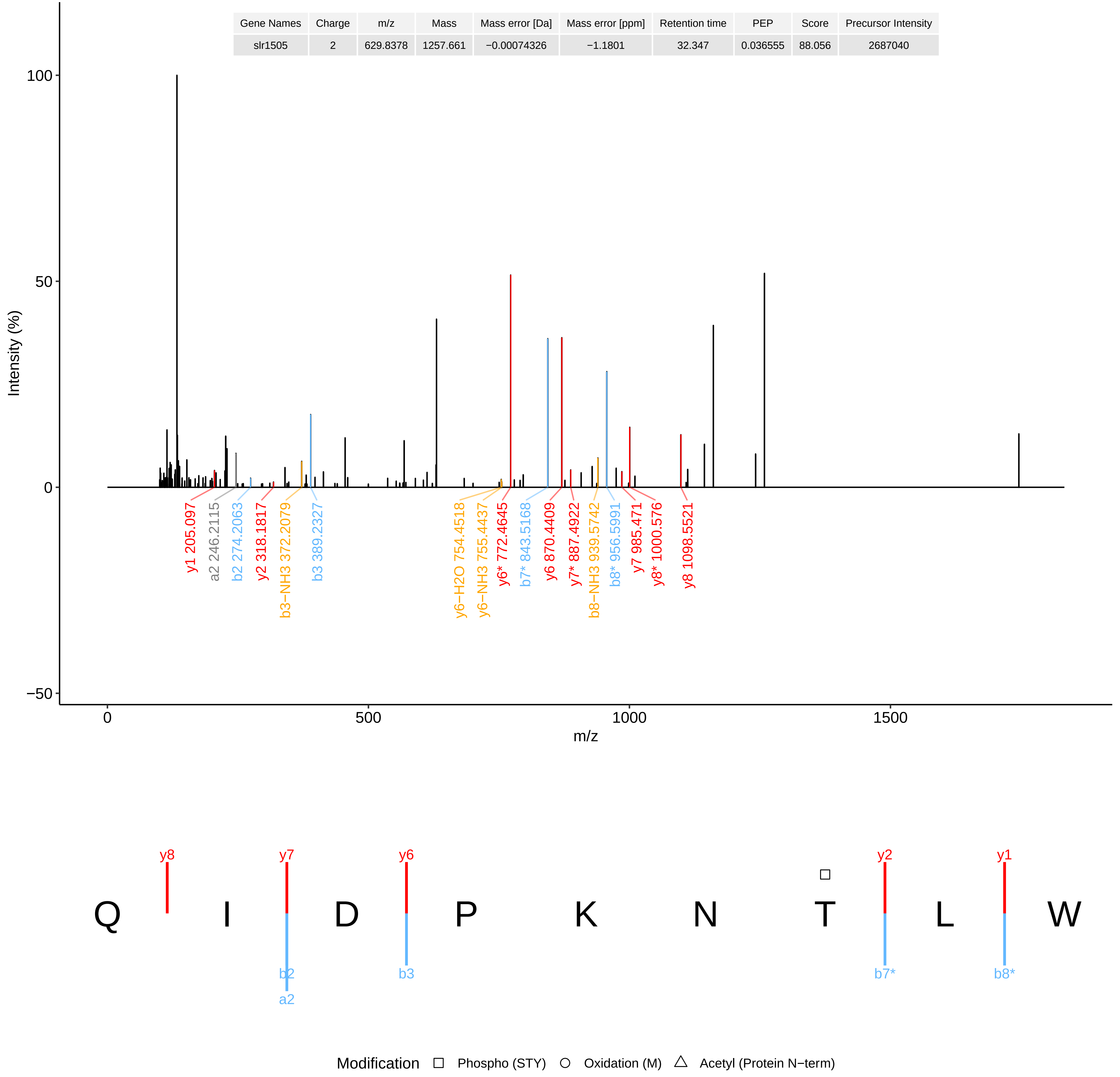

20210828_PS_SCy031_phospho_R3_TiO2_LC24h_F2 − Scan number: 14341

Gene Names	Charge	m/z	Mass	Mass error [Da]	Mass error [ppm]	Retention time	PEP	Score	Precursor Intensity
slr1513	2	848.8931	1695.772	0.0014546	1.7136	21.035	0.0032747	123.14	4818986

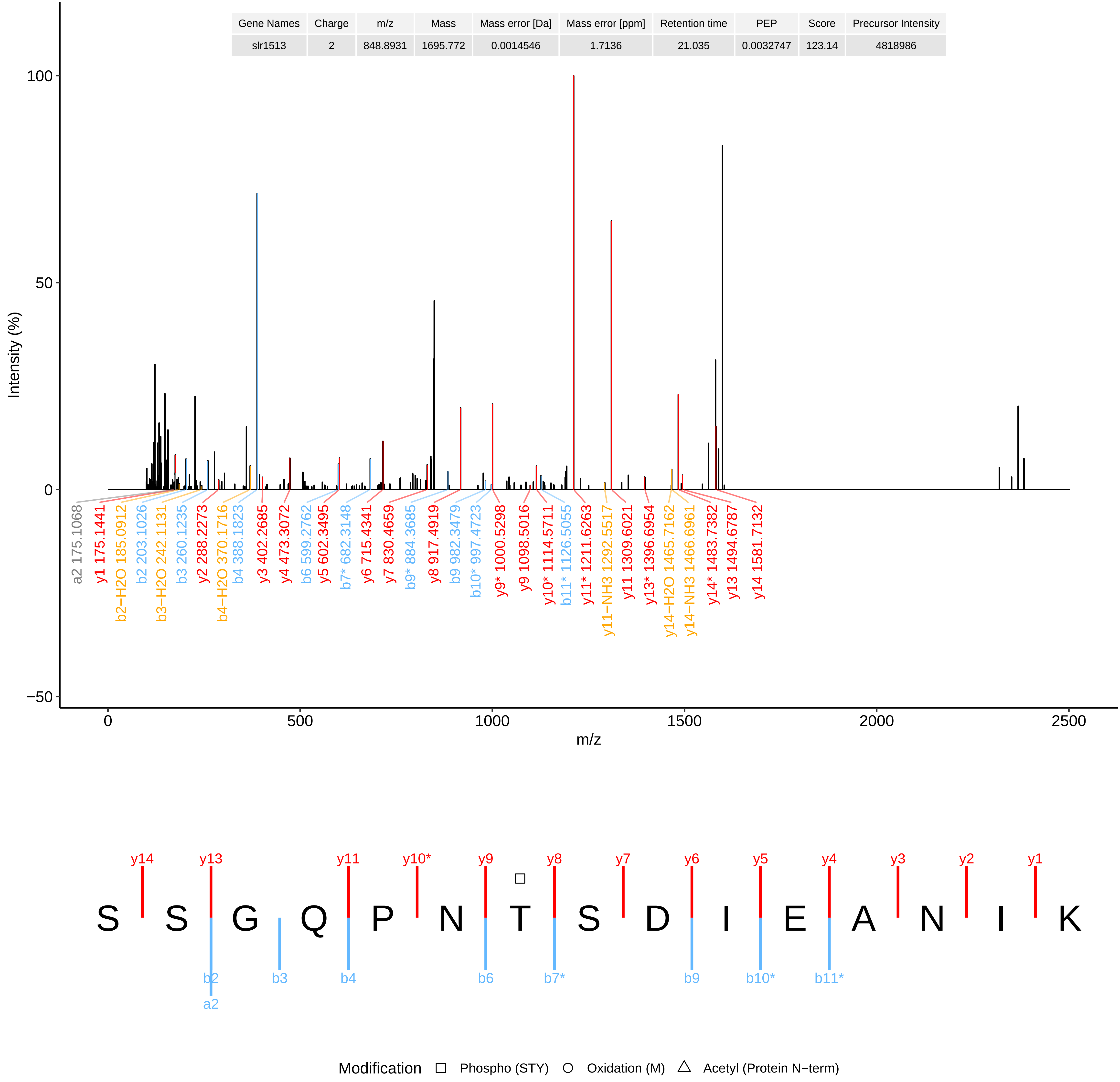

20210828_PS_SCy031_phospho_R3_TiO2_LC24h_F2 − Scan number: 16044

Gene Names	Charge	m/z	Mass	Mass error [Da]	Mass error [ppm]	Retention time	PEP	Score	Precursor Intensity
slr1513	2	852.9182	1703.822	NA	NA	23.176	2.0855e−12	157.82	NA

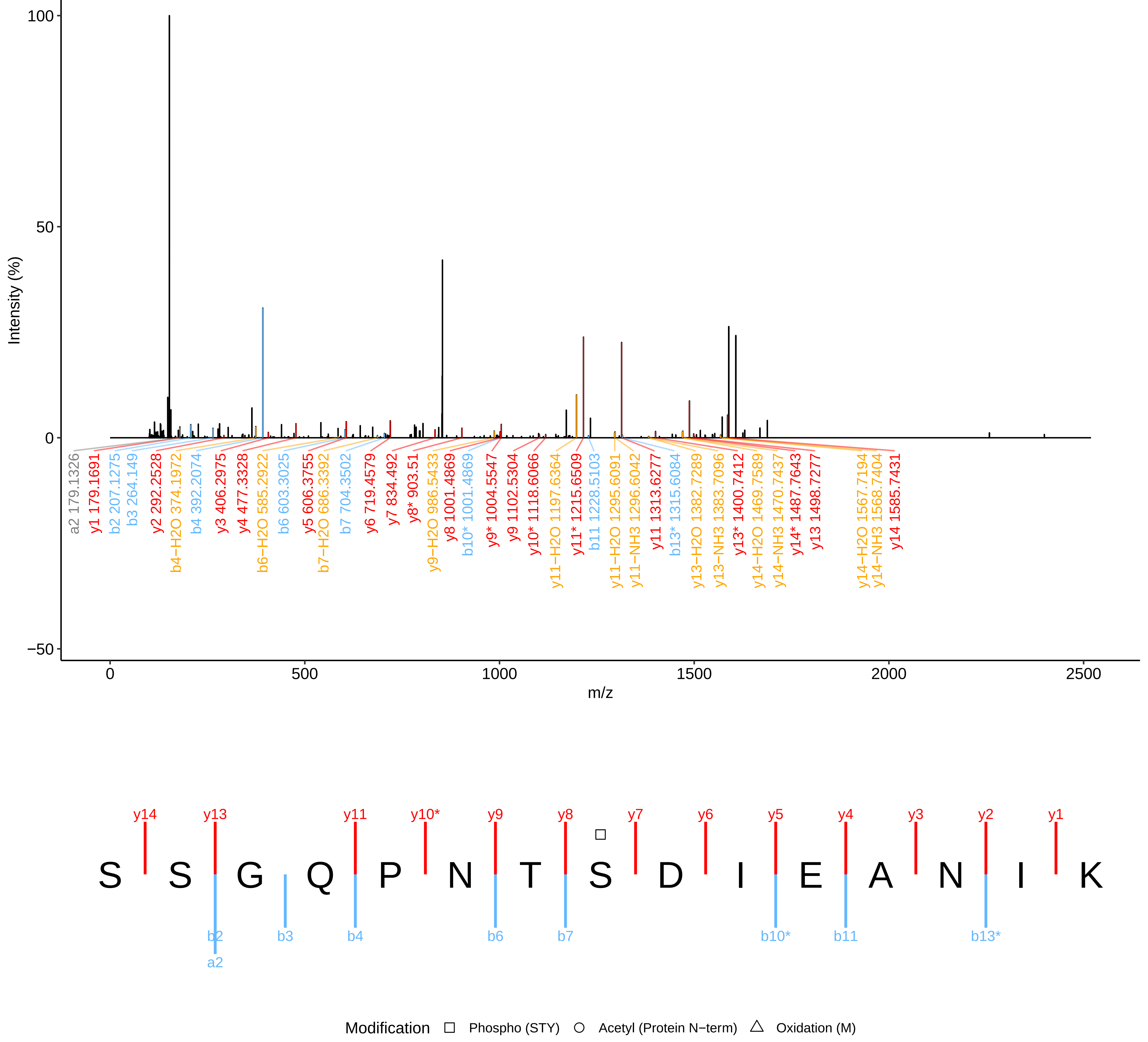

20210828_PS_SCy031_phospho_R2_TiO2_LC3h_F3 − Scan number: 5929

Gene Names	Charge	m/z	Mass	Mass error [Da]	Mass error [ppm]	Retention time	PEP	Score	Precursor Intensity
slr1516	3	419.8754	1256.604	−7.5054e−05	−0.18834	10.621	1.6273e−07	107.85	4676250

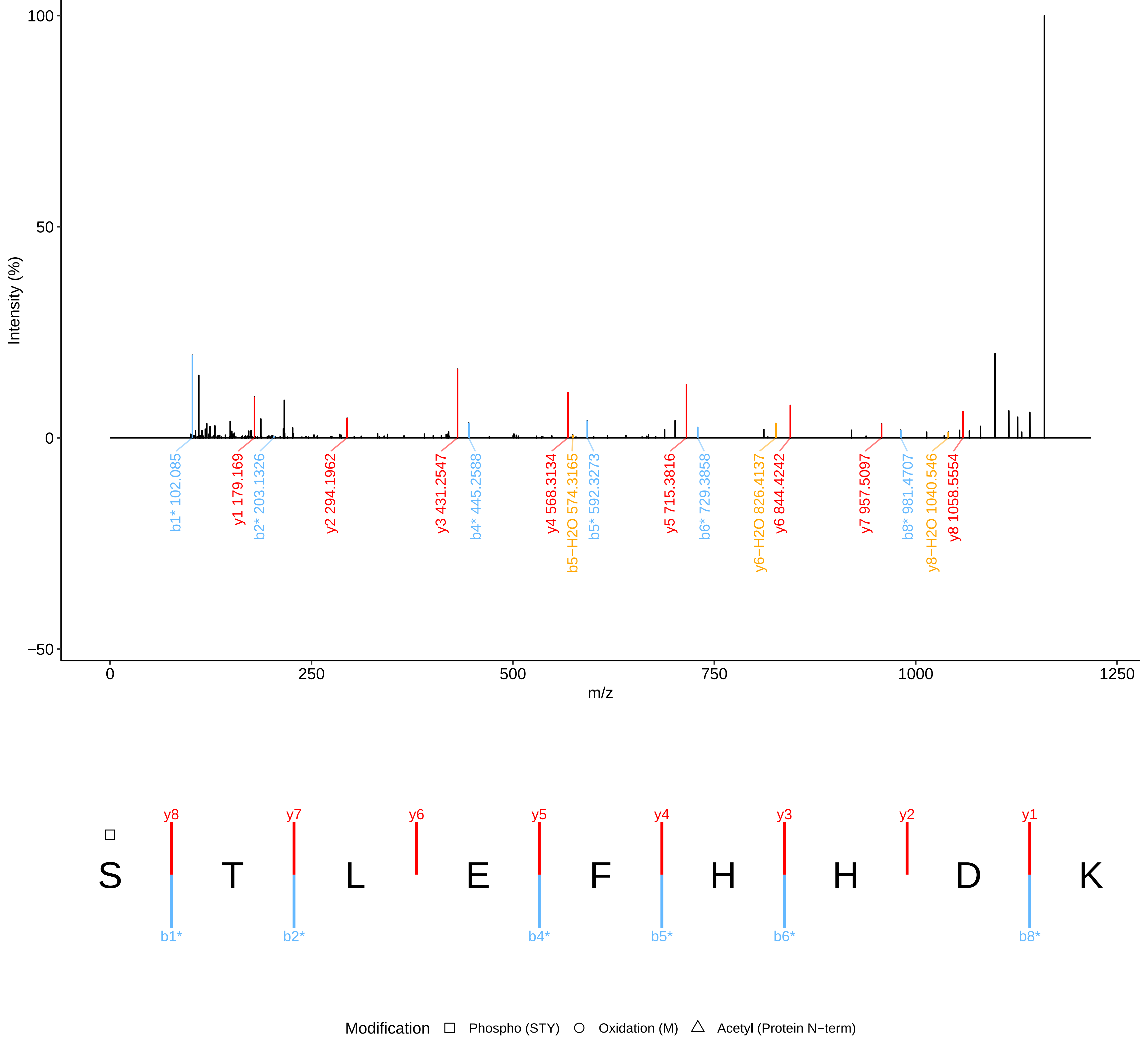

20210828_PS_SCy031_phospho_R2_TiO2_LC3h_F3 − Scan number: 3477

Gene Names	Charge	m/z	Mass	Mass error [Da]	Mass error [ppm]	Retention time	PEP	Score	Precursor Intensity
slr1536	2	705.3247	1408.635	NA	NA	7.5578	0.0028503	63.419	NA

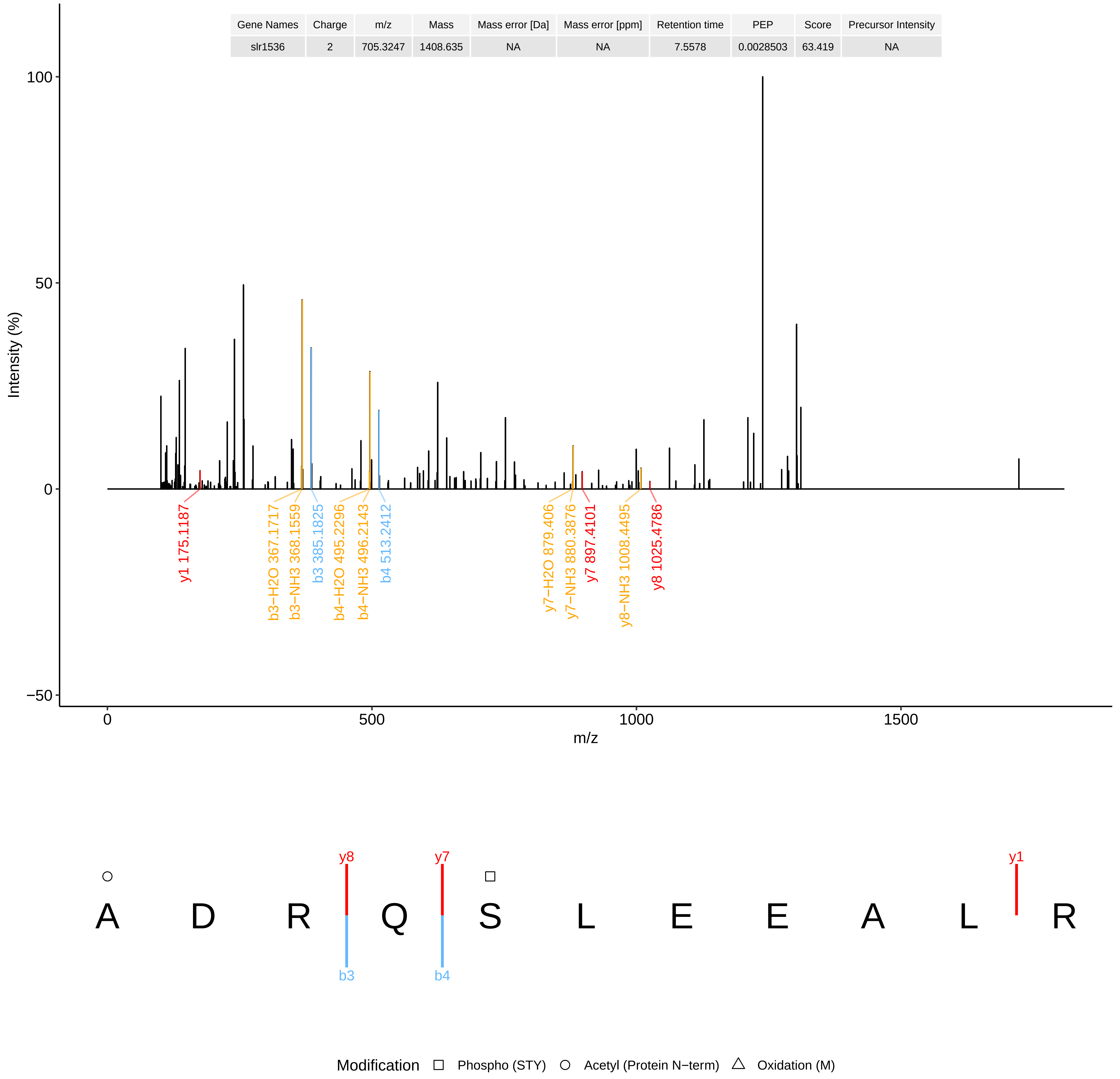

20210828_PS_SCy031_phospho_R3_TiO2_LC24h_F2 − Scan number: 32045

Gene Names	Charge	m/z	Mass	Mass error [Da]	Mass error [ppm]	Retention time	PEP	Score	Precursor Intensity
slr1655	3	1107.19	3318.55	NA	NA	43.353	4.4556e−68	199.58	2426371

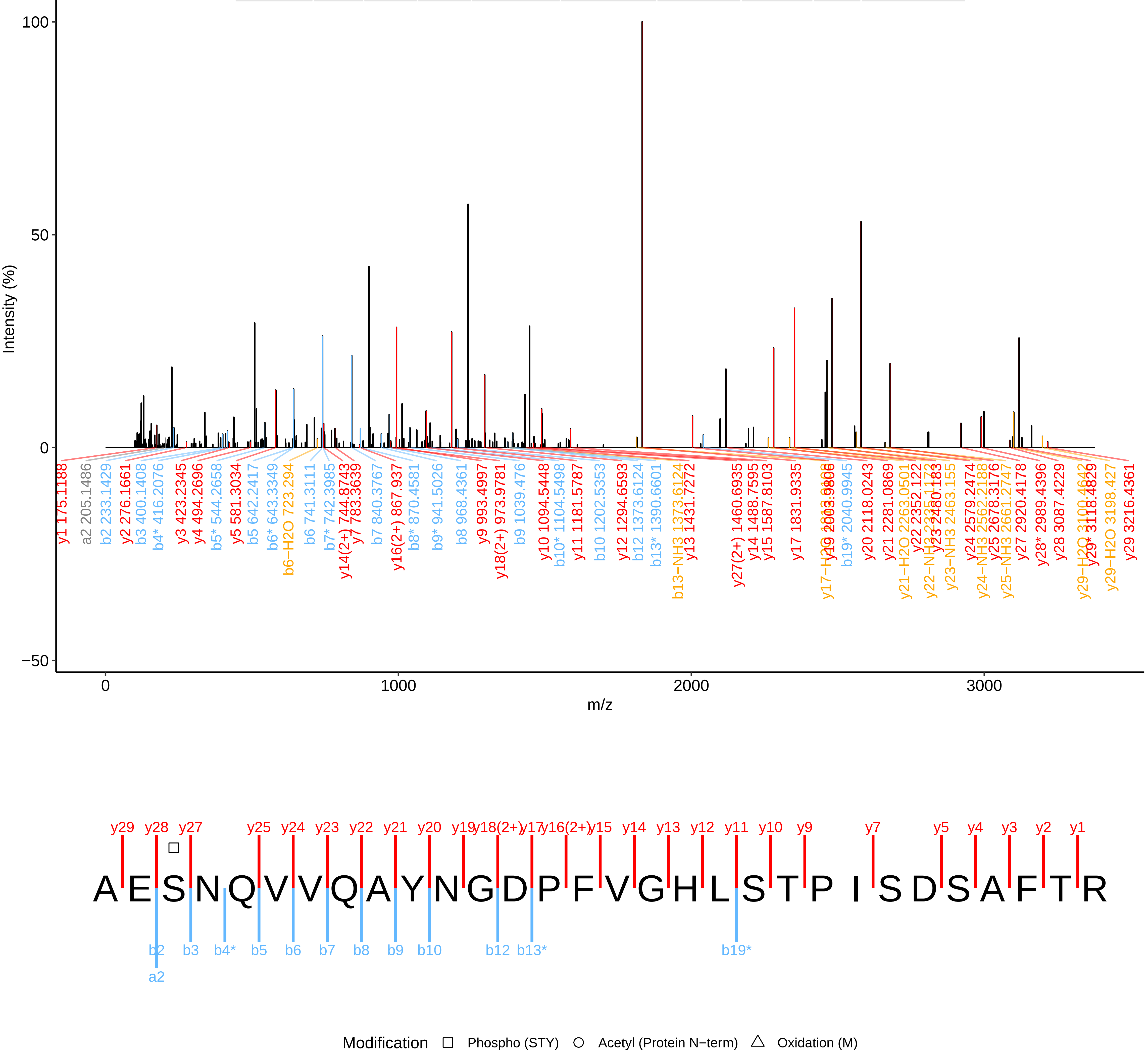

20210828_PS_SCy031_phospho_R3_TiO2_HC_F3 − Scan number: 10371

Gene Names	Charge	m/z	Mass	Mass error [Da]	Mass error [ppm]	Retention time	PEP	Score	Precursor Intensity
slr1686	2	734.3884	1466.762	NA	NA	16.128	0.0010157	124.51	42746496

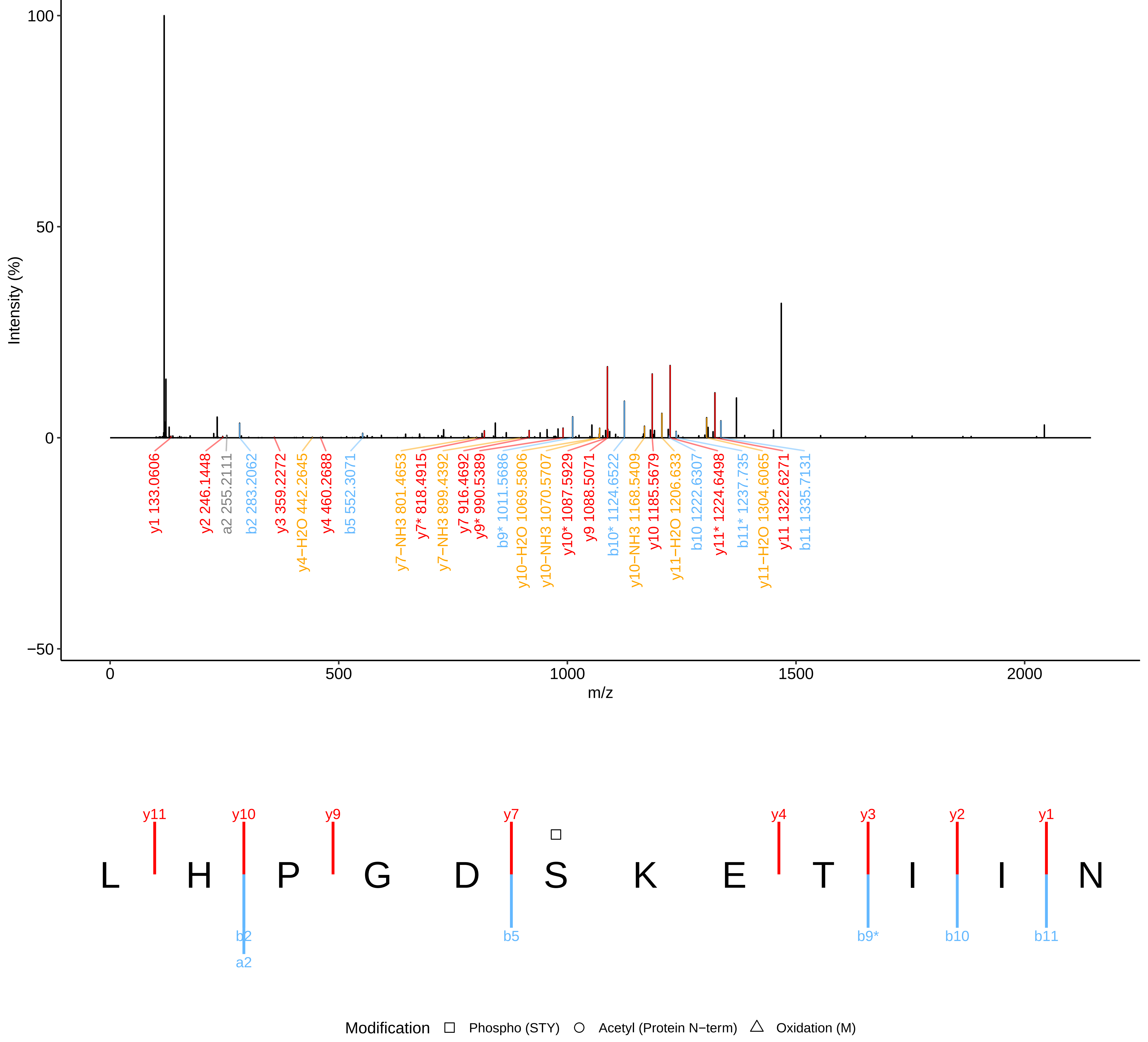

20210828_PS_SCy031_phospho_R2_TiO2_HC_F2 − Scan number: 10123

Gene Names	Charge	m/z	Mass	Mass error [Da]	Mass error [ppm]	Retention time	PEP	Score	Precursor Intensity
slr1686	2	730.3633	1458.712	0.00030648	0.41962	15.685	0.0085841	131.06	39885184

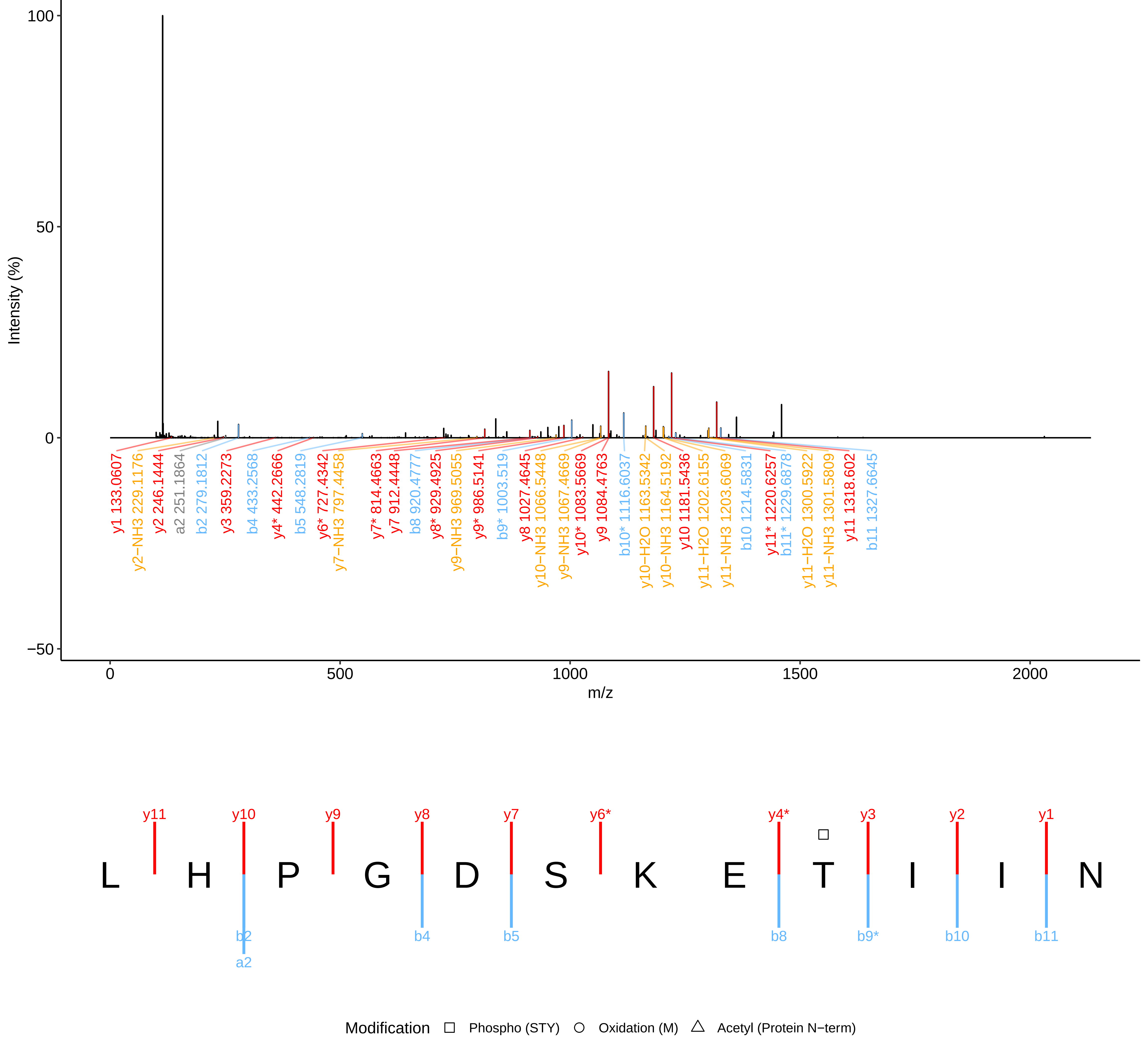

20210828_PS_SCy031_phospho_R3_TiO2_HC_F1 − Scan number: 30480

Gene Names	Charge	m/z	Mass	Mass error [Da]	Mass error [ppm]	Retention time	PEP	Score	Precursor Intensity
slr1686	3	1025.161	3072.463	NA	NA	41.368	0.00081133	72.214	NA

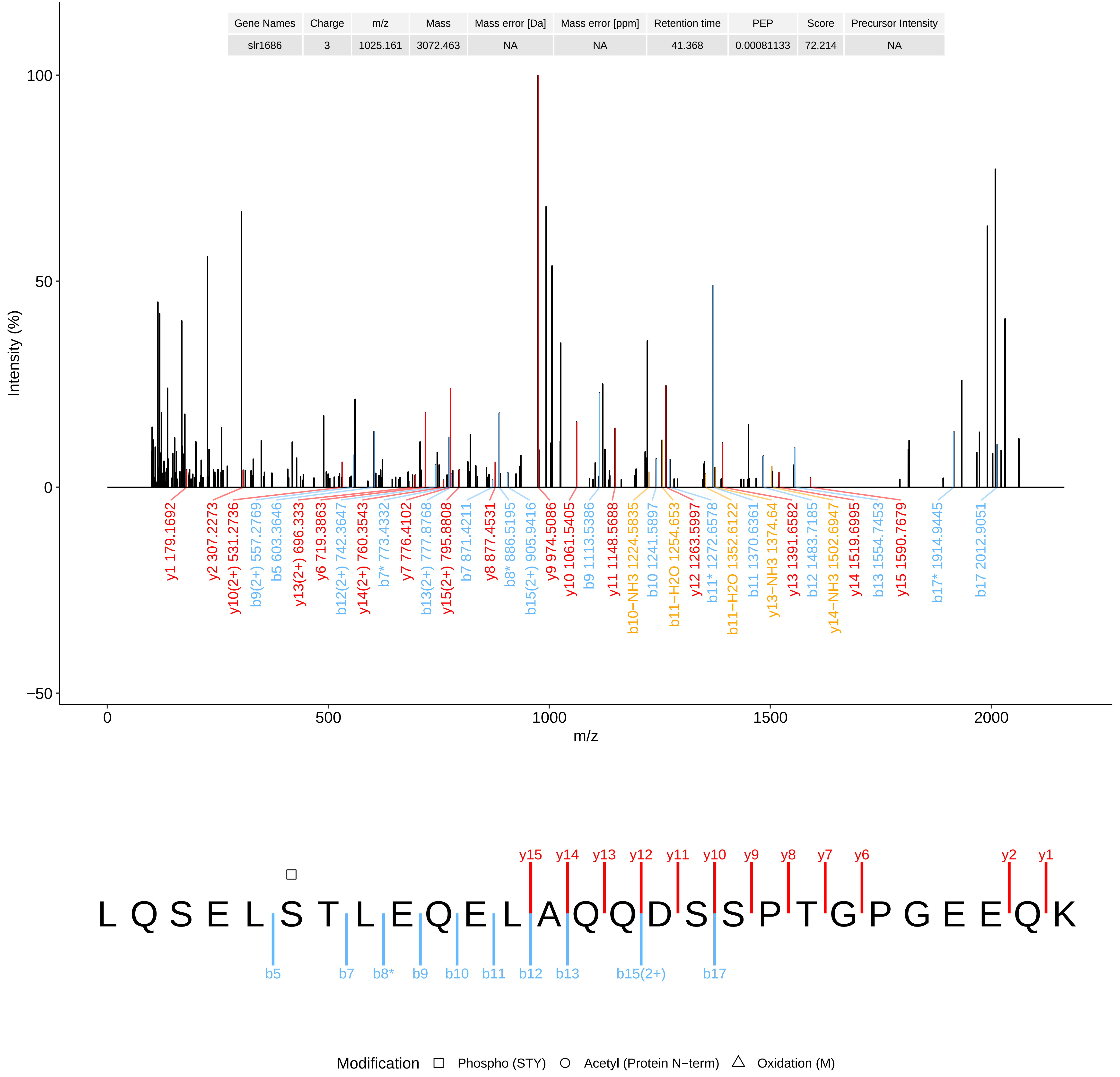

20210828_PS_SCy031_phospho_R3_TiO2_HC_F2 − Scan number: 14037

Gene Names	Charge	m/z	Mass	Mass error [Da]	Mass error [ppm]	Retention time	PEP	Score	Precursor Intensity
slr1697	3	884.3779	2650.112	0.0024397	2.7576	20.621	3.2041e−06	83.022	5074377

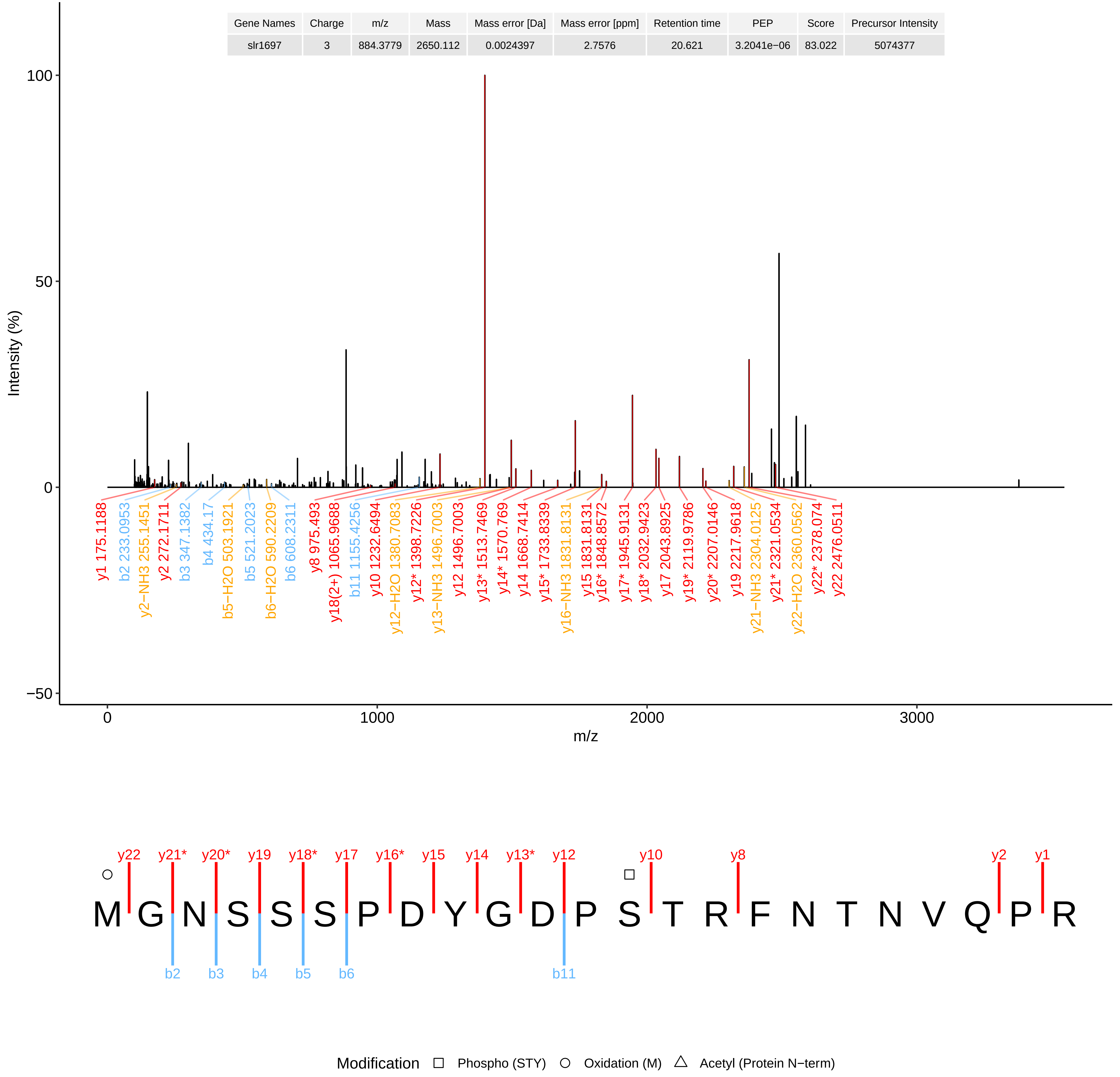

20210828_PS_SCy031_phospho_R3_TiO2_HC_F1 − Scan number: 3786

Gene Names	Charge	m/z	Mass	Mass error [Da]	Mass error [ppm]	Retention time	PEP	Score	Precursor Intensity
slr1710	2	742.8407	1483.667	NA	NA	7.6483	3.956e−08	147.7	NA

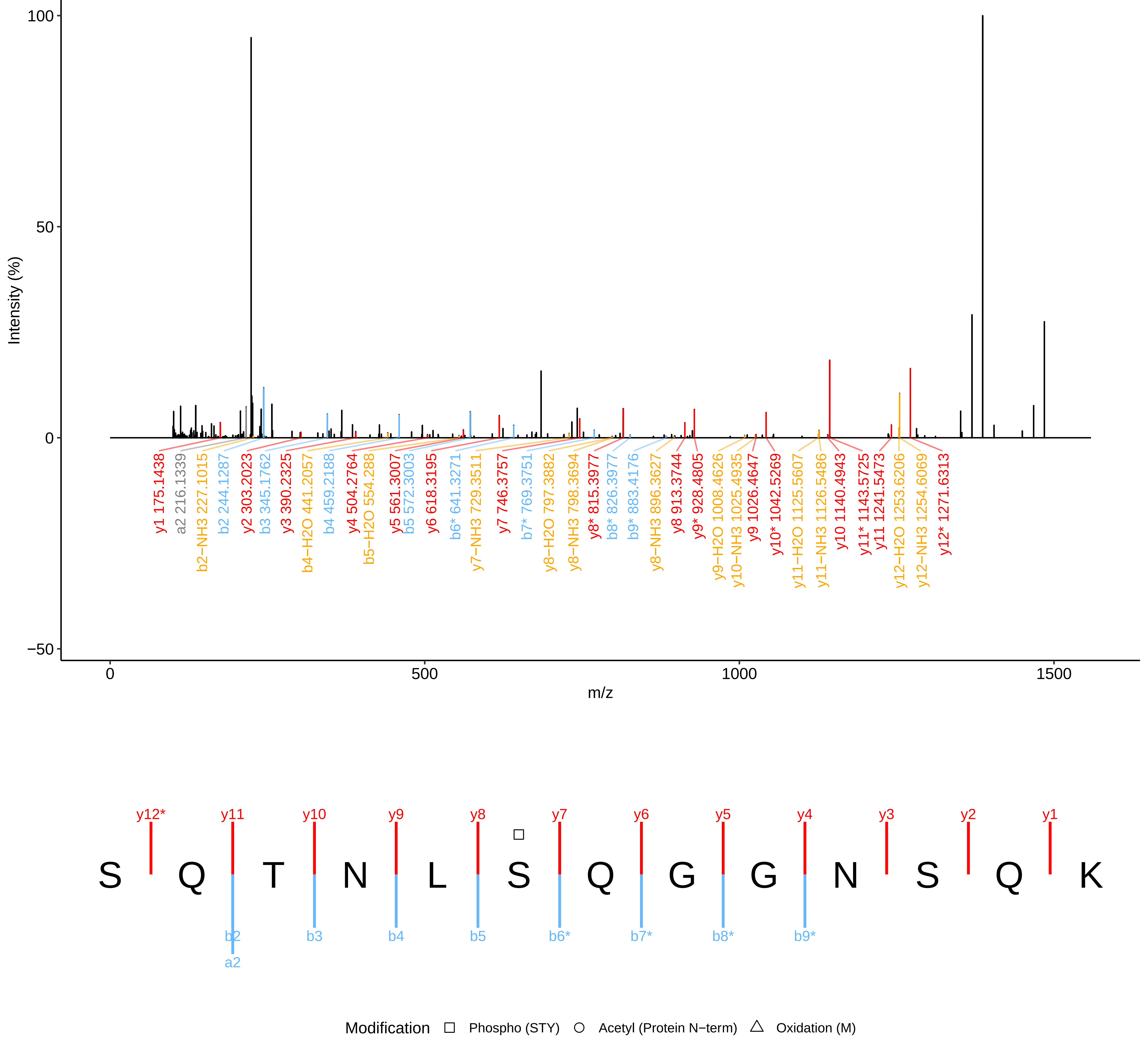

20210828_PS_SCy031_phospho_R3_TiO2_LC24h_F2 − Scan number: 14823

Gene Names	Charge	m/z	Mass	Mass error [Da]	Mass error [ppm]	Retention time	PEP	Score	Precursor Intensity
slr1783	3	642.6626	1924.966	−0.00049569	−0.77091	21.634	4.3993e−07	135.54	3706945

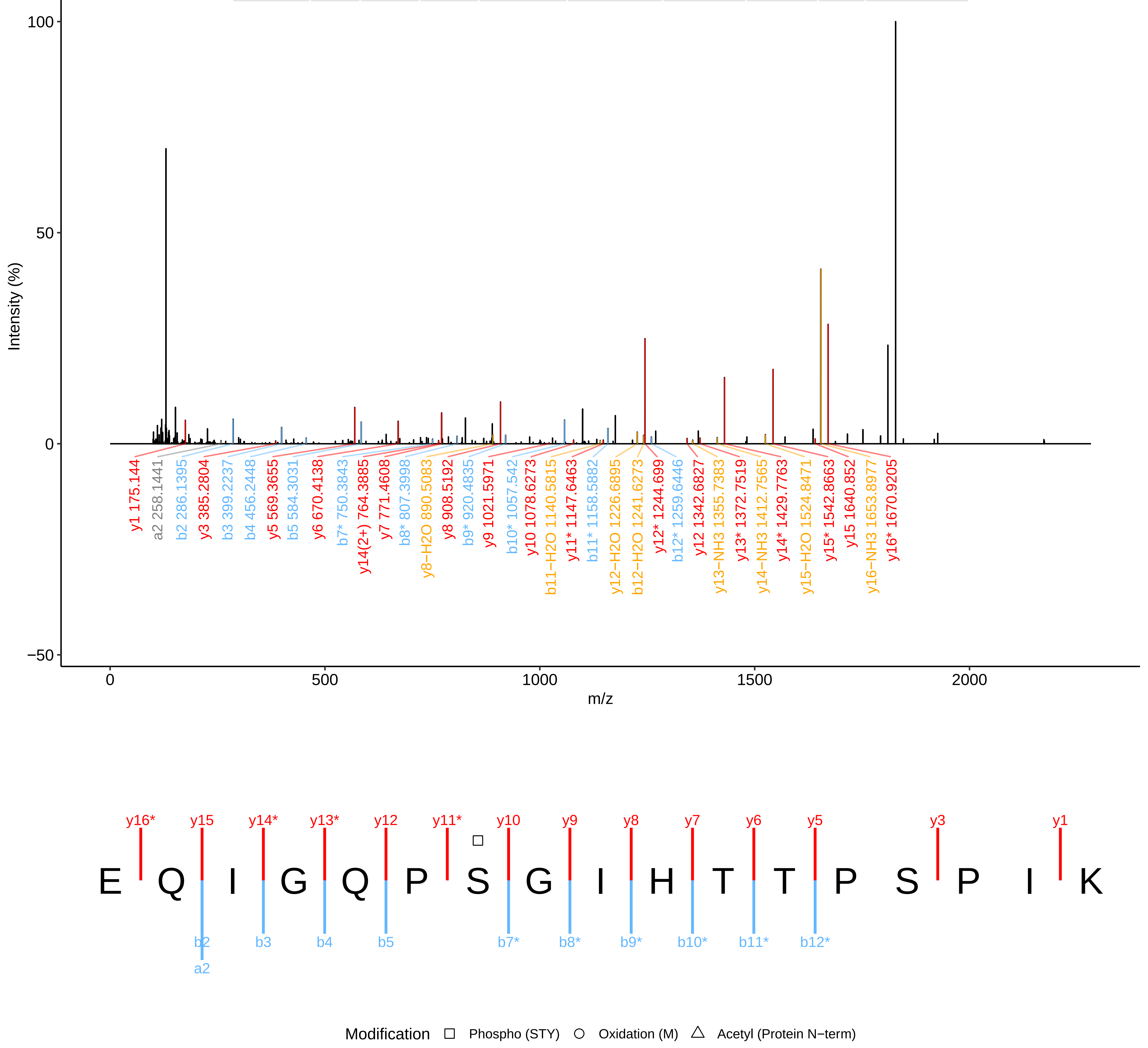

20210828_PS_SCy031_phospho_R2_TiO2_LC3h_F3 − Scan number: 13936

Gene Names	Charge	m/z	Mass	Mass error [Da]	Mass error [ppm]	Retention time	PEP	Score	Precursor Intensity
slr1783	3	645.346	1933.016	0.0010508	1.6274	20.661	6.6789e−05	114.88	3678312

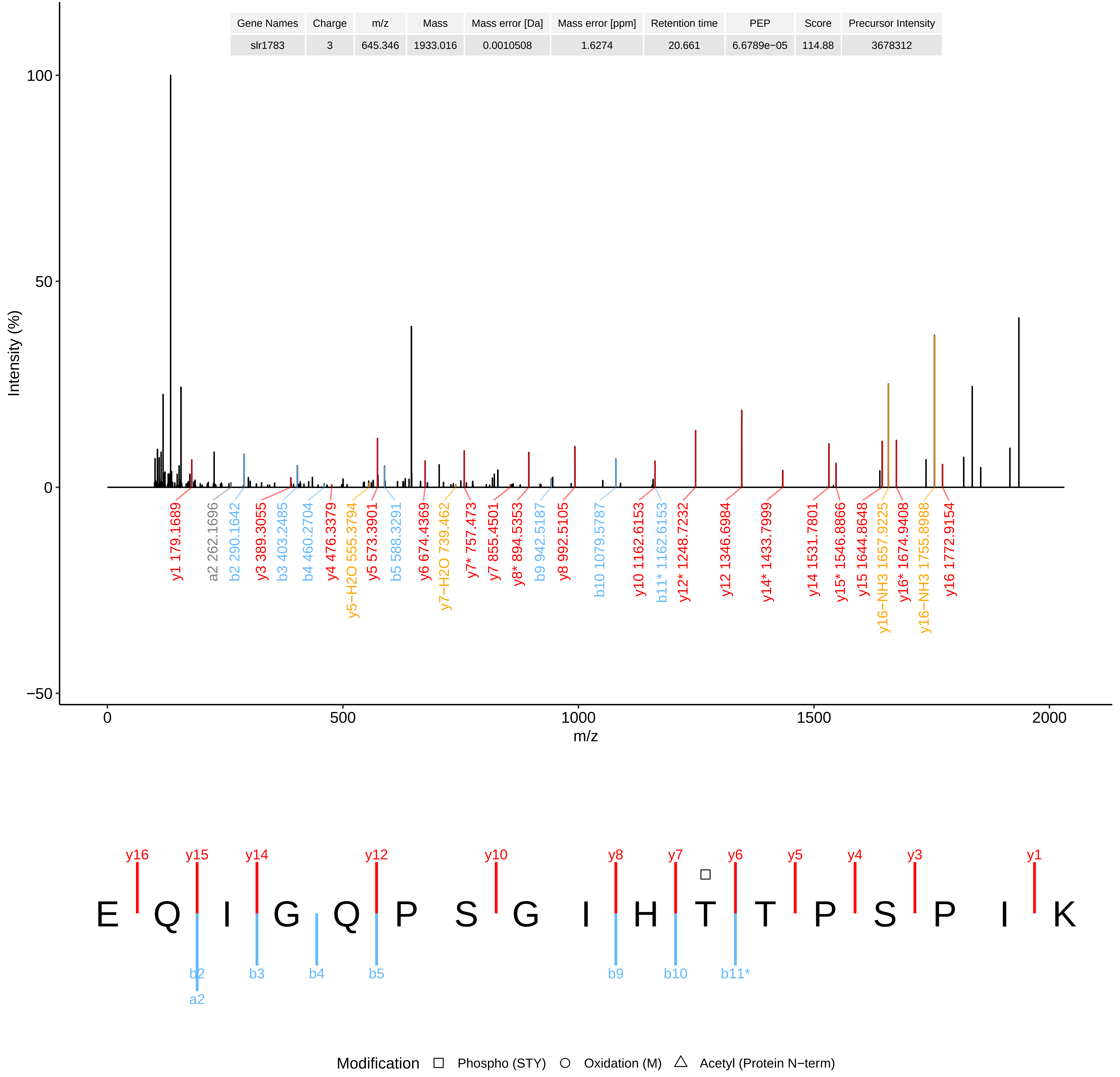

20210828_PS_SCy031_phospho_R3_TiO2_LC3h_F2 − Scan number: 11093

Gene Names	Charge	m/z	Mass	Mass error [Da]	Mass error [ppm]	Retention time	PEP	Score	Precursor Intensity
slr1789	2	574.8199	1147.625	−0.0019463	−3.4956	16.924	0.00065013	83.614	19576960

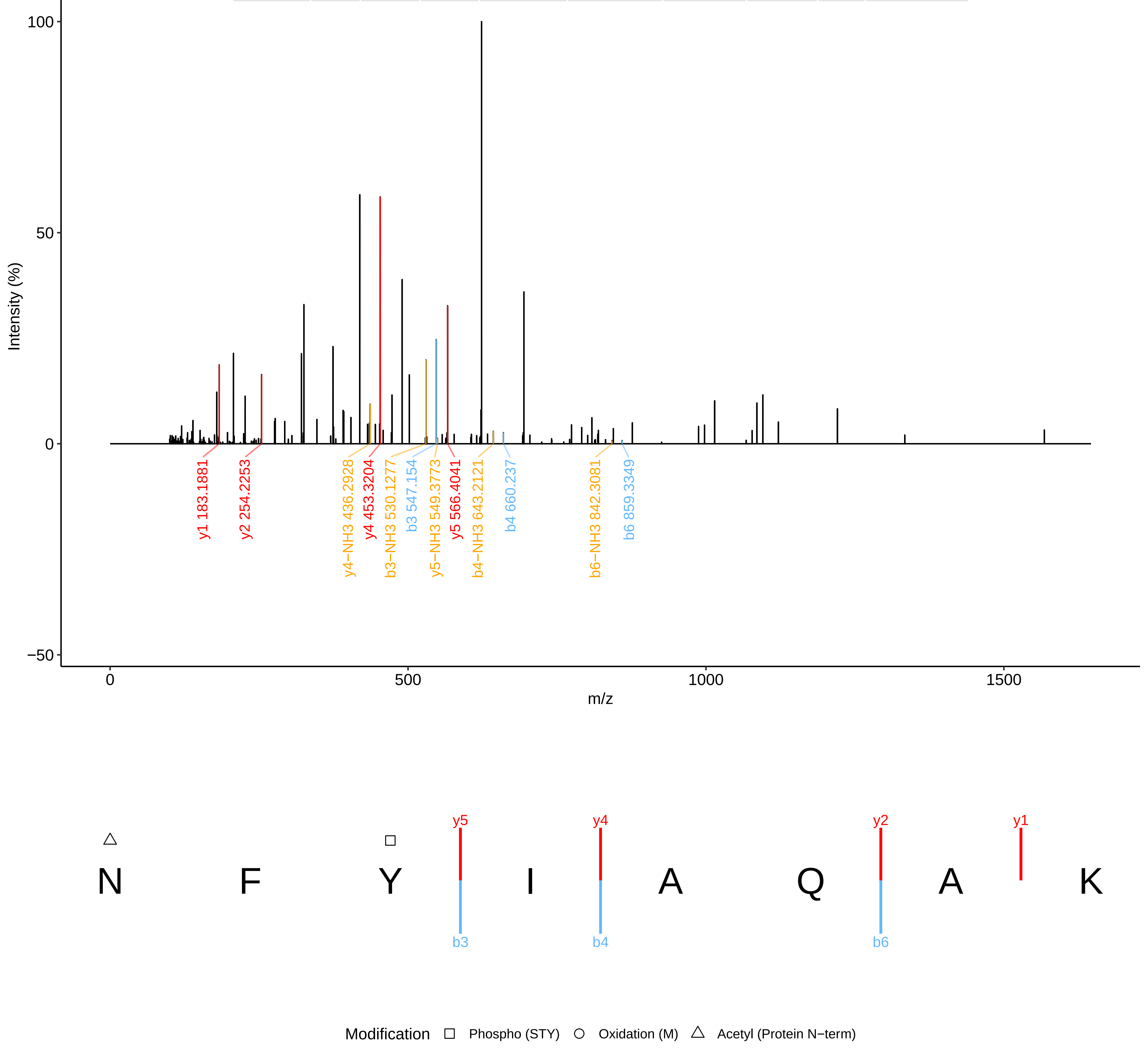

20210823_PS_SCy031_R1_phospho_HC_TiO2_1 − Scan number: 23792

Gene Names	Charge	m/z	Mass	Mass error [Da]	Mass error [ppm]	Retention time	PEP	Score	Precursor Intensity
slr1841	3	1298.978	3893.912	NA	NA	32.776	9.6368e−09	76.51	2383789

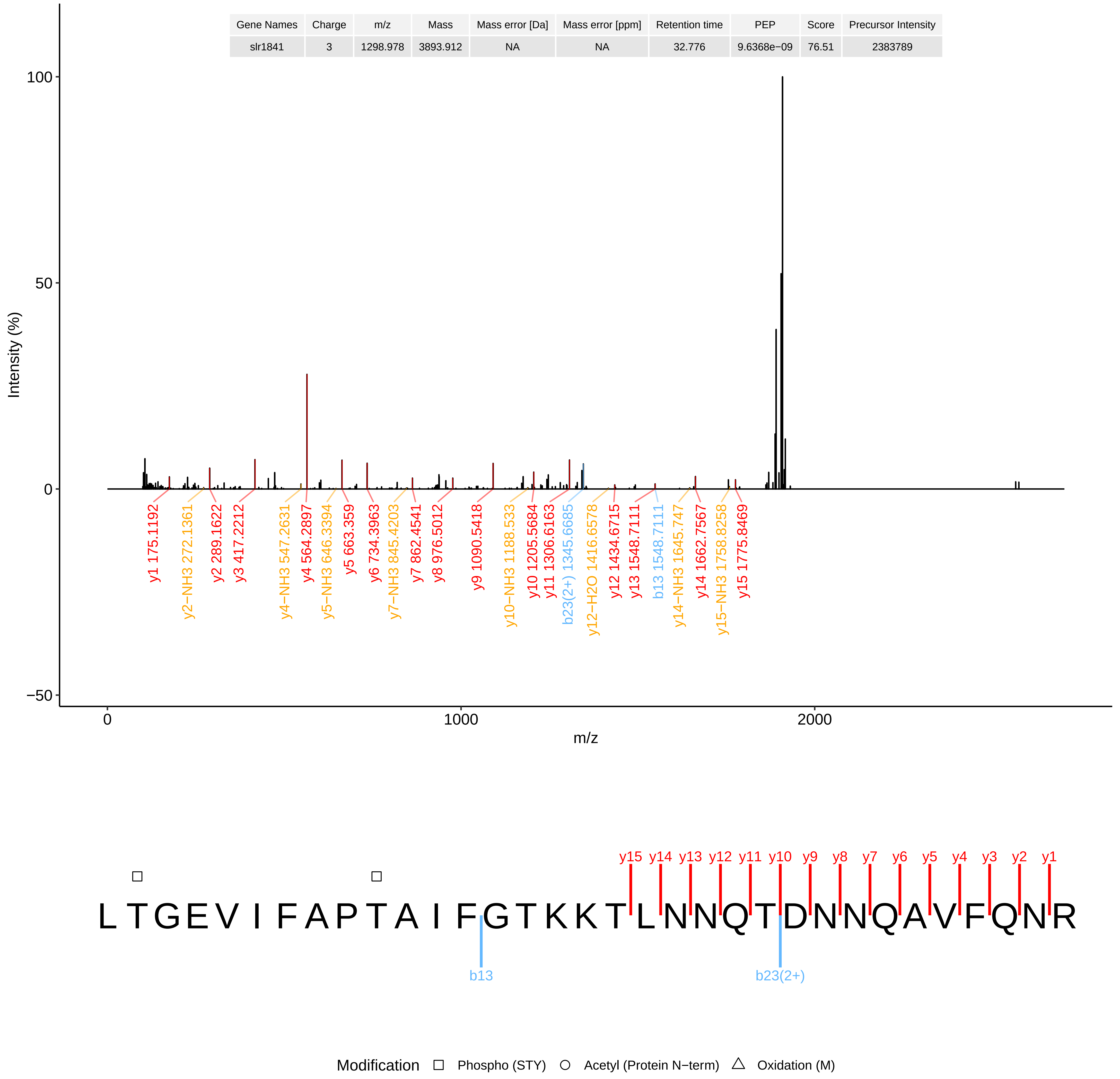

20210828_PS_SCy031_phospho_R3_TiO2_LC3h_F1 − Scan number: 13076

Gene Names	Charge	m/z	Mass	Mass error [Da]	Mass error [ppm]	Retention time	PEP	Score	Precursor Intensity
slr1841	2	994.9598	1987.905	0.002101	2.1106	19.369	7.6073e−118	272.95	6668134

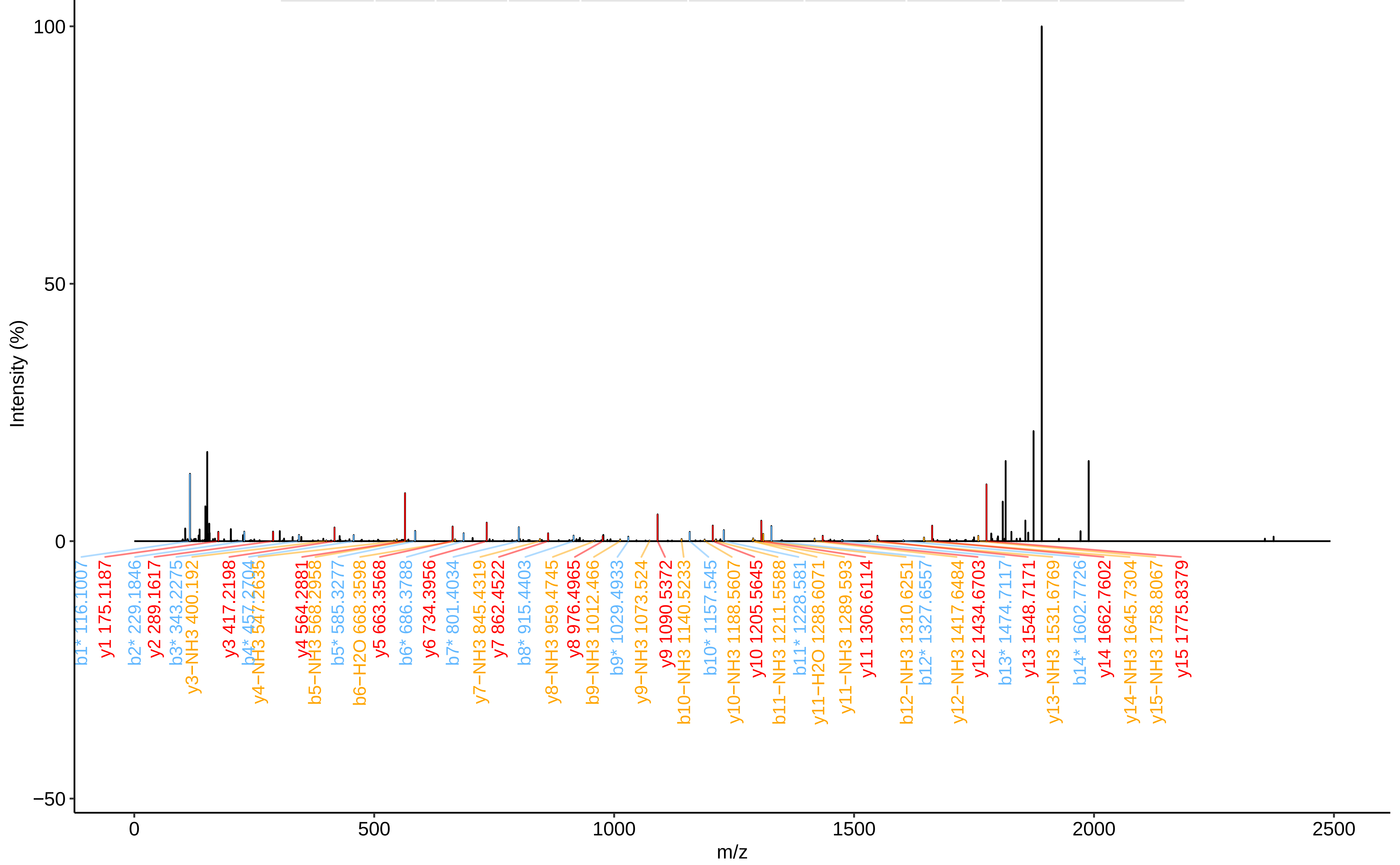

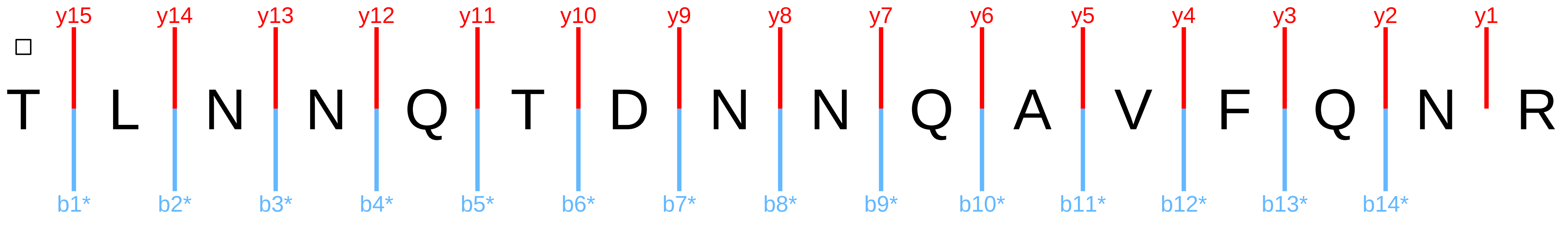

Modification □ Phospho (STY) ○ Oxidation (M) △ Acetyl (Protein N−term)

20210828_PS_SCy031_phospho_R3_TiO2_LC24h_F1 − Scan number: 12356

Gene Names	Charge	m/z	Mass	Mass error [Da]	Mass error [ppm]	Retention time	PEP	Score	Precursor Intensity
slr1841	2	996.9695	1991.924	NA	NA	18.288	0.0075263	96.26	NA

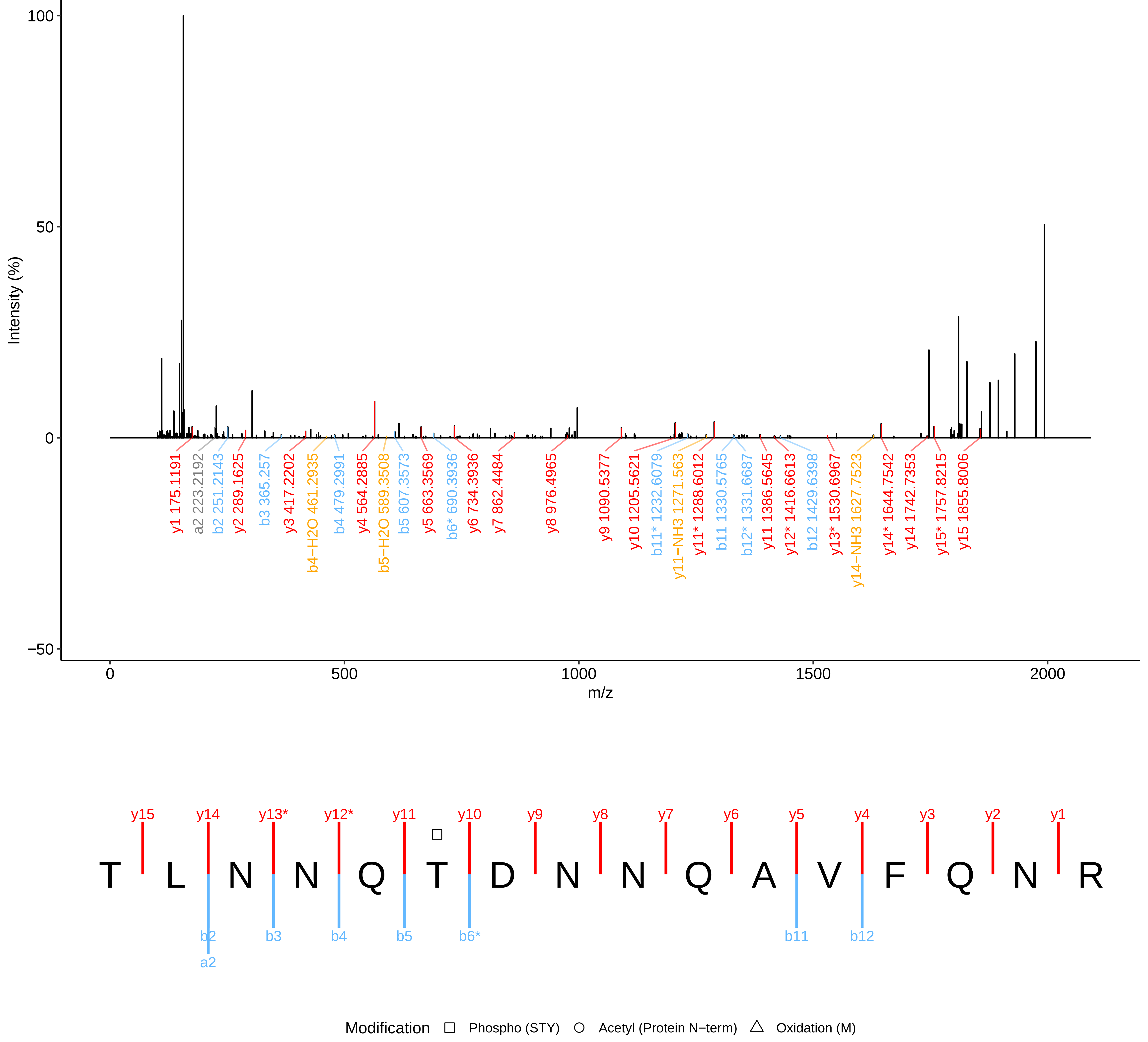

20210823_PS_SCy031_R1_phospho_LC3h_TiO2_2 − Scan number: 17881

Gene Names	Charge	m/z	Mass	Mass error [Da]	Mass error [ppm]	Retention time	PEP	Score	Precursor Intensity
slr1841	2	821.4165	1640.819	0.00066845	0.81328	25.496	2.3304e−14	160.43	8937615

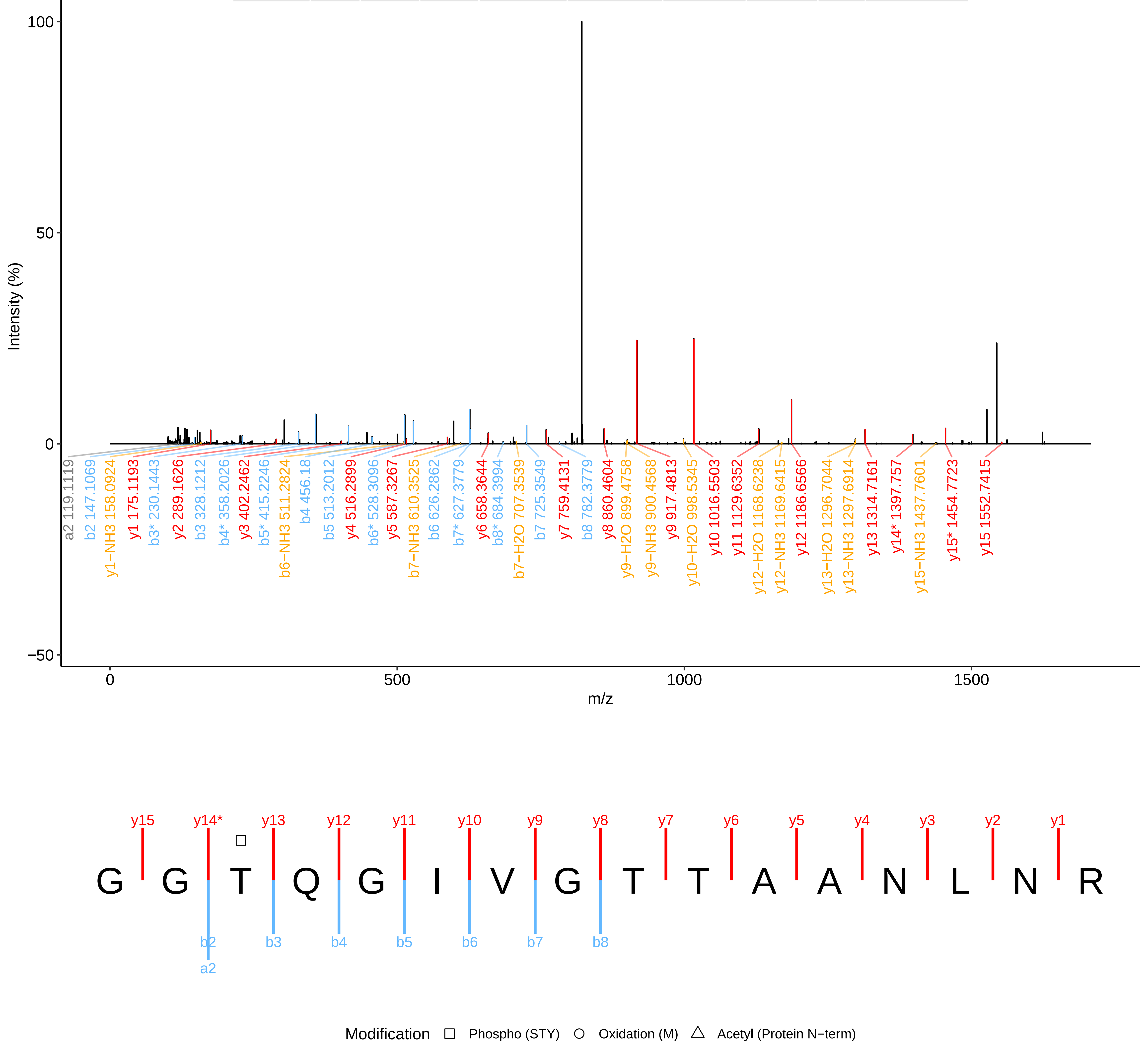

20210828_PS_SCy031_phospho_R2_TiO2_HC_F2 − Scan number: 37016

Gene Names	Charge	m/z	Mass	Mass error [Da]	Mass error [ppm]	Retention time	PEP	Score	Precursor Intensity
slr1856	2	975.4925	1948.97	0.00018316	0.18776	49.688	2.4559e−84	254.59	7656416

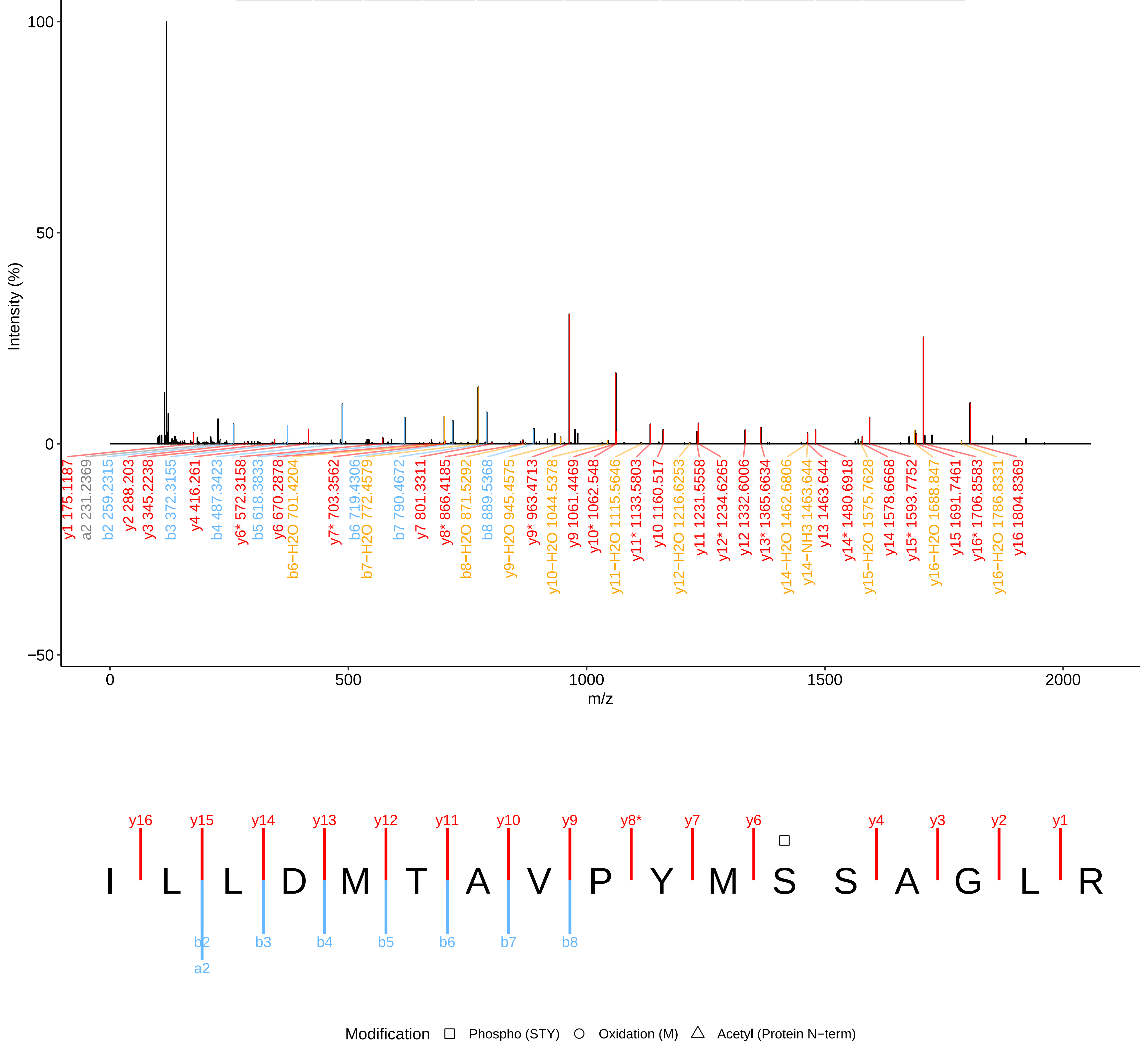

20210828_PS_SCy031_phospho_R3_TiO2_LC3h_F1 − Scan number: 36943

Gene Names	Charge	m/z	Mass	Mass error [Da]	Mass error [ppm]	Retention time	PEP	Score	Precursor Intensity
slr1856	2	973.48	1944.945	0.00051467	0.53641	49.77	1.0536e−183	307.92	25793800

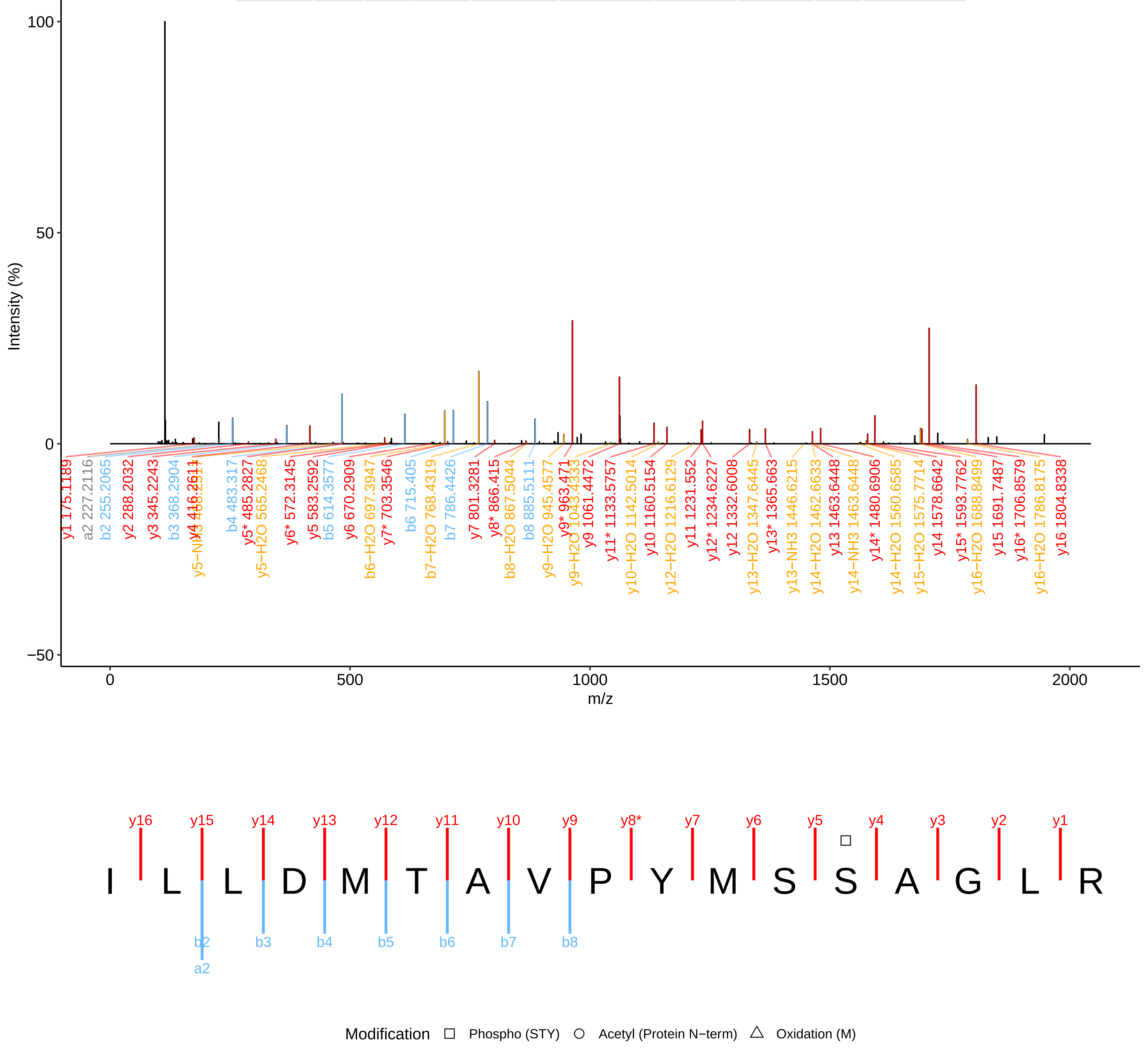

20210828_PS_SCy031_phospho_R2_TiO2_LC24h_F1 − Scan number: 33228

Gene Names	Charge	m/z	Mass	Mass error [Da]	Mass error [ppm]	Retention time	PEP	Score	Precursor Intensity
slr1859	2	1052.999	2103.984	0.0017845	1.6938	44.953	3.0683e−05	105.02	2672474

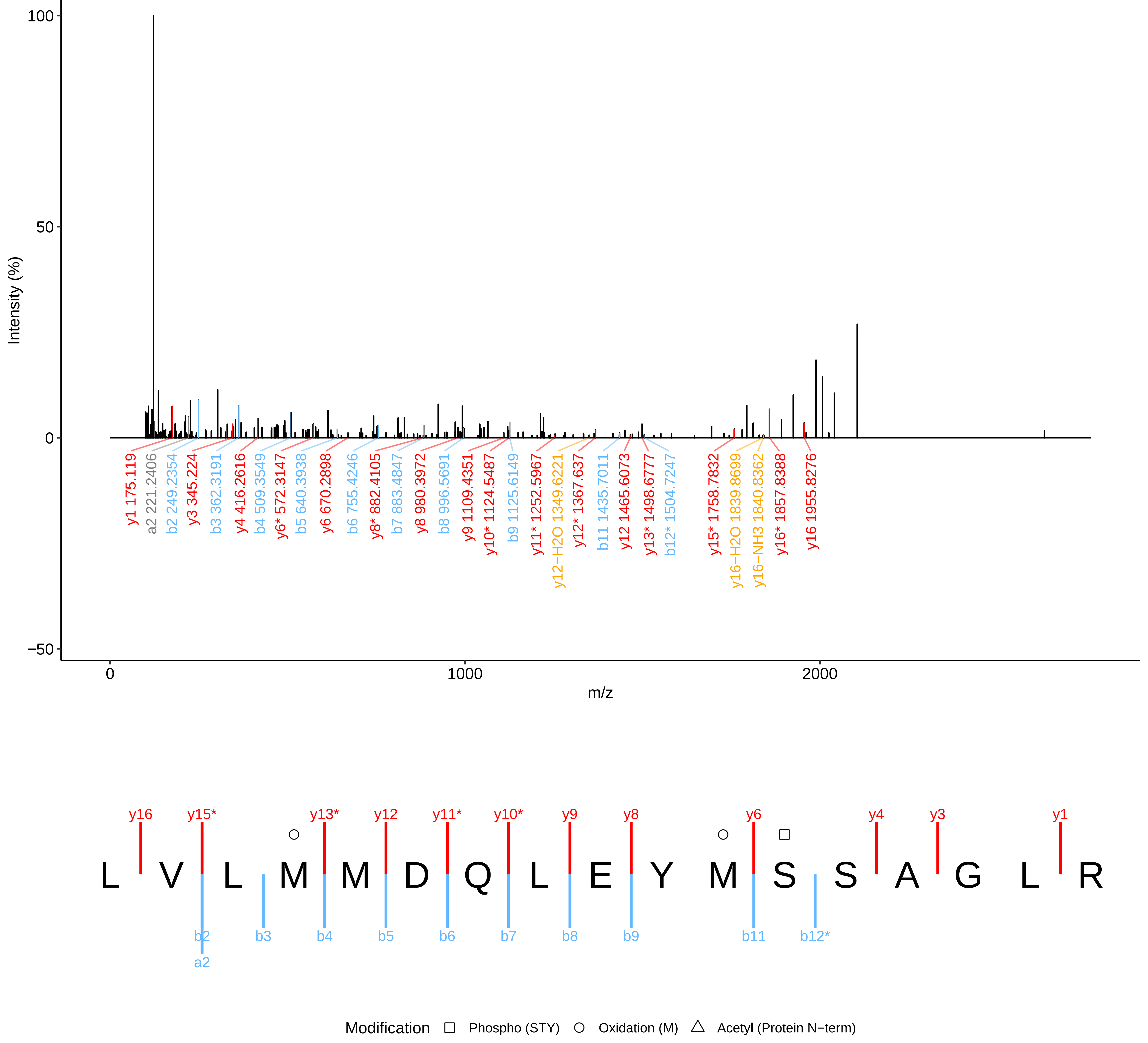

20210823_PS_SCy031_R1_phospho_HC_TiO2_1 − Scan number: 27346

Gene Names	Charge	m/z	Mass	Mass error [Da]	Mass error [ppm]	Retention time	PEP	Score	Precursor Intensity
slr1859	2	1058.987	2115.959	−0.00079783	−0.75268	37.292	3.4959e−12	155.88	NA

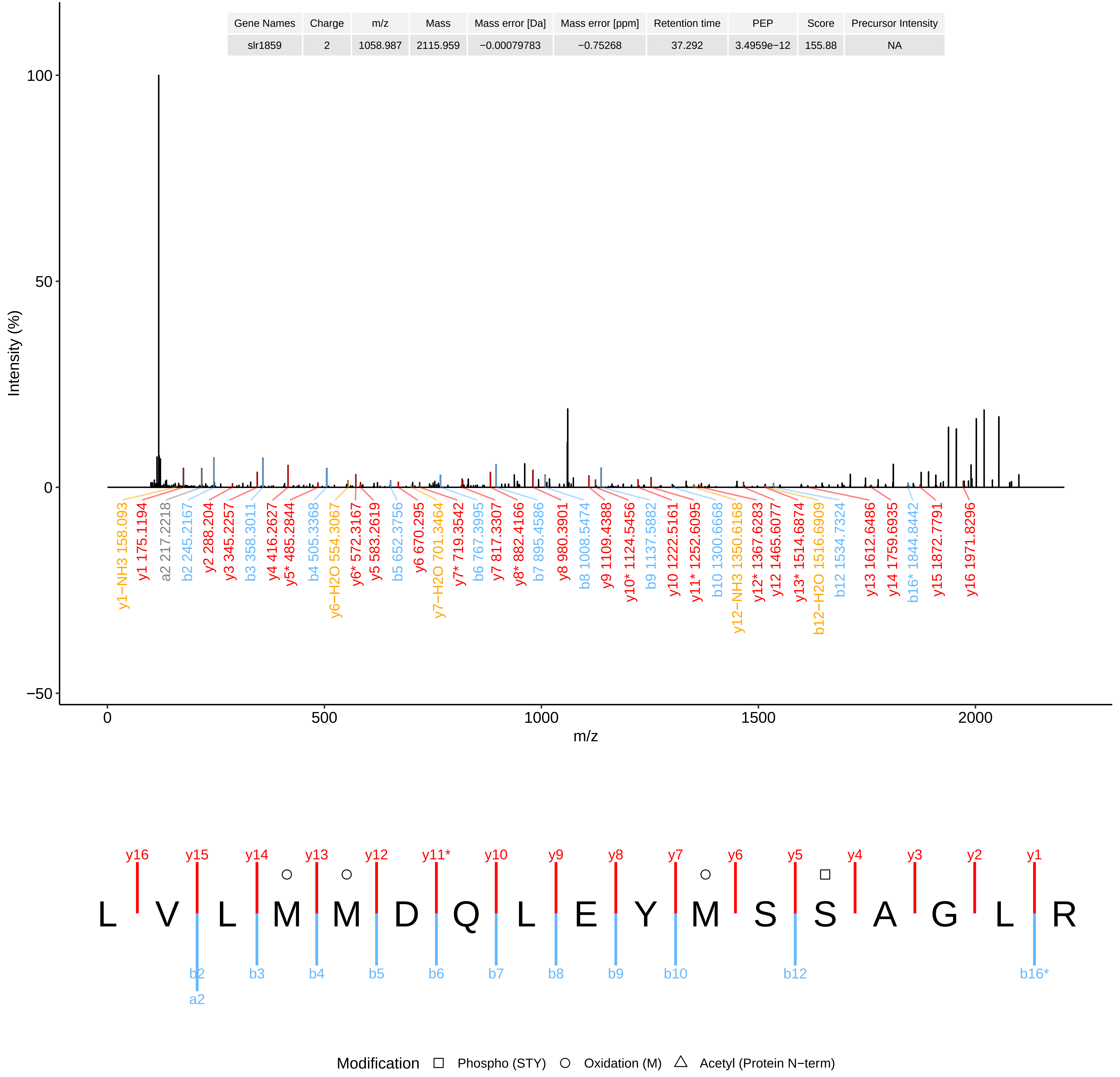

20211212_PS_SCy031_proteome_R1_HC_F5 − Scan number: 25347

Gene Names	Charge	m/z	Mass	Mass error [Da]	Mass error [ppm]	Retention time	PEP	Score	Precursor Intensity
slr1870	2	948.4897	1894.965	32.058	35604	38.083	0.0091023	44.203	10617052

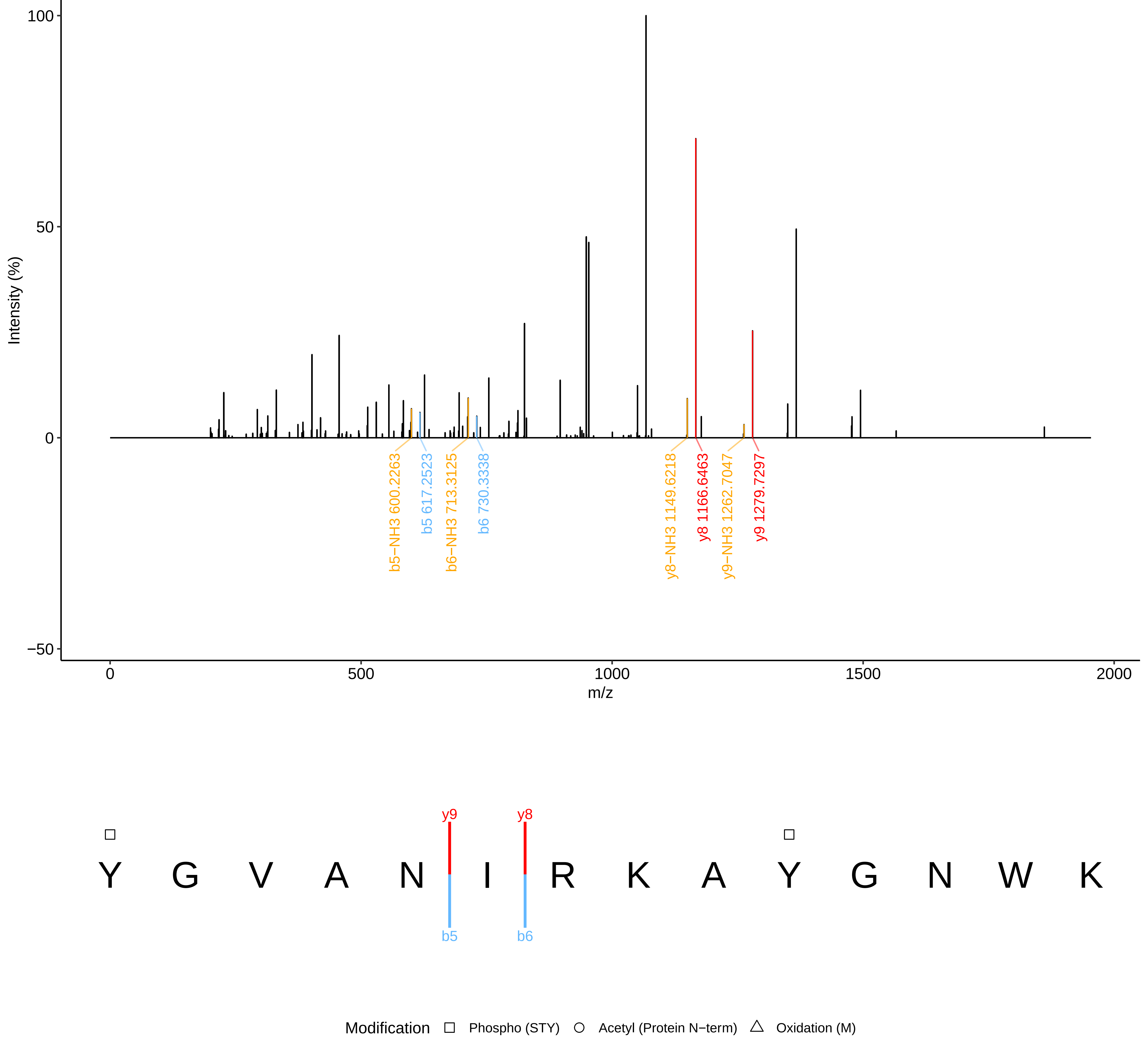

20210828_PS_SCy031_phospho_R3_TiO2_LC3h_F3 − Scan number: 16421

Gene Names	Charge	m/z	Mass	Mass error [Da]	Mass error [ppm]	Retention time	PEP	Score	Precursor Intensity
slr1945	2	904.9242	1807.834	NA	NA	23.736	0.00011603	125.98	2439484

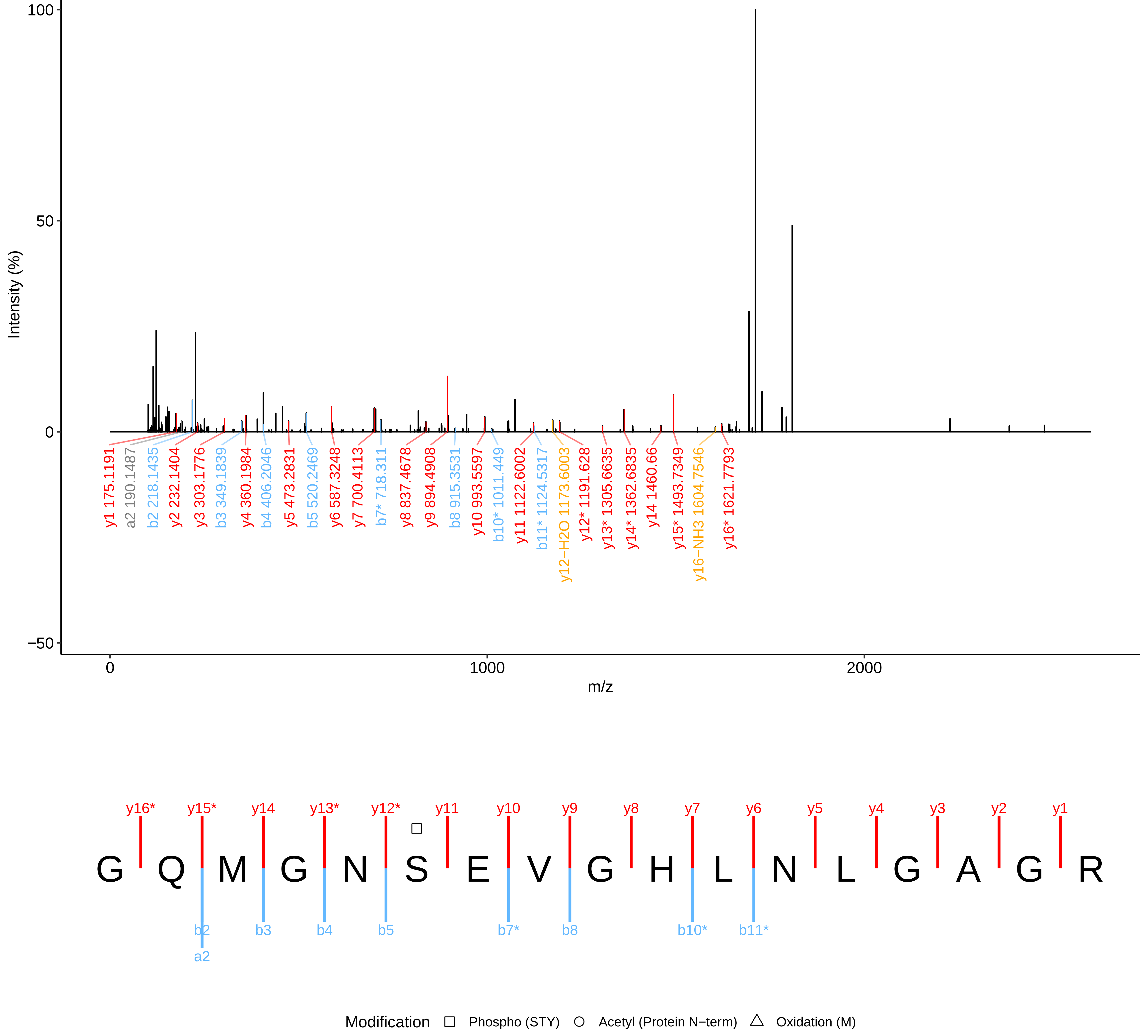

20210823_PS_SCy031_R1_phospho_LC24h_TiO2_3 − Scan number: 7735

Gene Names	Charge	m/z	Mass	Mass error [Da]	Mass error [ppm]	Retention time	PEP	Score	Precursor Intensity
slr1983	2	948.4302	1894.846	−0.0040638	−4.4152	12.741	0.00049409	66.19	11484297

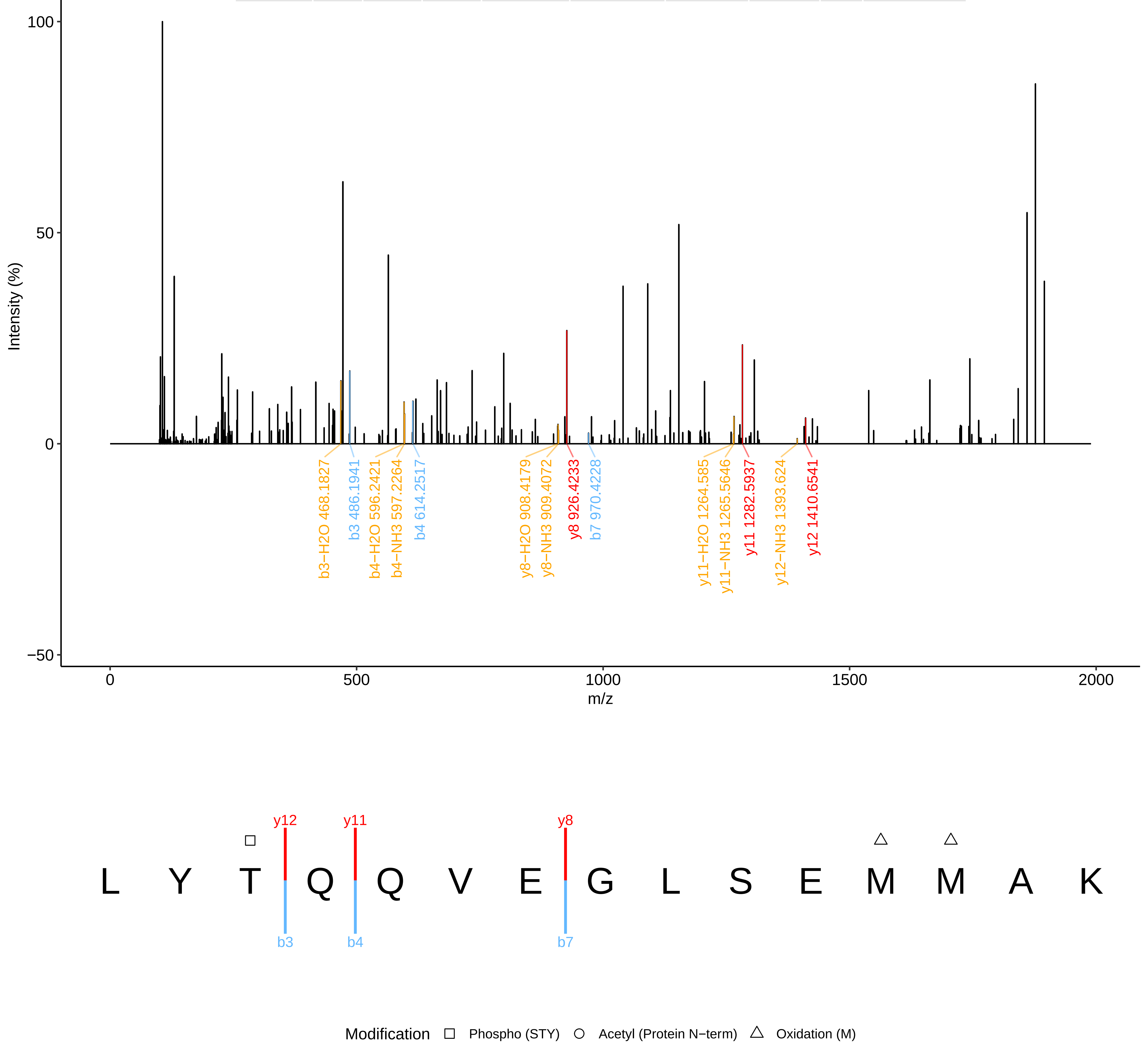

20210828_PS_SCy031_phospho_R2_TiO2_LC24h_F3 − Scan number: 30569

Gene Names	Charge	m/z	Mass	Mass error [Da]	Mass error [ppm]	Retention time	PEP	Score	Precursor Intensity
slr1984	2	926.453	1850.891	NA	NA	41.562	0.00071316	68.845	NA

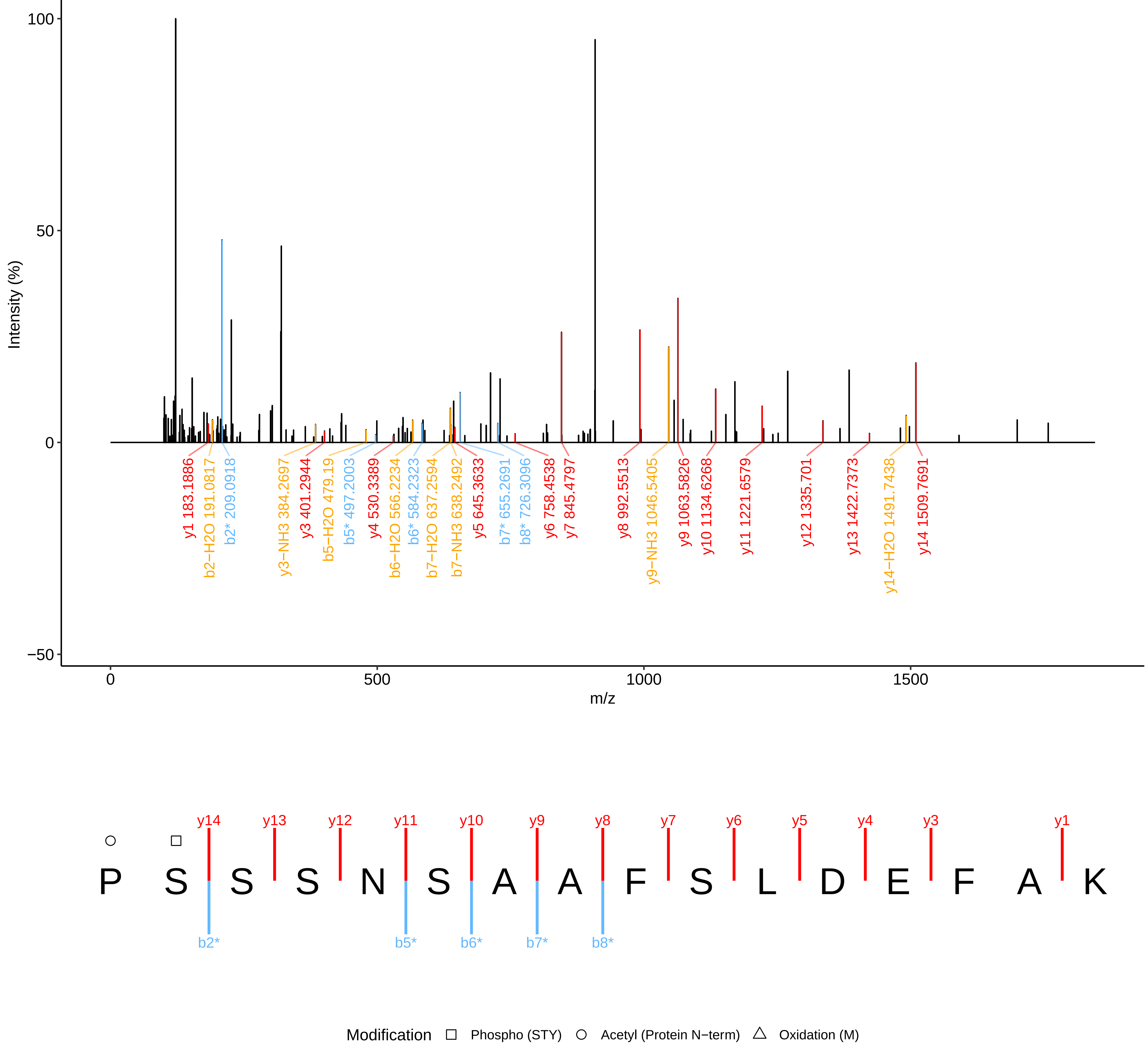

20211217_PS_SCy031_Proteome_R2_24hLC_F3 − Scan number: 28559

Gene Names	Charge	m/z	Mass	Mass error [Da]	Mass error [ppm]	Retention time	PEP	Score	Precursor Intensity
slr1986	2	986.0076	1970.001	NA	NA	40.843	0.0019692	59.198	NA

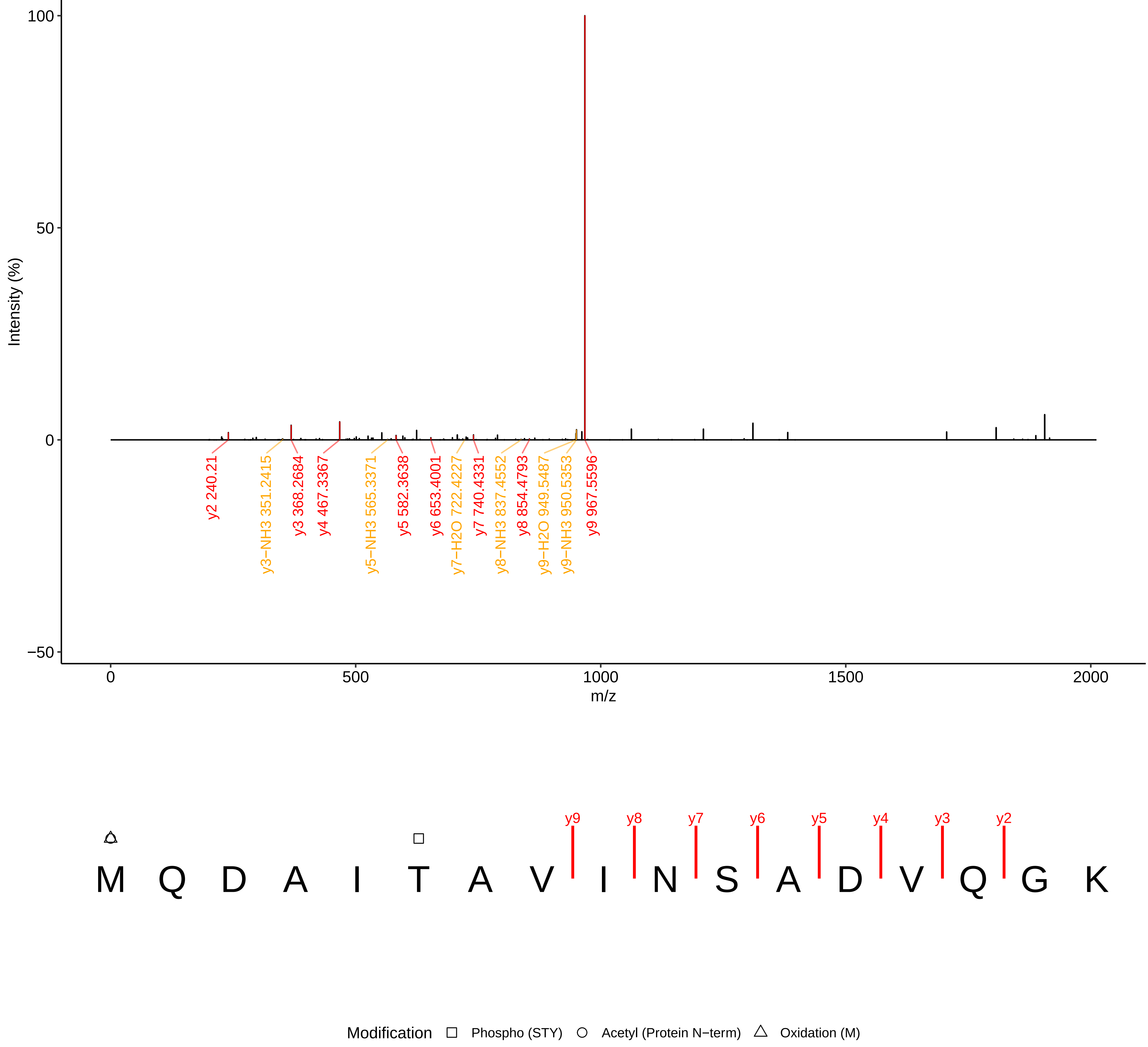

20210828_PS_SCy031_phospho_R2_TiO2_LC24h_F1 − Scan number: 26020

Gene Names	Charge	m/z	Mass	Mass error [Da]	Mass error [ppm]	Retention time	PEP	Score	Precursor Intensity
slr1986	2	957.0048	1911.995	0.00077786	0.81281	35.789	1.5205e−84	256.13	4233258

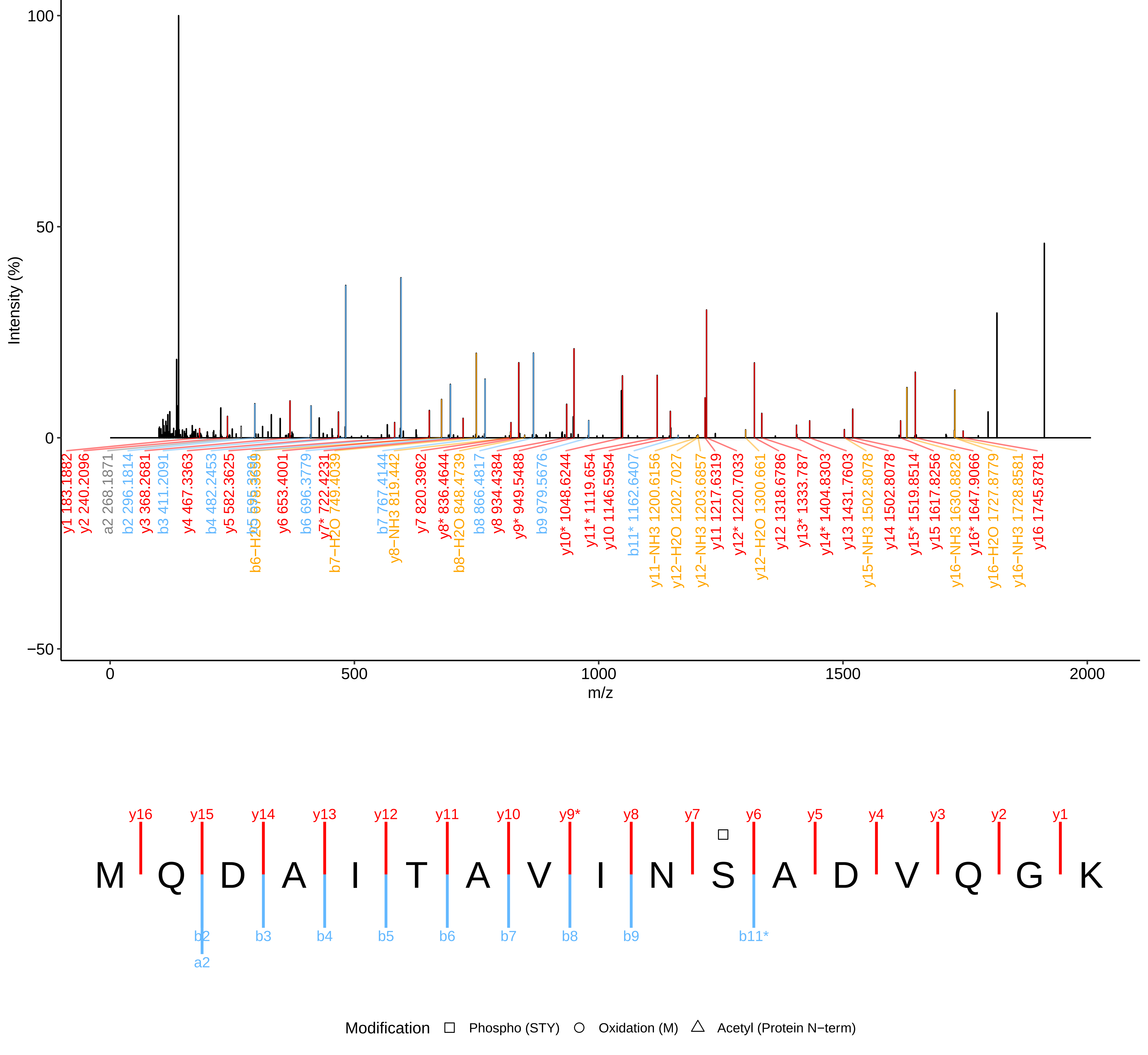

20210828_PS_SCy031_phospho_R3_TiO2_LC3h_F1 − Scan number: 21373

Gene Names	Charge	m/z	Mass	Mass error [Da]	Mass error [ppm]	Retention time	PEP	Score	Precursor Intensity
slr1986	2	734.4415	1466.868	0.00057476	0.78258	29.868	1.0528e−06	148.33	5392586

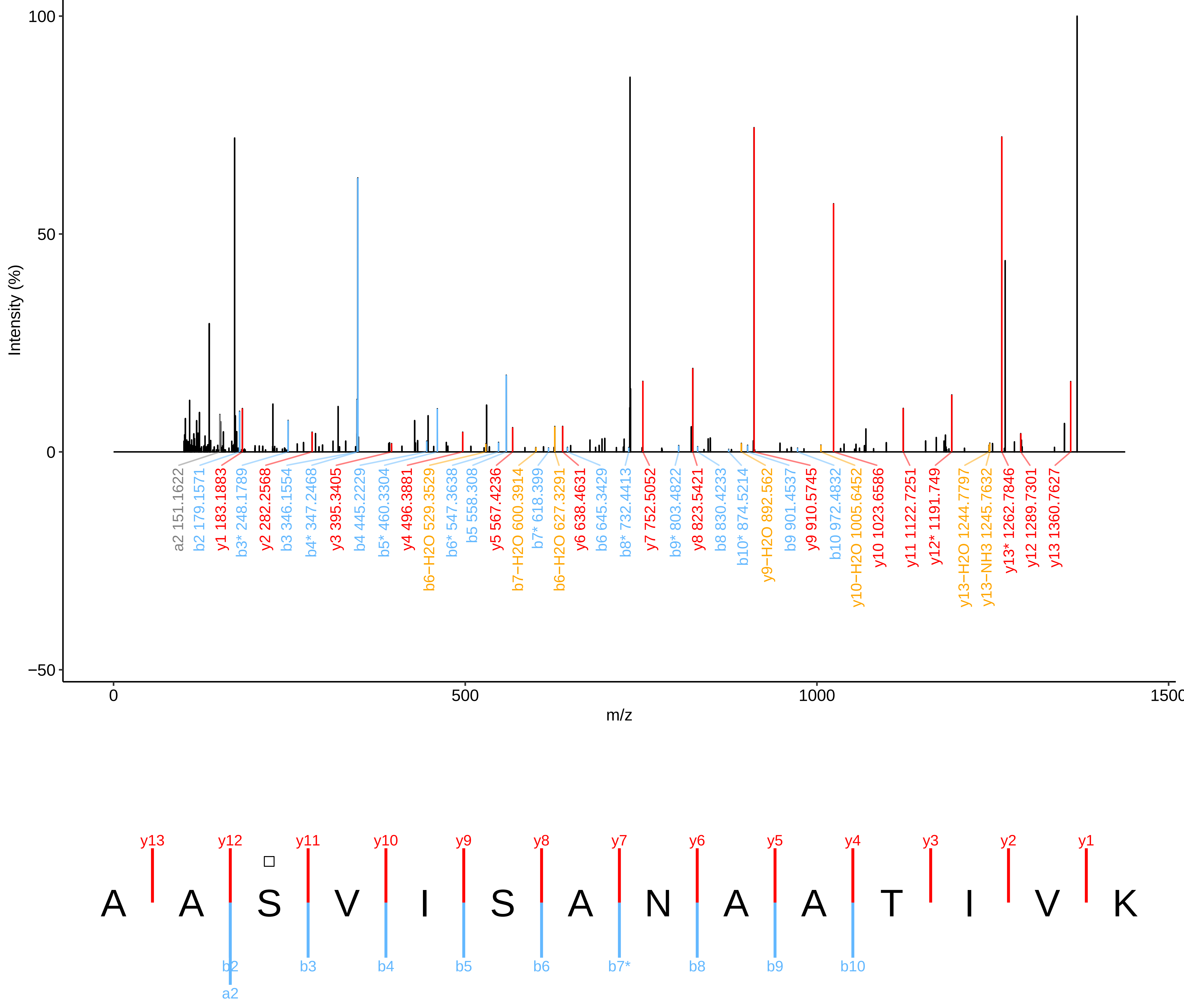

Modification □ Phospho (STY) ○ Oxidation (M) △ Acetyl (Protein N−term)

20210828_PS_SCy031_phospho_R2_TiO2_LC24h_F3 − Scan number: 36756

Gene Names	Charge	m/z	Mass	Mass error [Da]	Mass error [ppm]	Retention time	PEP	Score	Precursor Intensity
slr1986	3	788.4285	2362.264	−0.00065835	−0.85828	49.447	3.2017e−29	140.4	18480236

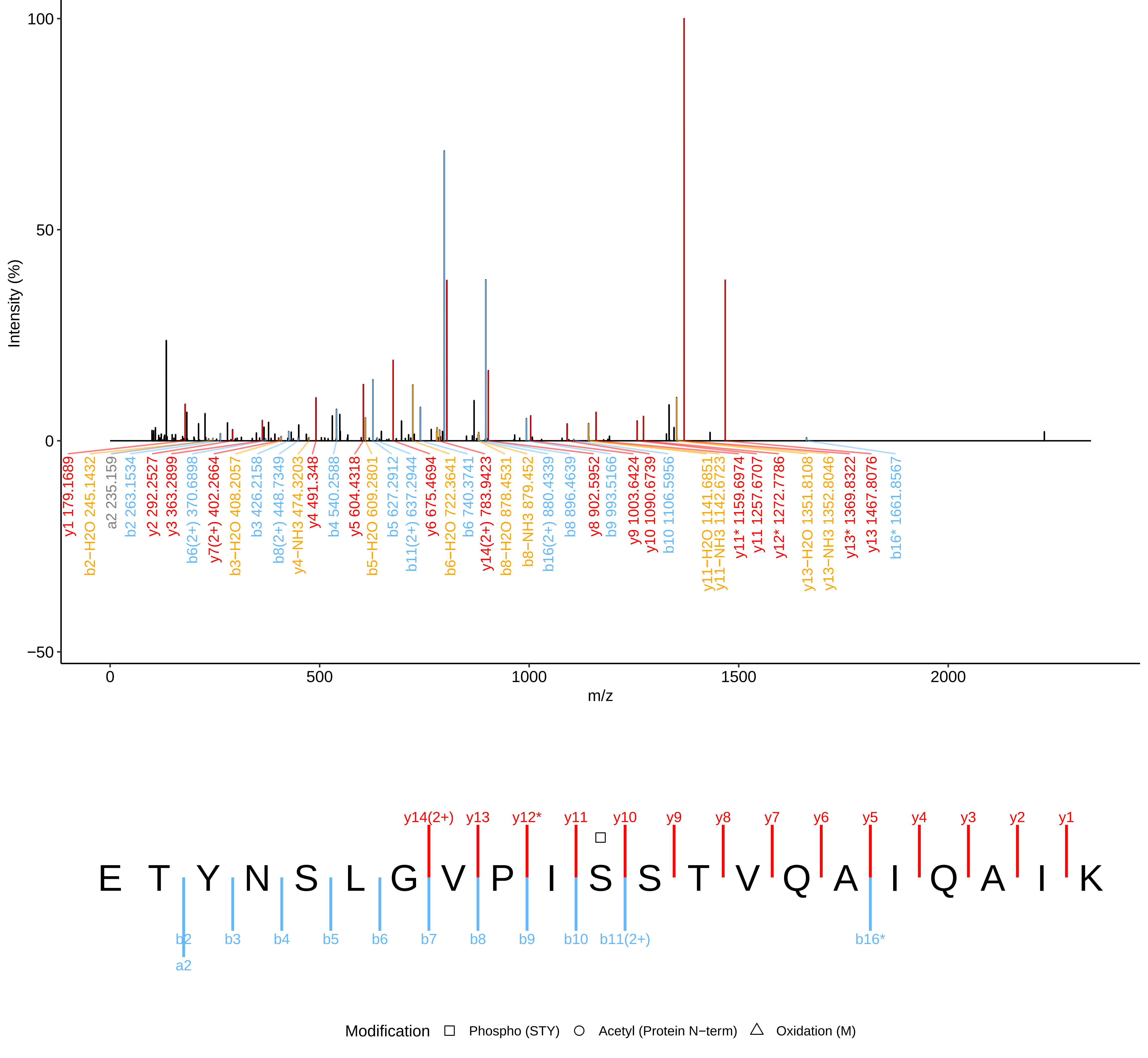

20210828_PS_SCy031_phospho_R2_TiO2_LC24h_F1 − Scan number: 29408

Gene Names	Charge	m/z	Mass	Mass error [Da]	Mass error [ppm]	Retention time	PEP	Score	Precursor Intensity
slr1986	4	954.9086	3815.606	0.0011341	1.1861	40.1	0.00010971	66.825	3281789

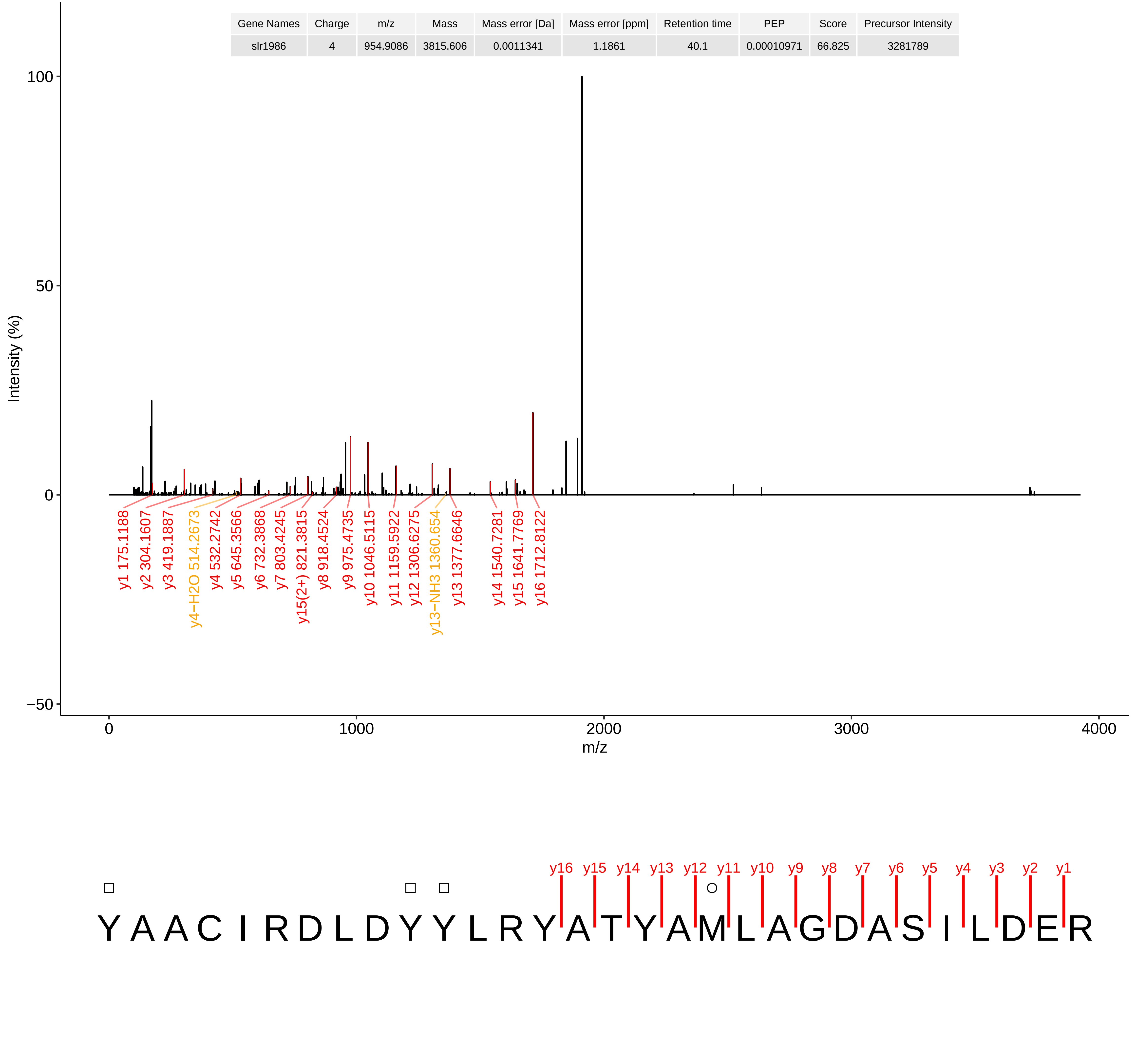

Modification □ Phospho (STY) ○ Oxidation (M) △ Acetyl (Protein N−term)

20210828_PS_SCy031_phospho_R3_TiO2_HC_F1 − Scan number: 5614

Gene Names	Charge	m/z	Mass	Mass error [Da]	Mass error [ppm]	Retention time	PEP	Score	Precursor Intensity
slr1994	2	696.3607	1390.707	NA	NA	9.94	0.11002	104.9	NA

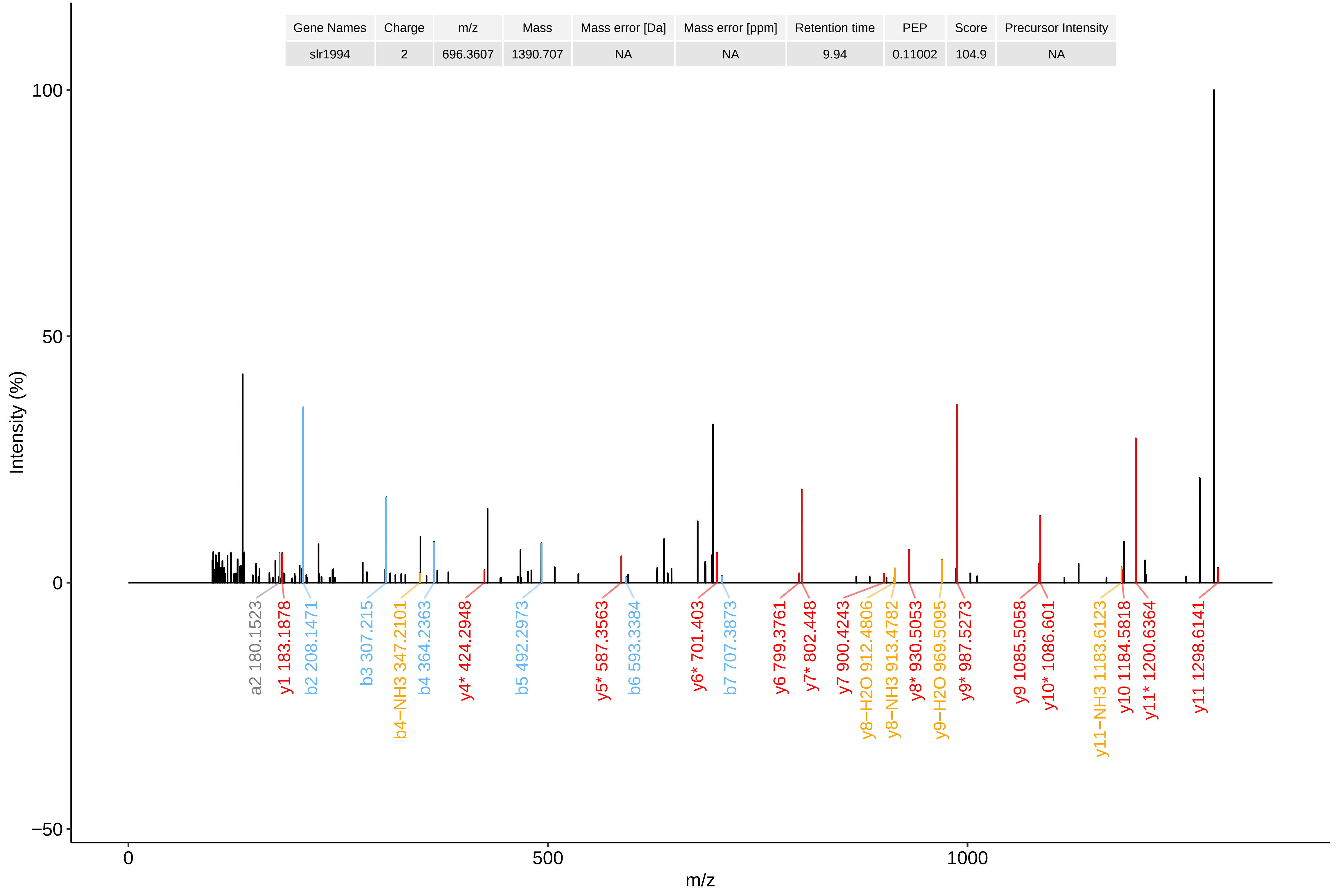

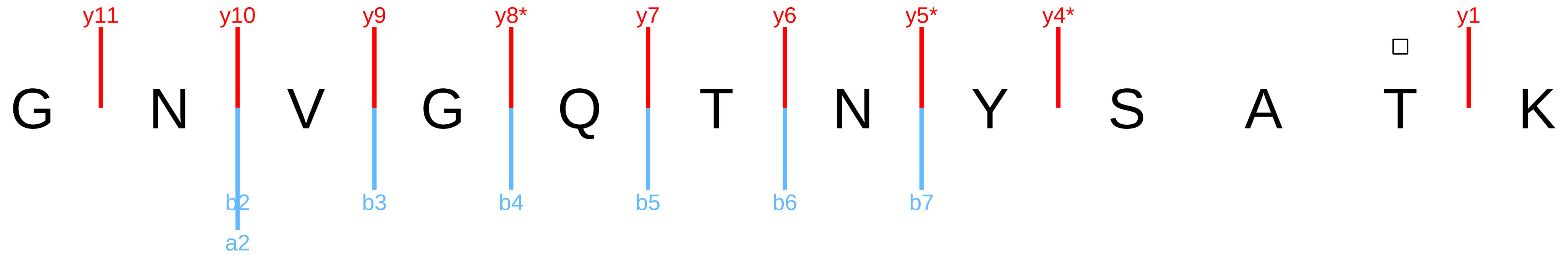

Modification □ Phospho (STY) ○ Acetyl (Protein N−term) △ Oxidation (M)

20210828_PS_SCy031_phospho_R3_TiO2_LC3h_F3 − Scan number: 9020

Gene Names	Charge	m/z	Mass	Mass error [Da]	Mass error [ppm]	Retention time	PEP	Score	Precursor Intensity
slr2067	2	581.2883	1160.562	−0.00020655	−0.35533	14.418	0.01929	113.24	4716956

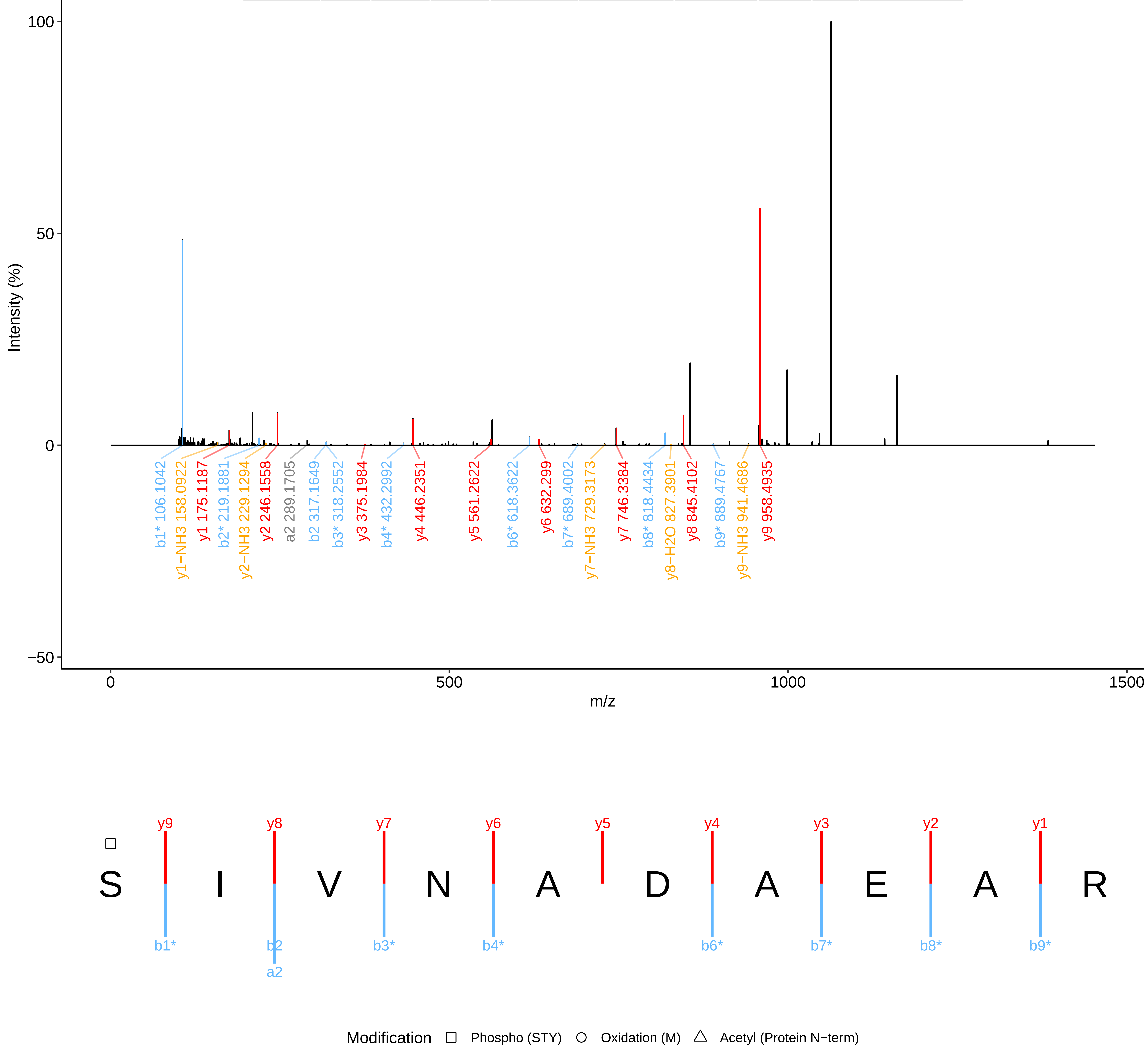

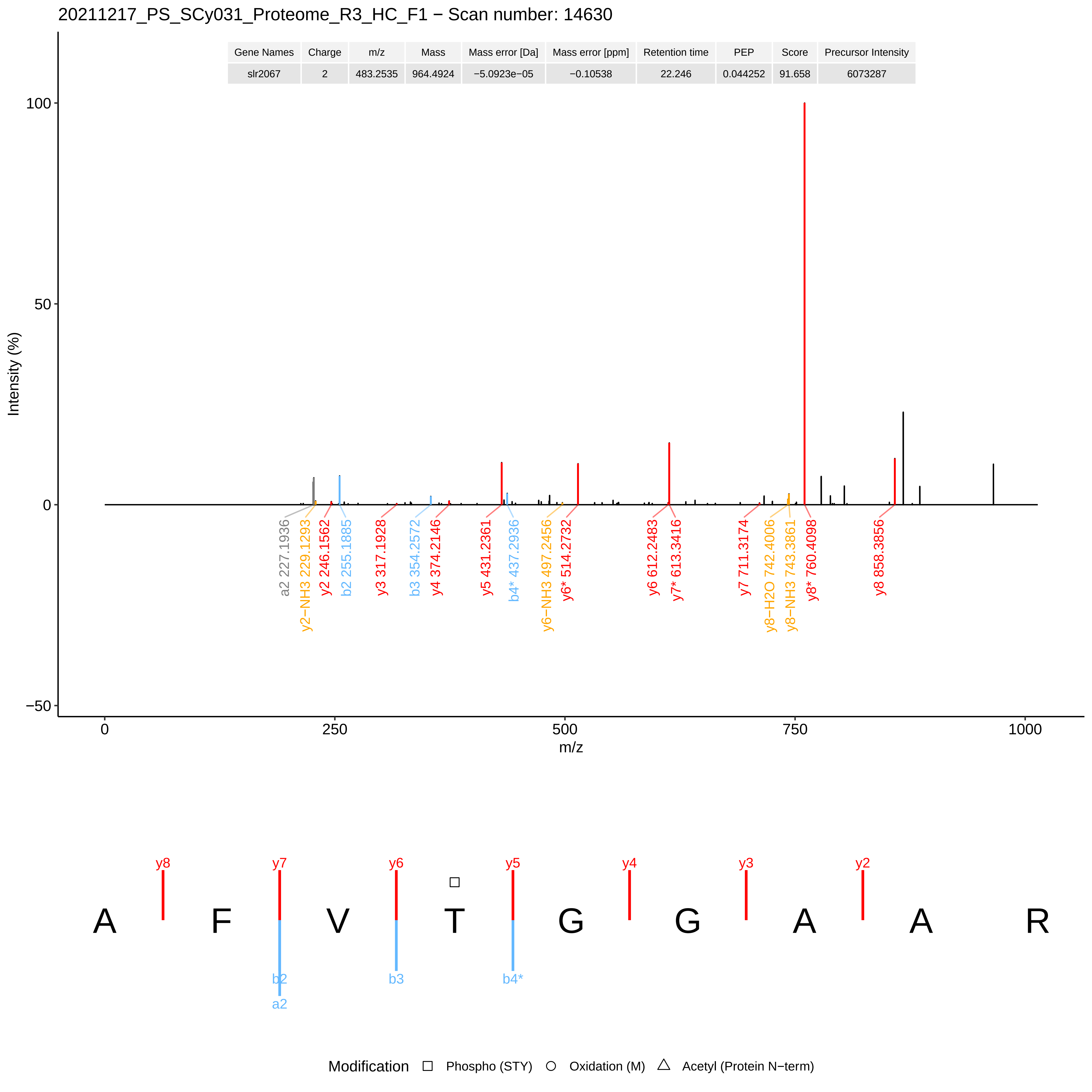

20211217_PS_SCy031_Proteome_R3_HC_F1 − Scan number: 14630
Gene Names | Charge | m/z | Mass | Mass error [Da] | Mass error [ppm] | Retention time | PEP | Score | Precursor Intensity
slr2067 | 2 | 483.2535 | 964.4924 | −5.0923e−05 | −0.10538 | 22.246 | 0.044252 | 91.658 | 6073287
Intensity (%)
100
50
0
−50
0
250
500
750
1000
m/z
a2 227.1936
y2−NH3 229.1293
y2 246.1562
b2 255.1885
y3 317.1928
b3 354.2572
y4 374.2146
y5 431.2361
b4* 437.2936
y6−NH3 497.2456
y6* 514.2732
y6 612.2483
y7* 613.3416
y7 711.3174
y8−H2O 742.4006
y8−NH3 743.3861
y8* 760.4098
y8 858.3856
y8
y7
y6
y5
y4
y3
y2
A F V T G G A A R
b2
a2
b3
b4*
Modification
Phospho (STY)
Oxidation (M)
Acetyl (Protein N−term)

20211212_PS_SCy031_proteome_R1_HC_F4 − Scan number: 37744

Gene Names	Charge	m/z	Mass	Mass error [Da]	Mass error [ppm]	Retention time	PEP	Score	Precursor Intensity
slr2067	3	1226.208	3675.603	NA	NA	53.445	0.00080879	55.046	NA

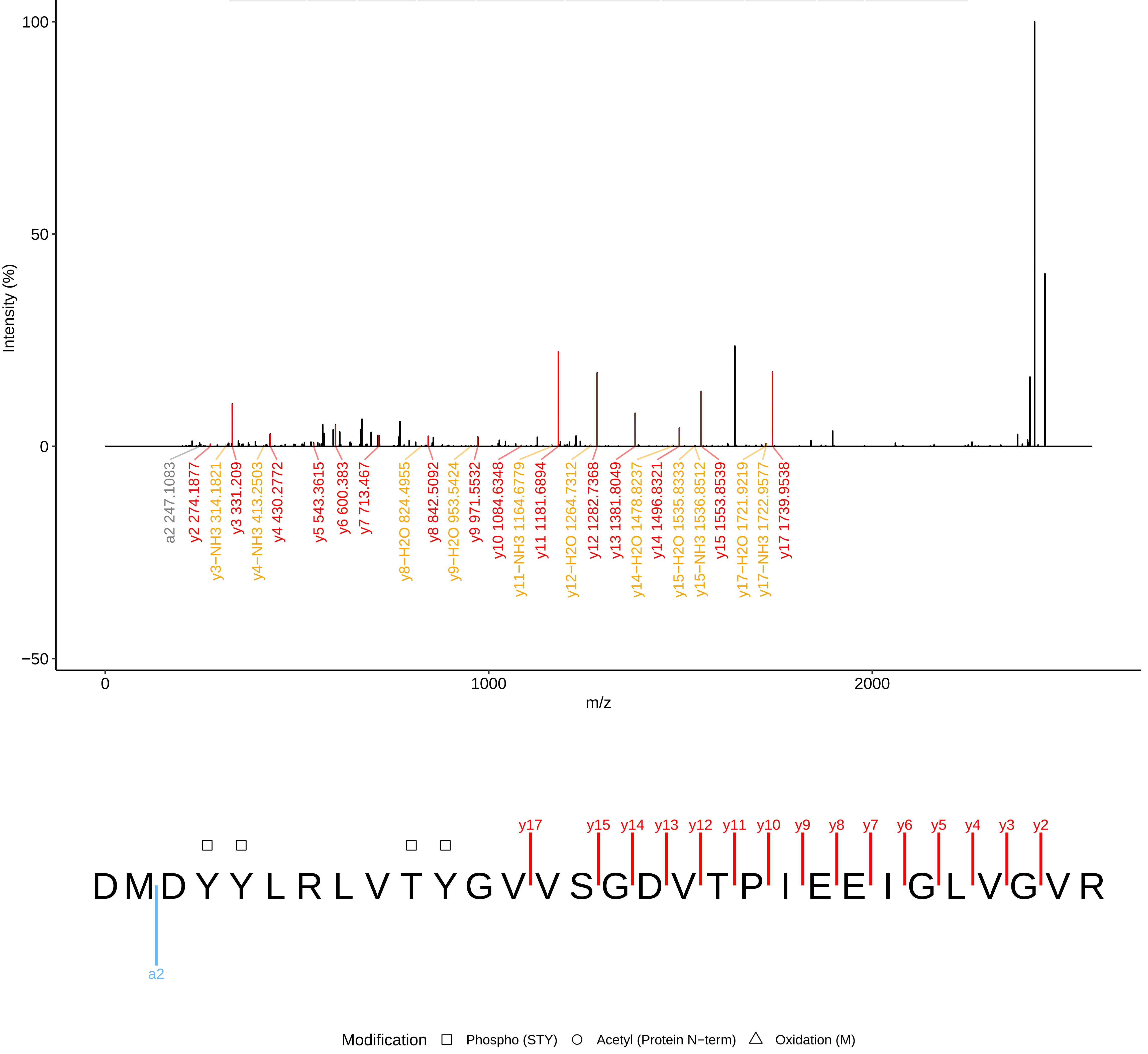

20210828_PS_SCy031_phospho_R3_TiO2_LC3h_F2 − Scan number: 32049

Gene Names	Charge	m/z	Mass	Mass error [Da]	Mass error [ppm]	Retention time	PEP	Score	Precursor Intensity
slr2067	2	770.4076	1538.801	−8.6362e−05	−0.11205	43.331	7.1289e−06	141.82	4203656

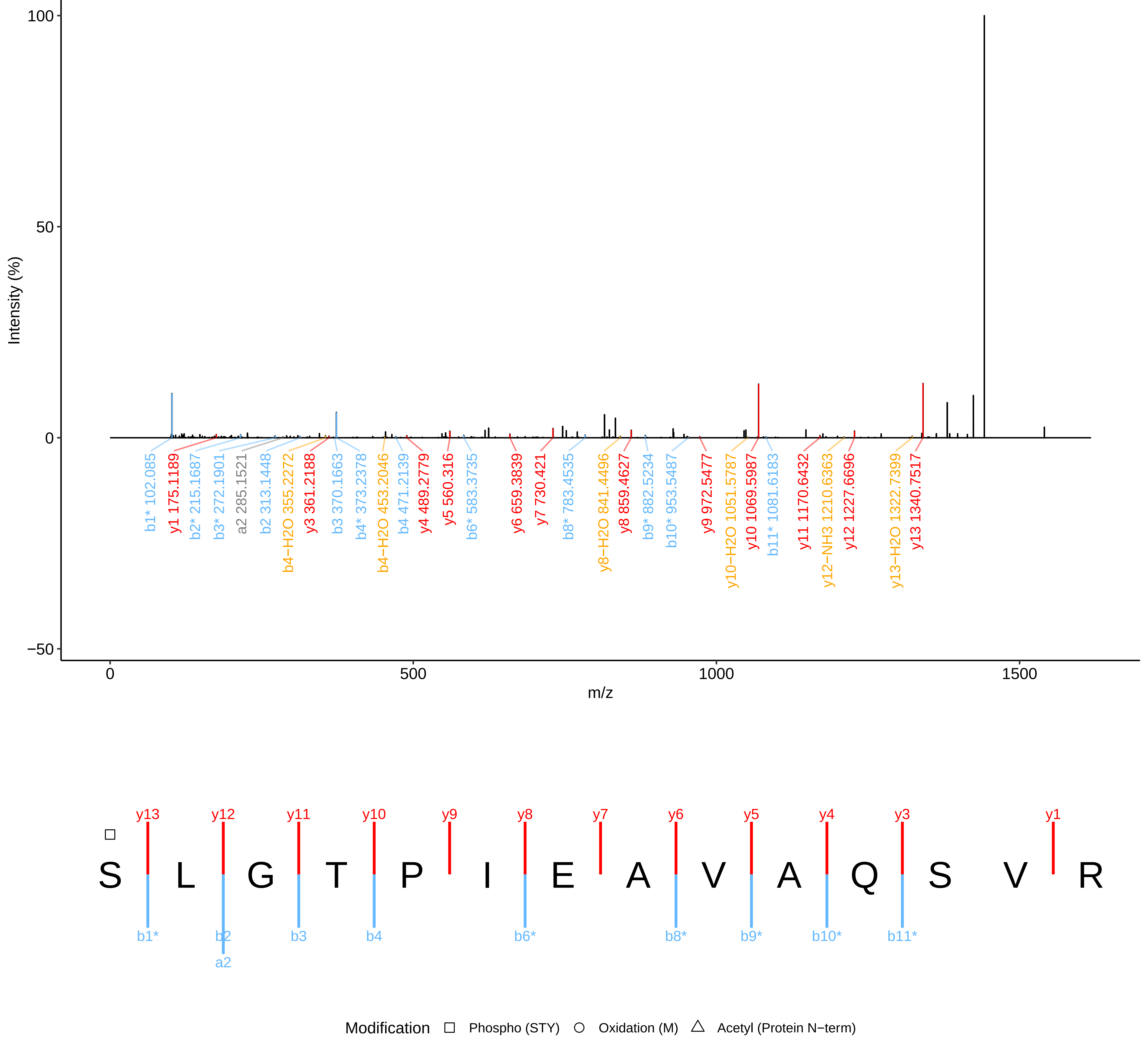

20210828_PS_SCy031_phospho_R3_TiO2_LC24h_F2 − Scan number: 22630

Gene Names	Charge	m/z	Mass	Mass error [Da]	Mass error [ppm]	Retention time	PEP	Score	Precursor Intensity
slr2067	2	770.4076	1538.801	NA	NA	31.472	0.0040575	110.78	3804075

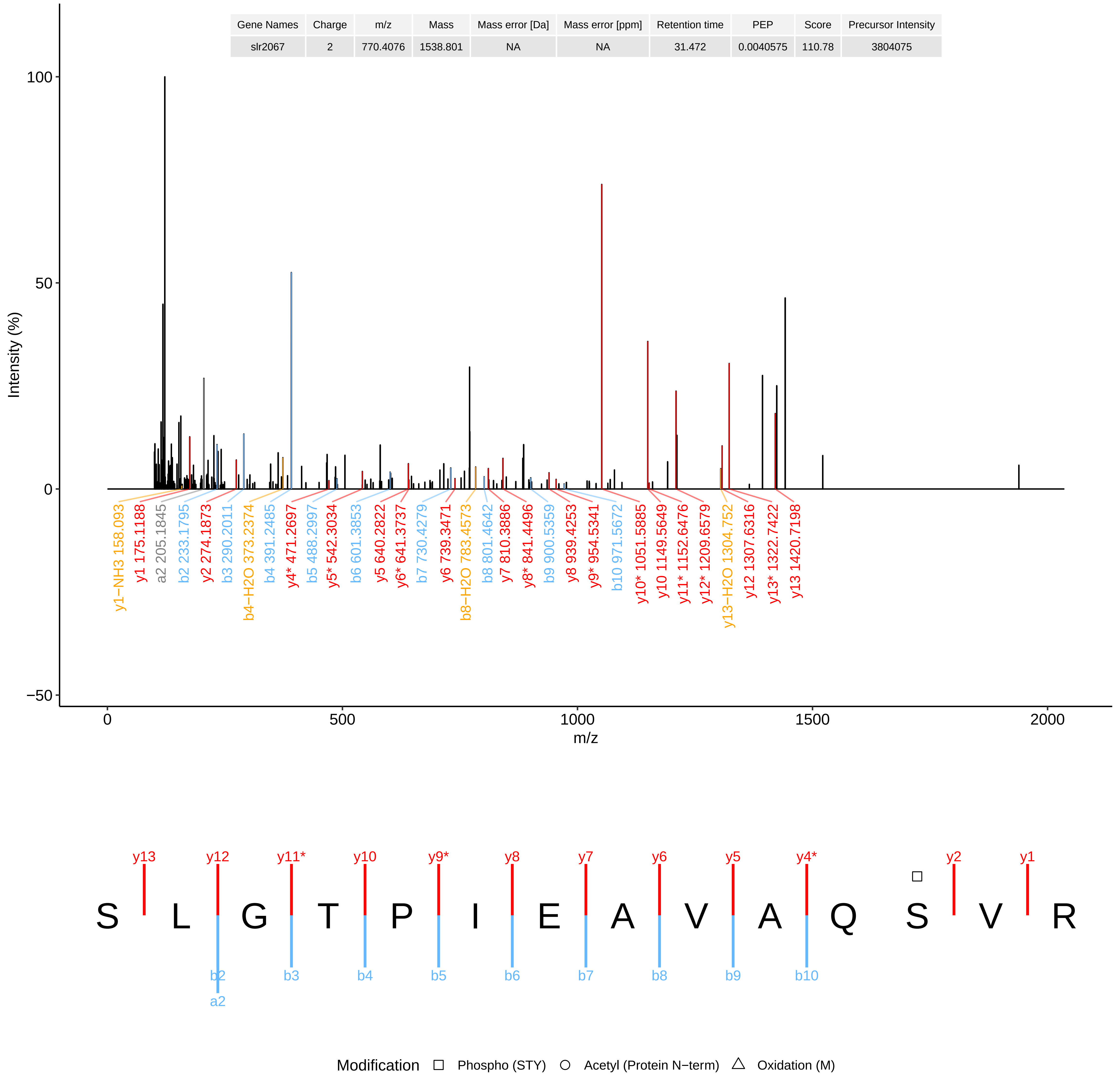

20210828_PS_SCy031_phospho_R3_TiO2_LC3h_F2 − Scan number: 18868

Gene Names	Charge	m/z	Mass	Mass error [Da]	Mass error [ppm]	Retention time	PEP	Score	Precursor Intensity
slr2073	3	719.0401	2154.098	−9.4773e−05	−0.13174	26.715	6.1462e−08	137.89	6171217

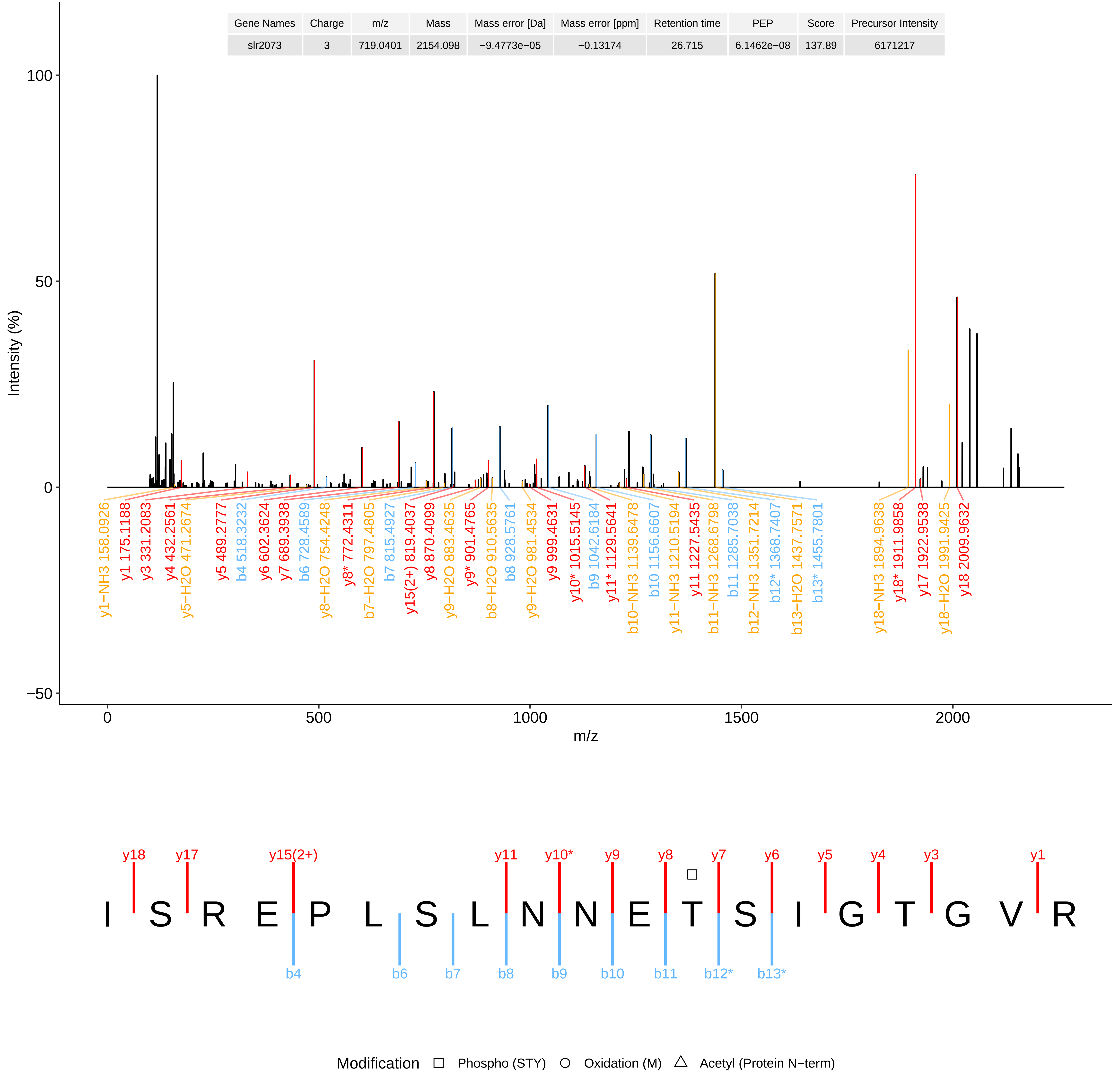

20210828_PS_SCy031_phospho_R3_TiO2_LC3h_F2 − Scan number: 24500

Gene Names	Charge	m/z	Mass	Mass error [Da]	Mass error [ppm]	Retention time	PEP	Score	Precursor Intensity
slr2073	3	966.7425	2897.206	0.00018771	0.19661	33.803	2.3834e−66	181.63	7637605

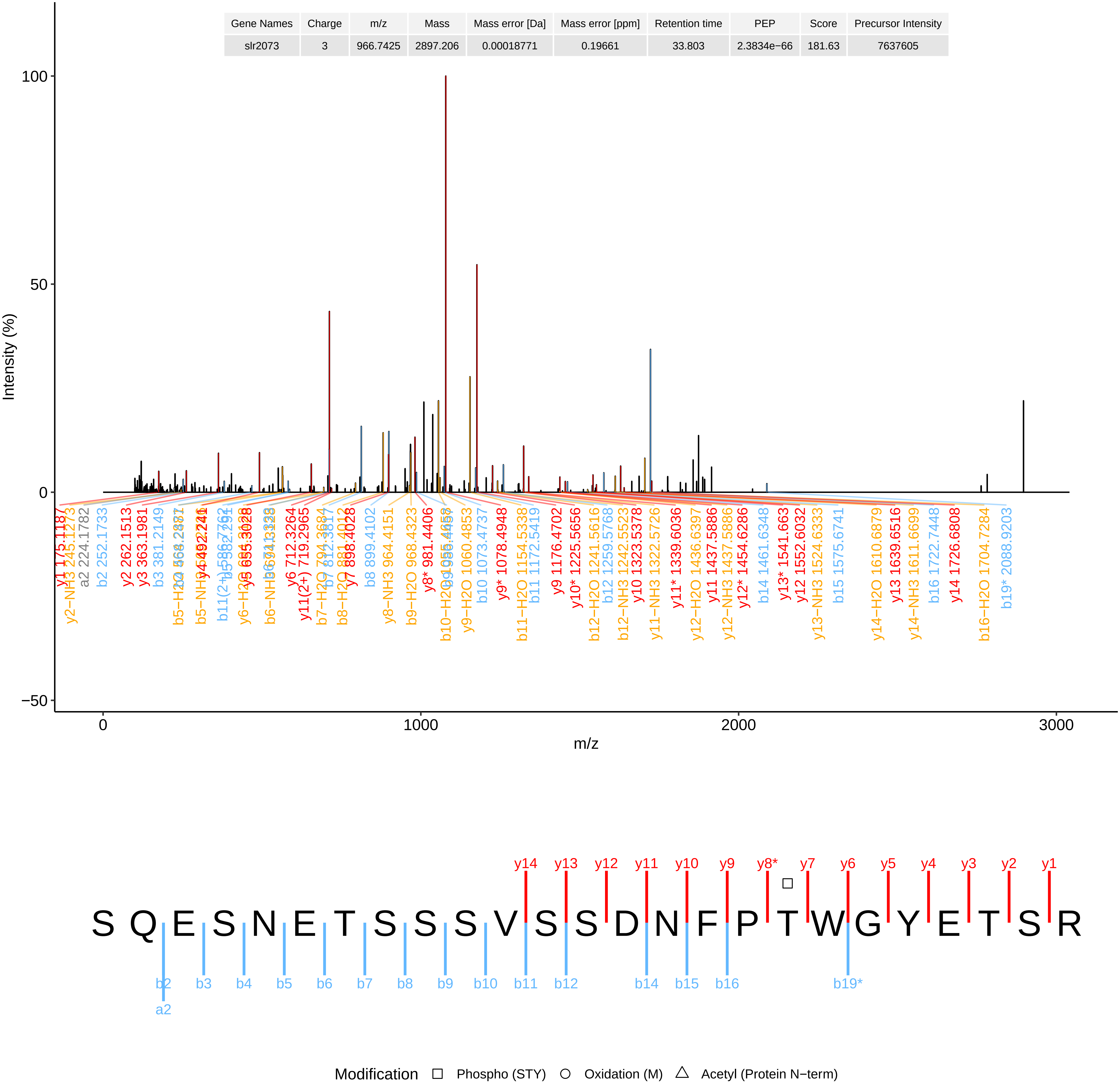

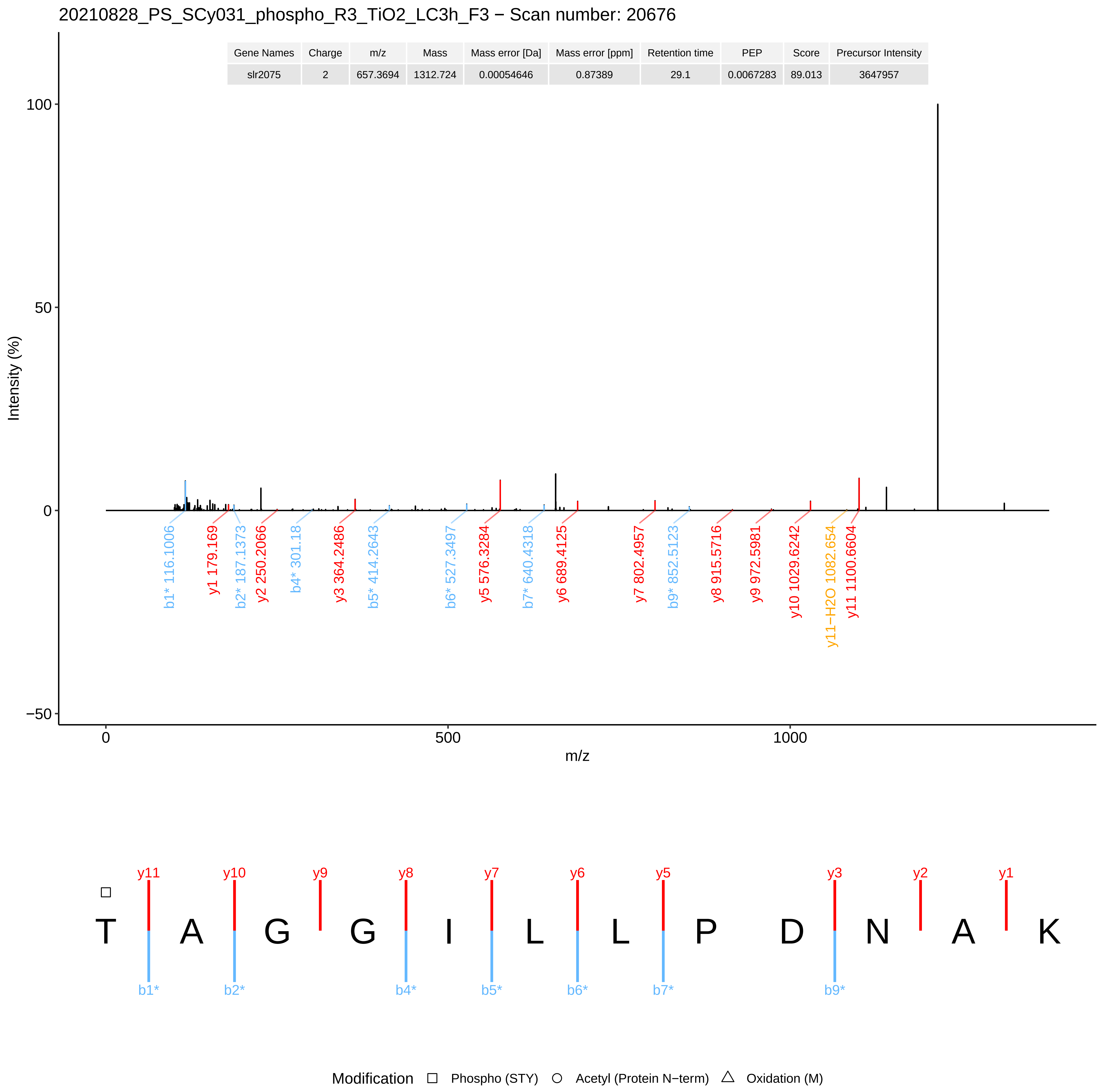
20210828_PS_SCy031_phospho_R3_TiO2_LC3h_F3 − Scan number: 20676
Gene Names | Charge | m/z | Mass | Mass error [Da] | Mass error [ppm] | Retention time | PEP | Score | Precursor Intensity
slr2075 | 2 | 657.3694 | 1312.724 | 0.00054646 | 0.87389 | 29.1 | 0.0067283 | 89.013 | 3647957
Intensity (%)
m/z
b1* 116.1006
y1 179.169
b2* 187.1373
y2 250.2066
b4* 301.18
y3 364.2486
b5* 414.2643
b6* 527.3497
y5 576.3284
b7* 640.4318
y6 689.4125
y7 802.4957
b9* 852.5123
y8 915.5716
y9 972.5981
y10 1029.6242
y11−H2O 1082.654
y11 1100.6604
T A G G I L L P D N A K
Modification
Phospho (STY)
Acetyl (Protein N−term)
Oxidation (M)

20210828_PS_SCy031_phospho_R3_TiO2_HC_F2 − Scan number: 12734

Gene Names	Charge	m/z	Mass	Mass error [Da]	Mass error [ppm]	Retention time	PEP	Score	Precursor Intensity
slr2094	2	788.8976	1575.781	NA	NA	18.983	0.004026	110.57	2702629

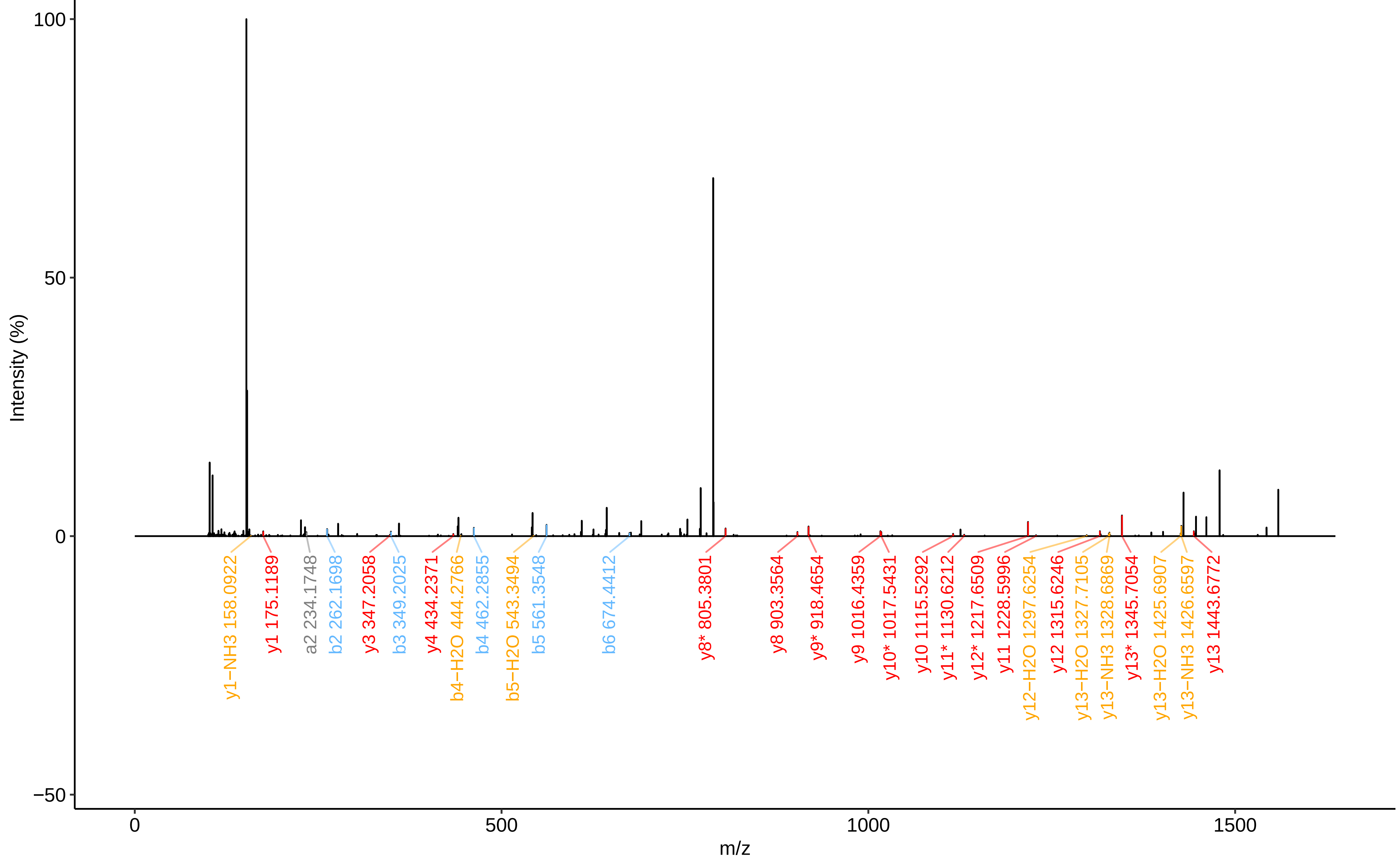

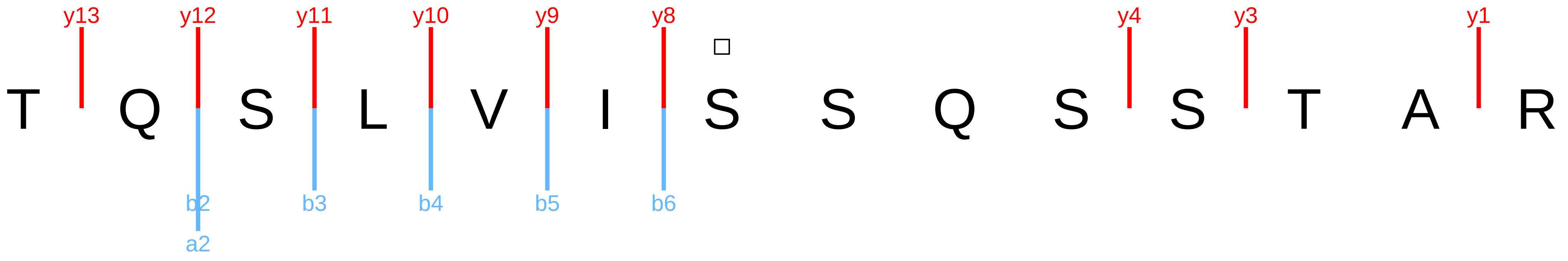

Modification □ Phospho (STY) ○ Acetyl (Protein N−term) △ Oxidation (M)

20210828_PS_SCy031_phospho_R2_TiO2_LC3h_F3 − Scan number: 17831

Gene Names	Charge	m/z	Mass	Mass error [Da]	Mass error [ppm]	Retention time	PEP	Score	Precursor Intensity
slr6039	5	800.961	3999.768	0.0014402	1.7972	25.586	3.1849e−16	100.1	3959648

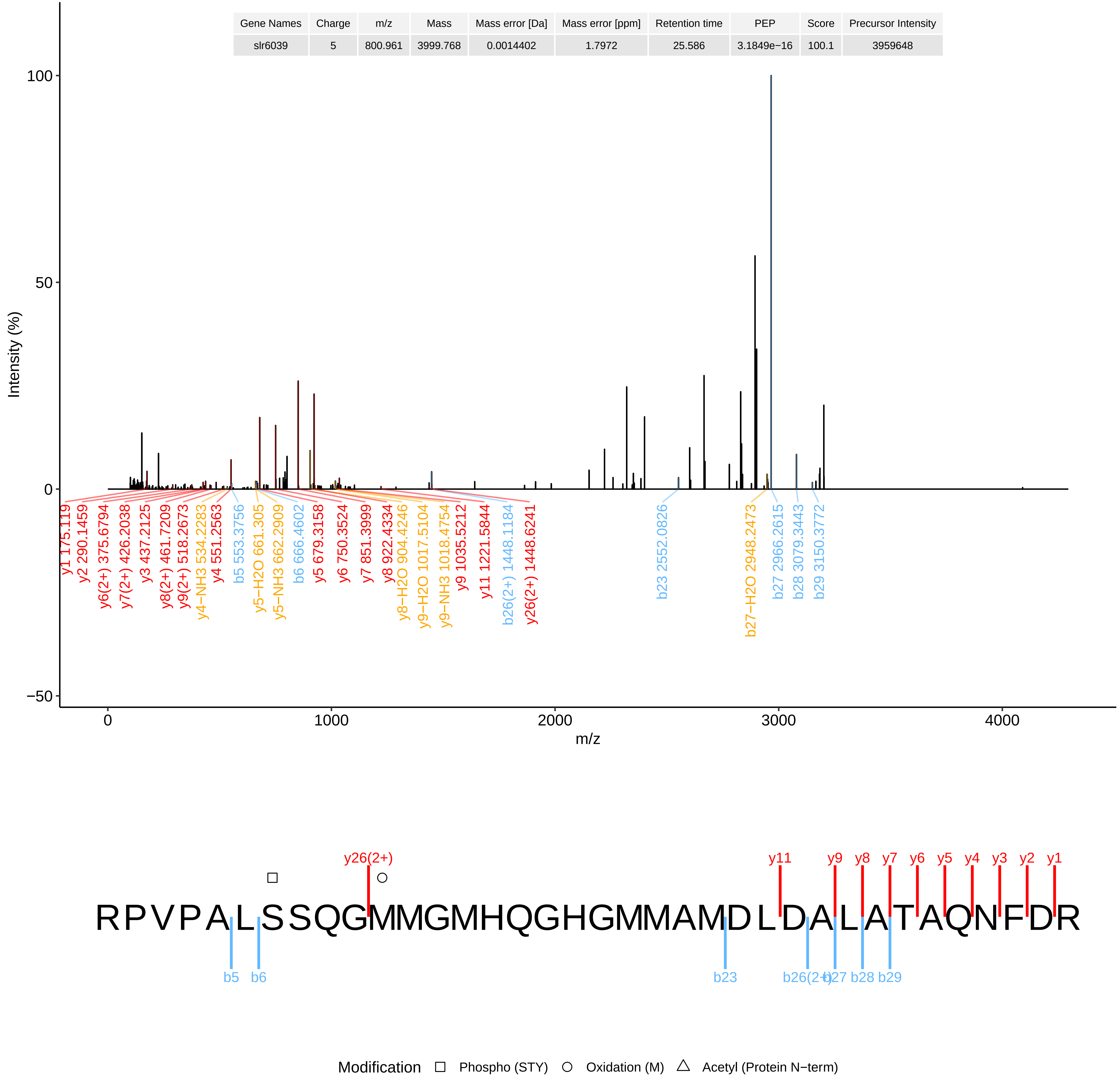

20210828_PS_SCy031_phospho_R3_TiO2_HC_F3 − Scan number: 28753

Gene Names	Charge	m/z	Mass	Mass error [Da]	Mass error [ppm]	Retention time	PEP	Score	Precursor Intensity
slr7010	2	887.9225	1773.83	16.027	18726	39.322	0.0052758	46.378	7703590

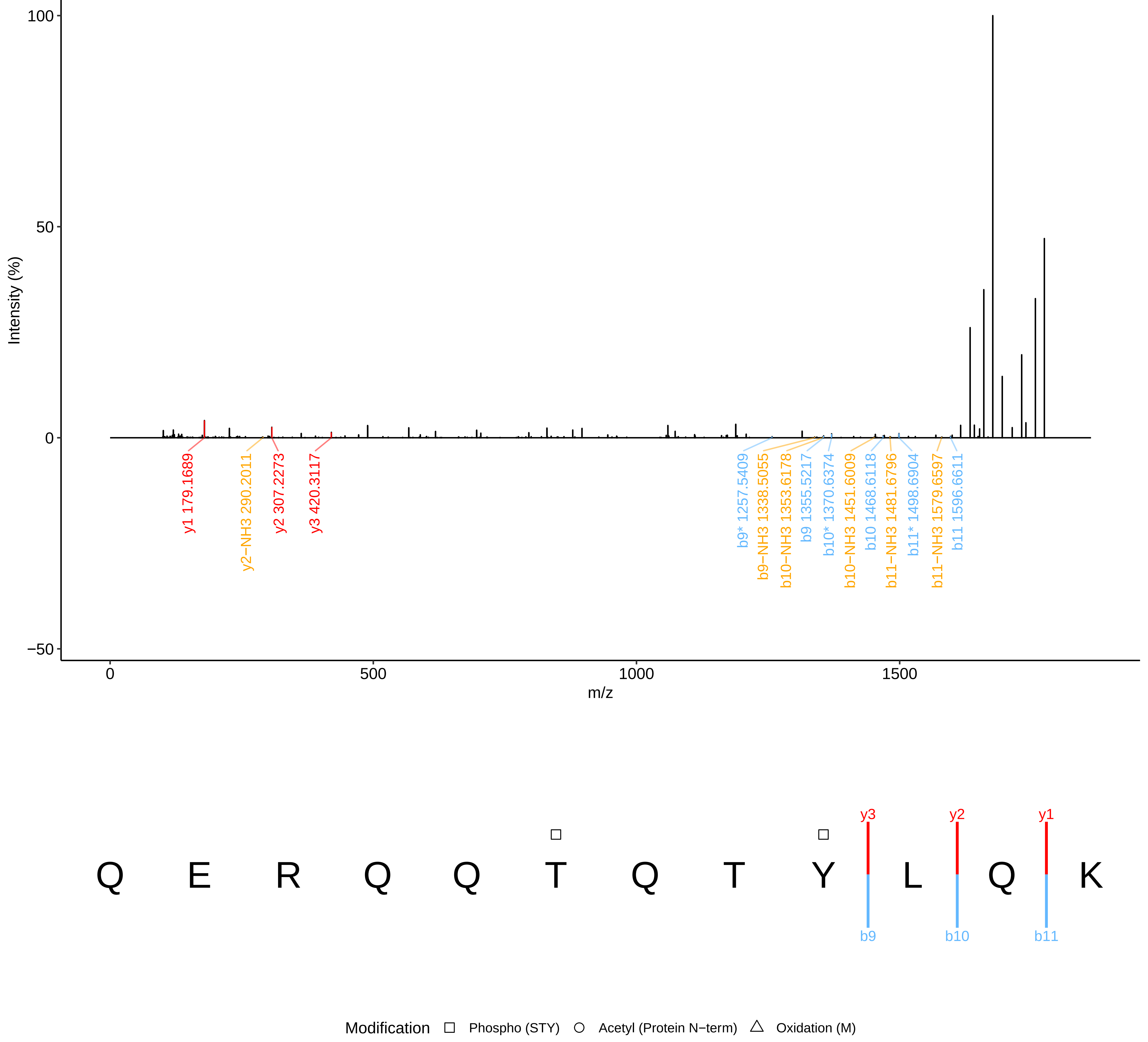

20210823_PS_SCy031_R1_phospho_LC24h_TiO2_2 − Scan number: 12527

Gene Names	Charge	m/z	Mass	Mass error [Da]	Mass error [ppm]	Retention time	PEP	Score	Precursor Intensity
ssl0563	2	563.2903	1124.566	NA	NA	18.712	0.053025	83.862	7164698

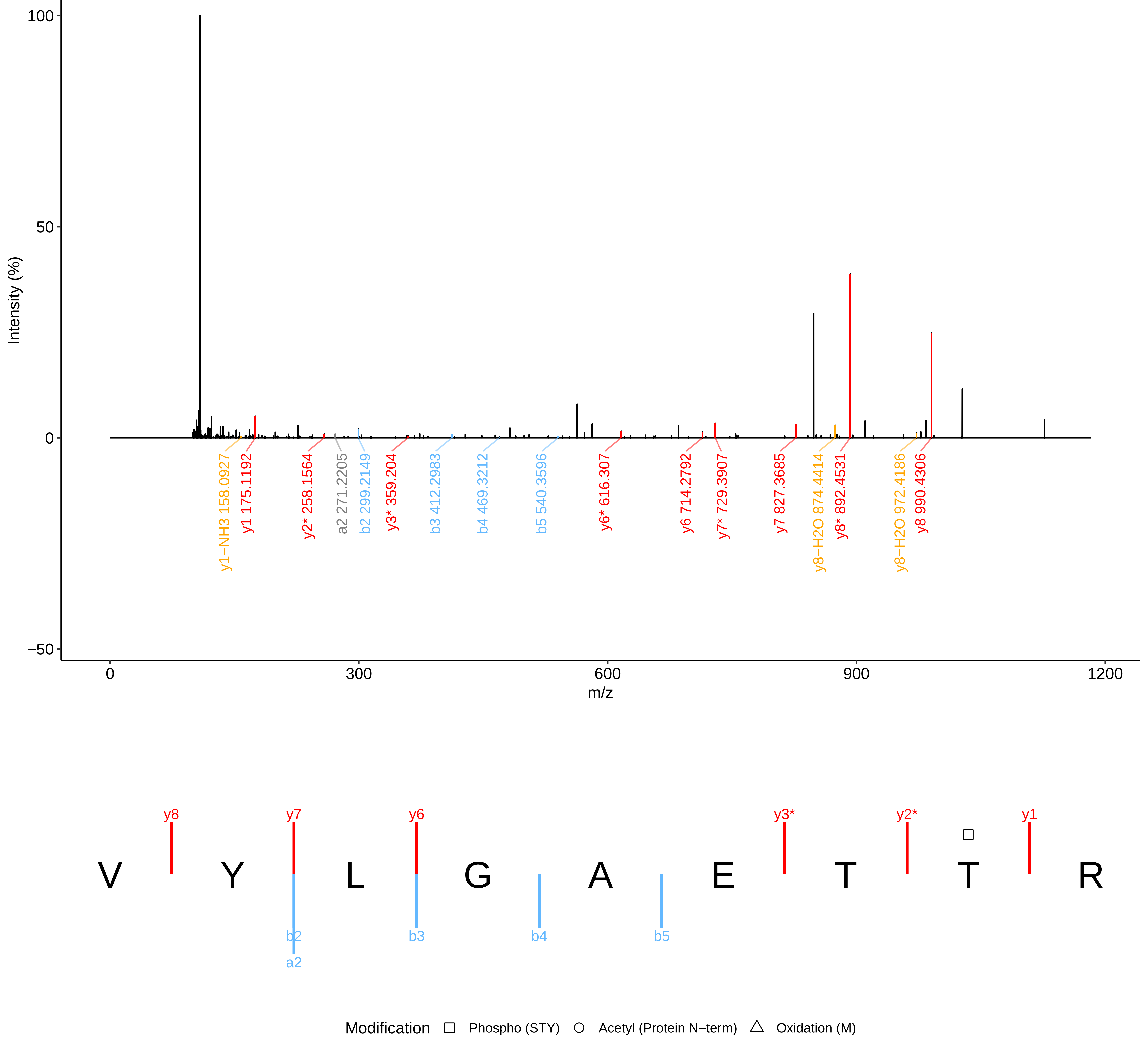

20211217_PS_SCy031_Proteome_R3_HC_F4 − Scan number: 15393

Gene Names	Charge	m/z	Mass	Mass error [Da]	Mass error [ppm]	Retention time	PEP	Score	Precursor Intensity
ssl0563	3	842.3926	2524.156	NA	NA	25.366	4.897e−08	105.39	11554511

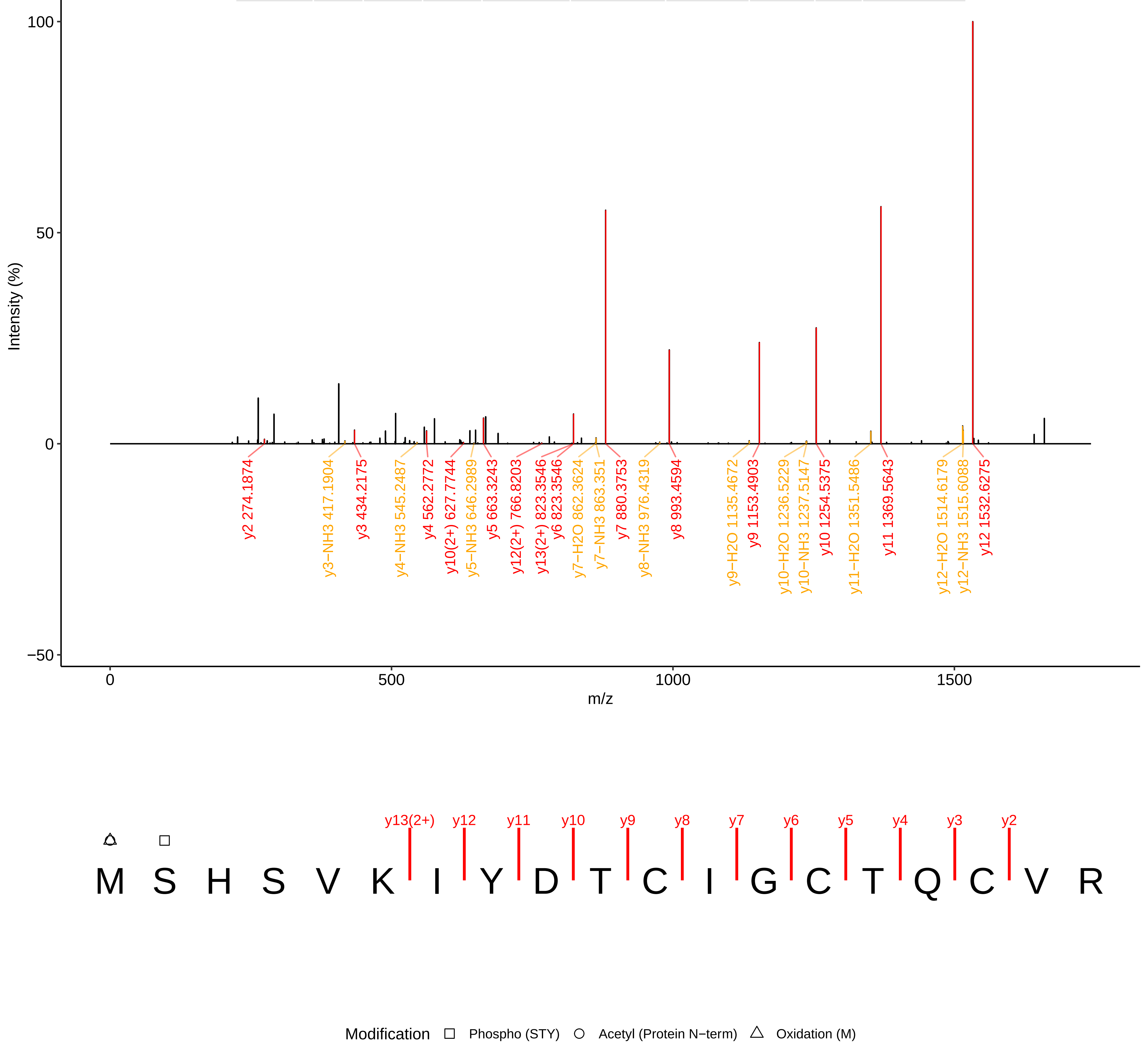

20210823_PS_SCy031_R1_phospho_LC3h_TiO2_3 − Scan number: 20146

Gene Names	Charge	m/z	Mass	Mass error [Da]	Mass error [ppm]	Retention time	PEP	Score	Precursor Intensity
ssl0601	3	668.6806	2003.02	7.5267e−05	0.11416	28.337	6.7778e−06	83.064	7961935

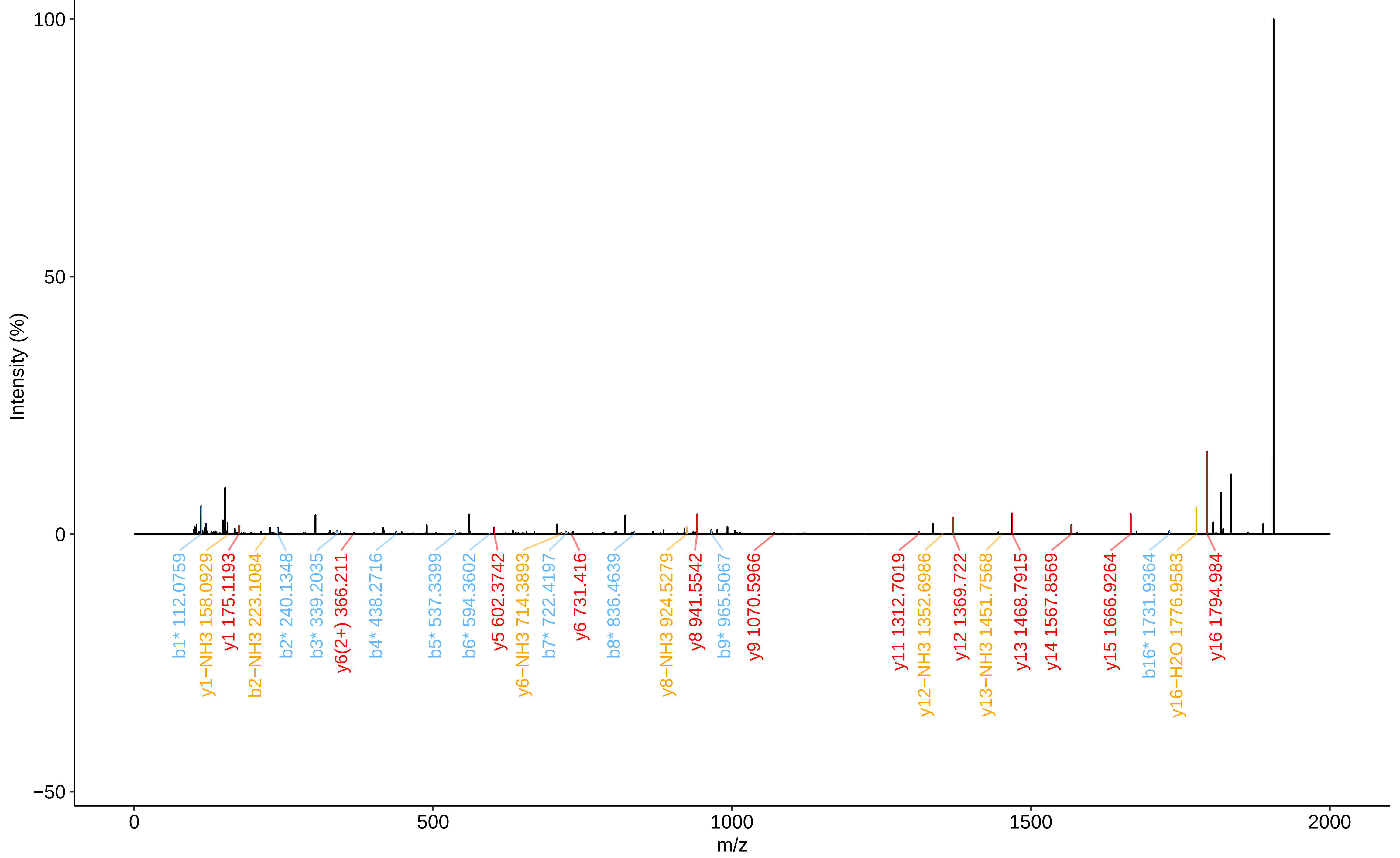

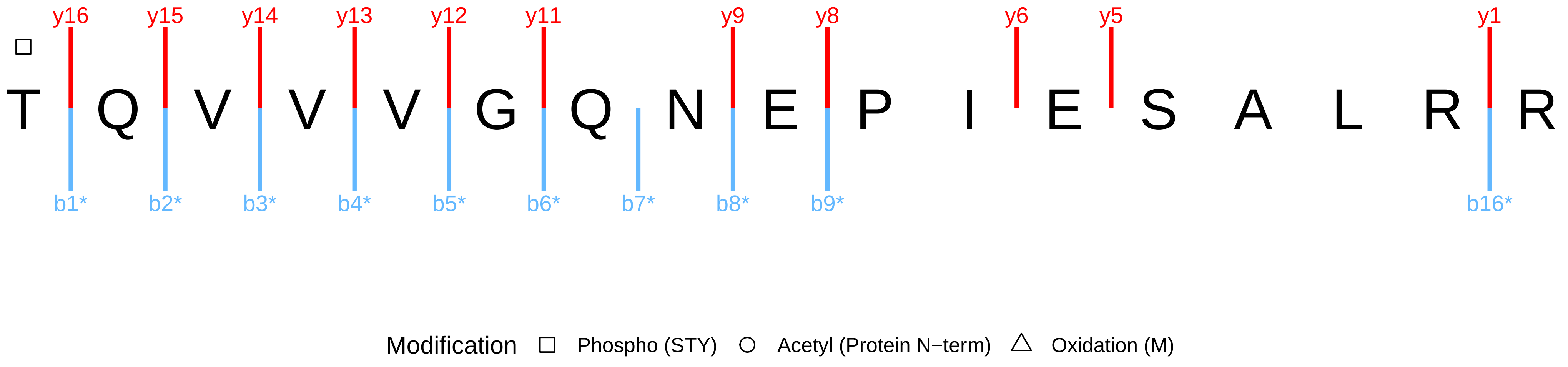

20210828_PS_SCy031_phospho_R3_TiO2_HC_F3 − Scan number: 25697

Gene Names	Charge	m/z	Mass	Mass error [Da]	Mass error [ppm]	Retention time	PEP	Score	Precursor Intensity
ssl0707	2	718.8391	1435.664	3.3632e−05	0.046787	35.452	3.8313e−26	201	4199602

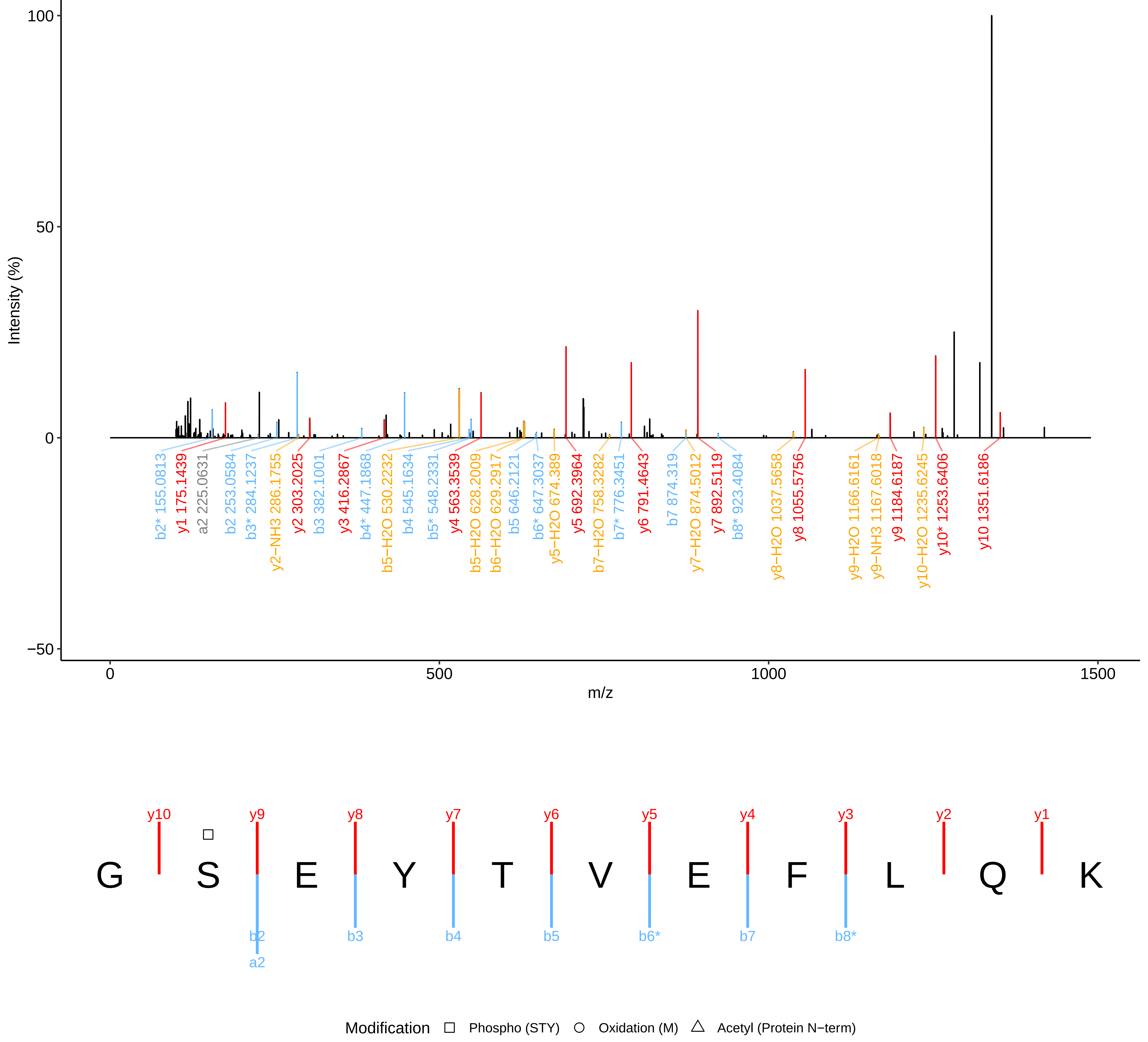

20210828_PS_SCy031_phospho_R3_TiO2_LC24h_F2 − Scan number: 18004

Gene Names	Charge	m/z	Mass	Mass error [Da]	Mass error [ppm]	Retention time	PEP	Score	Precursor Intensity
ssl3093	2	1014.984	2027.953	−7.1056e−05	−0.070007	25.651	1.3315e−23	179.09	5050195

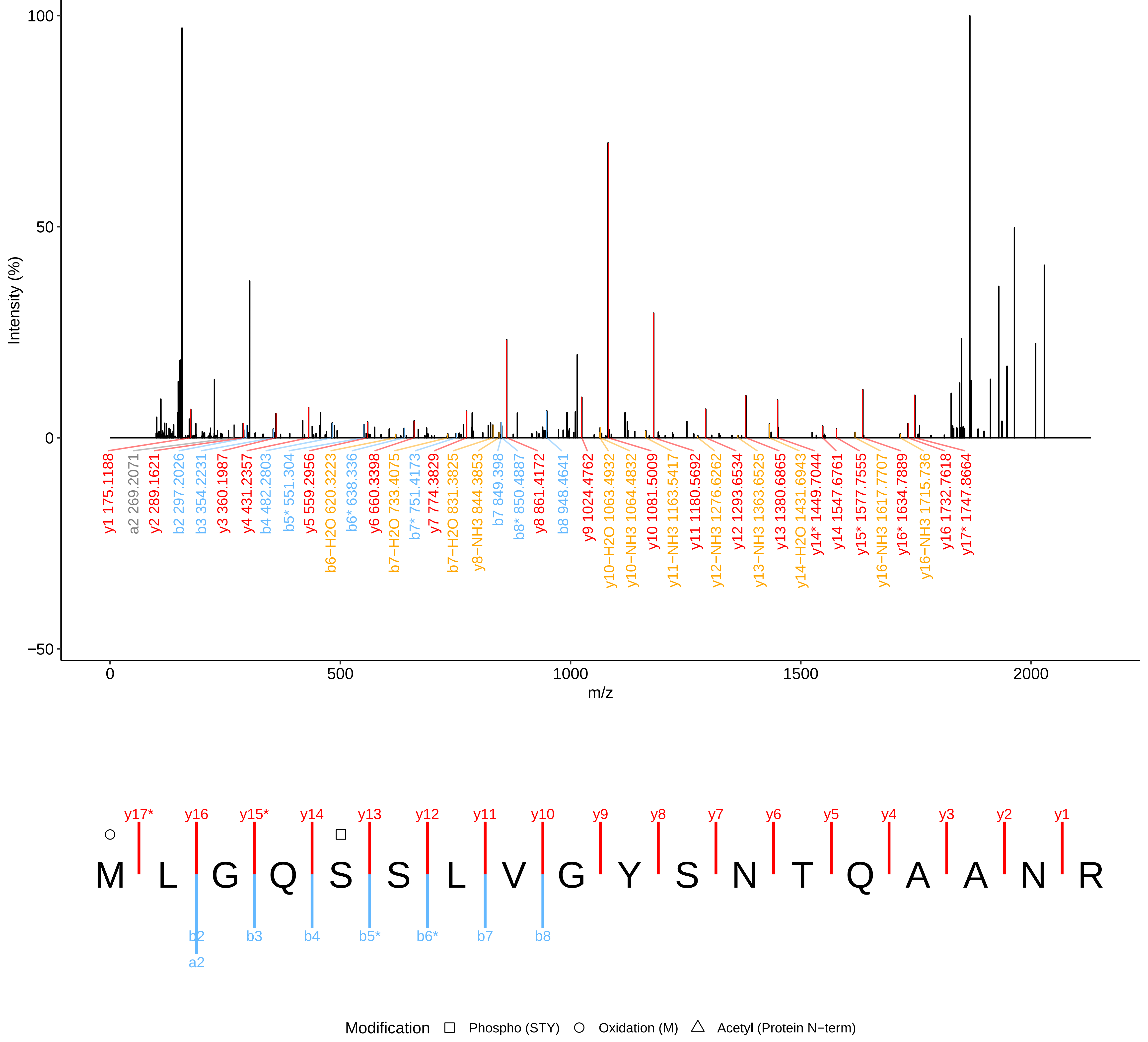

20210828_PS_SCy031_phospho_R3_TiO2_HC_F2 − Scan number: 16557

Gene Names	Charge	m/z	Mass	Mass error [Da]	Mass error [ppm]	Retention time	PEP	Score	Precursor Intensity
ssl3093	2	1014.984	2027.953	NA	NA	23.788	0.0029441	69.111	NA

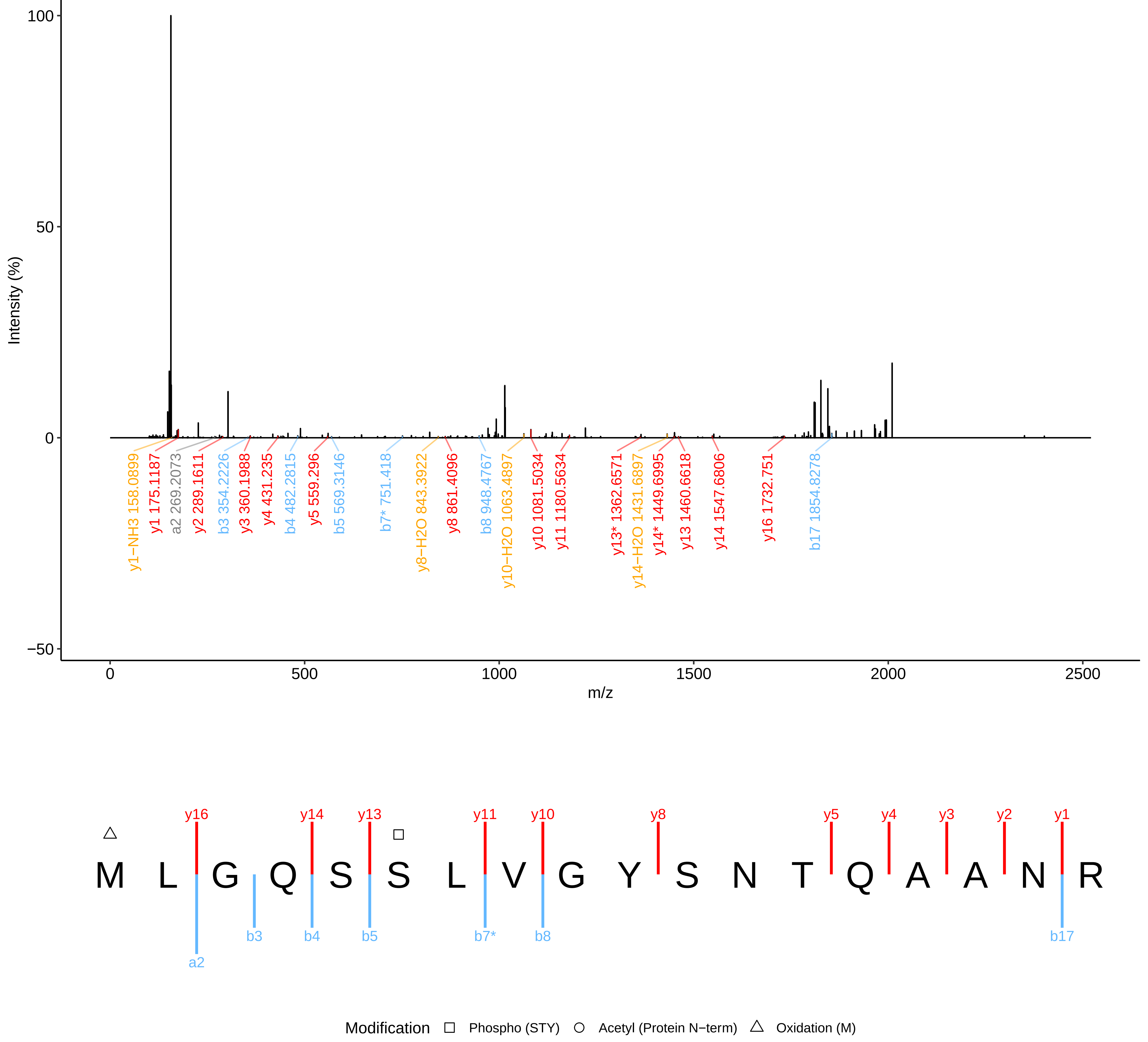

20210828_PS_SCy031_phospho_R3_TiO2_HC_F2 − Scan number: 16739

Gene Names	Charge	m/z	Mass	Mass error [Da]	Mass error [ppm]	Retention time	PEP	Score	Precursor Intensity
ssl3093	2	1014.984	2027.953	−0.00053353	−0.52539	24.016	6.9342e−05	117.27	3255863

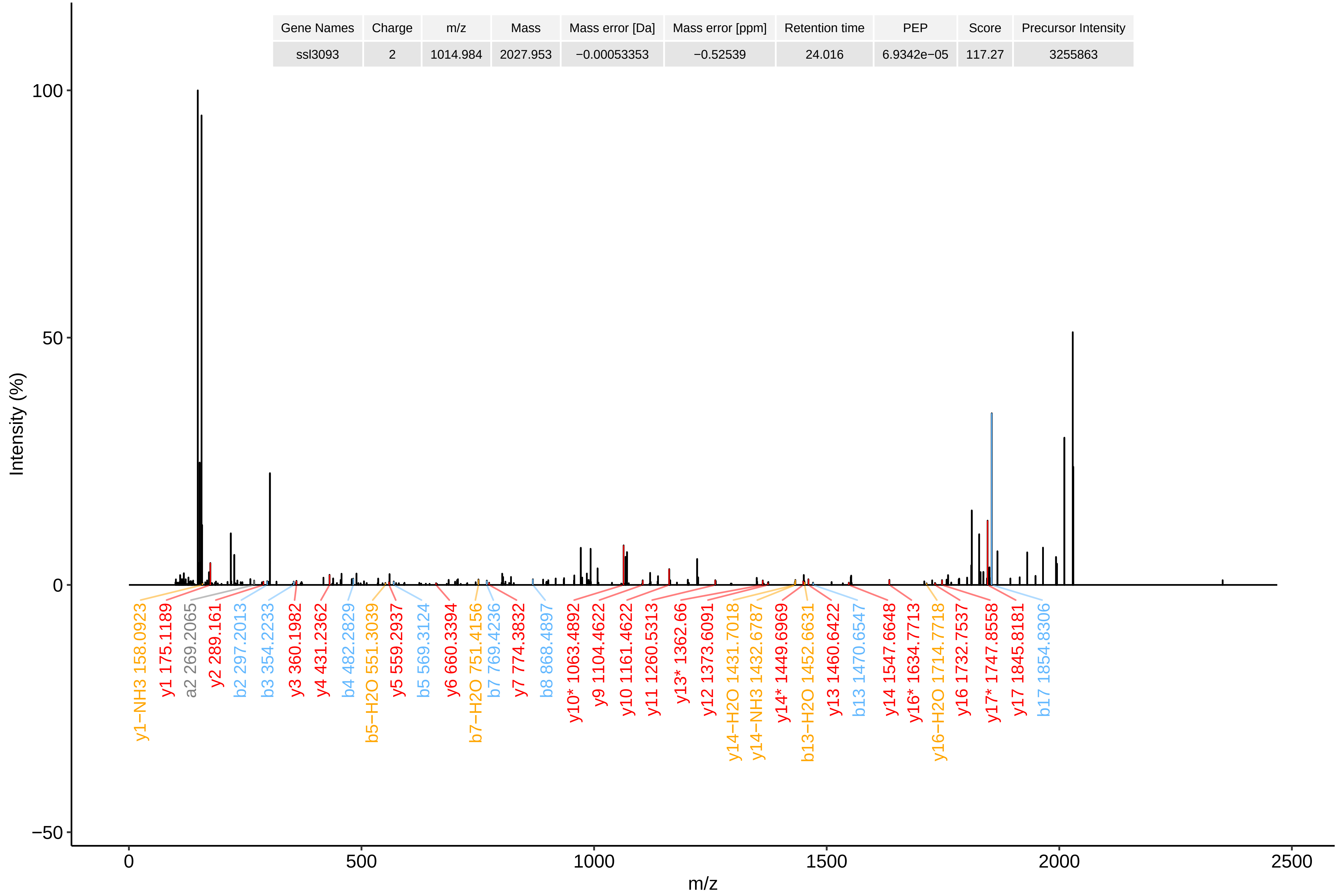

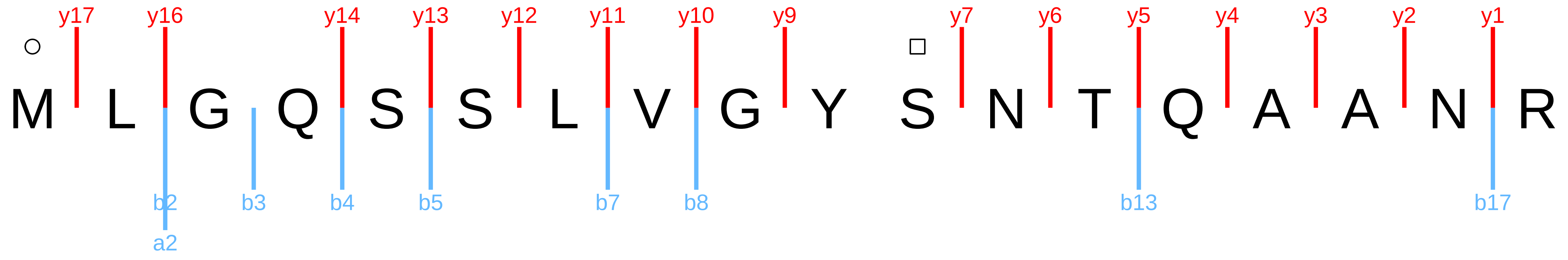

Modification □ Phospho (STY) ○ Oxidation (M) △ Acetyl (Protein N−term)

20210828_PS_SCy031_phospho_R3_TiO2_HC_F1 − Scan number: 24021

Gene Names	Charge	m/z	Mass	Mass error [Da]	Mass error [ppm]	Retention time	PEP	Score	Precursor Intensity
ssl3093	2	1101.03	2200.046	NA	NA	33.149	1.346e−20	173.56	1790700

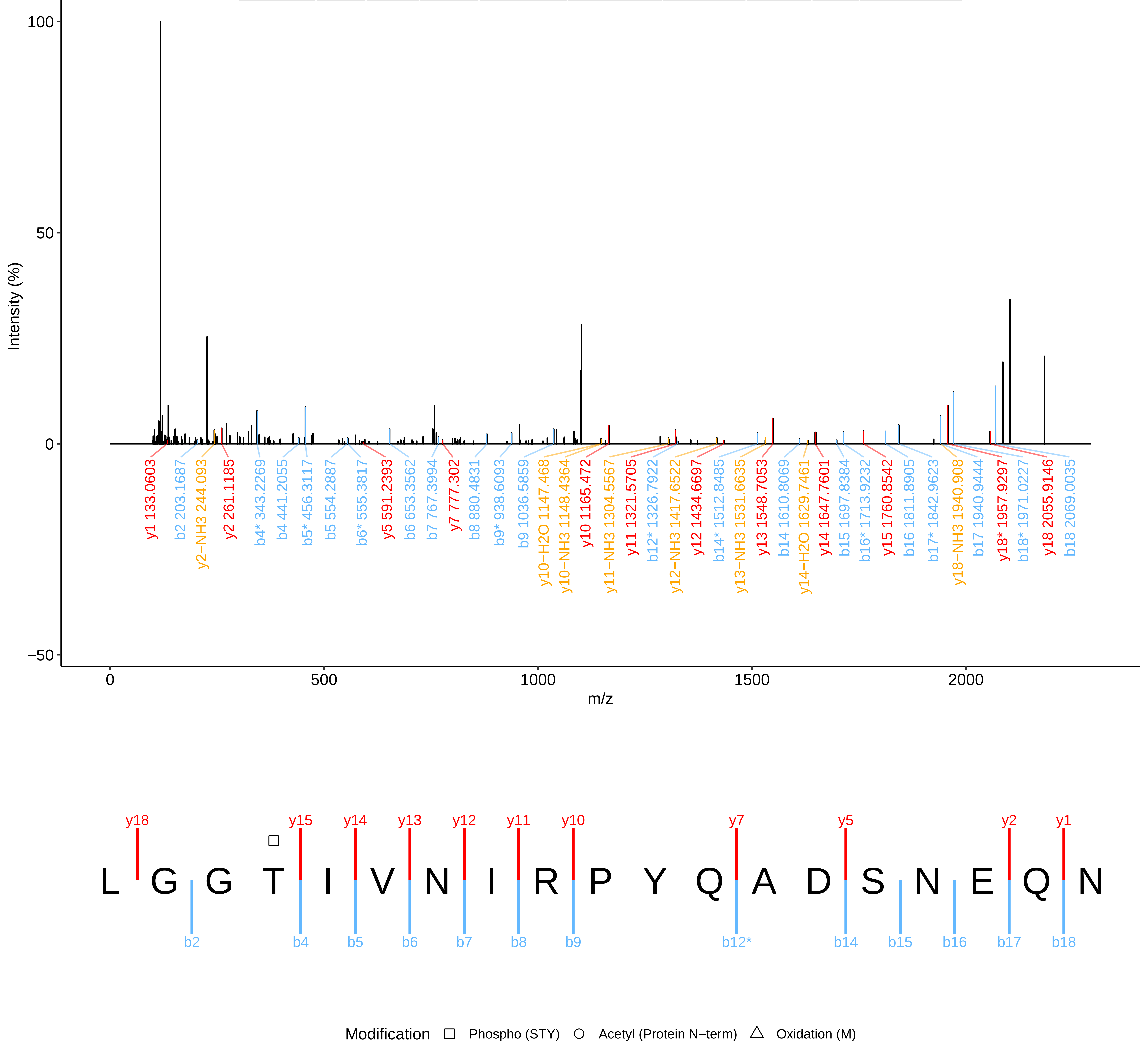

20210828_PS_SCy031_phospho_R3_TiO2_LC3h_F3 − Scan number: 4362

Gene Names	Charge	m/z	Mass	Mass error [Da]	Mass error [ppm]	Retention time	PEP	Score	Precursor Intensity
ssl3364	3	485.5879	1453.742	0.00015661	0.33735	8.5858	2.0835e−08	145.43	9152975

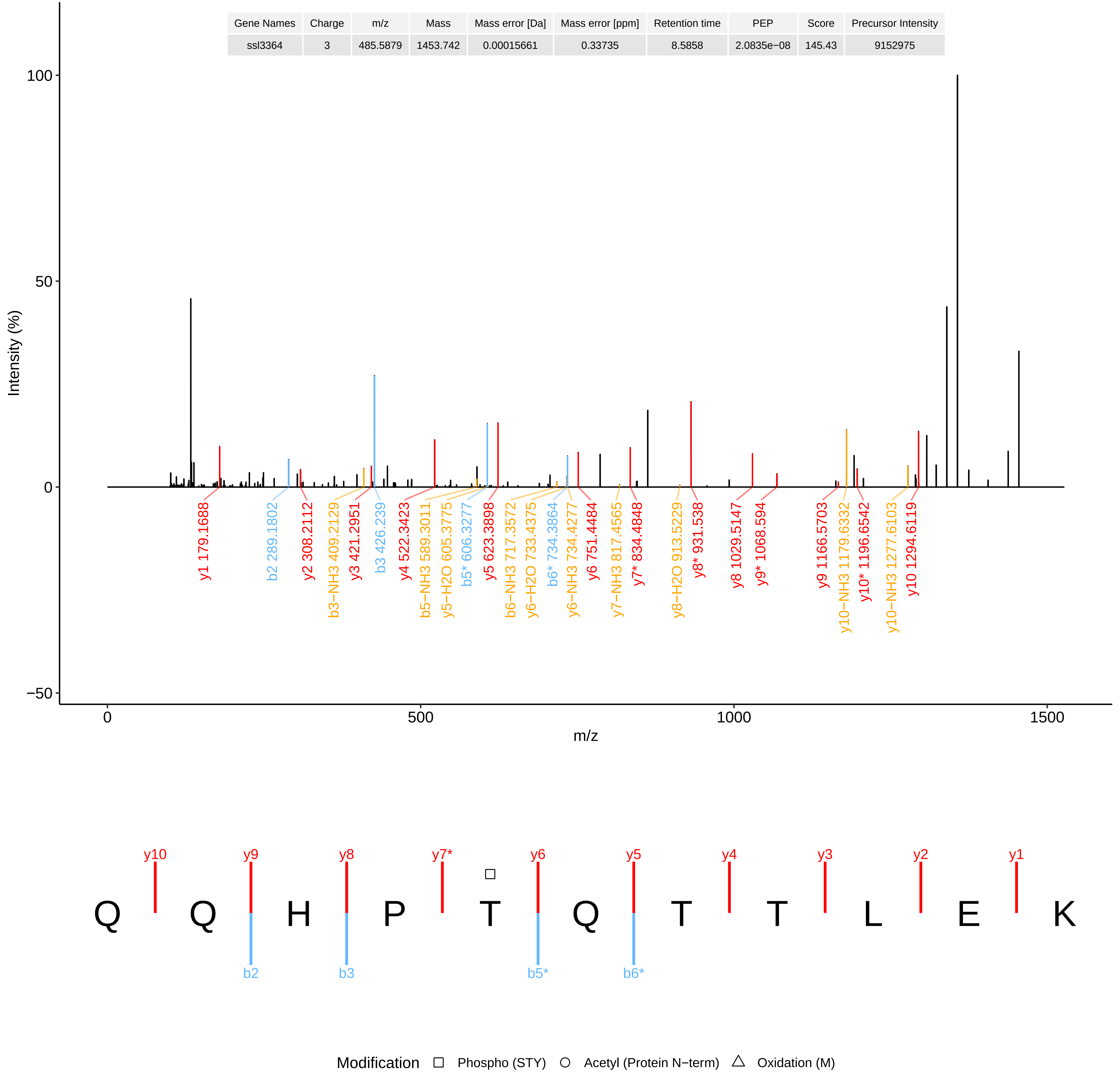

20210828_PS_SCy031_phospho_R3_TiO2_HC_F3 − Scan number: 4259

Gene Names	Charge	m/z	Mass	Mass error [Da]	Mass error [ppm]	Retention time	PEP	Score	Precursor Intensity
ssl3364	3	482.9045	1445.692	0.00022741	0.48988	8.4792	9.1456e−05	109.1	6812958

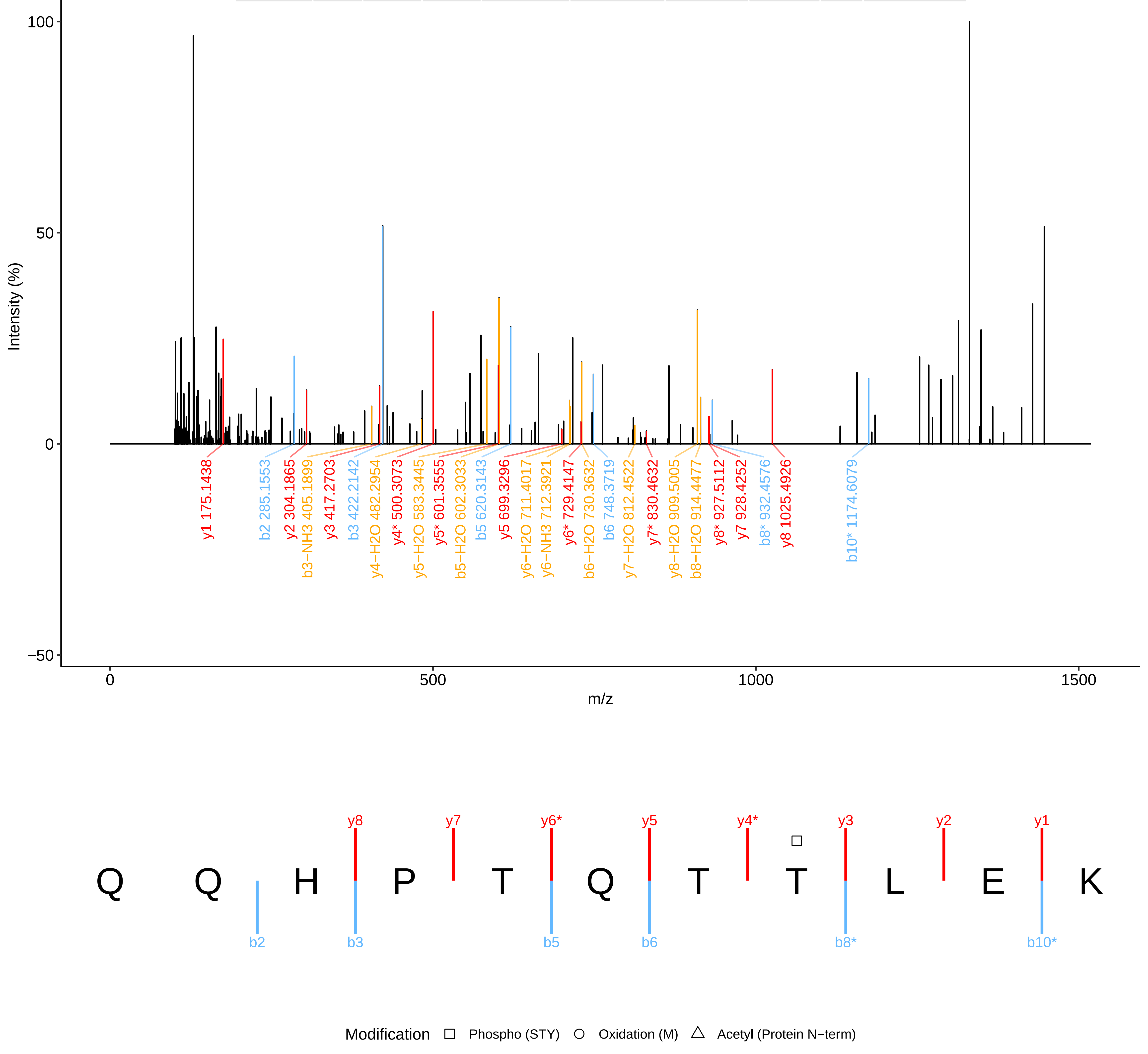

20211212_PS_SCy031_proteome_R1_3hLC_F2 − Scan number: 4729

Gene Names	Charge	m/z	Mass	Mass error [Da]	Mass error [ppm]	Retention time	PEP	Score	Precursor Intensity
ssr0680	2	749.8364	1497.658	NA	NA	10.092	0.0068851	59.198	NA

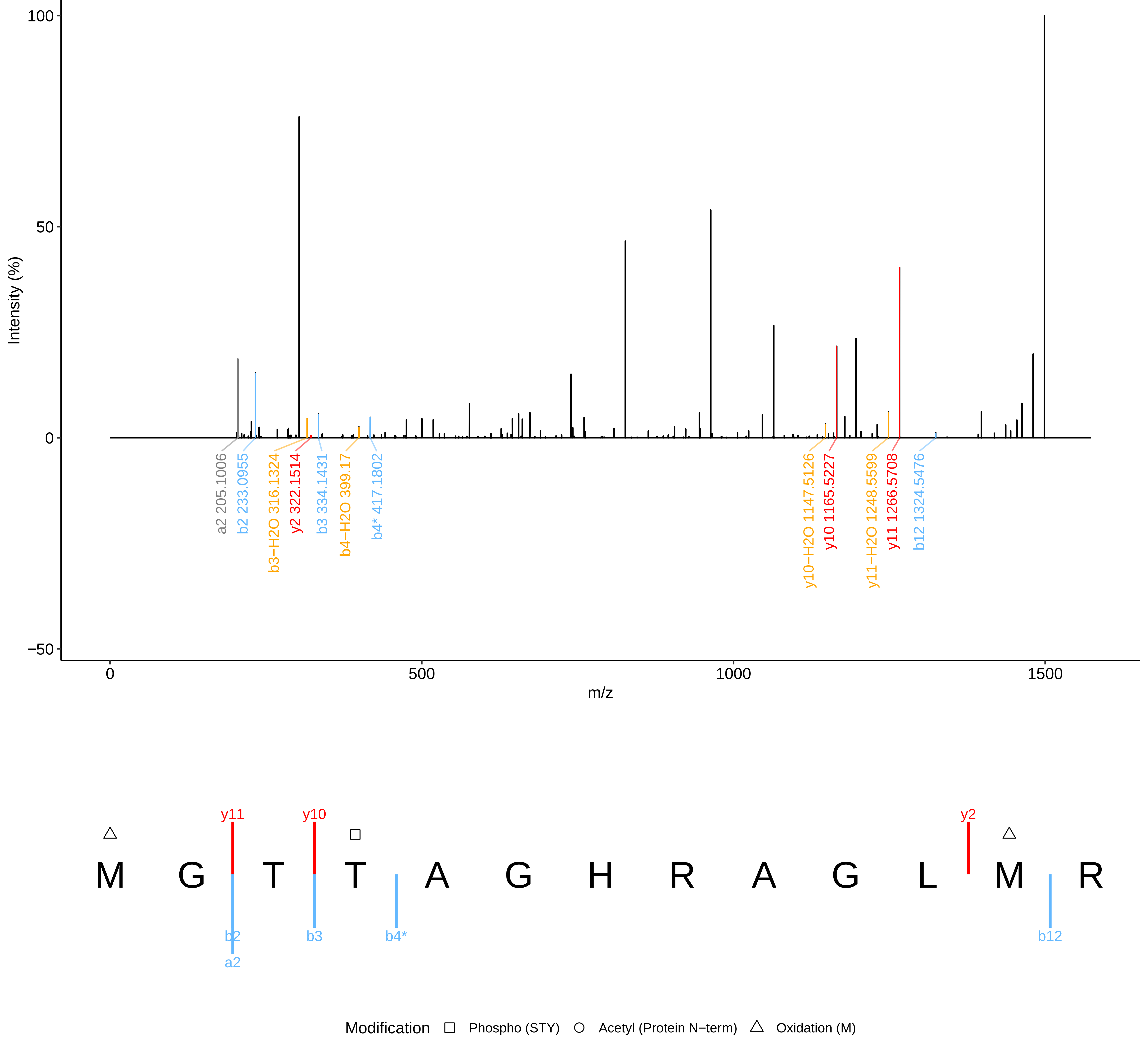

20210828_PS_SCy031_phospho_R3_TiO2_LC24h_F2 − Scan number: 3536

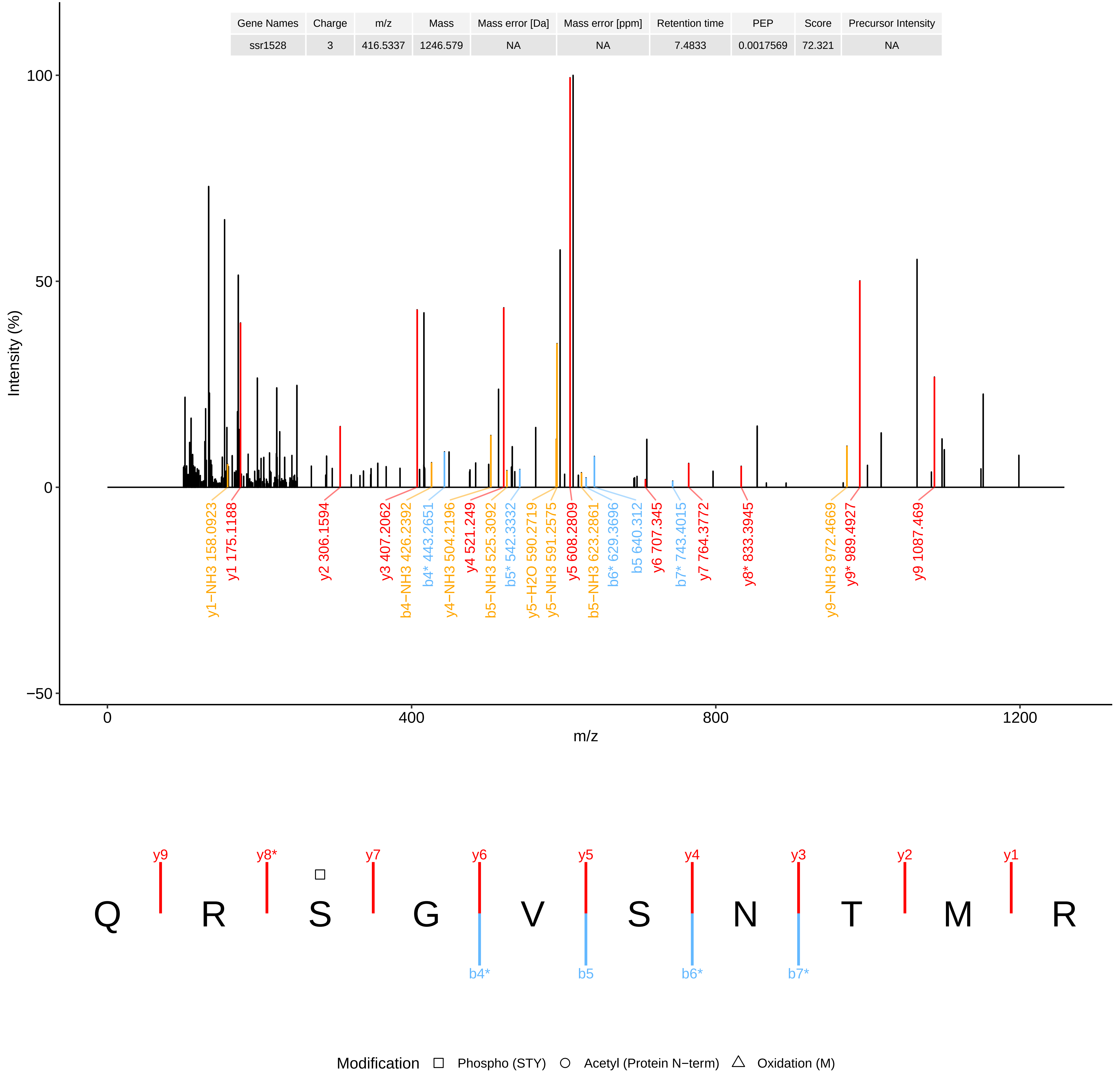

Gene Names	Charge	m/z	Mass	Mass error [Da]	Mass error [ppm]	Retention time	PEP	Score	Precursor Intensity
ssr1528	3	416.5337	1246.579	NA	NA	7.4833	0.0017569	72.321	NA

20210828_PS_SCy031_phospho_R2_TiO2_LC24h_F2 − Scan number: 30742

Gene Names	Charge	m/z	Mass	Mass error [Da]	Mass error [ppm]	Retention time	PEP	Score	Precursor Intensity
ssr2831	2	614.8538	1227.693	0.00042469	0.70921	41.681	0.00036162	83.862	2904451

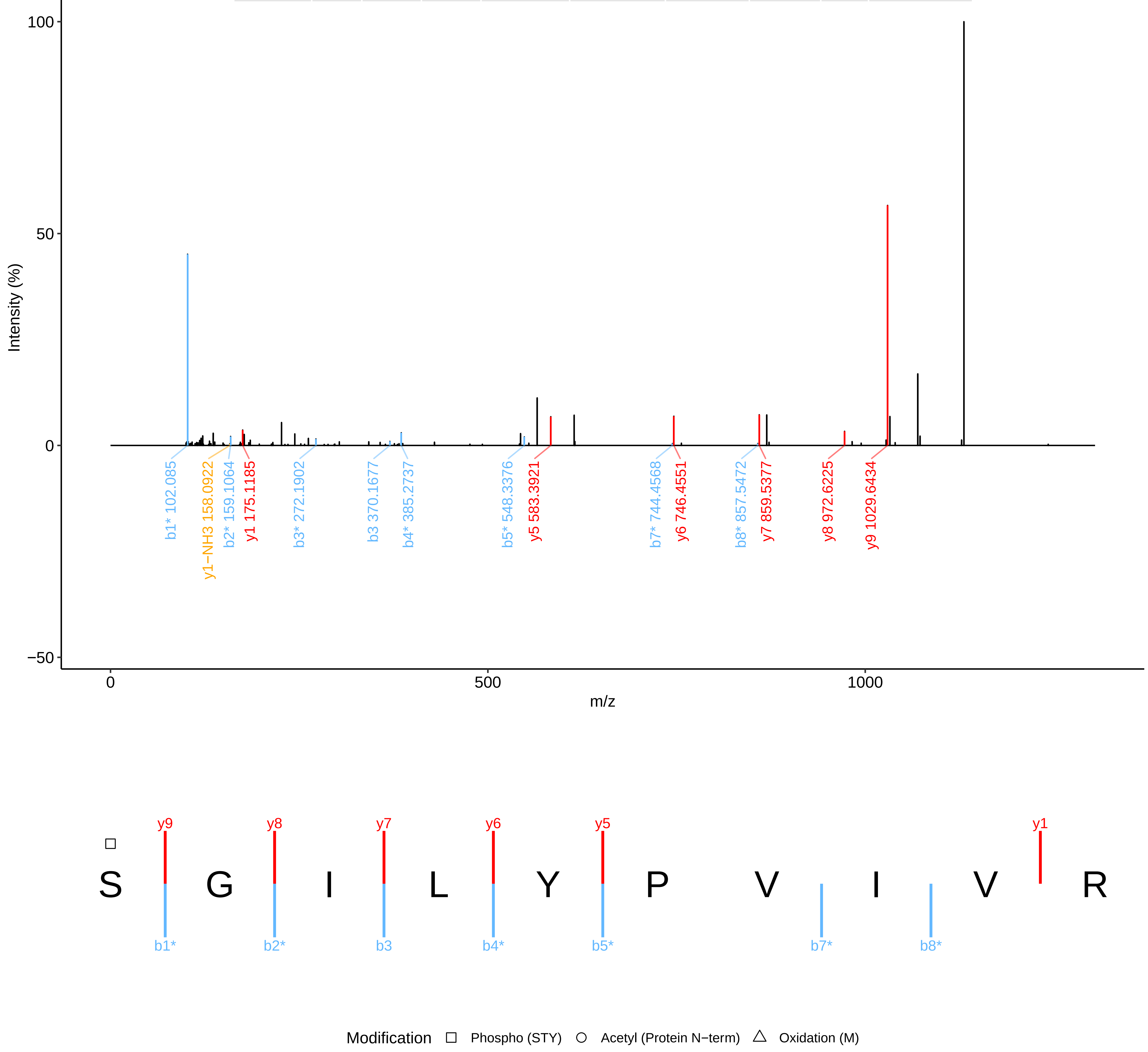

20210828_PS_SCy031_phospho_R2_TiO2_LC3h_F2 − Scan number: 37152

Gene Names	Charge	m/z	Mass	Mass error [Da]	Mass error [ppm]	Retention time	PEP	Score	Precursor Intensity
ssr2831	3	1120.539	3358.596	NA	NA	49.705	0.0046728	54.53	NA

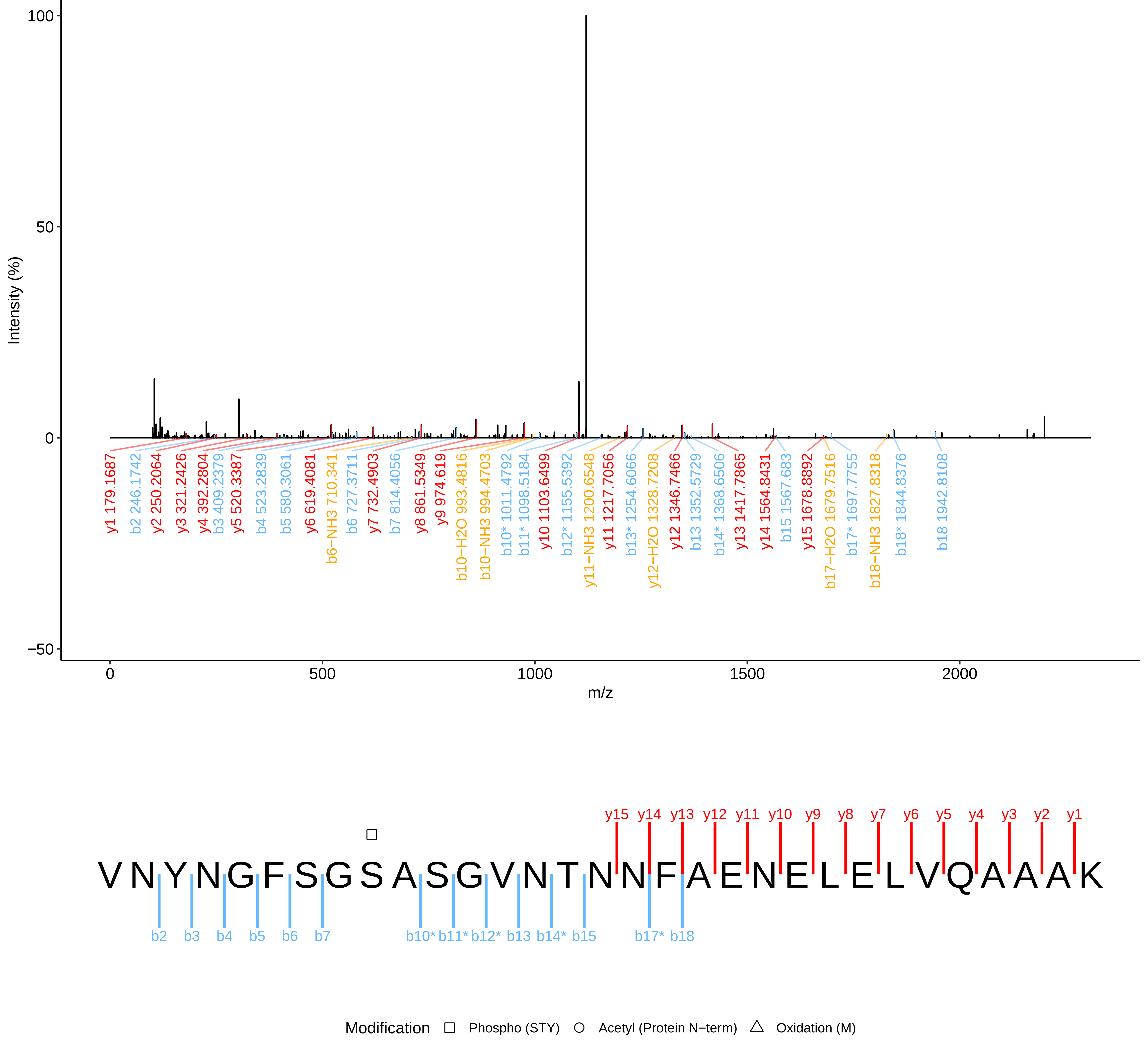

20210828_PS_SCy031_phospho_R3_TiO2_LC3h_F1 − Scan number: 5838

Gene Names	Charge	m/z	Mass	Mass error [Da]	Mass error [ppm]	Retention time	PEP	Score	Precursor Intensity
ssr3189	2	860.3414	1718.668	−0.00037242	−0.43262	10.251	4.9891e−09	151.59	5687201

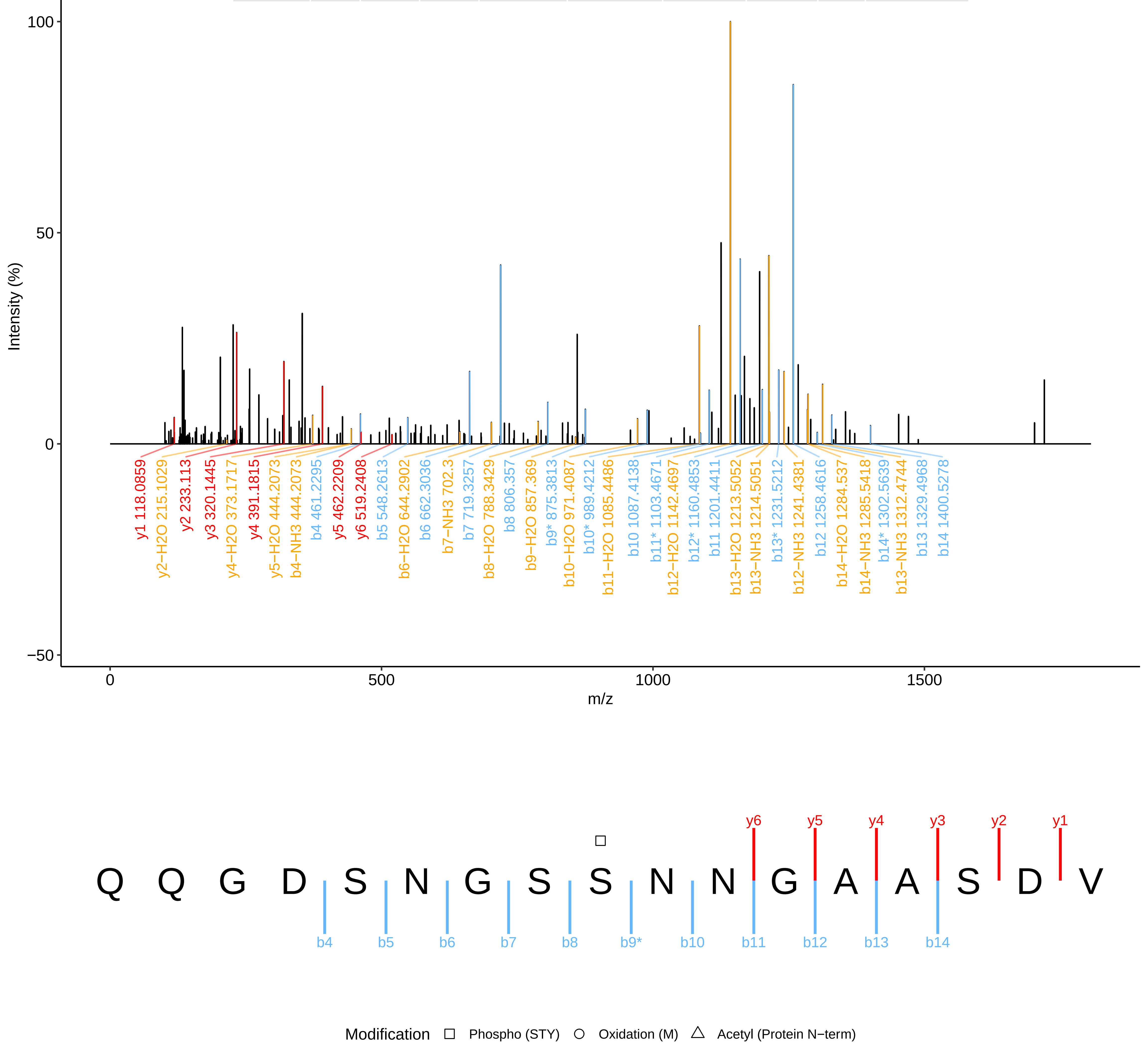

Supplement: Supplemental spectra of phosphopeptides [file mmc3.pdf]
