# Supplementary material for: The Role of Serine/Threonine-Specific Protein Kinases in Cyanobacteria - SpkB Is Involved in Acclimation to Fluctuating Conditions in Synechocystis sp. PCC 6803
Source: Mol Cell Proteomics. 2023 Oct 4;22(11):100656. doi: 10.1016/j.mcpro.2023.100656 (PMC10651672; doi:10.1016/j.mcpro.2023.100656)

Supplemental data for the manuscript:

The role of serine/threonine-specific protein kinases in cyanobacteria - SpkB is involved in acclimation to fluctuating conditions in *Synechocystis* sp. PCC 6803

Thomas Barske[1]*, Philipp Spät[2,3]*, Hendrik Schubert[4], Peter Walke[1], Boris Maček[3], Martin Hagemann[1,5]**

* - the first two authors contributed equally to the study

1 – Institute of Biosciences, Department of Plant Physiology, University of Rostock, Rostock, Germany

2 - Interfaculty Institute of Microbiology and Infection Medicine Tübingen, Department of Organismic Interactions, University of Tübingen, Tübingen, Germany

3 - Interfaculty Institute for Cell Biology, Department of Quantitative Proteomics, University of Tübingen, Tübingen, Germany

4 - Institute of Biosciences, Department of Aquatic Ecology, University of Rostock, Rostock, Germany

5 – Interdisciplinary Faculty, Department Life, Light and Matter, University of Rostock, Rostock, Germany

Corresponding author: Martin Hagemann, Institut für Biowissenschaften, Abteilung Pflanzenphysiologie, Universität Rostock, A.-Einstein-Str. 3, Rostock D-18059, Germany; Tel: +49(0)3814986110; Fax: +49(0)3814986112; Email: martin.hagemann@uni-rostock.de

Supplemental spectra of single peptide identifications

20211217_PS_SCy031_Proteome_R2_24hLC_F1 − Scan number: 12761

Gene Names	Charge	m/z	Mass	Mass error [Da]	Mass error [ppm]	Retention time	PEP	Score	Precursor Intensity
sll0146	3	420.5679	1258.682	NA	NA	19.779	3.7899e−05	108.7	NA

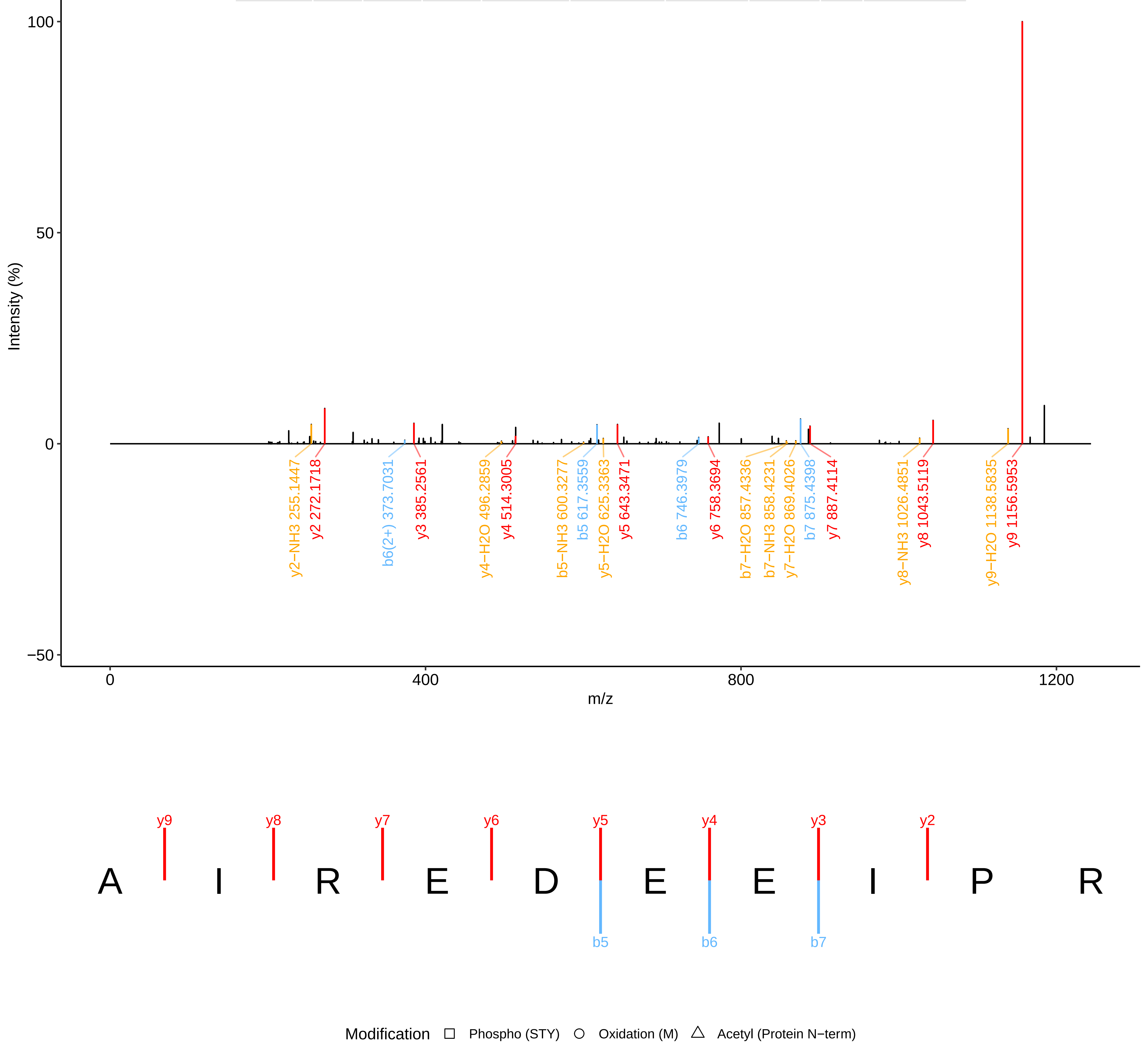

20211212_PS_SCy031_proteome_R1_3hLC_F4 − Scan number: 28359

Gene Names	Charge	m/z	Mass	Mass error [Da]	Mass error [ppm]	Retention time	PEP	Score	Precursor Intensity
sll0167	2	956.5095	1911.005	−0.00049862	−0.52097	41.192	1.4064e−58	225.32	9098824

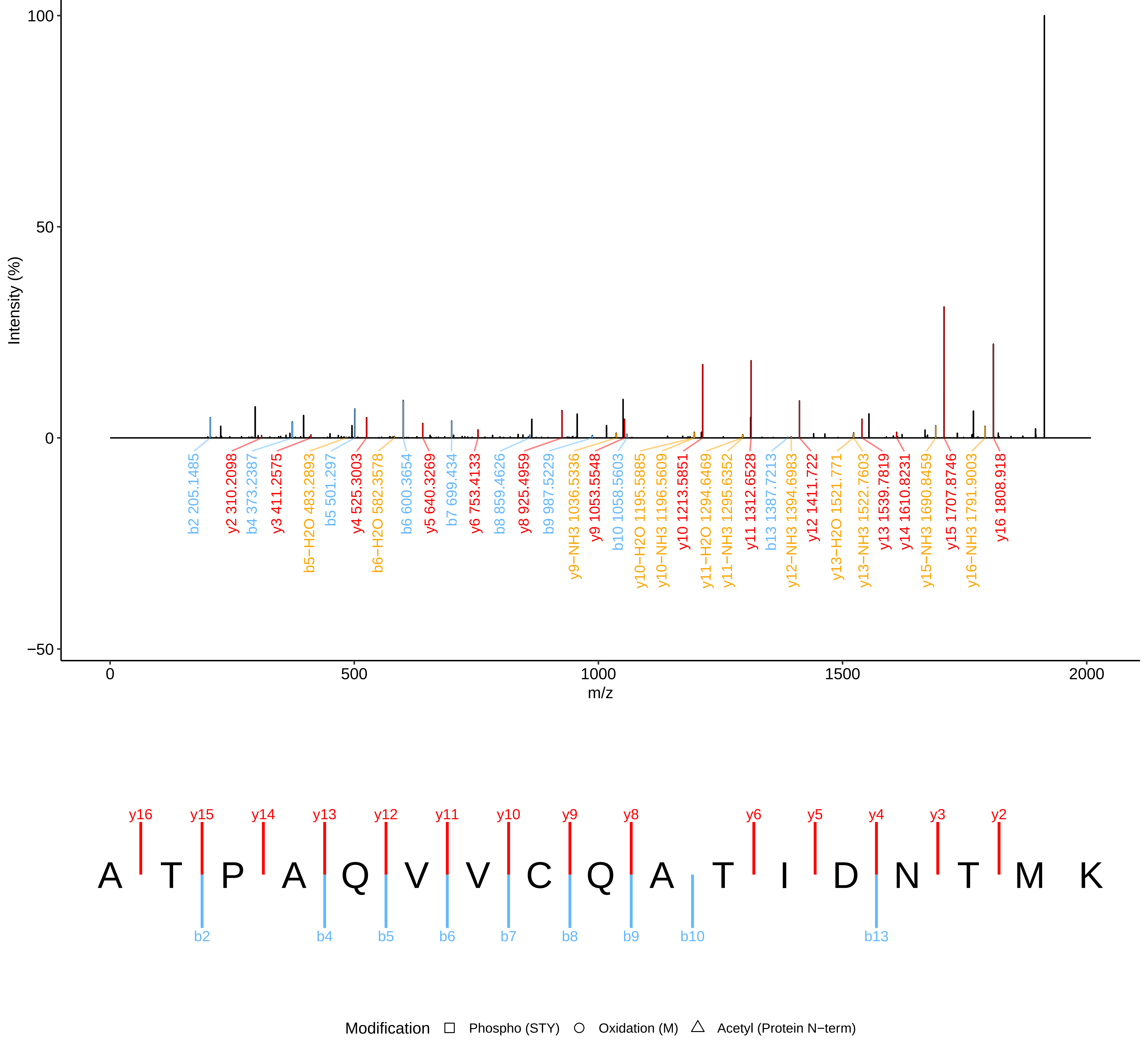

20211212_PS_SCy031_proteome_R1_24hLC_F5 − Scan number: 26632

Gene Names	Charge	m/z	Mass	Mass error [Da]	Mass error [ppm]	Retention time	PEP	Score	Precursor Intensity
sll0189	2	491.843	981.6714	−8.8724e−05	−0.19297	38.899	0.00044333	72.789	3276756

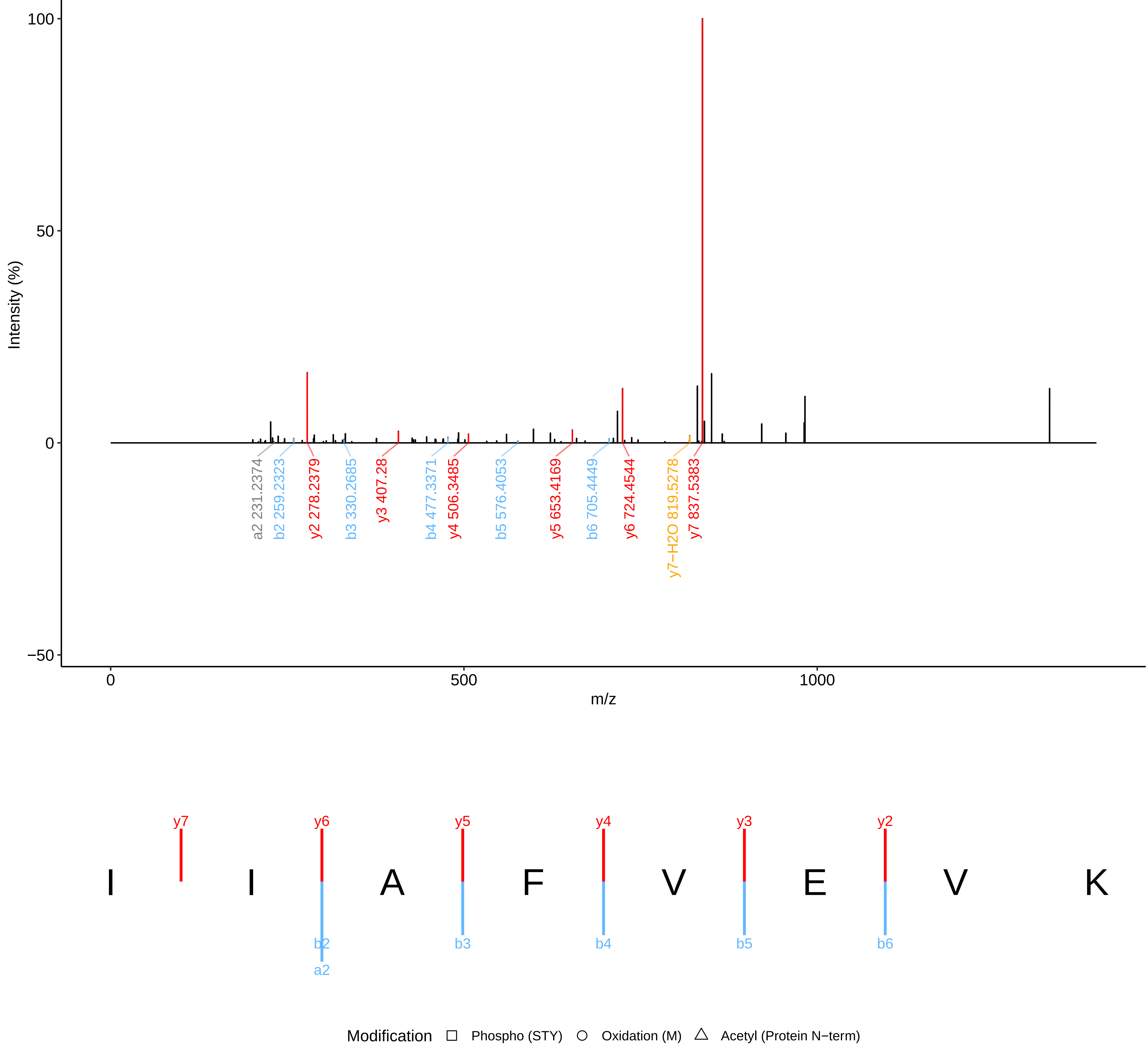

20211212_PS_SCy031_proteome_R1_HC_F1 − Scan number: 29252

Gene Names	Charge	m/z	Mass	Mass error [Da]	Mass error [ppm]	Retention time	PEP	Score	Precursor Intensity
sll0253	3	778.0096	2331.007	0.00058614	0.75306	41.057	0.00016908	85.927	3179082

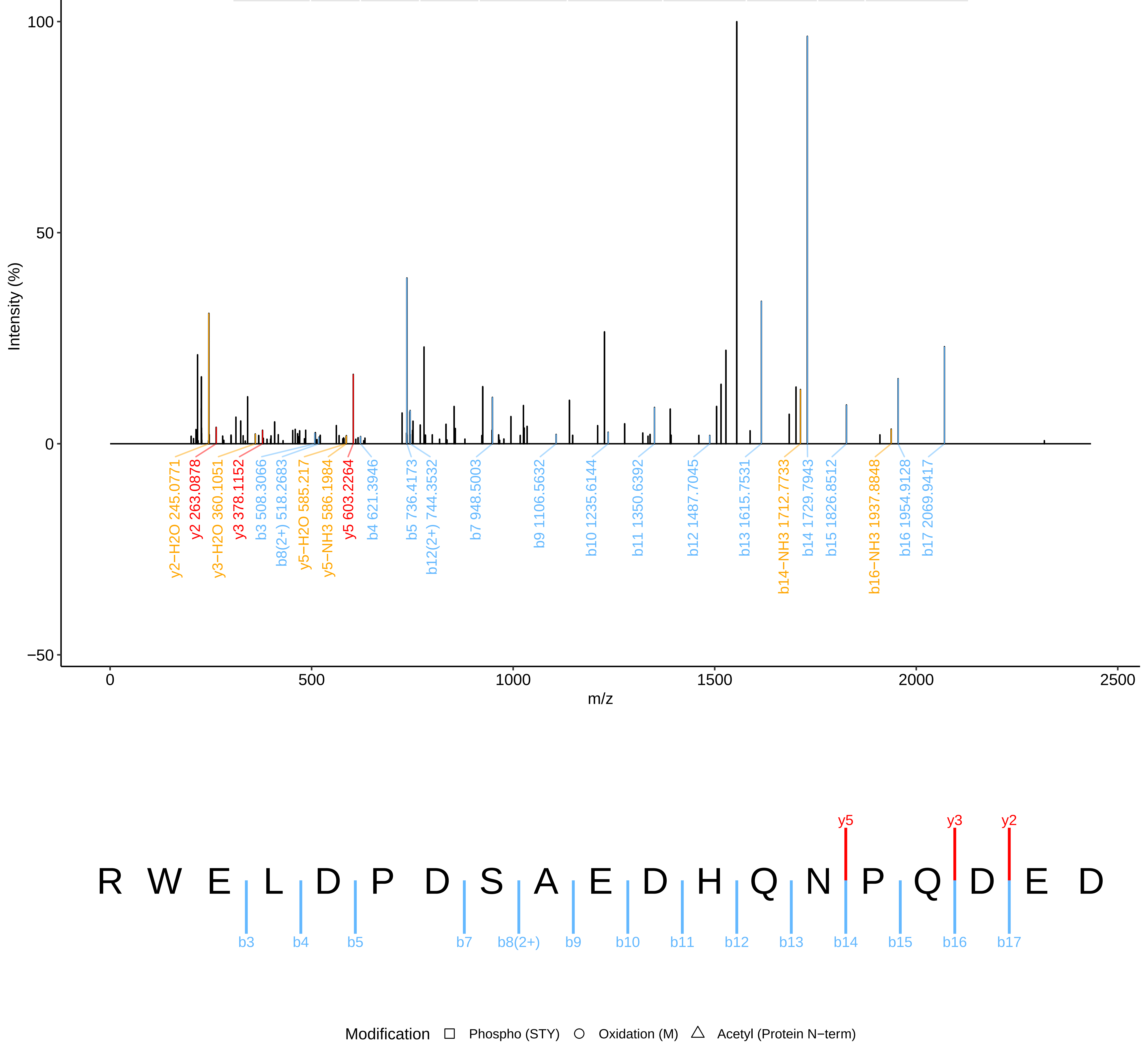

20211217_PS_SCy031_Proteome_R2_3hLC_F9 − Scan number: 18821

Gene Names	Charge	m/z	Mass	Mass error [Da]	Mass error [ppm]	Retention time	PEP	Score	Precursor Intensity
sll0364	2	450.7795	899.5444	−0.00053724	−1.2415	33.558	0.0001605	98.513	2915428

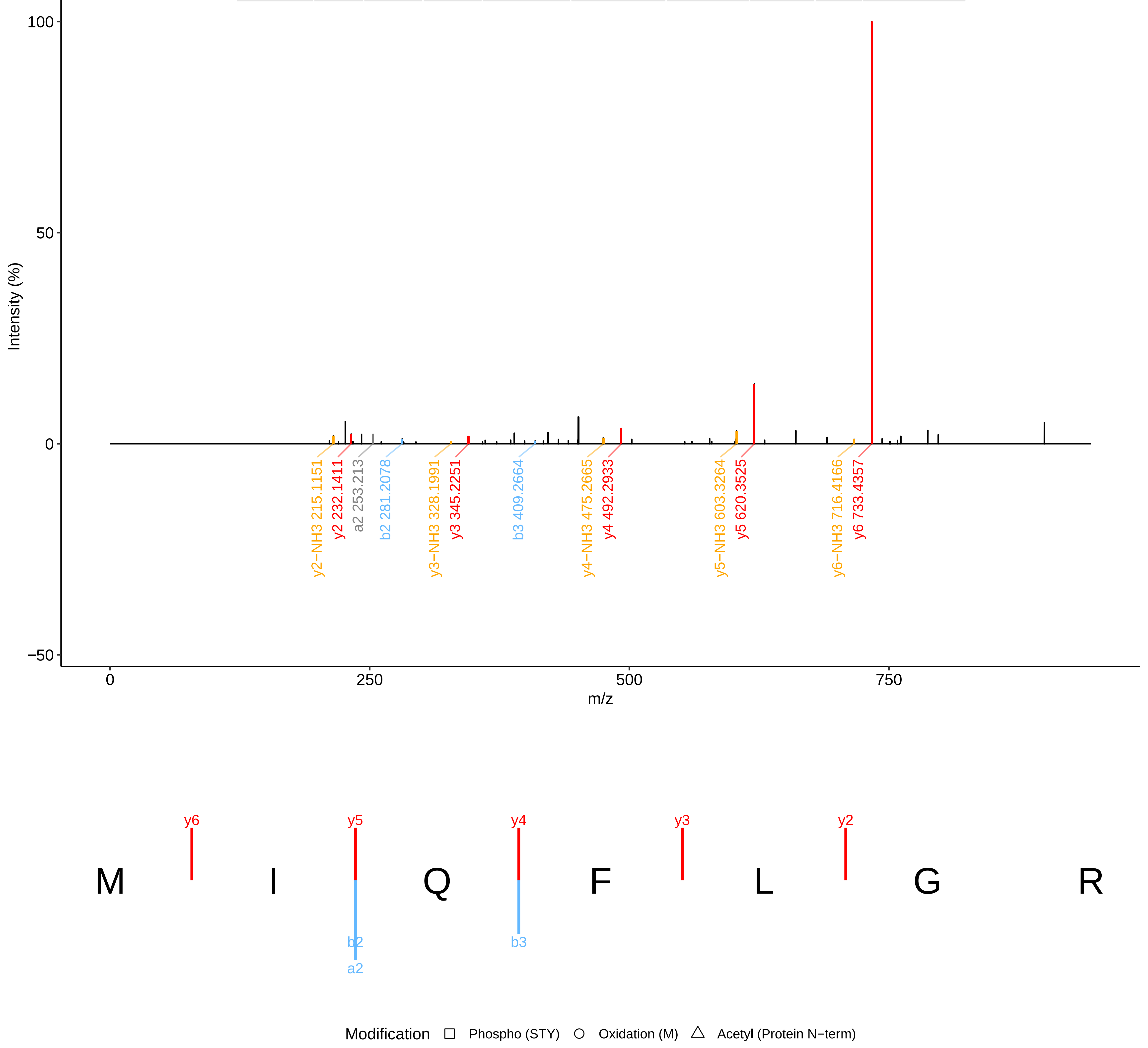

20211212_PS_SCy031_proteome_R1_HC_F8 − Scan number: 29637

Gene Names	Charge	m/z	Mass	Mass error [Da]	Mass error [ppm]	Retention time	PEP	Score	Precursor Intensity
sll0382	3	687.3619	2059.064	−4.5759e−05	−0.06749	45.677	8.8558e−24	141.97	6265856

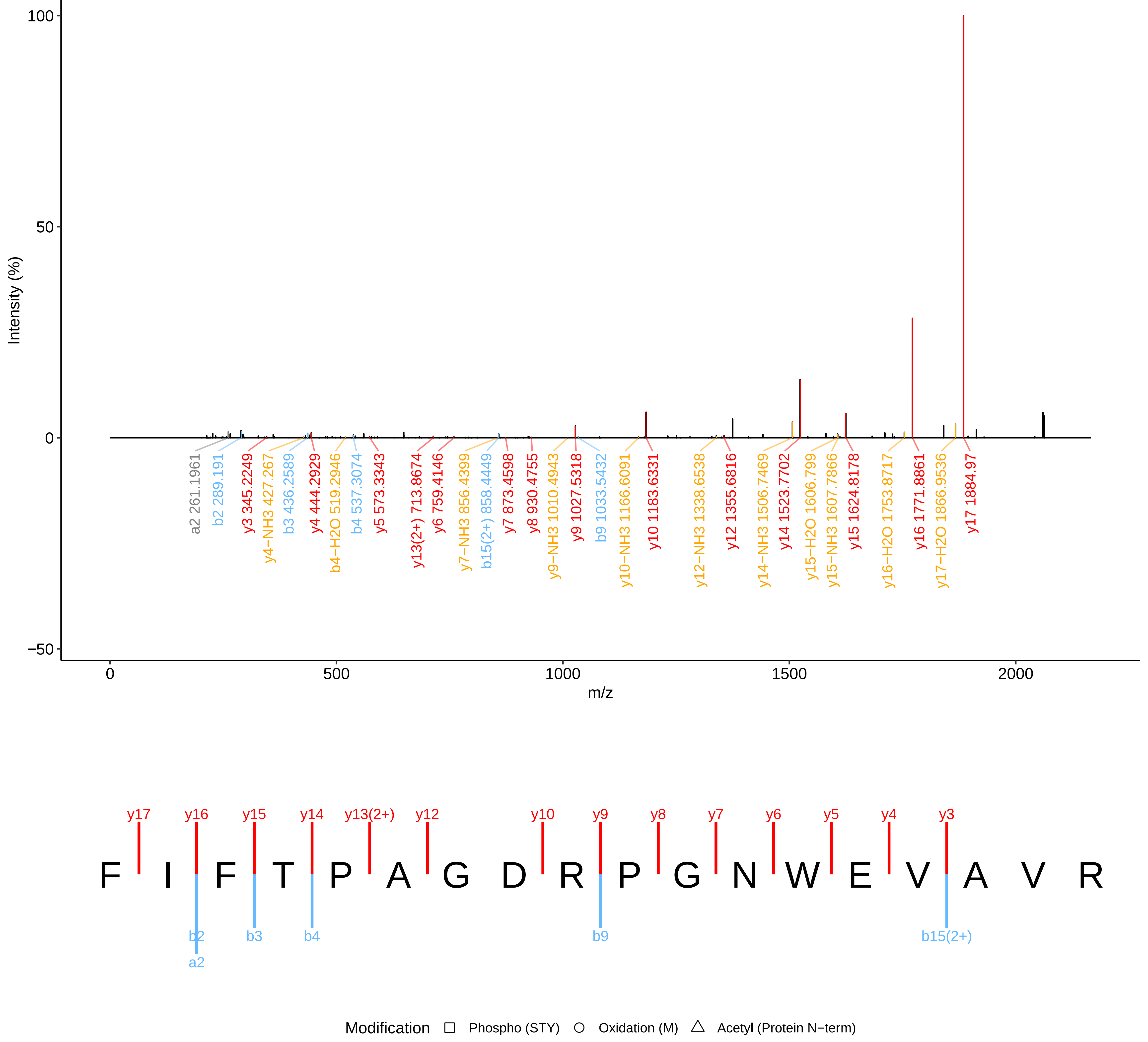

20211217_PS_SCy031_Proteome_R3_HC_F6 − Scan number: 6815

Gene Names	Charge	m/z	Mass	Mass error [Da]	Mass error [ppm]	Retention time	PEP	Score	Precursor Intensity
sll0385	3	397.2369	1188.689	−0.00035536	−0.94545	14.581	6.0373e−05	70.399	2241999

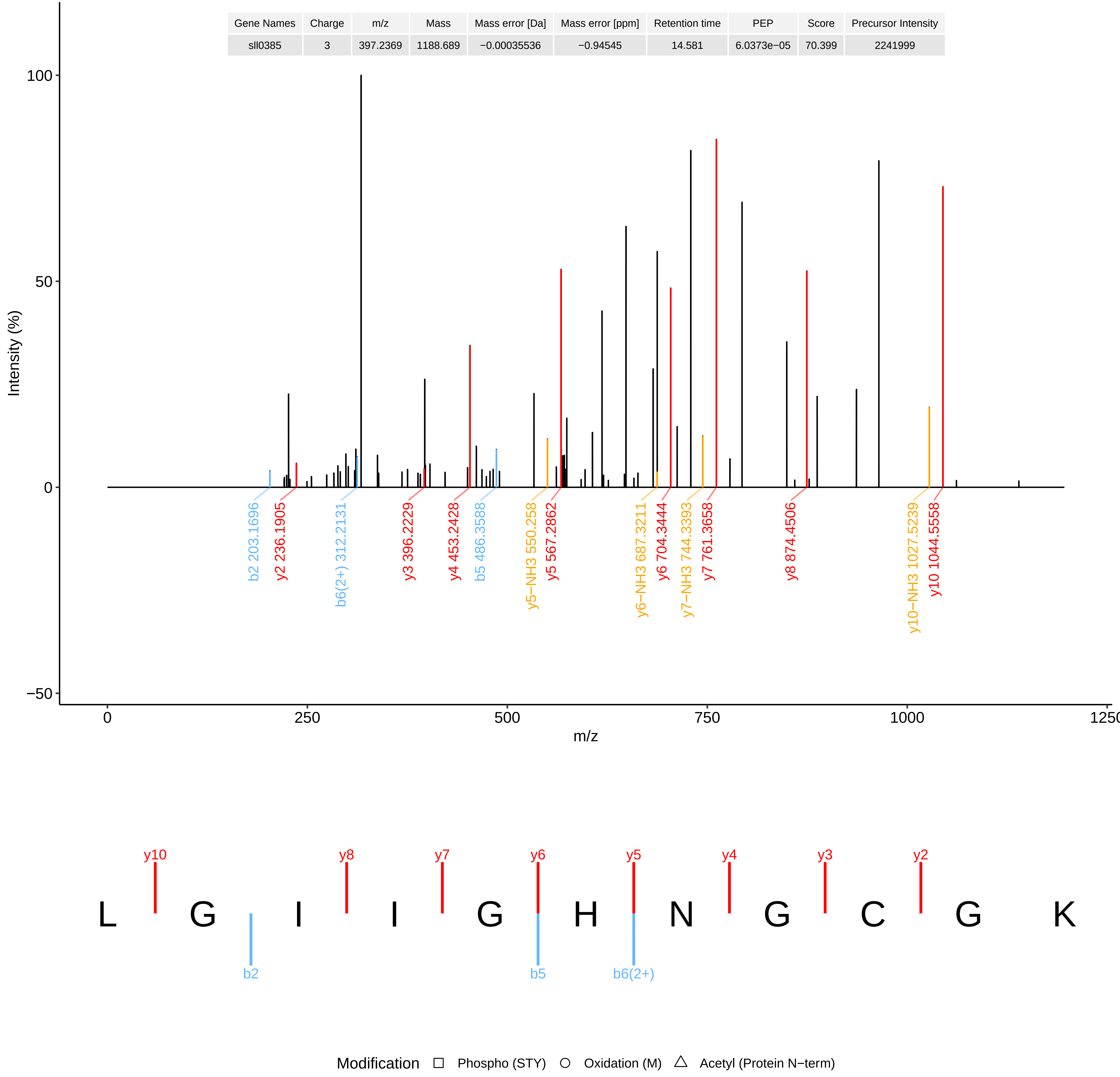

20211217_PS_SCy031_Proteome_R2_24hLC_F9 − Scan number: 31016

Gene Names	Charge	m/z	Mass	Mass error [Da]	Mass error [ppm]	Retention time	PEP	Score	Precursor Intensity
sll0403	2	714.8777	1427.741	0.00015146	0.21172	49.706	0.00077569	97.911	4538221

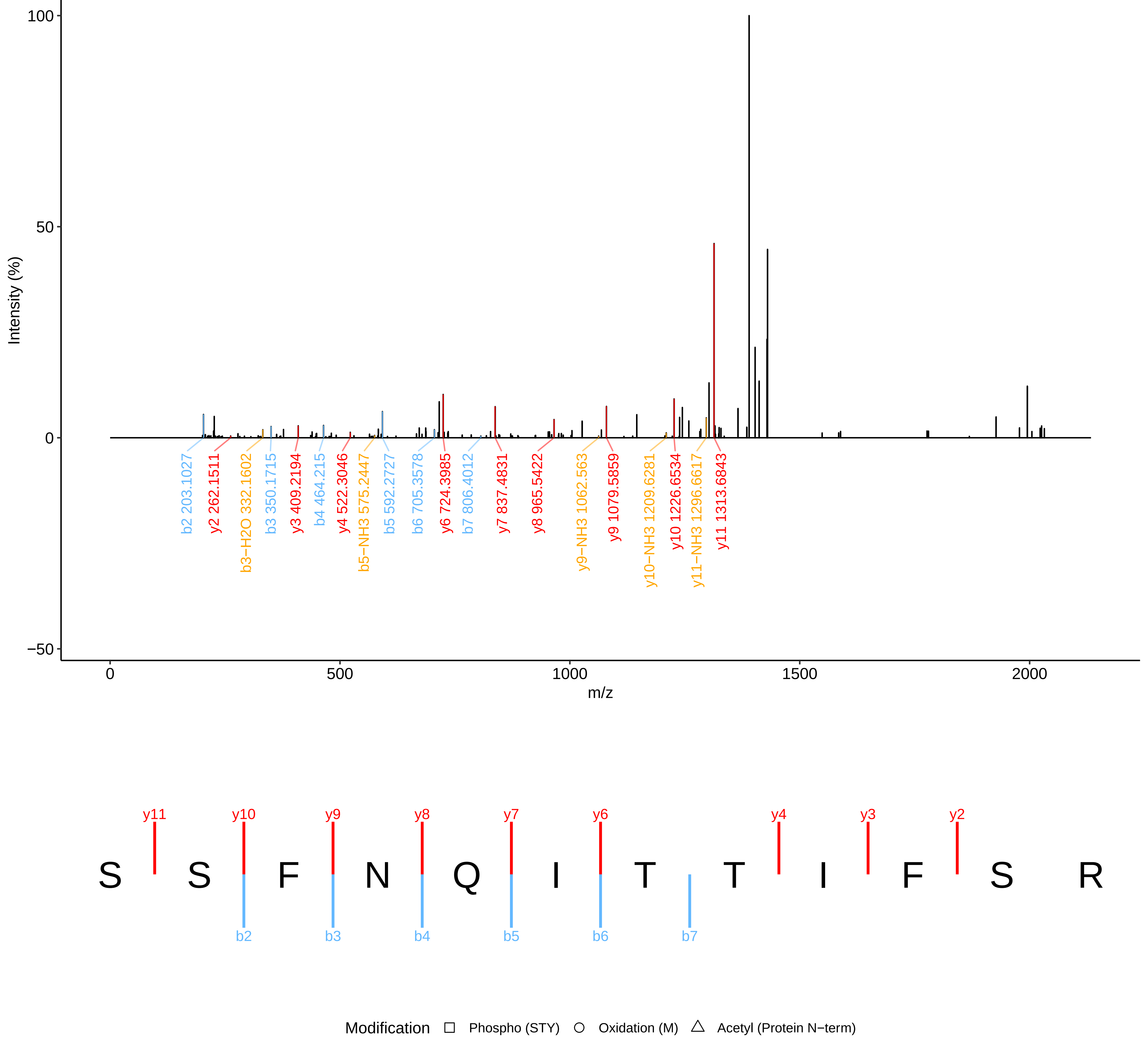

20211217_PS_SCy031_Proteome_R2_HC_F6 − Scan number: 28775

Gene Names	Charge	m/z	Mass	Mass error [Da]	Mass error [ppm]	Retention time	PEP	Score	Precursor Intensity
sll0419	2	793.0291	1584.044	NA	NA	45.004	0.00014331	92.866	816768

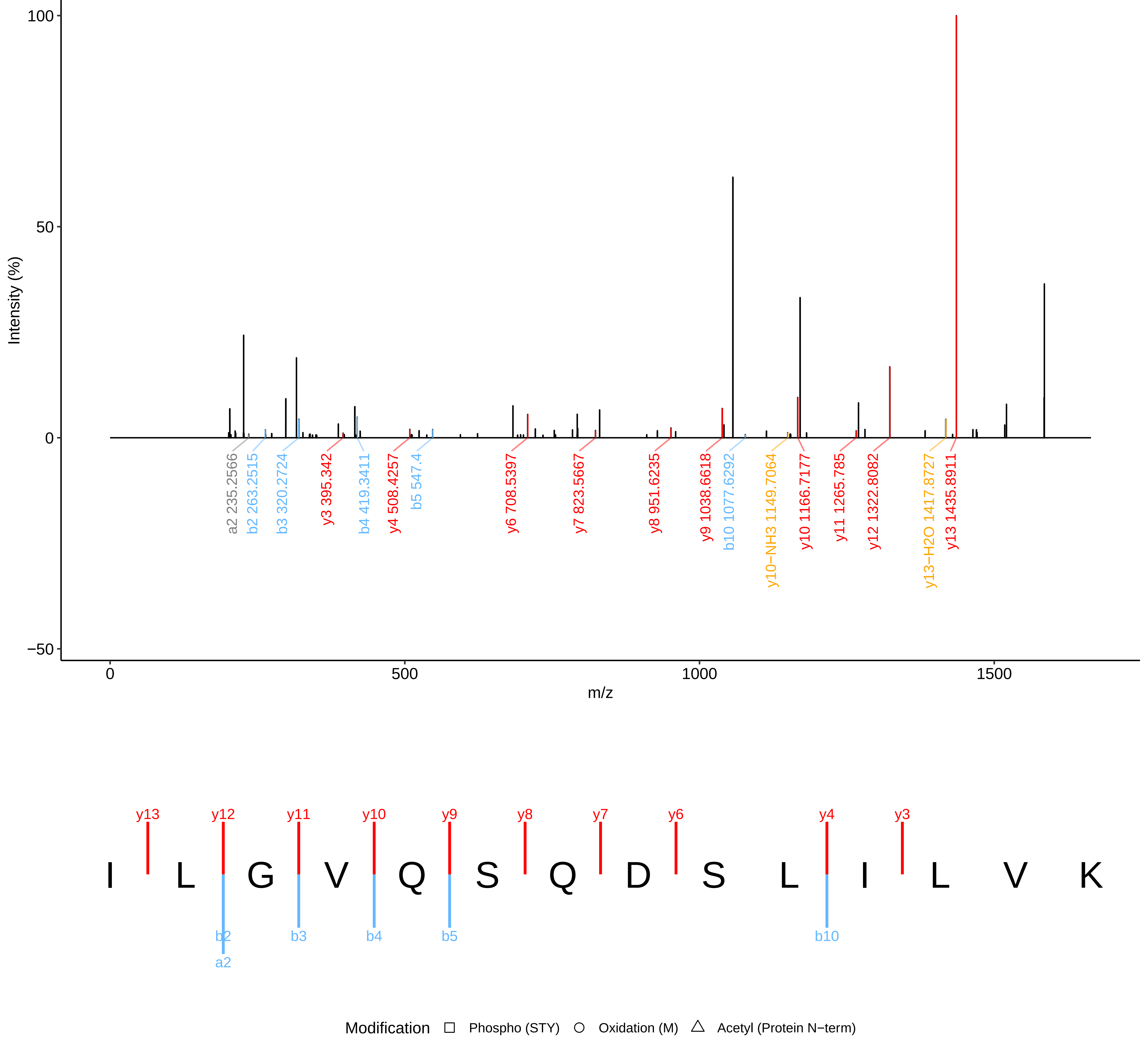

20211212_PS_SCy031_proteome_R1_24hLC_F3 − Scan number: 11346

Gene Names	Charge	m/z	Mass	Mass error [Da]	Mass error [ppm]	Retention time	PEP	Score	Precursor Intensity
sll0488	3	477.9162	1430.727	−0.00059994	−1.2804	18.587	5.9696e−06	99.539	18374518

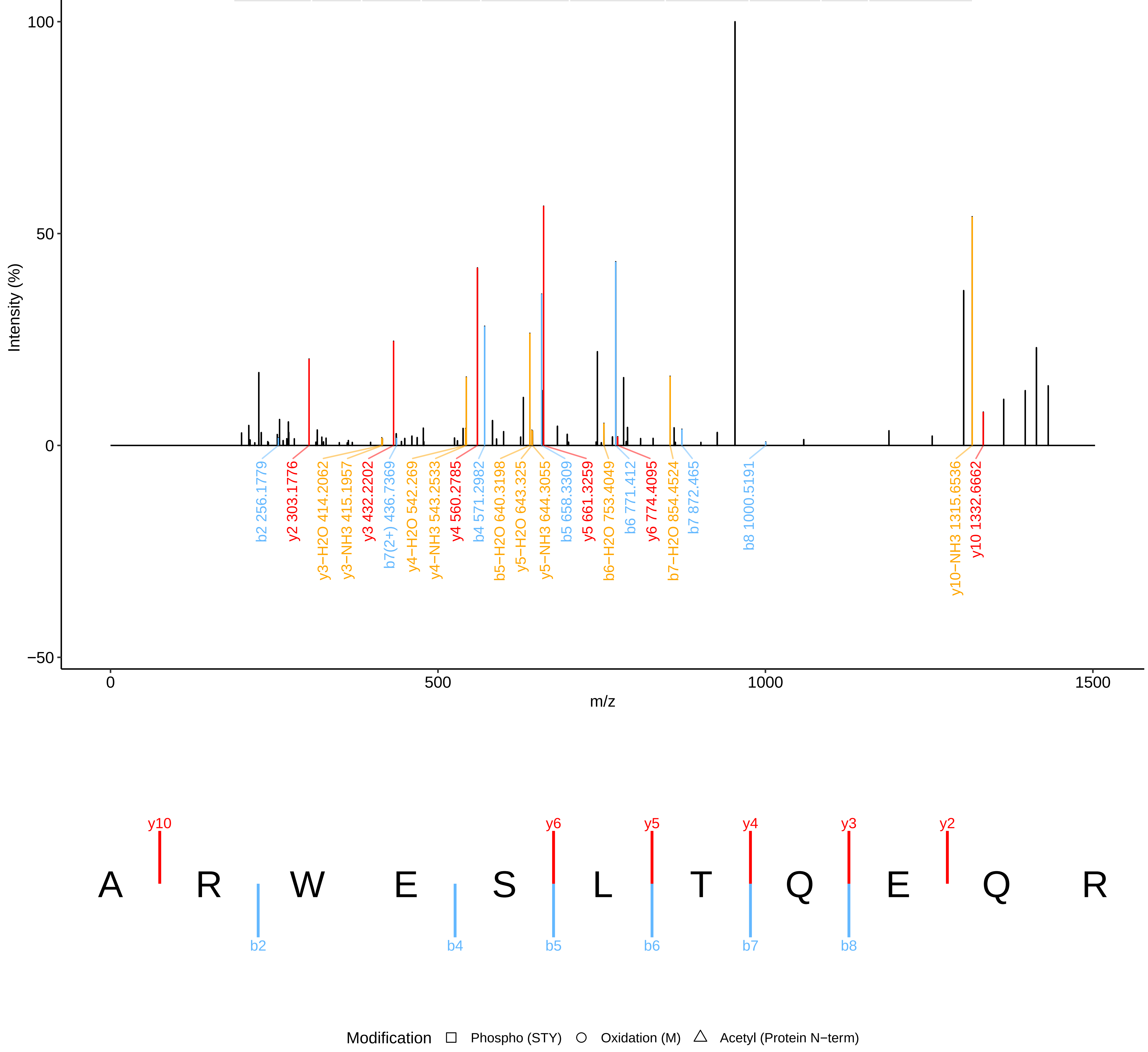

20211212_PS_SCy031_proteome_R1_3hLC_F8 − Scan number: 15726

Gene Names	Charge	m/z	Mass	Mass error [Da]	Mass error [ppm]	Retention time	PEP	Score	Precursor Intensity
sll0574	2	514.2878	1026.561	0.00017454	0.3489	25.893	0.00060737	67.981	2236401

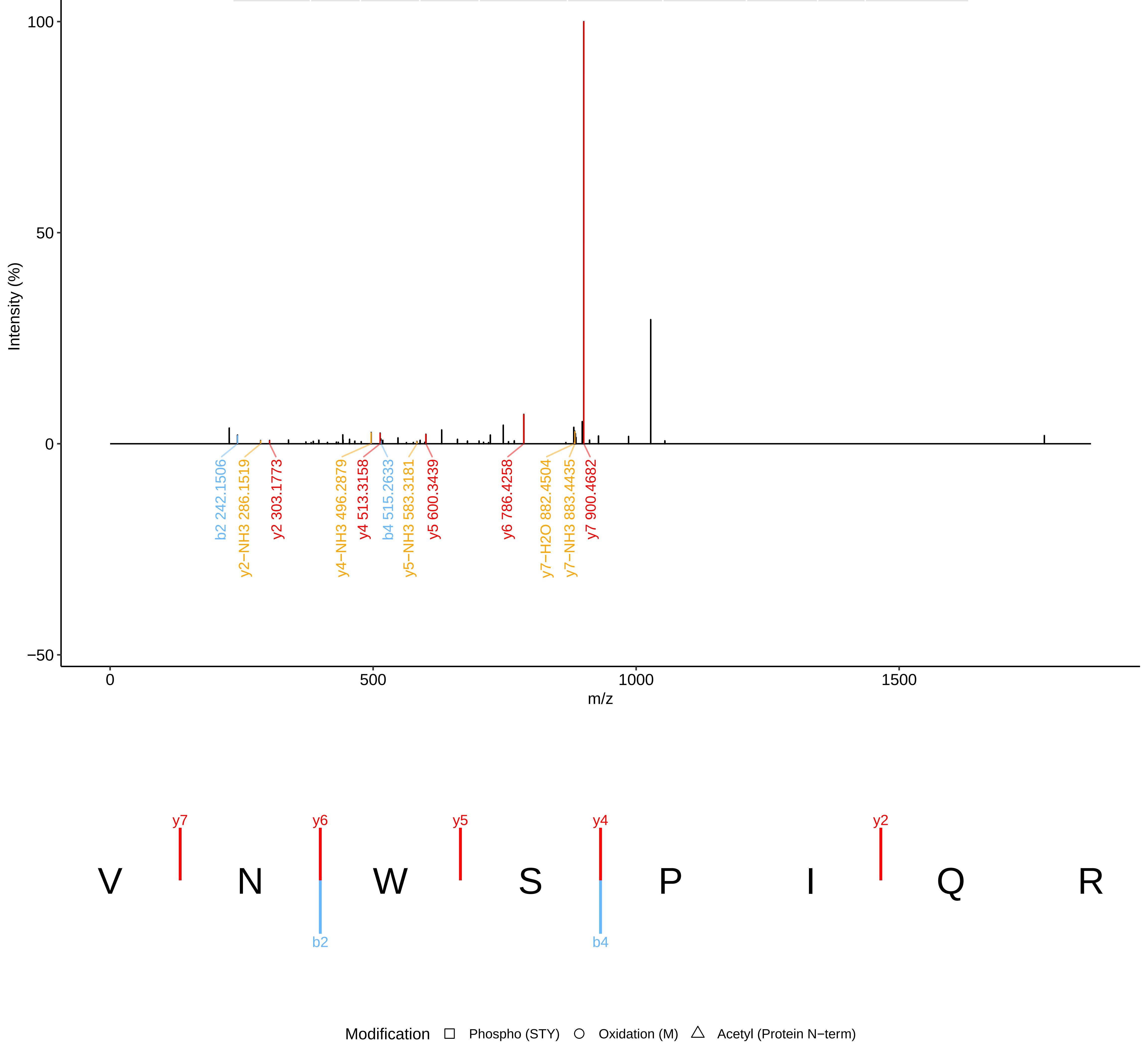

20211217_PS_SCy031_Proteome_R3_HC_F2 − Scan number: 23723

Gene Names	Charge	m/z	Mass	Mass error [Da]	Mass error [ppm]	Retention time	PEP	Score	Precursor Intensity
sll0614	2	566.8161	1131.618	1.7531e−05	0.032538	35.019	8.0478e−08	110.87	4947225

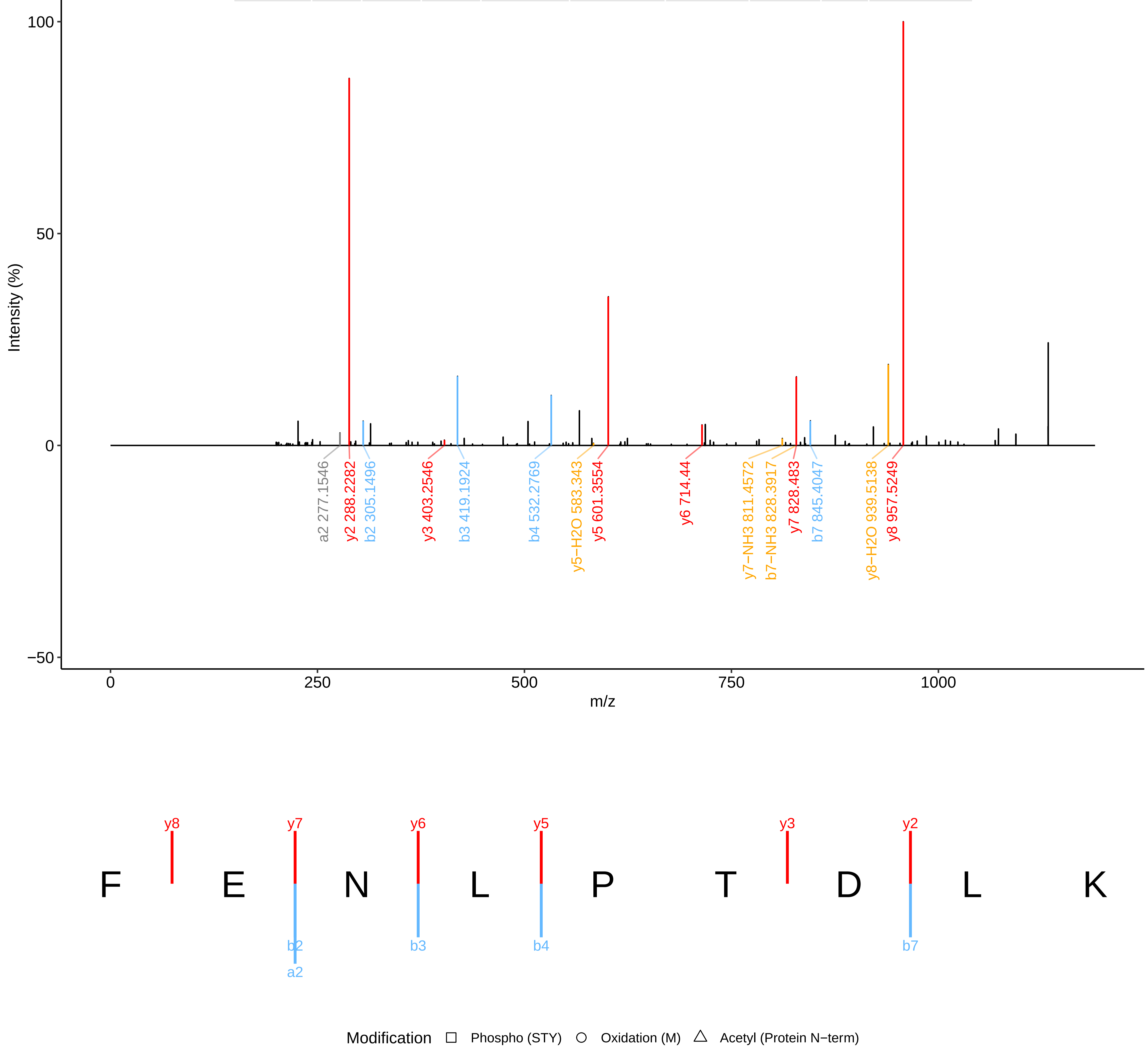

20211212_PS_SCy031_proteome_R1_3hLC_F1 − Scan number: 26025

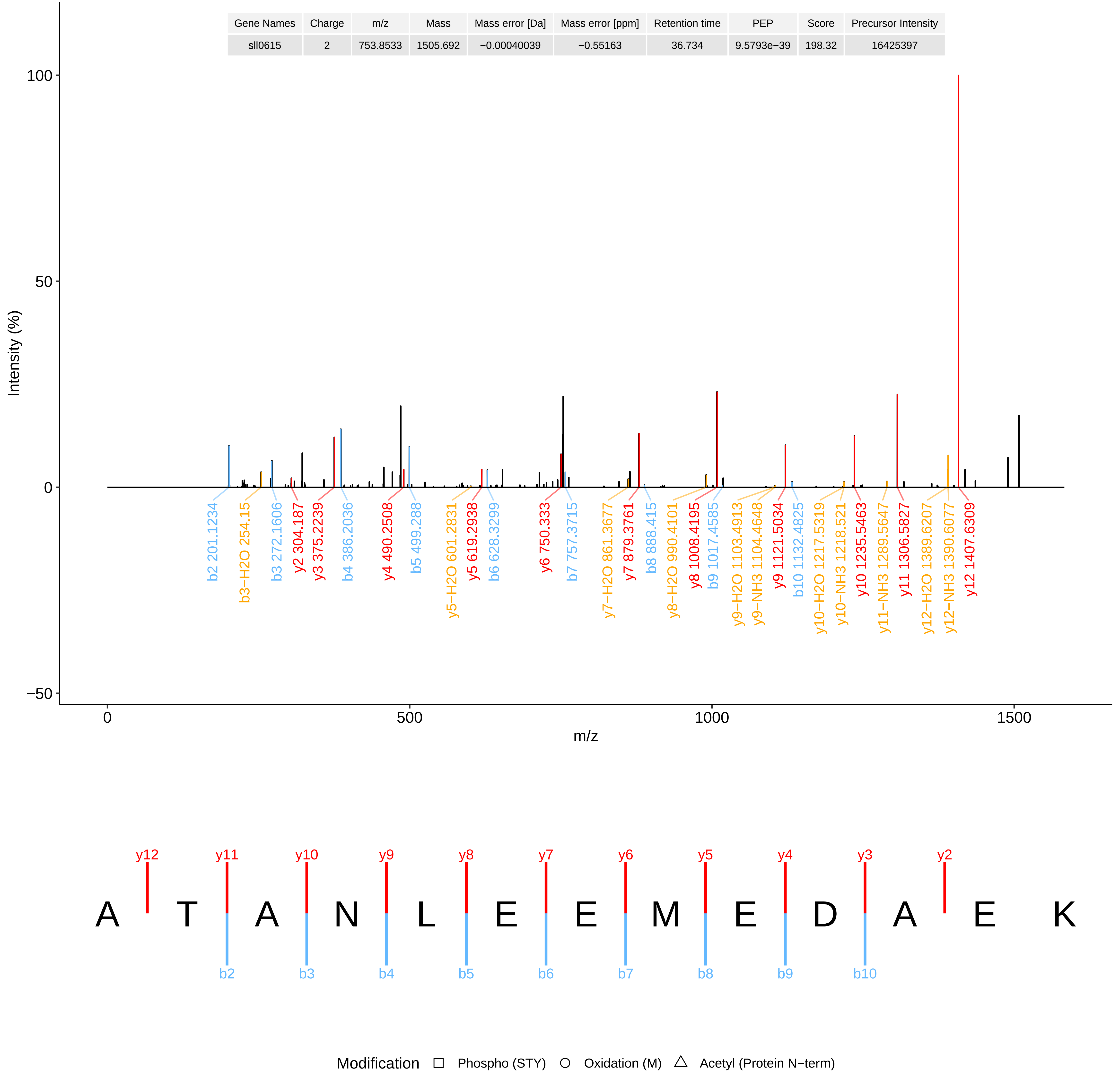

20211212_PS_SCy031_proteome_R1_HC_F3 − Scan number: 8972

Gene Names	Charge	m/z	Mass	Mass error [Da]	Mass error [ppm]	Retention time	PEP	Score	Precursor Intensity
sll0657	3	553.6486	1657.924	1.6308e−05	0.030637	15.73	8.2254e−34	190.87	6056113

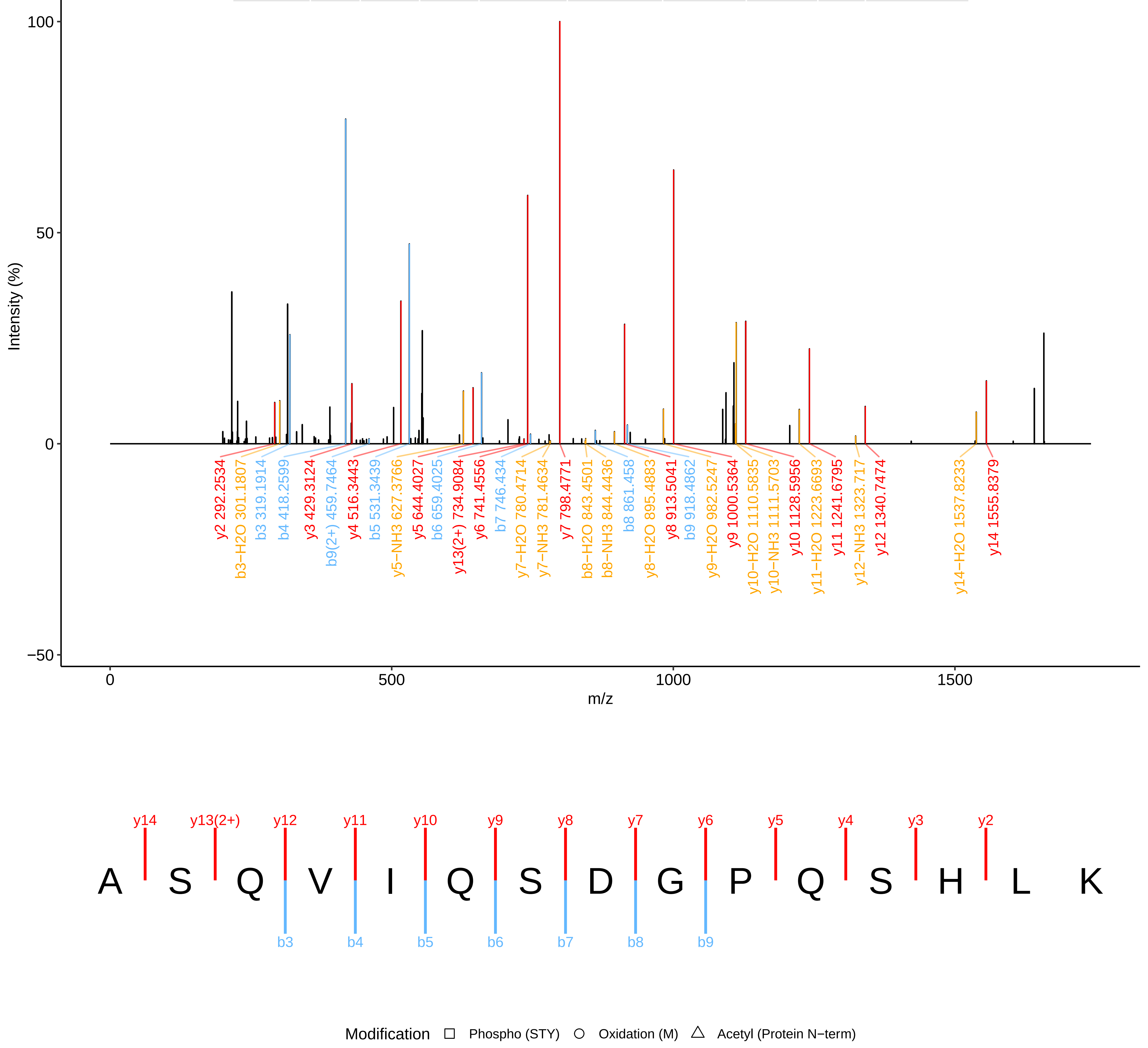

20211212_PS_SCy031_proteome_R1_3hLC_F9 − Scan number: 30911

Gene Names	Charge	m/z	Mass	Mass error [Da]	Mass error [ppm]	Retention time	PEP	Score	Precursor Intensity
sll0802	3	352.2702	1053.789	NA	NA	45.624	0.0003729	77.674	7764132

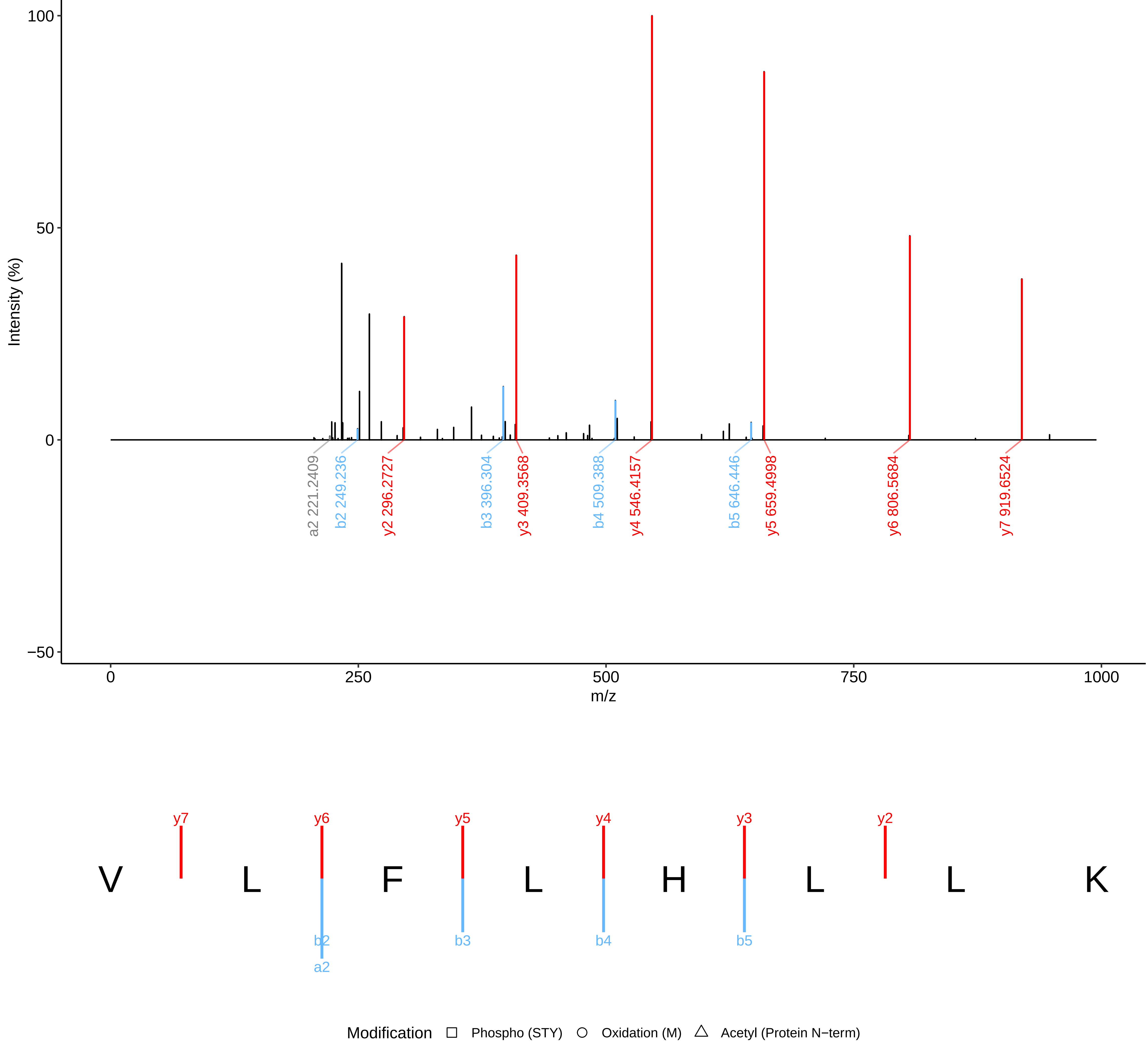

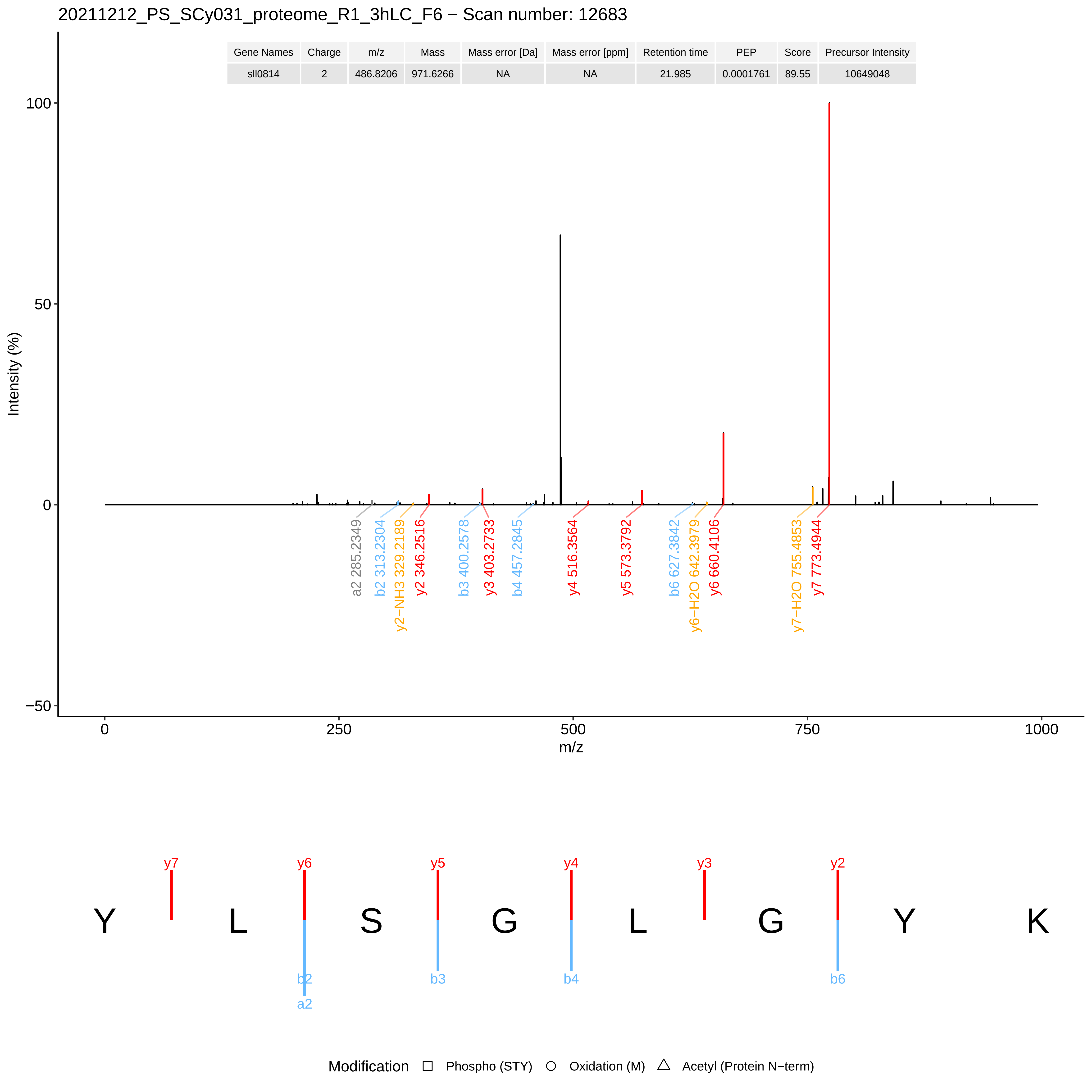

20211212_PS_SCy031_proteome_R1_3hLC_F6 − Scan number: 12683
Gene Names | Charge | m/z | Mass | Mass error [Da] | Mass error [ppm] | Retention time | PEP | Score | Precursor Intensity
sll0814 | 2 | 486.8206 | 971.6266 | NA | NA | 21.985 | 0.0001761 | 89.55 | 10649048
Intensity (%)
m/z
a2 285.2349
b2 313.2304
y2−NH3 329.2189
y2 346.2516
b3 400.2578
y3 403.2733
b4 457.2845
y4 516.3564
y5 573.3792
b6 627.3842
y6−H2O 642.3979
y6 660.4106
y7−H2O 755.4853
y7 773.4944
Y L S G L G Y K
y7 y6 y5 y4 y3 y2
b2 a2 b3 b4 b6
Modification Phospho (STY) Oxidation (M) Acetyl (Protein N−term)

20211217_PS_SCy031_Proteome_R2_HC_F9 − Scan number: 28724

Gene Names	Charge	m/z	Mass	Mass error [Da]	Mass error [ppm]	Retention time	PEP	Score	Precursor Intensity
sll0875	2	768.4705	1534.926	1.5479e−05	0.020142	49.46	2.7878e−05	127.87	2239702

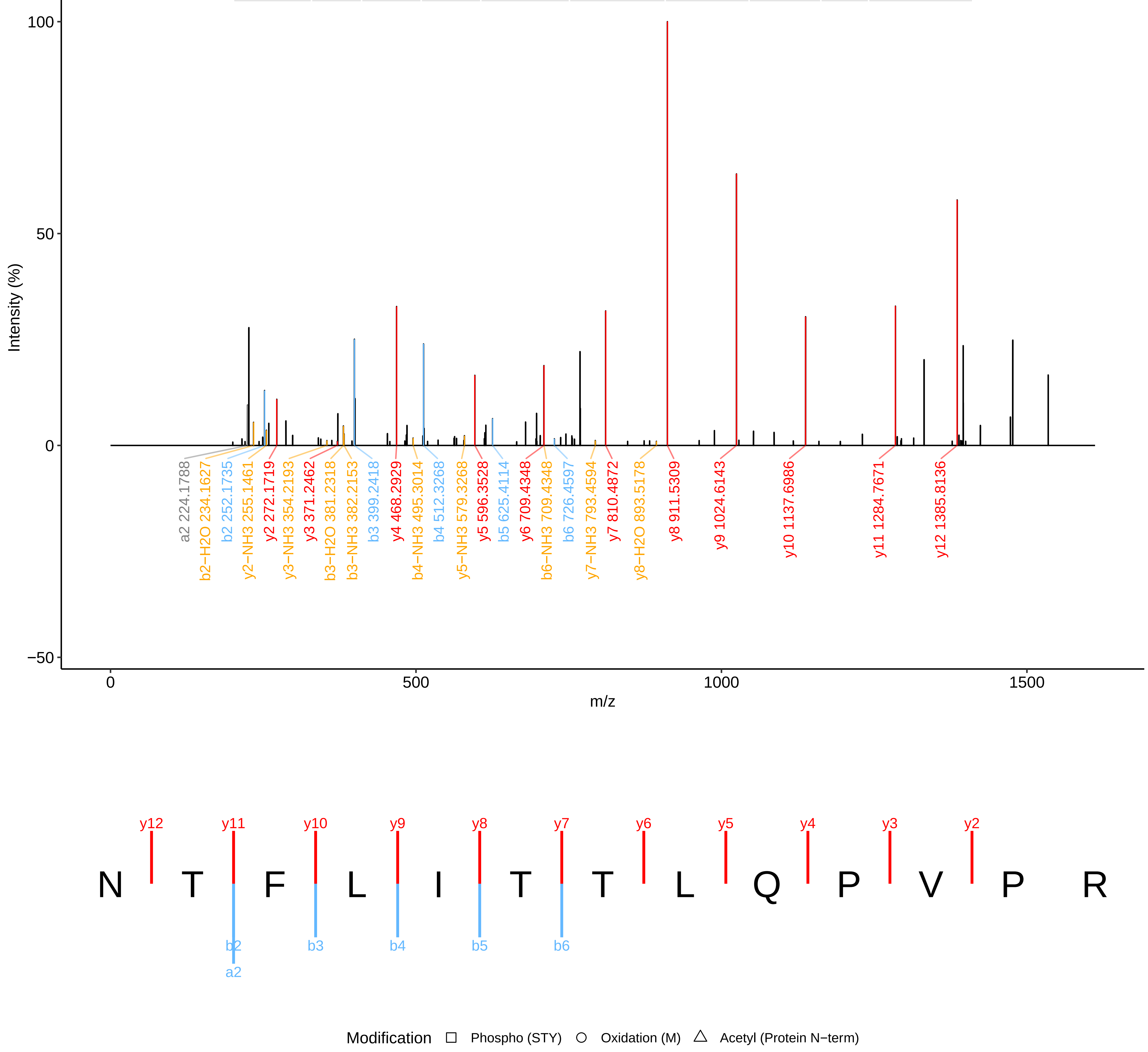

20211217_PS_SCy031_Proteome_R3_HC_F9 − Scan number: 19058

Gene Names	Charge	m/z	Mass	Mass error [Da]	Mass error [ppm]	Retention time	PEP	Score	Precursor Intensity
sll0896	3	543.6626	1627.966	0.00022266	0.40931	34.801	1.9682e−07	150.11	855275.8

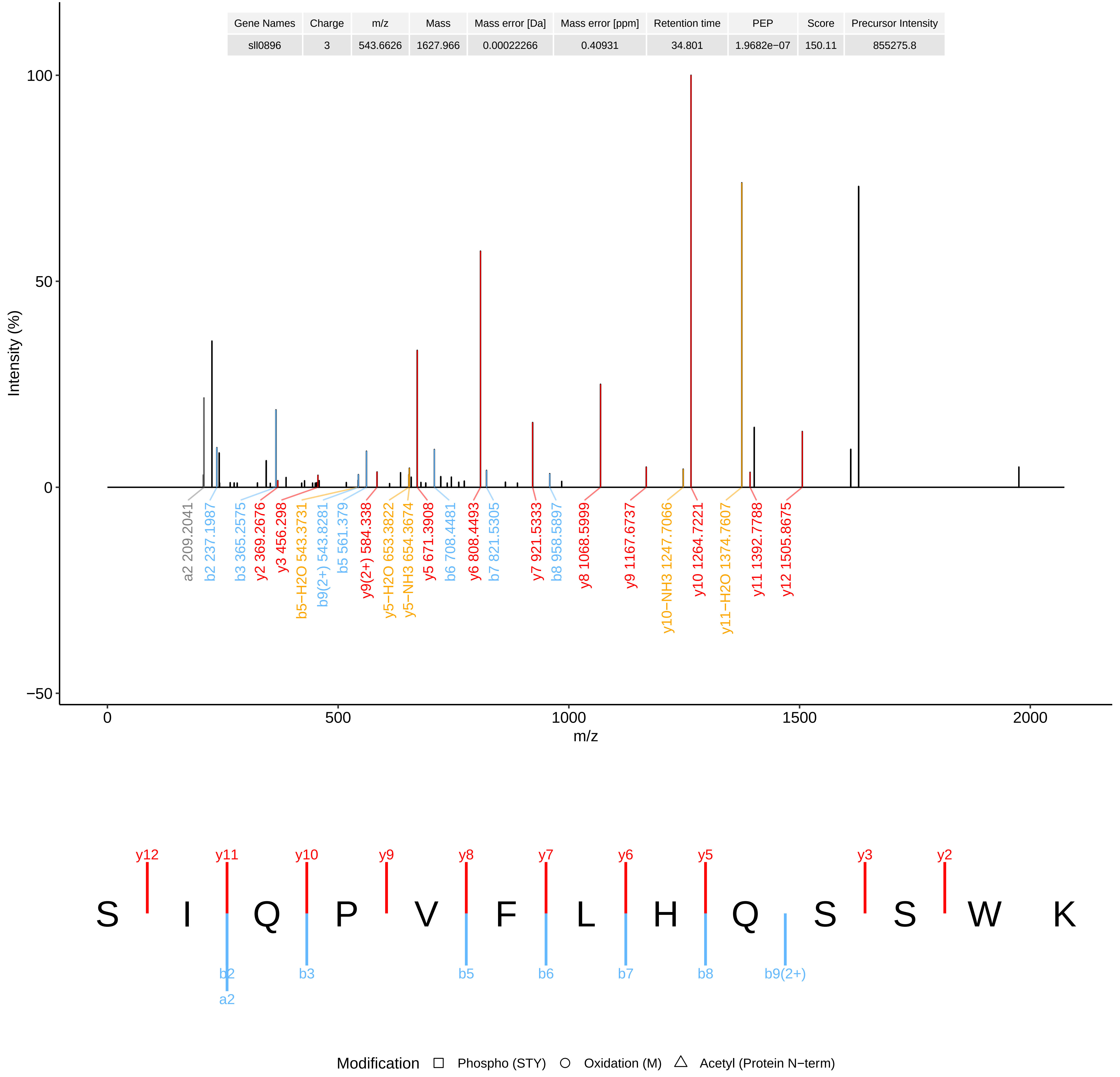

20211212_PS_SCy031_proteome_R1_HC_F3 − Scan number: 21344

Gene Names	Charge	m/z	Mass	Mass error [Da]	Mass error [ppm]	Retention time	PEP	Score	Precursor Intensity
sll1068	2	444.262	886.5094	6.3043e−06	0.014791	31.335	0.00015999	89.457	9961633

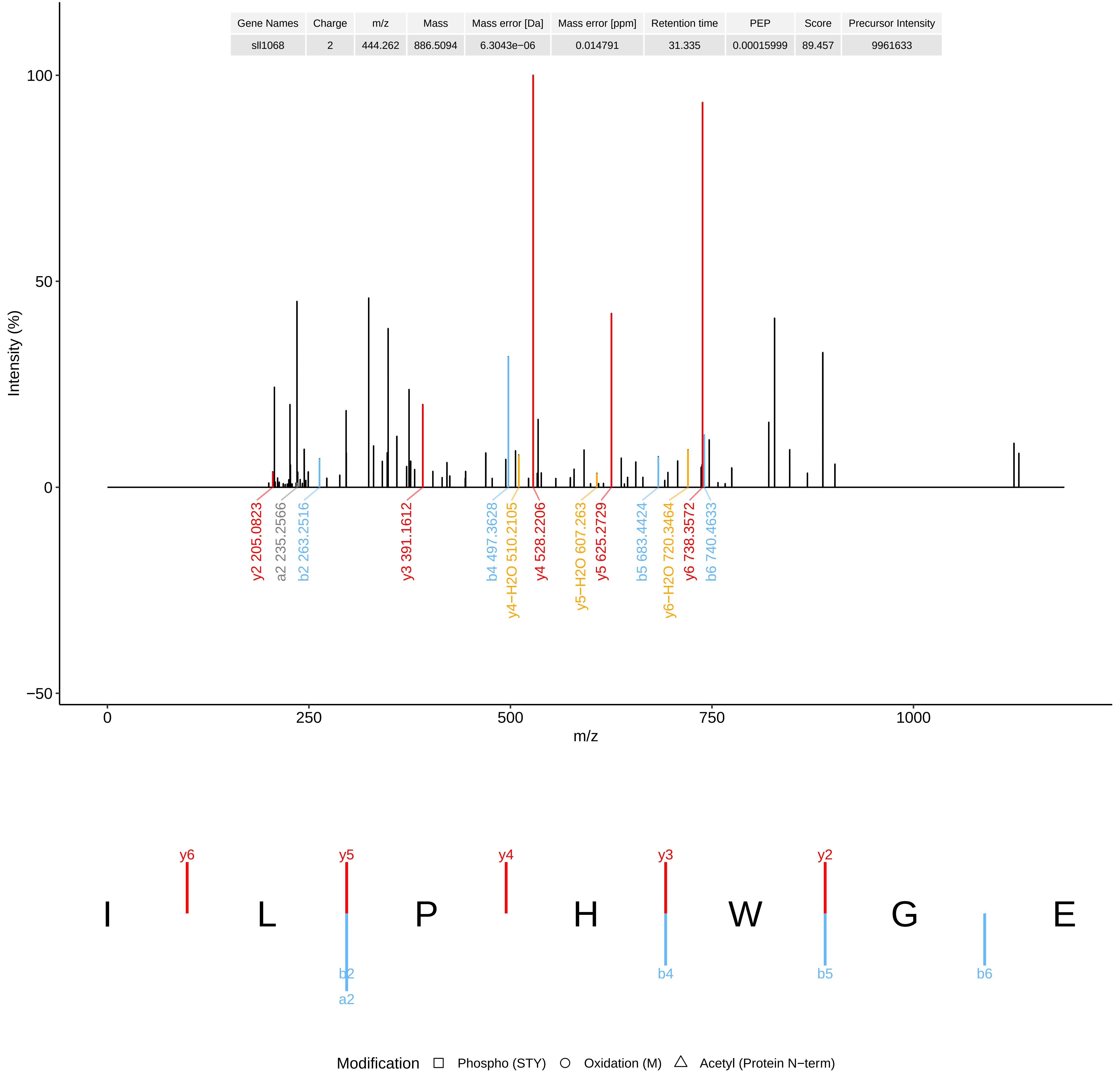

20211212_PS_SCy031_proteome_R1_3hLC_F9 − Scan number: 29693

Gene Names	Charge	m/z	Mass	Mass error [Da]	Mass error [ppm]	Retention time	PEP	Score	Precursor Intensity
sll1086	2	479.2976	956.5807	−1.4048e−05	−0.030191	44.077	1.3659e−05	123.99	5093321

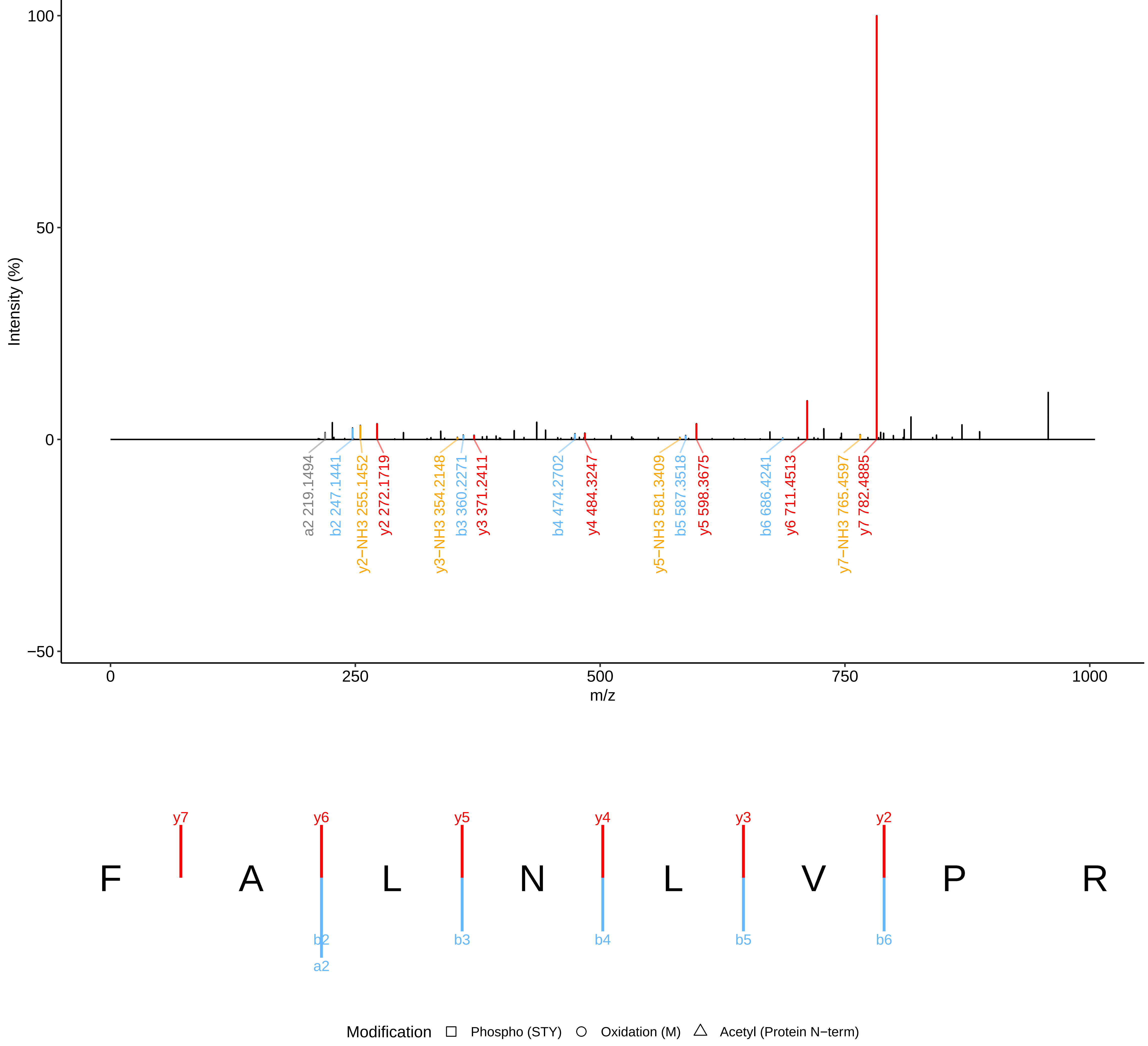

20211212_PS_SCy031_proteome_R1_HC_F2 − Scan number: 26388

Gene Names	Charge	m/z	Mass	Mass error [Da]	Mass error [ppm]	Retention time	PEP	Score	Precursor Intensity
sll1147	2	908.4296	1814.845	0.00087995	0.98605	38.132	3.5395e−21	151.1	1972011

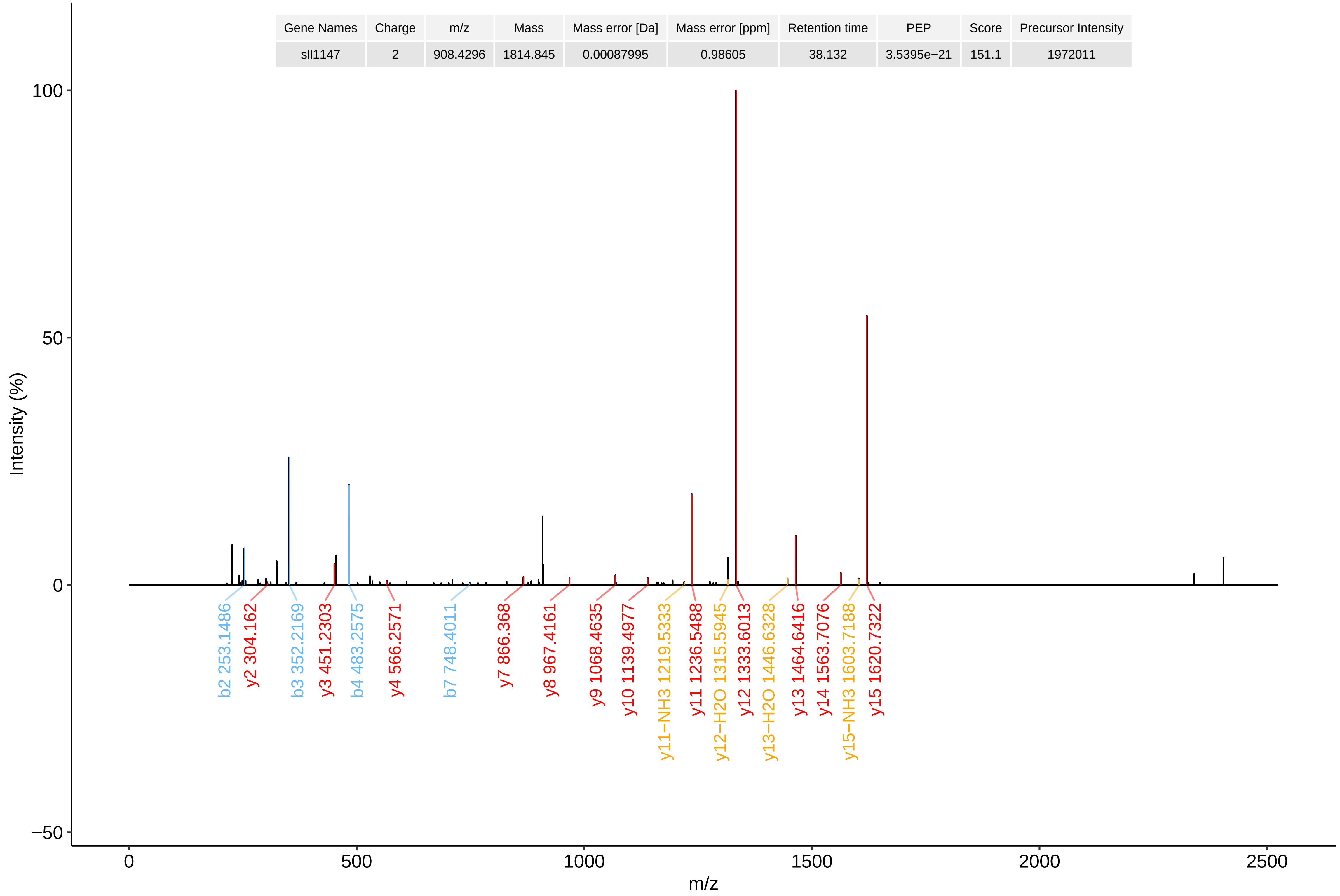

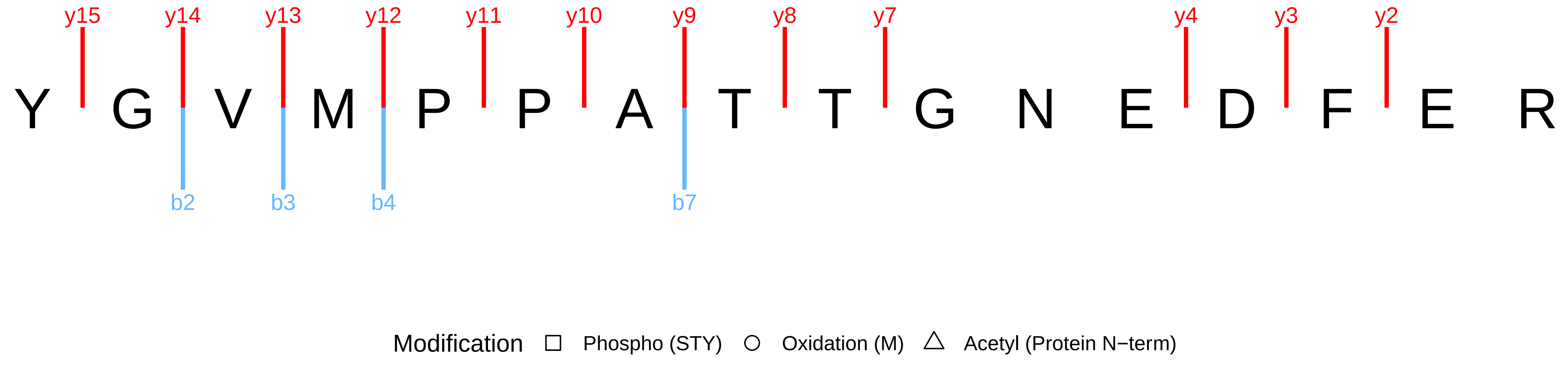

20211212_PS_SCy031_proteome_R1_24hLC_F2 − Scan number: 5231

Gene Names	Charge	m/z	Mass	Mass error [Da]	Mass error [ppm]	Retention time	PEP	Score	Precursor Intensity
sll1164	2	442.7423	883.4701	0.00038175	0.89464	10.772	0.00069052	66.994	16011254

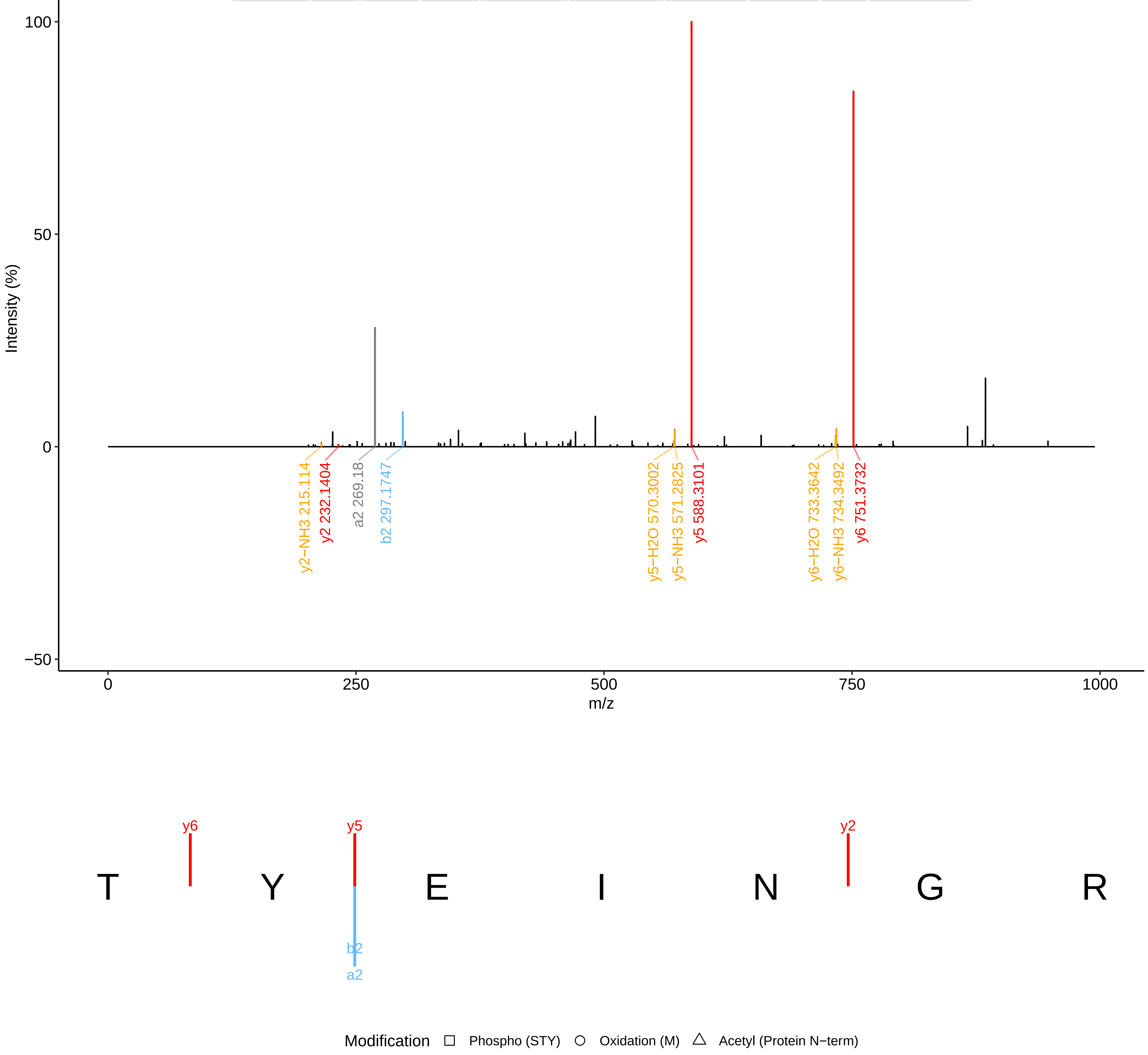

20211217_PS_SCy031_Proteome_R2_3hLC_F8 − Scan number: 24432

Gene Names	Charge	m/z	Mass	Mass error [Da]	Mass error [ppm]	Retention time	PEP	Score	Precursor Intensity
sll1267	2	668.428	1334.841	0.00067523	1.0611	42.344	2.5585e−06	108.67	2199788

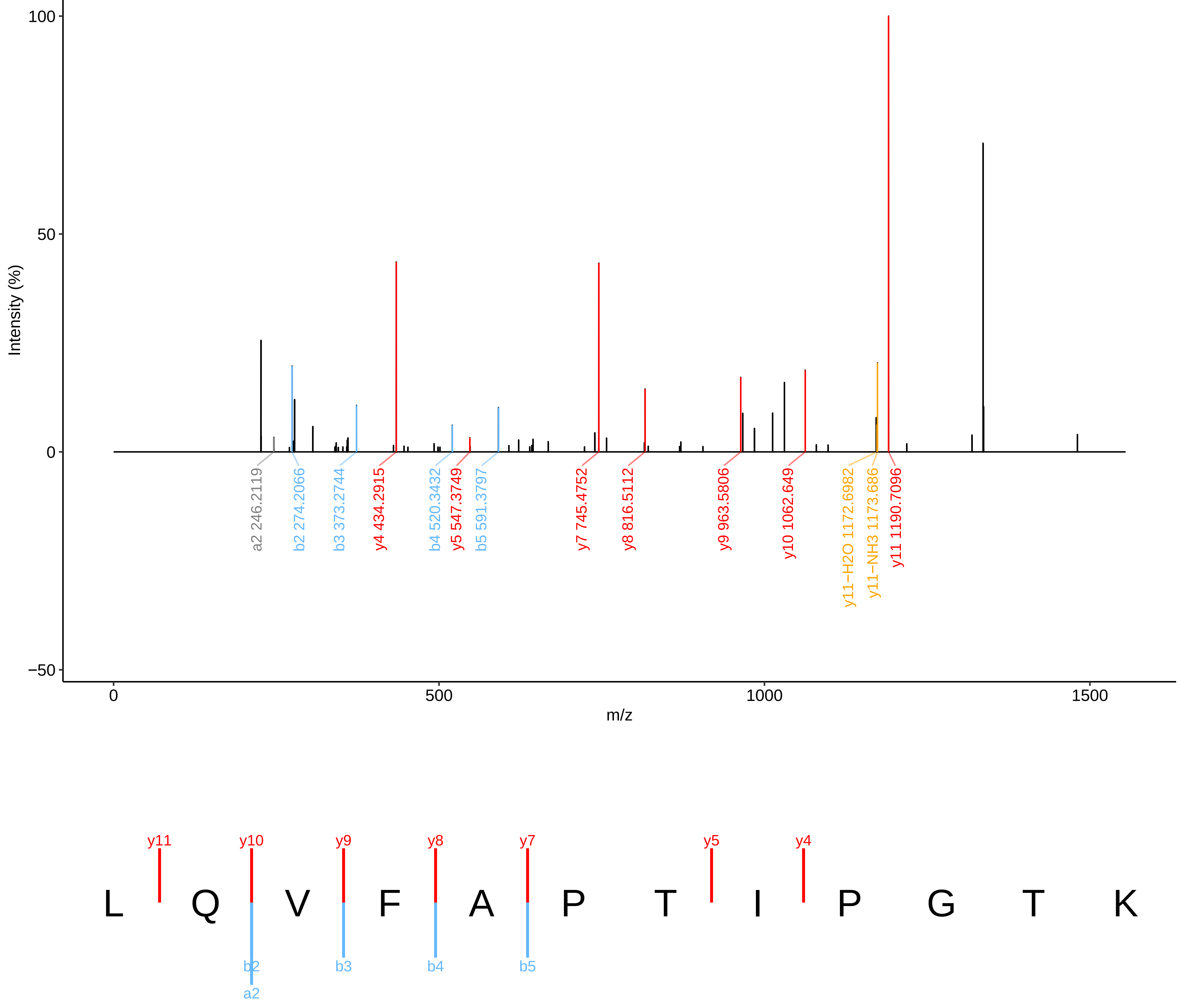

Modification □ Phospho (STY) ○ Oxidation (M) △ Acetyl (Protein N−term)

20211217_PS_SCy031_Proteome_R3_HC_F2 − Scan number: 27407

Gene Names	Charge	m/z	Mass	Mass error [Da]	Mass error [ppm]	Retention time	PEP	Score	Precursor Intensity
sll1504	2	747.8833	1493.752	0.00026366	0.36027	39.971	7.996e−10	151.15	2760398

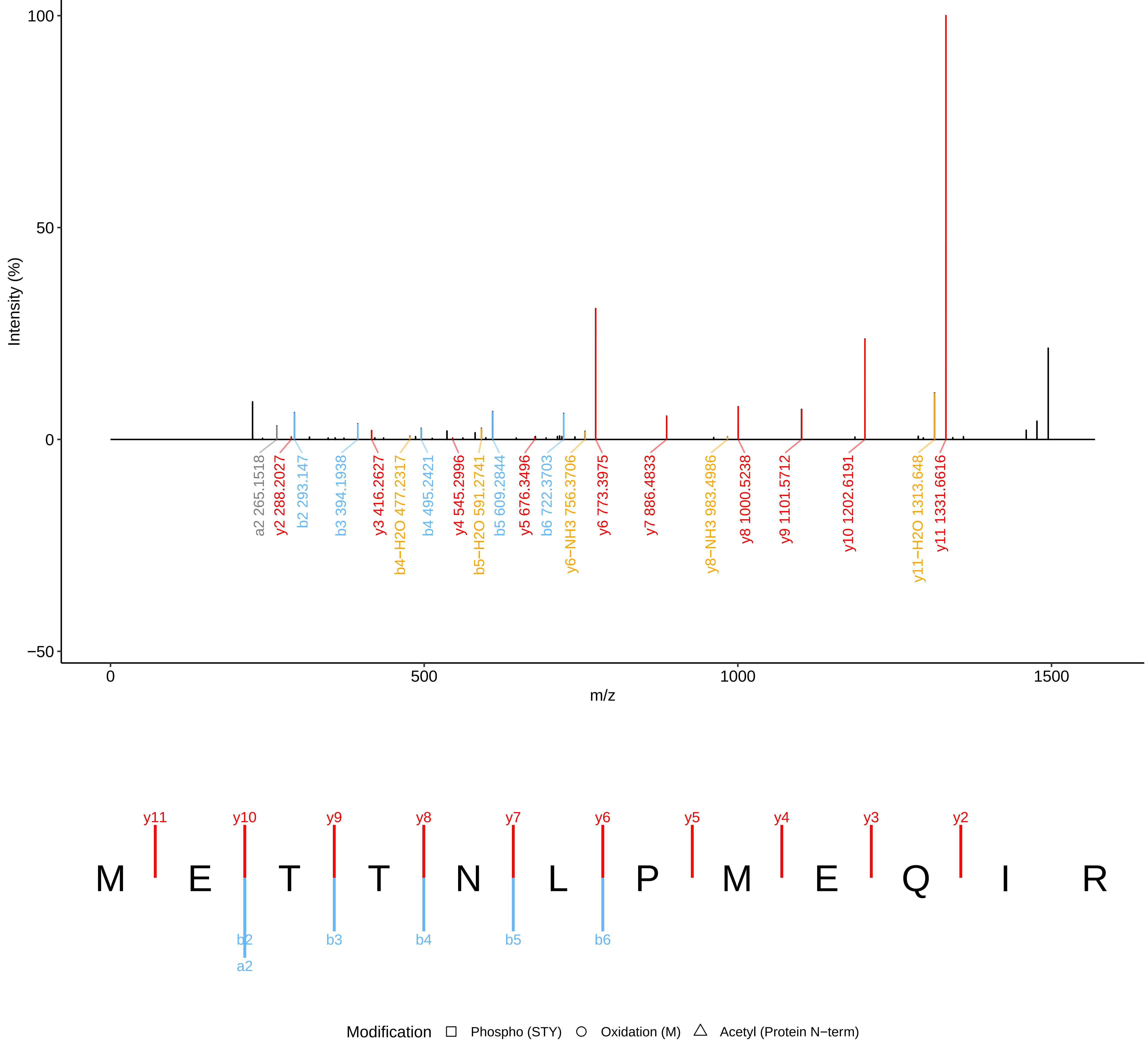

20211212_PS_SCy031_proteome_R1_3hLC_F8 − Scan number: 21486

Gene Names	Charge	m/z	Mass	Mass error [Da]	Mass error [ppm]	Retention time	PEP	Score	Precursor Intensity
sll1509	2	573.826	1145.638	0.00060159	1.0824	33.655	5.5304e−05	80.684	2957210

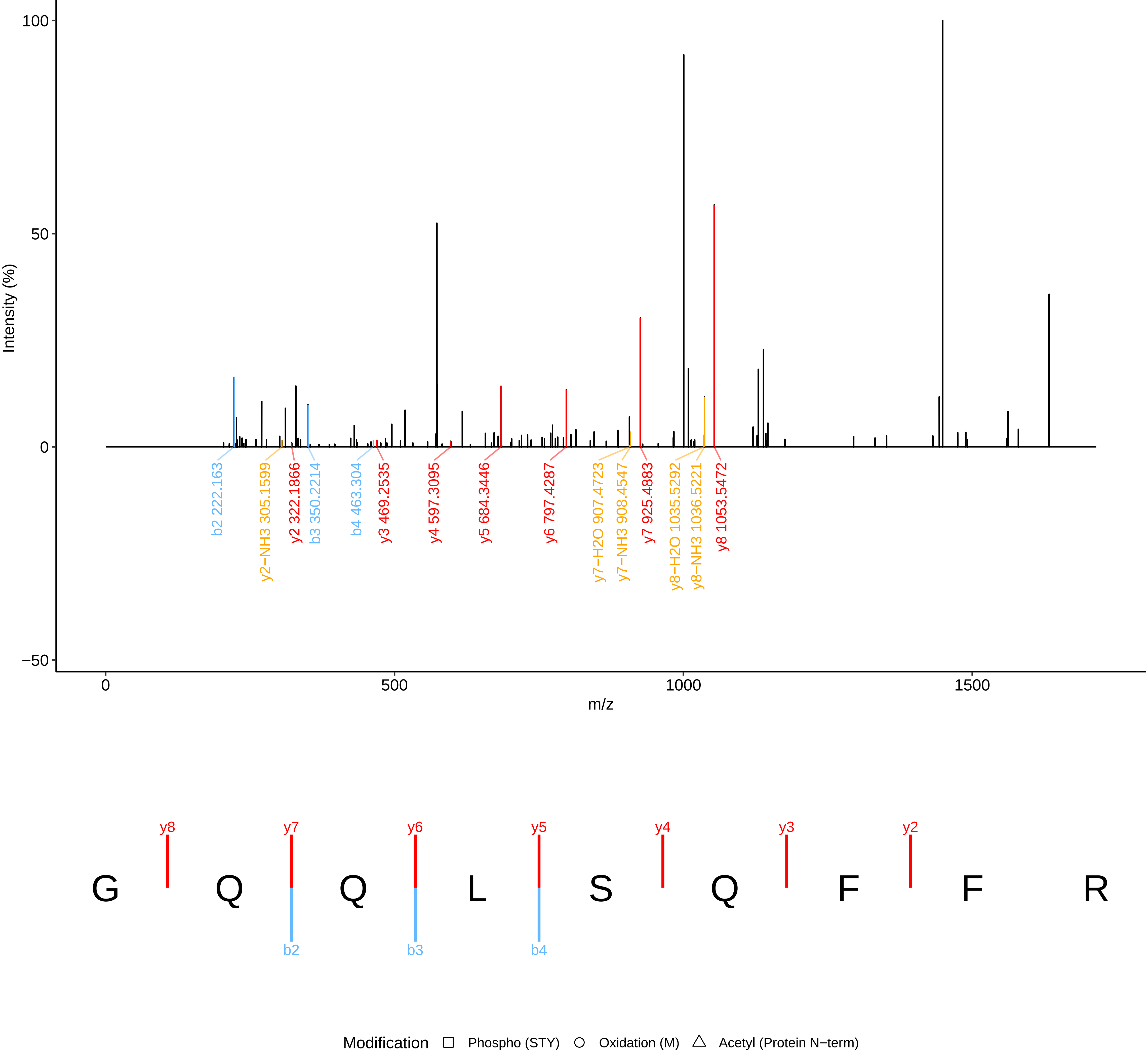

20211212_PS_SCy031_proteome_R1_24hLC_F4 − Scan number: 5163

Gene Names	Charge	m/z	Mass	Mass error [Da]	Mass error [ppm]	Retention time	PEP	Score	Precursor Intensity
sll1572	3	407.9345	1220.782	0.00047152	1.2283	11.225	4.3369e−06	85.958	2408889

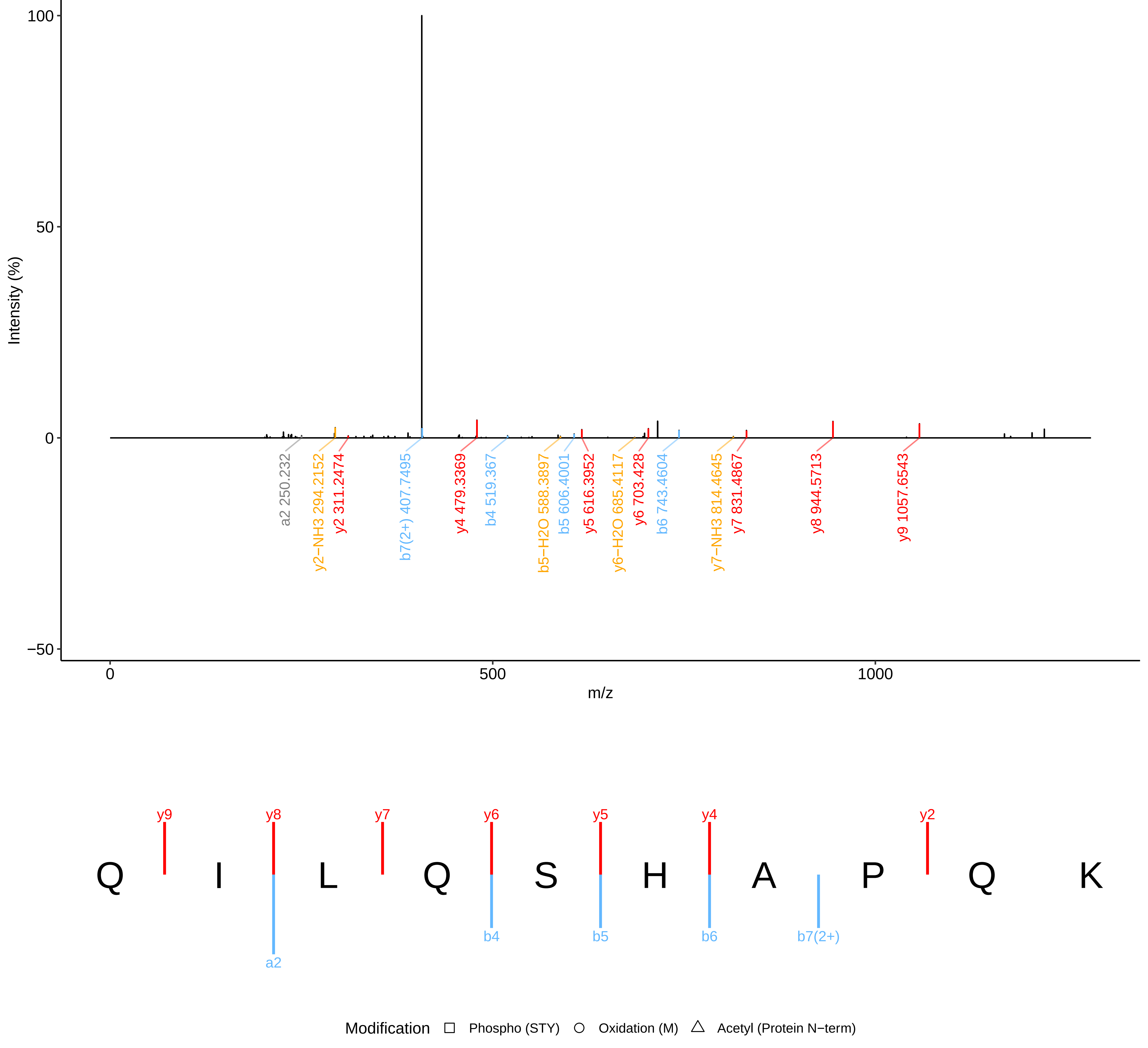

20211217_PS_SCy031_Proteome_R2_HC_F7 − Scan number: 5086

Gene Names	Charge	m/z	Mass	Mass error [Da]	Mass error [ppm]	Retention time	PEP	Score	Precursor Intensity
sll1639	3	320.4997	958.4773	0.00010716	0.34441	11.35	0.00010224	86.113	2854253

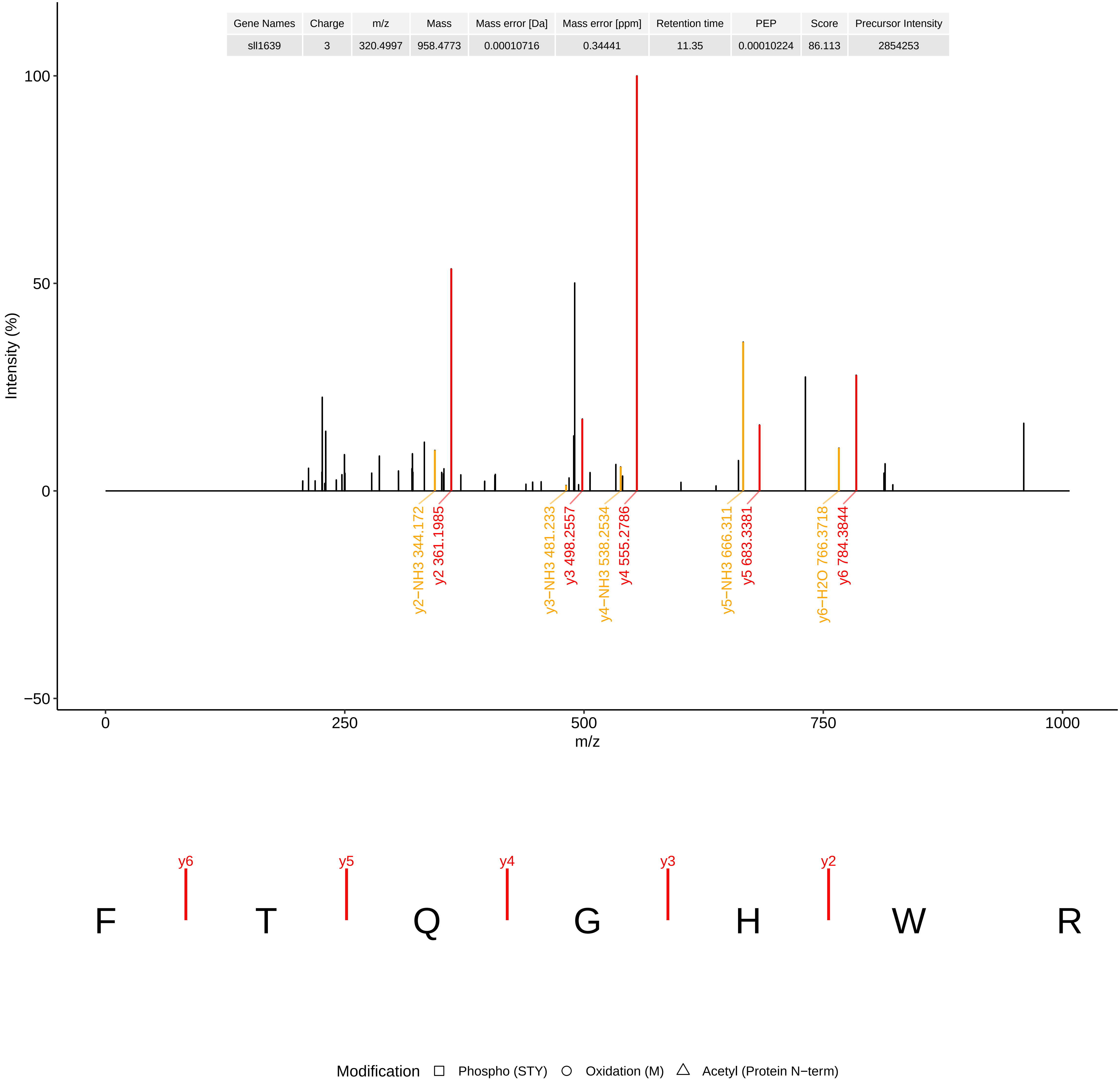

20211217_PS_SCy031_Proteome_R2_24hLC_F9 − Scan number: 15117

Gene Names	Charge	m/z	Mass	Mass error [Da]	Mass error [ppm]	Retention time	PEP	Score	Precursor Intensity
sll1671	3	478.5965	1432.768	0.00021996	0.47145	27.224	0.00027133	54.65	2277042

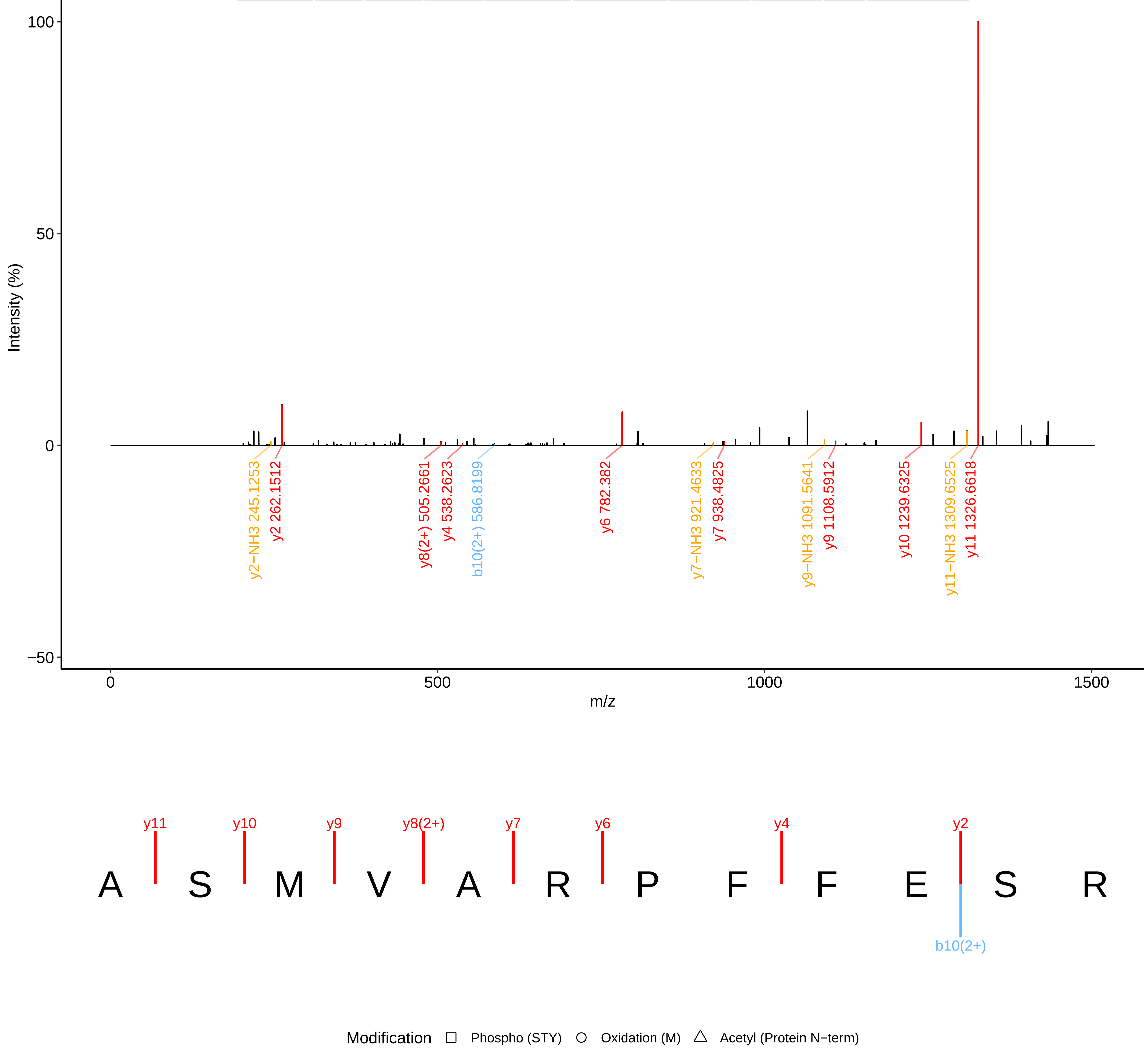

20211217_PS_SCy031_Proteome_R3_24hLC_F7 − Scan number: 24455

Gene Names	Charge	m/z	Mass	Mass error [Da]	Mass error [ppm]	Retention time	PEP	Score	Precursor Intensity
sll1732	2	496.2842	990.5539	−1.6448e−07	−0.00035126	40.085	0.00044346	71.342	7913359

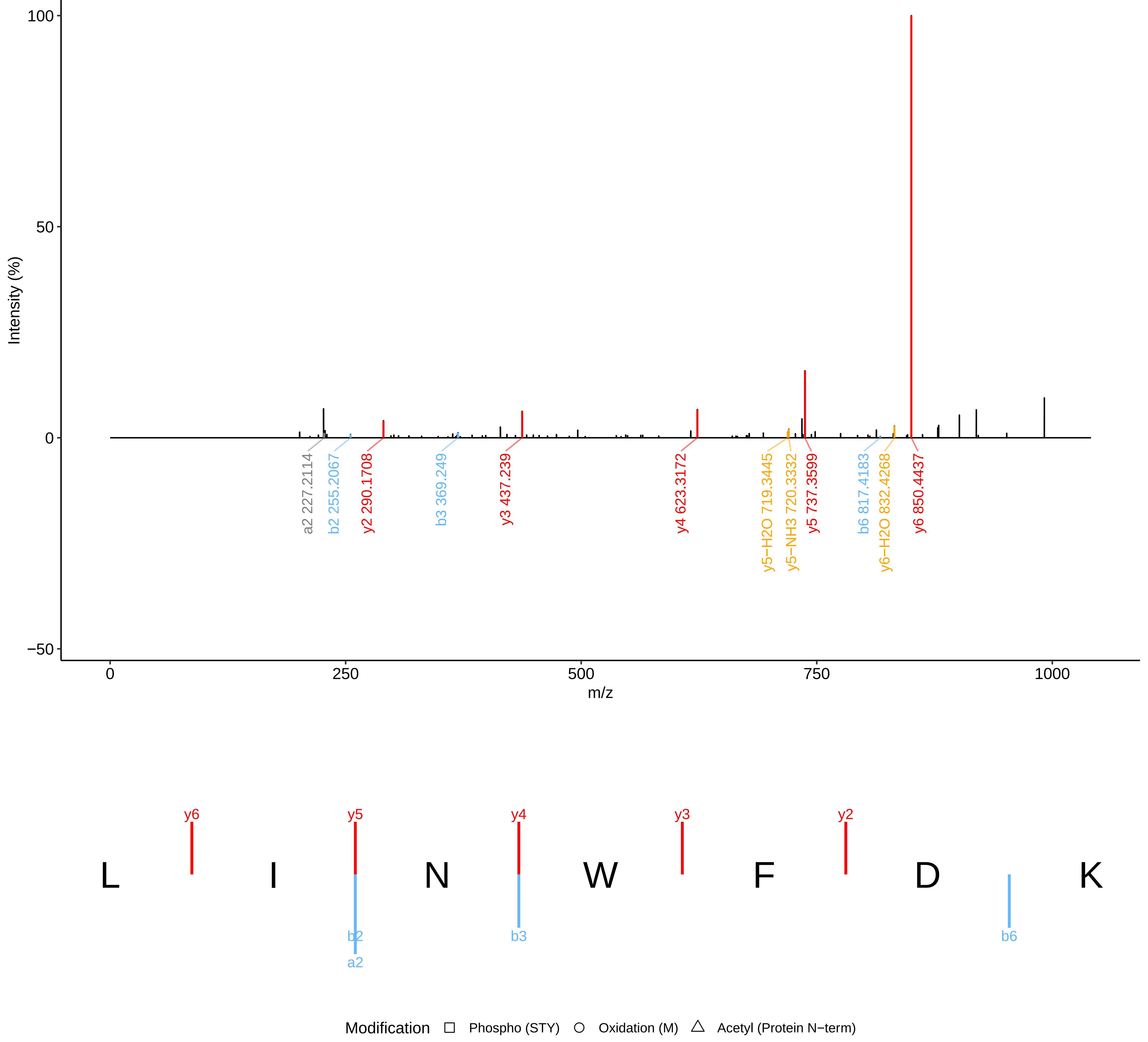

20211212_PS_SCy031_proteome_R1_3hLC_F6 − Scan number: 35565

Gene Names	Charge	m/z	Mass	Mass error [Da]	Mass error [ppm]	Retention time	PEP	Score	Precursor Intensity
sll1733	2	753.4305	1504.847	−0.00091099	−1.2081	52.179	1.9258e−38	211.64	11752030

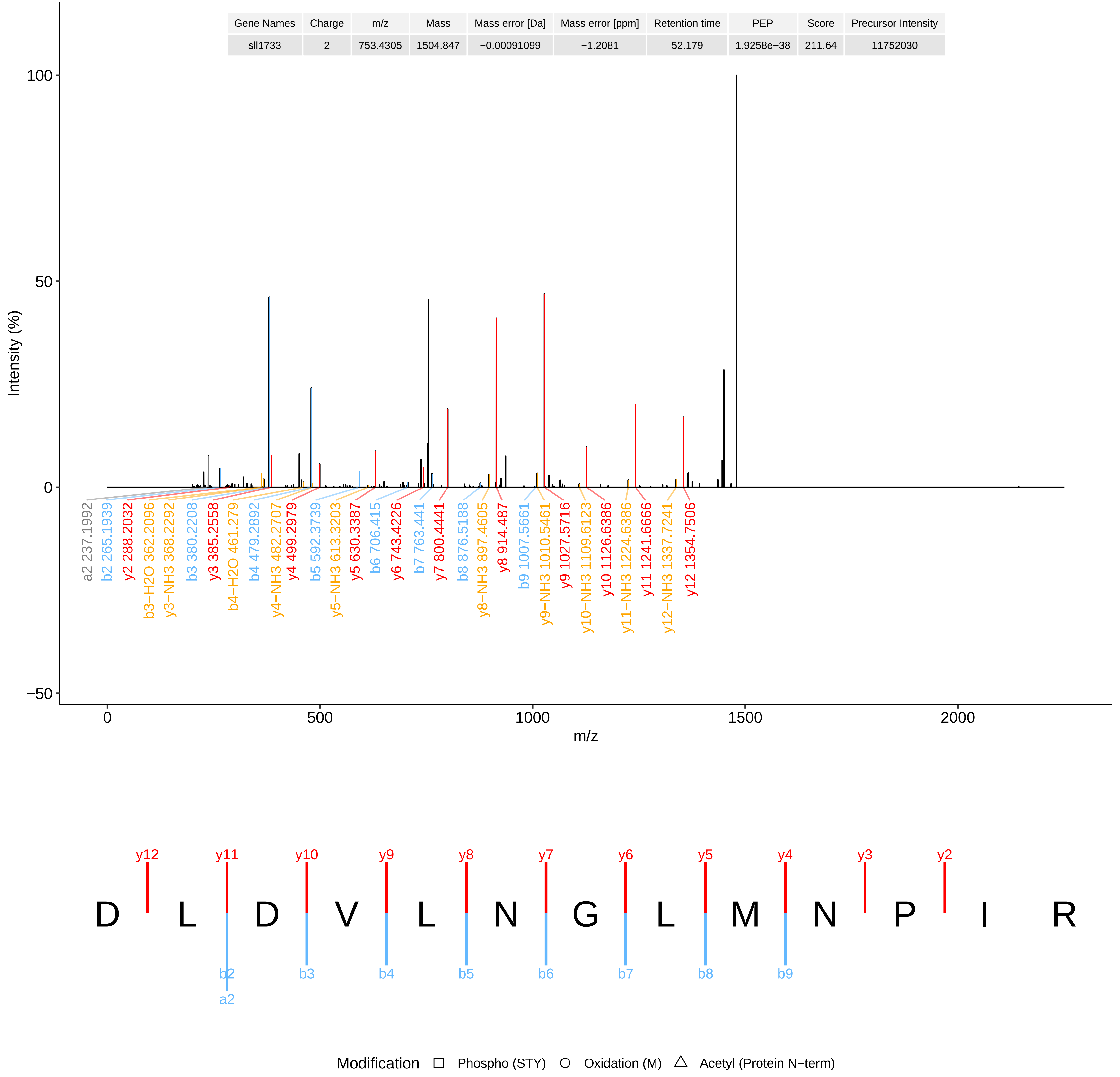

20211217_PS_SCy031_Proteome_R2_HC_F5 − Scan number: 7465

Gene Names	Charge	m/z	Mass	Mass error [Da]	Mass error [ppm]	Retention time	PEP	Score	Precursor Intensity
sll1738	3	492.9392	1475.796	0.0005008	1.0153	14.907	0.00058331	73.273	1376146

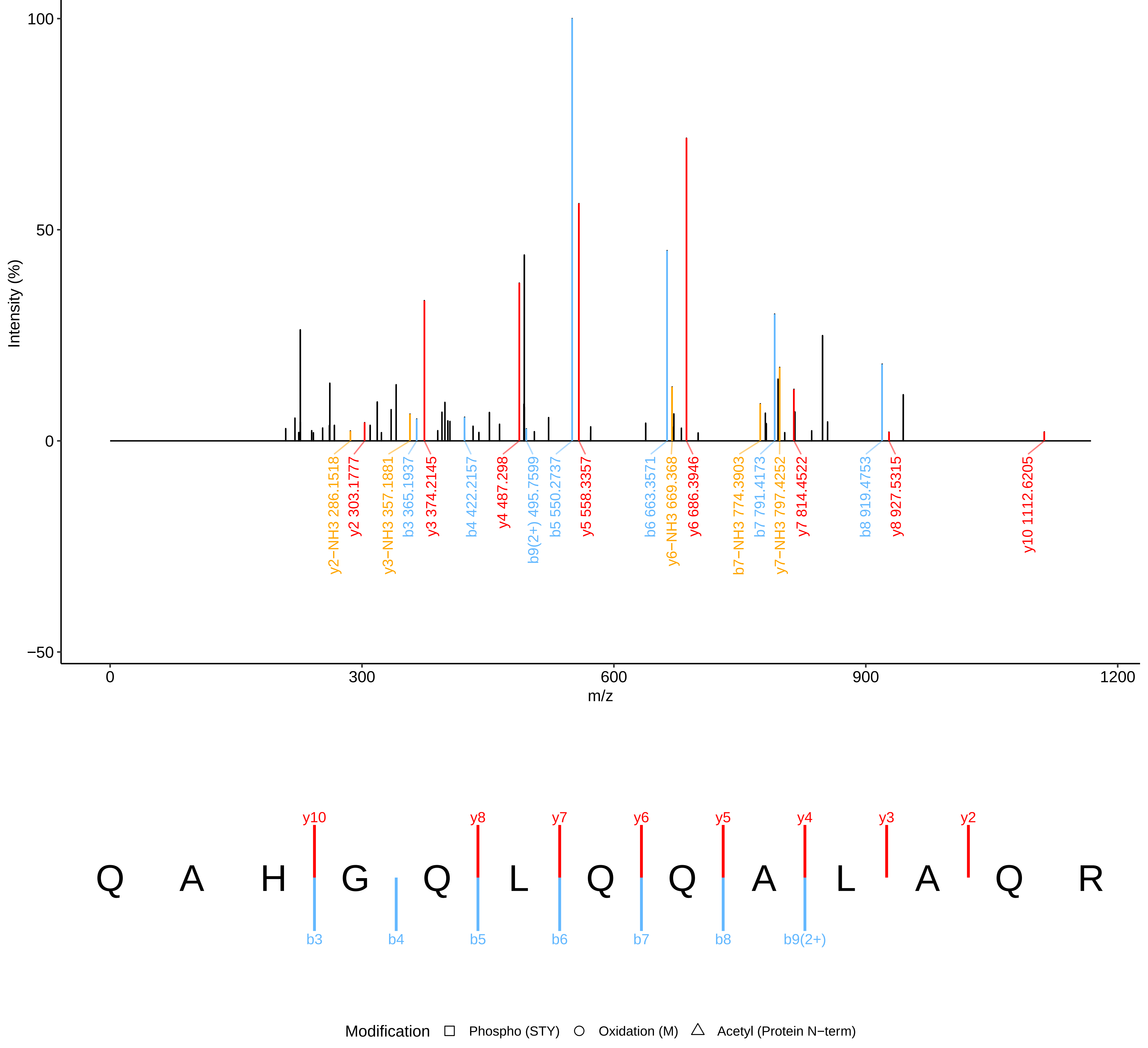

20211212_PS_SCy031_proteome_R1_HC_F7 − Scan number: 10358

Gene Names	Charge	m/z	Mass	Mass error [Da]	Mass error [ppm]	Retention time	PEP	Score	Precursor Intensity
sll1751	2	416.7556	831.4967	−2.3189e−05	−0.057578	18.166	9.9742e−05	121.68	NA

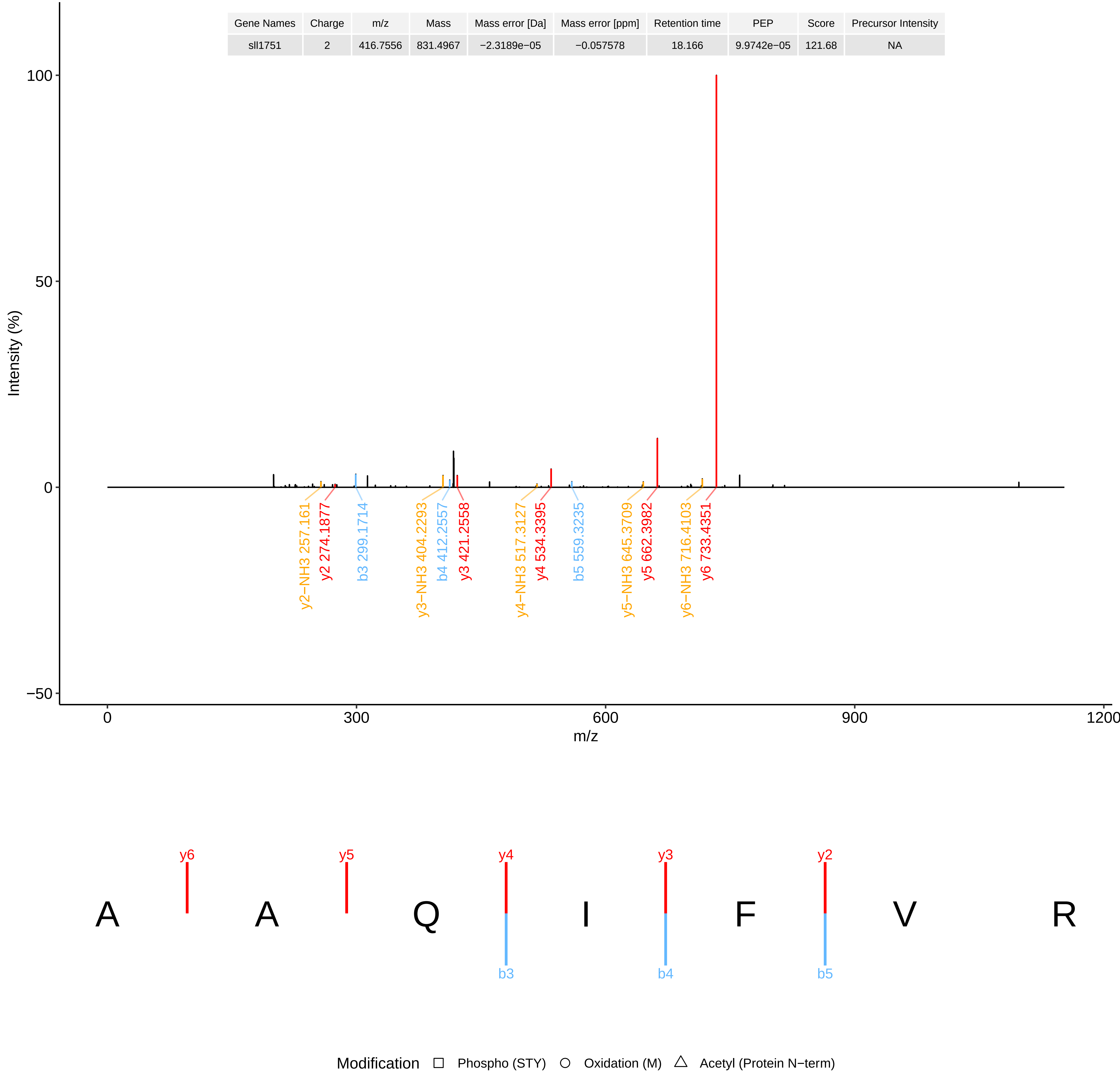

20211217_PS_SCy031_Proteome_R3_HC_F6 − Scan number: 13078

Gene Names	Charge	m/z	Mass	Mass error [Da]	Mass error [ppm]	Retention time	PEP	Score	Precursor Intensity
sll1764	2	636.3569	1270.699	−0.00044486	−0.71482	23.259	1.5552e−05	109.29	5836202

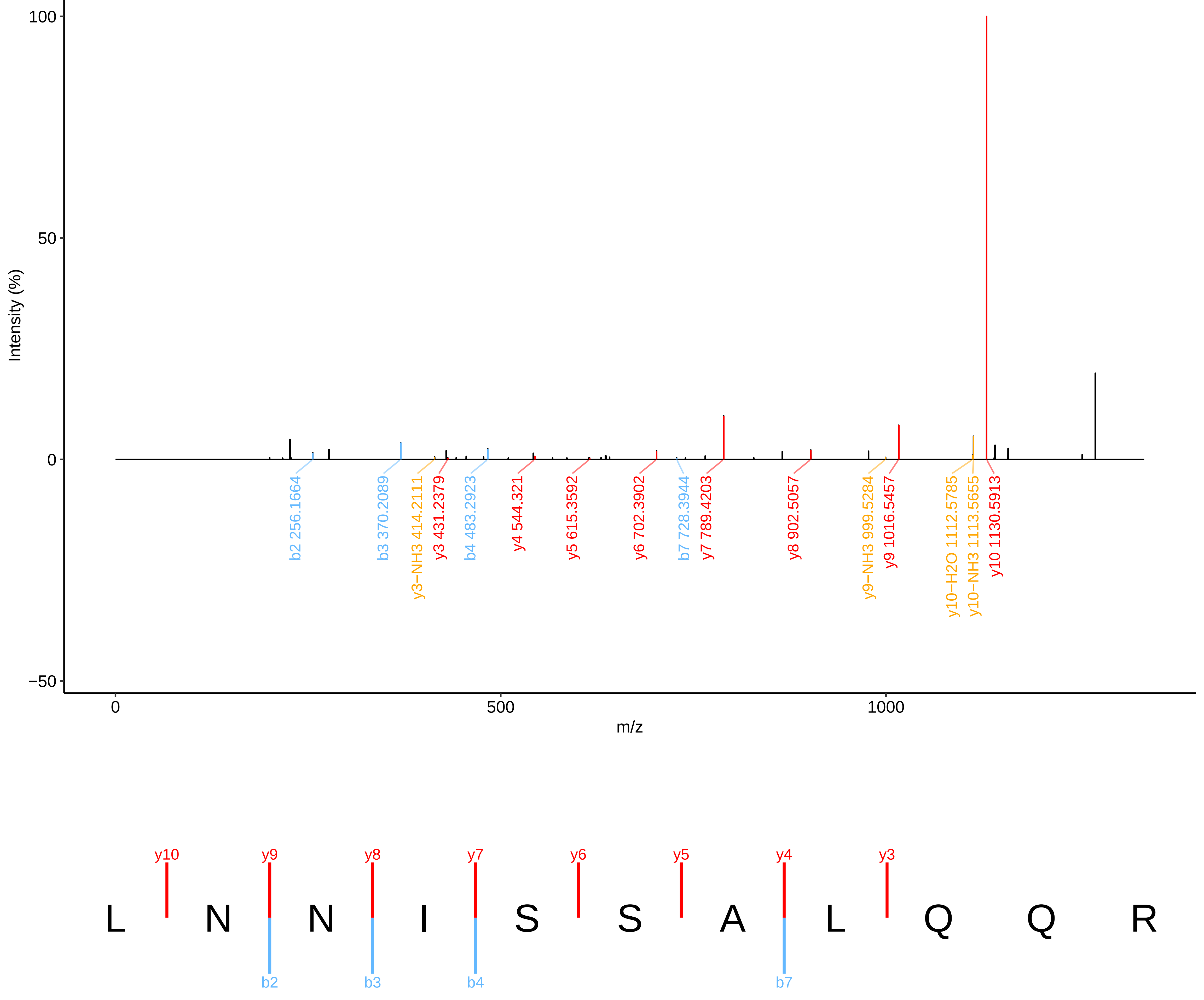

Modification □ Phospho (STY) ○ Oxidation (M) △ Acetyl (Protein N−term)

20211217_PS_SCy031_Proteome_R2_24hLC_F2 − Scan number: 9607

Gene Names	Charge	m/z	Mass	Mass error [Da]	Mass error [ppm]	Retention time	PEP	Score	Precursor Intensity
sll1862	2	754.8979	1507.781	0.00010876	0.1476	16.431	2.7631e−97	258.48	7613172

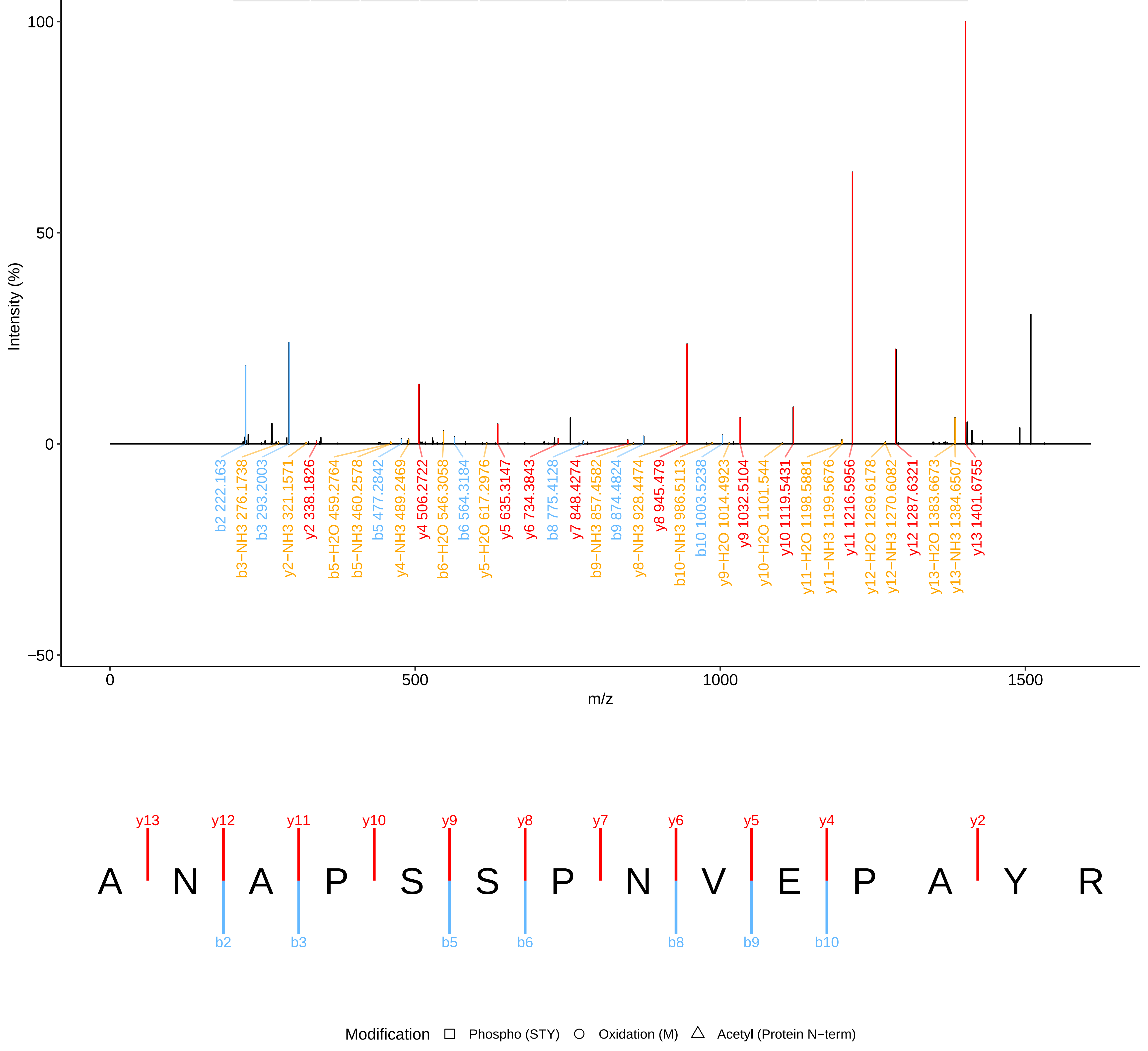

20211212_PS_SCy031_proteome_R1_3hLC_F2 − Scan number: 10412

Gene Names	Charge	m/z	Mass	Mass error [Da]	Mass error [ppm]	Retention time	PEP	Score	Precursor Intensity
sll1874	2	431.2707	860.5269	8.7821e−05	0.21149	17.333	1.5507e−06	138.37	38422176

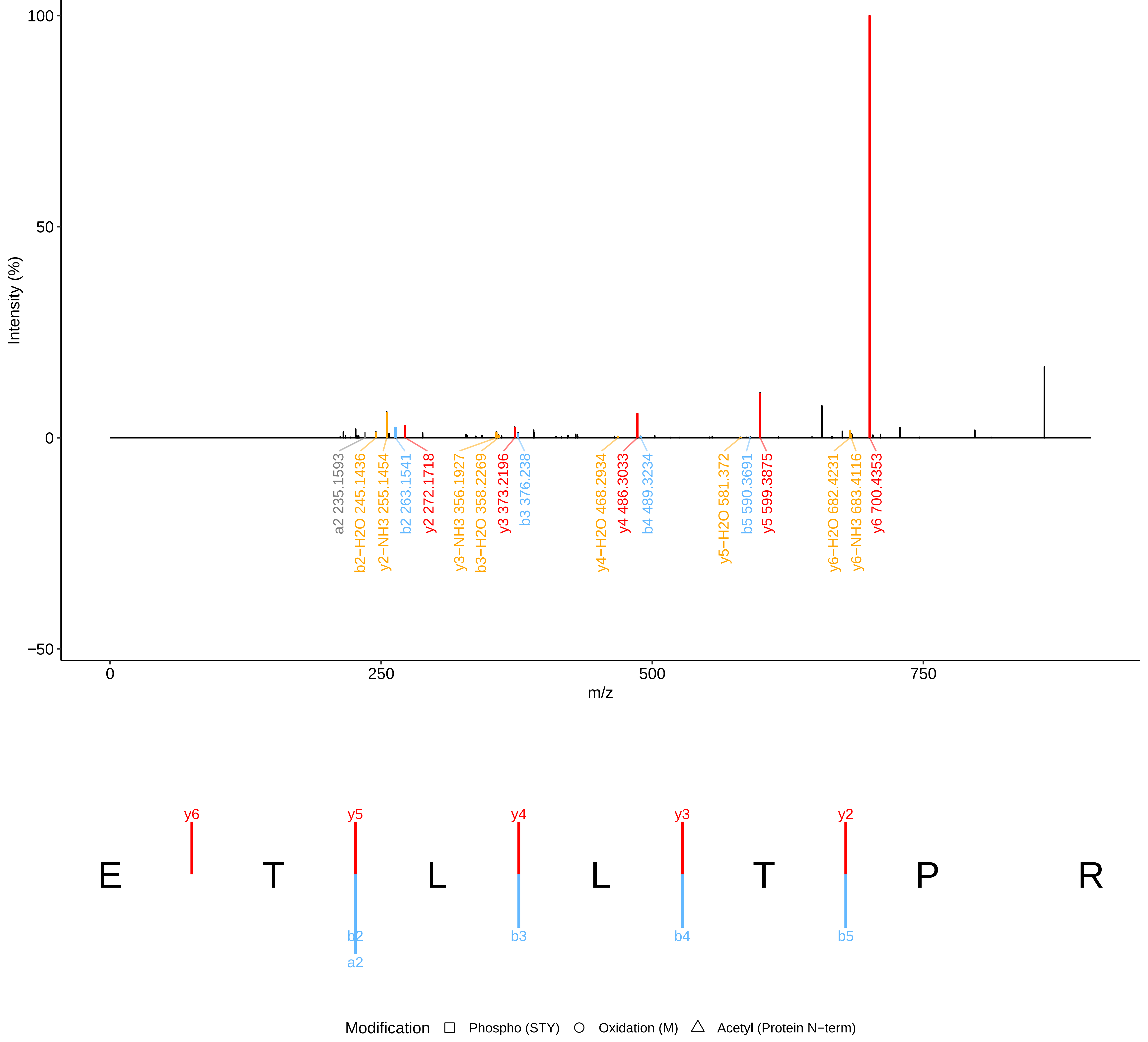

20211212_PS_SCy031_proteome_R1_3hLC_F8 − Scan number: 9795

Gene Names	Charge	m/z	Mass	Mass error [Da]	Mass error [ppm]	Retention time	PEP	Score	Precursor Intensity
sll1890	3	487.9544	1460.841	2.8104e−05	0.060235	17.909	0.00017176	57.019	824323.7

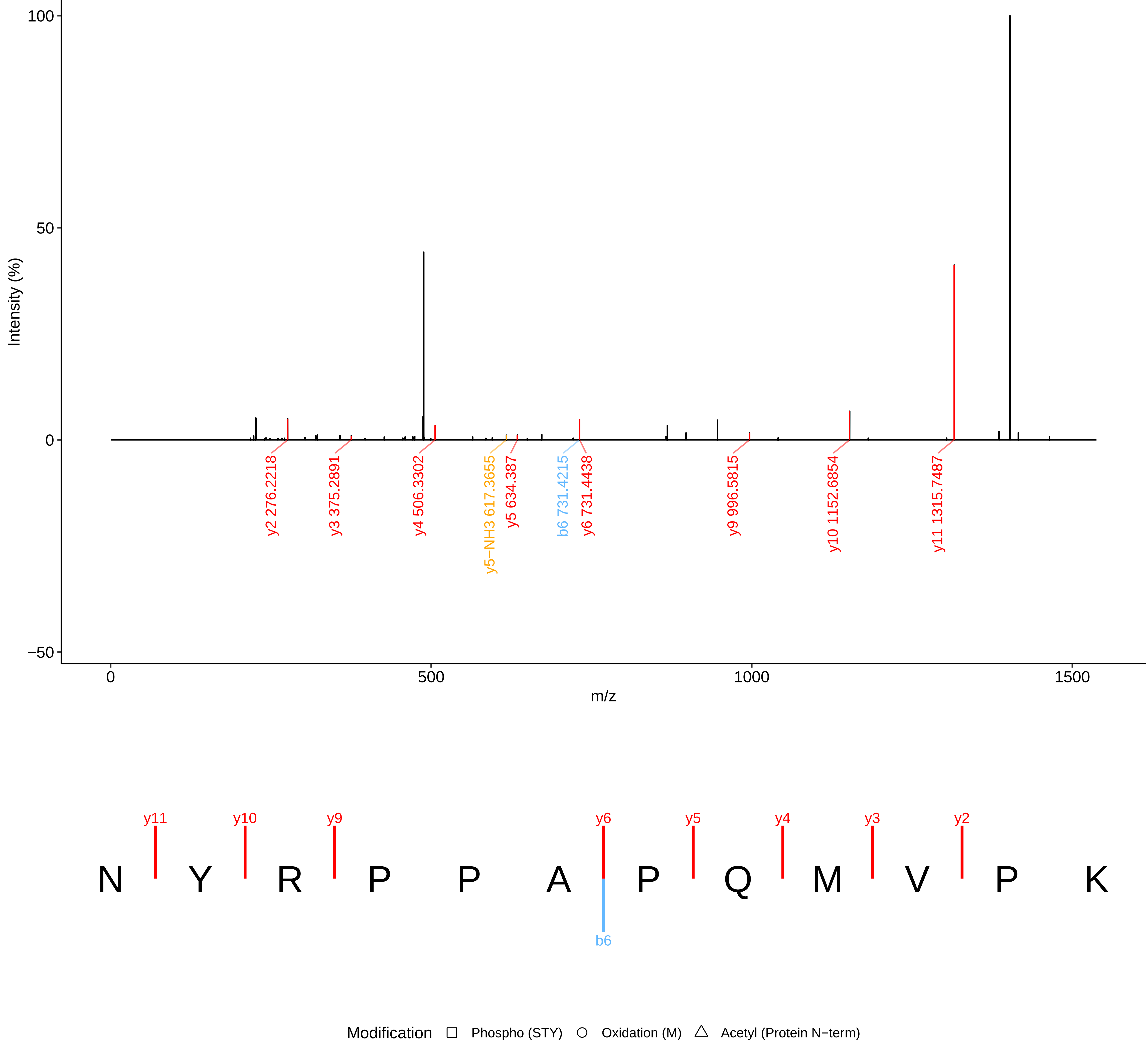

20211212_PS_SCy031_proteome_R1_3hLC_F9 − Scan number: 32626

Gene Names	Charge	m/z	Mass	Mass error [Da]	Mass error [ppm]	Retention time	PEP	Score	Precursor Intensity
sll1969	3	839.7969	2516.369	7.7797e−05	0.093832	47.862	4.4569e−50	157.73	3386101

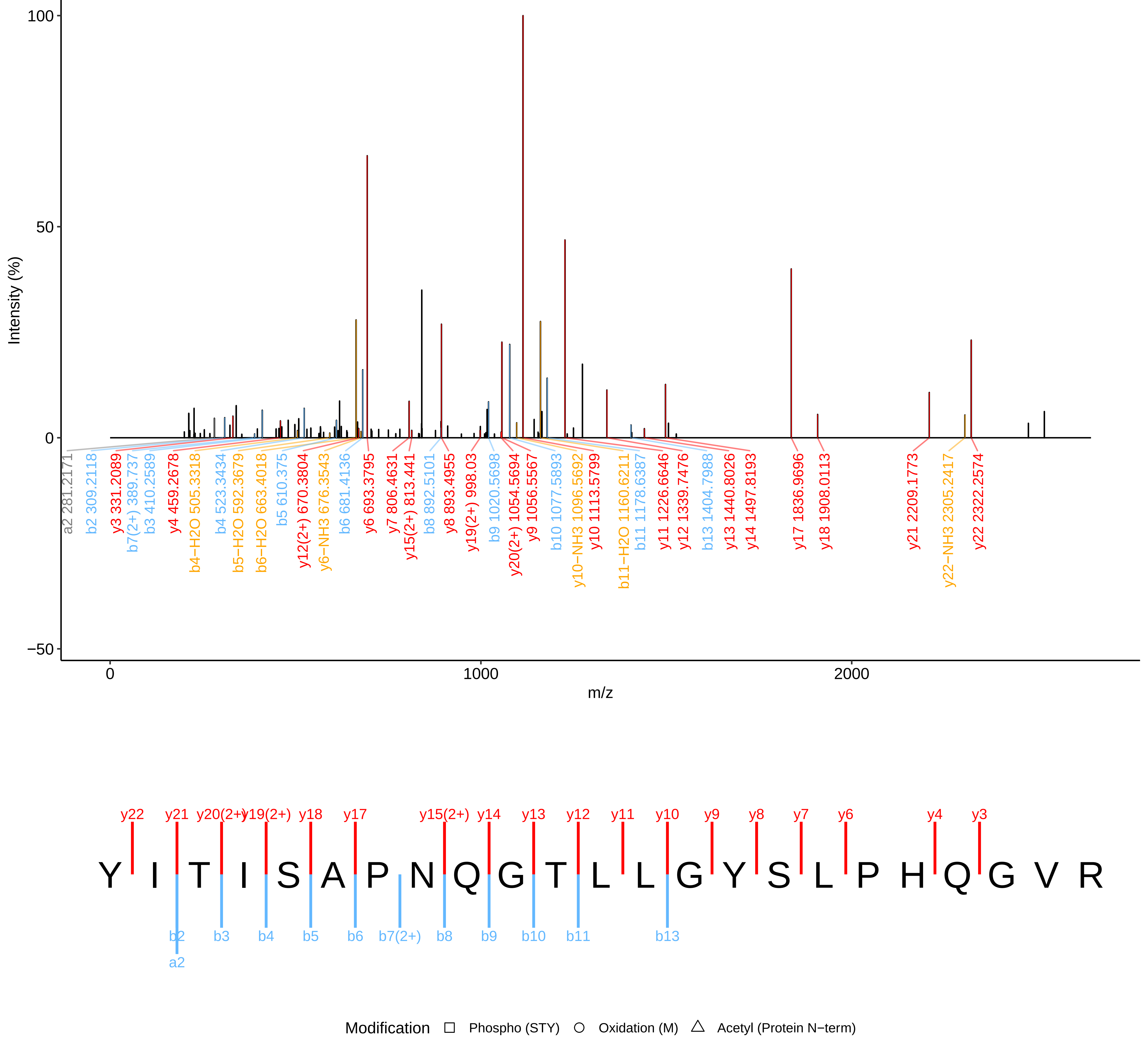

20211212_PS_SCy031_proteome_R1_HC_F7 − Scan number: 34403

Gene Names	Charge	m/z	Mass	Mass error [Da]	Mass error [ppm]	Retention time	PEP	Score	Precursor Intensity
sll2006	3	596.9859	1787.936	−0.0012272	−2.0557	49.879	2.1789e−15	180.09	32209674

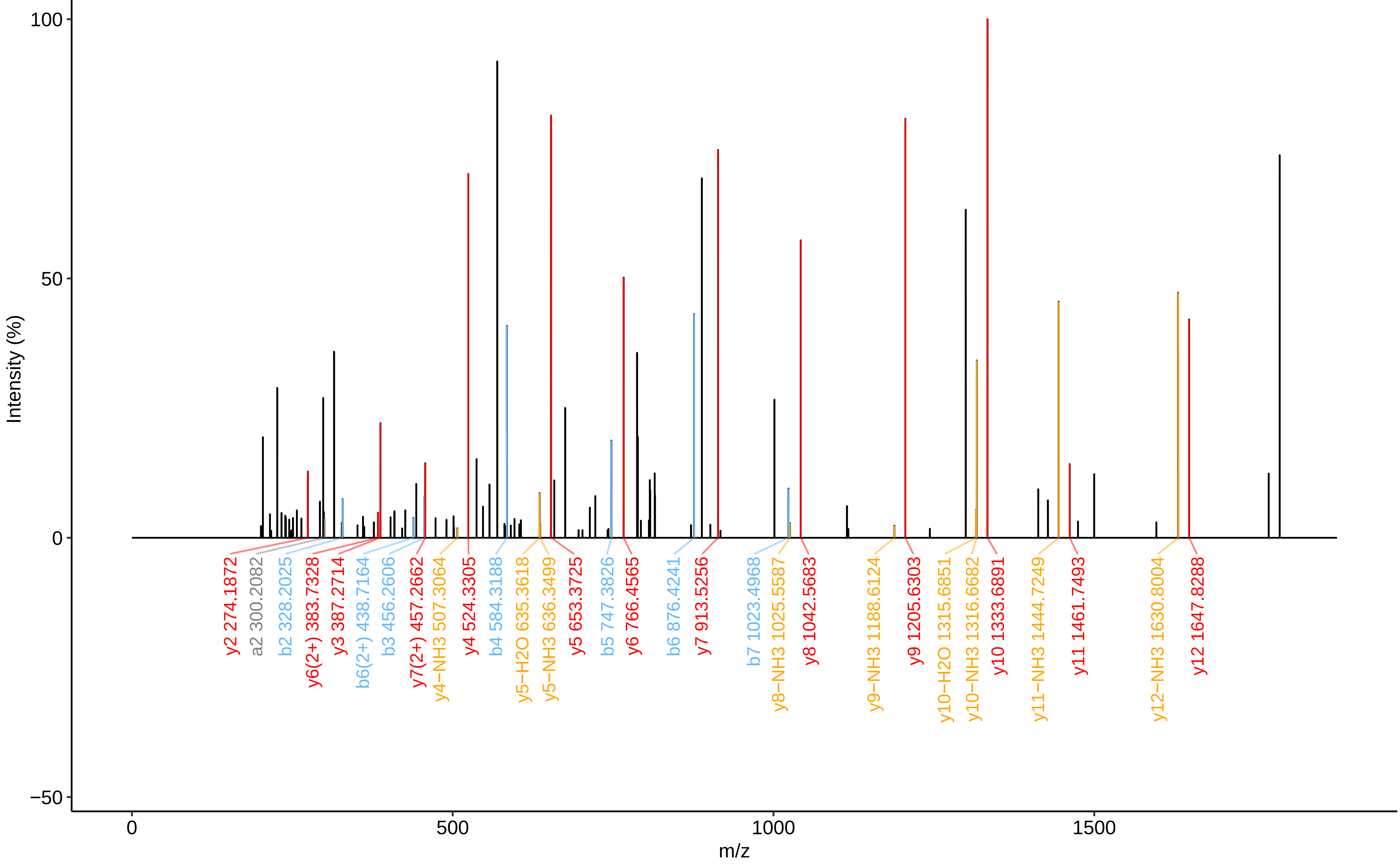

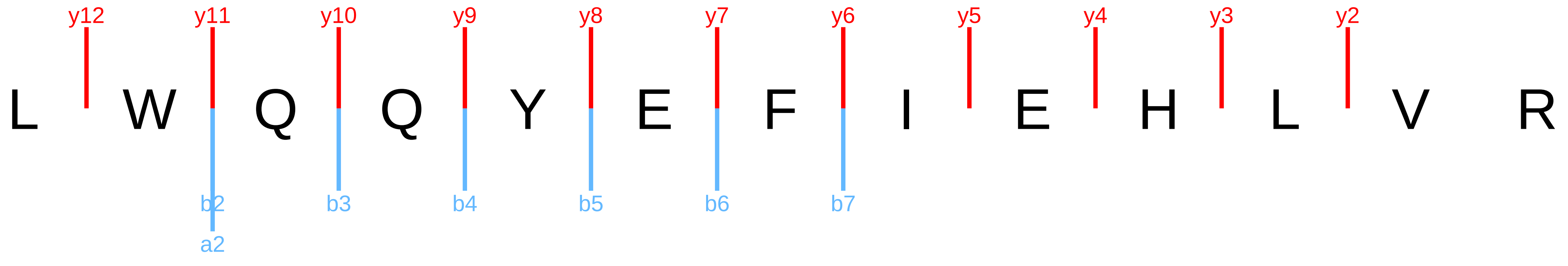

Modification □ Phospho (STY) ○ Oxidation (M) △ Acetyl (Protein N−term)

20211212_PS_SCy031_proteome_R1_24hLC_F5 − Scan number: 17236

Gene Names	Charge	m/z	Mass	Mass error [Da]	Mass error [ppm]	Retention time	PEP	Score	Precursor Intensity
sll7065	2	906.4188	1810.823	NA	NA	26.791	0.00039401	80.688	6814258

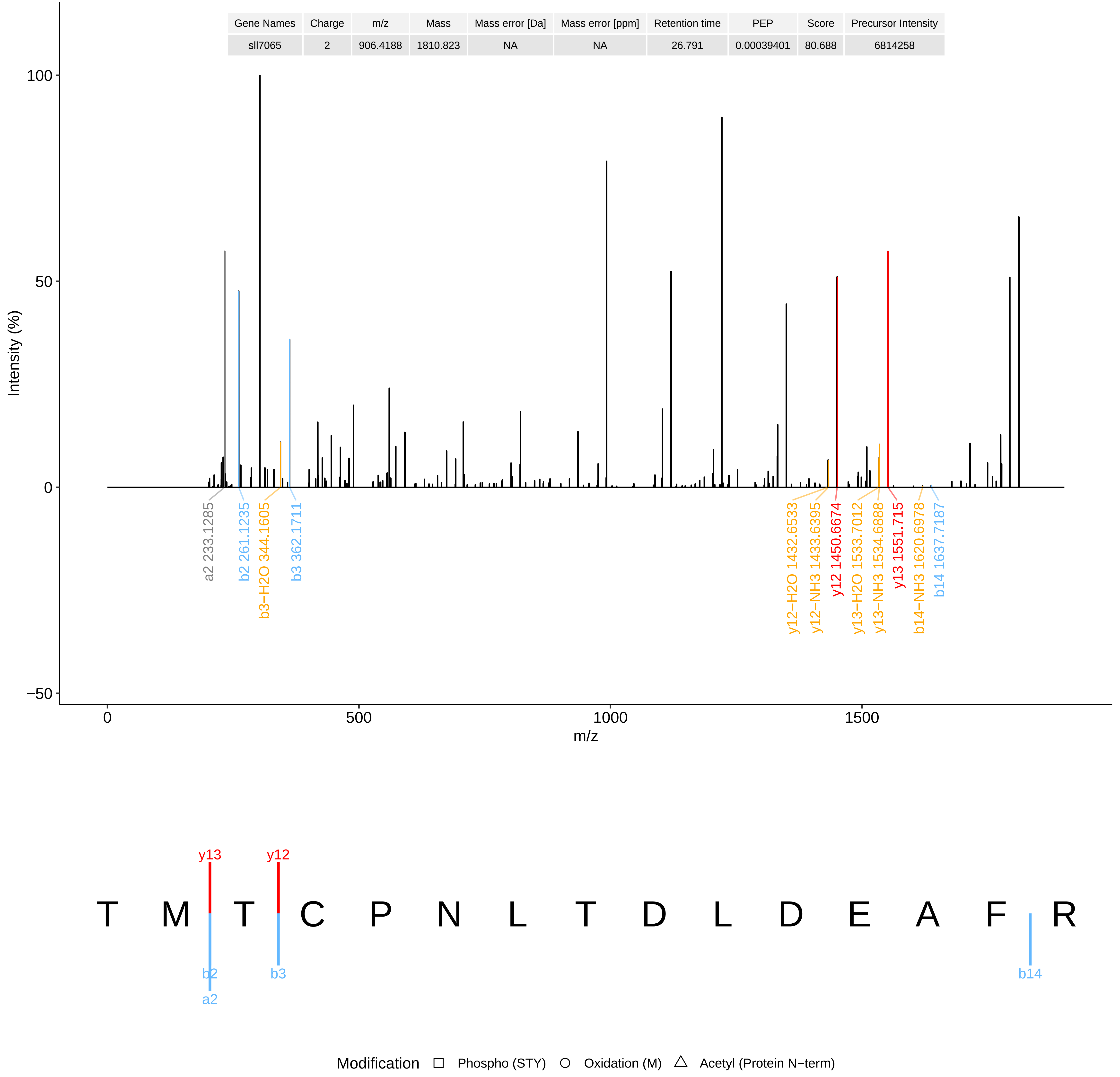

20211212_PS_SCy031_proteome_R1_HC_F3 − Scan number: 4459

Gene Names	Charge	m/z	Mass	Mass error [Da]	Mass error [ppm]	Retention time	PEP	Score	Precursor Intensity
slr0023	2	398.7455	795.4764	2.4183e−05	0.065948	9.879	0.00037878	74.439	4685772

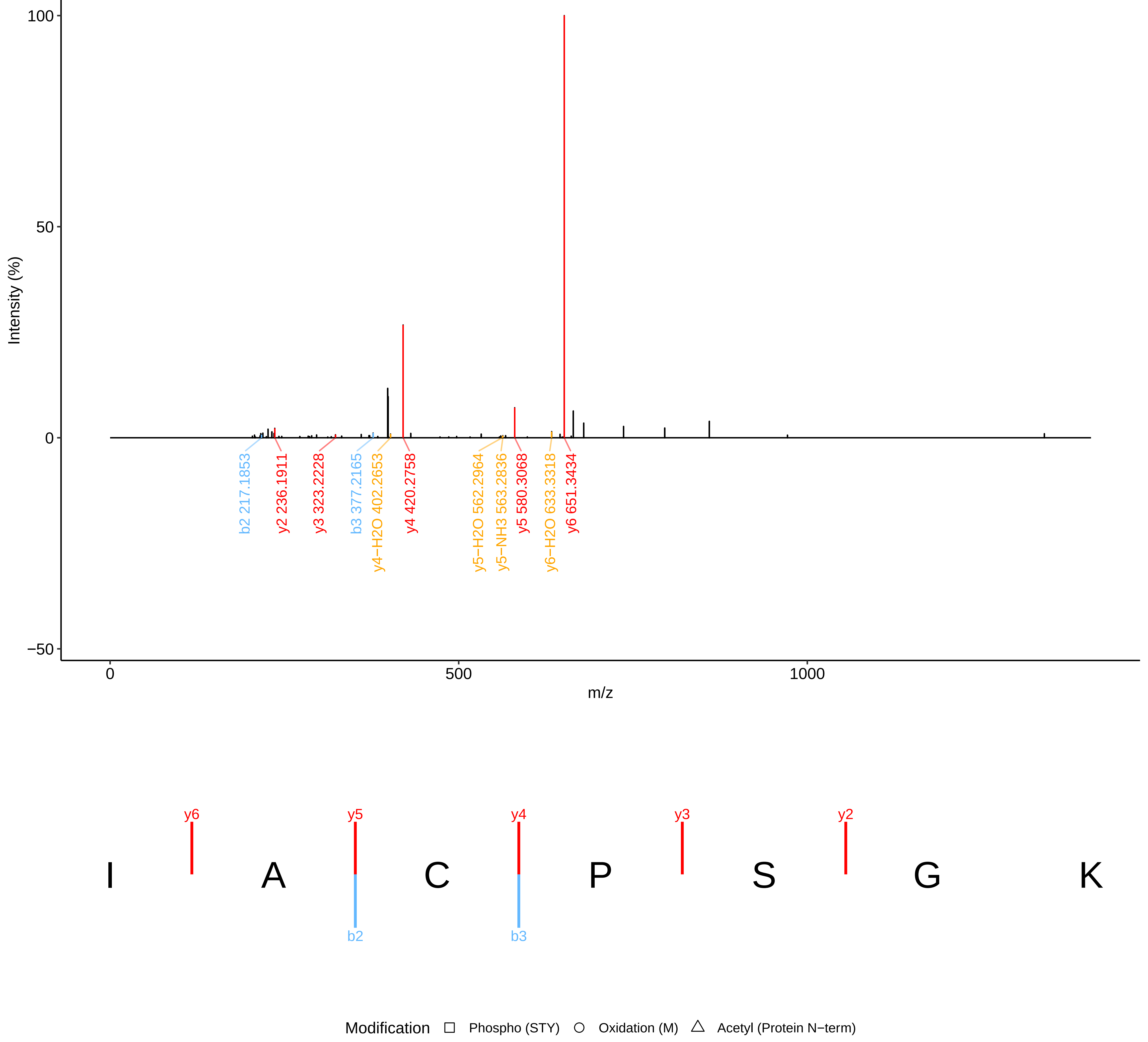

20211217_PS_SCy031_Proteome_R3_24hLC_F3 − Scan number: 3933

Gene Names	Charge	m/z	Mass	Mass error [Da]	Mass error [ppm]	Retention time	PEP	Score	Precursor Intensity
slr0053	3	324.8973	971.6702	3.4134e−05	0.11346	9.3089	0.00024123	70.912	2888123

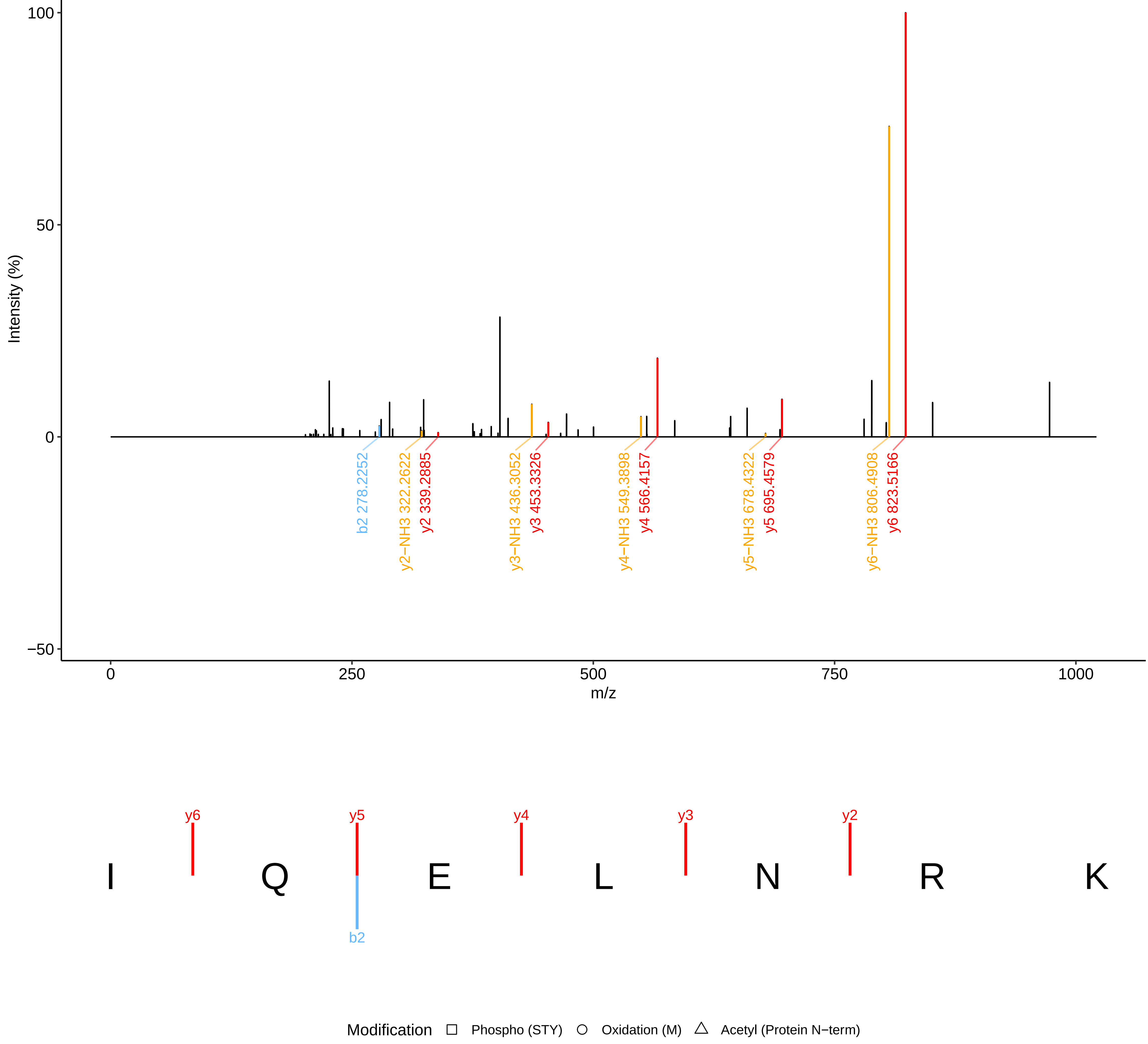

20211217_PS_SCy031_Proteome_R2_24hLC_F7 − Scan number: 21410

Gene Names	Charge	m/z	Mass	Mass error [Da]	Mass error [ppm]	Retention time	PEP	Score	Precursor Intensity
slr0254	3	420.5933	1258.758	0.00010291	0.25188	33.58	4.0158e−06	86.624	1923068

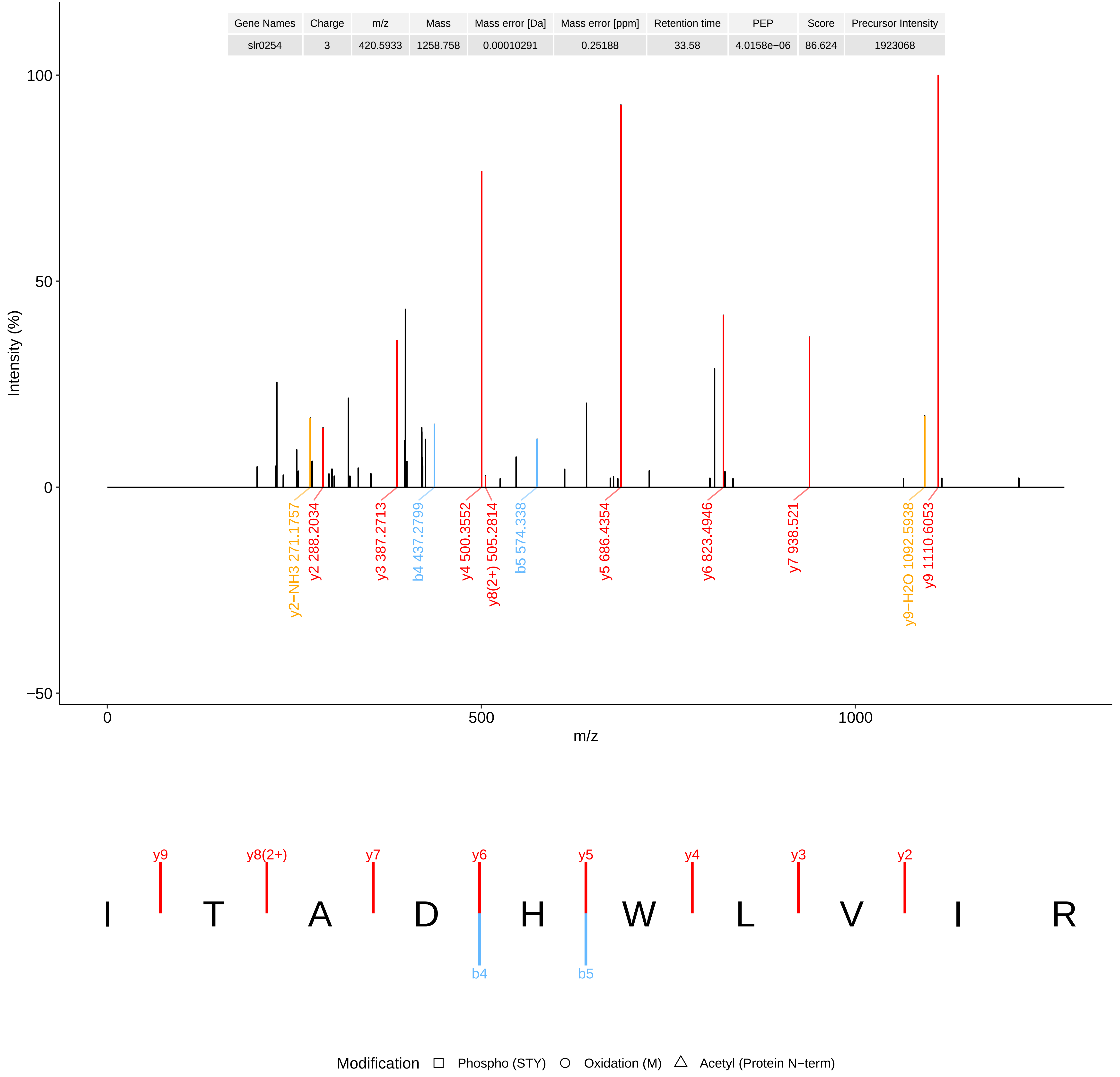

20211217_PS_SCy031_Proteome_R3_3hLC_F6 − Scan number: 16532

Gene Names	Charge	m/z	Mass	Mass error [Da]	Mass error [ppm]	Retention time	PEP	Score	Precursor Intensity
slr0271	2	596.8199	1191.625	NA	NA	27.979	0.00033812	84.244	2474002

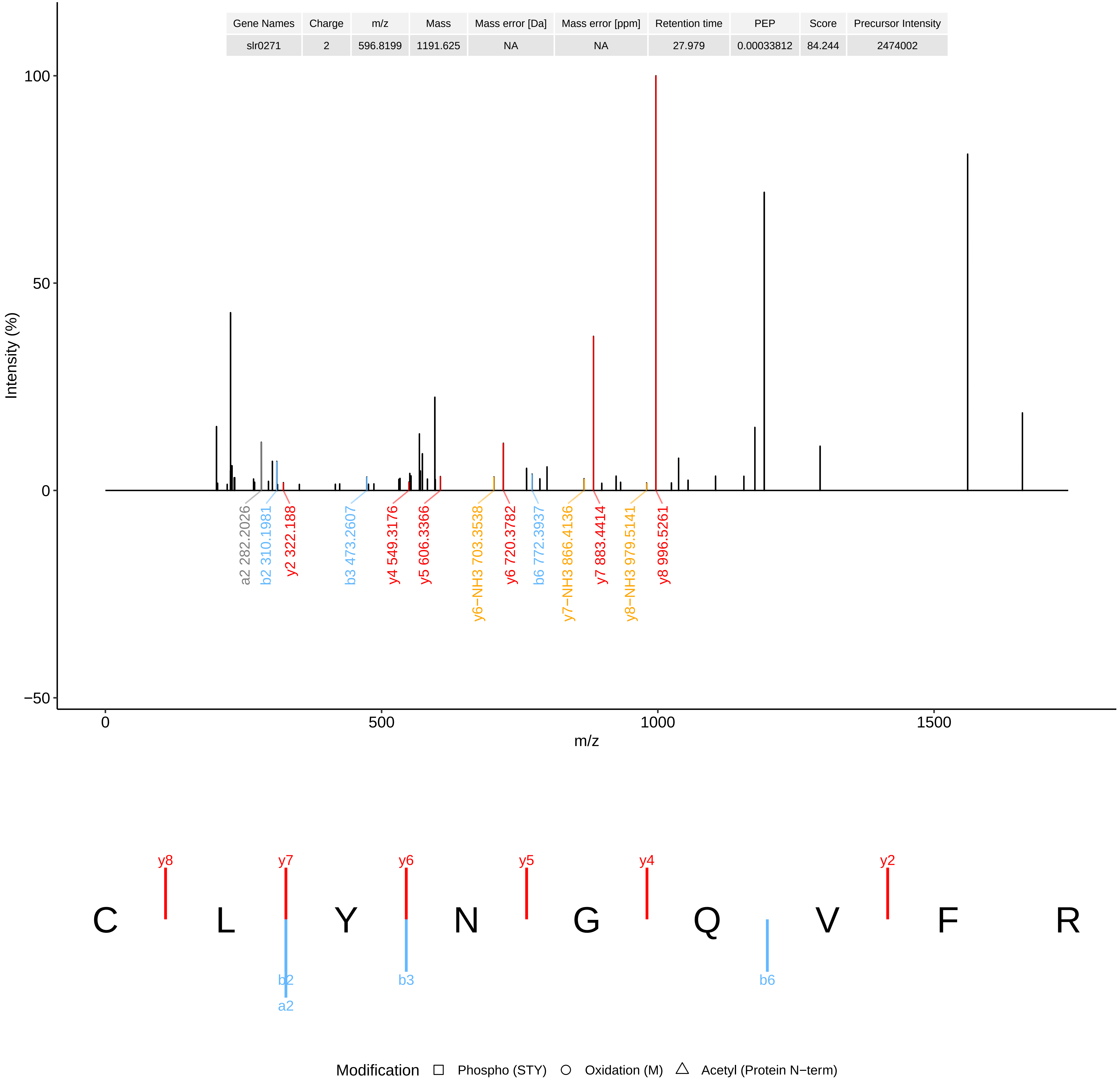

20211212_PS_SCy031_proteome_R1_HC_F8 − Scan number: 10923

Gene Names	Charge	m/z	Mass	Mass error [Da]	Mass error [ppm]	Retention time	PEP	Score	Precursor Intensity
slr0299	3	384.5451	1150.613	−4.3957e−05	−0.11716	20.036	8.7179e−09	126.07	8825350

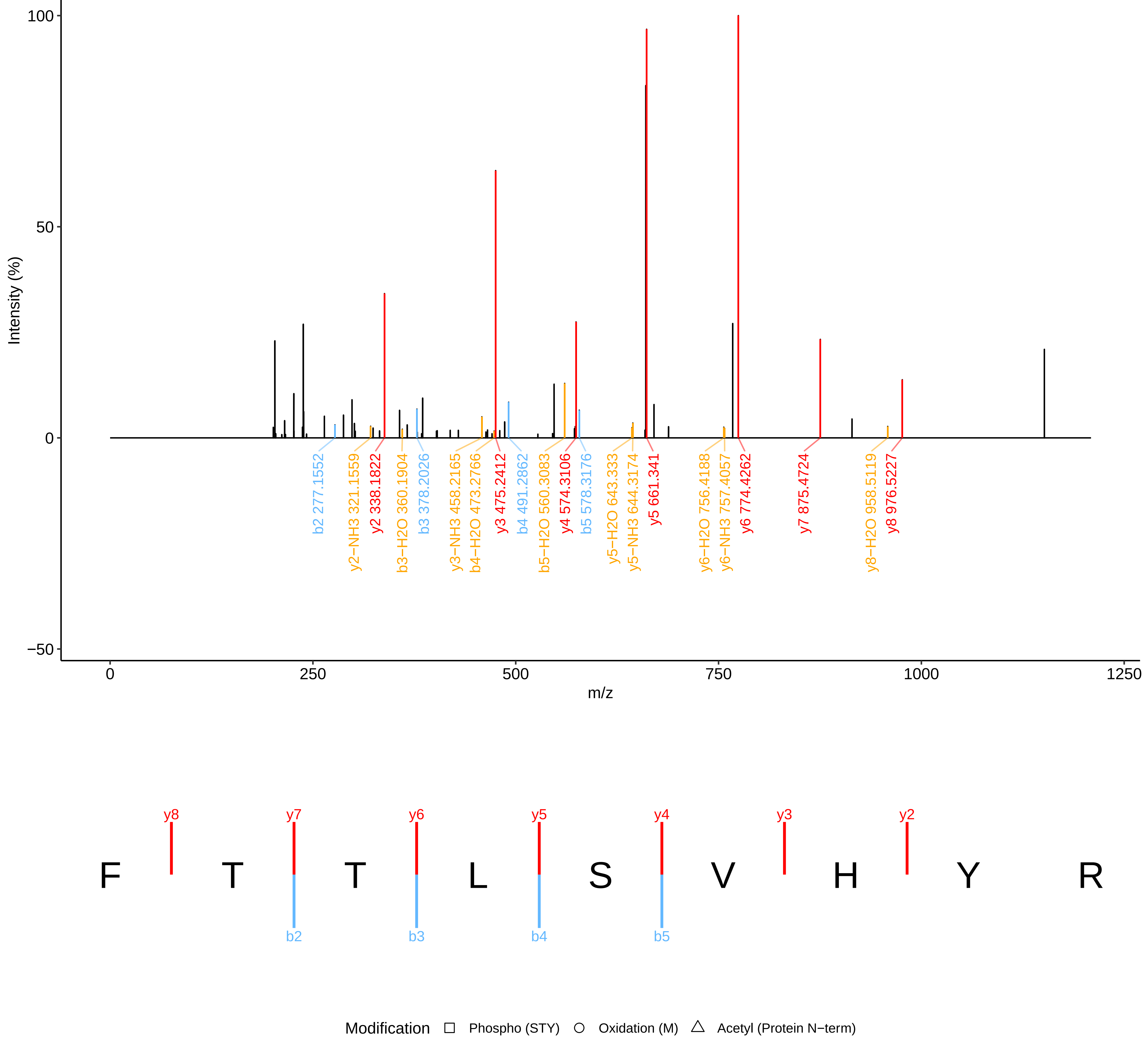

20211217_PS_SCy031_Proteome_R2_HC_F9 − Scan number: 30421

Gene Names	Charge	m/z	Mass	Mass error [Da]	Mass error [ppm]	Retention time	PEP	Score	Precursor Intensity
slr0519	3	1175.315	3522.923	0.00023772	0.20435	51.607	3.1718e−178	253.12	22914132

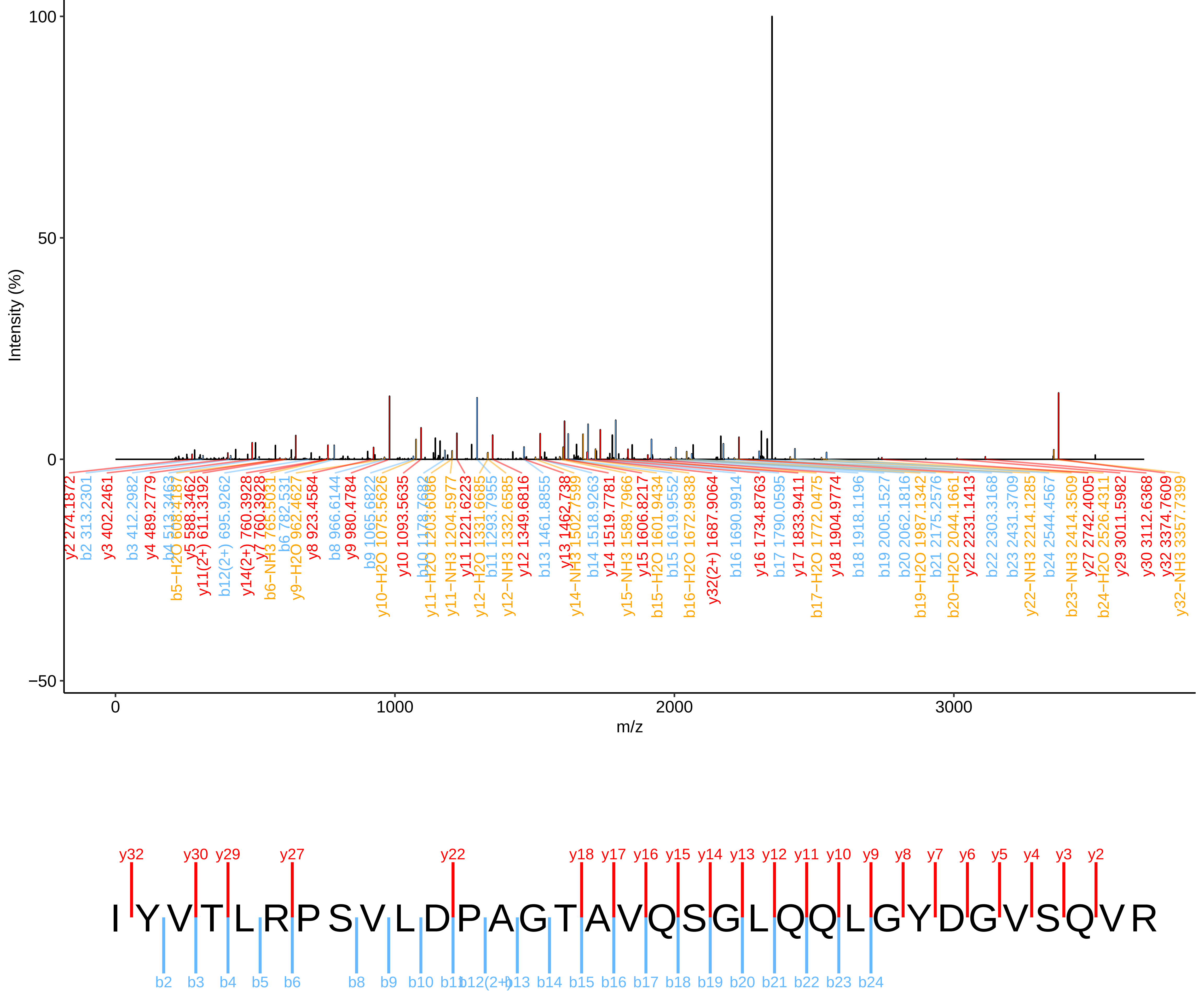

Modification □ Phospho (STY) ○ Oxidation (M) △ Acetyl (Protein N−term)

20211212_PS_SCy031_proteome_R1_3hLC_F7 − Scan number: 18710

Gene Names	Charge	m/z	Mass	Mass error [Da]	Mass error [ppm]	Retention time	PEP	Score	Precursor Intensity
slr0612	3	562.3339	1683.98	NA	NA	28.765	0.00049324	72.417	1277149

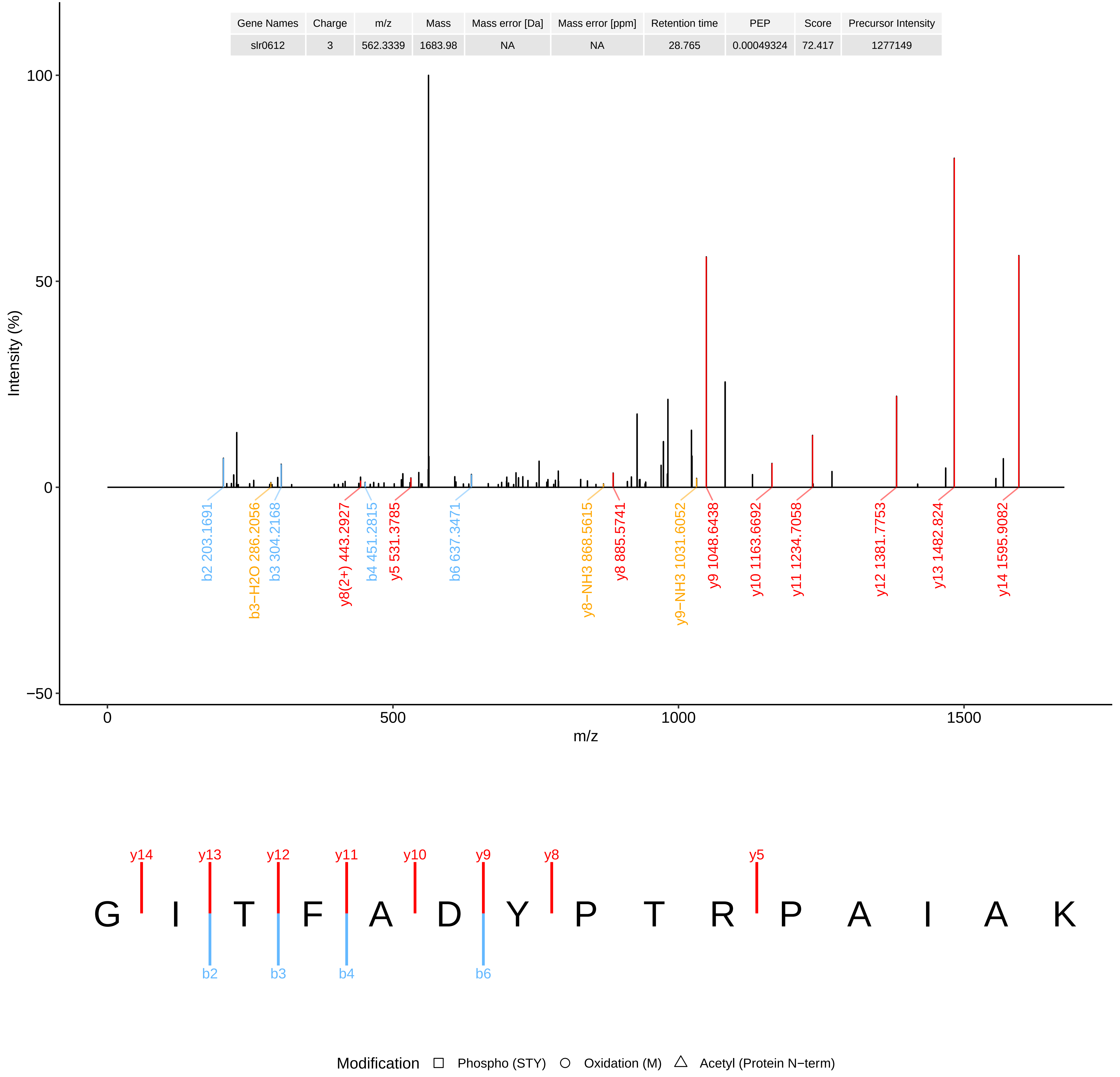

20211217_PS_SCy031_Proteome_R2_HC_F7 − Scan number: 20034

Gene Names	Charge	m/z	Mass	Mass error [Da]	Mass error [ppm]	Retention time	PEP	Score	Precursor Intensity
slr0679	4	560.8145	2239.229	−9.2303e−05	−0.16728	31.971	0.00010553	57.338	1683173

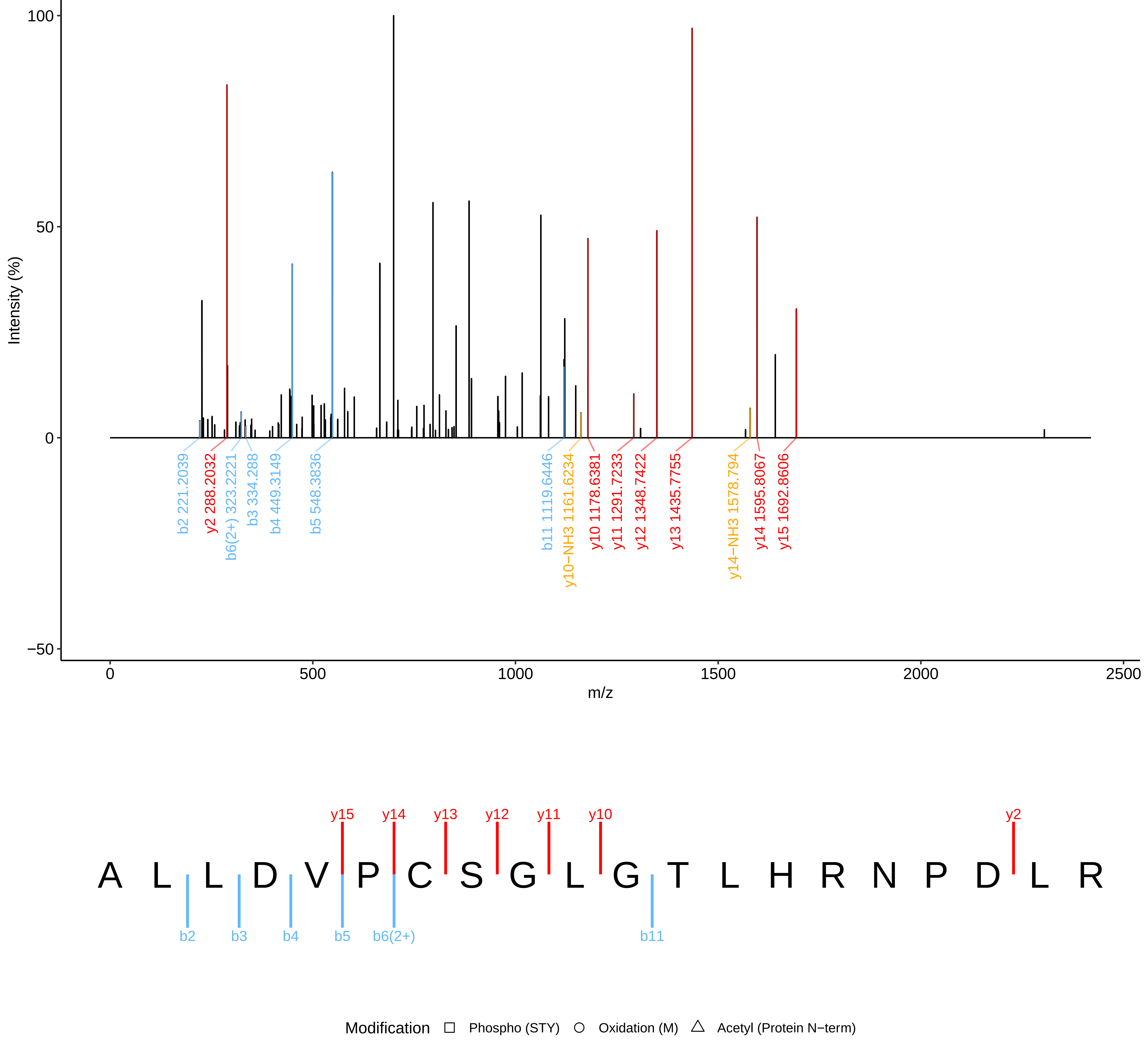

20211217_PS_SCy031_Proteome_R3_3hLC_F2 − Scan number: 10198

Gene Names	Charge	m/z	Mass	Mass error [Da]	Mass error [ppm]	Retention time	PEP	Score	Precursor Intensity
slr0747	2	448.7605	895.5065	−0.00019696	−0.45514	17.284	0.00043281	71.852	6209976

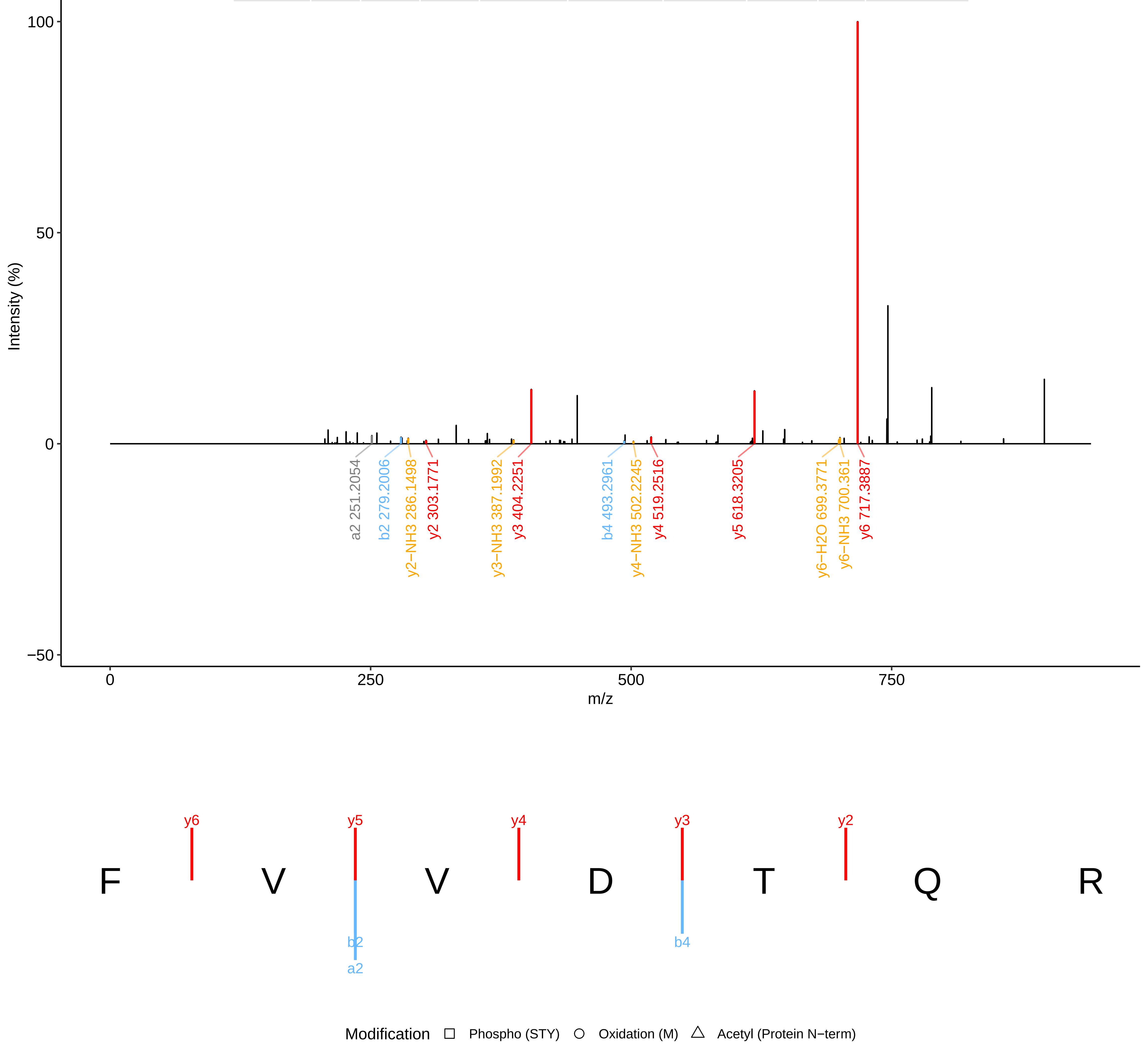

20211217_PS_SCy031_Proteome_R3_3hLC_F9 − Scan number: 22933

Gene Names	Charge	m/z	Mass	Mass error [Da]	Mass error [ppm]	Retention time	PEP	Score	Precursor Intensity
slr0784	2	481.2758	960.5371	4.3462e−05	0.093418	40.041	0.00043078	71.949	1302856

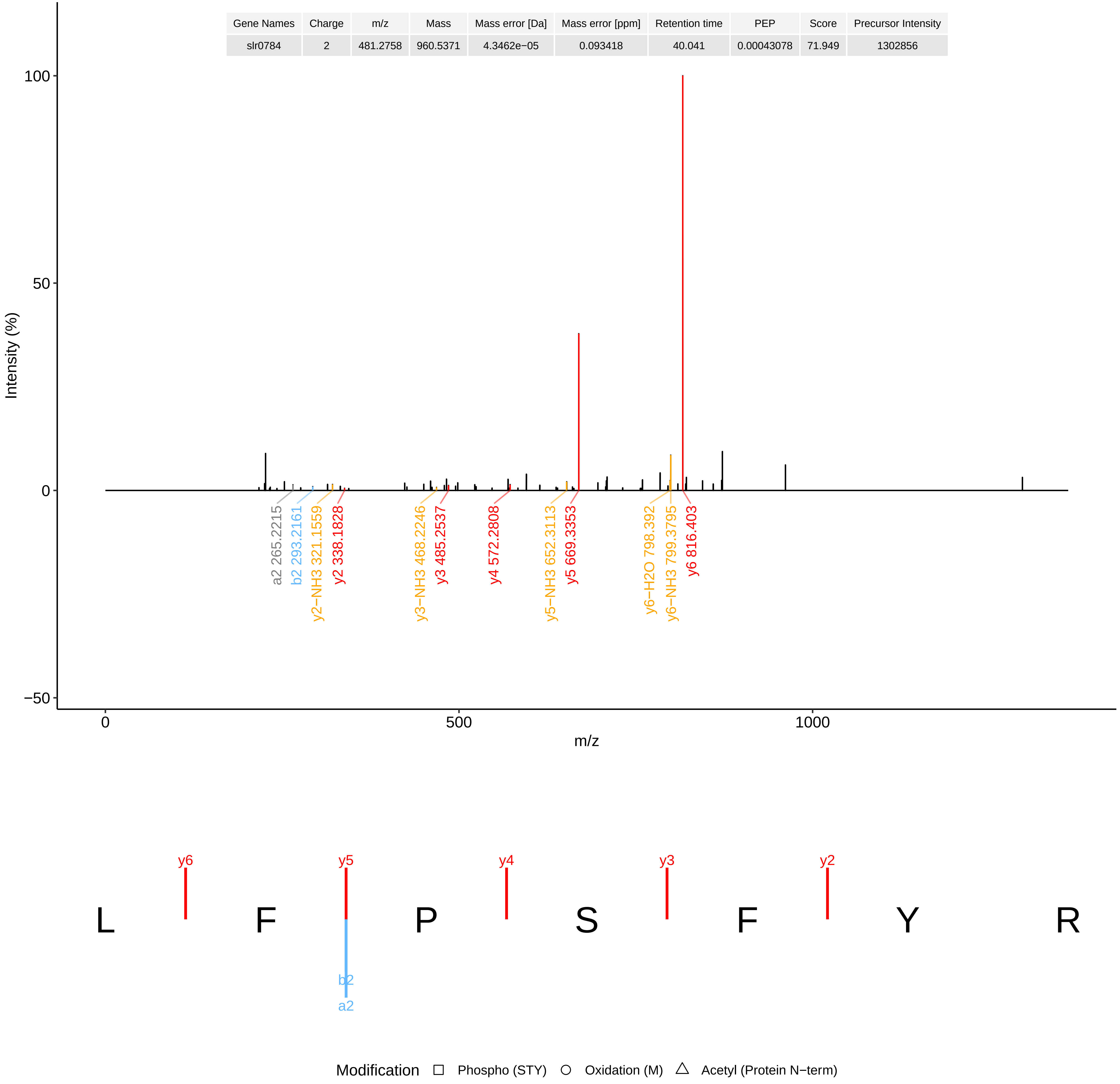

20211212_PS_SCy031_proteome_R1_HC_F8 − Scan number: 32765

Gene Names	Charge	m/z	Mass	Mass error [Da]	Mass error [ppm]	Retention time	PEP	Score	Precursor Intensity
slr0853	3	507.3034	1518.888	−0.0002257	−0.45324	49.784	9.6465e−06	98.531	6106340

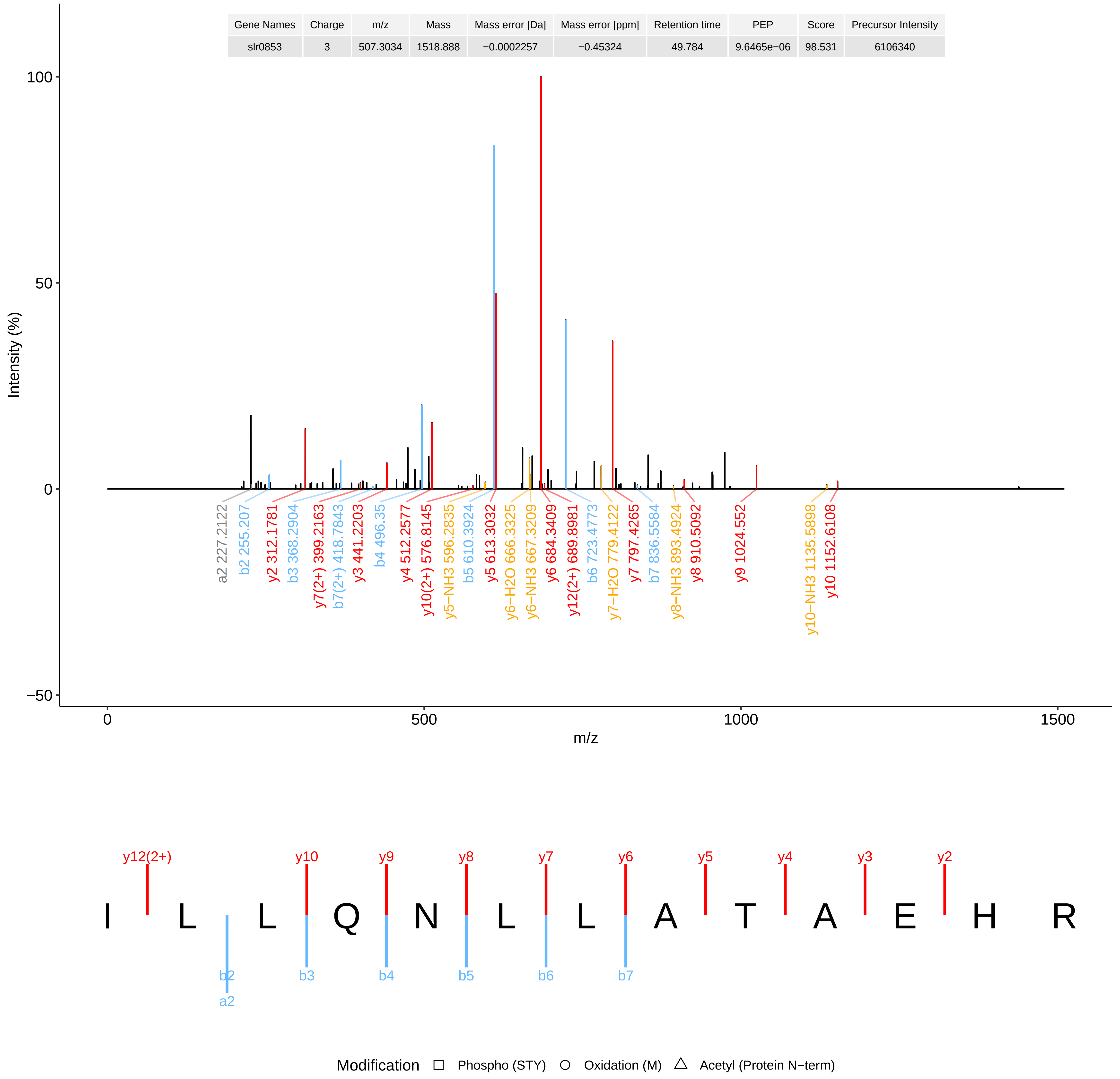

20211212_PS_SCy031_proteome_R1_HC_F1 − Scan number: 17608

Gene Names	Charge	m/z	Mass	Mass error [Da]	Mass error [ppm]	Retention time	PEP	Score	Precursor Intensity
slr0949	2	536.3059	1070.597	−0.00020125	−0.39596	25.935	8.4164e−06	85.533	5626370

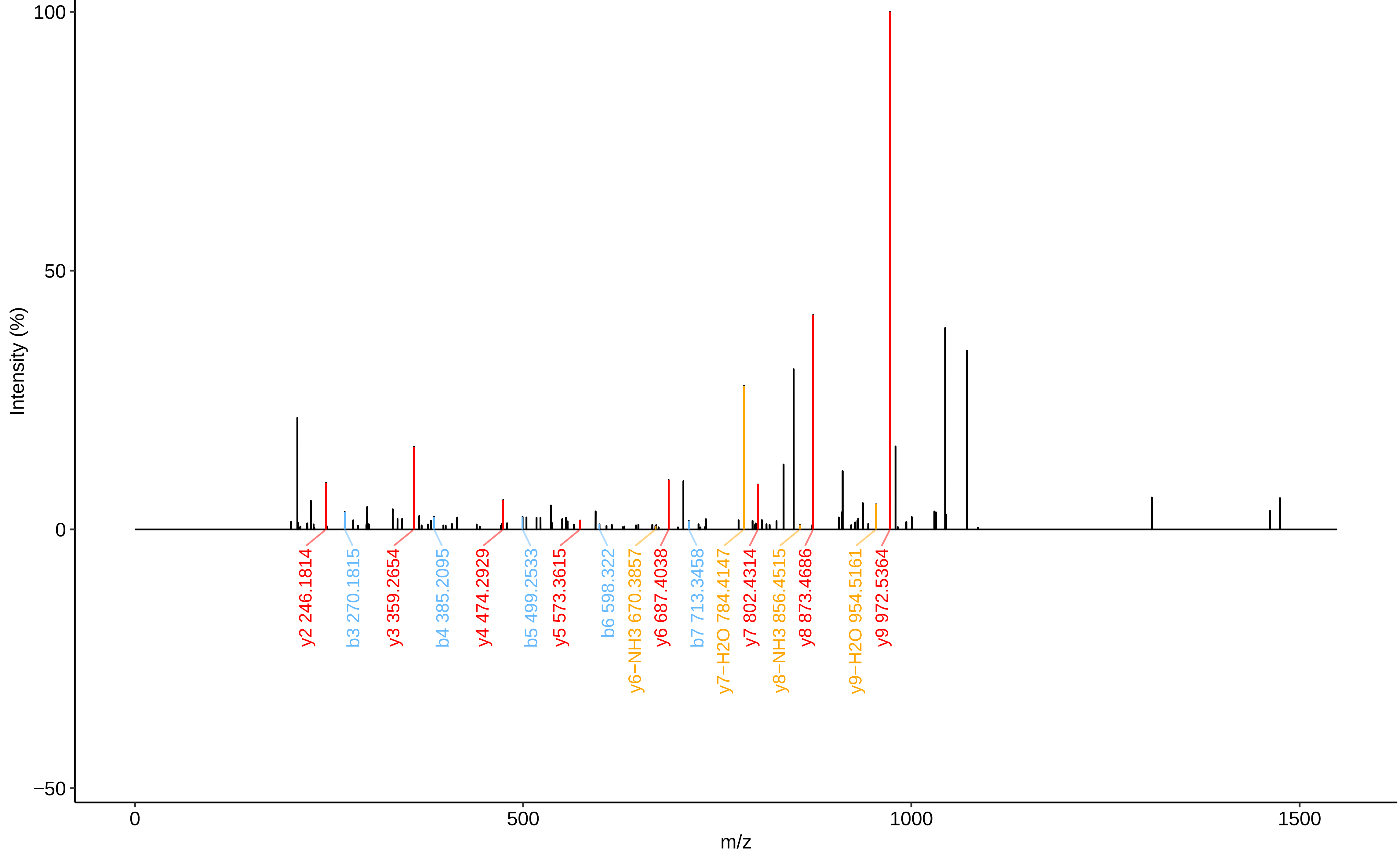

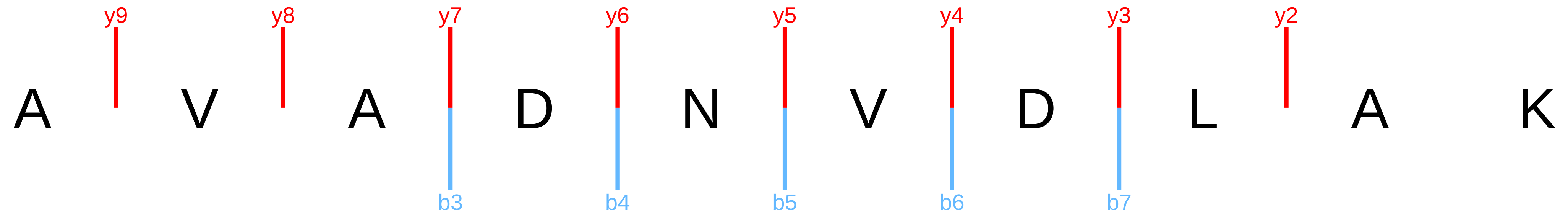

Modification □ Phospho (STY) ○ Oxidation (M) △ Acetyl (Protein N−term)

20211212_PS_SCy031_proteome_R1_3hLC_F8 − Scan number: 12043

Gene Names	Charge	m/z	Mass	Mass error [Da]	Mass error [ppm]	Retention time	PEP	Score	Precursor Intensity
slr1079	2	426.7656	851.5167	0.00034813	0.84757	20.953	0.00032875	76.833	1863357

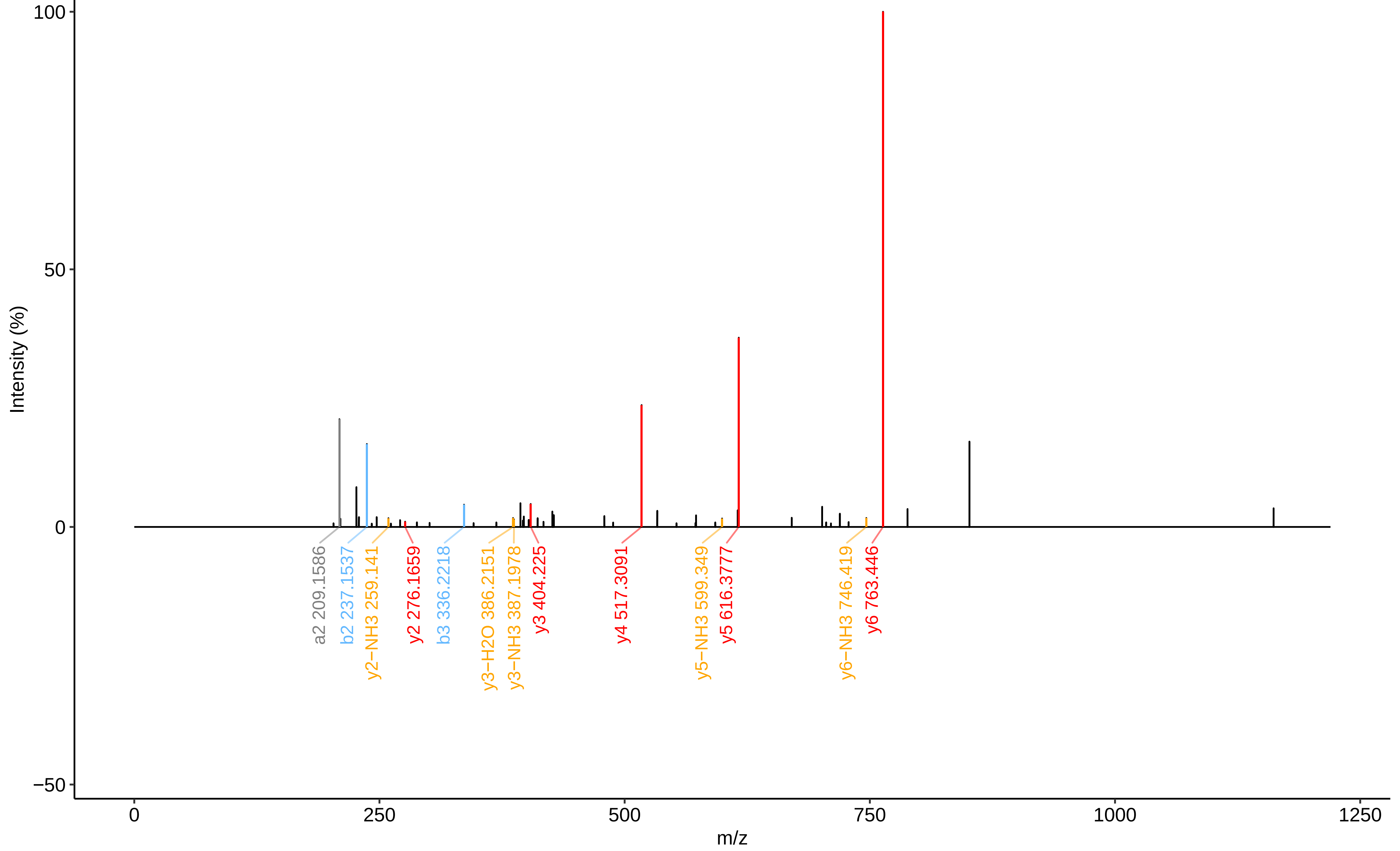

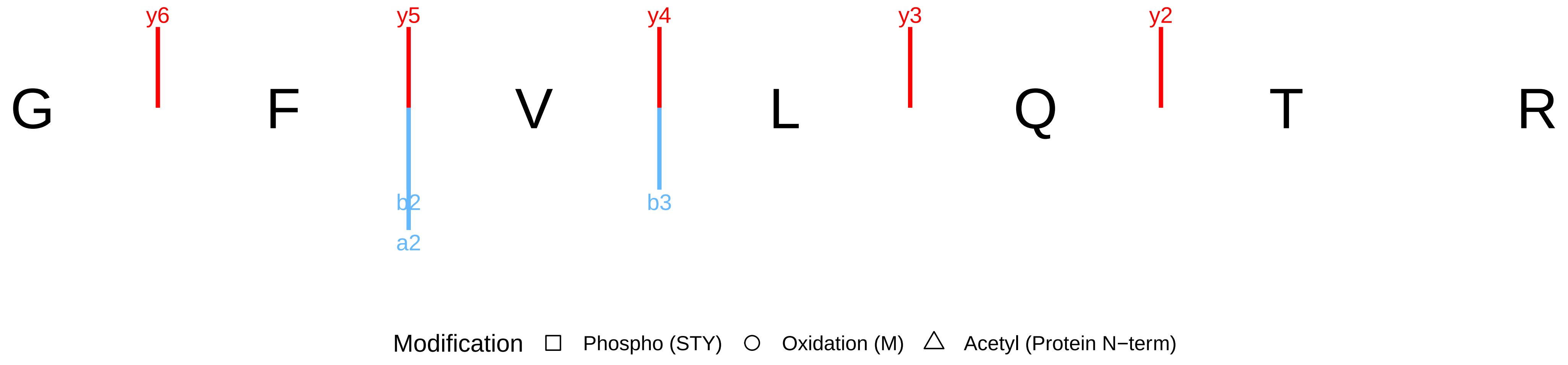

20211217_PS_SCy031_Proteome_R3_24hLC_F5 − Scan number: 15310

Gene Names	Charge	m/z	Mass	Mass error [Da]	Mass error [ppm]	Retention time	PEP	Score	Precursor Intensity
slr1094	3	465.5895	1393.747	0.00020128	0.43199	26.132	0.00024064	81.799	NA

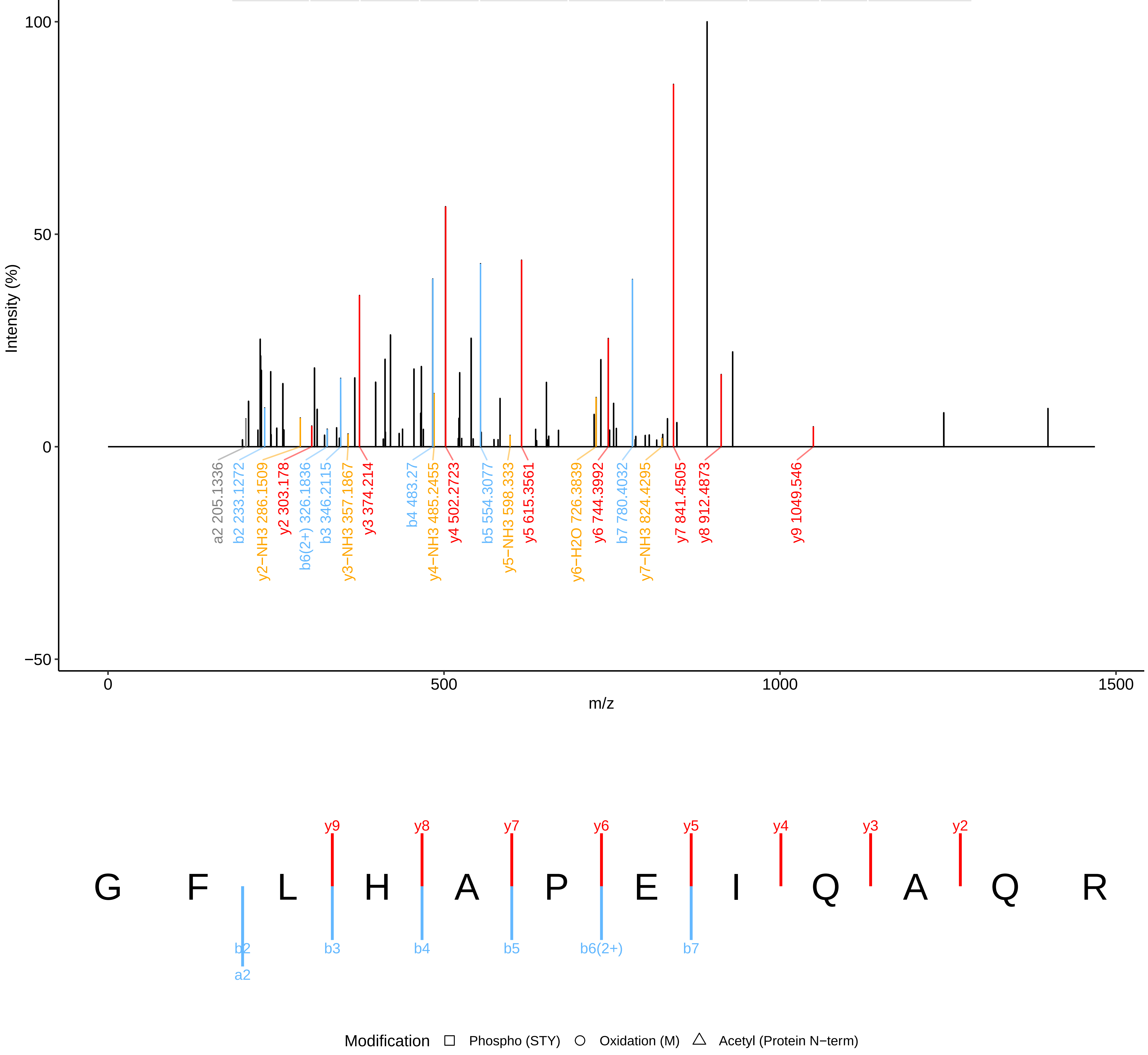

20211212_PS_SCy031_proteome_R1_3hLC_F9 − Scan number: 29083

Gene Names	Charge	m/z	Mass	Mass error [Da]	Mass error [ppm]	Retention time	PEP	Score	Precursor Intensity
slr1107	2	546.8393	1091.664	−0.00060092	−1.1321	43.3	0.00014579	70.919	772565.1

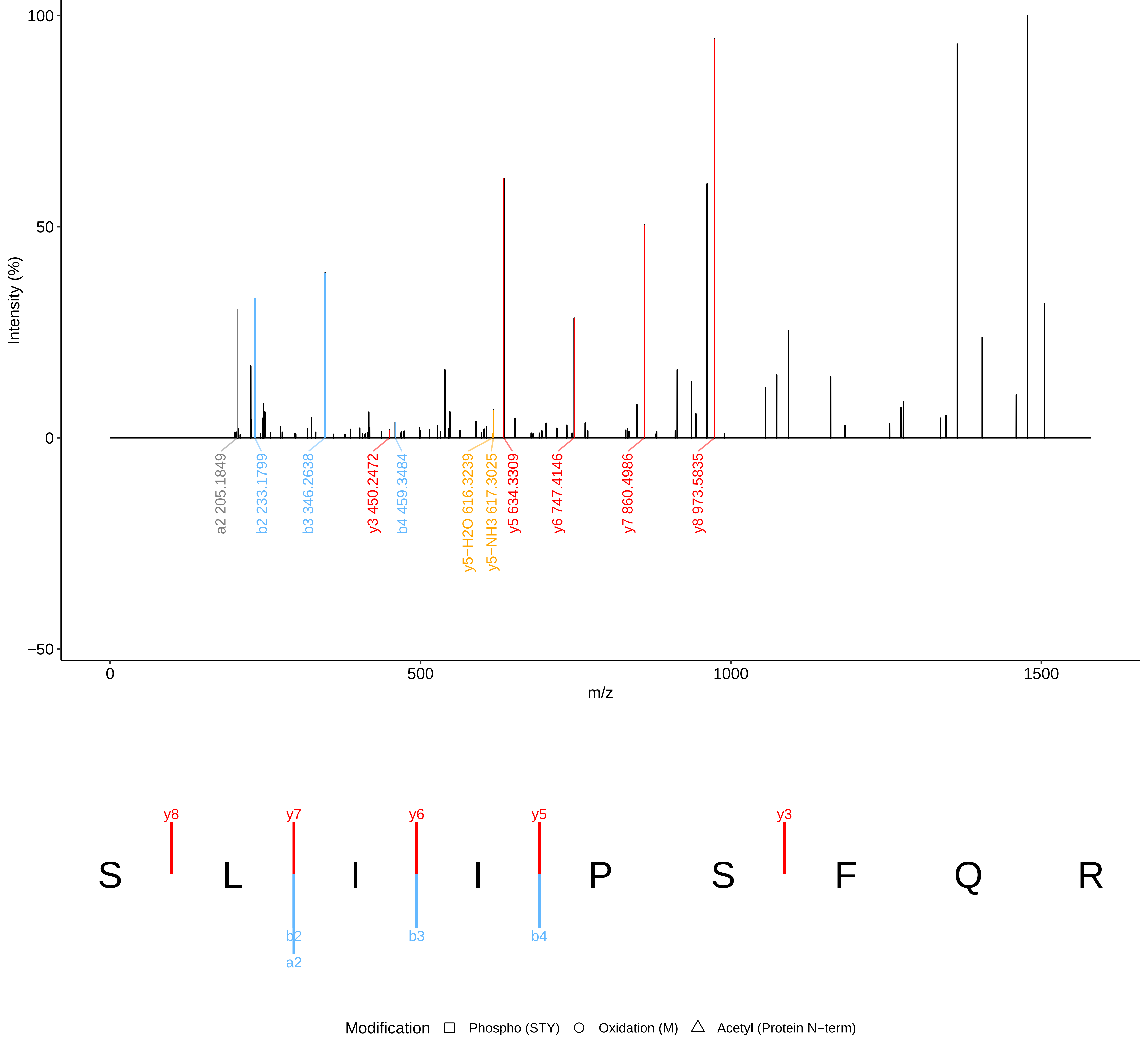

20211217_PS_SCy031_Proteome_R3_24hLC_F4 − Scan number: 11130

Gene Names	Charge	m/z	Mass	Mass error [Da]	Mass error [ppm]	Retention time	PEP	Score	Precursor Intensity
slr1113	2	530.371	1058.727	NA	NA	20.168	8.6758e−05	106.18	4305878

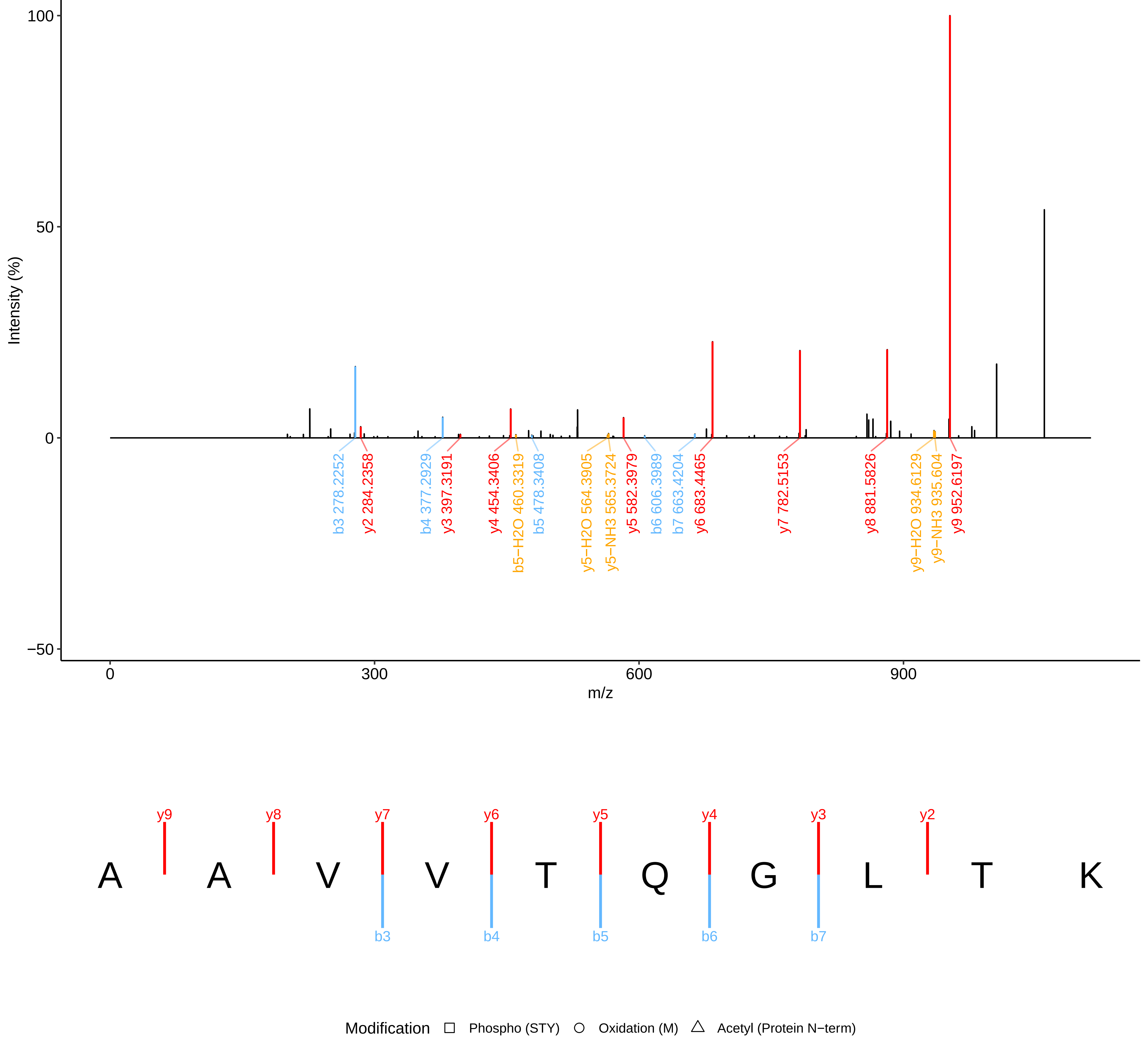

20211217_PS_SCy031_Proteome_R3_24hLC_F9 − Scan number: 7798

Gene Names	Charge	m/z	Mass	Mass error [Da]	Mass error [ppm]	Retention time	PEP	Score	Precursor Intensity
slr1253	3	611.6527	1831.936	−0.00012394	−0.20578	16.933	1.7353e−33	187.16	1221989

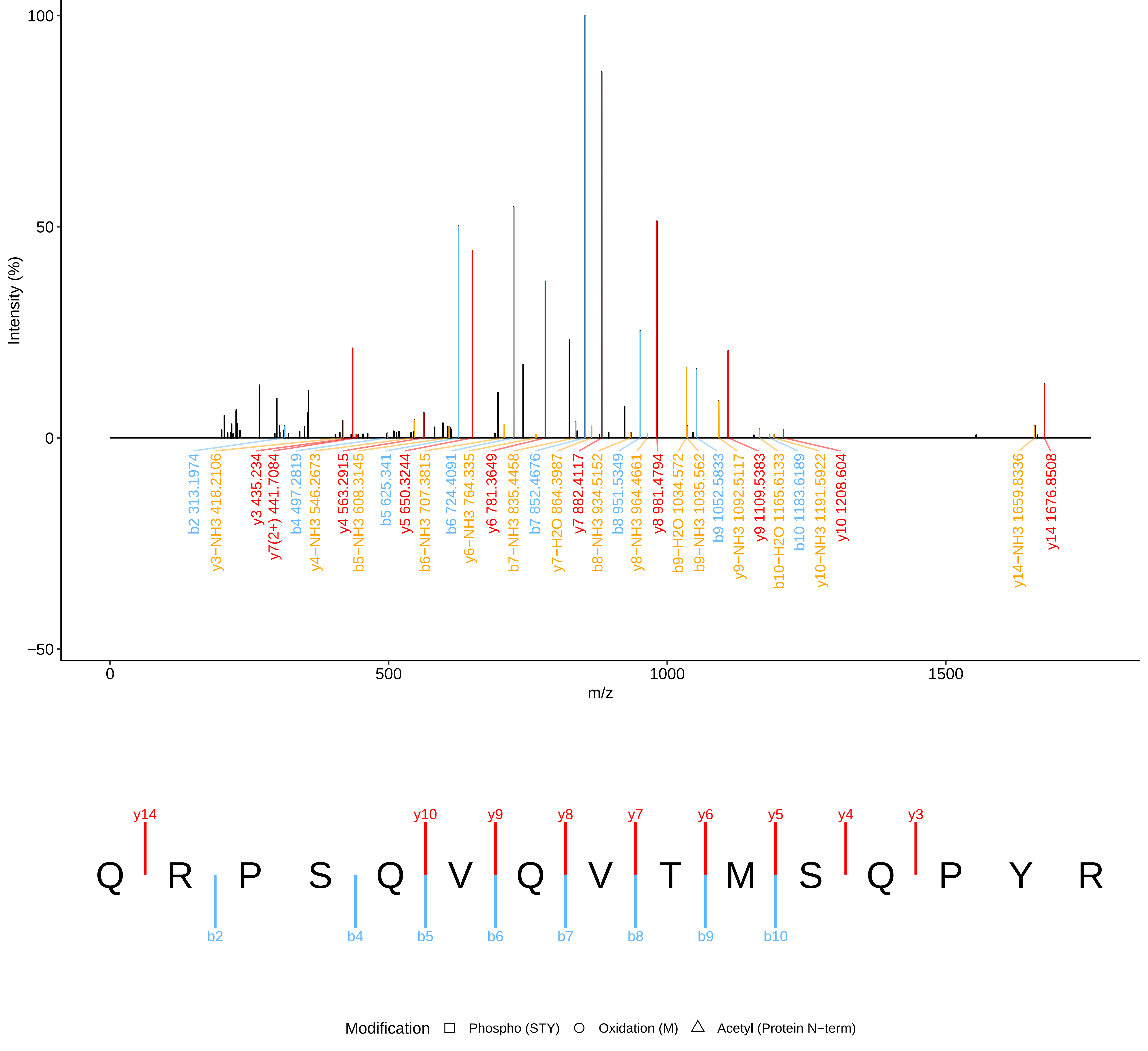

20211217_PS_SCy031_Proteome_R3_24hLC_F3 − Scan number: 7503

Gene Names	Charge	m/z	Mass	Mass error [Da]	Mass error [ppm]	Retention time	PEP	Score	Precursor Intensity
slr1376	3	392.2625	1173.766	1.082e−05	0.029384	14.08	2.0936e−05	74.841	4382893

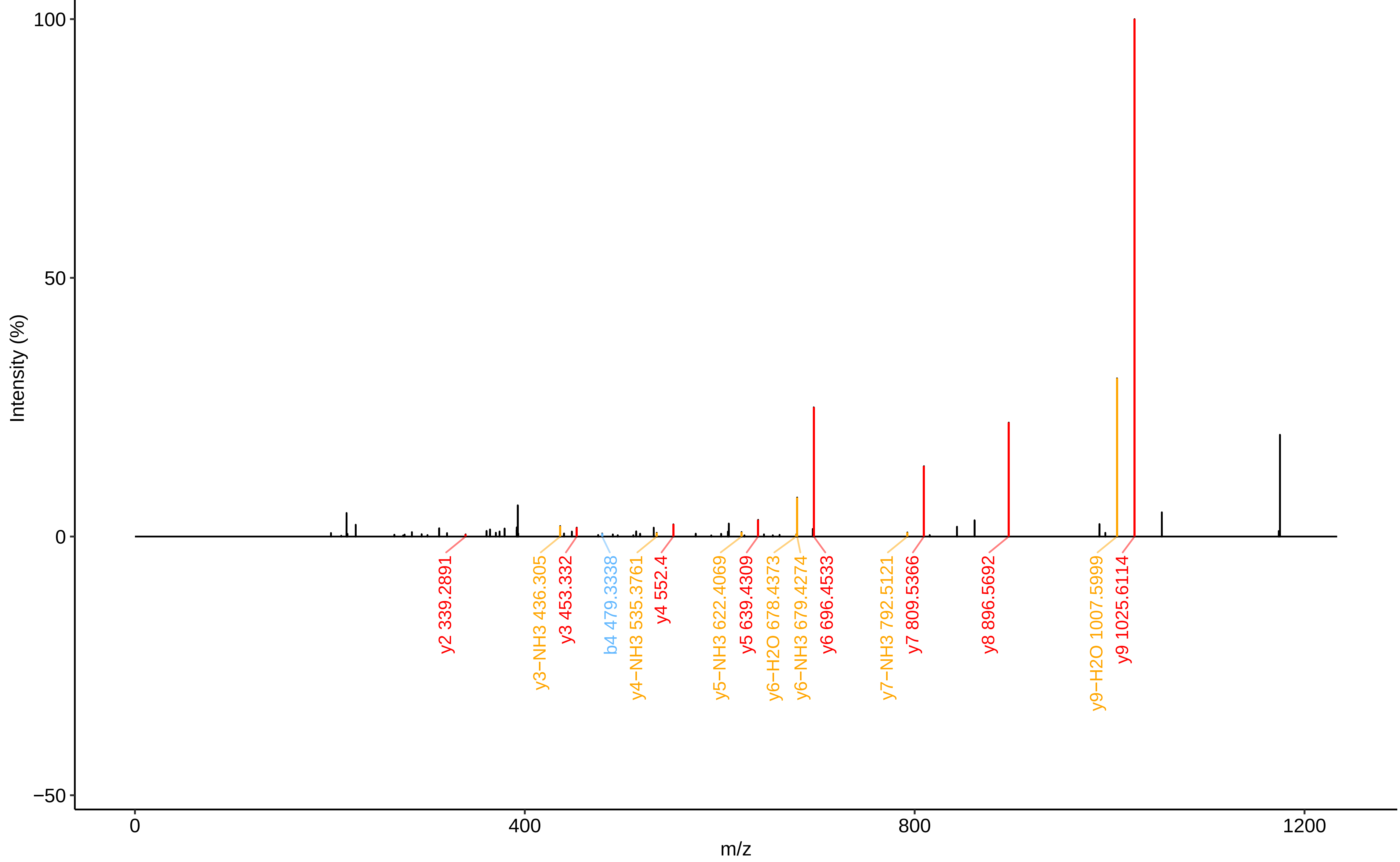

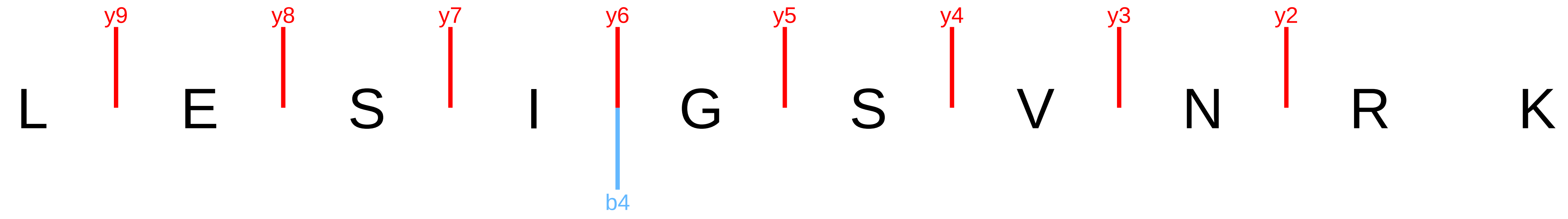

Modification □ Phospho (STY) ○ Oxidation (M) △ Acetyl (Protein N−term)

20211212_PS_SCy031_proteome_R1_3hLC_F9 − Scan number: 28327

Gene Names	Charge	m/z	Mass	Mass error [Da]	Mass error [ppm]	Retention time	PEP	Score	Precursor Intensity
slr1400	2	625.4201	1248.826	0.00028753	0.48457	42.327	9.5708e−06	99.973	2775148

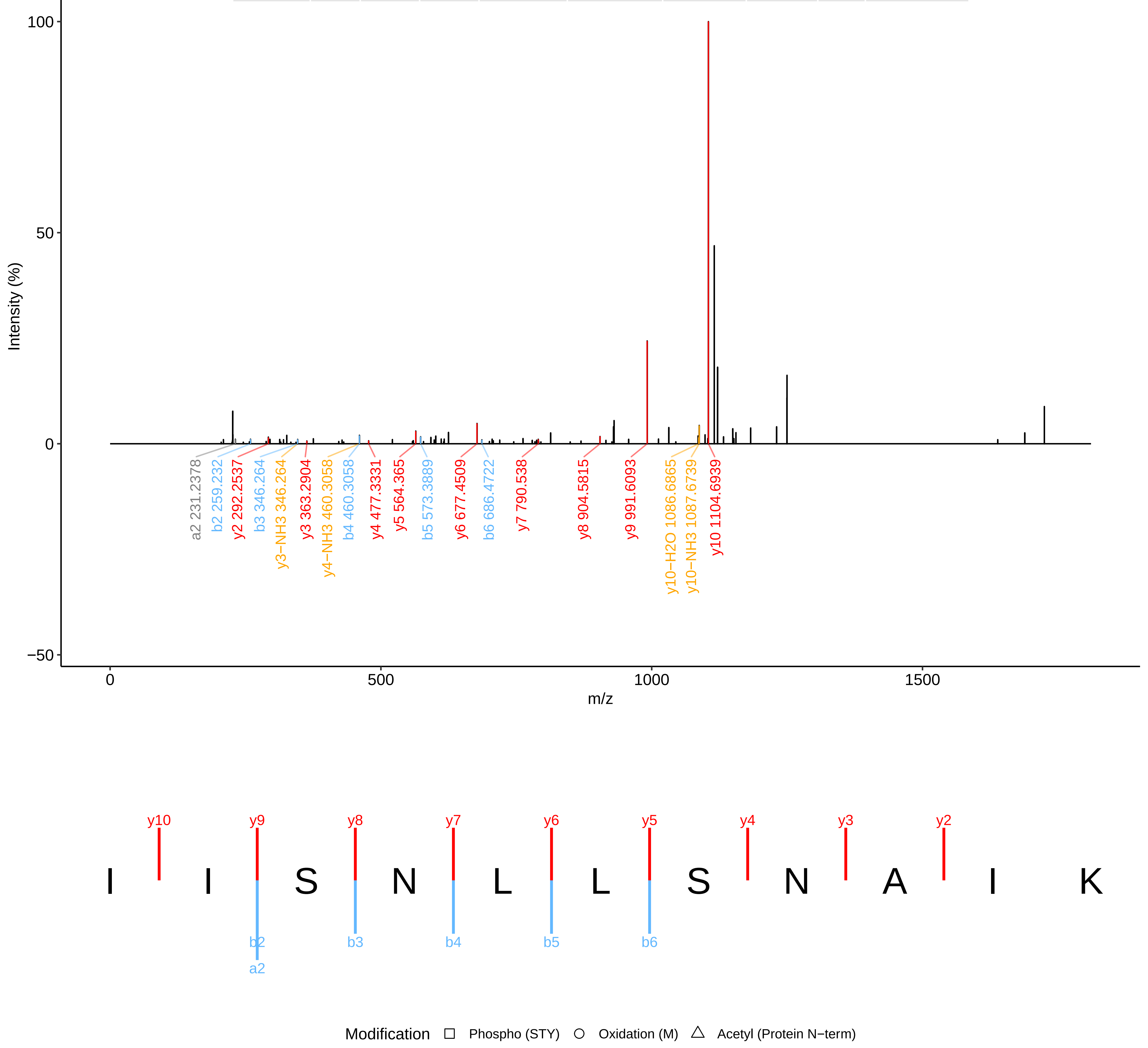

20211212_PS_SCy031_proteome_R1_24hLC_F3 − Scan number: 18795

Gene Names	Charge	m/z	Mass	Mass error [Da]	Mass error [ppm]	Retention time	PEP	Score	Precursor Intensity
slr1417	2	544.3684	1086.722	−0.00027634	−0.50763	27.997	3.1483e−05	142.43	62443848

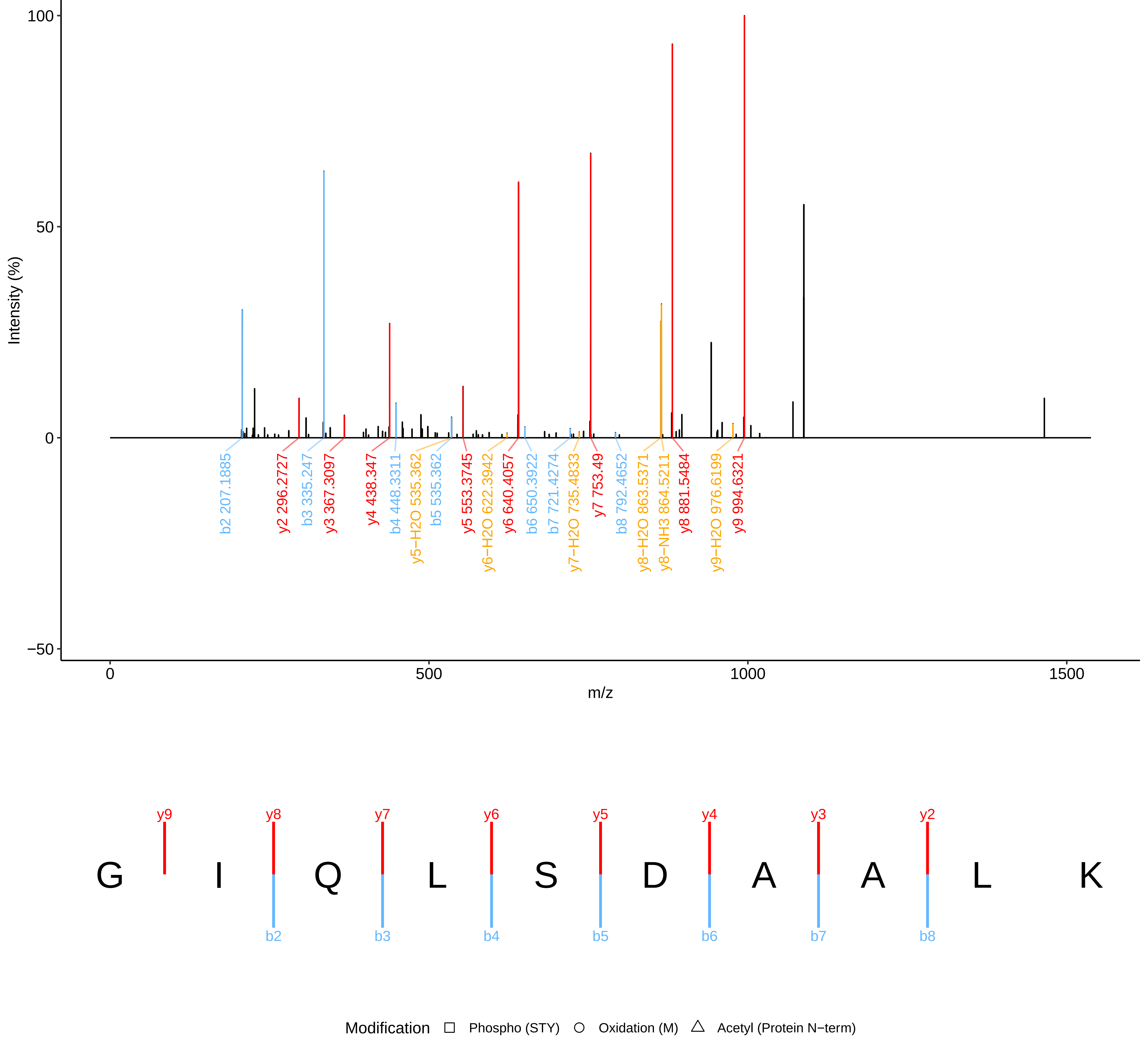

20211217_PS_SCy031_Proteome_R2_HC_F3 − Scan number: 28243

Gene Names	Charge	m/z	Mass	Mass error [Da]	Mass error [ppm]	Retention time	PEP	Score	Precursor Intensity
slr1429	2	696.3952	1390.776	−0.0014791	−2.1739	40.48	0.00030351	56.043	2645613

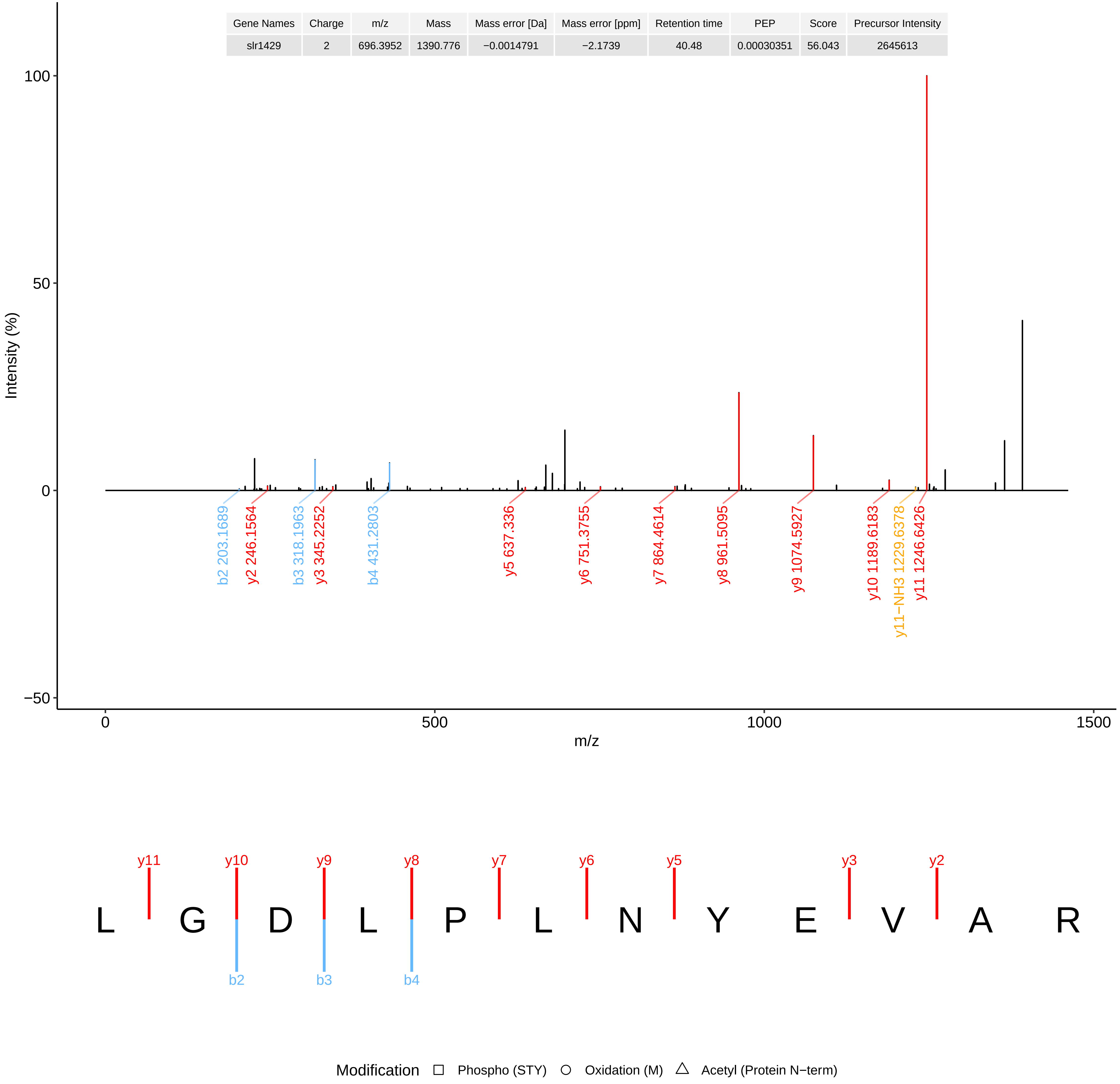

20211212_PS_SCy031_proteome_R1_3hLC_F5 − Scan number: 27015

Gene Names	Charge	m/z	Mass	Mass error [Da]	Mass error [ppm]	Retention time	PEP	Score	Precursor Intensity
slr1461	2	640.8353	1279.656	NA	NA	39.496	9.0578e−06	137.05	NA

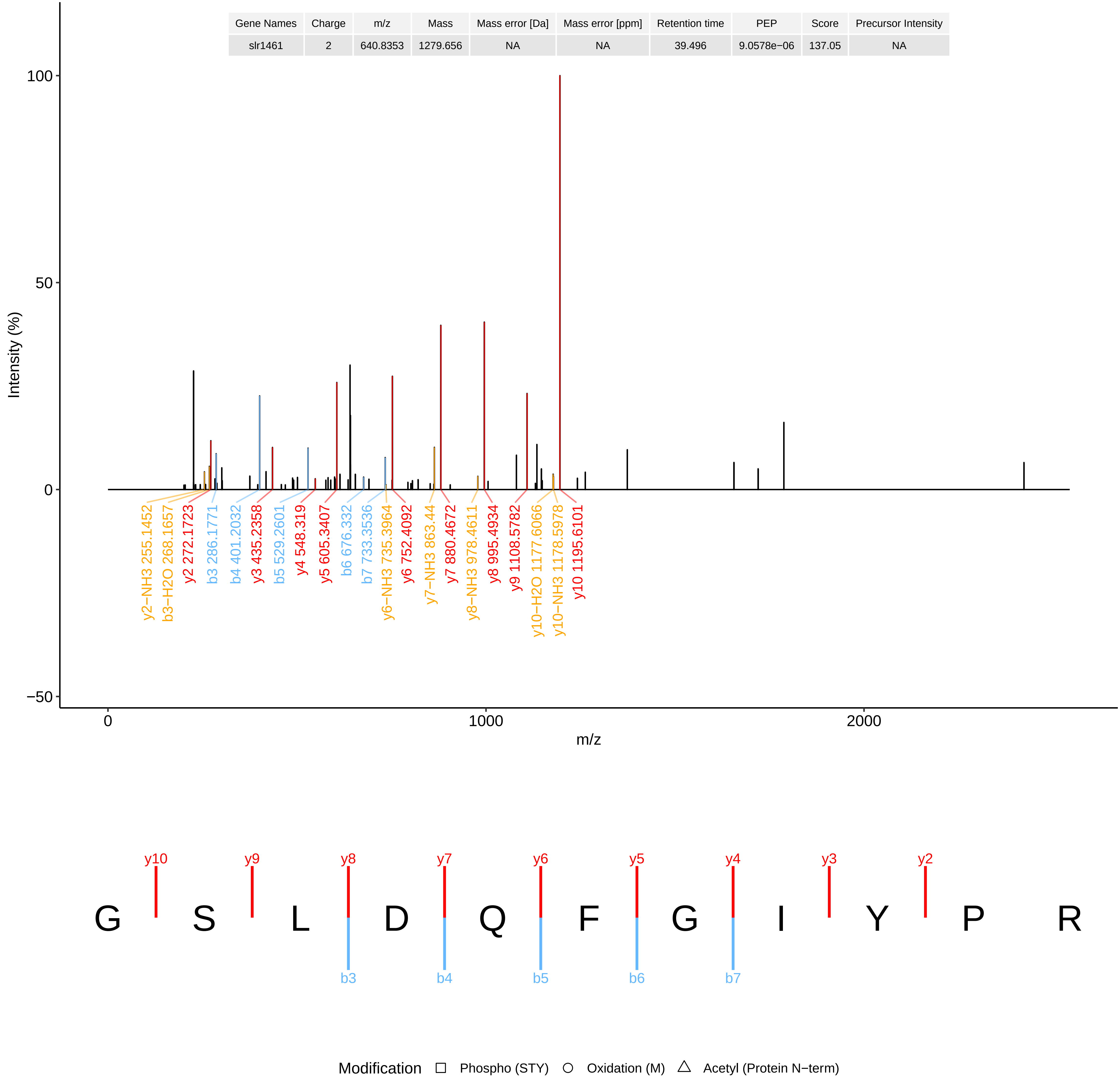

20211212_PS_SCy031_proteome_R1_24hLC_F2 − Scan number: 6784

Gene Names	Charge	m/z	Mass	Mass error [Da]	Mass error [ppm]	Retention time	PEP	Score	Precursor Intensity
slr1614	3	322.5163	964.5272	−0.00011828	−0.38094	12.728	0.00061885	62.464	12235540

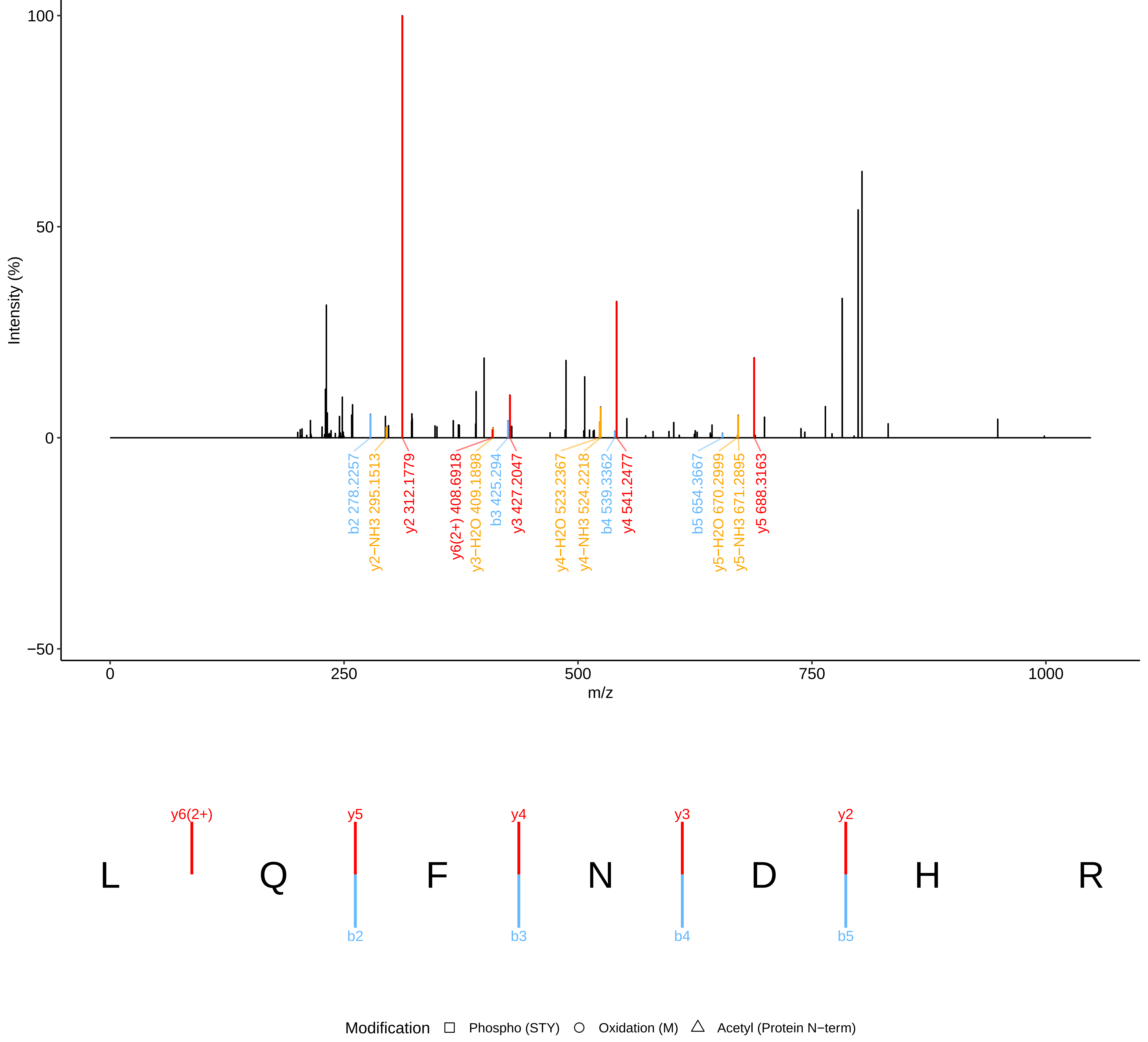

20211212_PS_SCy031_proteome_R1_24hLC_F4 − Scan number: 7673

Gene Names	Charge	m/z	Mass	Mass error [Da]	Mass error [ppm]	Retention time	PEP	Score	Precursor Intensity
slr1647	2	567.3446	1132.675	7.3543e−05	0.13388	14.43	1.7951e−08	144.12	6395237

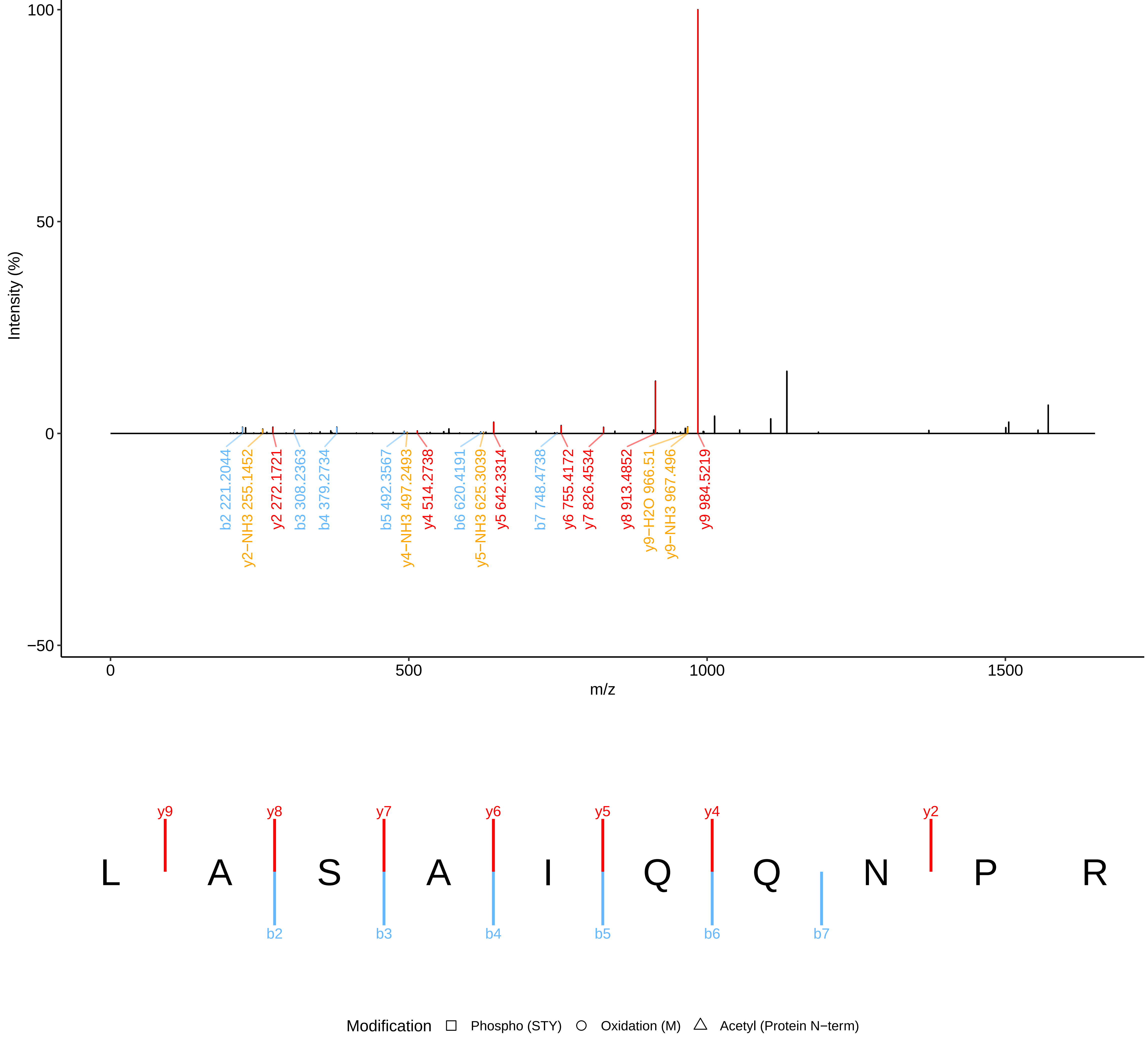

20211217_PS_SCy031_Proteome_R2_HC_F2 − Scan number: 8568

Gene Names	Charge	m/z	Mass	Mass error [Da]	Mass error [ppm]	Retention time	PEP	Score	Precursor Intensity
slr1657	3	348.5451	1042.613	−0.00011998	−0.3537	15.093	5.8461e−05	90.906	8096481

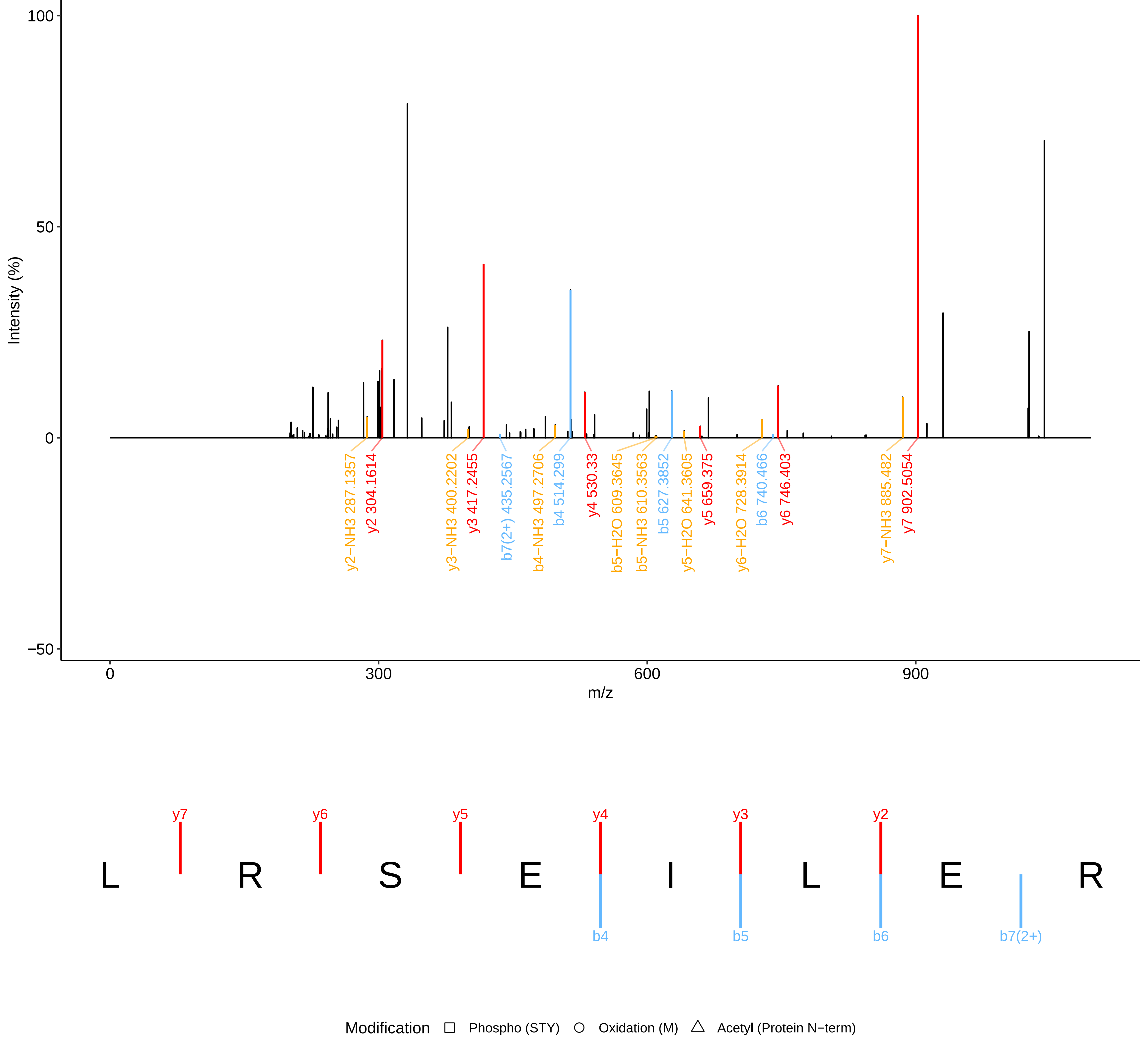

20211212_PS_SCy031_proteome_R1_3hLC_F4 − Scan number: 4792

Gene Names	Charge	m/z	Mass	Mass error [Da]	Mass error [ppm]	Retention time	PEP	Score	Precursor Intensity
slr1770	3	315.8722	944.5949	7.3766e−05	0.24277	10.604	0.00063429	58.981	917071.6

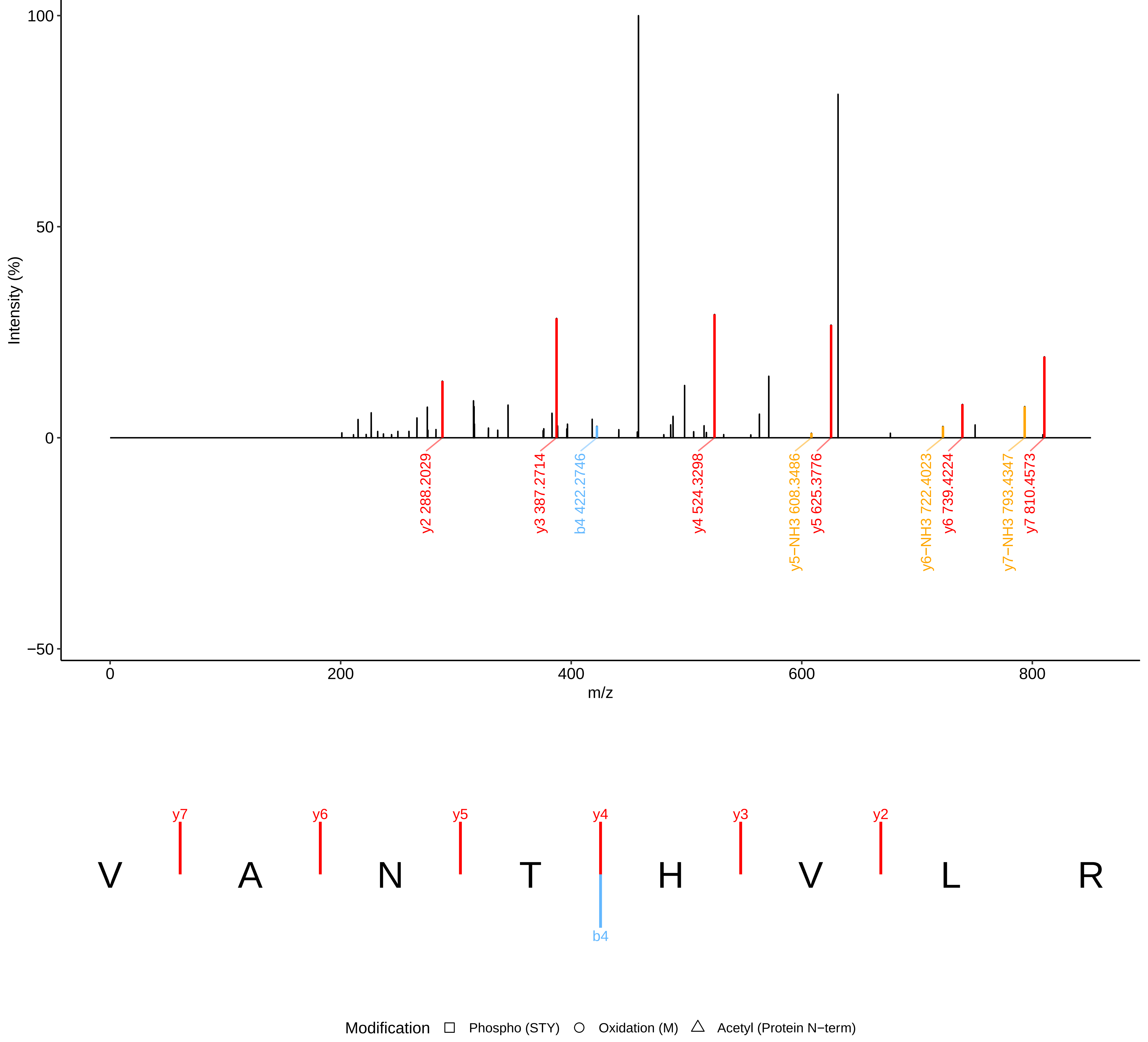

20211212_PS_SCy031_proteome_R1_HC_F7 − Scan number: 23927

Gene Names	Charge	m/z	Mass	Mass error [Da]	Mass error [ppm]	Retention time	PEP	Score	Precursor Intensity
slr1805	3	466.6015	1396.783	−0.00016816	−0.3604	36.09	0.00014774	103.46	3732798

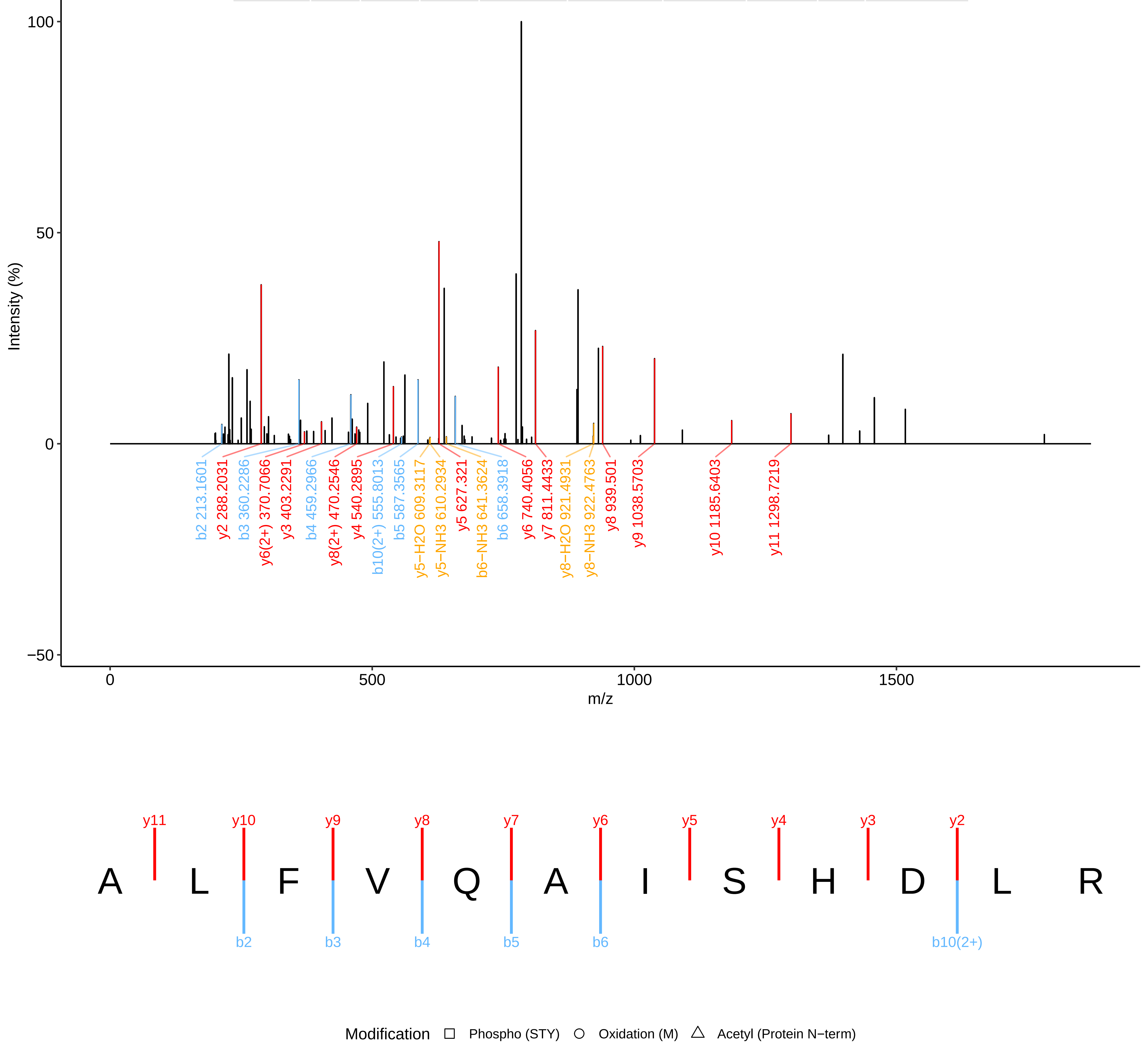

20211217_PS_SCy031_Proteome_R2_HC_F4 − Scan number: 15334

Gene Names	Charge	m/z	Mass	Mass error [Da]	Mass error [ppm]	Retention time	PEP	Score	Precursor Intensity
slr1964	3	332.2106	993.6099	3.689e−05	0.11868	24.879	2.0036e−06	130.27	9403970

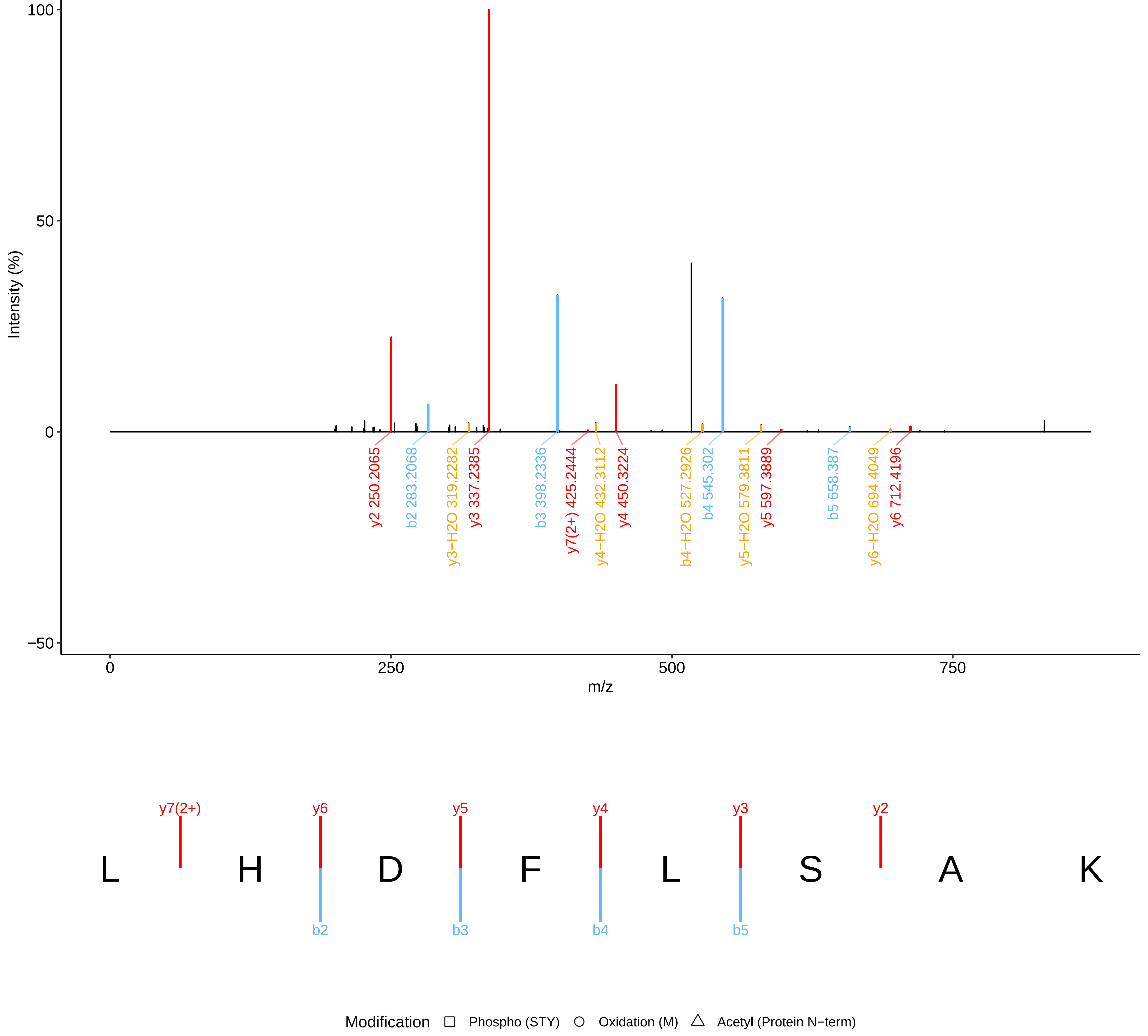

20211217_PS_SCy031_Proteome_R2_3hLC_F4 − Scan number: 11474

Gene Names	Charge	m/z	Mass	Mass error [Da]	Mass error [ppm]	Retention time	PEP	Score	Precursor Intensity
slr2017	2	580.3058	1158.597	−0.00014685	−0.25306	19.895	6.5818e−24	211.45	6322473

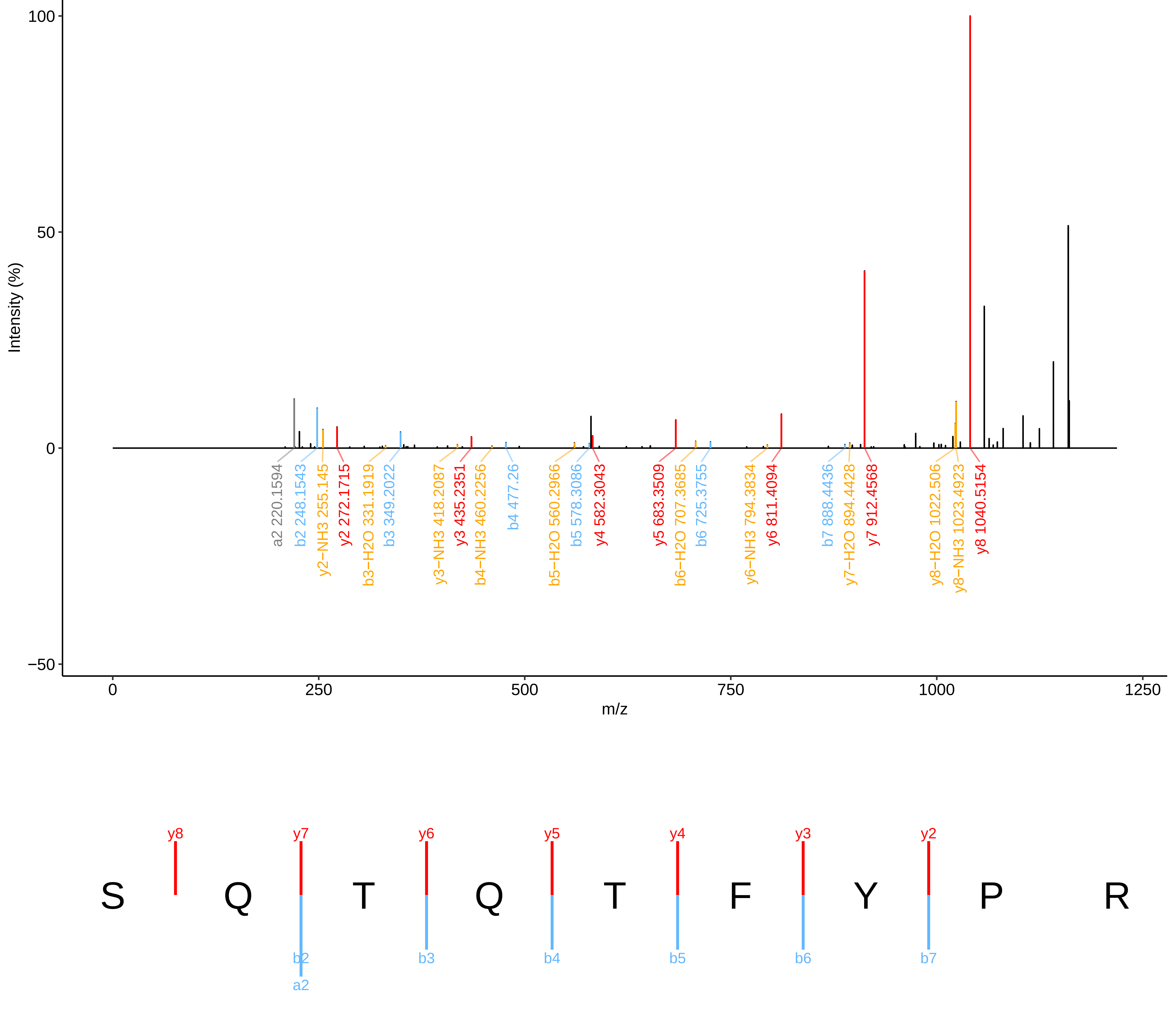

Modification □ Phospho (STY) ○ Oxidation (M) △ Acetyl (Protein N−term)

20211212_PS_SCy031_proteome_R1_3hLC_F9 − Scan number: 24874

Gene Names	Charge	m/z	Mass	Mass error [Da]	Mass error [ppm]	Retention time	PEP	Score	Precursor Intensity
slr2120	3	770.114	2307.32	0.00052882	0.70882	37.87	2.9974e−13	108.92	2579318

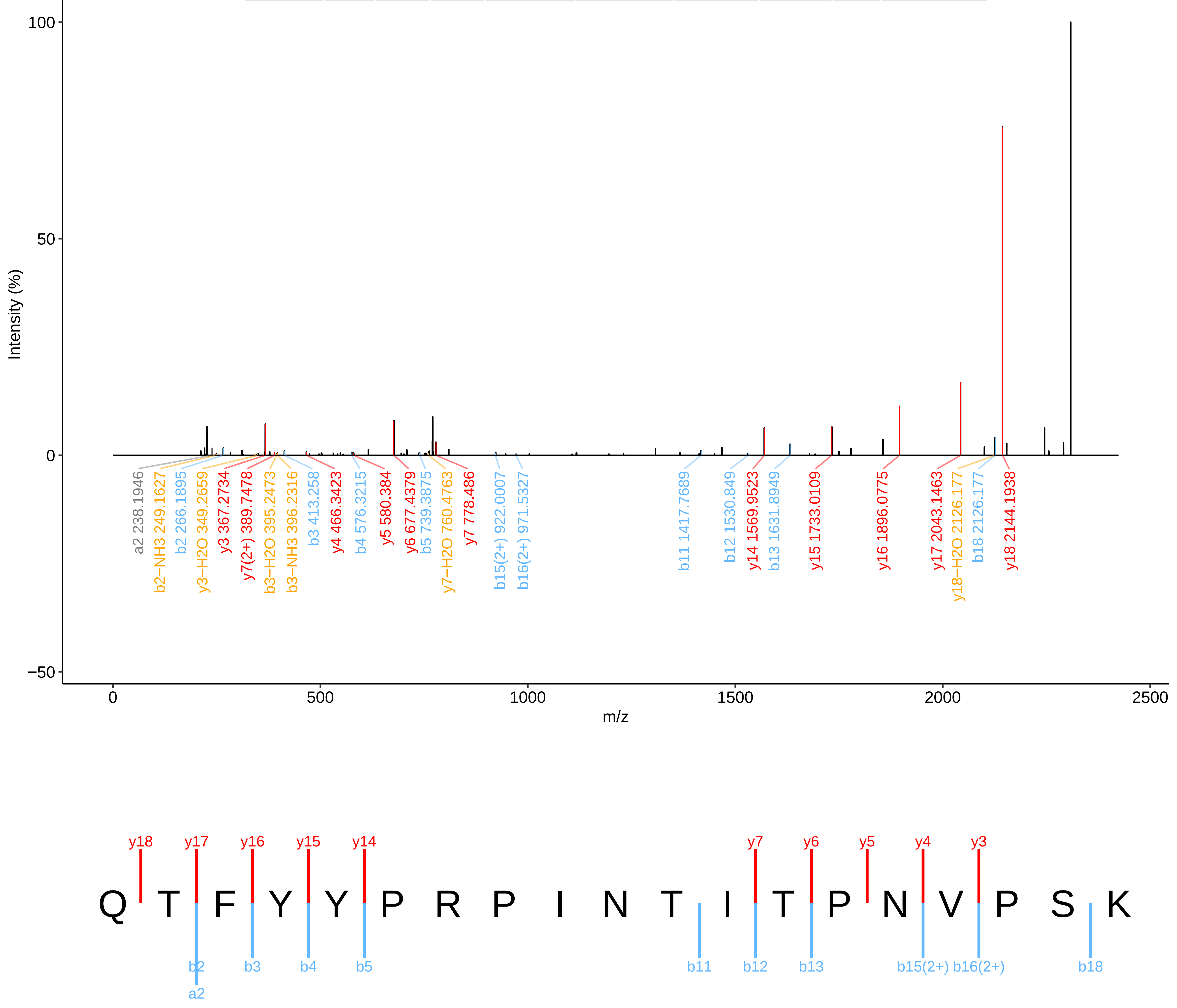

Modification □ Phospho (STY) ○ Oxidation (M) △ Acetyl (Protein N−term)

20211212_PS_SCy031_proteome_R1_HC_F7 − Scan number: 26816

Gene Names	Charge	m/z	Mass	Mass error [Da]	Mass error [ppm]	Retention time	PEP	Score	Precursor Intensity
slr2128	3	495.3009	1482.881	−0.00010117	−0.21226	39.791	2.0352e−14	161.1	8027443

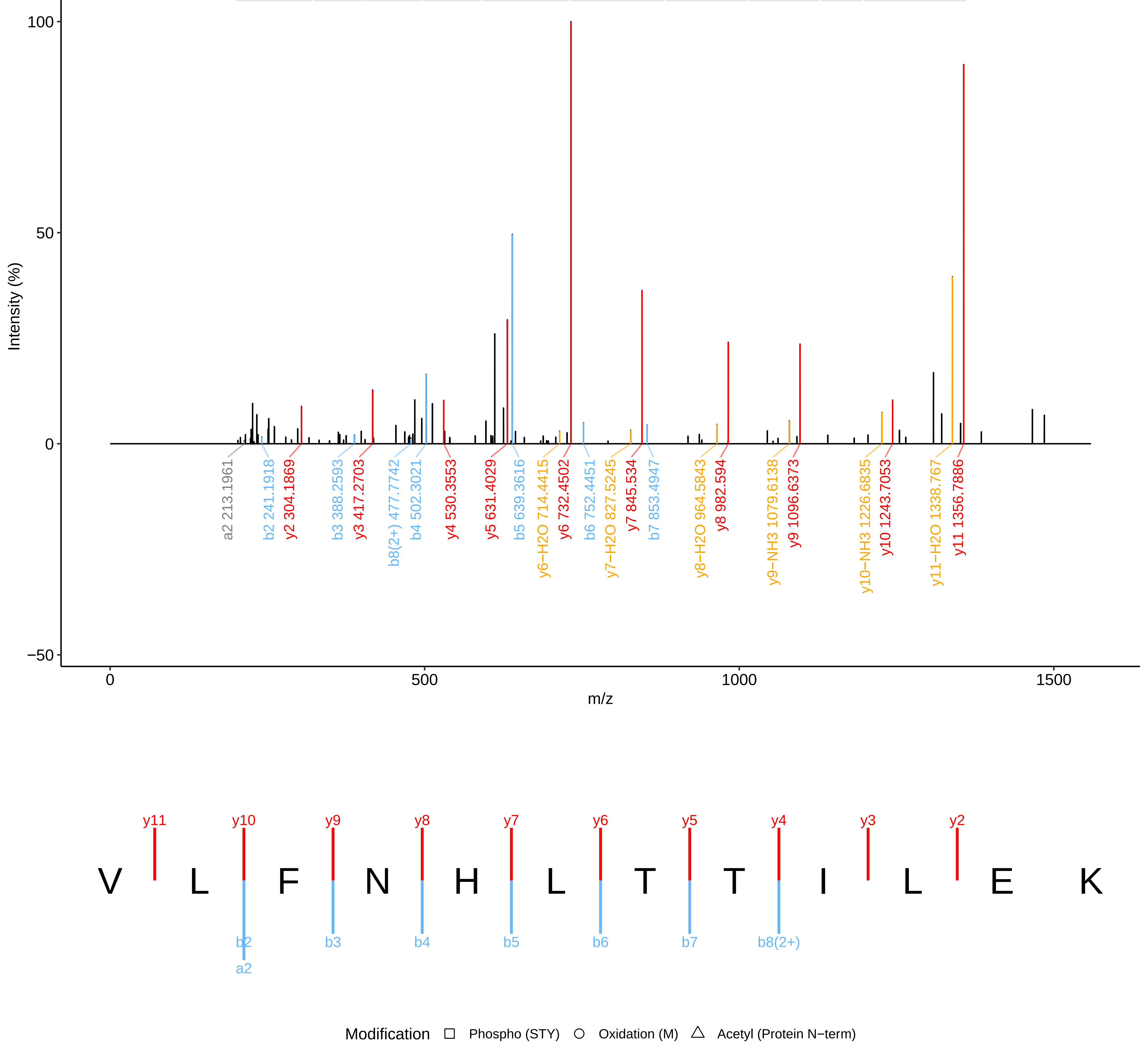

20211212_PS_SCy031_proteome_R1_3hLC_F6 − Scan number: 9425

Gene Names	Charge	m/z	Mass	Mass error [Da]	Mass error [ppm]	Retention time	PEP	Score	Precursor Intensity
slr5056	2	476.2816	950.5487	−0.00057591	−1.2513	17.535	5.2039e−05	94.662	4951131

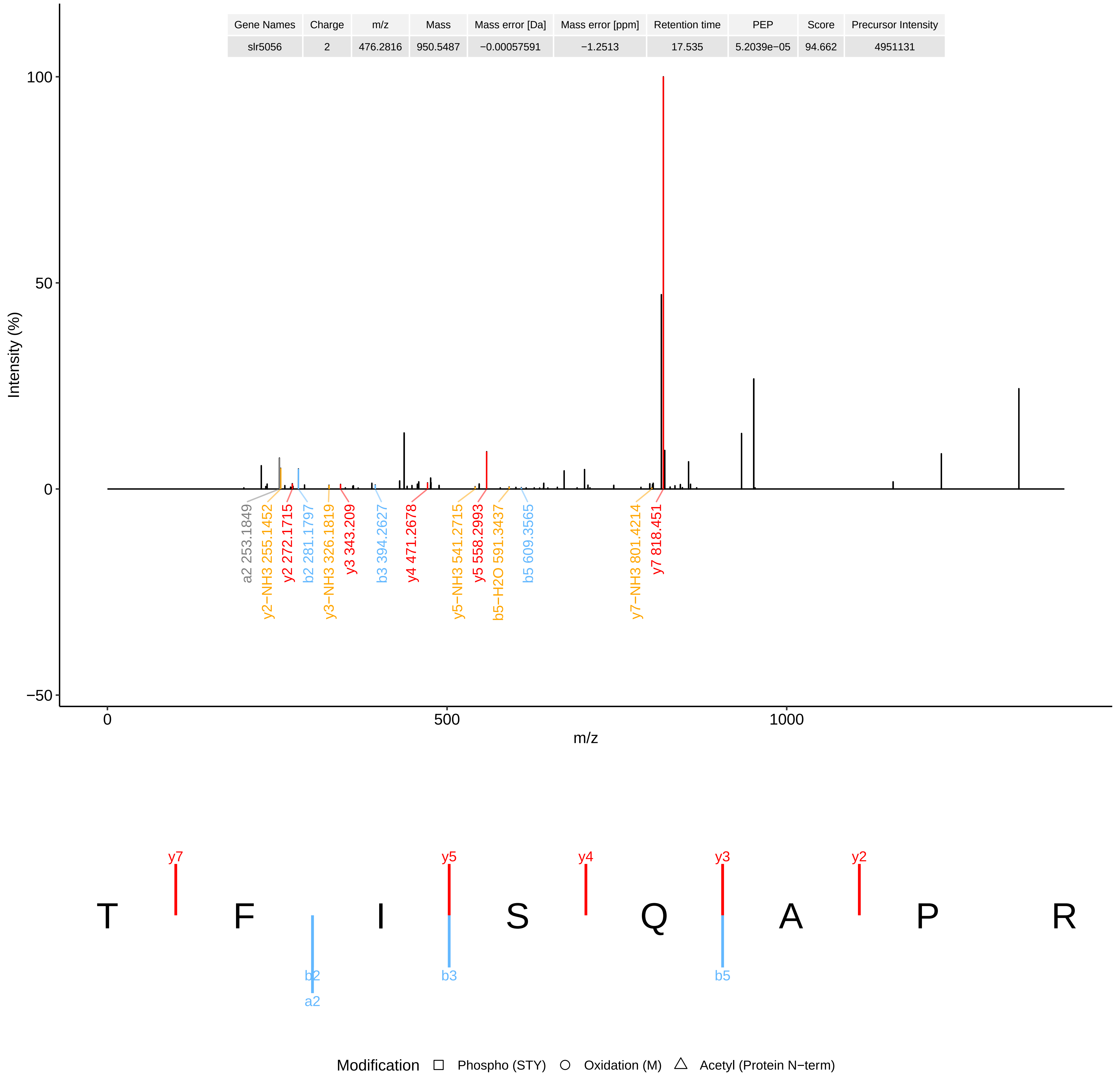

20211217_PS_SCy031_Proteome_R3_24hLC_F7 − Scan number: 15058

Gene Names	Charge	m/z	Mass	Mass error [Da]	Mass error [ppm]	Retention time	PEP	Score	Precursor Intensity
slr5101	2	620.8722	1239.73	NA	NA	26.589	0.00061475	79.116	NA

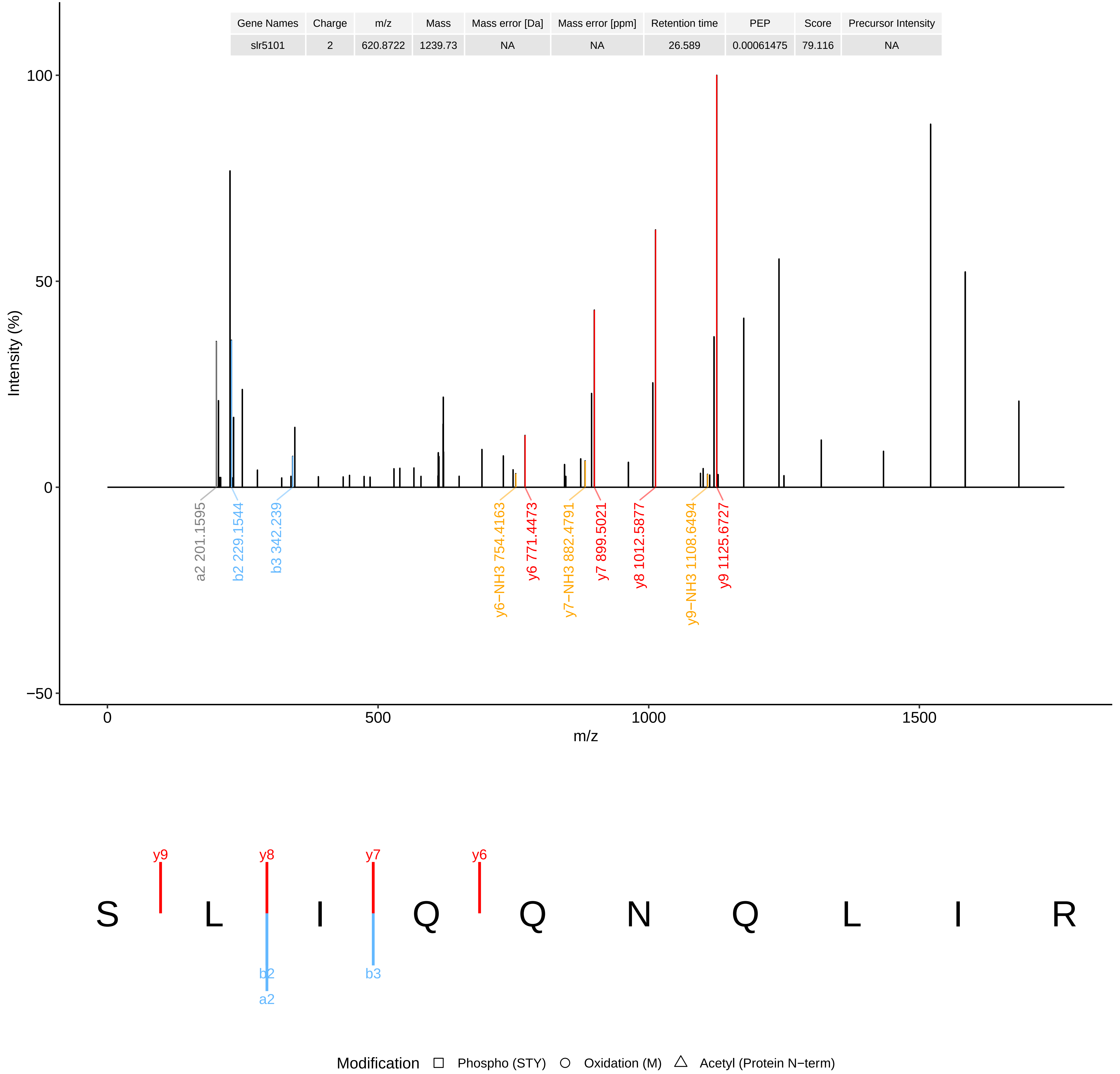

20211212_PS_SCy031_proteome_R1_24hLC_F6 − Scan number: 11301

Gene Names	Charge	m/z	Mass	Mass error [Da]	Mass error [ppm]	Retention time	PEP	Score	Precursor Intensity
sml0006	2	595.8641	1189.714	−0.00017467	−0.31204	19.691	1.2449e−06	99.802	10003246

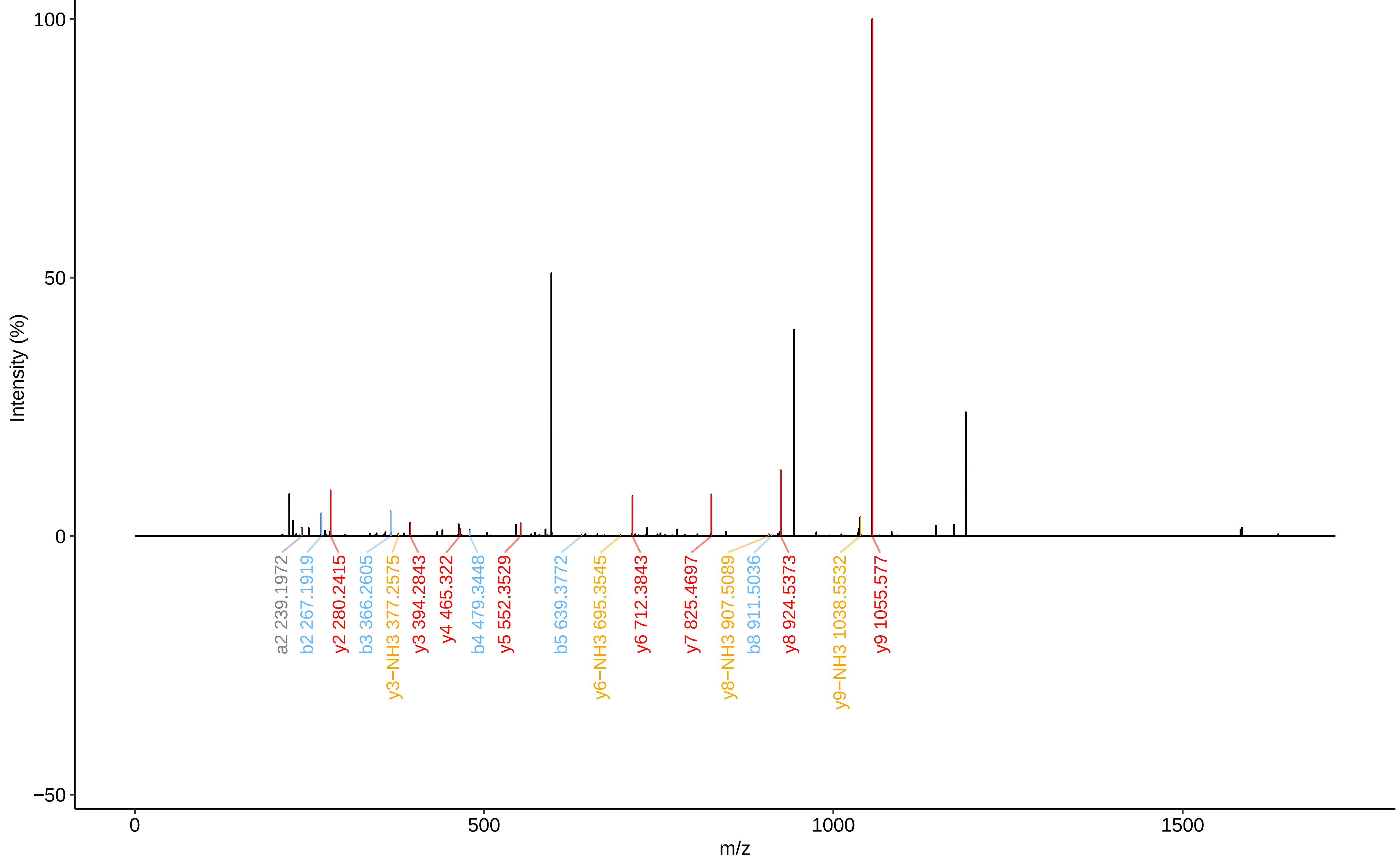

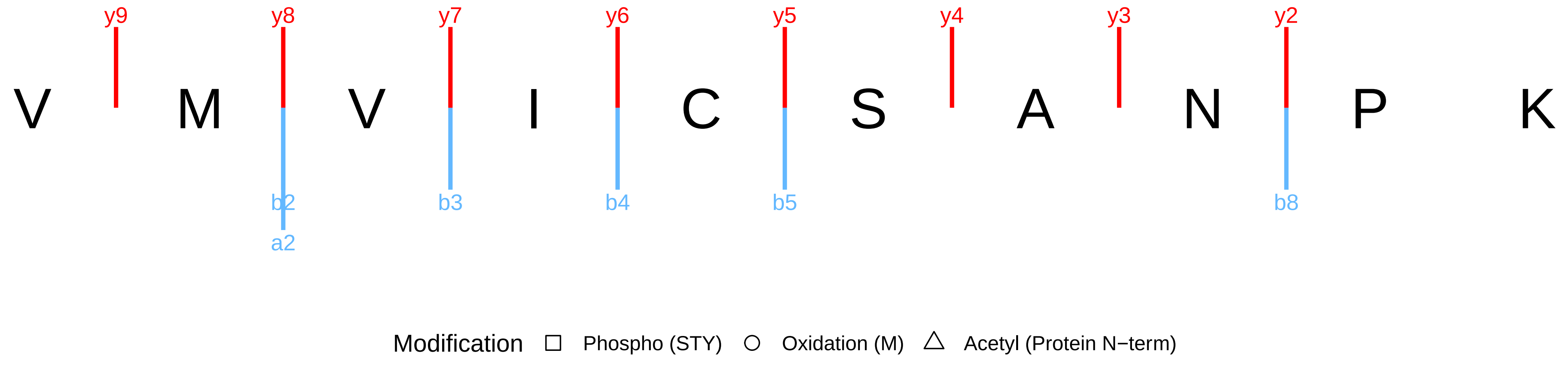

20211217_PS_SCy031_Proteome_R2_24hLC_F5 − Scan number: 35167

Gene Names	Charge	m/z	Mass	Mass error [Da]	Mass error [ppm]	Retention time	PEP	Score	Precursor Intensity
sml0008	2	588.808	1175.601	NA	NA	51.822	0.00056784	81.297	21902364

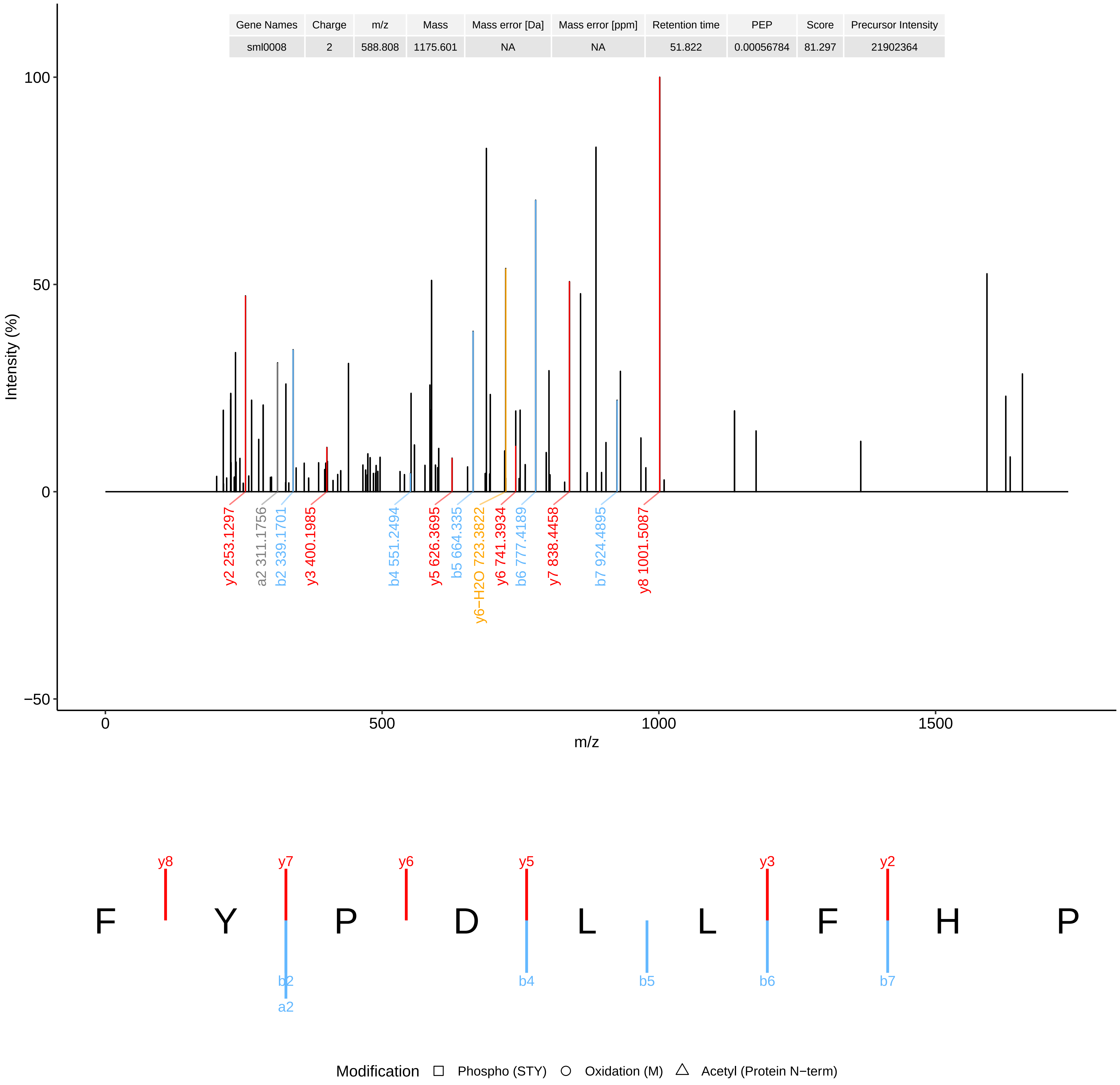

20211212_PS_SCy031_proteome_R1_3hLC_F2 − Scan number: 12967

Gene Names	Charge	m/z	Mass	Mass error [Da]	Mass error [ppm]	Retention time	PEP	Score	Precursor Intensity
smr0005	2	463.3126	924.6106	0.00016807	0.39339	20.592	0.00042683	72.138	131399096

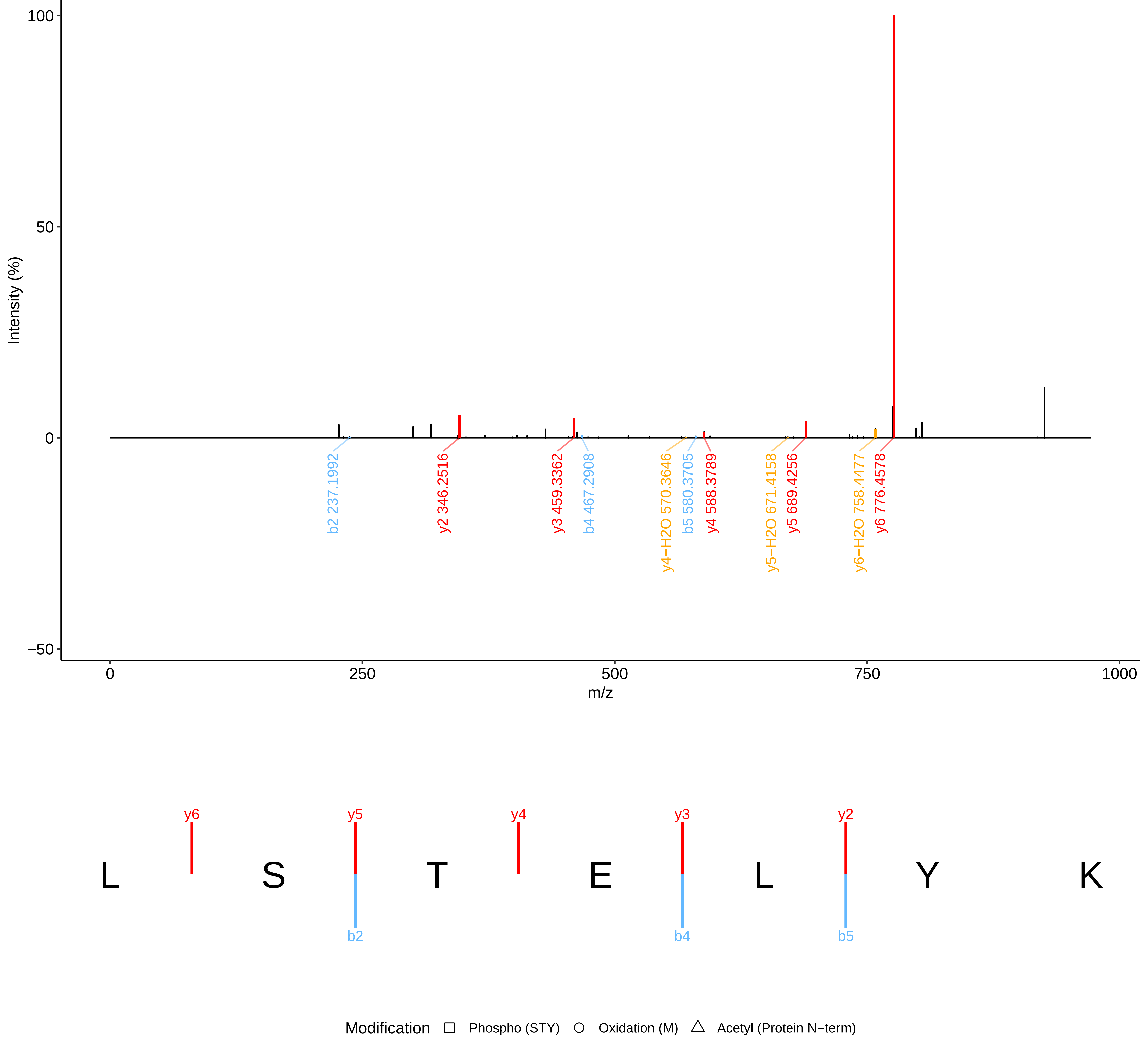

20211212_PS_SCy031_proteome_R1_3hLC_F8 − Scan number: 28634

Gene Names	Charge	m/z	Mass	Mass error [Da]	Mass error [ppm]	Retention time	PEP	Score	Precursor Intensity
smr0006	2	989.5384	1977.062	0.00027438	0.28185	43.285	1.2254e−264	338.14	36515936

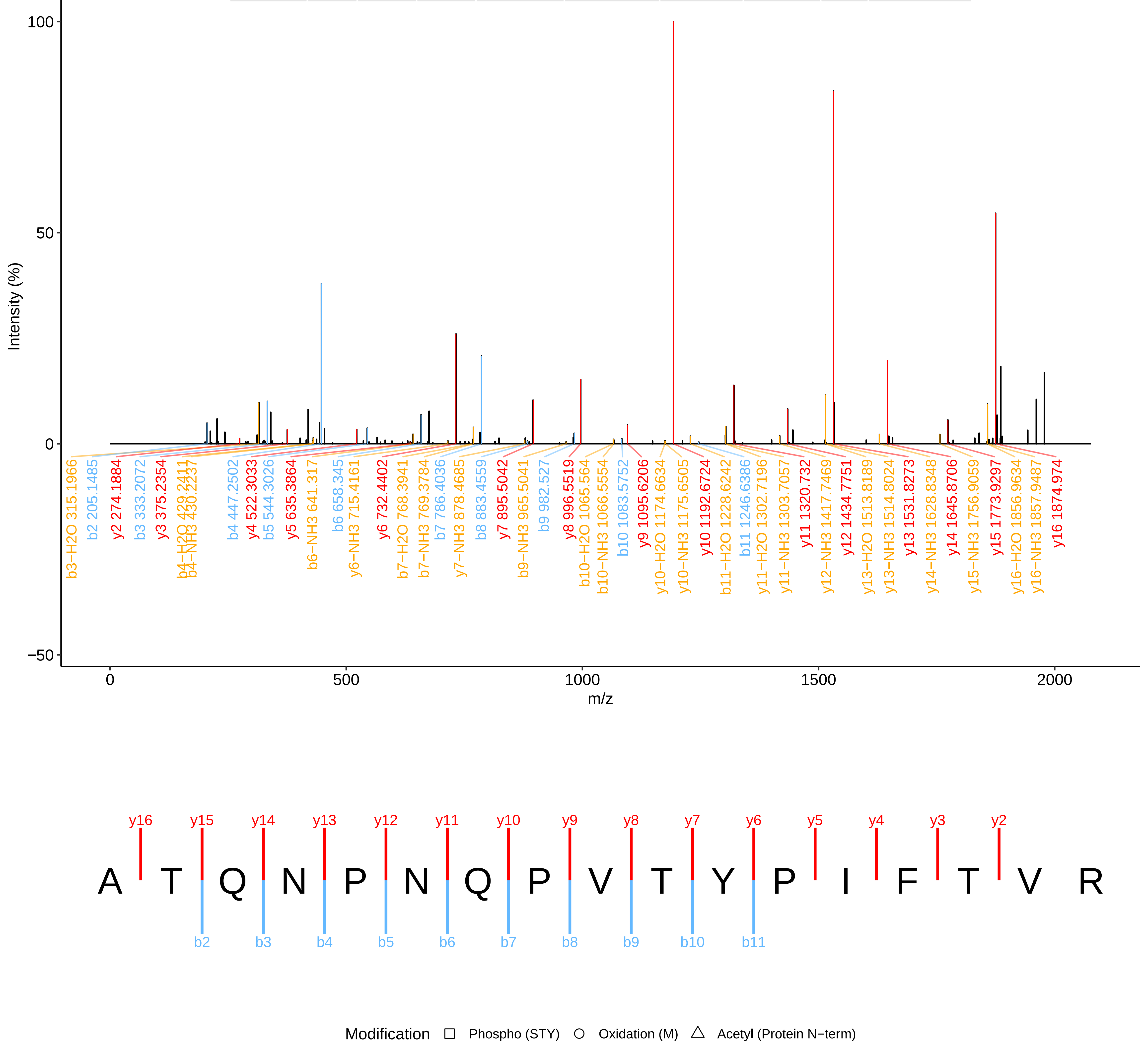

20211217_PS_SCy031_Proteome_R2_HC_F7 − Scan number: 4417

Gene Names	Charge	m/z	Mass	Mass error [Da]	Mass error [ppm]	Retention time	PEP	Score	Precursor Intensity
ssl0258	3	368.5652	1102.674	−7.8292e−05	−0.2255	10.43	0.00018276	65.305	1653547

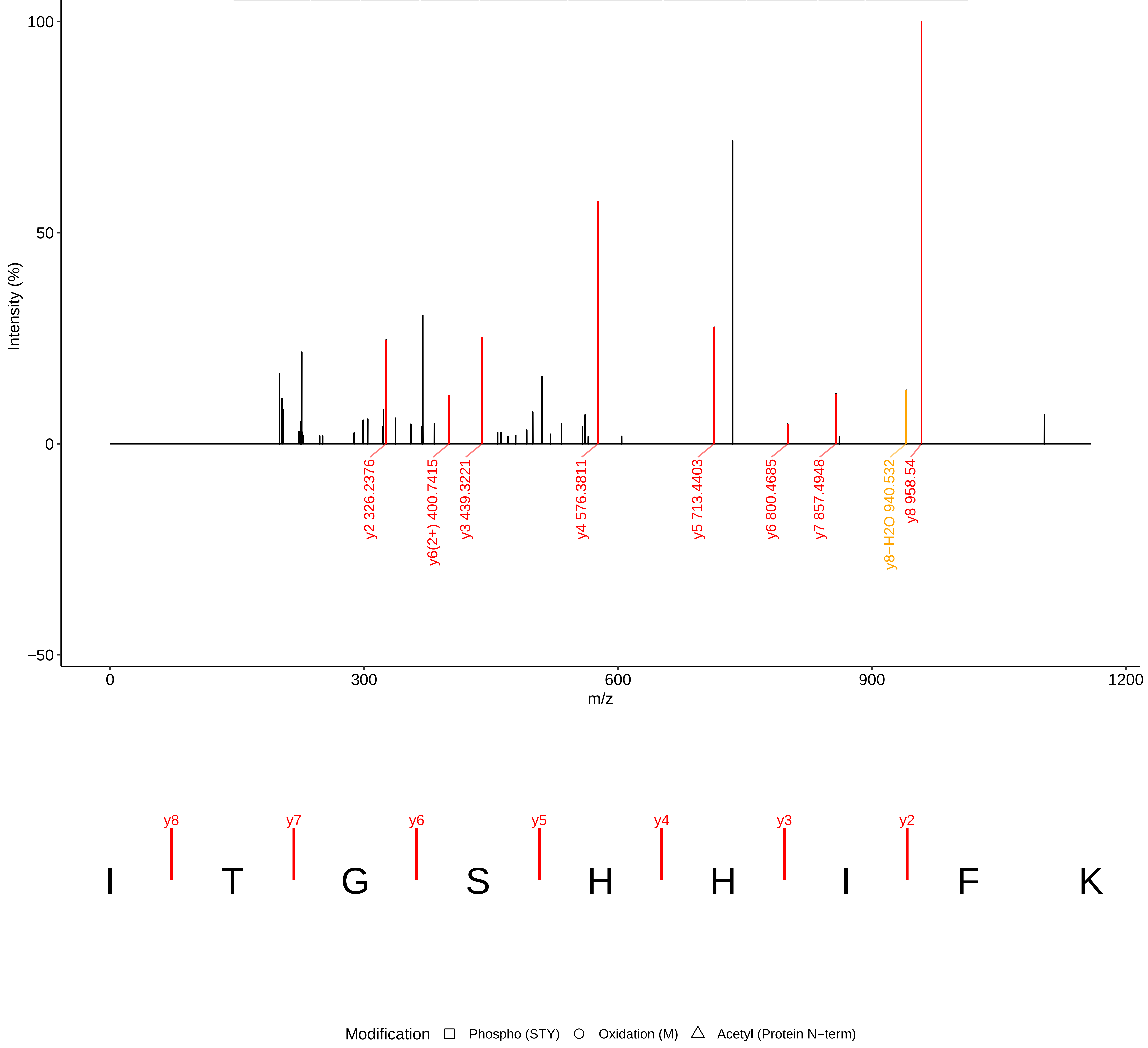

20211217_PS_SCy031_Proteome_R2_HC_F1 − Scan number: 12985

Gene Names	Charge	m/z	Mass	Mass error [Da]	Mass error [ppm]	Retention time	PEP	Score	Precursor Intensity
ssl0350	2	548.3346	1094.655	−0.00021804	−0.42564	20.046	9.7152e−08	107.9	12485100

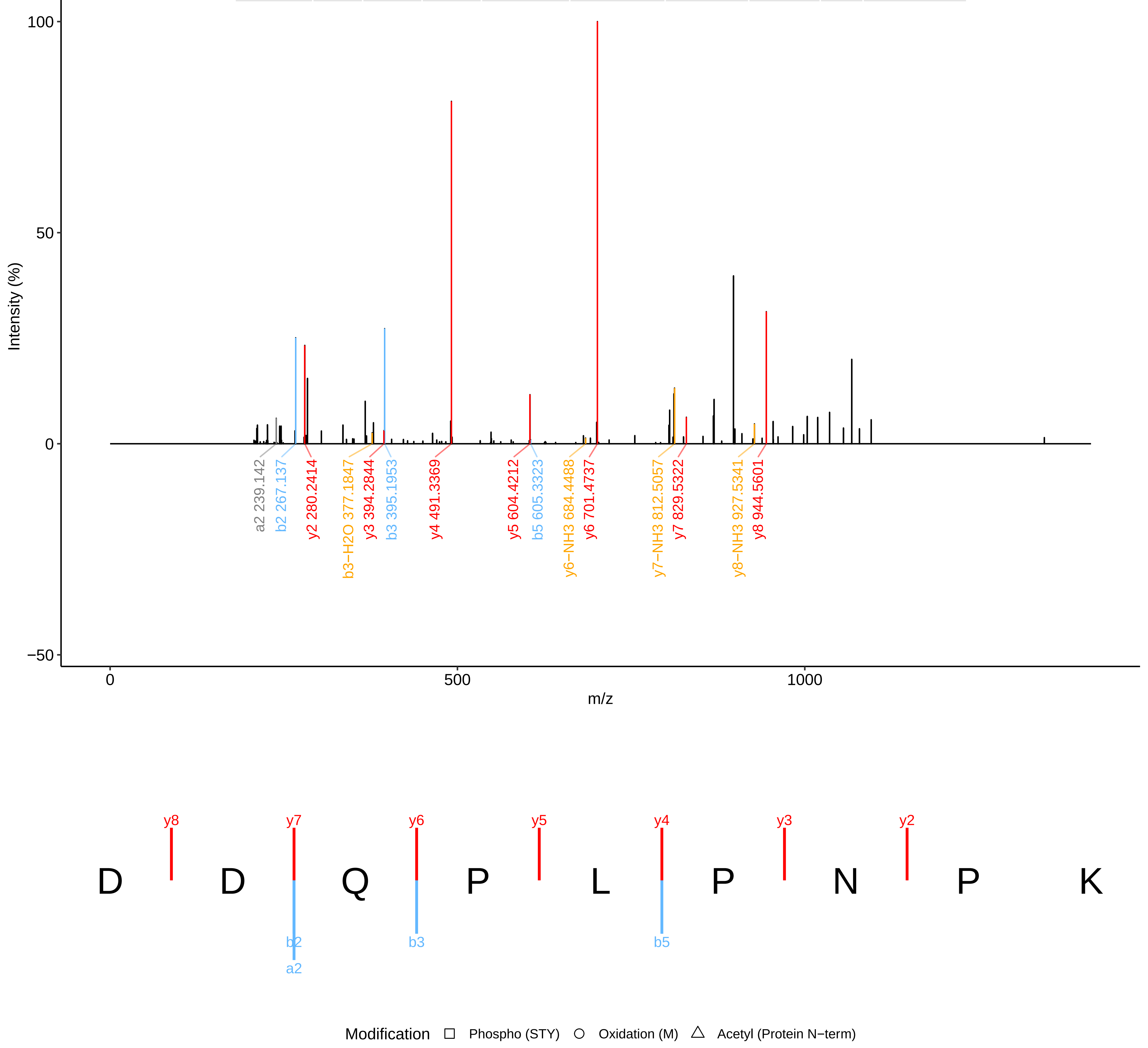

20211217_PS_SCy031_Proteome_R3_24hLC_F1 − Scan number: 8745

Gene Names	Charge	m/z	Mass	Mass error [Da]	Mass error [ppm]	Retention time	PEP	Score	Precursor Intensity
ssl1004	2	465.2487	928.4829	0.00041163	0.92043	14.65	0.00037839	75.213	5726639

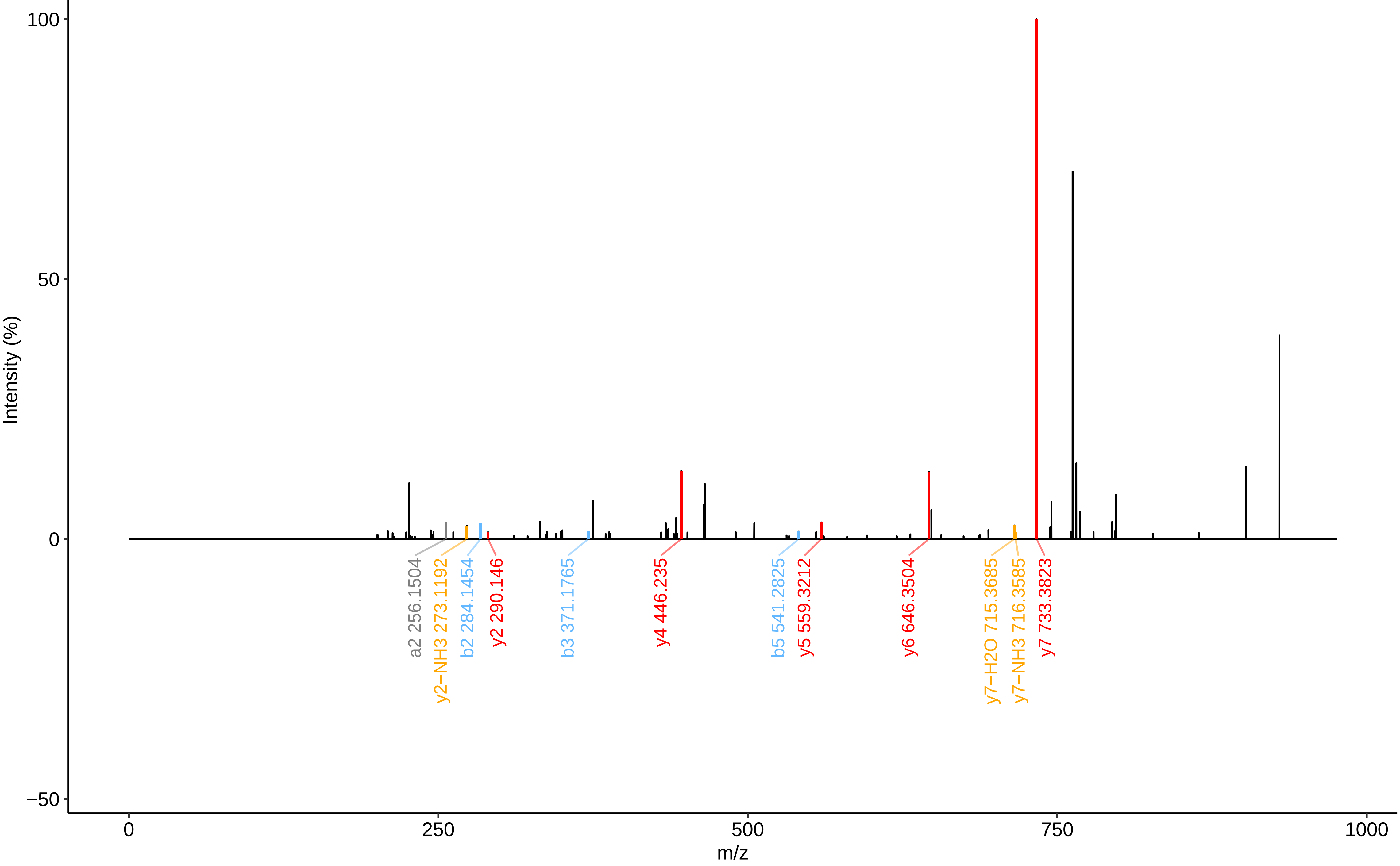

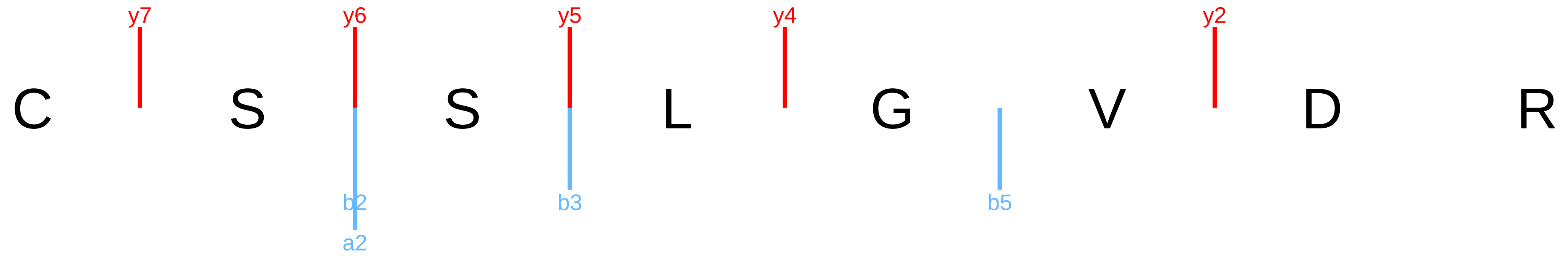

Modification □ Phospho (STY) ○ Oxidation (M) △ Acetyl (Protein N−term)

20211212_PS_SCy031_proteome_R1_3hLC_F1 − Scan number: 15100

Gene Names	Charge	m/z	Mass	Mass error [Da]	Mass error [ppm]	Retention time	PEP	Score	Precursor Intensity
ssl1046	2	591.353	1180.691	0.00034915	0.62878	22.682	9.2219e−06	94.309	107558440

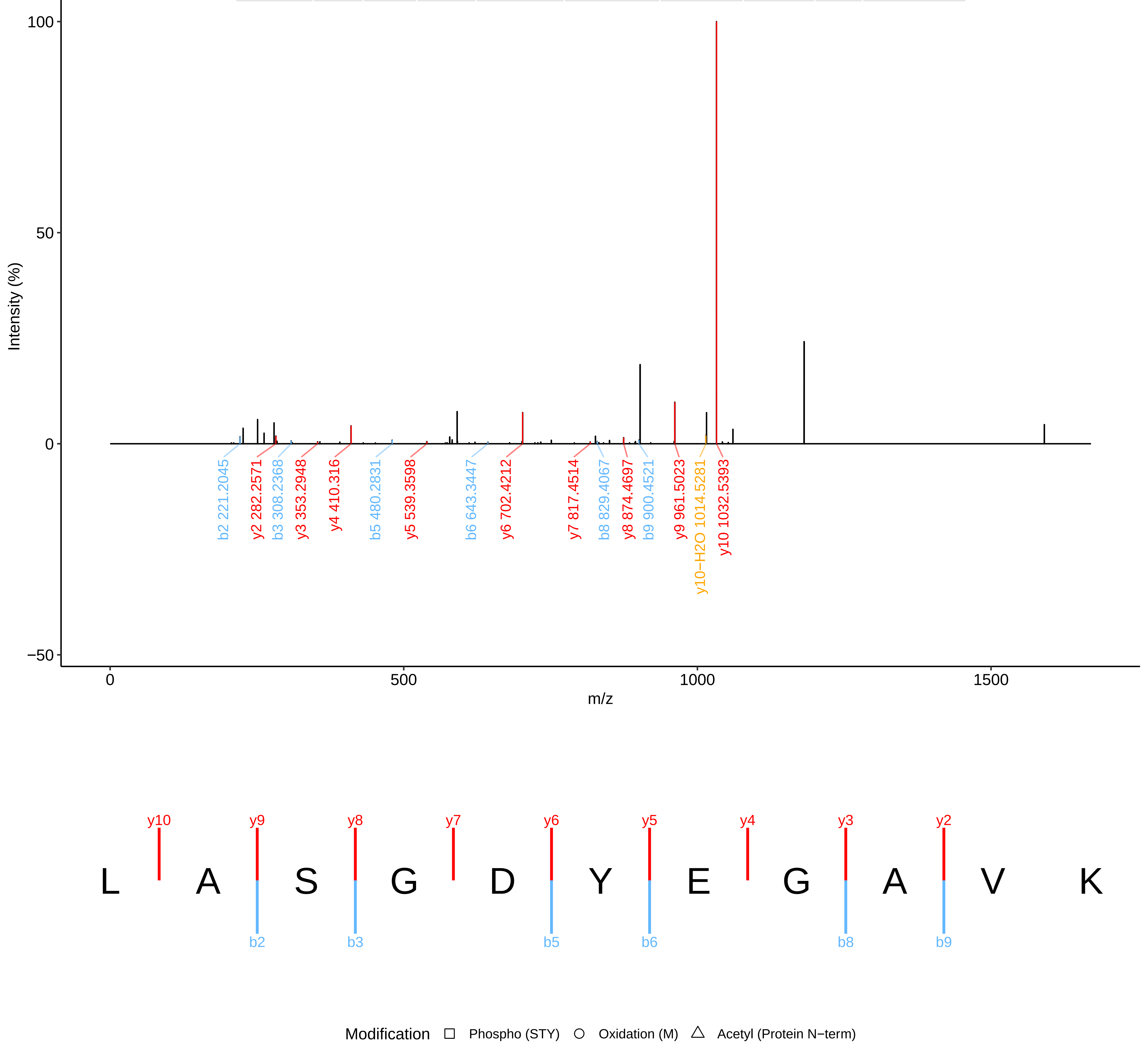

20211217_PS_SCy031_Proteome_R3_HC_F2 − Scan number: 11885

Gene Names	Charge	m/z	Mass	Mass error [Da]	Mass error [ppm]	Retention time	PEP	Score	Precursor Intensity
ssl1263	2	482.2922	962.5698	6.5356e−05	0.14017	19.342	7.3855e−18	167.9	26068356

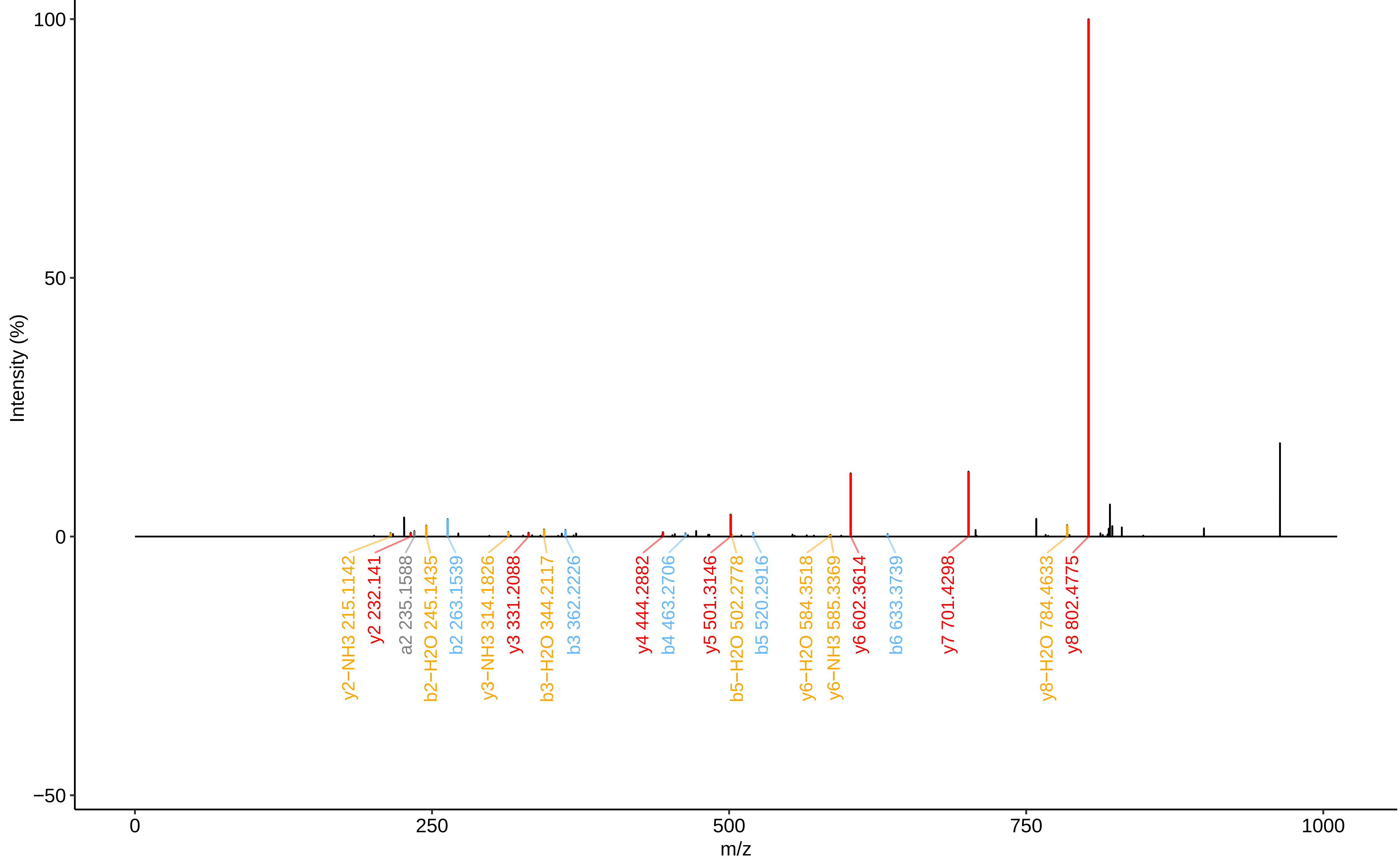

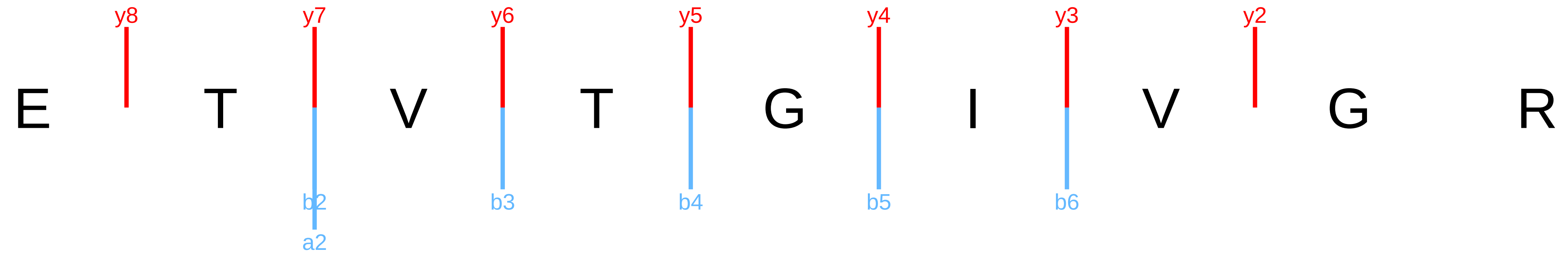

Modification □ Phospho (STY) ○ Oxidation (M) △ Acetyl (Protein N−term)

20211217_PS_SCy031_Proteome_R3_HC_F2 − Scan number: 11890

Gene Names	Charge	m/z	Mass	Mass error [Da]	Mass error [ppm]	Retention time	PEP	Score	Precursor Intensity
ssl1493	2	495.3	988.5855	NA	NA	19.348	0.00025565	102	7902506

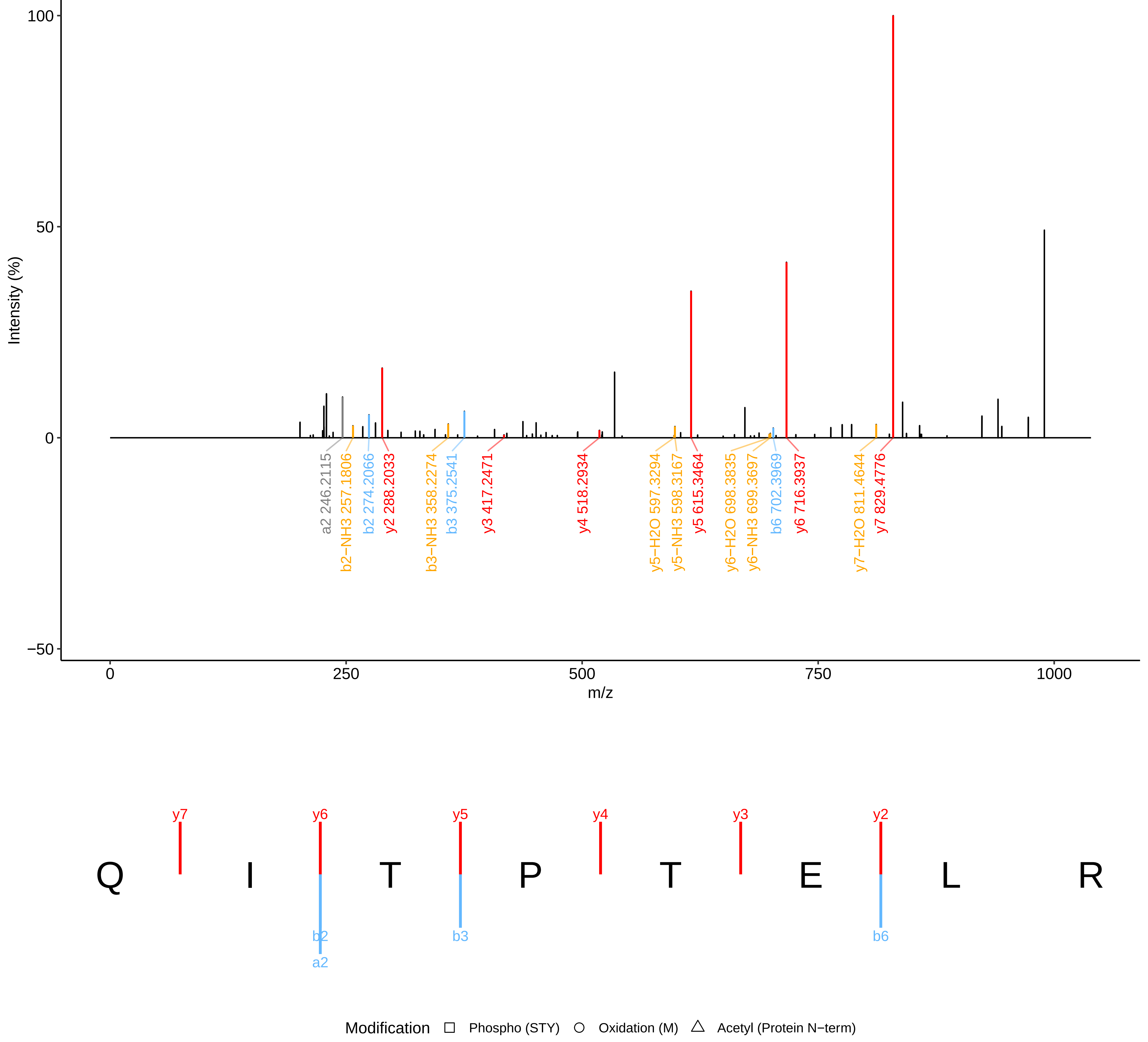

20211217_PS_SCy031_Proteome_R2_3hLC_F8 − Scan number: 29900

Gene Names	Charge	m/z	Mass	Mass error [Da]	Mass error [ppm]	Retention time	PEP	Score	Precursor Intensity
ssl1633	2	772.8677	1543.721	−0.00010692	−0.1409	51.184	2.5076e−133	274.31	55131480

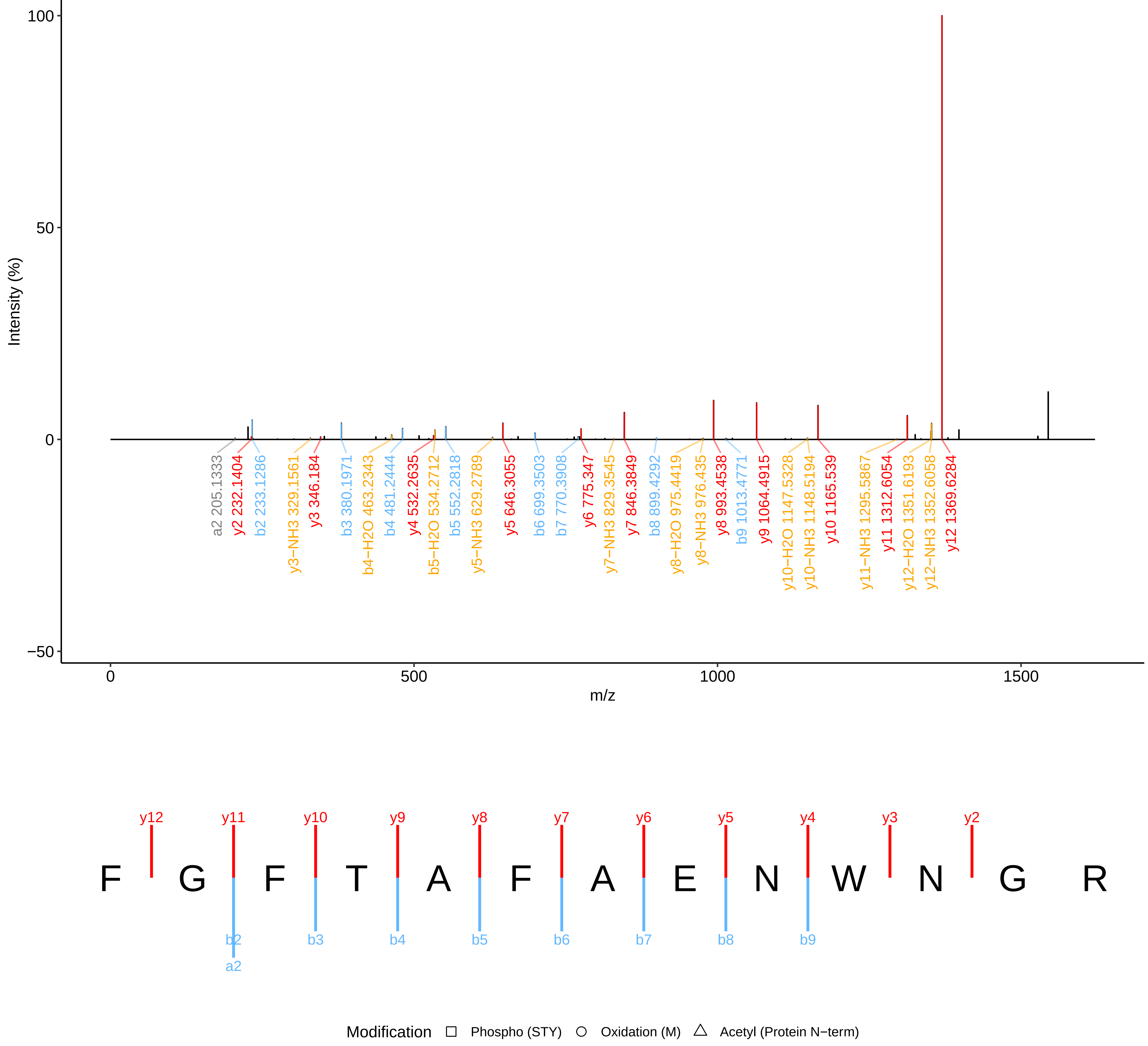

20211217_PS_SCy031_Proteome_R2_3hLC_F7 − Scan number: 18199

Gene Names	Charge	m/z	Mass	Mass error [Da]	Mass error [ppm]	Retention time	PEP	Score	Precursor Intensity
ssl1707	2	598.8355	1195.657	0.00010677	0.18383	29.062	1.683e−06	103.83	15460231

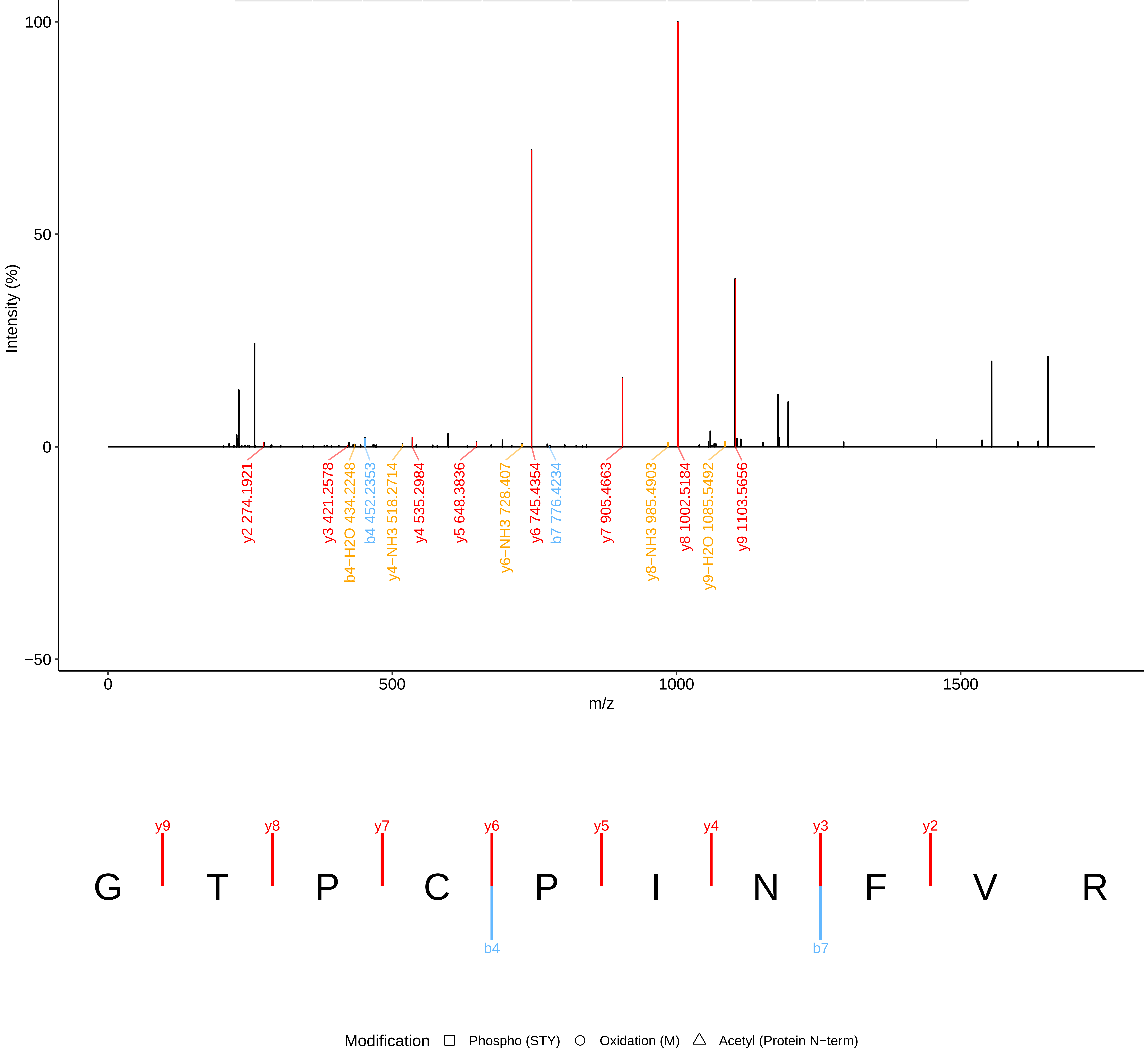

20211217_PS_SCy031_Proteome_R3_HC_F6 − Scan number: 15706

Gene Names	Charge	m/z	Mass	Mass error [Da]	Mass error [ppm]	Retention time	PEP	Score	Precursor Intensity
ssl2823	3	394.9429	1181.807	NA	NA	26.83	0.00070794	69.451	2373002

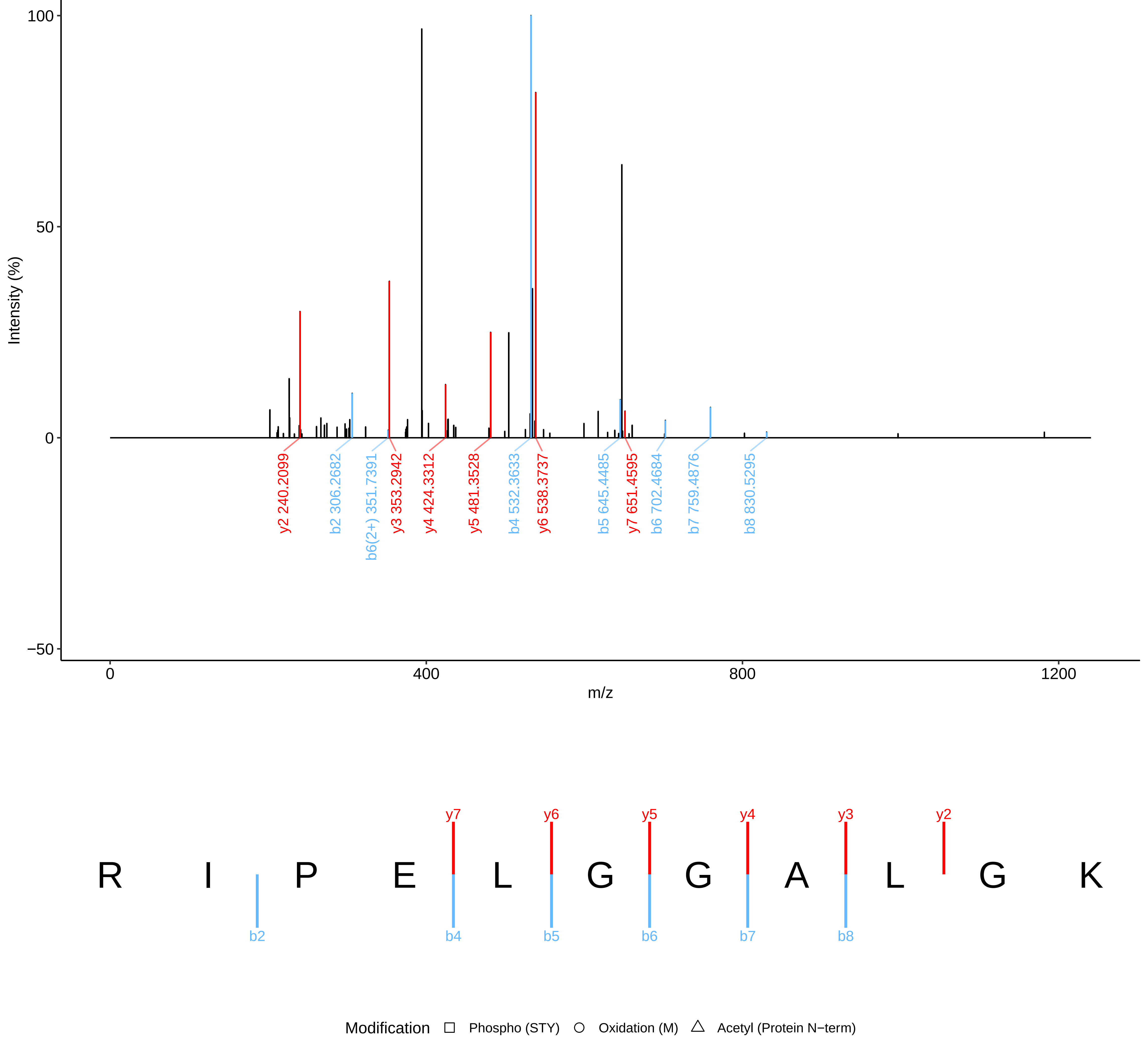

20211217_PS_SCy031_Proteome_R2_24hLC_F9 − Scan number: 26480

Gene Names	Charge	m/z	Mass	Mass error [Da]	Mass error [ppm]	Retention time	PEP	Score	Precursor Intensity
ssl3291	2	472.7682	943.5218	5.0687e−05	0.11098	43.613	3.3306e−29	185.25	4720567

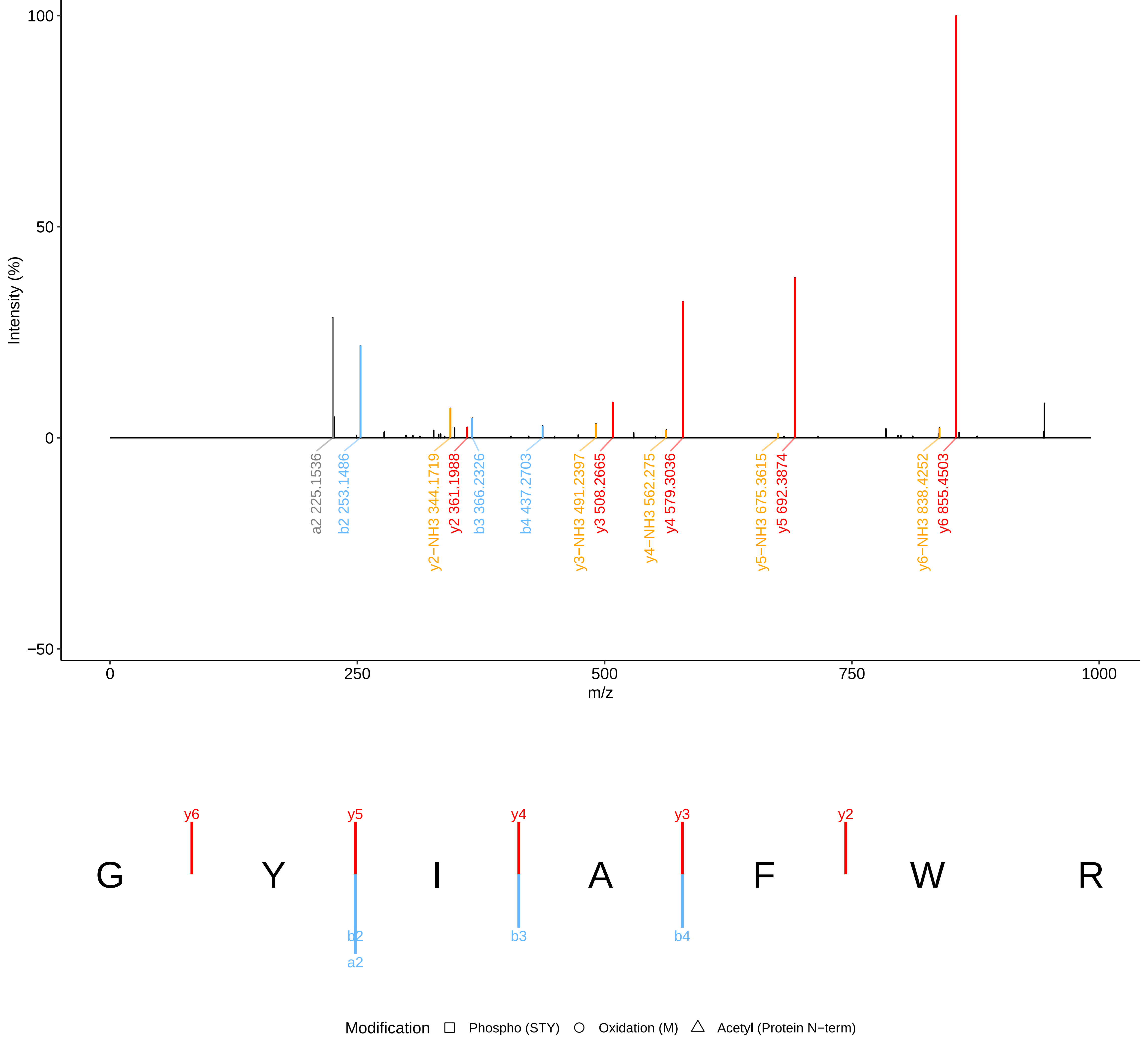

20211217_PS_SCy031_Proteome_R2_3hLC_F1 − Scan number: 33874

Gene Names	Charge	m/z	Mass	Mass error [Da]	Mass error [ppm]	Retention time	PEP	Score	Precursor Intensity
ssl3379	2	576.8429	1151.671	−0.00036455	−0.66917	47.119	2.6753e−07	134.25	17541828

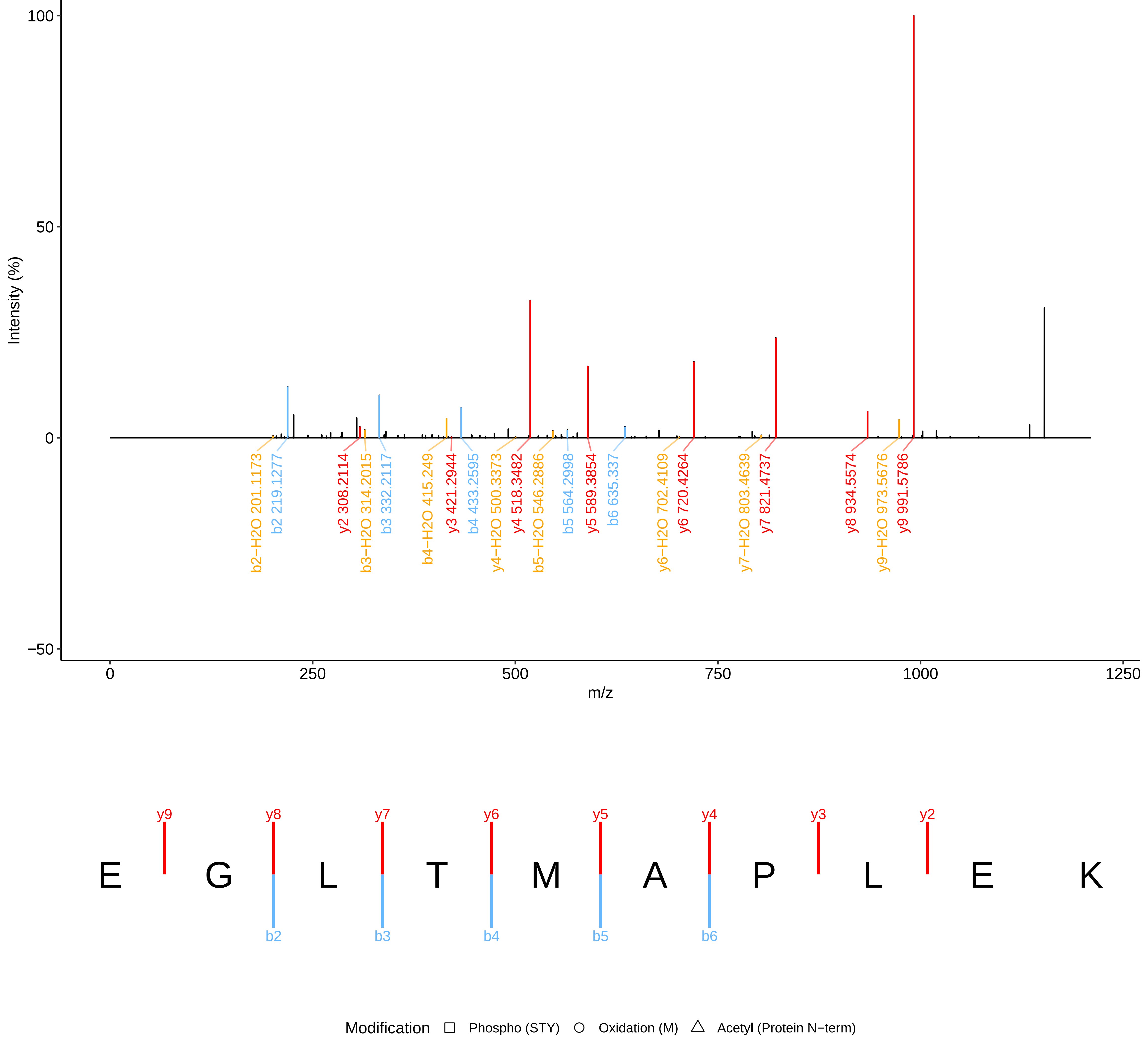

20211217_PS_SCy031_Proteome_R3_24hLC_F4 − Scan number: 14702

Gene Names	Charge	m/z	Mass	Mass error [Da]	Mass error [ppm]	Retention time	PEP	Score	Precursor Intensity
ssl6092	2	441.2708	880.527	0.00025114	0.60775	24.912	0.00027407	79.451	4232459

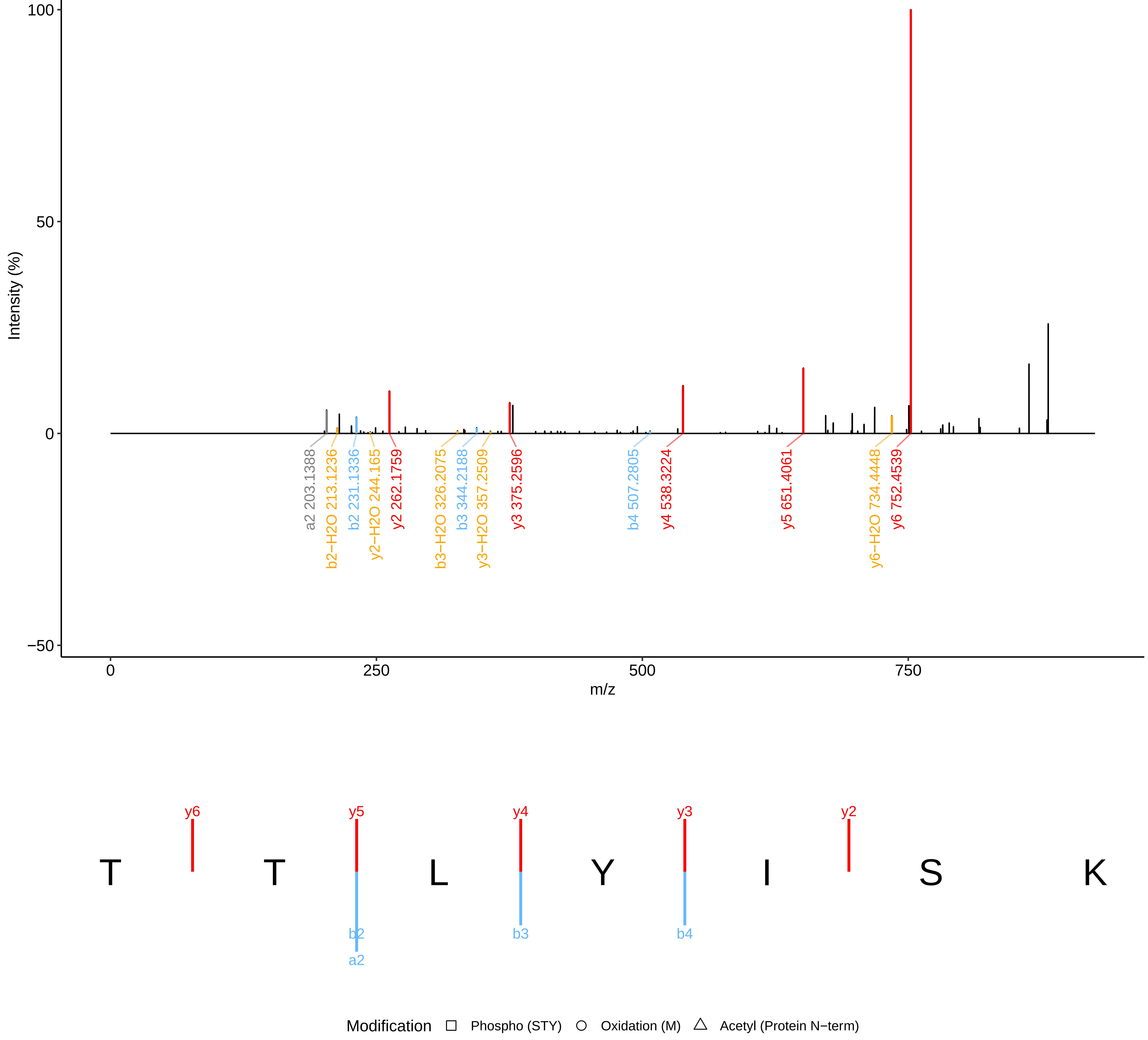

20211217_PS_SCy031_Proteome_R3_HC_F2 − Scan number: 36435

Gene Names	Charge	m/z	Mass	Mass error [Da]	Mass error [ppm]	Retention time	PEP	Score	Precursor Intensity
ssr0109	3	902.4508	2704.331	−0.00024787	−0.28047	52.588	5.7632e−32	127.35	8291516

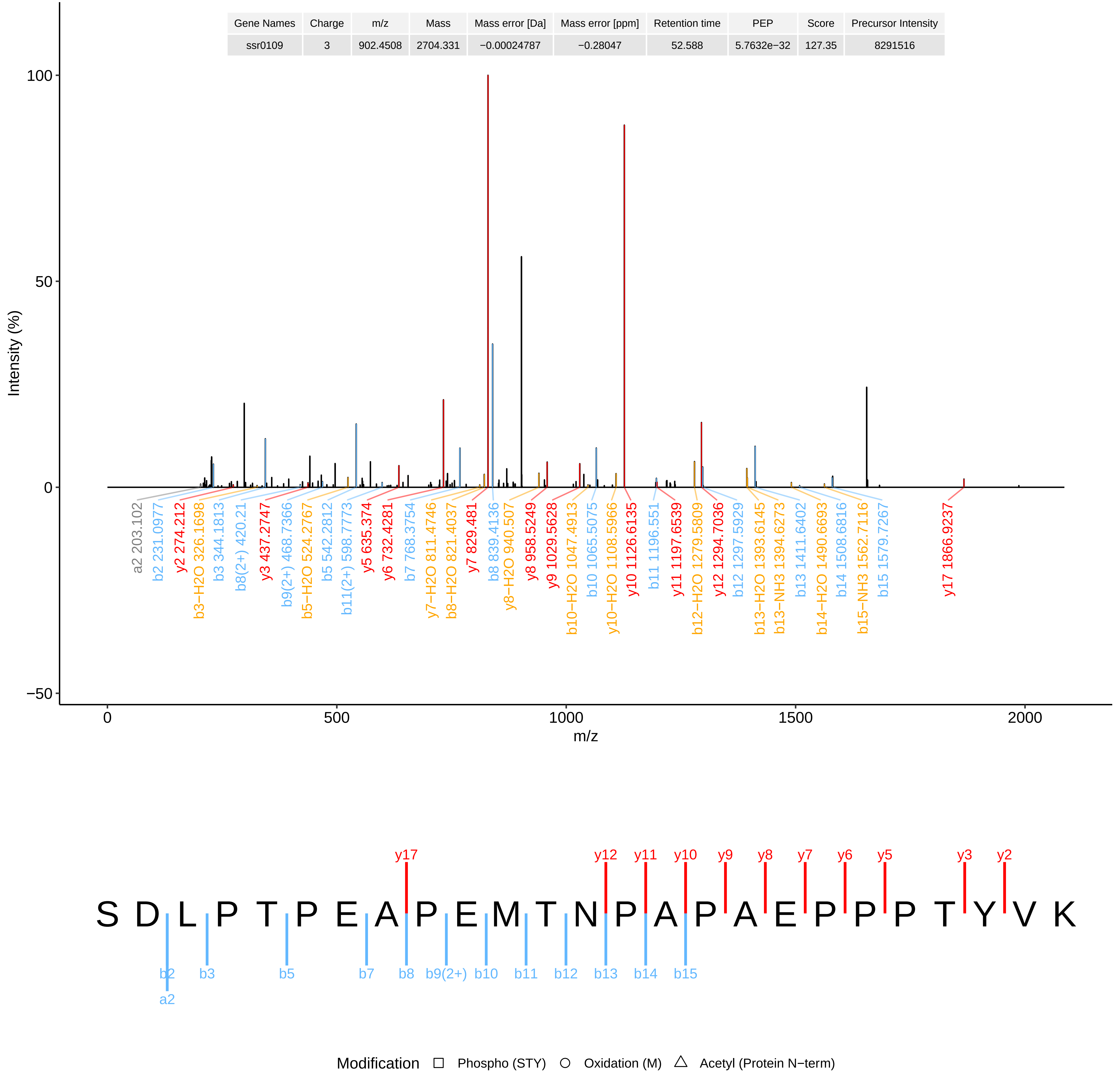

20211212_PS_SCy031_proteome_R1_HC_F5 − Scan number: 25441

Gene Names	Charge	m/z	Mass	Mass error [Da]	Mass error [ppm]	Retention time	PEP	Score	Precursor Intensity
ssr1375	4	737.1504	2944.573	4.2079e−05	0.058515	38.201	1.0379e−61	158.2	NA

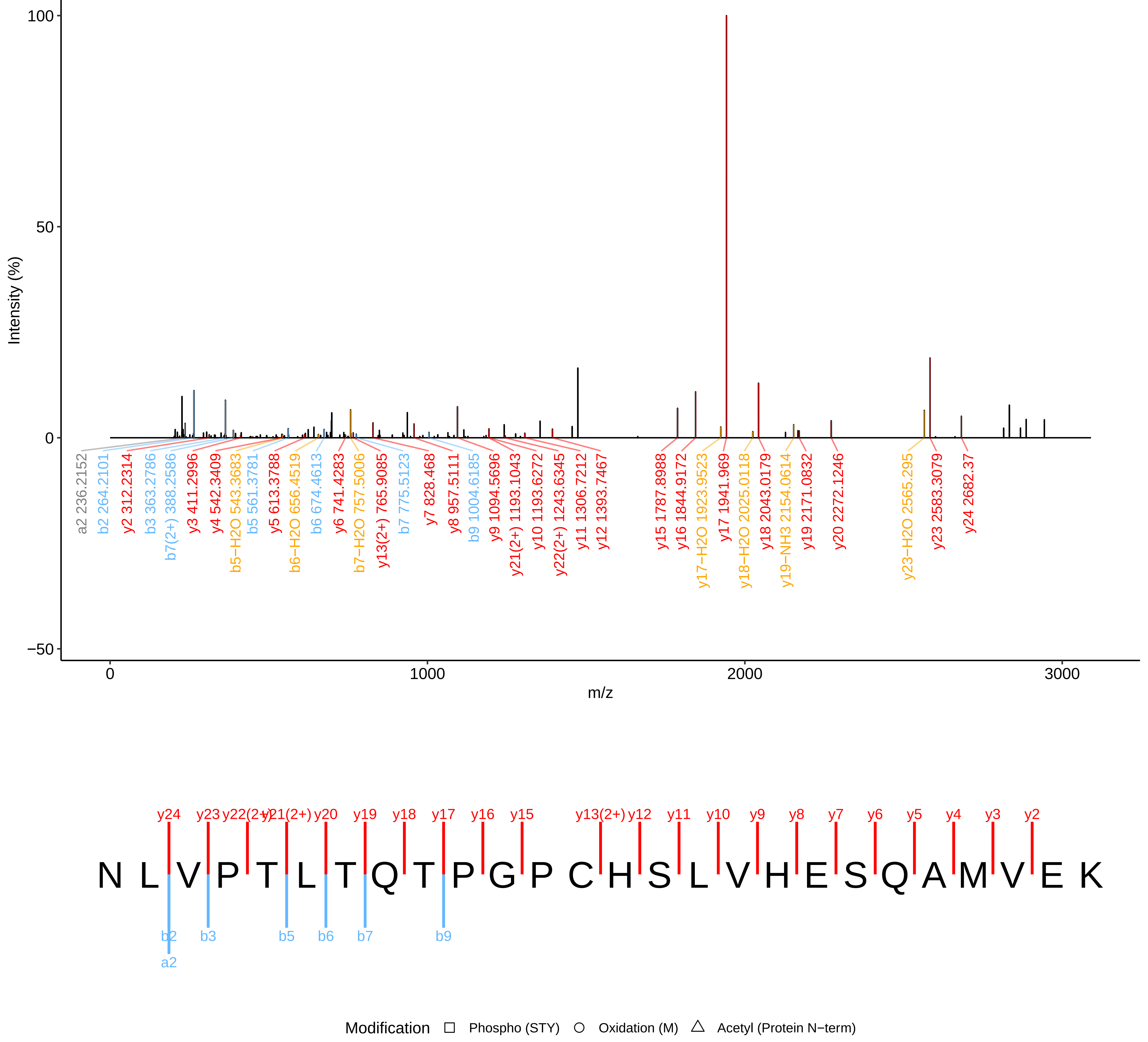

20211217_PS_SCy031_Proteome_R3_3hLC_F7 − Scan number: 17134

Gene Names	Charge	m/z	Mass	Mass error [Da]	Mass error [ppm]	Retention time	PEP	Score	Precursor Intensity
ssr1558	2	629.9188	1257.823	0.00019452	0.32757	28.68	9.2718e−06	102.66	2241086

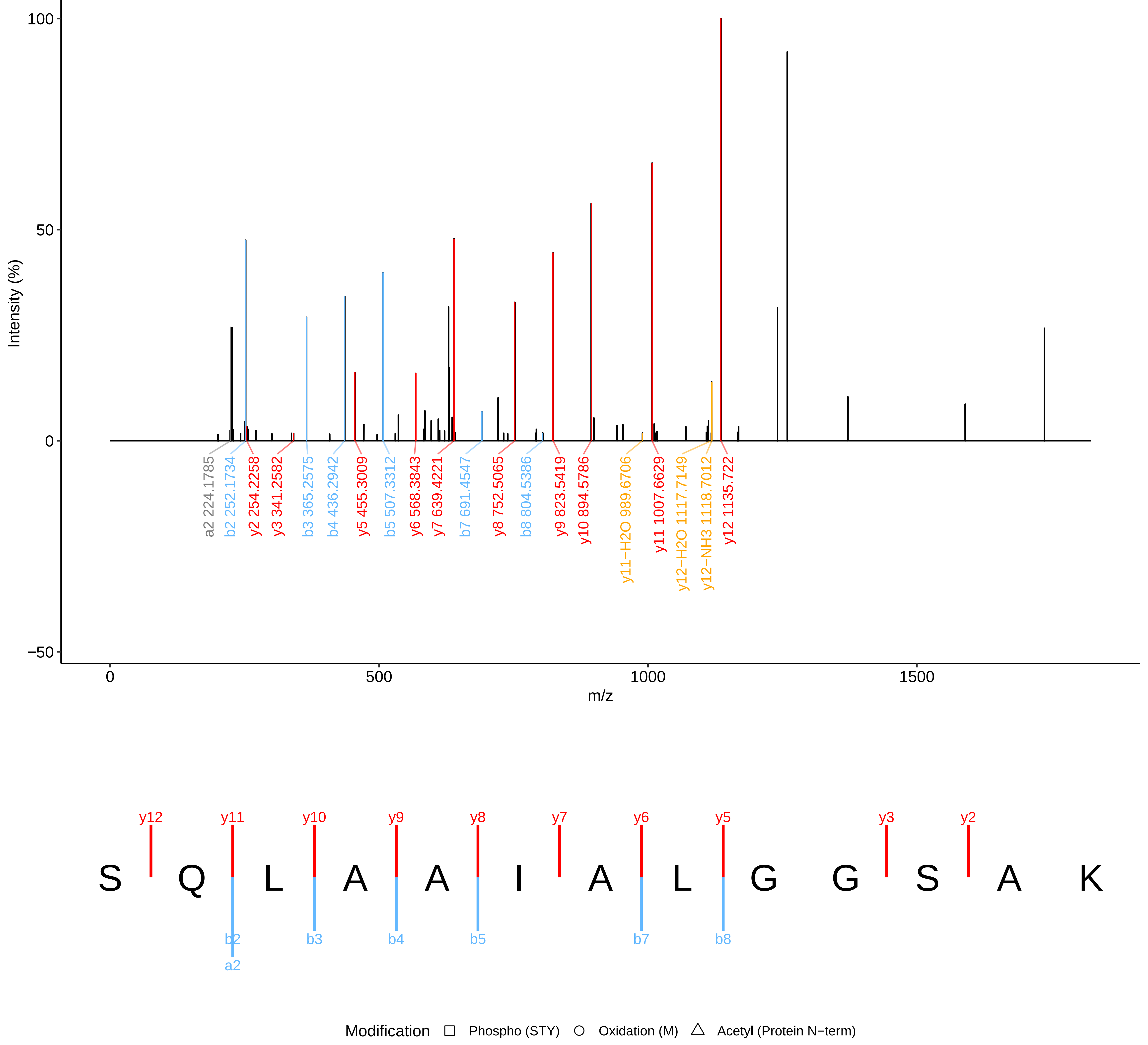

20211217_PS_SCy031_Proteome_R3_3hLC_F1 − Scan number: 25299

Gene Names	Charge	m/z	Mass	Mass error [Da]	Mass error [ppm]	Retention time	PEP	Score	Precursor Intensity
ssr2067	2	598.8626	1195.711	−7.0579e−05	−0.12151	36.368	6.0916e−06	88.136	3924170

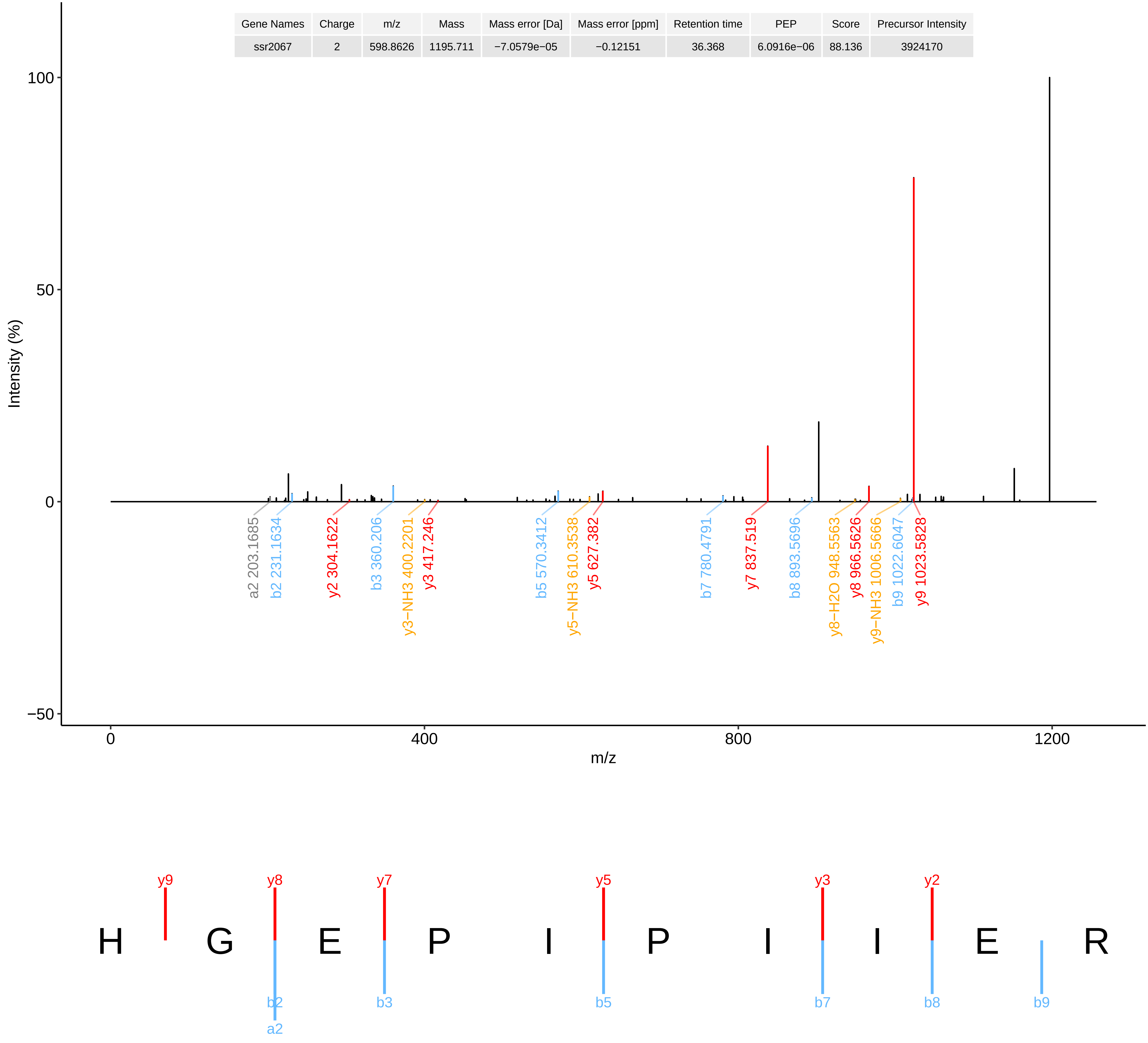

Modification □ Phospho (STY) ○ Oxidation (M) △ Acetyl (Protein N−term)

20211217_PS_SCy031_Proteome_R3_24hLC_F7 − Scan number: 9546

Gene Names	Charge	m/z	Mass	Mass error [Da]	Mass error [ppm]	Retention time	PEP	Score	Precursor Intensity
ssr2439	3	638.9674	1913.88	−0.00022027	−0.34456	18.882	1.9395e−07	136.01	2076681

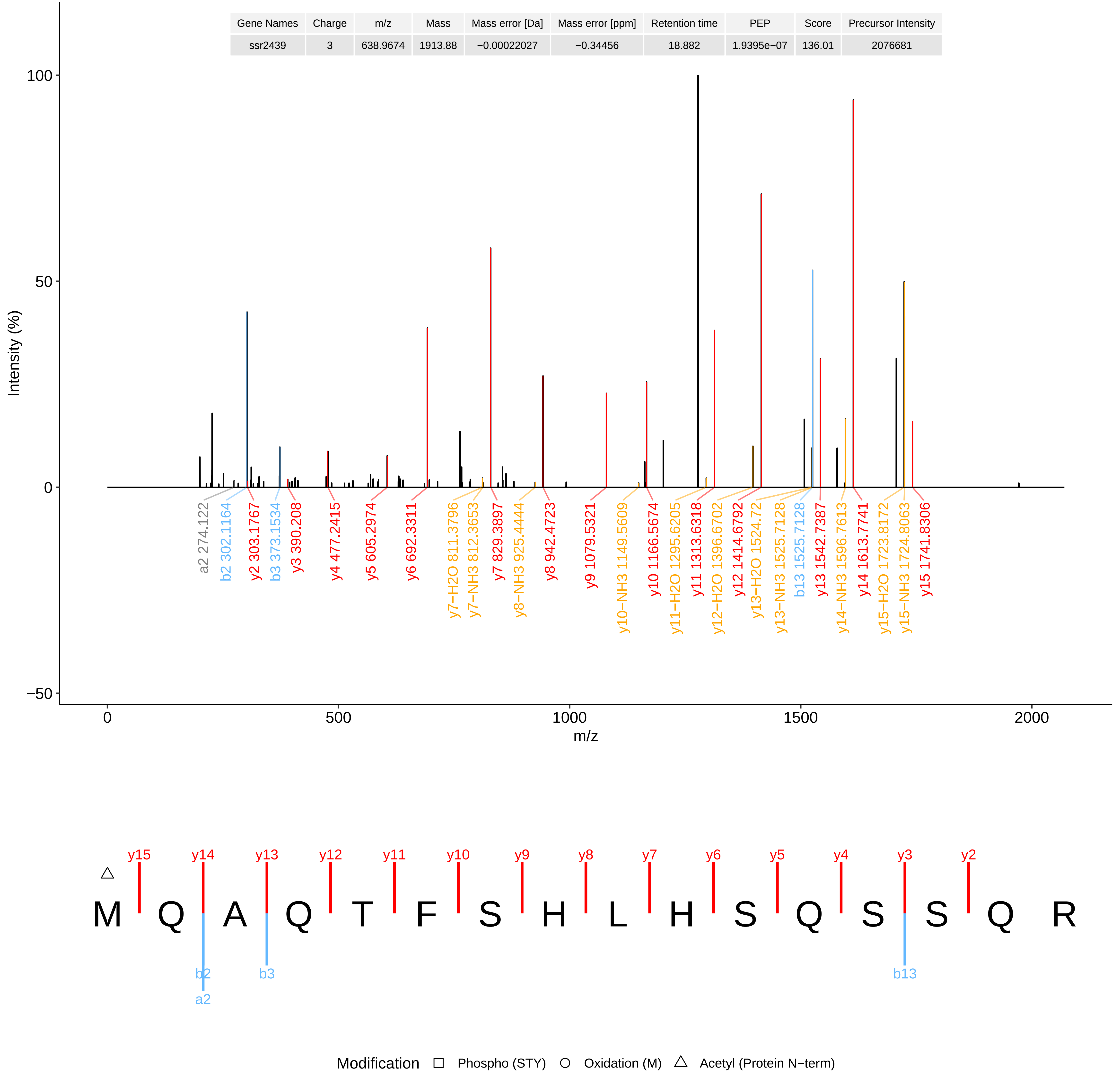

20211217_PS_SCy031_Proteome_R2_HC_F1 − Scan number: 17477

Gene Names	Charge	m/z	Mass	Mass error [Da]	Mass error [ppm]	Retention time	PEP	Score	Precursor Intensity
ssr2723	2	654.329	1306.643	−9.4513e−05	−0.14854	25.824	1.6565e−22	182.69	8471097

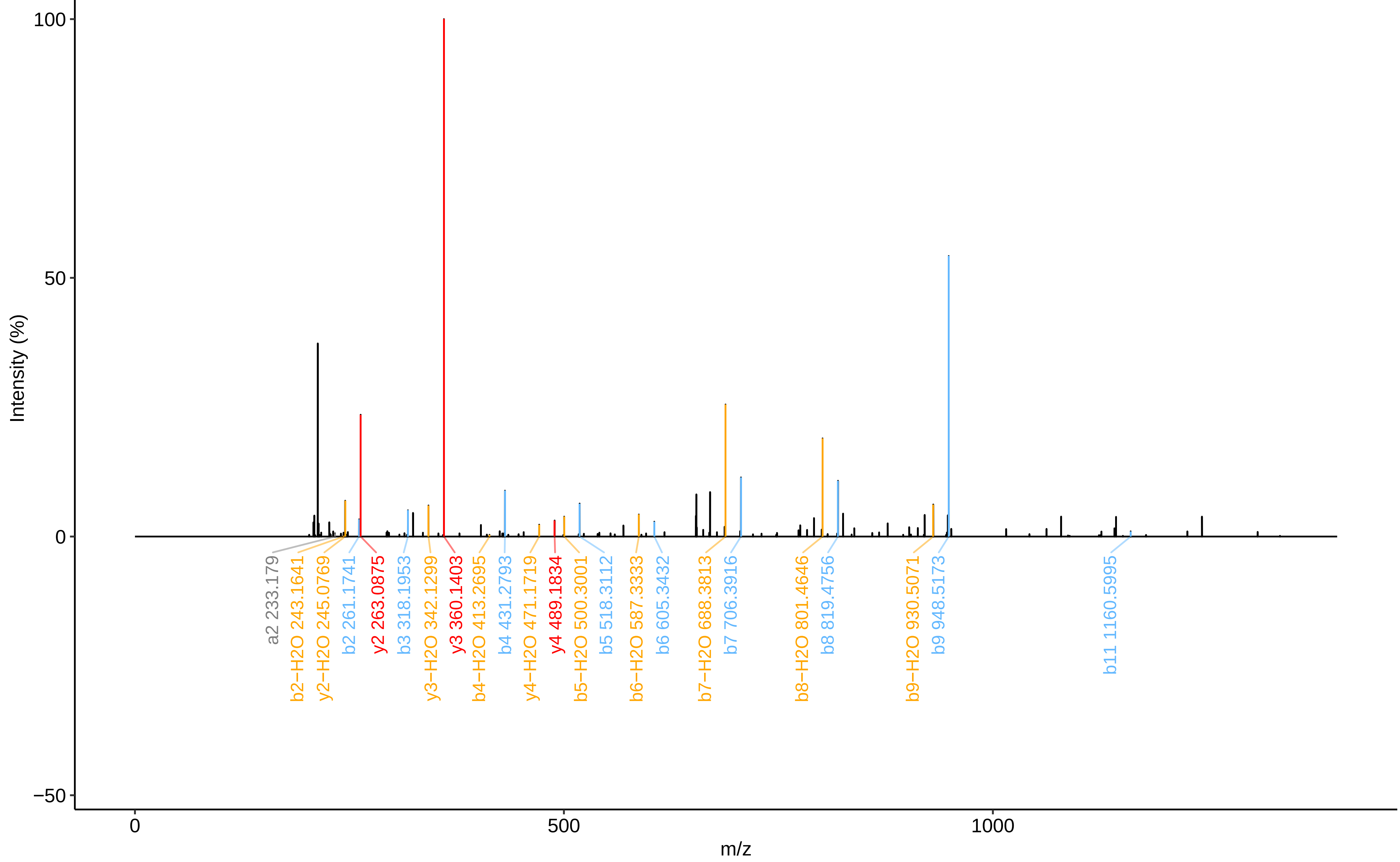

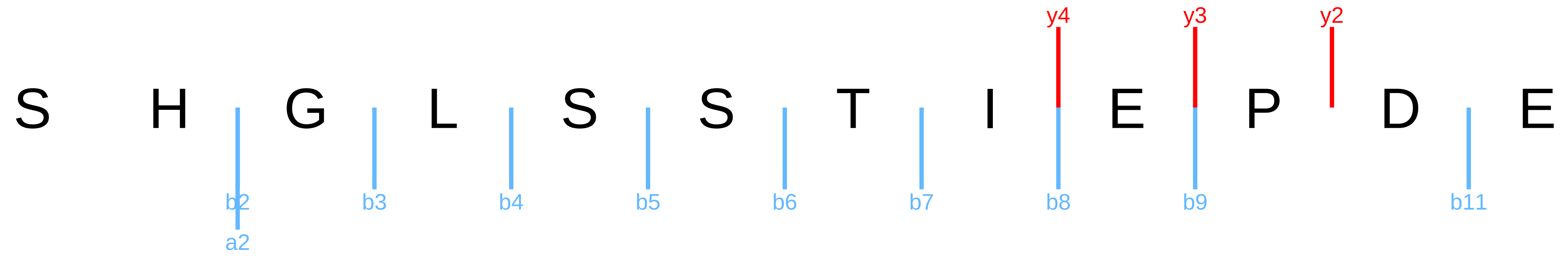

Modification □ Phospho (STY) ○ Oxidation (M) △ Acetyl (Protein N−term)

20211217_PS_SCy031_Proteome_R2_3hLC_F3 − Scan number: 38607

Gene Names	Charge	m/z	Mass	Mass error [Da]	Mass error [ppm]	Retention time	PEP	Score	Precursor Intensity
ssr3000	3	750.4239	2248.25	−6.7935e−05	−0.093527	53.679	5.9178e−19	130.38	6075015

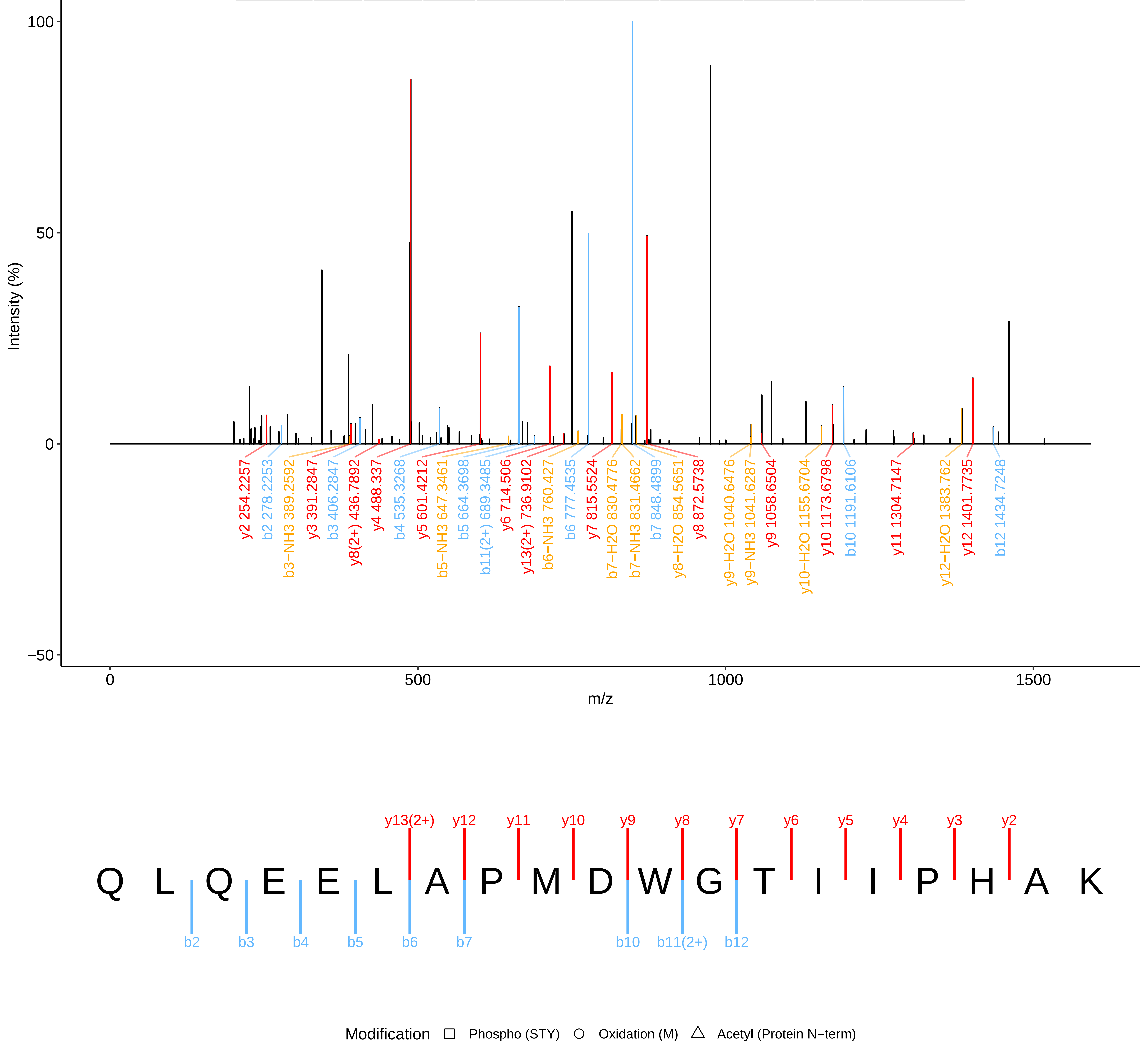

20210823_PS_SCy031_R1_phospho_HC_TiO2_1 − Scan number: 4737

Gene Names	Charge	m/z	Mass	Mass error [Da]	Mass error [ppm]	Retention time	PEP	Score	Precursor Intensity
ssr3189	1	1639.709	1638.702	0.0024371	1.516	8.7813	0	379.86	46263264

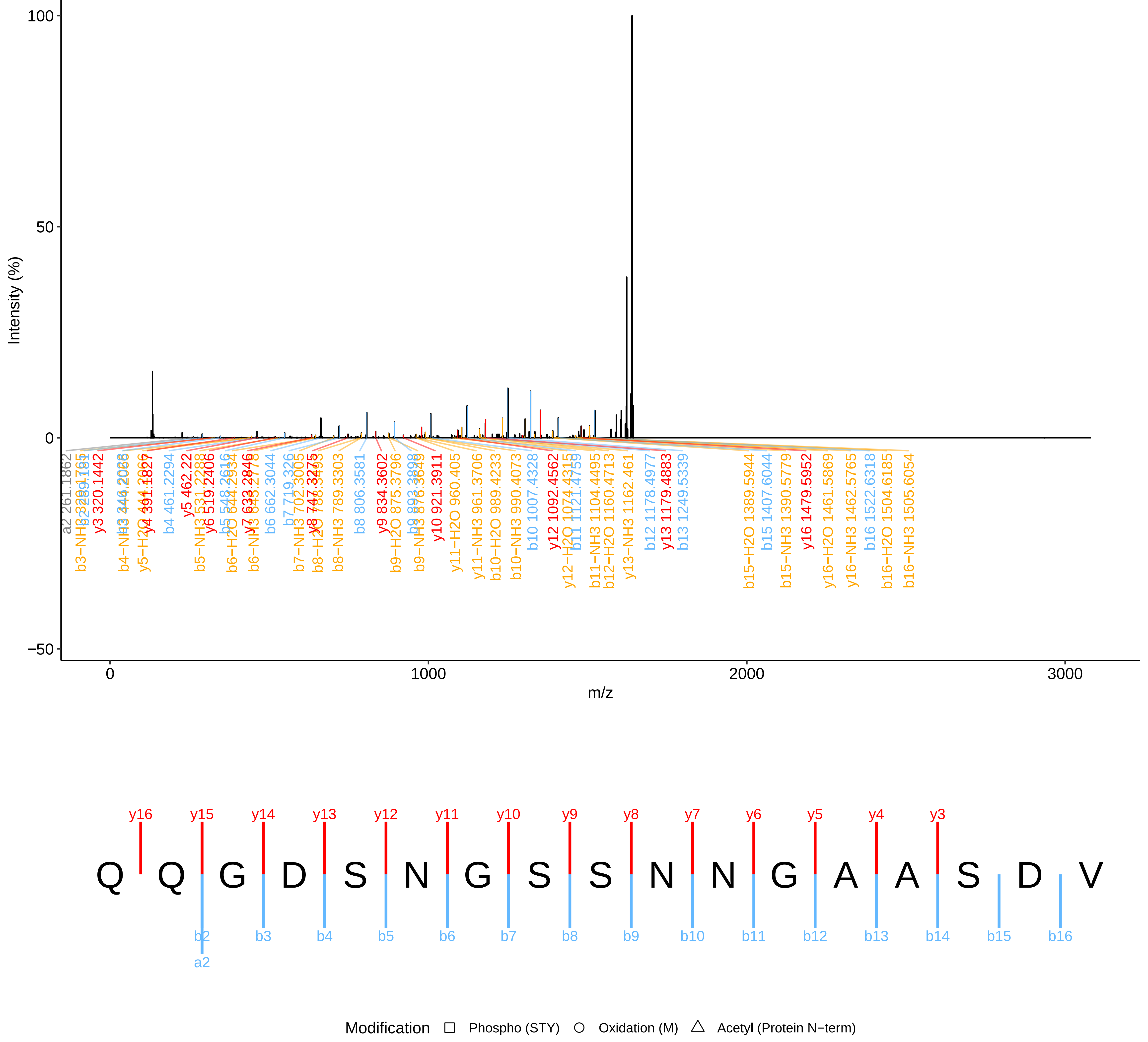

20211212_PS_SCy031_proteome_R1_HC_F4 − Scan number: 30857

Gene Names	Charge	m/z	Mass	Mass error [Da]	Mass error [ppm]	Retention time	PEP	Score	Precursor Intensity
ssr3307	2	877.998	1753.981	−0.0010512	−1.1966	44.758	1.9658e−64	225.21	NA

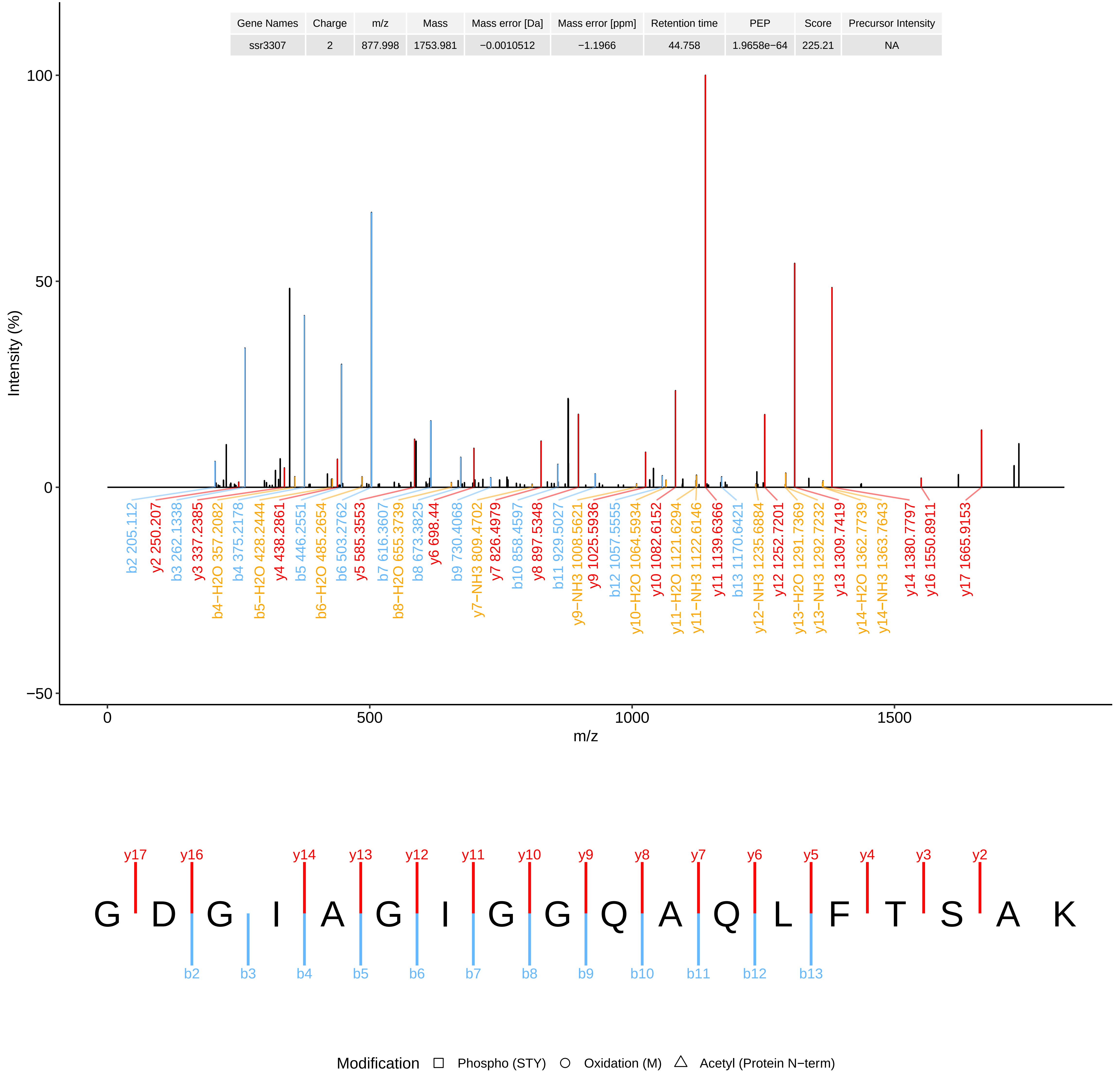

20211212_PS_SCy031_proteome_R1_HC_F2 − Scan number: 38444

Gene Names	Charge	m/z	Mass	Mass error [Da]	Mass error [ppm]	Retention time	PEP	Score	Precursor Intensity
Norf2	3	565.6347	1693.882	−3.5112e−05	−0.062075	54.053	6.3268e−06	103.79	3144660

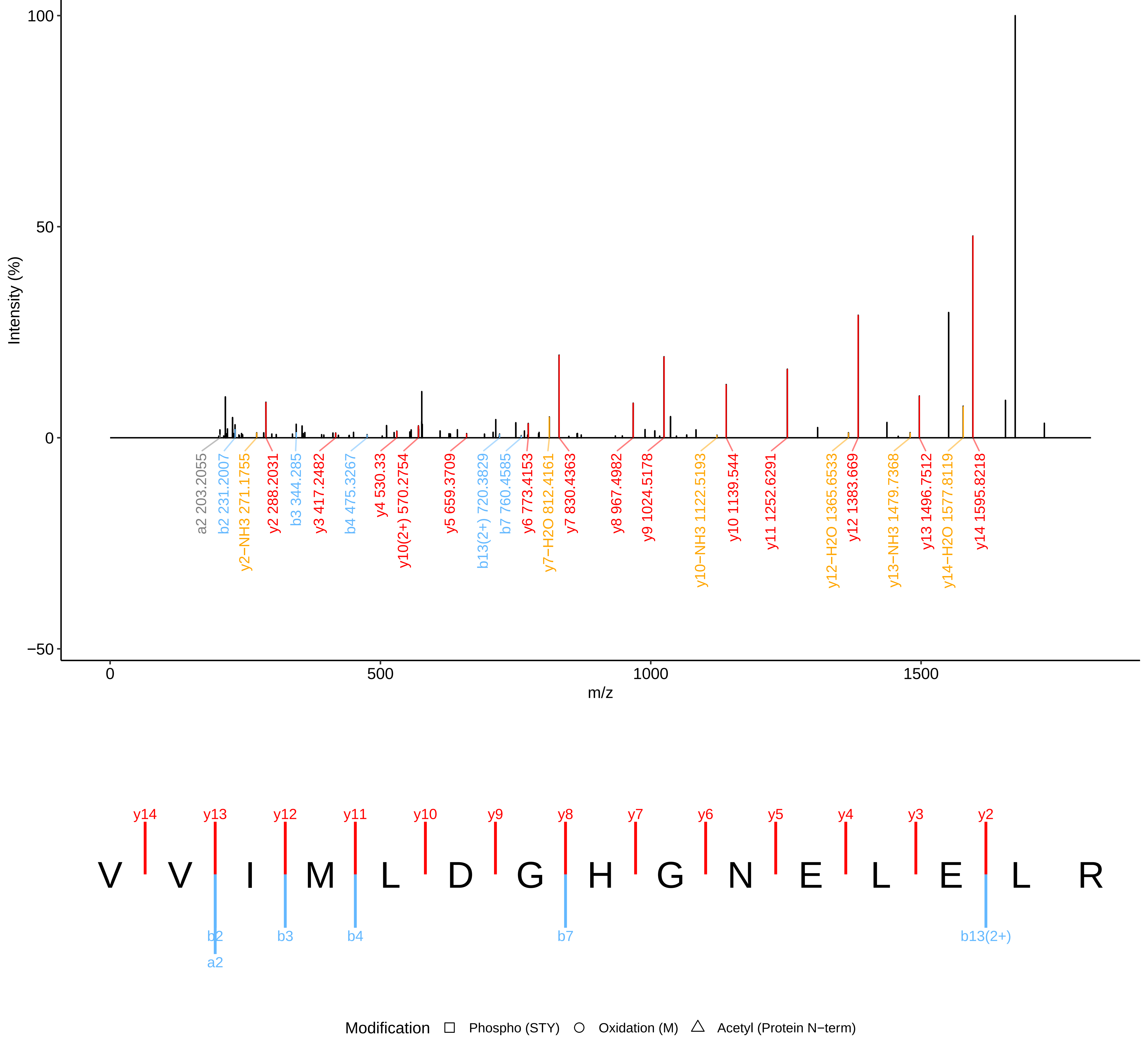

Supplement: Supplemental spectra of single peptide identifications [file mmc4.pdf]
